# Measuring performance on the Healthcare Access and Quality Index for 195 countries and territories and selected subnational locations: a systematic analysis from the Global Burden of Disease Study 2016

**DOI:** 10.1016/S0140-6736(18)30994-2

**Published:** 2018-06-02

**Authors:** GBD 2016 Healthcare Access and Quality Collaborators, Nancy Fullman, Jamal Yearwood, Solomon M Abay, Cristiana Abbafati, Foad Abd-Allah, Jemal Abdela, Ahmed Abdelalim, Zegeye Abebe, Teshome Abuka Abebo, Victor Aboyans, Haftom Niguse Abraha, Daisy M X Abreu, Laith J Abu-Raddad, Akilew Awoke Adane, Rufus Adesoji Adedoyin, Olatunji Adetokunboh, Tara Ballav Adhikari, Mohsen Afarideh, Ashkan Afshin, Gina Agarwal, Dominic Agius, Anurag Agrawal, Sutapa Agrawal, Aliasghar Ahmad Kiadaliri, Miloud Taki Eddine Aichour, Mohammed Akibu, Rufus Olusola Akinyemi, Tomi F Akinyemiju, Nadia Akseer, Faris Hasan Al Lami, Fares Alahdab, Ziyad Al-Aly, Khurshid Alam, Tahiya Alam, Deena Alasfoor, Mohammed I Albittar, Kefyalew Addis Alene, Ayman Al-Eyadhy, Syed Danish Ali, Mehran Alijanzadeh, Syed M Aljunid, Ala'a Alkerwi, François Alla, Peter Allebeck, Christine Allen, Mahmoud A Alomari, Rajaa Al-Raddadi, Ubai Alsharif, Khalid A Altirkawi, Nelson Alvis-Guzman, Azmeraw T Amare, Kebede Amenu, Walid Ammar, Yaw Ampem Amoako, Nahla Anber, Catalina Liliana Andrei, Sofia Androudi, Carl Abelardo T Antonio, Valdelaine E M Araújo, Olatunde Aremu, Johan Ärnlöv, Al Artaman, Krishna Kumar Aryal, Hamid Asayesh, Ephrem Tsegay Asfaw, Solomon Weldegebreal Asgedom, Rana Jawad Asghar, Mengistu Mitiku Ashebir, Netsanet Abera Asseffa, Tesfay Mehari Atey, Sachin R Atre, Madhu S Atteraya, Leticia Avila-Burgos, Euripide Frinel G Arthur Avokpaho, Ashish Awasthi, Beatriz Paulina Ayala Quintanilla, Animut Alebel Ayalew, Henok Tadesse Ayele, Rakesh Ayer, Tambe Betrand Ayuk, Peter Azzopardi, Natasha Azzopardi-Muscat, Tesleem Kayode Babalola, Hamid Badali, Alaa Badawi, Maciej Banach, Amitava Banerjee, Amrit Banstola, Ryan M Barber, Miguel A Barboza, Suzanne L Barker-Collo, Till Bärnighausen, Simon Barquera, Lope H Barrero, Quique Bassat, Sanjay Basu, Bernhard T Baune, Shahrzad Bazargan-Hejazi, Neeraj Bedi, Ettore Beghi, Masoud Behzadifar, Meysam Behzadifar, Bayu Begashaw Bekele, Abate Bekele Belachew, Saba Abraham Belay, Yihalem Abebe Belay, Michelle L Bell, Aminu K Bello, Derrick A Bennett, James R Bennett, Isabela M Bensenor, Derbew Fikadu Berhe, Eduardo Bernabé, Robert Steven Bernstein, Mircea Beuran, Ashish Bhalla, Paurvi Bhatt, Soumyadeep Bhaumik, Zulfiqar A Bhutta, Belete Biadgo, Ali Bijani, Boris Bikbov, Charles Birungi, Stan Biryukov, Hailemichael Bizuneh, Ian W Bolliger, Kaylin Bolt, Ibrahim R Bou-Orm, Kayvan Bozorgmehr, Oliver Jerome Brady, Alexandra Brazinova, Nicholas J K Breitborde, Hermann Brenner, Gabrielle Britton, Traolach S Brugha, Zahid A Butt, Lucero Cahuana-Hurtado, Ismael Ricardo Campos-Nonato, Julio Cesar Campuzano, Josip Car, Mate Car, Rosario Cárdenas, Juan Jesus Carrero, Felix Carvalho, Carlos A Castañeda-Orjuela, Jacqueline Castillo Rivas, Ferrán Catalá-López, Kelly Cercy, Julian Chalek, Hsing-Yi Chang, Jung-Chen Chang, Aparajita Chattopadhyay, Pankaj Chaturvedi, Peggy Pei-Chia Chiang, Vesper Hichilombwe Chisumpa, Jee-Young J Choi, Hanne Christensen, Devasahayam Jesudas Christopher, Sheng-Chia Chung, Liliana G Ciobanu, Massimo Cirillo, Danny Colombara, Sara Conti, Cyrus Cooper, Leslie Cornaby, Paolo Angelo Cortesi, Monica Cortinovis, Alexandre Costa Pereira, Ewerton Cousin, Michael H Criqui, Elizabeth A Cromwell, Christopher Stephen Crowe, John A Crump, Alemneh Kabeta Daba, Berihun Assefa Dachew, Abel Fekadu Dadi, Lalit Dandona, Rakhi Dandona, Paul I Dargan, Ahmad Daryani, Maryam Daryani, Jai Das, Siddharth Kumar Das, José das Neves, Nicole Davis Weaver, Kairat Davletov, Barbora de Courten, Diego De Leo, Jan-Walter De Neve, Robert P Dellavalle, Gebre Demoz, Kebede Deribe, Don C Des Jarlais, Subhojit Dey, Samath D Dharmaratne, Meghnath Dhimal, Shirin Djalalinia, David Teye Doku, Kate Dolan, E Ray Dorsey, Kadine Priscila Bender dos Santos, Kerrie E Doyle, Tim R Driscoll, Manisha Dubey, Eleonora Dubljanin, Bruce Bartholow Duncan, Michelle Echko, Dumessa Edessa, David Edvardsson, Joshua R Ehrlich, Erika Eldrenkamp, Ziad Ziad El-Khatib, Matthias Endres, Aman Yesuf Endries, Babak Eshrati, Sharareh Eskandarieh, Alireza Esteghamati, Mahdi Fakhar, Tamer Farag, Mahbobeh Faramarzi, Emerito Jose Aquino Faraon, André Faro, Farshad Farzadfar, Adesegun Fatusi, Mir Sohail Fazeli, Valery L Feigin, Andrea B Feigl, Netsanet Fentahun, Seyed-Mohammad Fereshtehnejad, Eduarda Fernandes, João C Fernandes, Daniel Obadare Fijabi, Irina Filip, Florian Fischer, Christina Fitzmaurice, Abraham D Flaxman, Luisa Sorio Flor, Nataliya Foigt, Kyle J Foreman, Joseph J Frostad, Thomas Fürst, Neal D Futran, Emmanuela Gakidou, Silvano Gallus, Ketevan Gambashidze, Amiran Gamkrelidze, Morsaleh Ganji, Abadi Kahsu Gebre, Tsegaye Tewelde Gebrehiwot, Amanuel Tesfay Gebremedhin, Yalemzewod Assefa Gelaw, Johanna M Geleijnse, Demeke Geremew, Peter W Gething, Reza Ghadimi, Khalil Ghasemi Falavarjani, Maryam Ghasemi-Kasman, Paramjit Singh Gill, Ababi Zergaw Giref, Maurice Giroud, Melkamu Dedefo Gishu, Giorgia Giussani, William W Godwin, Srinivas Goli, Hector Gomez-Dantes, Philimon N Gona, Amador Goodridge, Sameer Vali Gopalani, Yevgeniy Goryakin, Alessandra Carvalho Goulart, Ayman Grada, Max Griswold, Giuseppe Grosso, Harish Chander Gugnani, Yuming Guo, Rahul Gupta, Rajeev Gupta, Tanush Gupta, Tarun Gupta, Vipin Gupta, Juanita A Haagsma, Vladimir Hachinski, Nima Hafezi-Nejad, Gessessew Bugssa Hailu, Randah Ribhi Hamadeh, Samer Hamidi, Graeme J Hankey, Hilda L Harb, Heather C Harewood, Sivadasanpillai Harikrishnan, Josep Maria Haro, Hamid Yimam Hassen, Rasmus Havmoeller, Caitlin Hawley, Simon I Hay, Jiawei He, Stephen J C Hearps, Mohamed I Hegazy, Behzad Heibati, Mohsen Heidari, Delia Hendrie, Nathaniel J Henry, Victor Hugo Herrera Ballesteros, Claudiu Herteliu, Desalegn Tsegaw Hibstu, Molla Kahssay Hiluf, Hans W Hoek, Enayatollah Homaie Rad, Nobuyuki Horita, H Dean Hosgood, Mostafa Hosseini, Seyed Reza Hosseini, Mihaela Hostiuc, Sorin Hostiuc, Damian G Hoy, Mohamed Hsairi, Aung Soe Htet, Guoqing Hu, John J Huang, Kim Moesgaard Iburg, Fachmi Idris, Ehimario Uche Igumbor, Chad Ikeda, Bogdan Vasile Ileanu, Olayinka S Ilesanmi, Kaire Innos, Seyed Sina Naghibi Irvani, Caleb M S Irvine, Farhad Islami, Troy A Jacobs, Kathryn H Jacobsen, Nader Jahanmehr, Rajesh Jain, Sudhir Kumar Jain, Mihajlo B Jakovljevic, Moti Tolera Jalu, Amr A Jamal, Mehdi Javanbakht, Achala Upendra Jayatilleke, Panniyammakal Jeemon, Ravi Prakash Jha, Vivekanand Jha, Jacek Jóúwiak, Oommen John, Sarah Charlotte Johnson, Jost B Jonas, Vasna Joshua, Mikk Jürisson, Zubair Kabir, Rajendra Kadel, Amaha Kahsay, Rizwan Kalani, Chittaranjan Kar, Marina Karanikolos, André Karch, Corine Kakizi Karema, Seyed M Karimi, Amir Kasaeian, Dessalegn Haile Kassa, Getachew Mullu Kassa, Tesfaye Dessale Kassa, Nicholas J Kassebaum, Srinivasa Vittal Katikireddi, Anil Kaul, Norito Kawakami, Konstantin Kazanjan, Seifu Kebede, Peter Njenga Keiyoro, Grant Rodgers Kemp, Andre Pascal Kengne, Maia Kereselidze, Ezra Belay Ketema, Yousef Saleh Khader, Morteza Abdullatif Khafaie, Alireza Khajavi, Ibrahim A Khalil, Ejaz Ahmad Khan, Gulfaraz Khan, Md Nuruzzaman Khan, Muhammad Ali Khan, Mukti Nath Khanal, Young-Ho Khang, Mona M Khater, Abdullah Tawfih Abdullah Khoja, Ardeshir Khosravi, Jagdish Khubchandani, Getiye Dejenu Kibret, Daniel Ngari Kiirithio, Daniel Kim, Yun Jin Kim, Ruth W Kimokoti, Yohannes Kinfu, Sanjay Kinra, Adnan Kisa, Niranjan Kissoon, Sonali Kochhar, Yoshihiro Kokubo, Jacek A Kopec, Soewarta Kosen, Parvaiz A Koul, Ai Koyanagi, Michael Kravchenko, Kewal Krishan, Kristopher J Krohn, Barthelemy Kuate Defo, G Anil Kumar, Pushpendra Kumar, Michael Kutz, Igor Kuzin, Hmwe H Kyu, Deepesh Pravinkumar Lad, Alessandra Lafranconi, Dharmesh Kumar Lal, Ratilal Lalloo, Hilton Lam, Qing Lan, Justin J Lang, Van C Lansingh, Sonia Lansky, Anders Larsson, Arman Latifi, Jeffrey Victor Lazarus, Janet L Leasher, Paul H Lee, Yirga Legesse, James Leigh, Cheru Tesema Leshargie, Samson Leta, Janni Leung, Ricky Leung, Miriam Levi, Yongmei Li, Juan Liang, Misgan Legesse Liben, Lee-Ling Lim, Stephen S Lim, Margaret Lind, Shai Linn, Stefan Listl, Patrick Liu, Shiwei Liu, Rakesh Lodha, Alan D Lopez, Scott A Lorch, Stefan Lorkowski, Paulo A Lotufo, Timothy C D Lucas, Raimundas Lunevicius, Grégoire Lurton, Ronan A Lyons, Fadi Maalouf, Erlyn Rachelle King Macarayan, Mark T Mackay, Emilie R Maddison, Fabiana Madotto, Hassan Magdy Abd El Razek, Mohammed Magdy Abd El Razek, Marek Majdan, Reza Majdzadeh, Azeem Majeed, Reza Malekzadeh, Rajesh Malhotra, Deborah Carvalho Malta, Abdullah A Mamun, Trey Manhertz, Helena Manguerra, Mohammad Ali Mansournia, Lorenzo G Mantovani, Tsegahun Manyazewal, Chabila C Mapoma, Christopher Margono, Jose Martinez-Raga, Sheila Cristina Ouriques Martins, Francisco Rogerlândio Martins-Melo, Ira Martopullo, Winfried März, Benjamin Ballard Massenburg, Manu Raj Mathur, Pallab K Maulik, Mohsen Mazidi, Colm McAlinden, John J McGrath, Martin McKee, Suresh Mehata, Ravi Mehrotra, Kala M Mehta, Varshil Mehta, Toni Meier, Fabiola Mejia-Rodriguez, Kidanu Gebremariam Meles, Mulugeta Melku, Peter Memiah, Ziad A Memish, Walter Mendoza, Degu Abate Mengiste, Desalegn Tadese Mengistu, Bereket Gebremichael Menota, George A Mensah, Atte Meretoja, Tuomo J Meretoja, Haftay Berhane Mezgebe, Tomasz Miazgowski, Renata Micha, Robert Milam, Anoushka Millear, Ted R Miller, GK Mini, Shawn Minnig, Andreea Mirica, Erkin M Mirrakhimov, Awoke Misganaw, Philip B Mitchell, Fitsum Weldegebreal Mlashu, Babak Moazen, Karzan Abdulmuhsin Mohammad, Roghayeh Mohammadibakhsh, Ebrahim Mohammed, Mohammed A Mohammed, Shafiu Mohammed, Ali H Mokdad, Glen Liddell Mola, Mariam Molokhia, Fatemeh Momeniha, Lorenzo Monasta, Julio Cesar Montañez Hernandez, Mahmood Moosazadeh, Maziar Moradi-Lakeh, Paula Moraga, Lidia Morawska, Ilais Moreno Velasquez, Rintaro Mori, Shane D Morrison, Mark Moses, Seyyed Meysam Mousavi, Ulrich O Mueller, Manoj Murhekar, Gudlavalleti Venkata Satyanarayana Murthy, Srinivas Murthy, Jonah Musa, Kamarul Imran Musa, Ghulam Mustafa, Saravanan Muthupandian, Chie Nagata, Gabriele Nagel, Mohsen Naghavi, Aliya Naheed, Gurudatta A Naik, Nitish Naik, Farid Najafi, Luigi Naldi, Vinay Nangia, Jobert Richie Njingang Nansseu, KM Venkat Narayan, Bruno Ramos Nascimento, Ionut Negoi, Ruxandra Irina Negoi, Charles R Newton, Josephine Wanjiku Ngunjiri, Grant Nguyen, Long Nguyen, Trang Huyen Nguyen, Emma Nichols, Dina Nur Anggraini Ningrum, Ellen Nolte, Vuong Minh Nong, Ole F Norheim, Bo Norrving, Jean Jacques N Noubiap, Alypio Nyandwi, Carla Makhlouf Obermeyer, Richard Ofori-Asenso, Felix Akpojene Ogbo, In-Hwan Oh, Olanrewaju Oladimeji, Andrew Toyin Olagunju, Tinuke Oluwasefunmi Olagunju, Pedro R Olivares, Patricia Pereira Vasconcelos de Oliveira, Helen E Olsen, Bolajoko Olubukunola Olusanya, Jacob Olusegun Olusanya, Kanyin Ong, John Nelson Opio, Eyal Oren, Doris V Ortega-Altamirano, Alberto Ortiz, Raziye Ozdemir, Mahesh PA, Amanda W Pain, Marcos Roberto Tovani Palone, Adrian Pana, Songhomitra Panda-Jonas, Jeyaraj D Pandian, Eun-Kee Park, Hadi Parsian, Tejas Patel, Sanghamitra Pati, Snehal T Patil, Ajay Patle, George C Patton, Vishnupriya Rao Paturi, Deepak Paudel, Marcel de Moares Pedroso, Sandra P Pedroza, David M Pereira, Norberto Perico, Hannah Peterson, Max Petzold, Niloofar Peykari, Michael Robert Phillips, Frédéric B Piel, David M Pigott, Julian David Pillay, Michael A Piradov, Suzanne Polinder, Constance D Pond, Maarten J Postma, Farshad Pourmalek, Swayam Prakash, V Prakash, Narayan Prasad, Noela Marie Prasad, Caroline Purcell, Mostafa Qorbani, Hedley Knewjen Quintana, Amir Radfar, Anwar Rafay, Alireza Rafiei, Kazem Rahimi, Afarin Rahimi-Movaghar, Vafa Rahimi-Movaghar, Mahfuzar Rahman, Muhammad Aziz Rahman, Sajjad Ur Rahman, Rajesh Kumar Rai, Sree Bhushan Raju, Usha Ram, Saleem M Rana, Zane Rankin, Davide Rasella, David Laith Rawaf, Salman Rawaf, Sarah E Ray, Christian Aspacia Razo-García, Priscilla Reddy, Robert C Reiner, Cesar Reis, Marissa B Reitsma, Giuseppe Remuzzi, Andre M N Renzaho, Serge Resnikoff, Satar Rezaei, Mohammad Sadegh Rezai, Antonio L Ribeiro, Maria Jesus Rios Blancas, Juan A Rivera, Leonardo Roever, Luca Ronfani, Gholamreza Roshandel, Ali Rostami, Gregory A Roth, Dietrich Rothenbacher, Ambuj Roy, Nobhojit Roy, George Mugambage Ruhago, Yogesh Damodar Sabde, Perminder S Sachdev, Nafis Sadat, Mahdi Safdarian, Saeid Safiri, Rajesh Sagar, Amirhossein Sahebkar, Mohammad Ali Sahraian, Haniye Sadat Sajadi, Joseph Salama, Payman Salamati, Raphael de Freitas Saldanha, Hamideh Salimzadeh, Joshua A Salomon, Abdallah M Samy, Juan Ramon Sanabria, Parag K Sancheti, Maria Dolores Sanchez-Niño, Damian Santomauro, Itamar S Santos, Milena M Santric Milicevic, Abdur Razzaque Sarker, Nizal Sarrafzadegan, Benn Sartorius, Maheswar Satpathy, Miloje Savic, Monika Sawhney, Sonia Saxena, Mete I Saylan, Elke Schaeffner, Josef Schmidhuber, Maria Inês Schmidt, Ione J C Schneider, Austin E Schumacher, Aletta E Schutte, David C Schwebel, Falk Schwendicke, Mario Sekerija, Sadaf G Sepanlou, Edson E Servan-Mori, Azadeh Shafieesabet, Masood Ali Shaikh, Marina Shakh-Nazarova, Mehran Shams-Beyranvand, Heidar Sharafi, Mahdi Sharif-Alhoseini, Sheikh Mohammed Shariful Islam, Meenakshi Sharma, Rajesh Sharma, Jun She, Aziz Sheikh, Mebrahtu Teweldemedhin Shfare, Peilin Shi, Chloe Shields, Mika Shigematsu, Yukito Shinohara, Rahman Shiri, Reza Shirkoohi, Ivy Shiue, Mark G Shrime, Sharvari Rahul Shukla, Soraya Siabani, Inga Dora Sigfusdottir, Donald H Silberberg, Diego Augusto Santos Silva, João Pedro Silva, Dayane Gabriele Alves Silveira, Jasvinder A Singh, Lavanya Singh, Narinder Pal Singh, Virendra Singh, Dhirendra Narain Sinha, Abiy Hiruye Sinke, Mekonnen Sisay, Vegard Skirbekk, Karen Sliwa, Alison Smith, Adauto Martins Soares Filho, Badr H A Sobaih, Melek Somai, Samir Soneji, Moslem Soofi, Reed J D Sorensen, Joan B Soriano, Ireneous N Soyiri, Luciano A Sposato, Chandrashekhar T Sreeramareddy, Vinay Srinivasan, Jeffrey D Stanaway, Vasiliki Stathopoulou, Nicholas Steel, Dan J Stein, Mark Andrew Stokes, Lela Sturua, Muawiyyah Babale Sufiyan, Rizwan Abdulkader Suliankatchi, Bruno F Sunguya, Patrick J Sur, Bryan L Sykes, PN Sylaja, Rafael Tabarés-Seisdedos, Santosh Kumar Tadakamadla, Andualem Henok Tadesse, Getachew Redae Taffere, Nikhil Tandon, Amare Tariku Tariku, Nuno Taveira, Arash Tehrani-Banihashemi, Girma Temam Shifa, Mohamad-Hani Temsah, Abdullah Sulieman Terkawi, Azeb Gebresilassie Tesema, Dawit Jember Tesfaye, Belay Tessema, JS Thakur, Nihal Thomas, Matthew J Thompson, Taavi Tillmann, Quyen G To, Ruoyan Tobe-Gai, Marcello Tonelli, Roman Topor-Madry, Fotis Topouzis, Anna Torre, Miguel Tortajada, Bach Xuan Tran, Khanh Bao Tran, Avnish Tripathi, Srikanth Prasad Tripathy, Christopher Troeger, Thomas Truelsen, Derrick Tsoi, Lorainne Tudor Car, Kald Beshir Tuem, Stefanos Tyrovolas, Uche S Uchendu, Kingsley N Ukwaja, Irfan Ullah, Rachel Updike, Olalekan A Uthman, Benjamin S Chudi Uzochukwu, Pascual Rubén Valdez, Job F M van Boven, Santosh Varughese, Tommi Vasankari, Francesco S Violante, Sergey K Vladimirov, Vasiliy Victorovich Vlassov, Stein Emil Vollset, Theo Vos, Fasil Wagnew, Yasir Waheed, Mitchell T Wallin, Judd L Walson, Yafeng Wang, Yuan-Pang Wang, Molla Mesele Wassie, Marcia R Weaver, Elisabete Weiderpass, Robert G Weintraub, Jordan Weiss, Kidu Gidey Weldegwergs, Andrea Werdecker, T Eoin West, Ronny Westerman, Richard G White, Harvey A Whiteford, Justyna Widecka, Andrea Sylvia Winkler, Charles Shey Wiysonge, Charles DA Wolfe, Yohanes Ayele Wondimkun, Abdulhalik Workicho, Grant M A Wyper, Denis Xavier, Gelin Xu, Lijing L Yan, Yuichiro Yano, Mehdi Yaseri, Nigus Bililign Yimer, Peng Yin, Paul Yip, Biruck Desalegn Yirsaw, Naohiro Yonemoto, Gerald Yonga, Seok-Jun Yoon, Marcel Yotebieng, Mustafa Z Younis, Chuanhua Yu, Vesna Zadnik, Zoubida Zaidi, Maysaa El Sayed Zaki, Sojib Bin Zaman, Mohammad Zamani, Zerihun Menlkalew Zenebe, Maigeng Zhou, Jun Zhu, Stephanie R M Zimsen, Ben Zipkin, Sanjay Zodpey, Liesl Joanna Zuhlke, Christopher J L Murray, Rafael Lozano

## Abstract

**Background:**

A key component of achieving universal health coverage is ensuring that all populations have access to quality health care. Examining where gains have occurred or progress has faltered across and within countries is crucial to guiding decisions and strategies for future improvement. We used the Global Burden of Diseases, Injuries, and Risk Factors Study 2016 (GBD 2016) to assess personal health-care access and quality with the Healthcare Access and Quality (HAQ) Index for 195 countries and territories, as well as subnational locations in seven countries, from 1990 to 2016.

**Methods:**

Drawing from established methods and updated estimates from GBD 2016, we used 32 causes from which death should not occur in the presence of effective care to approximate personal health-care access and quality by location and over time. To better isolate potential effects of personal health-care access and quality from underlying risk factor patterns, we risk-standardised cause-specific deaths due to non-cancers by location-year, replacing the local joint exposure of environmental and behavioural risks with the global level of exposure. Supported by the expansion of cancer registry data in GBD 2016, we used mortality-to-incidence ratios for cancers instead of risk-standardised death rates to provide a stronger signal of the effects of personal health care and access on cancer survival. We transformed each cause to a scale of 0–100, with 0 as the first percentile (worst) observed between 1990 and 2016, and 100 as the 99th percentile (best); we set these thresholds at the country level, and then applied them to subnational locations. We applied a principal components analysis to construct the HAQ Index using all scaled cause values, providing an overall score of 0–100 of personal health-care access and quality by location over time. We then compared HAQ Index levels and trends by quintiles on the Socio-demographic Index (SDI), a summary measure of overall development. As derived from the broader GBD study and other data sources, we examined relationships between national HAQ Index scores and potential correlates of performance, such as total health spending per capita.

**Findings:**

In 2016, HAQ Index performance spanned from a high of 97·1 (95% UI 95·8–98·1) in Iceland, followed by 96·6 (94·9–97·9) in Norway and 96·1 (94·5–97·3) in the Netherlands, to values as low as 18·6 (13·1–24·4) in the Central African Republic, 19·0 (14·3–23·7) in Somalia, and 23·4 (20·2–26·8) in Guinea-Bissau. The pace of progress achieved between 1990 and 2016 varied, with markedly faster improvements occurring between 2000 and 2016 for many countries in sub-Saharan Africa and southeast Asia, whereas several countries in Latin America and elsewhere saw progress stagnate after experiencing considerable advances in the HAQ Index between 1990 and 2000. Striking subnational disparities emerged in personal health-care access and quality, with China and India having particularly large gaps between locations with the highest and lowest scores in 2016. In China, performance ranged from 91·5 (89·1–93·6) in Beijing to 48·0 (43·4–53·2) in Tibet (a 43·5-point difference), while India saw a 30·8-point disparity, from 64·8 (59·6–68·8) in Goa to 34·0 (30·3–38·1) in Assam. Japan recorded the smallest range in subnational HAQ performance in 2016 (a 4·8-point difference), whereas differences between subnational locations with the highest and lowest HAQ Index values were more than two times as high for the USA and three times as high for England. State-level gaps in the HAQ Index in Mexico somewhat narrowed from 1990 to 2016 (from a 20·9-point to 17·0-point difference), whereas in Brazil, disparities slightly increased across states during this time (a 17·2-point to 20·4-point difference). Performance on the HAQ Index showed strong linkages to overall development, with high and high-middle SDI countries generally having higher scores and faster gains for non-communicable diseases. Nonetheless, countries across the development spectrum saw substantial gains in some key health service areas from 2000 to 2016, most notably vaccine-preventable diseases. Overall, national performance on the HAQ Index was positively associated with higher levels of total health spending per capita, as well as health systems inputs, but these relationships were quite heterogeneous, particularly among low-to-middle SDI countries.

**Interpretation:**

GBD 2016 provides a more detailed understanding of past success and current challenges in improving personal health-care access and quality worldwide. Despite substantial gains since 2000, many low-SDI and middle-SDI countries face considerable challenges unless heightened policy action and investments focus on advancing access to and quality of health care across key health services, especially non-communicable diseases. Stagnating or minimal improvements experienced by several low-middle to high-middle SDI countries could reflect the complexities of re-orienting both primary and secondary health-care services beyond the more limited foci of the Millennium Development Goals. Alongside initiatives to strengthen public health programmes, the pursuit of universal health coverage hinges upon improving both access and quality worldwide, and thus requires adopting a more comprehensive view—and subsequent provision—of quality health care for all populations.

**Funding:**

Bill & Melinda Gates Foundation.

## Introduction

Providing access to quality health care is among the foremost objectives of health systems,^1^, ^2^ because the receipt of effective personal health care can substantially improve many health outcomes and avert premature mortality. The advancement of population health was elevated to global agendas with the Alma Ata Declaration of 1978, wherein WHO called for the achievement of “health for all” by 2000.^3^ Such aspirations garnered new momentum in the Sustainable Development Goal (SDG) era,^4^ with a heightened emphasis on attaining universal health coverage in this pursuit. Making progress on universal health coverage entails all people having access to quality health services they need without incurring financial hardship.^5^ To advance toward this ambition, it is crucial to monitor where improvements in health-care access and quality have occurred, and where progress must be accelerated, across the development spectrum.

**Research in context**

**Evidence before this study**

Improving, and subsequently measuring, health-care access and quality has emerged as an increasing priority alongside a heightened emphasis on universal health coverage in the Sustainable Development Goal era. Nevertheless, few studies have sought to assess personal health-care access and quality across a wide range of key health service dimensions and the development spectrum. Primarily focused on high-income countries, past analyses have used amenable mortality—deaths from causes that should not occur in the presence of high-quality health care—to approximate national levels of personal health-care access and quality. Drawing from the Global Burden of Diseases, Injuries, and Risk Factors Study 2015 (GBD 2015), the GBD collaboration used this amenable mortality framework in developing the Healthcare Access and Quality (HAQ) Index, and subsequently offered several advances from previous work. First, the extensive cause-of-death standardisation processes that occur as part of GBD enabled better comparisons across locations and over time. Second, risk-standardising death rates for environmental and behavioural risk factors helped isolate differences in health-care access and quality from variations in death rates due to background risk exposure. Third, estimating the HAQ Index for 195 countries and territories from 1990 to 2015, allowed for a broader investigation of trends in personal health-care access and quality across the development spectrum. Despite these methodological strengths, additional areas for improvement were identified, including the consideration of health outcomes that more directly reflect the progression of disease onset to mortality for amenable causes and examining subnational inequalities.

**Added value of this study**

Based on updated cause of death and risk factor estimates from the GBD 2016 study, our analysis offers an improved assessment of national levels of personal health-care access and quality from 1990 to 2016. For the first time, we report subnational levels and trends on the HAQ Index for seven countries: Brazil, China, England, India, Japan, Mexico, and the USA. Because of major improvements in cancer estimation and data availability, we used mortality-to-incidence ratios rather than risk-standardised death rates from cancer, ultimately providing a more robust approximation of cancer detection and treatment effects across countries. To improve index stability, we used percentiles (ie, first and 99th percentile) for transforming HAQ Index components to a scale of 0–100. Finally, we did an exploratory analysis of national HAQ Index levels and potential correlates of performance, examining relationships between the HAQ Index and some indicators such as health financing (eg, total health spending per capita).

**Implications of all the available evidence**

Globally, personal health-care access and quality improved since 1990, with many countries in sub-Saharan Africa and southeast Asia accelerating their pace of progress from 2000 to 2016. Such gains in the more recent time period could reflect the catalytic effects of the Millennium Development Goals and their focus on a subset of health service areas (ie, vaccine-preventable diseases, infectious diseases, and maternal and child health). Nonetheless, inequalities increased in some parts of the world, which might be related to many low-to-middle income countries recording much slower gains for cancers and other non-communicable diseases. Large disparities in subnational levels of personal health-care access and quality emerged for several countries, especially China and India. These results emphasise the urgent need to improve both access to and quality of health care across service areas and for all populations; otherwise, health systems could face widening gaps between the health services they provide and the disease burden experienced by local communities. Going forward, the HAQ Index can provide a robust measure for both informing and monitoring the effects of policy action on health-care access and quality, a key component of achieving universal health coverage. To deliver health systems for the next generation and hasten progress in the Sustainable Development Goal era, now is the time to align investments for improving access and quality across the full range of health-care needs.

Measuring health-care access and quality has become an increasingly important priority alongside its ascent in global health policy. In particular, the use of amenable mortality—deaths from causes that should not occur in the presence of effective medical care—to approximate national levels of personal health-care access and quality has gained greater traction.^6^, ^7^, ^8^, ^9^, ^10^, ^11^, ^12^, ^13^, ^14^, ^15^ Amenable mortality metrics are thought to provide a strong signal of what can or should be addressed by the receipt of effective health care, and thus performance on overall personal health-care access and quality. Combining such measures with those capturing avertable or preventable health outcomes (ie, burden that can be avoided through public health programmes or policies implemented outside the immediate health sector) can offer a more complete set of potential pathways for improving health.^1^, ^16^ The Nolte and McKee list of causes amenable to health care^6^, ^7^, ^8^, ^9^ remains the most widely used framework to quantify national levels of health-care access and quality on the basis of amenable mortality. This is particularly true for Europe,^11^, ^15^, ^17^ the Organisation for Economic Co-operation and Development (OECD),^12^ and the USA,^13^ but increasingly also for other country-specific analyses (eg, Brazil,^14^ China,^18^ and Mexico^19^). As part of the Global Burden of Diseases, Injuries, and Risk Factors Study 2015 (GBD 2015),^20^ the GBD collaboration applied this framework to develop a novel measure, the Healthcare Access and Quality (HAQ) Index, to track gains and gaps in personal health-care access and quality in 195 countries and territories over time.

The HAQ Index offered several strengths and insights into personal health-care access and quality across countries, which has prompted calls for further improvements. First, 32 causes considered amenable to health care comprise the HAQ Index, representing a range of health service areas: vaccine-preventable diseases; infectious diseases and maternal and child health; non-communicable diseases, including cancers, cardiovascular diseases, and other non-communicable diseases such as diabetes; and gastrointestinal conditions from which surgery can easily avert death (eg, appendicitis). Other than in high-income countries, past research rarely accounts for this array of services,^21^ even though effective preventive interventions, treatment, and medical technologies exist; instead, these studies often focus on infectious diseases and maternal and child health, and do not shed light on potential challenges across service areas. Second, because GBD quantifies risk exposure and risk-attributable deaths, we could account for local variations in risk exposure and better isolate differences in mortality related to health care. Nonetheless, challenges can still exist in ensuring that these measures provide a strong signal on health-care access and quality. For instance, in the absence of stronger monitoring systems, low rates of cancer mortality could actually represent inadequate detection and treatment of cancer rather than good access to cancer screening and high-quality care.^22^ Third, although some insights into the relationship between the HAQ Index and sociodemographic development were explored in GBD 2015,^20^ further examination of how health financing and system measures are related to the HAQ Index has yet to occur. Fourth, considerable debate continues about how well the current cause list represents the range of causes amenable to health care, particularly non-fatal outcomes, as well as the ages at which health care can substantially improve outcomes. Finally, GBD 2015 highlighted sizeable inequalities across countries^20^ but did not capture subnational differences in personal health-care access and quality, a crucial need in light of the magnitude by which health outcomes can vary within countries.^23^, ^24^, ^25^, ^26^, ^27^, ^28^, ^29^, ^30^

In this study, we provide updated estimates from 1990 to 2016 for the HAQ Index in 195 countries and territories, as well as at global and regional levels. For the first time, we report subnational estimates of the HAQ Index for seven countries, allowing for a more in-depth examination of inequalities in personal health-care access and quality. With the improved estimation of cancers in GBD 2016,^31^, ^32^, ^33^ we use mortality-to-incidence ratios (MIRs) for cancers to better reflect potential differences in cancer diagnostic and treatment capacity across locations. Finally, we do an exploratory analysis of the associations between the HAQ Index and potential correlates of performance.

## Methods

### Overview

Drawing from methods established in GBD 2015,^20^ our analysis involved four steps: mapping the Nolte and McKee cause list to GBD causes; constructing MIRs for cancers and risk-standardising non-cancer deaths to remove variations in mortality not directly amenable to health care; calculating the HAQ Index on the basis of principal components analysis (PCA), providing an overall score of personal health-care access and quality on a scale of 0–100; and examining associations between national HAQ Index scores and potential correlates of performance.

Our study draws from GBD 2016 results,^31^, ^32^, ^33^ which entail several improvements since GBD 2015, including 169 new country-years of vital registration data, 528 new cancer-registry years with a total of 92 countries' cancer registries,^31^ five new risk factors,^32^ and cause-specific mortality modelling updates (eg, cancers, tuberculosis).^31^ Further information can be found in the appendix (pp 12–89) and the GBD 2016 capstone series.^31^, ^32^, ^33^

In addition to national and aggregated HAQ Index results, we report estimates at the subnational level for Brazil (26 states and the Federal District), China (33 provinces and special administrative regions), England (nine regions and 150 local government areas), India (31 states and union territories), Japan (47 prefectures), Mexico (32 states), and the USA (50 states and the District of Columbia).

As with all GBD revisions, GBD 2016 HAQ Index estimates for the full time series published here supersede previous iterations. This analysis complies with the Guidelines for Accurate and Transparent Health Estimates Reporting (GATHER);^34^ additional information is found in the appendix (pp 5–7).

### Mapping the Nolte and McKee amenable cause list to GBD causes

We mapped 32 of 33 causes from the Nolte and McKee cause list^6^, ^7^, ^8^, ^9^ to GBD causes in accordance with International Classification of Diseases codes (table 1; appendix p 156). GBD includes thyroid diseases within a larger residual category, and only non-fatal outcomes are estimated for benign prostatic hyperplasia; consequently, these causes were not included in our analyses. GBD provides separate estimates for diphtheria and tetanus, so we disaggregated these causes from the original Nolte and McKee list.

**Table 1:** Causes for which mortality is amenable to health care, mapped to GBD causes, and amenable age range

		**Amenable age range (years)**
**Communicable, maternal, neonatal, and nutritional diseases**		
Tuberculosis		0–74
Diarrhoea, lower respiratory, and other common infectious diseases		
	Diarrhoeal diseases	0–14
	Lower respiratory infections	0–74
	Upper respiratory infections	0–74
	Diphtheria	0–74
	Whooping cough	0–14
	Tetanus	0–74
	Measles	1–14
Maternal disorders		0–74
Neonatal disorders		0–74
**Non-communicable diseases**		
Neoplasms		
	Colon and rectum cancer	0–74
	Non-melanoma skin cancer (squamous-cell carcinoma)	0–74
	Breast cancer	0–74
	Cervical cancer	0–74
	Uterine cancer	0–44
	Testicular cancer	0–74
	Hodgkin's lymphoma	0–74
	Leukaemia	0–44
Cardiovascular diseases		
	Rheumatic heart disease	0–74
	Ischaemic heart disease	0–74
	Cerebrovascular disease	0–74
	Hypertensive heart disease	0–74
Chronic respiratory diseases		1–14
Digestive diseases		
	Peptic ulcer disease	0–74
	Appendicitis	0–74
	Inguinal, femoral, and abdominal hernia	0–74
	Gallbladder and biliary diseases	0–74
Neurological disorders		
	Epilepsy	0–74
Diabetes, urogenital, blood, and endocrine diseases		
	Diabetes	0–49
	Chronic kidney disease	0–74
Other non-communicable diseases		
	Congenital heart anomalies	0–74
**Injuries**		
Unintentional injuries		
	Adverse effects of medical treatment	0–74

Although 0 (at birth) to 1 are listed as the lower bound of age ranges, age restrictions are applied for many causes such that mortality estimates are not produced before a given age group (eg, 15–19 years for many non-communicable diseases). Causes are ordered on the basis of the GBD cause list and corresponding group hierarchies. GBD=Global Burden of Disease.

### Mortality-to-incidence ratios for cancers

GBD cancer mortality estimates are informed by MIRs, which are derived from incidence and mortality data recorded in cancer registries; more detail on MIR estimation is in the appendix (pp 41–49).^31^ MIRs provide a good approximation of cancer survival and have been used to identify countries with higher or lower cancer mortality relative to incidence.^22^, ^35^ Because of the improved quantity and quality of cancer registry data from GBD 2016, we used cancer-specific MIRs instead of risk-standardised death rates. As detailed in the appendix (pp 10–11), cancer-specific MIRs were more strongly correlated with the Socio-demographic Index (SDI), a measure of overall development, than were risk-standardised death rates. These results, and the distribution of MIRs by SDI quintile (appendix pp 96–111), showed that cancer MIRs provide a more robust signal of cancer care access and quality than do risk-standardised death rates.

### Risk-standardisation of death rates for non-cancer causes

To better isolate differences in mortality associated with health-care access and quality from differences associated with underlying risk exposure, we risk-standardised cause-specific deaths to global levels of risk exposure.^32^ We did not risk-standardise differences in exposure to three metabolic risk factors (high systolic blood pressure, high total cholesterol, and high fasting plasma glucose) given their amenability to health care (eg, diagnosis and treatment of hypertension in primary care). For the 24 non-cancer causes, we risk-standardised deaths by removing the joint effects of location-specific behavioural and environmental risk exposure, and replaced these estimates with the global level of joint risk exposure (appendix pp 9–10).

Joint population attributable fraction (PAF) estimation accounts for effects of multiple risks combined, including the mediation of different risk factors through each other. More detail on the PAF calculations and risk-standardisation is provided in the appendix (pp 9–10). Since GBD 2015,^36^ five risk factors were added, most notably low birthweight and short gestation,^32^ which enabled the risk-standardisation of neonatal disorder deaths. Risk-standardised deaths equalled observed deaths for causes in which no risk–outcome pairs have met evidence thresholds for inclusion in GBD (eg, diphtheria, appendicitis).

### Age-standardisation

Using the GBD world population data,^37^ we age-standardised risk-standardised death rates, as well as cancer mortality and incidence estimates, before producing MIRs. We rescaled age weights to equal 1, by cause, a necessary step since included age groups represented a subset of the age groups comprising the world population standard.

### Constructing the HAQ Index

By cause, we log-transformed age-standardised risk-standardised death rates (or MIRs for cancers) and scaled them from 0 to 100 across locations from 1990–2016. Zero was determined by the first percentile observed (ie, highest death rates or MIRs), and 100 was applied to the 99th percentile (ie, lowest death rates or MIRs). This scaling approach differs somewhat from that of GBD 2015,^20^ wherein maximum values determined zero and minimum values set 100. Using a percentile-based approach more closely aligns with other index construction methods used in GBD,^38^ and is less sensitive to outliers or fluctuations in estimates over time. We then applied cause-specific thresholds set by the national level to subnational locations.

We used PCA to construct the HAQ Index on the basis of scaled cause values, resulting in an overall score on a scale of 0–100. The GBD 2016 HAQ Index differed in three main ways from GBD 2015. First, no cause had negative PCA weights (ie, implying that higher death rates were associated with access to higher-quality health care), so all causes contributed to the final index. In GBD 2015, colon and breast cancers had negative PCA weights in the first PCA iteration, so their weights were ultimately set to zero. Second, some cancers had PCA weights more similar to communicable, maternal, and neonatal causes, which meant these causes were weighted more equally (appendix p 157). Finally, we derived PCA weights from country-level estimates and applied them to subnational results; this approach provides greater stability across GBD iterations, particularly as the GBD continues to expand its subnational assessments.

### Examining correlates of HAQ Index performance

The HAQ Index reflects many factors that affect service access and quality across the continuums of care and therapeutic areas, and thus it is challenging to distinguish the unique contribution of access versus quality from other potential drivers.^39^ To provide an initial examination of correlates with HAQ Index performance, we ran Pearson correlations between location-specific HAQ Index values with financial measures (eg, total health spending per capita),^40^ and health system inputs and outputs (eg, outpatient and inpatient utilisation).^33^ We selected these indicators on the basis of data availability in relation to GBD locations, and thus they do not represent all possible correlates.

### Comparing performance on the HAQ Index across the development spectrum

As well as examining global patterns, we report differences in the HAQ Index across levels of development. To do this, we used SDI, a summary measure of overall development based on average income per capita, educational attainment, and total fertility rates.^41^ Countries are grouped by SDI quintiles, as established in GBD 2016, on the basis of their 2016 SDI values.^31^

### Uncertainty analysis

GBD aims to propagate uncertainty throughout its estimation process, which results in uncertainty intervals (UIs) accompanying each estimate. We estimated the HAQ Index for each location-year on the basis of 1000 draws from the posterior distribution for each included cause of death. 95% UIs were based on the 2·5th and 97·5th quantiles of the draws for each measure.

### Role of the funding source

The funder of the study had no role in study design, data collection, data analysis, data interpretation, or writing of the report. The corresponding author had full access to all the data in the study and had final responsibility for the decision to submit for publication.

## Results

### National and subnational patterns in personal health-care access and quality

**Figure 1:**
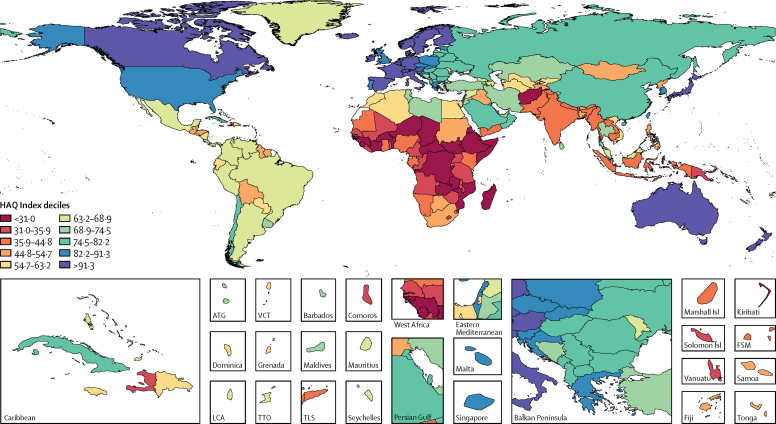
Map of HAQ Index values, by decile, in 2016 Deciles are based on the distribution of HAQ Index values in 2016. Where lower and upper bounds of deciles appear to overlap, they should be interpreted as values up to but not equalling the upper bound in the preceding decile (ie, exclusive of the upper bound value) and values equalling the lower bound of the following decile (ie, inclusive of the lower bound value). HAQ Index=Healthcare Access and Quality Index. ATG=Antigua and Barbuda. VCT=Saint Vincent and the Grenadines. LCA=Saint Lucia. TTO=Trinidad and Tobago. FSM=Federated States of Micronesia. TLS=Timor-Leste.

The HAQ Index performance followed distinct geographical patterns in 2016 (figure 1), with most countries in the highest decile clustered in Europe or nearby (ie, Iceland), and almost all countries in the lowest decile located in sub-Saharan Africa. Exceptions to this pattern included Canada, Japan, Australia, and New Zealand in the tenth decile, and Afghanistan in the first decile. More heterogeneity emerged among the next deciles of performance (eg, USA, UK, Malta, Lebanon, Singapore, and South Korea, in the ninth decile; Cuba, Chile, Saudi Arabia, and Russia, in the eighth decile). Most Latin American countries scored between the fourth and sixth deciles, whereas southeast Asia featured a broader range, spanning from the seventh (Thailand and Sri Lanka) to third deciles (Cambodia, Indonesia, Laos, Myanmar, and Timor-Leste). By 2016, many sub-Saharan African countries improved their performance from 1990 and 2000 (appendix pp 113–14), such as South Africa and Botswana rising to the fourth decile, and several locations moving to the third decile (eg, Kenya, Rwanda, Namibia, Nigeria, Ghana). African countries that remained in the first decile since 1990 were generally concentrated in central and eastern sub-Saharan Africa.

**Figure 2:**
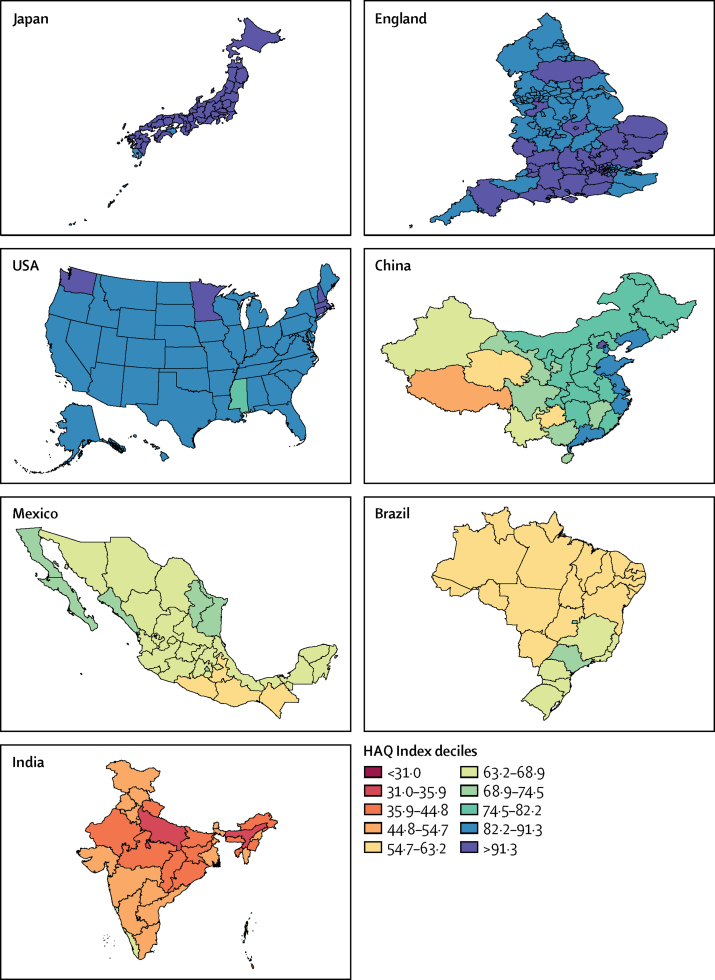
Map of HAQ Index values for selected subnational locations in 2016 Deciles are based on the distribution of HAQ Index values for countries and territories in 2016 (as shown in figure 1), and then applied for subnational locations. Where lower and upper bounds of deciles appear to overlap, they should be interpreted as values up to but not equalling the upper bound in the preceding decile (ie, exclusive of the upper bound value) and values equalling the lower bound of the following decile (ie, inclusive of the lower bound value). HAQ Index=Healthcare Access and Quality Index.

We applied the deciles set by national HAQ Index scores in 2016 to subnational locations (figure 2), and a more nuanced landscape surfaced regarding inequalities in personal health-care access and quality. China was in the eighth decile in 2016, and had provinces spanning from the tenth decile (Beijing 91·5, 95% UI 89·1–93·6) to the fourth decile (Tibet 48·0, 43·5–53·2), with a higher performance (ie, eighth and ninth deciles) among eastern provinces and lower (ie, fifth and sixth deciles) in western provinces. For India, which was in the third decile in 2016, subnational performance ranged from the sixth (Goa 64·8, 59·6–68·8; Kerala 63·9, 58·6–67·0) to the second deciles (Assam 34·0, 30·3–38·1; and Uttar Pradesh 34·9, 31·1–38·4). Brazil and Mexico, each in the sixth decile nationally for 2016, had variable subnational patterns. In Brazil, performance was as high as the eighth decile for the Federal District (75·4, 72·3–78·1), but most states, particularly northern ones, were in the fifth decile. Conversely, Mexico featured six states in the seventh decile, whereas most others were in the sixth decile; four states, all along Mexico's southern border, fell within the fifth decile. Both occupying the ninth decile nationally, England and the USA had subnational locations spanning from the tenth to seventh deciles in 2016; Blackpool (79·7 [76·6–82·8]) had the lowest HAQ Index score in England and Mississippi (81·5 [78·6–84·2]) had the lowest score in the USA. The USA's highest HAQ Index scores were limited to a subset of northeastern states, Minnesota, and Washington state, and higher performance was primarily dispersed across southern England. Nearly all Japanese prefectures occupied the top decile of HAQ Index performance in 2016. The appendix contains a more in-depth exploration of subnational trends over time by country (pp 115–28).

**Figure 3:**
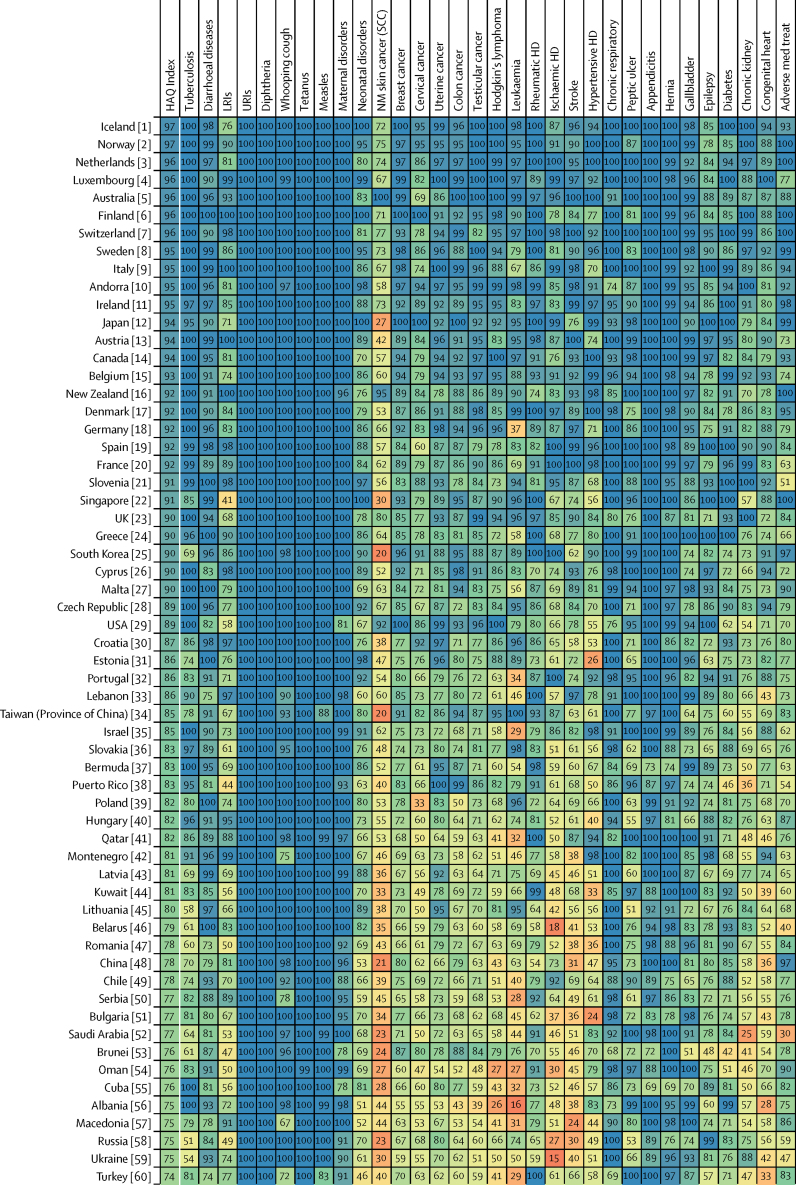

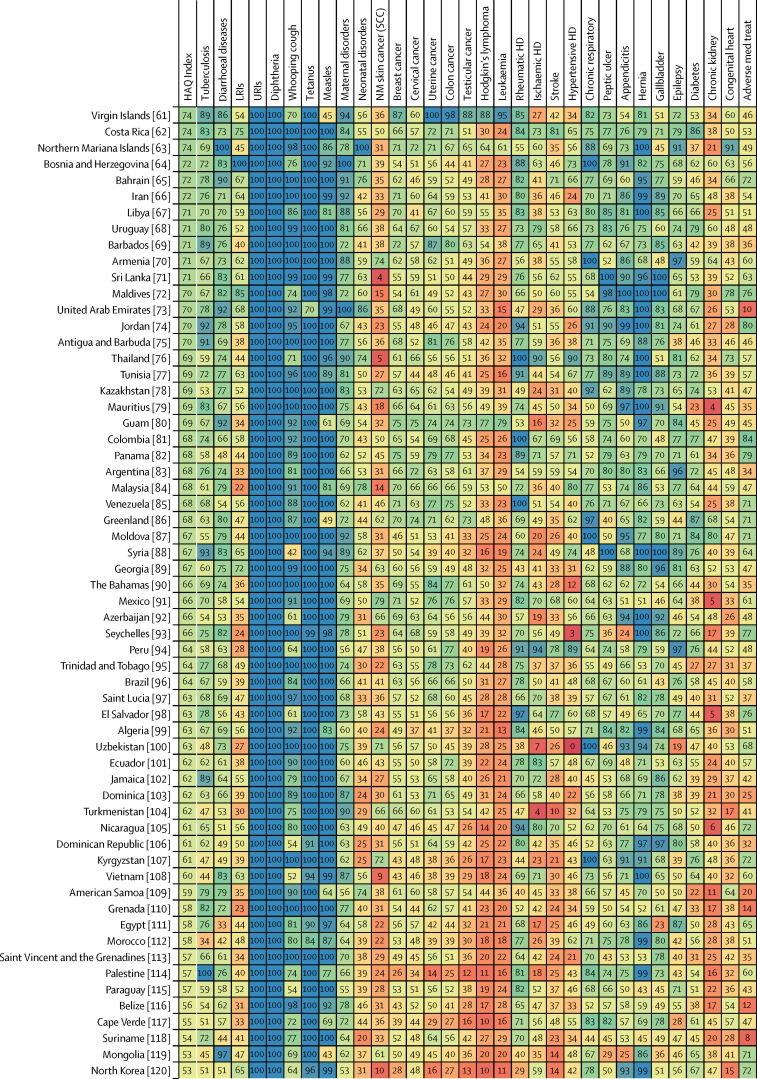

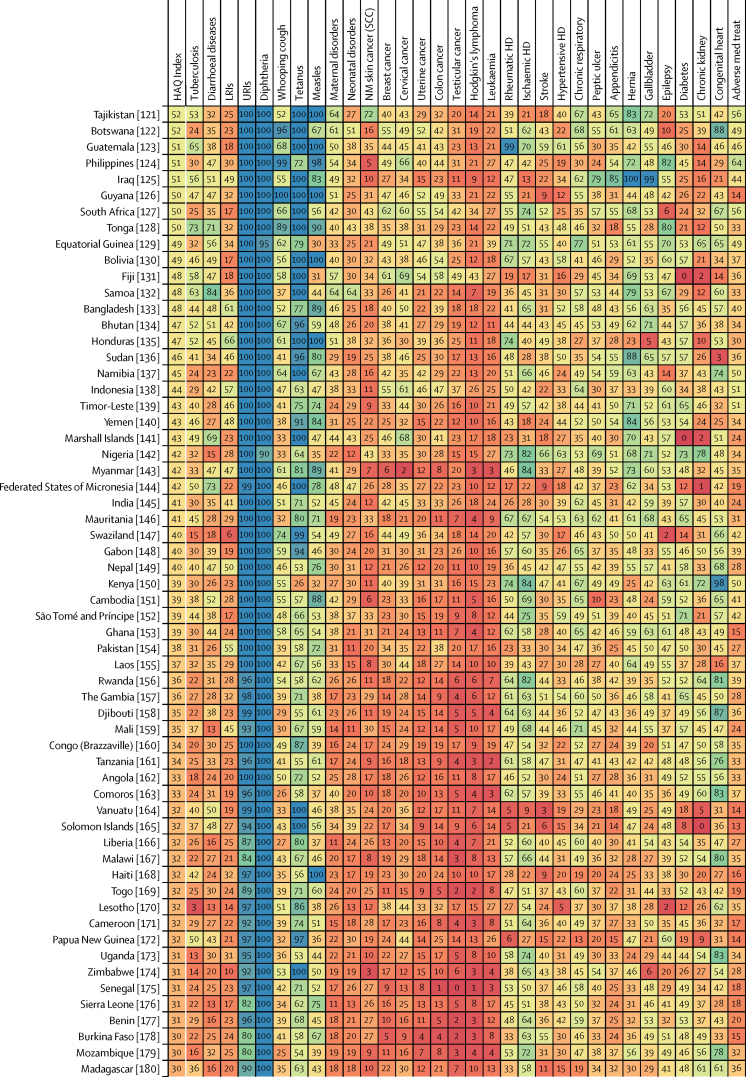

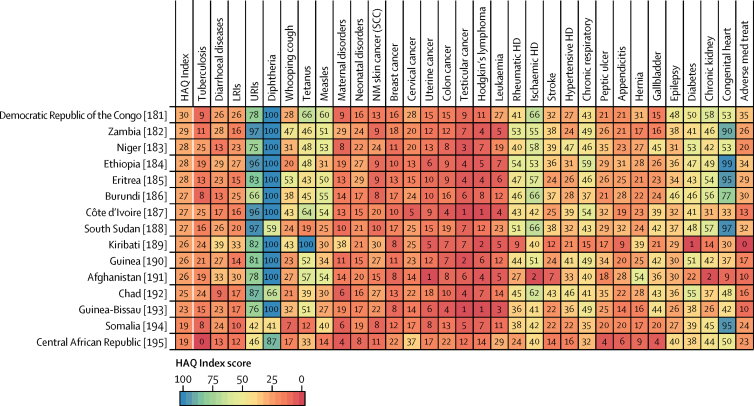
Performance on the HAQ Index and 32 individual causes, by country or territory, in 2016 Countries are ranked by their HAQ Index score from highest to lowest in 2016. The HAQ Index and individual causes are reported on a scale of 0–100, with 0 representing the worst levels observed from 1990 to 2016, and 100 reflecting the best during that time. HAQ Index=Healthcare Access and Quality Index. LRIs=lower respiratory infections. URIs=upper respiratory infections. NM=non-melanoma. SCC=squamous-cell carcinoma. Colon cancer=colon and rectum cancer. HD=heart disease. Chronic respiratory=chronic respiratory diseases. Peptic ulcer=peptic ulcer disease. Hernia=inguinal, femoral, and abdominal hernia. Gallbladder=gallbladder and biliary diseases. Chronic kidney=chronic kidney disease. Congenital heart=congenital heart anomalies. Adverse med treat=adverse effects of medical treatment.

Patterns of performance on the overall HAQ Index and health areas varied considerably across countries in 2016 (figure 3). Locations that scored approximately 90 or higher on the HAQ Index had generally high scores across broader causes, including vaccine-preventable diseases, infectious diseases and maternal and child health, and causes that require complex case management (eg, epilepsy, diabetes, and chronic kidney disease). Nonetheless, many of these countries had lower scores for cancers and some non-communicable diseases. Greater heterogeneity occurred across causes for countries that scored below 90 on the HAQ Index, though many locations achieved greater consistency, and high scores, for vaccine-preventable diseases and gastrointestinal causes for which surgery could avert death. For these countries, a mixture of relatively low values on cancers and some non-communicable diseases, and then comparably better performance on other health areas, was commonplace. Among countries with lower HAQ Index scores in 2016 (ie, lower than approximately 50), most fared poorly across health areas and recorded particularly low scores on cancers, some infectious causes like tuberculosis, and maternal and child health. Nonetheless, many still exceeded a score of 90 for some causes (eg, diphtheria, upper respiratory infections).

### Progress on personal health-care access and quality

**Figure 4:**
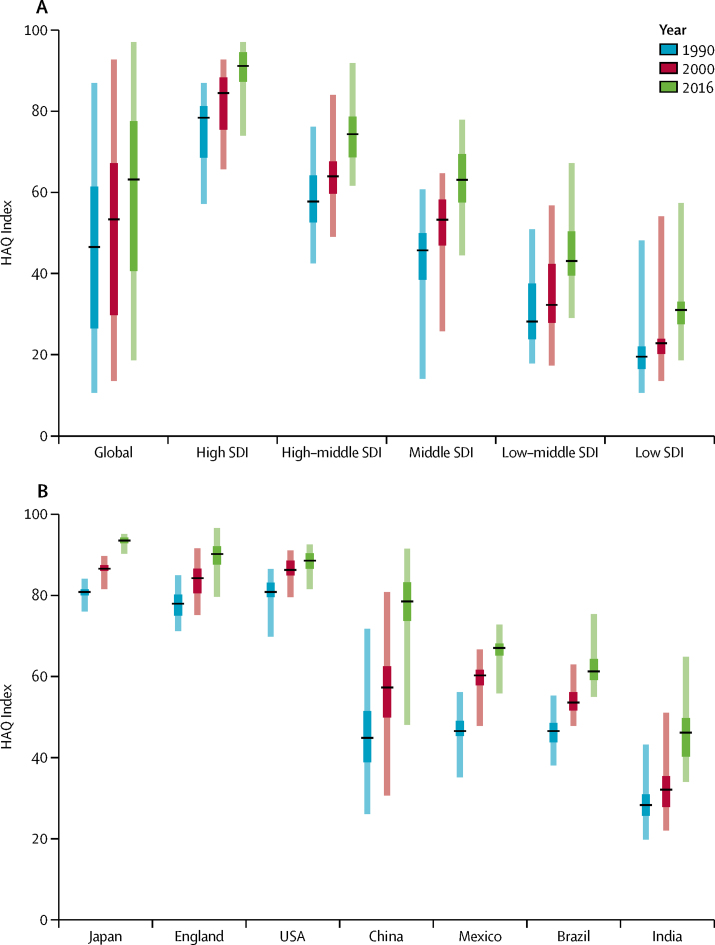
Median, IQR, and range of the HAQ Index in 1990, 2000, and 2016, globally and by SDI quintile (A), and for seven countries with subnational estimates (B) Black lines represent the median, dark-coloured boxes represent the IQR, and the light-coloured boxes represent the full range of values within a given group. Subnational locations represented in panel B are as follows: 47 prefectures in Japan; 150 local government areas in England; 50 states and the District of Columbia in the USA; 33 provinces and special administrative regions in China; 32 states in Mexico; 26 states and the Federal District in Brazil; and 31 states and union territories in India. HAQ Index=Healthcare Access and Quality Index. SDI=Socio-demographic Index.

Although global gaps between the highest and lowest HAQ Index values slightly widened over time (from 76·4 in 1990 to 78·5 in 2016), changes by SDI quintile showed more diverse trends (figure 4A). Low-middle-SDI countries saw some differences increase since 1990, with HAQ Index scores ranging from 29·0 to 67·2 by 2016. Conversely, disparities considerably narrowed among middle-SDI countries from 1990 (a 46·8-point difference) to 2016 (a 30·6-point difference). Among countries with subnational HAQ Index estimates (figure 4B), there was variation in when and how much local inequalities changed. In the USA, state-level differences decreased since 1990, but then comparably little progress occurred from 2000 to 2016. On the other hand, in Japan, absolute differences between prefectures narrowed to a 4·8-point difference between 2000 and 2016. In England, disparities slightly increased since 1990, from a 13·7-point difference in 1990, to a 16·9-point difference in 2016. China's overall gains quickened since 2000, though absolute differences between Chinese provinces remained high in 2016 (a 43·5-point gap). Mexico's progress on the HAQ Index was much faster from 1990 to 2000, than from 2000 to 2016, although absolute inequalities somewhat narrowed by 2016 (ie, a 20·9-point difference to a 17·0-point difference). Brazil's state-level disparities slightly widened after 2000, rising from an absolute difference of 17·2 in 1990, to 20·4 in 2016. However, compared with Mexico, Brazil's overall progress was more consistent across time periods. Although India's improvements on the HAQ Index hastened from 2000 to 2016, the gap between the country's highest and lowest scores widened (23·4-point difference in 1990, and 30·8-point difference in 2016).

**Figure 5:**
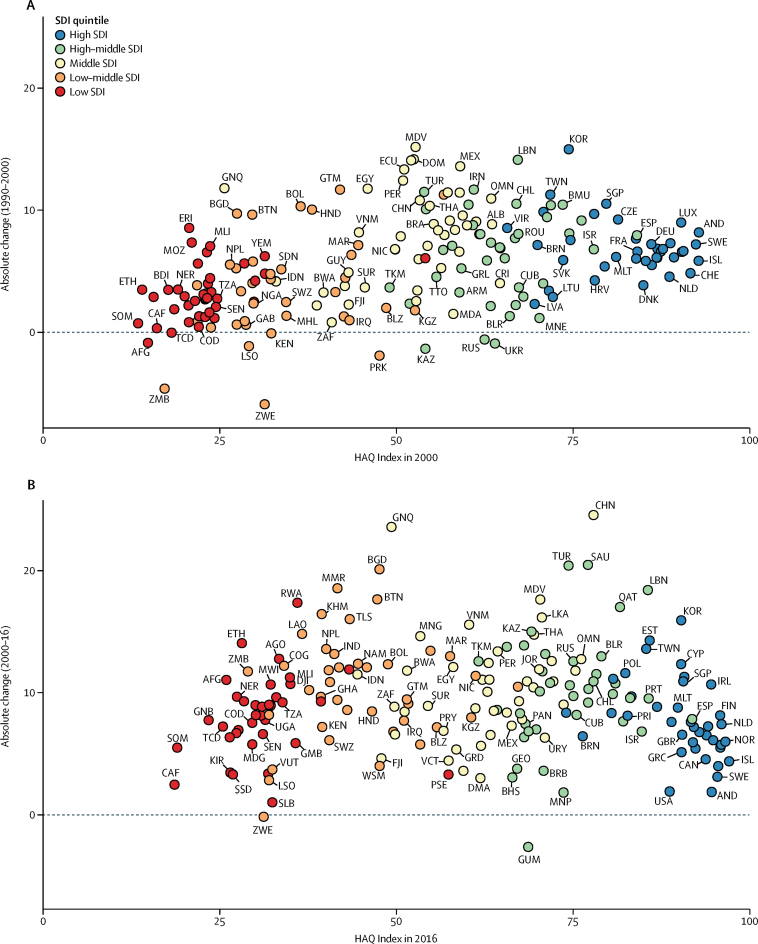
Absolute change on the HAQ Index, by SDI quintile, 1990–2000 (A) and 2000–16 (B) Countries and territories are colour-coded by their SDI quintile, and are abbreviated according to their ISO3 codes, which are listed in the appendix (pp 90–95). HAQ Index=Healthcare Access and Quality Index. SDI=Socio-demographic Index.

From 1990 to 2016, 186 of 195 countries and territories significantly increased their HAQ Index score, with several middle-SDI countries, including China, the Maldives, Equatorial Guinea, Peru, and Thailand achieving among the most pronounced gains (table 2; appendix p 130). South Korea, Taiwan (Province of China), and Cyprus recorded the largest improvements among high-SDI countries, and Lebanon, Turkey, and Saudi Arabia had the most progress for high-middle-SDI countries. For many low-middle-SDI and low-SDI countries, advances in the HAQ Index either primarily took place or accelerated from 2000 to 2016 (figure 5; appendix pp 133–35). Bangladesh, Myanmar, Bhutan, Cambodia, and Laos (low-middle SDI), and Rwanda and Ethiopia (low SDI), exemplified this trend. Some countries in eastern Europe and central Asia (eg, Russia, Belarus, Kazakhstan) also experienced substantive progress from 2000 to 2016, after stalled gains or faltering performance from 1990 to 2000. A subset of countries, including Vietnam and Nepal, recorded more comparable rates of change for each time period, whereas others, including several countries in Latin America and the Caribbean (eg, Guatemala, Mexico, Dominican Republic; table 2, appendix pp 133–35), had much slower progress after making considerable gains from 1990 to 2000. Nine countries, all low-to-middle SDI, did not record significant increases from 1990 to 2016. Table 2 and the appendix (pp 158–64) provide estimates of HAQ Index values, as well as absolute change and annualised rates of change for 1990–2000, 2000–16, and 1990–2016.

**Table 2:** Global, regional, and national or territory estimates of the HAQ Index for 1990, 2000, and 2016, and absolute change and annualised rates of change for 1990–2016, 1990–2000, and 2000–16

			**HAQ Index (95% UI)**			**Absolute change (95% UI)**			**Annualised rate of change (95% UI)**		
			1990	2000	2016	1990–2016	1990–2000	2000–16	1990–2016	1990–2000	2000–16
Global			37·6 (36·8 to 38·8)	42·4 (41·6 to 43·2)	54·4 (53·5 to 55·4)	16·8 (15·2 to 18·0)*	4·7 (4·0 to 5·4)*	12·0 (10·9 to 13·1)*	1·42 (1·28 to 1·53)*	1·18 (0·99 to 1·36)*	1·56 (1·42 to 1·70)*
**Southeast Asia, east Asia, and Oceania**†			**37·1 (35·9 to 38·6)**	**44·9 (43·9 to 46·2)**	**62·9 (61·8 to 64·2)**	25·9 (24·1 to 27·3)*	7·8 (6·9 to 8·8)*	18·0 (16·6 to 19·4)*	2·04 (1·88 to 2·16)*	1·92 (1·67 to 2·17)*	2·11 (1·93 to 2·27)*
	East Asia		42·8 (41·4 to 44·6)	53·3 (52·1 to 54·9)	77·0 (75·5 to 78·1)	34·2 (31·7 to 35·9)*	10·5 (8·8 to 12·2)*	23·7 (21·7 to 25·3)*	2·26 (2·08 to 2·39)*	2·20 (1·80 to 2·56)*	2·30 (2·11 to 2·46)*
		China	42·6 (41·2 to 44·5)	53·3 (52·0 to 55·1)	77·9 (76·5 to 78·9)	35·3 (32·8 to 37·0)*	10·8 (8·8 to 12·6)*	24·6 (22·4 to 26·2)*	2·33 (2·13 to 2·46)*	2·25 (1·83 to 2·63)*	2·37 (2·15 to 2·54)*
		North Korea	49·6 (46·2 to 52·9)	47·6 (44·1 to 51·2)	53·4 (49·6 to 56·9)	3·8 (−1·3 to 8·2)	−1·9 (−6·2 to 2·0)	5·7 (1·2 to 10·2)*	0·28 (−0·10 to 0·62)	−0·40 (−1·26 to 0·41)	0·71 (0·15 to 1·26)*
		Taiwan (Province of China)	60·6 (58·6 to 62·7)	71·8 (69·9 to 73·7)	85·4 (82·5 to 88·2)	24·8 (21·4 to 28·1)*	11·2 (8·6 to 13·6)*	13·6 (10·2 to 16·7)*	1·32 (1·14 to 1·49)*	1·70 (1·30 to 2·07)*	1·08 (0·82 to 1·32)*
	Oceania		27·2 (22·9 to 31·0)	32·4 (28·4 to 36·3)	36·0 (31·8 to 40·4)	8·8 (4·0 to 13·5)*	5·2 (1·9 to 8·5)*	3·6 (−0·5 to 7·8)	1·08 (0·49 to 1·66)*	1·76 (0·62 to 2·97)*	0·66 (−0·10 to 1·44)
		American Samoa	47·6 (44·6 to 50·6)	55·9 (52·9 to 59·1)	59·5 (55·0 to 64·1)	11·9 (6·5 to 17·4)*	8·3 (4·1 to 12·5)*	3·6 (−1·8 to 8·9)	0·86 (0·46 to 1·23)*	1·61 (0·79 to 2·42)*	0·38 (−0·20 to 0·96)
		Federated States of Micronesia	27·9 (23·4 to 32·5)	32·2 (27·2 to 37·1)	41·6 (34·8 to 49·1)	13·7 (5·8 to 21·4)*	4·3 (0·0 to 8·0)	9·4 (2·3 to 17·2)*	1·54 (0·68 to 2·40)*	1·44 (0·02 to 2·72)*	1·59 (0·44 to 2·77)*
		Fiji	41·0 (34·8 to 47·2)	43·3 (39·7 to 47·0)	47·9 (41·9 to 54·3)	6·8 (−1·9 to 15·4)	2·2 (−4·0 to 8·6)	4·6 (−2·4 to 11·8)	0·59 (−0·17 to 1·35)	0·55 (−0·92 to 2·16)	0·62 (−0·34 to 1·59)
		Guam	61·9 (59·0 to 64·9)	71·3 (68·7 to 74·0)	68·7 (64·8 to 72·9)	6·7 (2·0 to 11·6)*	9·4 (5·6 to 13·4)*	−2·7 (−7·5 to 2·5)	0·40 (0·12 to 0·67)*	1·41 (0·83 to 2·03)*	−0·24 (−0·67 to 0·21)
		Kiribati	20·3 (17·0 to 23·8)	23·0 (19·9 to 26·3)	26·5 (21·4 to 31·1)	6·2 (1·0 to 11·1)*	2·7 (−1·0 to 6·0)	3·4 (−1·1 to 7·9)	1·02 (0·19 to 1·81)*	1·27 (−0·49 to 2·79)	0·86 (−0·27 to 1·95)
		Marshall Islands	33·1 (30·4 to 36·1)	34·5 (31·1 to 38·0)	43·0 (38·0 to 48·2)	9·9 (4·3 to 15·1)*	1·3 (−2·5 to 5·3)	8·6 (3·5 to 13·7)*	1·00 (0·46 to 1·50)*	0·38 (−0·76 to 1·54)	1·39 (0·56 to 2·17)*
		Northern Mariana Islands	61·5 (56·0 to 67·0)	71·9 (67·7 to 75·9)	73·7 (69·2 to 78·3)	12·2 (5·4 to 19·4)*	10·4 (5·6 to 15·1)*	1·8 (−3·8 to 7·4)	0·70 (0·30 to 1·12)*	1·56 (0·83 to 2·37)*	0·15 (−0·33 to 0·64)
		Papua New Guinea	22·9 (17·8 to 27·7)	28·5 (23·2 to 33·6)	31·8 (26·2 to 37·4)	8·9 (2·7 to 15·1)*	5·6 (1·4 to 9·8)*	3·3 (−2·1 to 8·6)	1·27 (0·37 to 2·15)*	2·19 (0·52 to 3·95)*	0·70 (−0·43 to 1·85)
		Samoa	37·4 (32·8 to 41·7)	43·6 (38·8 to 48·2)	47·6 (42·8 to 52·6)	10·3 (4·5 to 16·1)*	6·3 (2·6 to 9·7)*	4·0 (−1·2 to 9·0)	0·93 (0·41 to 1·48)*	1·56 (0·67 to 2·43)*	0·55 (−0·16 to 1·21)
		Solomon Islands	26·7 (21·2 to 32·3)	31·4 (25·9 to 36·8)	32·4 (27·1 to 37·7)	5·8 (−0·9 to 12·3)	4·8 (0·4 to 8·8)*	1·0 (−4·6 to 6·2)	0·76 (−0·11 to 1·65)	1·66 (0·14 to 3·15)*	0·20 (−0·87 to 1·24)
		Tonga	38·4 (33·7 to 42·9)	42·8 (38·4 to 47·2)	49·6 (44·4 to 54·4)	11·2 (5·0 to 17·4)*	4·4 (0·4 to 8·3)*	6·8 (1·7 to 11·8)*	0·99 (0·44 to 1·55)*	1·10 (0·08 to 2·11)*	0·92 (0·22 to 1·60)*
		Vanuatu	28·2 (23·2 to 33·1)	28·7 (24·0 to 33·2)	32·4 (26·9 to 37·5)	4·3 (−2·0 to 10·3)	0·6 (−3·4 to 4·8)	3·7 (−1·9 to 8·9)	0·55 (−0·26 to 1·36)	0·21 (−1·17 to 1·73)	0·75 (−0·39 to 1·82)
	Southeast Asia		29·3 (27·8 to 30·8)	34·5 (33·0 to 36·0)	47·5 (45·9 to 49·2)	18·1 (16·4 to 20·0)*	5·1 (4·0 to 6·2)*	13·0 (11·4 to 14·6)*	1·85 (1·67 to 2·05)*	1·61 (1·25 to 1·97)*	2·00 (1·76 to 2·27)*
		Cambodia	20·3 (17·7 to 23·6)	23·0 (20·9 to 25·3)	39·4 (36·4 to 42·5)	19·1 (14·8 to 23·0)*	2·7 (−0·7 to 5·7)	16·5 (13·0 to 19·9)*	2·56 (1·90 to 3·12)*	1·25 (−0·33 to 2·76)	3·38 (2·65 to 4·10)*
		Indonesia	28·9 (26·4 to 31·7)	33·0 (31·1 to 35·3)	44·5 (42·6 to 46·8)	15·6 (12·8 to 18·4)*	4·1 (1·8 to 6·0)*	11·5 (9·2 to 13·8)*	1·67 (1·33 to 2·01)*	1·34 (0·57 to 2·00)*	1·87 (1·49 to 2·27)*
		Laos	18·0 (15·4 to 21·4)	21·8 (18·8 to 24·7)	36·6 (32·6 to 41·1)	18·6 (13·6 to 24·0)*	3·8 (0·4 to 7·1)*	14·8 (10·2 to 19·6)*	2·74 (1·97 to 3·51)*	1·94 (0·21 to 3·58)*	3·24 (2·23 to 4·25)*
		Malaysia	44·2 (42·5 to 46·1)	54·2 (52·6 to 55·9)	68·1 (65·9 to 70·2)	23·9 (21·3 to 26·6)*	10·0 (7·8 to 12·3)*	13·9 (11·5 to 16·2)*	1·66 (1·47 to 1·85)*	2·05 (1·59 to 2·51)*	1·43 (1·19 to 1·66)*
		Maldives	37·6 (33·6 to 41·0)	52·7 (49·9 to 55·4)	70·4 (65·7 to 74·8)	32·8 (26·9 to 39·1)*	15·1 (11·9 to 18·6)*	17·6 (11·9 to 22·8)*	2·41 (1·98 to 2·92)*	3·39 (2·62 to 4·32)*	1·80 (1·24 to 2·29)*
		Mauritius	53·9 (52·6 to 55·3)	61·9 (60·4 to 63·2)	68·7 (65·5 to 71·9)	14·8 (11·6 to 18·0)*	8·0 (6·4 to 9·4)*	6·8 (3·6 to 10·0)*	0·93 (0·75 to 1·12)*	1·38 (1·10 to 1·64)*	0·65 (0·35 to 0·94)*
		Myanmar	19·9 (17·2 to 22·6)	23·1 (20·2 to 26·0)	41·6 (38·0 to 45·5)	21·7 (17·4 to 26·4)*	3·1 (−0·2 to 6·2)	18·6 (14·5 to 22·5)*	2·84 (2·29 to 3·44)*	1·46 (−0·08 to 2·86)	3·70 (2·82 to 4·54)*
		Philippines	39·0 (37·3 to 40·6)	42·7 (40·7 to 44·5)	51·2 (47·9 to 54·4)	12·2 (8·7 to 15·8)*	3·8 (1·9 to 5·7)*	8·4 (4·8 to 11·9)*	1·05 (0·76 to 1·33)*	0·92 (0·46 to 1·41)*	1·12 (0·65 to 1·56)*
		Seychelles	45·9 (43·8 to 48·1)	57·3 (55·2 to 59·4)	65·6 (62·2 to 68·9)	19·8 (16·1 to 23·5)*	11·4 (8·6 to 14·0)*	8·4 (4·6 to 12·0)*	1·38 (1·12 to 1·63)*	2·22 (1·67 to 2·75)*	0·85 (0·48 to 1·22)*
		Sri Lanka	47·4 (45·1 to 49·8)	54·4 (52·1 to 56·9)	70·6 (66·3 to 75·3)	23·2 (18·5 to 28·1)*	7·0 (3·8 to 10·2)*	16·2 (11·5 to 21·0)*	1·53 (1·24 to 1·84)*	1·38 (0·75 to 2·02)*	1·62 (1·16 to 2·08)*
		Thailand	44·4 (42·4 to 46·6)	54·7 (52·2 to 57·4)	69·5 (66·5 to 72·6)	25·1 (21·4 to 28·7)*	10·3 (7·2 to 13·3)*	14·8 (10·9 to 18·6)*	1·72 (1·46 to 1·95)*	2·09 (1·49 to 2·66)*	1·49 (1·09 to 1·88)*
		Timor–Leste	22·2 (17·2 to 27·8)	27·3 (23·0 to 34·5)	43·4 (37·2 to 51·9)	21·2 (12·9 to 29·8)*	5·2 (−0·9 to 12·3)	16·0 (9·3 to 22·8)*	2·60 (1·51 to 3·68)*	2·12 (−0·33 to 4·94)	2·89 (1·61 to 4·06)*
		Vietnam	36·6 (33·1 to 40·4)	44·7 (41·6 to 48·2)	60·3 (56·3 to 64·1)	23·7 (18·1 to 29·0)*	8·1 (4·2 to 12·1)*	15·6 (10·8 to 20·3)*	1·92 (1·46 to 2·40)*	2·01 (1·01 to 3·05)*	1·87 (1·28 to 2·47)*
Central Europe, eastern Europe, and central Asia†			57·1 (55·8 to 58·6)	59·5 (58·1 to 60·8)	71·4 (68·1 to 74·3)	14·3 (10·9 to 17·4)*	2·5 (0·6 to 4·2)*	11·8 (8·4 to 14·9)*	0·86 (0·66 to 1·03)*	0·43 (0·10 to 0·73)*	1·13 (0·82 to 1·41)*
	Central Asia		48·4 (47·0 to 49·9)	49·6 (48·2 to 51·0)	60·2 (58·2 to 62·4)	11·8 (9·5 to 14·1)*	1·2 (−0·5 to 2·8)	10·6 (8·3 to 12·9)*	0·84 (0·68 to 1·00)*	0·25 (−0·10 to 0·58)	1·21 (0·96 to 1·47)*
		Armenia	55·7 (53·6 to 58·0)	58·9 (57·2 to 61·0)	70·7 (67·8 to 73·5)	15·0 (11·9 to 18·0)*	3·2 (1·1 to 5·3)*	11·7 (8·9 to 14·8)*	0·92 (0·74 to 1·10)*	0·56 (0·18 to 0·94)*	1·14 (0·87 to 1·42)*
		Azerbaijan	49·6 (47·0 to 52·1)	51·9 (49·4 to 54·4)	65·6 (61·2 to 69·6)	16·1 (11·1 to 20·6)*	2·3 (−1·1 to 5·6)	13·8 (9·2 to 18·4)*	1·08 (0·76 to 1·37)*	0·46 (−0·21 to 1·10)	1·47 (1·00 to 1·93)*
		Georgia	61·2 (59·0 to 63·5)	63·4 (60·8 to 65·4)	67·1 (62·7 to 71·0)	5·9 (1·1 to 10·7)*	2·1 (−0·7 to 4·7)	3·7 (−0·8 to 7·9)	0·35 (0·07 to 0·63)*	0·34 (−0·11 to 0·76)	0·36 (−0·08 to 0·76)
		Kazakhstan	55·5 (53·1 to 57·6)	54·1 (51·4 to 56·5)	69·1 (64·7 to 73·2)	13·6 (9·3 to 18·0)*	−1·4 (−4·3 to 1·5)	15·0 (10·2 to 19·6)*	0·84 (0·58 to 1·10)*	−0·25 (−0·80 to 0·27)	1·53 (1·05 to 1·97)*
		Kyrgyzstan	50·9 (49·5 to 53·1)	52·6 (51·3 to 54·2)	60·6 (58·3 to 62·8)	9·7 (6·7 to 12·4)*	1·8 (0·1 to 3·3)*	8·0 (5·2 to 10·3)*	0·67 (0·46 to 0·85)*	0·34 (0·02 to 0·63)*	0·88 (0·57 to 1·13)*
		Mongolia	36·6 (34·0 to 39·3)	38·7 (36·1 to 41·5)	53·4 (49·1 to 57·6)	16·8 (11·3 to 21·9)*	2·2 (−1·3 to 5·6)	14·6 (9·5 to 19·7)*	1·45 (0·98 to 1·86)*	0·58 (−0·35 to 1·46)	2·00 (1·32 to 2·65)*
		Tajikistan	41·3 (38·7 to 44·2)	42·6 (39·9 to 45·5)	51·7 (47·7 to 55·5)	10·4 (5·7 to 15·3)*	1·3 (−2·7 to 5·1)	9·1 (4·4 to 13·8)*	0·86 (0·48 to 1·25)*	0·30 (−0·64 to 1·24)	1·21 (0·60 to 1·82)*
		Turkmenistan	45·4 (43·8 to 46·9)	49·1 (47·1 to 51·0)	61·6 (58·7 to 64·8)	16·2 (13·0 to 20·2)*	3·6 (1·3 to 6·1)*	12·6 (9·8 to 15·3)*	1·17 (0·96 to 1·44)*	0·77 (0·27 to 1·29)*	1·43 (1·12 to 1·71)*
		Uzbekistan	50·3 (48·4 to 52·2)	52·8 (51·0 to 54·6)	62·9 (59·3 to 66·0)	12·6 (8·6 to 16·1)*	2·5 (0·2 to 4·8)*	10·1 (6·2 to 13·2)*	0·86 (0·60 to 1·09)*	0·49 (0·04 to 0·92)*	1·09 (0·69 to 1·42)*
	Central Europe		58·8 (57·7 to 60·2)	68·9 (67·6 to 69·9)	80·6 (79·2 to 81·7)	21·8 (19·6 to 23·2)*	10·1 (8·3 to 11·3)*	11·7 (10·5 to 12·9)*	1·21 (1·09 to 1·30)*	1·58 (1·30 to 1·79)*	0·98 (0·88 to 1·09)*
		Albania	54·8 (52·7 to 56·9)	63·6 (61·5 to 65·7)	75·4 (72·5 to 78·2)	20·6 (17·2 to 24·0)*	8·8 (6·1 to 11·7)*	11·8 (8·4 to 15·0)*	1·23 (1·03 to 1·42)*	1·49 (1·03 to 1·96)*	1·06 (0·77 to 1·35)*
		Bosnia and Herzegovina	52·3 (49·4 to 55·2)	61·3 (58·1 to 64·4)	72·2 (67·2 to 76·4)	19·9 (14·8 to 24·6)*	9·0 (5·8 to 12·3)*	10·9 (5·9 to 16·1)*	1·24 (0·94 to 1·52)*	1·59 (1·01 to 2·18)*	1·02 (0·56 to 1·51)*
		Bulgaria	65·1 (64·0 to 66·4)	68·0 (66·5 to 69·0)	77·2 (73·3 to 80·7)	12·1 (8·4 to 15·8)*	2·9 (1·2 to 4·2)*	9·2 (5·4 to 12·8)*	0·65 (0·46 to 0·84)*	0·43 (0·18 to 0·63)*	0·79 (0·48 to 1·08)*
		Croatia	73·9 (71·9 to 76·2)	78·1 (76·5 to 79·7)	86·9 (84·5 to 89·4)	13·0 (9·7 to 16·4)*	4·2 (1·6 to 6·7)*	8·8 (5·8 to 11·8)*	0·63 (0·46 to 0·79)*	0·55 (0·21 to 0·90)*	0·67 (0·45 to 0·89)*
		Czech Republic	72·2 (70·9 to 73·4)	81·4 (79·8 to 82·4)	89·0 (87·5 to 90·4)	16·8 (14·9 to 18·7)*	9·2 (7·4 to 10·4)*	7·6 (6·0 to 9·5)*	0·80 (0·72 to 0·89)*	1·20 (0·96 to 1·35)*	0·56 (0·44 to 0·70)*
		Hungary	66·4 (64·8 to 68·6)	74·5 (73·0 to 76·0)	82·1 (79·5 to 84·9)	15·7 (12·6 to 18·7)*	8·0 (6·0 to 9·9)*	7·6 (4·7 to 10·7)*	0·81 (0·66 to 0·96)*	1·14 (0·83 to 1·41)*	0·61 (0·38 to 0·84)*
		Macedonia	59·3 (57·2 to 61·6)	65·3 (63·6 to 67·4)	75·1 (72·6 to 77·5)	15·7 (12·3 to 18·9)*	6·0 (3·4 to 8·4)*	9·7 (6·7 to 12·6)*	0·90 (0·71 to 1·09)*	0·96 (0·54 to 1·36)*	0·87 (0·61 to 1·11)*
		Montenegro	69·1 (66·5 to 71·7)	70·3 (68·4 to 72·4)	81·0 (78·6 to 83·5)	11·9 (8·3 to 15·5)*	1·1 (−1·8 to 3·9)	10·8 (7·8 to 13·9)*	0·61 (0·42 to 0·80)*	0·16 (−0·26 to 0·57)	0·89 (0·64 to 1·14)*
		Poland	61·0 (59·8 to 62·4)	70·8 (69·1 to 72·0)	82·4 (79·7 to 84·6)	21·4 (18·2 to 23·8)*	9·8 (7·6 to 11·4)*	11·6 (9·3 to 14·0)*	1·16 (0·99 to 1·28)*	1·49 (1·16 to 1·73)*	0·95 (0·77 to 1·13)*
		Romania	59·1 (57·6 to 61·0)	66·8 (65·2 to 68·4)	78·3 (75·9 to 80·7)	19·2 (16·3 to 21·9)*	7·7 (5·3 to 9·5)*	11·5 (8·9 to 14·2)*	1·08 (0·91 to 1·22)*	1·22 (0·83 to 1·51)*	0·99 (0·78 to 1·21)*
		Serbia	64·7 (61·9 to 67·5)	66·9 (64·9 to 69·2)	77·2 (74·9 to 79·3)	12·5 (9·3 to 15·6)*	2·2 (−0·7 to 5·2)	10·3 (7·4 to 13·0)*	0·68 (0·51 to 0·86)*	0·33 (−0·11 to 0·80)	0·90 (0·64 to 1·13)*
		Slovakia	67·8 (65·8 to 69·4)	73·6 (71·6 to 75·4)	83·3 (80·4 to 86·3)	15·5 (12·3 to 18·9)*	5·9 (3·6 to 8·1)*	9·7 (6·6 to 12·8)*	0·79 (0·64 to 0·95)*	0·83 (0·51 to 1·15)*	0·77 (0·53 to 1·02)*
		Slovenia	74·1 (72·2 to 76·1)	79·5 (77·8 to 81·3)	90·8 (88·2 to 93·4)	16·6 (13·5 to 19·8)*	5·3 (3·0 to 7·9)*	11·3 (8·0 to 14·6)*	0·78 (0·63 to 0·92)*	0·70 (0·39 to 1·03)*	0·83 (0·59 to 1·06)*
	Eastern Europe		63·5 (61·7 to 65·3)	63·1 (61·1 to 64·8)	75·0 (69·6 to 80·2)	11·5 (5·7 to 16·5)*	−0·4 (−3·0 to 1·9)	11·9 (6·4 to 17·1)*	0·64 (0·33 to 0·90)*	−0·07 (−0·48 to 0·29)	1·08 (0·60 to 1·51)*
		Belarus	64·8 (63·4 to 66·3)	66·1 (63·7 to 67·6)	79·0 (75·3 to 82·8)	14·3 (10·5 to 18·1)*	1·3 (−1·6 to 3·1)	13·0 (9·1 to 16·9)*	0·76 (0·58 to 0·96)*	0·20 (−0·25 to 0·48)	1·12 (0·79 to 1·46)*
		Estonia	68·2 (66·8 to 69·8)	71·6 (70·2 to 72·8)	85·9 (83·6 to 88·3)	17·7 (15·1 to 20·6)*	3·4 (1·7 to 5·0)*	14·3 (11·8 to 17·0)*	0·89 (0·76 to 1·03)*	0·48 (0·25 to 0·72)*	1·14 (0·94 to 1·35)*
		Latvia	67·3 (65·9 to 68·8)	69·6 (68·1 to 71·0)	80·7 (78·0 to 83·3)	13·4 (10·5 to 16·4)*	2·3 (0·4 to 4·1)*	11·1 (8·3 to 14·2)*	0·70 (0·55 to 0·84)*	0·33 (0·06 to 0·61)*	0·93 (0·70 to 1·17)*
		Lithuania	69·3 (68·0 to 70·6)	72·1 (70·6 to 73·4)	80·5 (78·7 to 82·3)	11·2 (9·2 to 13·2)*	2·9 (1·2 to 4·4)*	8·3 (6·0 to 10·7)*	0·58 (0·47 to 0·68)*	0·40 (0·17 to 0·62)*	0·68 (0·49 to 0·87)*
		Moldova	56·6 (54·4 to 59·0)	58·1 (56·0 to 60·2)	67·4 (64·5 to 70·4)	10·8 (7·3 to 14·0)*	1·5 (−1·5 to 4·3)	9·3 (6·2 to 12·6)*	0·67 (0·46 to 0·86)*	0·26 (−0·25 to 0·76)	0·93 (0·62 to 1·24)*
		Russia	63·1 (60·6 to 65·4)	62·5 (60·1 to 64·7)	75·1 (67·7 to 81·7)	11·9 (4·5 to 19·0)*	−0·6 (−3·8 to 2·5)	12·6 (5·0 to 19·4)*	0·66 (0·26 to 1·01)*	−0·10 (−0·63 to 0·40)	1·14 (0·48 to 1·73)*
		Ukraine	64·9 (63·3 to 66·5)	64·0 (61·8 to 65·8)	74·6 (68·3 to 79·8)	9·6 (3·3 to 15·2)*	−1·0 (−3·6 to 1·2)	10·6 (4·2 to 16·5)*	0·53 (0·19 to 0·81)*	−0·15 (−0·56 to 0·18)	0·95 (0·39 to 1·45)*
**High income**†			**75·5 (74·4 to 76·6)**	**83·2 (82·3 to 83·8)**	**89·8 (89·2 to 90·4)**	14·4 (13·3 to 15·5)*	7·7 (6·7 to 8·8)*	6·6 (6·0 to 7·4)*	0·67 (0·62 to 0·73)*	0·98 (0·84 to 1·11)*	0·48 (0·43 to 0·54)*
	Australasia		83·2 (82·4 to 84·0)	89·7 (89·0 to 90·5)	95·5 (94·5 to 96·4)	12·3 (11·2 to 13·3)*	6·5 (5·8 to 7·3)*	5·8 (4·8 to 6·8)*	0·53 (0·48 to 0·57)*	0·76 (0·67 to 0·85)*	0·39 (0·32 to 0·46)*
		Australia	83·9 (83·0 to 84·7)	90·4 (89·6 to 91·2)	95·9 (94·8 to 96·8)	12·0 (10·9 to 13·1)*	6·5 (5·6 to 7·5)*	5·5 (4·4 to 6·6)*	0·51 (0·47 to 0·56)*	0·75 (0·65 to 0·86)*	0·37 (0·30 to 0·44)*
		New Zealand	80·2 (79·2 to 81·4)	87·0 (86·0 to 87·8)	92·4 (90·3 to 94·3)	12·2 (9·8 to 14·3)*	6·8 (5·4 to 7·9)*	5·4 (3·1 to 7·4)*	0·54 (0·44 to 0·64)*	0·81 (0·64 to 0·95)*	0·38 (0·22 to 0·51)*
	High-income Asia Pacific		73·7 (72·1 to 75·6)	81·8 (80·6 to 83·1)	93·2 (91·8 to 94·2)	19·5 (16·9 to 21·5)*	8·1 (5·9 to 10·0)*	11·4 (9·7 to 13·0)*	0·90 (0·78 to 1·00)*	1·04 (0·75 to 1·30)*	0·81 (0·69 to 0·93)*
		Brunei	62·9 (60·0 to 65·6)	70·0 (67·5 to 72·7)	76·4 (71·9 to 81·0)	13·5 (8·4 to 18·7)*	7·1 (3·9 to 10·6)*	6·4 (1·4 to 11·3)*	0·75 (0·48 to 1·02)*	1·07 (0·60 to 1·60)*	0·55 (0·12 to 0·94)*
		Japan	80·9 (80·3 to 81·7)	86·9 (86·3 to 87·5)	94·1 (93·5 to 94·6)	13·3 (12·2 to 13·9)*	6·1 (5·4 to 6·4)*	7·2 (6·6 to 7·8)*	0·58 (0·54 to 0·62)*	0·72 (0·65 to 0·77)*	0·50 (0·45 to 0·54)*
		Singapore	69·2 (66·5 to 72·0)	79·7 (77·2 to 82·0)	90·6 (87·2 to 93·3)	21·4 (17·5 to 25·0)*	10·5 (7·1 to 13·9)*	10·9 (7·1 to 14·8)*	1·04 (0·85 to 1·21)*	1·41 (0·95 to 1·88)*	0·80 (0·53 to 1·08)*
		South Korea	59·5 (56·2 to 62·9)	74·4 (71·4 to 77·0)	90·3 (85·6 to 93·9)	30·9 (24·6 to 35·7)*	14·9 (10·0 to 18·9)*	15·9 (10·9 to 20·4)*	1·61 (1·28 to 1·87)*	2·24 (1·47 to 2·86)*	1·21 (0·84 to 1·54)*
	High-income North America		81·0 (80·1 to 81·7)	87·1 (86·5 to 87·7)	89·1 (88·4 to 89·8)	8·1 (7·4 to 9·0)*	6·1 (5·5 to 6·8)*	2·0 (1·5 to 2·6)*	0·37 (0·34 to 0·41)*	0·73 (0·66 to 0·81)*	0·14 (0·11 to 0·18)*
		Canada	83·2 (82·2 to 84·1)	89·3 (88·4 to 90·2)	93·8 (92·8 to 94·8)	10·6 (9·3 to 11·9)*	6·1 (5·1 to 6·9)*	4·5 (3·4 to 5·7)*	0·46 (0·40 to 0·52)*	0·71 (0·59 to 0·80)*	0·31 (0·24 to 0·39)*
		Greenland	54·0 (50·6 to 57·5)	59·2 (56·4 to 62·8)	67·5 (62·7 to 72·7)	13·5 (8·0 to 19·0)*	5·2 (1·6 to 8·9)*	8·3 (3·3 to 13·5)*	0·86 (0·52 to 1·19)*	0·92 (0·29 to 1·55)*	0·82 (0·33 to 1·31)*
		USA	80·7 (79·8 to 81·5)	86·8 (86·1 to 87·4)	88·7 (88·0 to 89·4)	8·0 (7·2 to 8·8)*	6·1 (5·5 to 6·7)*	1·9 (1·4 to 2·5)*	0·36 (0·33 to 0·40)*	0·72 (0·65 to 0·81)*	0·13 (0·10 to 0·18)*
	Southern Latin America		54·2 (52·9 to 55·5)	62·6 (61·0 to 63·8)	70·0 (67·9 to 72·0)	15·8 (13·7 to 17·8)*	8·4 (6·8 to 9·7)*	7·4 (5·2 to 9·5)*	0·99 (0·86 to 1·10)*	1·45 (1·17 to 1·66)*	0·70 (0·50 to 0·89)*
		Argentina	53·8 (52·3 to 55·2)	61·7 (59·8 to 63·1)	68·1 (65·8 to 70·1)	14·3 (12·0 to 16·5)*	8·0 (6·1 to 9·5)*	6·3 (4·2 to 8·5)*	0·91 (0·77 to 1·04)*	1·38 (1·06 to 1·64)*	0·61 (0·41 to 0·82)*
		Chile	56·5 (54·9 to 58·4)	67·0 (65·4 to 68·5)	77·9 (72·3 to 83·7)	21·4 (15·5 to 27·5)*	10·5 (8·4 to 12·5)*	10·9 (5·3 to 16·4)*	1·23 (0·93 to 1·53)*	1·70 (1·35 to 2·03)*	0·94 (0·47 to 1·39)*
		Uruguay	57·9 (56·7 to 59·1)	64·7 (63·2 to 65·8)	71·0 (68·9 to 73·0)	13·1 (10·9 to 15·2)*	6·8 (5·1 to 8·2)*	6·3 (4·1 to 8·5)*	0·79 (0·66 to 0·90)*	1·12 (0·84 to 1·34)*	0·58 (0·39 to 0·78)*
	Western Europe		78·6 (77·9 to 79·6)	85·3 (84·6 to 86·0)	92·6 (91·7 to 93·3)	13·9 (12·8 to 14·8)*	6·7 (6·0 to 7·3)*	7·2 (6·6 to 7·9)*	0·63 (0·58 to 0·67)*	0·82 (0·73 to 0·90)*	0·51 (0·46 to 0·56)*
		Andorra	84·7 (79·5 to 89·3)	92·8 (88·9 to 96·0)	94·7 (91·2 to 97·0)	10·0 (4·4 to 15·4)*	8·1 (3·8 to 12·6)*	1·8 (−2·5 to 5·8)	0·43 (0·19 to 0·67)*	0·92 (0·43 to 1·45)*	0·12 (−0·17 to 0·39)
		Austria	80·9 (79·9 to 82·2)	87·4 (86·5 to 88·5)	93·9 (92·6 to 95·3)	13·1 (11·3 to 14·7)*	6·6 (5·6 to 7·6)*	6·5 (5·1 to 8·0)*	0·58 (0·50 to 0·65)*	0·78 (0·66 to 0·91)*	0·45 (0·36 to 0·55)*
		Belgium	80·7 (79·4 to 82·2)	86·1 (84·8 to 87·3)	92·9 (90·7 to 95·0)	12·2 (9·6 to 14·7)*	5·4 (3·7 to 7·1)*	6·8 (4·6 to 9·1)*	0·54 (0·43 to 0·65)*	0·65 (0·44 to 0·85)*	0·47 (0·32 to 0·63)*
		Cyprus	68·3 (66·3 to 70·5)	78·0 (76·6 to 79·7)	90·3 (88·8 to 91·8)	22·0 (19·6 to 24·3)*	9·6 (7·7 to 11·5)*	12·3 (10·5 to 14·3)*	1·07 (0·95 to 1·20)*	1·32 (1·04 to 1·59)*	0·92 (0·78 to 1·06)*
		Denmark	81·1 (79·3 to 82·7)	85·0 (83·5 to 86·8)	92·1 (89·8 to 94·3)	11·0 (8·2 to 13·7)*	3·8 (1·7 to 6·5)*	7·2 (4·5 to 10·0)*	0·49 (0·36 to 0·61)*	0·46 (0·20 to 0·78)*	0·51 (0·32 to 0·70)*
		Finland	81·0 (79·8 to 82·3)	87·7 (86·7 to 88·7)	95·9 (94·6 to 96·9)	14·9 (13·0 to 16·5)*	6·8 (5·3 to 8·0)*	8·1 (6·7 to 9·5)*	0·65 (0·56 to 0·72)*	0·80 (0·62 to 0·95)*	0·55 (0·46 to 0·65)*
		France	77·6 (76·4 to 79·1)	84·1 (83·0 to 85·3)	91·7 (90·3 to 93·1)	14·1 (12·1 to 16·0)*	6·6 (5·4 to 7·7)*	7·6 (6·0 to 9·1)*	0·64 (0·55 to 0·73)*	0·81 (0·66 to 0·95)*	0·54 (0·42 to 0·65)*
		Germany	78·9 (77·5 to 80·6)	86·1 (84·9 to 87·3)	92·0 (90·4 to 93·6)	13·1 (10·8 to 15·1)*	7·2 (5·4 to 8·9)*	5·9 (4·1 to 8·0)*	0·59 (0·49 to 0·68)*	0·87 (0·65 to 1·09)*	0·42 (0·29 to 0·56)*
		Greece	79·5 (78·4 to 80·5)	85·3 (84·4 to 86·3)	90·4 (88·8 to 91·9)	10·9 (9·1 to 12·6)*	5·8 (4·8 to 6·8)*	5·1 (3·5 to 6·7)*	0·49 (0·42 to 0·57)*	0·70 (0·58 to 0·84)*	0·36 (0·25 to 0·47)*
		Iceland	87·0 (85·6 to 88·5)	92·8 (91·5 to 93·9)	97·1 (95·8 to 98·1)	10·2 (8·6 to 11·7)*	5·8 (4·1 to 7·3)*	4·4 (2·8 to 6·0)*	0·42 (0·36 to 0·49)*	0·65 (0·46 to 0·81)*	0·29 (0·18 to 0·39)*
		Ireland	76·3 (74·9 to 77·5)	83·9 (82·4 to 85·4)	94·6 (91·8 to 96·8)	18·3 (15·3 to 20·9)*	7·6 (6·0 to 9·3)*	10·7 (7·8 to 13·4)*	0·83 (0·70 to 0·94)*	0·95 (0·75 to 1·17)*	0·75 (0·55 to 0·93)*
		Israel	71·2 (68·9 to 73·7)	77·9 (75·5 to 80·5)	84·8 (80·7 to 88·4)	13·5 (8·6 to 18·0)*	6·7 (3·4 to 10·0)*	6·8 (2·3 to 10·8)*	0·67 (0·43 to 0·88)*	0·90 (0·46 to 1·34)*	0·52 (0·18 to 0·83)*
		Italy	81·5 (80·6 to 82·4)	88·8 (87·8 to 89·7)	94·9 (93·4 to 96·0)	13·3 (11·8 to 14·7)*	7·2 (6·3 to 8·1)*	6·1 (4·7 to 7·4)*	0·58 (0·52 to 0·64)*	0·85 (0·74 to 0·96)*	0·41 (0·32 to 0·51)*
		Luxembourg	81·4 (79·7 to 83·0)	90·3 (88·8 to 91·6)	96·0 (94·4 to 97·3)	14·7 (12·4 to 16·7)*	8·9 (7·2 to 10·6)*	5·7 (3·9 to 7·4)*	0·64 (0·53 to 0·73)*	1·04 (0·83 to 1·24)*	0·38 (0·26 to 0·49)*
		Malta	75·0 (73·0 to 77·0)	81·1 (79·0 to 83·0)	89·9 (86·3 to 93·0)	14·9 (10·8 to 18·8)*	6·1 (3·5 to 8·7)*	8·8 (4·9 to 12·6)*	0·70 (0·52 to 0·87)*	0·78 (0·45 to 1·11)*	0·64 (0·36 to 0·91)*
		Netherlands	84·1 (82·8 to 85·4)	88·6 (87·1 to 89·8)	96·1 (94·5 to 97·3)	11·9 (10·0 to 13·6)*	4·5 (3·1 to 6·0)*	7·4 (5·6 to 9·1)*	0·51 (0·43 to 0·58)*	0·52 (0·36 to 0·69)*	0·50 (0·38 to 0·62)*
		Norway	84·0 (82·9 to 85·1)	90·6 (89·5 to 91·7)	96·6 (94·9 to 97·9)	12·6 (10·6 to 14·3)*	6·6 (5·4 to 7·9)*	6·0 (4·1 to 7·6)*	0·54 (0·46 to 0·61)*	0·76 (0·62 to 0·91)*	0·40 (0·27 to 0·51)*
		Portugal	67·1 (65·9 to 68·3)	76·2 (75·1 to 77·3)	85·7 (84·1 to 87·3)	18·6 (16·9 to 20·4)*	9·1 (8·0 to 10·4)*	9·5 (7·8 to 11·3)*	0·94 (0·86 to 1·03)*	1·27 (1·11 to 1·46)*	0·74 (0·61 to 0·87)*
		Spain	76·2 (75·2 to 77·2)	84·1 (83·1 to 84·9)	91·9 (90·5 to 93·2)	15·7 (14·2 to 17·3)*	7·9 (6·9 to 8·8)*	7·8 (6·5 to 9·2)*	0·72 (0·65 to 0·79)*	0·99 (0·87 to 1·11)*	0·56 (0·46 to 0·65)*
		Sweden	85·2 (84·2 to 86·2)	92·4 (91·5 to 93·2)	95·5 (93·4 to 97·2)	10·2 (7·9 to 12·1)*	7·1 (6·1 to 8·2)*	3·1 (1·0 to 5·0)*	0·44 (0·34 to 0·51)*	0·81 (0·69 to 0·93)*	0·21 (0·07 to 0·33)*
		Switzerland	86·8 (85·2 to 88·2)	91·6 (90·2 to 93·0)	95·6 (92·4 to 97·8)	8·8 (5·3 to 11·4)*	4·8 (3·0 to 6·6)*	4·0 (0·5 to 6·7)*	0·37 (0·22 to 0·48)*	0·54 (0·33 to 0·74)*	0·26 (0·04 to 0·45)*
		UK	78·0 (77·1 to 78·6)	83·9 (83·0 to 84·6)	90·5 (89·6 to 91·3)	12·5 (11·8 to 13·4)*	6·0 (5·5 to 6·5)*	6·5 (5·9 to 7·2)*	0·57 (0·54 to 0·61)*	0·74 (0·69 to 0·80)*	0·47 (0·43 to 0·51)*
**Latin America and Caribbean**†			**41·3 (40·3 to 42·5)**	**52·6 (51·3 to 53·7)**	**61·8 (60·4 to 63·0)**	20·5 (19·0 to 21·8)*	11·3 (9·8 to 12·3)*	9·2 (8·1 to 10·2)*	1·55 (1·43 to 1·65)*	2·42 (2·09 to 2·66)*	1·01 (0·89 to 1·12)*
	Andean Latin America		34·1 (32·4 to 36·0)	46·9 (45·3 to 48·6)	59·3 (56·3 to 62·4)	25·2 (21·4 to 28·8)*	12·8 (10·0 to 15·0)*	12·4 (9·5 to 15·3)*	2·13 (1·82 to 2·42)*	3·19 (2·47 to 3·76)*	1·47 (1·14 to 1·77)*
		Bolivia	26·2 (23·6 to 29·0)	36·5 (34·2 to 38·9)	48·8 (43·5 to 54·0)	22·6 (16·6 to 28·1)*	10·3 (7·1 to 13·2)*	12·3 (6·8 to 17·6)*	2·39 (1·82 to 2·93)*	3·31 (2·27 to 4·38)*	1·81 (1·08 to 2·51)*
		Ecuador	37·8 (36·1 to 39·9)	51·1 (48·9 to 52·8)	62·2 (59·5 to 64·6)	24·3 (20·8 to 27·4)*	13·3 (10·4 to 15·6)*	11·1 (8·8 to 13·4)*	1·91 (1·63 to 2·15)*	3·01 (2·35 to 3·55)*	1·22 (0·98 to 1·47)*
		Peru	38·6 (36·3 to 41·3)	51·0 (48·8 to 53·4)	64·3 (59·2 to 69·4)	25·8 (19·8 to 31·4)*	12·4 (8·7 to 15·7)*	13·4 (8·0 to 18·5)*	1·97 (1·52 to 2·34)*	2·79 (1·92 to 3·53)*	1·45 (0·90 to 1·97)*
	Caribbean		37·9 (36·1 to 40·0)	45·6 (43·6 to 47·7)	54·2 (51·1 to 57·3)	16·3 (12·7 to 19·7)*	7·7 (5·0 to 10·2)*	8·7 (5·3 to 12·1)*	1·38 (1·09 to 1·64)*	1·85 (1·20 to 2·43)*	1·09 (0·67 to 1·50)*
		Antigua and Barbuda	57·0 (54·5 to 59·5)	62·8 (60·2 to 65·4)	69·8 (66·5 to 73·3)	12·8 (8·7 to 16·7)*	5·8 (2·7 to 9·0)*	7·0 (3·2 to 11·2)*	0·78 (0·53 to 1·01)*	0·97 (0·46 to 1·51)*	0·66 (0·31 to 1·04)*
		Barbados	59·3 (57·1 to 61·6)	67·3 (64·3 to 69·7)	70·8 (67·3 to 73·8)	11·6 (7·5 to 15·4)*	8·0 (4·8 to 11·0)*	3·6 (−0·2 to 7·5)	0·69 (0·45 to 0·90)*	1·27 (0·76 to 1·73)*	0·32 (−0·02 to 0·67)
		Belize	46·6 (44·3 to 48·8)	48·6 (46·1 to 50·8)	55·7 (50·8 to 59·9)	9·1 (4·0 to 13·6)*	2·0 (−1·0 to 4·8)	7·2 (2·5 to 11·4)*	0·69 (0·31 to 1·01)*	0·41 (−0·21 to 1·02)	0·86 (0·31 to 1·35)*
		Bermuda	63·1 (60·8 to 65·8)	73·5 (71·0 to 76·0)	83·1 (79·7 to 86·3)	20·0 (15·7 to 24·0)*	10·4 (6·8 to 13·7)*	9·6 (5·6 to 13·5)*	1·06 (0·84 to 1·26)*	1·52 (0·98 to 2·01)*	0·76 (0·45 to 1·08)*
		Cuba	63·7 (62·4 to 65·5)	67·3 (66·2 to 68·6)	75·5 (73·5 to 77·7)	11·8 (9·5 to 14·2)*	3·6 (2·1 to 5·2)*	8·2 (6·0 to 10·4)*	0·65 (0·53 to 0·78)*	0·56 (0·32 to 0·79)*	0·72 (0·53 to 0·91)*
		Dominica	52·4 (50·1 to 54·8)	58·9 (56·3 to 61·2)	61·9 (58·2 to 65·3)	9·5 (5·3 to 13·2)*	6·5 (3·8 to 9·3)*	3·0 (−1·3 to 6·9)	0·64 (0·37 to 0·88)*	1·18 (0·69 to 1·67)*	0·31 (−0·14 to 0·71)
		Dominican Republic	38·4 (35·8 to 41·5)	52·5 (49·5 to 55·5)	61·2 (57·3 to 65·6)	22·8 (17·8 to 27·5)*	14·1 (9·6 to 18·1)*	8·7 (4·2 to 13·4)*	1·80 (1·40 to 2·14)*	3·14 (2·07 to 3·95)*	0·96 (0·46 to 1·44)*
		Grenada	47·2 (44·1 to 50·4)	53·2 (50·4 to 55·8)	58·5 (54·7 to 62·2)	11·3 (6·7 to 16·2)*	5·9 (2·0 to 9·6)*	5·3 (1·2 to 9·7)*	0·82 (0·49 to 1·19)*	1·19 (0·40 to 1·95)*	0·60 (0·13 to 1·07)*
		Guyana	38·4 (36·3 to 40·5)	43·2 (41·0 to 45·1)	49·8 (46·8 to 53·0)	11·4 (8·0 to 15·3)*	4·8 (1·9 to 7·2)*	6·6 (3·4 to 9·9)*	1·00 (0·71 to 1·32)*	1·19 (0·48 to 1·77)*	0·88 (0·46 to 1·32)*
		Haiti	16·7 (13·8 to 19·8)	23·2 (19·6 to 26·9)	32·1 (26·6 to 37·8)	15·4 (9·5 to 21·4)*	6·5 (2·0 to 10·9)*	8·9 (2·7 to 15·1)*	2·51 (1·59 to 3·48)*	3·30 (1·02 to 5·61)*	2·02 (0·65 to 3·35)*
		Jamaica	51·1 (48·2 to 54·2)	56·4 (52·4 to 59·8)	62·0 (56·8 to 67·3)	10·8 (5·0 to 16·7)*	5·2 (0·7 to 9·2)*	5·6 (0·2 to 10·9)*	0·74 (0·35 to 1·12)*	0·97 (0·13 to 1·69)*	0·59 (0·03 to 1·12)*
		Puerto Rico	67·1 (65·7 to 68·8)	74·6 (73·0 to 76·2)	82·7 (80·2 to 85·0)	15·6 (12·7 to 18·2)*	7·5 (5·7 to 9·4)*	8·1 (5·5 to 10·7)*	0·80 (0·66 to 0·93)*	1·06 (0·80 to 1·32)*	0·64 (0·45 to 0·84)*
		Saint Lucia	48·9 (46·6 to 51·1)	56·8 (54·5 to 58·9)	63·3 (60·3 to 66·0)	14·4 (10·9 to 17·7)*	7·9 (5·0 to 10·8)*	6·5 (3·3 to 9·7)*	1·00 (0·76 to 1·21)*	1·50 (0·95 to 2·07)*	0·68 (0·35 to 0·98)*
		Saint Vincent and the Grenadines	49·6 (47·2 to 51·7)	53·0 (50·7 to 55·1)	57·4 (54·8 to 59·9)	7·8 (4·6 to 11·1)*	3·4 (0·9 to 5·8)*	4·4 (1·5 to 7·6)*	0·56 (0·34 to 0·79)*	0·66 (0·18 to 1·14)*	0·50 (0·17 to 0·86)*
		Suriname	41·9 (39·9 to 44·2)	45·6 (43·0 to 47·9)	54·5 (51·2 to 57·6)	12·5 (8·3 to 16·4)*	3·6 (0·3 to 6·4)*	8·9 (5·6 to 12·4)*	1·01 (0·66 to 1·29)*	0·83 (0·07 to 1·47)*	1·12 (0·71 to 1·55)*
		The Bahamas	56·1 (54·0 to 58·3)	63·4 (61·3 to 65·4)	66·4 (62·9 to 69·7)	10·3 (6·3 to 14·0)*	7·3 (4·7 to 9·8)*	3·0 (−0·7 to 6·4)	0·65 (0·40 to 0·88)*	1·22 (0·79 to 1·67)*	0·29 (−0·06 to 0·61)
		Trinidad and Tobago	51·2 (49·7 to 52·6)	55·7 (53·7 to 57·3)	64·3 (60·7 to 67·5)	13·1 (9·0 to 16·6)*	4·5 (2·3 to 6·4)*	8·6 (5·3 to 11·8)*	0·87 (0·62 to 1·10)*	0·84 (0·43 to 1·18)*	0·89 (0·57 to 1·20)*
		Virgin Islands	57·2 (54·6 to 60·4)	65·7 (63·0 to 68·8)	74·0 (70·0 to 79·1)	16·8 (11·9 to 21·9)*	8·5 (4·9 to 12·1)*	8·3 (4·0 to 13·2)*	0·99 (0·72 to 1·28)*	1·38 (0·80 to 1·96)*	0·75 (0·36 to 1·18)*
	Central Latin America		43·3 (42·3 to 44·5)	55·8 (54·2 to 56·8)	64·4 (62·6 to 65·6)	21·1 (19·3 to 22·6)*	12·5 (10·8 to 13·7)*	8·6 (7·6 to 9·7)*	1·53 (1·40 to 1·63)*	2·54 (2·20 to 2·78)*	0·90 (0·79 to 1·01)*
		Colombia	48·5 (46·7 to 50·6)	57·6 (55·9 to 59·0)	68·5 (65·8 to 70·9)	20·0 (16·6 to 23·0)*	9·1 (6·9 to 11·0)*	10·9 (8·3 to 13·4)*	1·33 (1·11 to 1·53)*	1·72 (1·29 to 2·11)*	1·09 (0·84 to 1·31)*
		Costa Rica	60·7 (59·2 to 61·9)	64·7 (63·2 to 65·9)	73·7 (71·2 to 76·0)	13·0 (10·4 to 15·5)*	4·0 (2·5 to 5·5)*	9·0 (6·5 to 11·6)*	0·75 (0·60 to 0·88)*	0·64 (0·40 to 0·88)*	0·82 (0·60 to 1·04)*
		El Salvador	38·1 (35·9 to 41·8)	52·1 (49·5 to 54·5)	63·2 (58·9 to 67·2)	25·1 (17·9 to 29·7)*	14·0 (8·5 to 17·2)*	11·1 (7·6 to 15·0)*	1·95 (1·38 to 2·27)*	3·14 (1·86 to 3·86)*	1·20 (0·84 to 1·60)*
		Guatemala	30·4 (27·4 to 33·4)	42·0 (38·3 to 45·7)	51·5 (45·3 to 57·7)	21·1 (14·5 to 27·5)*	11·6 (7·1 to 16·1)*	9·4 (2·8 to 16·2)*	2·02 (1·42 to 2·57)*	3·24 (1·97 to 4·51)*	1·26 (0·38 to 2·10)*
		Honduras	28·1 (24·8 to 31·3)	38·1 (33·1 to 43·3)	46·5 (40·1 to 53·1)	18·5 (11·4 to 25·5)*	10·0 (5·5 to 15·2)*	8·5 (2·1 to 15·1)*	1·94 (1·26 to 2·65)*	3·04 (1·73 to 4·52)*	1·25 (0·32 to 2·21)*
		Mexico	45·5 (44·5 to 46·9)	59·0 (57·6 to 59·9)	66·3 (64·9 to 67·4)	20·8 (19·5 to 22·0)*	13·5 (12·0 to 14·6)*	7·3 (6·4 to 8·2)*	1·45 (1·34 to 1·54)*	2·61 (2·29 to 2·82)*	0·73 (0·64 to 0·82)*
		Nicaragua	43·1 (41·0 to 46·2)	49·8 (47·9 to 52·0)	61·2 (57·0 to 65·4)	18·1 (11·9 to 22·9)*	6·7 (3·1 to 9·6)*	11·4 (7·2 to 15·7)*	1·35 (0·88 to 1·67)*	1·45 (0·65 to 2·09)*	1·28 (0·83 to 1·74)*
		Panama	52·1 (49·3 to 55·5)	60·8 (58·6 to 62·9)	68·3 (64·6 to 71·9)	16·1 (10·8 to 21·2)*	8·7 (5·0 to 12·0)*	7·4 (3·3 to 11·6)*	1·04 (0·69 to 1·36)*	1·55 (0·86 to 2·15)*	0·72 (0·33 to 1·11)*
		Venezuela	51·3 (49·0 to 53·9)	60·0 (58·0 to 61·8)	67·8 (63·6 to 71·8)	16·5 (11·1 to 21·5)*	8·7 (5·4 to 11·6)*	7·8 (3·5 to 11·9)*	1·07 (0·74 to 1·38)*	1·57 (0·97 to 2·10)*	0·76 (0·35 to 1·15)*
	Tropical Latin America		46·1 (44·9 to 47·2)	54·9 (53·6 to 55·9)	63·4 (62·0 to 64·4)	17·3 (16·1 to 18·5)*	8·9 (7·9 to 9·7)*	8·4 (7·3 to 9·6)*	1·23 (1·14 to 1·31)*	1·76 (1·57 to 1·94)*	0·89 (0·77 to 1·02)*
		Brazil	46·5 (45·2 to 47·7)	55·3 (53·9 to 56·4)	63·8 (62·3 to 64·9)	17·3 (16·1 to 18·5)*	8·8 (8·0 to 9·6)*	8·5 (7·4 to 9·6)*	1·22 (1·13 to 1·30)*	1·74 (1·57 to 1·90)*	0·89 (0·78 to 1·02)*
		Paraguay	43·1 (41·1 to 45·1)	49·8 (46·8 to 52·3)	56·7 (53·1 to 60·2)	13·6 (9·9 to 17·4)*	6·8 (3·7 to 9·5)*	6·9 (3·4 to 10·3)*	1·06 (0·78 to 1·32)*	1·46 (0·82 to 2·07)*	0·81 (0·41 to 1·20)*
**North Africa and Middle East**†			**35·9 (33·7 to 37·9)**	**42·3 (40·5 to 44·0)**	**55·8 (54·0 to 57·8)**	19·9 (17·6 to 22·2)*	6·4 (5·1 to 7·6)*	13·5 (11·6 to 15·5)*	1·70 (1·49 to 1·93)*	1·63 (1·29 to 2·00)*	1·73 (1·50 to 2·00)*
	North Africa and Middle East		**35·9 (33·7 to 37·9)**	**42·3 (40·5 to 44·0)**	**55·8 (54·0 to 57·8)**	19·9 (17·6 to 22·2)*	6·4 (5·1 to 7·6)*	13·5 (11·6 to 15·5)*	1·70 (1·49 to 1·93)*	1·63 (1·29 to 2·00)*	1·73 (1·50 to 2·00)*
		Afghanistan	15·8 (12·2 to 19·4)	14·9 (11·5 to 19·1)	25·9 (22·0 to 29·5)	10·1 (5·2 to 14·5)*	−0·9 (−4·1 to 2·7)	11·0 (6·4 to 15·4)*	1·93 (0·96 to 2·83)*	−0·60 (−2·75 to 1·68)	3·51 (1·88 to 5·10)*
		Algeria	42·8 (37·6 to 46·7)	50·6 (46·1 to 54·2)	63·1 (59·4 to 66·4)	20·2 (16·0 to 24·6)*	7·8 (4·2 to 11·6)*	12·4 (8·7 to 16·7)*	1·49 (1·16 to 1·90)*	1·68 (0·89 to 2·56)*	1·38 (0·95 to 1·88)*
		Bahrain	49·9 (46·7 to 53·1)	59·4 (56·3 to 62·2)	72·0 (67·3 to 76·5)	22·1 (16·5 to 27·2)*	9·5 (5·4 to 13·6)*	12·6 (7·3 to 17·9)*	1·41 (1·07 to 1·73)*	1·75 (0·98 to 2·51)*	1·20 (0·71 to 1·68)*
		Egypt	34·2 (31·9 to 37·7)	45·9 (43·4 to 49·2)	58·0 (53·9 to 62·5)	23·8 (19·1 to 28·4)*	11·7 (8·8 to 14·5)*	12·1 (8·1 to 16·5)*	2·03 (1·64 to 2·39)*	2·94 (2·21 to 3·66)*	1·46 (1·00 to 1·95)*
		Iran	49·3 (45·0 to 53·5)	61·0 (57·2 to 64·7)	71·8 (67·3 to 76·3)	22·4 (16·3 to 28·6)*	11·6 (6·4 to 16·7)*	10·8 (5·0 to 16·3)*	1·44 (1·04 to 1·87)*	2·12 (1·16 to 3·11)*	1·02 (0·47 to 1·54)*
		Iraq	42·4 (38·5 to 47·1)	43·4 (40·0 to 46·8)	51·1 (45·9 to 56·6)	8·6 (1·2 to 15·8)*	0·9 (−3·8 to 5·6)	7·7 (1·6 to 13·7)*	0·71 (0·11 to 1·29)*	0·23 (−0·87 to 1·34)	1·02 (0·21 to 1·75)*
		Jordan	50·0 (46·5 to 53·4)	58·3 (53·8 to 62·7)	70·2 (64·8 to 75·3)	20·2 (13·5 to 26·3)*	8·3 (4·0 to 13·0)*	11·9 (5·4 to 18·4)*	1·31 (0·88 to 1·70)*	1·54 (0·76 to 2·41)*	1·16 (0·54 to 1·82)*
		Kuwait	66·8 (63·3 to 70·3)	70·8 (68·3 to 73·5)	80·7 (75·5 to 86·1)	13·8 (7·8 to 19·7)*	4·0 (−0·4 to 8·4)	9·9 (4·4 to 15·4)*	0·72 (0·42 to 1·02)*	0·58 (−0·05 to 1·22)	0·81 (0·37 to 1·25)*
		Lebanon	53·1 (48·5 to 57·1)	67·2 (63·6 to 70·6)	85·6 (82·8 to 88·2)	32·5 (27·5 to 38·0)*	14·1 (9·8 to 18·5)*	18·4 (14·2 to 23·0)*	1·84 (1·52 to 2·21)*	2·36 (1·60 to 3·19)*	1·52 (1·15 to 1·93)*
		Libya	50·9 (46·8 to 54·5)	57·9 (54·5 to 61·0)	71·1 (67·4 to 74·6)	20·2 (15·7 to 24·7)*	7·0 (4·1 to 9·9)*	13·2 (9·5 to 16·8)*	1·29 (1·00 to 1·60)*	1·30 (0·74 to 1·87)*	1·28 (0·93 to 1·65)*
		Morocco	37·5 (34·7 to 40·7)	44·6 (41·5 to 47·5)	57·6 (54·5 to 60·8)	20·1 (16·2 to 23·6)*	7·1 (4·1 to 10·0)*	13·0 (9·9 to 16·1)*	1·65 (1·33 to 1·95)*	1·73 (0·99 to 2·45)*	1·60 (1·23 to 2·00)*
		Oman	52·5 (49·6 to 55·5)	63·4 (61·1 to 65·9)	76·2 (74·0 to 78·6)	23·7 (20·4 to 27·1)*	10·9 (8·4 to 13·6)*	12·8 (10·0 to 15·4)*	1·43 (1·21 to 1·67)*	1·89 (1·44 to 2·38)*	1·15 (0·89 to 1·38)*
		Palestine	48·1 (43·1 to 53·5)	54·1 (51·2 to 57·6)	57·4 (54·1 to 60·6)	9·3 (2·7 to 15·3)*	6·0 (0·4 to 11·7)*	3·3 (−1·0 to 7·2)	0·68 (0·20 to 1·15)*	1·19 (0·07 to 2·37)*	0·37 (−0·11 to 0·80)
		Qatar	57·7 (53·3 to 62·2)	64·6 (60·3 to 69·1)	81·7 (75·9 to 86·6)	23·9 (16·8 to 30·8)*	6·9 (1·0 to 12·9)*	17·0 (10·4 to 24·0)*	1·33 (0·94 to 1·74)*	1·13 (0·16 to 2·11)*	1·46 (0·89 to 2·03)*
		Saudi Arabia	49·9 (47·0 to 53·0)	56·6 (54·8 to 58·7)	77·1 (74·9 to 79·3)	27·2 (23·4 to 31·2)*	6·7 (3·9 to 9·7)*	20·5 (17·8 to 23·2)*	1·67 (1·42 to 1·95)*	1·26 (0·72 to 1·85)*	1·93 (1·69 to 2·18)*
		Sudan	28·6 (24·3 to 31·8)	33·7 (29·8 to 36·7)	45·8 (41·0 to 50·0)	17·2 (13·1 to 21·3)*	5·1 (2·5 to 8·0)*	12·1 (8·0 to 16·0)*	1·81 (1·37 to 2·28)*	1·65 (0·76 to 2·66)*	1·91 (1·31 to 2·51)*
		Syria	45·5 (42·6 to 48·3)	56·7 (54·6 to 58·8)	67·2 (64·4 to 70·2)	21·7 (17·9 to 25·7)*	11·2 (8·1 to 14·5)*	10·5 (7·1 to 14·0)*	1·50 (1·23 to 1·79)*	2·21 (1·58 to 2·94)*	1·06 (0·74 to 1·41)*
		Tunisia	47·6 (43·2 to 50·9)	59·0 (55·3 to 62·3)	69·4 (65·4 to 73·7)	21·8 (17·1 to 26·8)*	11·4 (8·1 to 14·7)*	10·4 (6·6 to 14·3)*	1·45 (1·14 to 1·83)*	2·15 (1·50 to 2·85)*	1·02 (0·64 to 1·40)*
		Turkey	42·5 (38·8 to 46·3)	53·9 (50·8 to 56·8)	74·4 (70·0 to 78·4)	31·9 (26·2 to 37·3)*	11·4 (7·9 to 15·2)*	20·4 (15·5 to 25·2)*	2·16 (1·76 to 2·53)*	2·39 (1·61 to 3·22)*	2·01 (1·53 to 2·50)*
		United Arab Emirates	49·8 (43·7 to 55·4)	60·2 (56·0 to 64·4)	70·3 (65·5 to 75·4)	20·5 (12·8 to 28·6)*	10·4 (5·2 to 15·8)*	10·1 (4·1 to 16·6)*	1·33 (0·81 to 1·90)*	1·91 (0·93 to 3·00)*	0·97 (0·39 to 1·59)*
		Yemen	25·2 (20·8 to 29·1)	31·4 (26·9 to 35·6)	43·3 (38·3 to 47·9)	18·1 (12·9 to 22·7)*	6·2 (2·6 to 9·9)*	11·9 (7·5 to 16·2)*	2·09 (1·45 to 2·72)*	2·20 (0·92 to 3·57)*	2·01 (1·22 to 2·75)*
**South Asia**†			**23·8 (22·3 to 25·6)**	**27·6 (26·1 to 29·3)**	**40·4 (38·7 to 42·2)**	16·6 (14·0 to 18·9)*	3·8 (2·1 to 5·2)*	12·9 (10·9 to 14·8)*	2·04 (1·70 to 2·32)*	1·47 (0·84 to 2·09)*	2·39 (2·01 to 2·77)*
	South Asia		**23·8 (22·3 to 25·6)**	**27·6 (26·1 to 29·3)**	**40·4 (38·7 to 42·2)**	16·6 (14·0 to 18·9)*	3·8 (2·1 to 5·2)*	12·9 (10·9 to 14·8)*	2·04 (1·70 to 2·32)*	1·47 (0·84 to 2·09)*	2·39 (2·01 to 2·77)*
		Bangladesh	17·8 (15·0 to 20·7)	27·5 (25·2 to 30·0)	47·6 (44·3 to 50·9)	29·8 (25·7 to 34·2)*	9·7 (6·5 to 12·8)*	20·1 (16·3 to 23·8)*	3·80 (3·18 to 4·50)*	4·36 (2·84 to 6·05)*	3·44 (2·78 to 4·09)*
		Bhutan	20·0 (16·2 to 23·9)	29·6 (26·1 to 33·1)	47·3 (42·6 to 52·0)	27·2 (22·1 to 32·6)*	9·6 (5·7 to 13·5)*	17·7 (13·1 to 22·3)*	3·32 (2·58 to 4·11)*	3·94 (2·29 to 5·80)*	2·93 (2·18 to 3·70)*
		India	24·7 (22·9 to 27·2)	28·0 (26·3 to 30·3)	41·2 (39·1 to 43·4)	16·5 (13·4 to 19·4)*	3·3 (1·3 to 5·5)*	13·2 (10·7 to 15·6)*	1·97 (1·56 to 2·31)*	1·27 (0·46 to 2·03)*	2·41 (1·93 to 2·85)*
		Nepal	21·0 (18·1 to 24·1)	26·5 (23·7 to 29·4)	40·0 (36·5 to 44·4)	19·1 (14·6 to 23·9)*	5·5 (2·5 to 8·5)*	13·6 (10·0 to 17·6)*	2·49 (1·90 to 3·14)*	2·33 (1·05 to 3·69)*	2·59 (1·94 to 3·31)*
		Pakistan	26·8 (24·0 to 30·0)	27·4 (24·9 to 30·5)	37·6 (33·7 to 41·9)	10·8 (6·1 to 15·5)*	0·6 (−2·4 to 3·5)	10·2 (5·7 to 14·6)*	1·30 (0·73 to 1·86)*	0·22 (−0·86 to 1·32)	1·98 (1·11 to 2·77)*
**Sub–Saharan Africa**†			**19·6 (18·2 to 21·1)**	**22·3 (20·9 to 23·8)**	**31·9 (30·5 to 33·7)**	12·3 (10·5 to 14·1)*	2·7 (1·4 to 4·1)*	9·6 (8·0 to 11·3)*	1·88 (1·58 to 2·17)*	1·30 (0·65 to 1·96)*	2·24 (1·85 to 2·65)*
	Central sub–Saharan Africa		19·6 (16·6 to 22·9)	20·6 (17·4 to 24·2)	29·2 (25·8 to 32·7)	9·7 (6·0 to 13·1)*	1·1 (−1·7 to 3·8)	8·6 (5·2 to 11·8)*	1·55 (0·96 to 2·19)*	0·54 (−0·86 to 1·87)	2·18 (1·26 to 3·11)*
		Angola	18·4 (12·7 to 24·4)	20·6 (14·2 to 27·2)	33·4 (25·5 to 40·4)	14·9 (7·2 to 22·6)*	2·2 (−2·6 to 6·9)	12·8 (6·1 to 19·7)*	2·31 (1·09 to 3·64)*	1·11 (−1·26 to 3·57)	3·06 (1·34 to 4·95)*
		Central African Republic	15·8 (12·7 to 19·6)	16·1 (11·2 to 22·1)	18·6 (13·1 to 24·4)	2·7 (−3·2 to 8·9)	0·3 (−4·7 to 5·4)	2·4 (−3·6 to 8·6)	0·59 (−0·80 to 1·85)	0·10 (−3·25 to 2·99)	0·89 (−1·33 to 3·19)
		Congo (Brazzaville)	21·0 (17·0 to 25·1)	21·9 (18·0 to 25·9)	34·1 (28·4 to 40·4)	13·0 (6·7 to 20·0)*	0·8 (−3·3 to 5·2)	12·2 (6·4 to 18·6)*	1·86 (0·96 to 2·84)*	0·40 (−1·49 to 2·44)	2·77 (1·49 to 4·19)*
		Democratic Republic of the Congo	21·7 (17·6 to 26·4)	22·1 (17·5 to 27·0)	29·6 (25·7 to 33·7)	7·9 (2·8 to 12·7)*	0·4 (−3·6 to 4·4)	7·5 (2·9 to 11·7)*	1·21 (0·44 to 2·00)*	0·19 (−1·59 to 1·98)	1·85 (0·69 to 2·94)*
		Equatorial Guinea	13·9 (8·9 to 19·3)	25·7 (18·7 to 34·1)	49·3 (38·3 to 62·0)	35·4 (24·4 to 47·7)*	11·8 (6·1 to 18·5)*	23·6 (13·3 to 33·8)*	4·90 (3·41 to 6·58)*	6·18 (3·18 to 9·59)*	4·11 (2·43 to 5·84)*
		Gabon	27·7 (24·2 to 31·4)	28·6 (24·5 to 32·9)	40·4 (35·0 to 46·1)	12·7 (6·6 to 18·9)*	0·9 (−3·7 to 5·4)	11·8 (5·4 to 17·9)*	1·45 (0·76 to 2·13)*	0·30 (−1·35 to 1·87)	2·17 (1·01 to 3·30)*
	Eastern sub–Saharan Africa		15·0 (13·3 to 16·8)	18·8 (17·0 to 20·6)	29·2 (27·3 to 31·3)	14·2 (11·9 to 16·4)*	3·7 (1·9 to 5·5)*	10·5 (8·4 to 12·5)*	2·56 (2·11 to 3·03)*	2·22 (1·12 to 3·31)*	2·77 (2·18 to 3·34)*
		Burundi	14·3 (10·7 to 18·2)	17·7 (14·2 to 21·3)	27·4 (23·1 to 32·1)	13·1 (7·3 to 18·2)*	3·4 (−0·8 to 7·5)	9·7 (4·6 to 14·5)*	2·52 (1·40 to 3·70)*	2·19 (−0·51 to 4·93)	2·73 (1·29 to 4·14)*
		Comoros	19·4 (16·1 to 23·1)	23·4 (20·3 to 26·4)	33·0 (29·5 to 36·7)	13·6 (8·5 to 18·2)*	3·9 (0·5 to 7·4)*	9·6 (5·6 to 13·7)*	2·05 (1·24 to 2·82)*	1·87 (0·20 to 3·57)*	2·16 (1·25 to 3·06)*
		Djibouti	23·1 (20·2 to 26·6)	24·3 (19·8 to 30·0)	35·0 (29·7 to 42·0)	11·8 (5·8 to 19·1)*	1·1 (−3·5 to 6·3)	10·7 (5·6 to 15·9)*	1·58 (0·78 to 2·41)*	0·45 (−1·58 to 2·47)	2·29 (1·22 to 3·43)*
		Eritrea	12·2 (9·2 to 15·6)	20·7 (17·3 to 24·3)	27·6 (23·7 to 31·3)	15·4 (10·7 to 19·9)*	8·5 (5·1 to 12·1)*	6·9 (2·8 to 10·8)*	3·16 (2·12 to 4·28)*	5·33 (3·13 to 7·94)*	1·81 (0·71 to 2·89)*
		Ethiopia	10·6 (7·8 to 14·1)	14·0 (11·1 to 17·3)	28·1 (24·3 to 32·2)	17·5 (12·2 to 22·1)*	3·5 (−0·5 to 7·2)	14·1 (9·3 to 18·9)*	3·79 (2·53 to 5·04)*	2·88 (−0·38 to 6·12)	4·36 (2·85 to 6·01)*
		Kenya	32·4 (27·6 to 37·4)	32·3 (28·0 to 36·8)	39·5 (35·0 to 43·9)	7·1 (3·3 to 11·0)*	−0·1 (−3·1 to 2·6)	7·2 (4·2 to 10·2)*	0·76 (0·33 to 1·20)*	−0·03 (−0·96 to 0·82)	1·26 (0·73 to 1·81)*
		Madagascar	20·6 (18·0 to 23·2)	23·8 (21·0 to 26·9)	29·6 (24·3 to 35·1)	9·0 (3·5 to 15·0)*	3·3 (0·1 to 6·6)*	5·8 (−0·1 to 11·6)	1·39 (0·57 to 2·23)*	1·47 (0·05 to 2·94)*	1·34 (−0·02 to 2·61)
		Malawi	19·0 (13·9 to 25·5)	21·5 (14·8 to 31·9)	32·2 (26·9 to 38·2)	13·2 (6·3 to 20·1)*	2·5 (−2·7 to 8·9)	10·7 (1·0 to 19·3)*	2·06 (0·96 to 3·30)*	1·15 (−1·45 to 3·95)	2·63 (0·19 to 5·04)*
		Mozambique	13·8 (11·0 to 17·0)	21·1 (15·9 to 28·1)	30·0 (25·3 to 35·0)	16·3 (11·2 to 21·4)*	7·3 (2·2 to 13·6)*	9·0 (2·0 to 15·2)*	3·01 (2·10 to 3·93)*	4·19 (1·50 to 7·25)*	2·27 (0·45 to 4·02)*
		Rwanda	16·7 (13·0 to 20·8)	18·6 (14·4 to 22·8)	36·0 (31·6 to 40·5)	19·2 (14·1 to 24·1)*	1·8 (−2·1 to 5·6)	17·4 (12·1 to 22·7)*	2·96 (2·06 to 3·90)*	1·05 (−1·22 to 3·27)	4·16 (2·77 to 5·75)*
		Somalia	12·8 (8·2 to 18·3)	13·5 (9·1 to 19·1)	19·0 (14·3 to 23·7)	6·2 (0·6 to 11·1)*	0·7 (−2·8 to 3·7)	5·5 (0·5 to 9·8)*	1·56 (0·13 to 3·01)*	0·56 (−2·17 to 3·03)	2·19 (0·19 to 4·19)*
		South Sudan	22·0 (16·8 to 28·9)	23·6 (17·4 to 30·7)	26·8 (21·0 to 33·1)	4·9 (−2·0 to 11·2)	1·6 (−3·4 to 6·6)	3·3 (−2·7 to 9·0)	0·78 (−0·31 to 1·81)	0·69 (−1·51 to 2·87)	0·84 (−0·66 to 2·31)
		Tanzania	21·9 (18·7 to 25·5)	24·7 (20·5 to 30·1)	33·9 (30·0 to 38·4)	11·9 (7·3 to 16·6)*	2·7 (−1·5 to 7·4)	9·2 (4·1 to 14·3)*	1·67 (1·01 to 2·35)*	1·15 (−0·68 to 2·99)	2·00 (0·83 to 3·19)*
		Uganda	19·3 (15·6 to 23·5)	23·7 (20·0 to 27·7)	31·4 (27·2 to 35·6)	12·1 (7·2 to 16·8)*	4·4 (0·5 to 8·4)*	7·8 (3·2 to 12·4)*	1·89 (1·11 to 2·70)*	2·06 (0·25 to 3·95)*	1·78 (0·73 to 2·88)*
		Zambia	21·9 (17·6 to 27·2)	17·2 (13·0 to 22·7)	29·0 (23·0 to 35·4)	7·1 (0·4 to 14·8)*	−4·7 (−9·2 to 0·3)	11·7 (5·0 to 19·1)*	1·08 (0·07 to 2·22)*	−2·44 (−4·83 to 0·13)	3·28 (1·32 to 5·30)*
	Southern sub–Saharan Africa		38·2 (36·3 to 40·4)	37·8 (34·8 to 40·6)	44·7 (42·4 to 47·0)	6·5 (3·8 to 9·1)*	−0·4 (−3·0 to 2·2)	7·0 (3·8 to 10·1)*	0·61 (0·34 to 0·84)*	−0·11 (−0·81 to 0·55)	1·06 (0·58 to 1·56)*
		Botswana	36·5 (30·6 to 43·0)	39·7 (22·3 to 55·7)	51·5 (40·8 to 69·2)	15·0 (3·5 to 32·8)*	3·2 (−11·5 to 17·2)	11·8 (−8·6 to 34·7)	1·31 (0·32 to 2·57)*	0·54 (−4·08 to 3·84)	1·79 (−1·07 to 5·36)
		Lesotho	30·3 (25·9 to 35·5)	29·2 (23·0 to 38·0)	32·0 (24·6 to 40·3)	1·6 (−6·2 to 10·0)	−1·2 (−7·8 to 7·5)	2·8 (−6·1 to 12·2)	0·19 (−0·81 to 1·17)	−0·46 (−2·73 to 2·36)	0·59 (−1·18 to 2·52)
		Namibia	27·5 (24·6 to 31·1)	32·2 (24·1 to 43·3)	44·6 (36·4 to 56·2)	17·1 (9·4 to 27·7)*	4·7 (−2·9 to 15·7)	12·4 (3·2 to 20·9)*	1·84 (1·14 to 2·71)*	1·45 (−1·08 to 4·60)	2·09 (0·49 to 3·62)*
		South Africa	40·1 (38·0 to 42·3)	40·9 (38·2 to 43·8)	49·7 (47·2 to 52·4)	9·6 (6·6 to 12·7)*	0·8 (−2·3 to 3·9)	8·8 (5·4 to 12·1)*	0·83 (0·57 to 1·08)*	0·19 (−0·56 to 0·95)	1·23 (0·75 to 1·70)*
		Swaziland	32·0 (27·3 to 37·0)	34·4 (22·6 to 43·6)	40·5 (30·4 to 52·2)	8·5 (−1·2 to 18·4)	2·4 (−11·1 to 13·5)	6·1 (−9·6 to 20·6)	0·88 (−0·14 to 1·78)	0·59 (−3·91 to 3·86)	1·06 (−1·64 to 3·73)
		Zimbabwe	37·3 (31·2 to 48·0)	31·4 (22·6 to 39·7)	31·2 (25·8 to 37·0)	−6·1 (−17·7 to 1·0)	−5·9 (−12·0 to 0·0)	−0·2 (−9·5 to 9·2)	−0·68 (−1·81 to 0·11)	−1·81 (−3·80 to 0·01)	0·02 (−1·79 to 2·04)
	Western sub–Saharan Africa		22·4 (20·3 to 24·4)	24·8 (22·4 to 27·2)	34·3 (31·9 to 36·7)	11·9 (9·2 to 14·6)*	2·4 (0·1 to 4·9)*	9·5 (6·5 to 12·6)*	1·64 (1·26 to 2·04)*	1·03 (0·05 to 2·04)*	2·02 (1·35 to 2·74)*
		Benin	19·7 (16·9 to 22·7)	22·7 (19·7 to 26·0)	30·8 (27·8 to 34·0)	11·2 (7·2 to 15·2)*	3·1 (−0·1 to 6·4)	8·1 (4·3 to 11·9)*	1·74 (1·09 to 2·38)*	1·45 (−0·06 to 3·08)	1·92 (0·98 to 2·82)*
		Burkina Faso	16·4 (13·4 to 20·3)	21·9 (18·7 to 25·6)	30·1 (27·0 to 33·3)	13·7 (9·2 to 17·6)*	5·6 (2·1 to 9·0)*	8·2 (4·2 to 12·1)*	2·36 (1·46 to 3·10)*	2·96 (1·07 to 4·76)*	1·99 (0·99 to 3·02)*
		Cameroon	23·4 (20·6 to 26·8)	23·8 (19·7 to 28·1)	31·9 (26·9 to 37·5)	8·5 (3·3 to 14·3)*	0·4 (−3·8 to 4·5)	8·2 (2·9 to 13·3)*	1·19 (0·49 to 1·92)*	0·13 (−1·73 to 1·88)	1·85 (0·69 to 3·00)*
		Cape Verde	38·1 (35·4 to 41·2)	41·4 (37·0 to 46·1)	54·8 (51·2 to 58·9)	16·7 (12·5 to 21·2)*	3·3 (−1·3 to 7·8)	13·4 (7·6 to 19·5)*	1·40 (1·03 to 1·76)*	0·81 (−0·34 to 1·91)	1·76 (0·99 to 2·61)*
		Chad	18·3 (15·6 to 21·4)	18·2 (15·3 to 21·5)	25·4 (21·9 to 29·0)	7·1 (2·8 to 11·6)*	−0·1 (−3·5 to 3·3)	7·2 (3·2 to 11·1)*	1·27 (0·49 to 2·05)*	−0·05 (−1·92 to 1·81)	2·09 (0·93 to 3·27)*
		Côte d'Ivoire	19·9 (17·3 to 22·6)	20·7 (17·1 to 24·3)	27·3 (24·2 to 31·1)	7·5 (3·3 to 11·1)*	0·8 (−2·5 to 4·3)	6·7 (2·5 to 10·8)*	1·23 (0·56 to 1·83)*	0·37 (−1·29 to 2·05)	1·76 (0·61 to 2·95)*
		Ghana	25·6 (22·5 to 28·9)	29·6 (26·2 to 33·5)	39·3 (36·0 to 43·4)	13·6 (9·1 to 18·4)*	4·0 (0·1 to 8·0)*	9·7 (5·1 to 14·1)*	1·64 (1·08 to 2·25)*	1·45 (0·04 to 2·82)*	1·77 (0·93 to 2·62)*
		Guinea	17·1 (14·3 to 20·3)	20·1 (17·2 to 23·0)	26·4 (22·6 to 30·2)	9·2 (4·5 to 14·2)*	2·9 (−0·6 to 6·3)	6·3 (2·2 to 10·8)*	1·66 (0·80 to 2·54)*	1·58 (−0·31 to 3·49)	1·71 (0·62 to 2·85)*
		Guinea–Bissau	12·8 (10·0 to 16·0)	15·7 (12·7 to 19·0)	23·4 (20·2 to 26·8)	10·6 (5·9 to 14·9)*	2·9 (−0·6 to 6·7)	7·7 (3·6 to 11·9)*	2·34 (1·25 to 3·36)*	2·03 (−0·40 to 4·67)	2·53 (1·17 to 3·97)*
		Liberia	20·5 (17·6 to 23·6)	23·2 (19·8 to 26·8)	32·2 (29·3 to 35·4)	11·7 (8·0 to 15·5)*	2·8 (−0·7 to 6·6)	8·9 (4·9 to 13·0)*	1·74 (1·15 to 2·36)*	1·26 (−0·31 to 2·98)	2·04 (1·09 to 3·07)*
		Mali	16·7 (13·7 to 20·5)	23·7 (20·4 to 27·2)	34·9 (29·9 to 40·1)	18·2 (12·6 to 23·8)*	7·0 (2·9 to 10·6)*	11·2 (5·8 to 16·7)*	2·85 (1·97 to 3·68)*	3·53 (1·38 to 5·44)*	2·43 (1·30 to 3·59)*
		Mauritania	24·0 (20·8 to 27·5)	29·7 (25·9 to 36·2)	40·6 (35·0 to 47·5)	16·6 (10·7 to 23·7)*	5·7 (1·8 to 11·5)*	10·9 (4·9 to 17·2)*	2·02 (1·35 to 2·70)*	2·13 (0·68 to 3·91)*	1·95 (0·86 to 2·98)*
		Niger	15·6 (12·6 to 19·3)	19·1 (16·0 to 22·3)	28·4 (23·9 to 33·1)	12·8 (7·2 to 18·1)*	3·5 (−0·1 to 7·4)	9·3 (3·8 to 14·7)*	2·30 (1·27 to 3·24)*	2·02 (−0·06 to 4·21)	2·48 (1·06 to 3·87)*
		Nigeria	27·5 (23·4 to 31·6)	29·8 (24·9 to 35·3)	41·9 (37·2 to 47·3)	14·4 (8·7 to 20·4)*	2·3 (−2·6 to 7·7)	12·1 (5·5 to 19·0)*	1·62 (0·97 to 2·35)*	0·80 (−0·92 to 2·66)	2·14 (0·93 to 3·38)*
		São Tomé and Principe	25·9 (22·4 to 29·7)	30·0 (25·7 to 40·5)	39·3 (34·9 to 44·4)	13·4 (8·2 to 19·2)*	4·2 (−0·1 to 13·3)	9·3 (−0·4 to 14·9)	1·61 (0·97 to 2·28)*	1·46 (−0·05 to 4·06)	1·71 (−0·06 to 2·74)
		Senegal	22·4 (19·8 to 25·1)	24·5 (22·0 to 27·3)	31·1 (28·3 to 33·8)	8·6 (5·3 to 11·9)*	2·0 (−0·8 to 5·1)	6·6 (3·4 to 9·7)*	1·26 (0·75 to 1·77)*	0·87 (−0·33 to 2·21)	1·49 (0·73 to 2·21)*
		Sierra Leone	20·8 (17·4 to 24·6)	22·1 (19·0 to 25·5)	31·0 (27·4 to 34·5)	10·1 (5·7 to 14·5)*	1·3 (−2·0 to 4·6)	8·8 (4·3 to 12·8)*	1·53 (0·84 to 2·22)*	0·60 (−0·94 to 2·25)	2·11 (1·05 to 3·10)*
		The Gambia	27·4 (23·9 to 30·9)	29·9 (26·5 to 33·4)	35·7 (32·3 to 39·3)	8·3 (4·1 to 12·9)*	2·5 (−0·7 to 5·6)	5·8 (1·9 to 9·6)*	1·02 (0·50 to 1·58)*	0·87 (−0·24 to 2·02)	1·12 (0·38 to 1·82)*
		Togo	21·7 (19·0 to 24·5)	23·0 (19·1 to 27·9)	32·0 (28·7 to 35·6)	10·2 (6·3 to 14·3)*	1·2 (−3·0 to 6·0)	9·0 (4·0 to 13·6)*	1·48 (0·89 to 2·09)*	0·52 (−1·46 to 2·53)	2·09 (0·85 to 3·24)*

HAQ Index=Healthcare Access and Quality Index. UI=uncertainty interval.

* Significant change during this time period.

† Refers to Global Burden of Disease super region.

Focusing on 2000–16, examining improvement across health areas highlights a mixture of progress and potential for worsening performance if past trends are not addressed (appendix pp 136–41). Across locations, the largest gains primarily took place for vaccine-preventable diseases (eg, measles), some infectious diseases (eg, diarrhoeal diseases), some cancers (eg, leukaemia), and some non-communicable diseases. Such advances were most pronounced among countries that also recorded substantive increases in their overall HAQ Index (eg, China, Turkey). At the same time, many low-to-middle SDI countries experienced relatively few gains across most non-communicable diseases. Furthermore, countries with minimal progress on overall HAQ Index performance had comparatively small advances, even for health areas in which improvements have been more widespread. The main exception was vaccine-preventable diseases, especially measles, for low-SDI to middle-SDI countries (appendix pp 136–41).

### Correlates of HAQ Index performance

**Figure 6:**
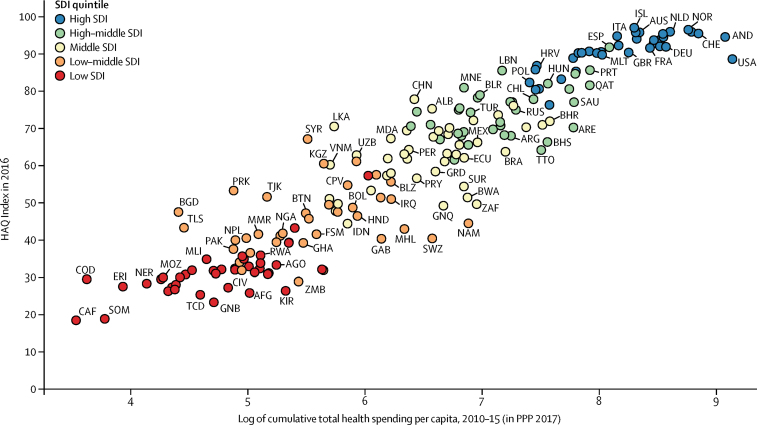
Comparing the HAQ Index in 2016 to the log of cumulative total health spending per capita, 2010–15 Total health spending per capita is based on the cumulative per capita spending from 2010 to 2015 in purchasing power parity (PPP) for 2017. Countries and territories are colour-coded by their SDI quintile, and are abbreviated according to their ISO3 codes, which are listed in the appendix (pp 90–95). HAQ Index=Healthcare Access and Quality Index. SDI=Socio-demographic Index.

Although total health spending per capita was strongly correlated with HAQ Index performance in 2016 (*r*=0·94; figure 6), large variation existed at similar spending levels. For instance, some countries with HAQ Index scores between 40 and 70 spent at least three times more than did peers with similar performance. Government spending as a fraction of total health spending had positive, albeit moderate, correlation with HAQ Index performance in 2016 (*r*=0·76; appendix p 145), whereas development assistance for health showed an opposite pattern (*r*=–0·71; appendix p 147). Country-level HAQ Index scores in 2016 were positively associated with physicians, nurses, and midwives per 1000 (*r*=0·79), and similar, though more moderate, correlations were found for hospital beds per 1000 and utilisation (appendix pp 149–52). Nonetheless, sizeable heterogeneity emerged across these health system measures and their relationships to the HAQ Index, particularly among middle-to-high SDI countries. All correlations and additional figures are in the appendix (pp 142–52, 165).

## Discussion

### Summary of findings

Amid gains on personal health-care access and quality, striking disparities remained regarding HAQ Index scores achieved by 2016, and how quickly locations improved over time. In 2016, HAQ Index performance diverged along the development spectrum, ranging from more than 97 in Iceland to less than 20 in the Central African Republic and Somalia. Subnational inequalities were particularly pronounced in China and India, although high-income countries, including England and the USA, also saw considerable local gaps in performance. The global pace of progress accelerated from 2000 to 2016, a trend fuelled by many low-SDI and low-middle-SDI countries in sub-Saharan Africa and southeast Asia. By contrast, several countries saw slowed or minimal improvement from 2000 to 2016 after recording larger gains from 1990 to 2000. Examining patterns in broader causes unveiled considerable heterogeneity in country-level improvements across health areas. These findings, coupled with the variable relationships between national HAQ Index values and potential correlates of performance, underscore the complexities of orienting health systems toward providing access to quality services across health needs and along continuums of care.

### Inequalities in personal health-care access and quality within countries

Our subnational assessment of HAQ Index performance shows the importance of monitoring health-care gaps and gains at more local levels. Further, because some factors might be more uniform because of country-level policy or health-care characteristics (eg, national insurance schemes, federally-maintained referral systems), this analysis offers the opportunity to consider if or how challenges in access and quality are experienced within countries. For instance, Mexico's subnational differences could be more related to state-level variations in quality given the country's concerted efforts to expand access and service coverage through a tiered insurance system.^42^, ^43^ Similar factors might underlie disparities in England, where the National Health Service ought to minimise financial barriers to accessing health care.^30^ Nonetheless, other obstacles probably exist, including inadequate utilisation of care across Mexican states,^44^ and local variations in health funding^45^ or human resource constraints within England.^46^ Striking disparities in China and India might represent myriad factors, including large variations in physical access to health facilities, health system infrastructure and scale-up of medical technologies, and provision of effective services across continuums of care. Brazil's universal health coverage-focused initiatives, including expanding community-based health programmes and governance functions, seem to have contributed to local reductions in amenable mortality from 2000 to 2012.^14^ However, state-level progress on the HAQ Index was generally faster from 1990 to 2000 than from 2000 to 2016, suggesting that advances in access might not always be accompanied by improved quality of care across health services, especially for non-communicable diseases. State-level differences in the USA could be linked to the country's widely acknowledged challenges in providing good health-care access to all populations,^13^, ^47^ and disparities in the quality of care found in its poorer regions.^13^ As future iterations of GBD endeavour to support subnational burden of disease assessments for more countries, we aim to expand locally focused monitoring of health-care access and quality in tandem.

### Pace of past progress and strengthening health systems for the next generation

Current HAQ Index estimates represent the culmination of past health-care policy actions, and thus offer an important entry point for strengthening health systems for the future. Recent demographic and epidemiological trends point to populations living longer and with higher disease burden worldwide,^48^ portending an escalation of health-care challenges if countries cannot more expediently shift their models of care away from reactive service delivery and toward more proactive continuums of care. Such action must be accompanied by efforts to further bolster public health programmes and policies, targeting risk factors and socioeconomic factors that are less directly amenable to health care but remain leading contributors to preventable disease burden (eg, smoking).^16^

Historically, global health priorities centred on a subset of health services (ie, vaccine-preventable diseases, infectious diseases, and maternal and child health), which was particularly true during the Millennium Development Goal (MDG) era. Successes in scaling up vaccine coverage, early diagnosis and treatment of infectious disease (eg, antibiotics for lower respiratory infections), and improving access to and quality of maternal care and delivery are illustrated by accelerated HAQ Index performance for many low-to-middle SDI countries from 2000 to 2016. The exact drivers of these improvements vary by context (eg, Timor-Leste emerged from years of conflict in the late 1990s; political strife and HIV devastated health systems throughout sub-Saharan Africa during the 1990s and early 2000s), but some combination of domestic policy action and increased development assistance for health might have hastened progress in many countries.^49^

In parallel, poor access to or quality of non-communicable disease-focused risk management and treatment could explain slower gains or minimal advances for these causes in many countries, a warning sign that health systems are not evolving at the same rate as changing population health needs. For non-communicable diseases, there was a strong divide in performance among high-SDI countries and low-to-middle SDI locations, potentially reflecting inadequate investments in advancing non-communicable disease services across continuums of care, integrating care across health areas, or some combination of both. The importance of, and potential for, improving non-communicable disease prevention and treatment is shown by trends from eastern Europe and central Asia,^50^, ^51^ where several countries saw substantive HAQ Index gains from 2000 to 2016 after stagnation or worsening performance during the 1990s.

Gains made against vaccine-preventable diseases and other causes prioritised during the MDGs must be sustained going forward, but not at the expense of preparing health systems for the next generation. Amid shifting epidemiological profiles,^48^ countries including China, Turkey, Vietnam, and Nepal recorded consistently sizeable rates of progress on the HAQ Index from 1990 to 2000, and 2000 to 2016. Such trends could reflect several factors (eg, health system structures, governance functions, health insurance expansion),^52^, ^53^, ^54^, ^55^ but also could represent successes in re-orienting and integrating services to accommodate evolving health-care needs.^56^

Finally, some countries did not experience such catalytic effects during the MDGs and are at risk of falling further behind in the SDG era. These locations include the Central African Republic, Somalia, and South Sudan, which consistently recorded among the lowest HAQ Index scores over time; and Zimbabwe and Lesotho, countries that have struggled to recover from faltering performance during the 1990s and early 2000s. Again, the precise factors underlying these countries' challenges are multifaceted, but commonalities include prolonged conflict, widespread poverty, and comparatively low levels of development assistance for health from development partners.^39^

### Progress towards universal health coverage

Providing access to quality health care is a key component of universal health coverage, and the HAQ Index offers a robust metric for monitoring progress across health service areas. This strength is particularly important since achieving universal health coverage is an objective for countries across the development spectrum, and thus comparable measures are needed for benchmarking progress and identifying specific health areas for policy action.^57^ For instance, gains in performance on neonatal disorders generally lagged behind those of maternal disorders in many low-to-middle SDI countries, which suggests that greater investment across the continuum of care, from antenatal services to neonatal intensive care units, might support faster progress.^58^ Access to quality health care is necessary but far from sufficient for achieving universal health coverage, which also requires provision of care without financial hardship and encompasses services that do not explicitly avert death or fully treat specific health conditions (eg, family planning services, palliative care).^59^, ^60^ Substantial debate exists around the effects of national insurance schemes and government health spending on improving access to high-quality health care and overall universal health coverage. Our exploratory analyses point to positive, albeit heterogeneous, relationships between total and government health spending and national HAQ Index scores. These results highlight the importance of dedicated financing for improving health-care access and quality, but also indicate that increased health financing alone is not adequate. Instead, how well health spending translates into heightened access to quality health care is probably shaped by many factors,^61^ including health system governance,^2^ efficiencies with which financial and health-care resources are dispersed,^62^ and relative distributions of health system inputs across service areas and subnational locations.^63^ Future work should assess the potential effect of improvements across these dimensions on advances in health-care access and quality.

### Future directions for measuring health-care access and quality

With its annual cycle, the GBD study supports ongoing methodological and conceptual improvements for measuring personal health-care access and quality. One priority area, which has been extensively debated, is determining how to best update the amenable cause list, both for fatal and non-fatal outcomes. One approach would entail a systematic review of GBD causes to identify intervention effectiveness by cause and then empirically establish thresholds at which health care significantly improves defined outcomes. Another approach could be to establish key health service areas to be represented by the HAQ Index and then selecting a set of amenable outcomes, fatal and non-fatal, to characterise each health area.^57^ The Nolte and McKee list of causes^6^, ^7^, ^8^, ^9^ includes a range of important areas, but how well performance in these high-priority areas reflects performance in others (eg, vision and hearing, trauma services) is not clear.

Using MIRs for cancers instead of risk-standardised death rates provided an improved indicator of country-level differences in access to effective cancer care. The quantity and quality of cancer-registry data in GBD 2016 supported our use of cancer MIRs, but broader MIR use might be limited by the sparsity of data and methodological demands (eg, reconciling long lag times between disease detection and death from causes like diabetes). Future iterations should consider whether and how to expand the application of MIRs to more GBD causes, particularly those where disease-specific registries or surveillance exist (eg, renal registries). Revisiting age dimensions related to amenable mortality is also warranted, because the current limit of 74 years, as defined by Nolte and McKee,^6^, ^7^, ^8^, ^9^ for most causes might not fully represent the potential of health care to avert death after that age. However, whether age-group bounds should be determined by changes in life expectancy or age-specific improvements in survival, or demarcated by cause-specific advances in reducing mortality by age group is not immediately clear. Relatedly, age-specific HAQ Index analyses might provide a better understanding of how health-care access and quality varies across the lifespan. Such work could shed light on how well health systems are responding to broader demographic shifts and population ageing.^64^, ^65^

Future work also should seek to disentangle the effects of access from quality on HAQ Index performance. We found that the HAQ Index was strongly correlated with total health spending, but it is not clear how more spending on health culminates in improved access (eg, investments in health-care infrastructure, financing national insurance schemes) versus quality (eg, funding training in effective medical care, purchase and maintenance of functional medical supplies). Further, the relative effect of improved access to, as compared with quality of, health care could vary by therapeutic area and the optimal levels of care. For instance, good access to hospitals with skilled medical personnel and functional surgical equipment without corresponding access to high-quality primary care could have more negative ramifications for vaccine-preventable diseases than for conditions mainly addressed by surgery. Strengthening the overall continuum of care,^66^ by and across health areas, also warrants prioritisation, since efforts to better align primary and specialty care could enhance both patient outcomes and systems efficiency.

Going forward, we aim to incorporate improvements in measuring health-care access and quality into more comprehensive assessments of health system performance. Expanding HAQ Index estimation to subnational locations directly supports this endeavour, and ongoing work to quantify human resources for health and financial risk protection within the broader GBD study support the assessment of other health system domains. Quantifying inequalities in health system responsiveness requires additional attention if the World Health Report 2000 framework is to be replicated,^1^ emphasising the need to better parse out the effects of improving quality of care versus access. Additionally, combining the HAQ Index with measures that reflect the effect of interventions on risk factors modifiable by public health programmes (eg, child growth failure) could provide a better assessment of overarching health-system action. Finally, substantial interest exists in translating HAQ Index scores into coverage of populations or number of people with access to quality health services. Multiplying HAQ Index values by population could approximate this (ie, the 0–100 scale approximates 0–100%), and the strong correlation between PCA-derived HAQ Index scores and the arithmetic mean of its component parts (*r*=0·99; appendix p 153) suggests that results might not be overly sensitive to index construction methods.

### Comparison with GBD 2015 assessment of personal health-care access and quality

Compared with GBD 2015,^20^ GBD 2016 HAQ Index scores are slightly higher for high-SDI countries and lower for low-to-middle SDI countries, whereas changes in overall rankings followed less consistent SDI patterns (appendix pp 154–55). Although individual country-level changes might represent several factors (eg, availability of new vital registration data, improved cause-specific modelling), the use of MIRs for cancers, and thus their increased contribution to overall HAQ Index scores, was a main contributor. In GBD 2015, many lower-SDI countries received relatively high scores for cancers,^20^ whereas conditionalising cancer mortality on incidence resulted in a distinct SDI gradient (appendix p 96–111). Subsequently, we view these results as substantially improved since GBD 2015.

### Limitations

Our analysis is subject to limitations beyond those already described. First, any limitations in GBD 2016 cause-of-death estimation are also applicable to this study.^27^ For GBD 2016, we aimed to better account for cause-of-death data quality by developing a metric for well-certified deaths and using this measure to inform GBD data standardisation and correction processes. Nonetheless, establishing and maintaining high-quality vital registration systems is essential to improved cause-of-death estimation. For instance, abrupt or prolonged conflict can lead to cause-of-death data gaps or lags in reporting; subsequently, HAQ Index performance might not yet fully capture the ramifications of conflict on health care in some locations. Second, continued updates to the GBD comparative risk assessment improved risk-standardisation of amenable causes, but we might not account for all possible differences in mortality related to underlying risk exposure. Third, our scaling approach (ie, transforming each cause to a scale of 0–100) does not allow for the potential for additional improvements in reducing cause-specific mortality. How to establish empirically-derived lower bounds for each cause remains unclear, but future work should consider the use of alternative scaling methods. Fourth, the HAQ Index does not expressly capture possible effects of personal health care on causes without substantial mortality. Although performance on these causes might be well correlated with the current HAQ Index formulation, their inclusion could strengthen overall measurement. Fifth, the HAQ Index does not explicitly distinguish between the effects of primary and secondary care,^66^ though some causes might give a stronger signal on certain health-system dimensions (eg, surgical intervention for appendicitis). Improved performance in particular therapeutic areas might represent a combination of advances in primary care (eg, diagnosis and treatment of hypertension) and secondary or referral services (eg, stroke unit, cardiology), or overall gains in continuums of care. Finally, our exploratory analysis of HAQ Index performance did not account for all potential factors related to health-care access and quality; future work should consider how other dimensions of health financing and health care are associated with the HAQ Index (eg, catastrophic health spending, insurance coverage), as well as broader social determinants of health (eg, poverty, accessibility).^67^

## Conclusions

The global ambition towards universal health coverage by 2030 necessitates ensuring that all populations have good access to quality health services. Progress is possible, as shown by accelerated gains on the HAQ Index for many low-SDI countries during the MDG era. However, such advances are not inevitable, as underscored by slowed improvements in several countries and for non-communicable diseases that are best targeted by quality services coordinated across continuums of care. Large geographical inequalities persist across and within countries, highlighting an urgent need for policy attention toward places at risk of being left behind. Current performance represents action from the past, and thus the pace of progress could accelerate for many middle-to-low SDI countries if recent investments can be translated into health-care gains. To strengthen and deliver health systems for the next generation, national and international health agencies alike must focus on improving health-care access and quality across health service areas and reaffirm their commitment to accelerating progress for the world's poorest populations.

Correspondence to: Prof Rafael Lozano, Institute for Health Metrics and Evaluation, University of Washington, Seattle, WA 98121, USA **rlozano@uw.edu**
