# Supplementary material for: Measuring performance on the Healthcare Access and Quality Index for 195 countries and territories and selected subnational locations: a systematic analysis from the Global Burden of Disease Study 2016
Source: Lancet. 2018 Jun 2;391(10136):2236–71. doi: 10.1016/S0140-6736(18)30994-2 (PMC5986687; doi:10.1016/S0140-6736(18)30994-2)
Supplement: Supplementary appendix [file mmc1.pdf]

THE LANCET

Supplementary appendix

This appendix formed part of the original submission and has been peer reviewed. We post it as supplied by the authors.

Supplement to: GBD 2016 Healthcare Access and Quality Collaborators. Measuring performance on the Healthcare Access and Quality Index for 195 countries and territories and selected subnational locations: a systematic analysis from the Global Burden of Disease Study 2016. *Lancet* 2018; published online May 23. http://dx.doi.org/10.1016/S0140-6736(18)30994-2.

Appendix to Measuring performance on the Healthcare Access and Quality Index for 195 countries and territories and selected subnational locations: a systematic analysis from the Global Burden of Disease Study 2016

This appendix provides further methodological detail and supplemental figures for the Healthcare Access and Quality Index. The appendix is organised into broad sections following the structure of the main paper.

Table of Contents

Author contributions……i

Preamble..1

List of Supplementary figures and tables...2

GATHER Statement...5

Part 1. Estimating the Healthcare Access and Quality Index 8

Section 1. Healthcare Access and Quality Index overview

Section 2. Overall modelling strategy for the Healthcare Access and Quality Index

Part 2. Cause write-ups………..14

Part 3. Online tools and glossary of terms 90

Section 1. Online tools

Section 2. List of abbreviations

Section 3. List of ISO3 codes and location names

Part 4. Supplementary results………………………………………………………………………………………………………96

Authors' Contributions

Managing the estimation process

Nancy Fullman, Rafael Lozano, and Christopher Murray.

Writing the first draft of the manuscript

Nancy Fullman, Rafael Lozano, and Christopher Murray.

Providing data or critical feedback on data sources

Cristiana Abbafati, Foad Abd-Allah, Victor Aboyans, Laith Abu-Raddad, Akilew Awoke Adane, Adesoji Adedoyin, Olatunji Adetokunboh, Tara Ballav Adhikari, Mohsen Afarideh, Dominic Agius, Anurag Agrawal, Aliasghar Ahmad Kiadaliri, Miloud Taki Eddine Aichour, Mohammed Akibu, Tomi Akinyemiju, Nadia Akseer, Fares Alahdab, Khurshid Alam, Mohammed Albittar, Kefyalew Addis Alene, Ayman Al-Eyadhy, Peter Allebeck, Ubai Alsharif, Khalid Altirkawi, Azmeraw T Amare, Yaw Ampem Amoako, Nahla Anber, Catalina Liliana Andrei, Valdelaine Etelvina Araújo, Olatunde Aremu, Al Artaman, Krishna Kumar Aryal, Hamid Asayesh, Ephrem Tsegay Asfaw, Solomon Weldegebreal Asgedom, Sachin Atre, Ashish Awasthi, Beatriz Paulina Ayala Quintanilla, Henok Tadesse Ayele, Rakesh Ayer, Tambe Betrand Ayuk, Alaa Badawi, Maciej Banach, Amitava Banerjee, Amrit Banstola, Miguel Barboza, Till Bärnighausen, Simón Barquera, Lope Barrero, Sanjay Basu, Neeraj Bedi, Bayu Begashaw Bekele, Saba Abraham Belay, Yihalem Abebe Belay, Aminu Bello, Isabela Bensenor, Derbew Fikdau Berhe, Eduardo Bernabe, Robert Bernstein, Mircea Beuran, Belete Biadgo, Ali Bijani, Boris Bikbov, Charles Birungi, Ibrahim Bou Orm, Gabrielle Britton, Traolach Brugha, Julio Cesar Campuzano R, Mate Car, Josip Car, Juan J Carrero, Carlos Castañeda-Orjuela, Ferrán Catalá-López, Jung-Chen Chang, Hsing-Yi Chang, Peggy Pei-Chia Chiang, Jee-Young Choi, Devasahayam Christopher, Sheng-Chia Chung, Massimo Cirillo, Cyrus Cooper, Paolo Angelo Cortesi, Monica Cortinovis, Michael Criqui, Elizabeth Cromwell, John Crump, Lalit Dandona, Rakhi Dandona, Ahmad Daryani, Maryam Daryani, Siddharth K Das, José Das Neves, Gebre Demoz, Kebede Deribe, Don Des Jarlais, Subhojit Dey, Samath Dharmaratne, Meghnath Dhimal, David Teye Doku, Ray Dorsey, Kerrie Doyle, Manisha Dubey, Michelle Echko, Joshua R. Ehrlich, Maysaa El Sayed Zaki, Ziad El-Khatib, Aman Endries, Sharareh Eskandarieh, Alireza Esteghamati, Tamer H Farag, Emerito Jose A Faraon, Andre Faro, Farshad Farzadfar, Valery Feigin, Netsanet Fentahun, Seyed-Mohammad Fereshtehnejad, Joao Fernandes, Florian Fischer, Abraham Flaxman, Joseph J Frostad, Nancy Fullman, Neal Futran, Emmanuela Gakidou, Ketevan Gambashidze, Abadi Kahsu Gebre, Amanuel Tesfay Gebremedhin, Bereket Gebremichael, Johanna Geleijnse, Melkamu Dedefo Gishu, Srinivas Goli, Hector Gomez-Dantes, Philimon Gona, Ayman Grada, Max Griswold, Harish Gugnani, Tanush Gupta, Tarun Gupta, Rahul Gupta, Murthy Gvs, Juanita Haagsma, Nima Hafezi-Nejad, Gessessew Bugssa Hailu, Hilda Harb, Sivadasanpillai Harikrishnan, Josep Maria Haro, Hamid Y Hassen, Rasmus Havmoeller, Behnam Heidari, Mohsen Heidari, Delia Hendrie, Long Hoang Nguyen, Hans Hoek, Nobuyuki Horita, Damian Hoy, Mohamed Hsairi, Aung Soe Htet, Guoqing Hu, Kim Moesgaard Iburg, Fachmi Idris, Ehimario Igumbor, Chad Ikeda, Bogdan Vasile Ileanu, Kaire Innos, Sheikh Mohammed Shariful Islam, Farhad Islami, Troy Jacobs, Kathryn H. Jacobsen, Nader Jahanmehr, Mihajlo Jakovljevic, Amr Jamal, Mehdi Javanbakht, Achala Jayatilleke, Panniyammakal Jeemon, Vivekanand Jha, Oommen John, Sarah Johnson, Jost B. Jonas, Vasna Joshua, Jacek Jóźwiak, Mikk Jürisson, Zubair Kabir, Rajendra Kadel, Molla Kahssay, Rizwan Kalani, Chittaranjan Kar, André Karch, Corine Karema, Seyed M Karimi, Amir Kasaeian, Getachew Mullu Kassa, Nicholas J Kassebaum, S Vittal Katikireddi, Norito Kawakami, Konstantin Kazanjan, Seifu Kebede, Peter Keiyoro, Andre P Kengne, Maia Kereselidze, Yousef Khader, Morteza Abdullatif Khafaie, Morteza

Khafaie, Ibrahim Khalil, Md Nuruzzaman Khan, Muhammad Ali Khan, Young-Ho Khang, Abdullah Khoja, Ardeshir Khosravi, Jagdish Khubchandani, Yun Jin Kim, Ruth Kimokoti, Adnan Kisa, Sonali Kochhar, Soewarta Kosen, Michael Kravchenko, Kewal Krishan, Kristopher J Krohn, Barthelemy Kuate Defo, Pushpendra Kumar, G Anil Kumar, Michael Kutz, Ihor Kuzin, Deepesh Lad, Dharmesh Lal, Hilton Lam, Qing Lan, Van Charles Lansingh, Anders Larsson, Janet Leasher, Yirga Legesse, Misgan Legesse Liben, James Leigh, Samson Leta, Ricky Leung, Juan Liang, Lee-Ling Lim, Stephen S Lim, Shiwei Liu, Alan Lopez, Stefan Lorkowski, Paulo Lotufo, Ronan Lyons, Hassan Magdy Abd El Razek, Mohammed Magdy Abd El Razek, Marek Majdan, Azeem Majeed, Reza Malekzadeh, Rajesh Malhotra, Abdullah Mamun, Mohammad Ali Mansournia, Lorenzo Mantovani, Tsegahun Manyazewal, Ira Martopullo, Winfried März, Manu Mathur, Mohsen Mazidi, Colm Mcalinden, John Mcgrath, Suresh Mehata, Varshil Mehta, Toni Meier, Kidanu Meles, Mulugeta Melku, Ziad Memish, Walter Mendoza, Desalegn Tadese Mengistu, Zerihun Menlkalew, Atte Meretoja, Tuomo Meretoja, Haftay Berhane Mezgebe, Tomasz Miazgowski, Eirini Micha, Robert Milam, Anoushka Millear, Ted Miller, Andreea Mirica, Erkin Mirrakhimov, Awoke Misganaw, Mengistu Mitiku, Karzan Mohammad, Roghayeh Mohammadibakhsh, Shafiu Mohammed, Ali H Mokdad, Mariam Molokhia, Lorenzo Monasta, Maziar Moradi-Lakeh, Ilais Moreno Velasquez, Rintaro Mori, Mark W. Moses, Ulrich Mueller, Manoj Murhekar, Srinivas Murthy, Kamarul Imran Musa, Ghulam Mustafa, Mohsen Naghavi, Seyed Sina Naghibi Irvani, Luigi Naldi, K.M. Venkat Narayan, Bruno Nascimento, Ionut Negoi, Ruxandra Irina Negoi, Charles Newton, Josephine Ngunjiri, Bo Norrving, Jean Jacques Noubiap, Carla Obermeyer, Felix Ogbo, Olanrewaju Oladimeji, Andrew T. Olagunju, Tinuke O Olagunju, Helen Olsen, Bolajoko Olusanya, Jacob Olusanya, Kanyin Ong, John Nelson Opio, Alberto Ortiz, Mahesh P A, Adrian Pana, Songhomitra Panda-Jonas, Jeyaraj Pandian, Sanghamitra Pati, Snehal Patil, Ajay Patle, George Patton, Vishnupriya Rao Paturi, Marcel Pedroso, Sandra P Pedroza, David Pereira, Alexandre Pereira, Norberto Perico, Max Petzold, Niloofar Peykari, Frédéric Piel, David M Pigott, Michael Piradov, Suzanne Polinder, V Prakash, Swayam Prakash, Narayan Prasad, Noela Prasad, Mostafa Qorbani, Amir Radfar, Anwar Rafay, Alireza Rafiei, Afarin Rahimi-Movaghar, Vafa Rahimi-Movaghar, Mahfuzar Rahman, Sajjad Rahman, Rajesh Kumar Rai, Saleem Rana, Salman Rawaf, Giuseppe Remuzzi, Andre Renzaho, Mohammad Sadegh Rezai, Antonio Luiz Ribeiro, Juan A Rivera, Leonardo Roever, Luca Ronfani, Gholamreza Roshandel, Ali Rostami, Dietrich Rothenbacher, Ambuj Roy, Nobhojit Roy, Perminder Sachdev, Nafis Sadat, Saeid Safiri, Rajesh Sagar, Mohammad Ali Sahraian, Joseph Salama, Payman Salamati, Hamideh Salimzadeh, Abdallah M. Samy, Parag Sancheti, Damian Santomauro, Itamar Santos, Milena Santric Milicevic, Abdur Sarker, Nizal Sarrafzadegan, Benn Sartorius, Maheswar Satpathy, Monika Sawhney, Mete Saylan, Elke Schaeffner, Josef Schmidhuber, David C Schwebel, Falk Schwendicke, Mario Šekerija, Sadaf Sepanlou, Edson Serván-Mori, Azadeh Shafieesabet, Masood Ali Shaikh, Marina Shakh-Nazarova, Meenakshi Sharma, Aziz Sheikh, Peilin Shi, Girma Temam Shifa, Mark Shrime, Soraya Siabani, Inga Sigfusdottir, Donald Silberberg, Diego Augusto Santos Silva, Jasvinder Singh, Narinder Pal Singh, Virendra Singh, Dhirendra Narain Sinha, Vegard Skirbekk, Karen Sliwa, Adauto Martins Soares Filho, Melek Somai, Samir Soneji, Reed Sorensen, Luisa Sorio Flor, Chandrashekhar T Sreeramareddy, Vasiliki Stathopoulou, Mark Stokes, Lela Sturua, Mu'awiyyah Babale Sufiyan, Rizwan Suliankatchi Abdulkader, Patrick Sur, Bryan L Sykes, Bryan L Sykes, Rafael Tabarés-Seisdedos, Nuno Taveira, Mohamad-Hani Temsah, Abdullah Terkawi, Belay Tessema, Nihal Thomas, Ruoyan Tobe-Gai, Marcello Tonelli, Miguel Tortajada-Girbés, Marcos Roberto Tovani-Palone, Bach Tran, Khanh Bao Tran, Srikanth Tripathy, Christopher Troeger, Thomas Truelsen, Derrick Tsoi, Kald Beshir Tuem, Kingsley Nnanna Ukwaja, Irfan Ullah, Olalekan Uthman, Pascual Valdez, Job Fm Van Boven, Tommi Vasankari, Patrcia Pereira Vasconcelos De Oliveira, Sergey Vladimirov, Vasiliy Vlassov, Stein Emil Vollset, Yasir

Waheed, Yuan-Pang Wang, Yanping Wang, Marcia R Weaver, Elisabete Weiderpass, Robert Weintraub, Jordan Weiss, Andrea Werdecker, Ronny Westerman, Harvey Whiteford, Justyna Widecka, Andrea Winkler, Charles Shey Wiysonge, Abdulhalik Workicho, Grant Wyper, Denis Xavier, Gelin Xu, Lijing Yan, Ebrahim M. Yimer, Peng Yin, Biruck Desalegn Yirsaw, Naohiro Yonemoto, Gerald Yonga, Seok-Jun Yoon, Marcel Yotebieng, Mustafa Younis, Vesna Zadnik, Zoubida Zaidi, Sojib Bin Zaman, Mohammad Zamani, Jun Zhu, Sanjay Zodpey, and Liesl Zuhlk.

Developing methods or computational machinery

Cristiana Abbafati, Olatunji Adetokunboh, Ashkan Afshin, Syed Danish Ali, Christine Allen, Nahla Anber, Ryan M Barber, Till Bärnighausen, Neeraj Bedi, James Bennett, Stan Biryukov, Ian Bolliger, Kelly Cercy, Pankaj Chaturvedi, Jee-Young Choi, Cyrus Cooper, Elizabeth Cromwell, Ahmad Daryani, Maryam Daryani, Alireza Esteghamati, Farshad Farzadfar, Christina Fitzmaurice, Abraham Flaxman, Kyle J Foreman, Joseph J Frostad, Emmanuela Gakidou, Max Griswold, Víctor Hugo Herrera Ballesteros, Enayatollah Homaie Rad, Nicholas J Kassebaum, Adnan Kisa, Qing Lan, Misgan Legesse Liben, Stephen S Lim, Patrick Y Liu, Tim Lucas, Mohsen Mazidi, Martin McKee, Shawn Minnig, Awoke Misganaw, Roghayeh Mohammadibakhsh, Ali H Mokdad, Mark W Moses, Mohsen Naghavi, Emma Nichols, Ellen Nolte, Carla Obermeyer, Helen E Olsen, Hannah Peterson, David M Pigott, Robert Reiner, Gregory Roth, Abdallah M. Samy, Damian Santomauro, Maheswar Satpathy, Austin Schumacher, Reed Sorensen, Vinay Srinivasan, Patrick Sur, Bryan L Sykes, Christopher Troeger, Irfan Ullah, Rachel L Updike, Theo Vos, Ronny Westerman, Harvey Whiteford, Jamal Yearwood, Ebrahim M. Yimer, And Seok-Jun Yoon.

Applying analytical methods to produce estimates

Khurshid Alam, Christine Allen, Azmeraw T Amare, Nahla Anber, Olatunde Aremu, Solomon Weldegebreal Asgedom, Ashish Awasthi, Tambe Betrand Ayuk, Ryan M Barber, Bayu Begashaw Bekele, James Bennett, Charles Birungi, Stan Biryukov, Ian Bolliger, Jacqueline Castillo Rivas, Kelly Cercy, Peggy Pei-Chia Chiang, Devasahayam Christopher, Danny Colombara, Cyrus Cooper, Elizabeth Cromwell, Ahmad Daryani, Maryam Daryani, Gebre Demoz, Aman Endries, Alireza Esteghamati, Christina Fitzmaurice, Abraham Flaxman, Joseph J Frostad, Emmanuela Gakidou, Bereket Gebremichael, William Godwin, Max Griswold, Hamid Y Hassen, Jiawei He, Nathaniel Henry, Enayatollah Homaie Rad, Chad Ikeda, Caleb Irvine, Mikk Jürisson, Amir Kasaeian, Nicholas J Kassebaum, Yun Jin Kim, Adnan Kisa, Pushpendra Kumar, Hmwe Kyu, Qing Lan, Misgan Legesse Liben, Janni Leung, Stephen S Lim, Margaret Lind, Patrick Y Liu, Tim Lucas, Emilie Maddison, Hassan Magdy Abd El Razek, Helena Manguerra, Ira Martopullo, Mohsen Mazidi, Anoushka Millear, Shawn Minnig, Awoke Misganaw, Roghayeh Mohammadibakhsh, Shafiu Mohammed, Ali H Mokdad, Mark W Moses, Mohsen Naghavi, Grant Nguyen, Emma Nichols, Carla Obermeyer, Helen E Olsen, Bolajoko Olusanya, kanyin ong, Caroline Purcell, Mostafa Qorbani, Alireza Rafiei, Saleem Rana, Zane Rankin, Marissa Reitsma, Gregory Roth, Abdallah M. Samy, Damian Santomauro, Maheswar Satpathy, Josef Schmidhuber, Peilin Shi, Jasvinder Singh, Alison Smith, Reed Sorensen, Luisa Sorio Flor, Vinay Srinivasan, Jeffrey Stanaway, Patrick Sur, Bach Tran, Christopher Troeger, Kingsley Nnanna Ukwaja, Irfan Ullah, Rachel L.Updike, Patrícia Pereira Vasconcelos de Oliveira, Ronny Westerman, Harvey Whiteford, Jamal Yearwood, Ebrahim M. Yimer, Paul Siu Fai Yip, Biruck Desalegn Yirsaw, Seok-Jun Yoon, and Zoubida Zaidi.

Providing critical feedback on methods or results

Degu Abate, Solomon Mequanente Abay, Cristiana Abbafati, Foad Abd-Allah, Jemal Abdela, Ahmed Abdelalim, Zegeye Abebe, Teshome Abuka Abebo, Victor Aboyans, Haftom Abraha, Daisy M X Abreu,

Laith Abu-Raddad, Olatunji Adetokunboh, Tara Ballav Adhikari, Mohsen Afarideh, Gina Agarwal, Anurag Agrawal, Aliasghar Ahmad Kiadaliri, Miloud Taki Eddine Aichour, Mohammed Akibu, Rufus Akinyemi, Tomi Akinyemiju, Nadia Akseer, Deena Al Asfoor, Fares Alahdab, Ziyad Al-Aly, Khurshid Alam, Tahiya Alam, Mohammed Albittar, Animut Alebel, Kefyalew Addis Alene, Mehran Alijanzadeh, Syed Aljunid, Ala'a Alkerwi, Franois Alla, Peter Allebeck, Christine Allen, Mahmoud Alomari, Rajaa Al-Raddadi, Ubai Alsharif, Khalid Altirkawi, Nelson Alvis-Guzman, Azmeraw T Amare, Kebede Amenu, Walid Ammar, Yaw Ampem Amoako, Nahla Anber, Catalina Andrei, Catalina Liliana Andrei, Sofia Androudi, Carl Abelardo Antonio, Valdelaine Etelvina Araújo, Olatunde Aremu, Johan rnlv, Al Artaman, Krishna Kumar Aryal, Hamid Asayesh, Ephrem Tsegay Asfaw, Solomon Weldegebreal Asgedom, Rana Jawad Asghar, Tesfay Mehari Atey, Sachin Atre, Madhu Sudhan Atteraya, Leticia Avila-Burgos, Euripide Avokpaho, Ashish Awasthi, Beatriz Paulina Ayala Quintanilla, Henok Tadesse Ayele, Yohanes Ayele, Tambe Betrand Ayuk, Peter Azzopardi, Natasha Azzopardi-Muscat, Tesleem Babalola, Alaa Badawi, Maciej Banach, Amitava Banerjee, Amrit Banstola, Miguel A. Barboza, Suzanne Barker-Collo, Till Bärnighausen, Simón Barquera, Lope Barrero, Quique Bassat, Sanjay Basu, Bernhard T Baune, Neeraj Bedi, Masoud Behzadifar, Meysam Behzadifar, Abate Bekele, Bayu Begashaw Bekele, Ezra Belay, Saba Abraham Belay, Yihalem Abebe Belay, Michelle Bell, Aminu K Bello, Kadine Priscila Bender Dos Santos, Derrick Bennett, Isabela Bensenor, Derbew Fikadu Berhe, Eduardo Bernabe, Robert Bernstein, Mircea Beuran, Ashish Bhalla, Paurvi Bhatt, Soumyadeep Bhaumik, Sree Bhushan Raju, Zulfiqar Bhutta, Belete Biadgo, Ali Bijani, Boris Bikbov, Nigus Bililign, Charles Birungi, Stan Biryukov, Ian Bolliger, Kayvan Bozorgmehr, Oliver Brady, Alexandra Brazinova, Nicholas Breitborde, Hermann Brenner, Gabrielle Britton, Traolach Brugha, Zahid Butt, Alessandra C Goulart, Lucero Cahuana-Hurtado, Ismael Campos-Nonato, Josip Car, Mate Car, Rosario Cárdenas, Juan J Carrero, Félix Carvalho, Deborah Carvalho Malta, Carlos Castañeda-Orjuela, Jacqueline Castillo Rivas, Ferrán Catalá-López, Jung-Chen Chang, Aparajita Chattopadhyay, Pankaj Chaturvedi, Peggy Pei-Chia Chiang, Vesper Chisumpa, Jee-Young Choi, Hanne Christensen, Devasahayam Christopher, Sheng-Chia Chung, Liliana G Ciobanu, Sara Conti, Cyrus Cooper, Paolo Angelo Cortesi, Monica Cortinovis, Ewerton Cousin, Michael Criqui, Elizabeth Cromwell, Christopher Crowe, John Crump, Berihun Dachew, Abel Dadi, Lalit Dandona, Rakhi Dandona, Paul Dargan, Ahmad Daryani, Maryam Daryani, Jai Kumar Das, José Das Neves, Kairat Davletov, Barbora De Courten, Diego De Leo, Jan-Walter De Neve, Robert Dellavalle, Gebre Demoz, Kebede Deribe, Subhojit Dey, Samath Dharmaratne, Meghnath Dhimal, Shirin Djalalinia, David Teye Doku, Ray Dorsey, Kerrie Doyle, Tim Driscoll, Manisha Dubey, Eleonora Dubljanin, Bruce Duncan, Dumessa Edessa, David Edvardsson, Joshua R. Ehrlich, Maysaa El Sayed Zaki, Ziad El-Khatib, Matthias Endres, Aman Endries, Babak Eshrati, Sharareh Eskandarieh, Alireza Esteghamati, Tamer H Farag, Mahbobeh Faramarzi, Emerito Jose A Faraon, Andre Faro, Farshad Farzadfar, Mir Sohail Fazeli, Valery Feigin, Andrea Feigl, Netsanet Fentahun, Seyed-Mohammad Fereshtehnejad, Eduarda Fernandes, Joao Fernandes, Garumma Feyissa, Irina Filip, Florian Fischer, Christina Fitzmaurice, Abraham Flaxman, Nataliya Foigt, Kyle J Foreman, Joseph J Frostad, Nancy Fullman, Thomas Fürst, Neal Futran, Emmanuela Gakidou, Silvano Gallus, Ketevan Gambashidze, Amiran Gamkrelidze, Morsaleh Ganji, Abadi Kahsu Gebre, Tsegaye Tewelde Gebrehiwot, Amanuel Tesfay Gebremedhin, Bereket Gebremichael, Yalemzewod Assefa Gelaw, Johanna Geleijnse, Demeke Geremew, Peter W Gething, Reza Ghadimi, Khalil Ghasemi Falavarjani, Maryam Ghasemi-Kasman, Kidu Gidey, Paramjit Gill, Ababi Giref, Maurice Giroud, Melkamu Dedefo Gishu, Giorgia Giussani, Srinivas Goli, Hector Gomez-Dantes, Philimon Gona, Amador Goodridge, Sameer V Gopalani, Yevgeniy Goryakin, Ayman Grada, Max Griswold, Giuseppe Grosso, Harish Gugnani, Yuming Guo, Rahul Gupta, Rajeev Gupta, Tanush Gupta, Tarun Gupta, Juanita Haagsma, Vladimir Hachinski, Nima Hafezi-Nejad, Gessessew

Bugssa Hailu, Randah Hamadeh, Samer Hamidi, Graeme Hankey, Hilda Harb, Heather Harewiood, Sivadasanpillai Harikrishnan, Josep Maria Haro, Badr Hasan, Hamid Y. Hassen, Rasmus Havmoeller, Caitlin Hawley, Simon Hay, Stephen Hearps, Mohamed Hegazy, Behzad Heibati, Behnam Heidari, Mohsen Heidari, Delia Hendrie, Andualem Henok, Vctor Hugo Herrera Ballesteros, Claudiu Herteliu, Desalegn Hibstu, Long Hoang Nguyen, Nobuyuki Horita, H Dean Hosgood, Seyedmostafa Hosseini, Mihaela Hostiuc, Sorin Hostiuc, Damian Hoy, Guoqing Hu, John Huang, Trang Huyen Nguyen, Ehimario Igumbor, Bogdan Vasile Ileanu, Olayinka Ilesanmi, Sheikh Mohammed Shariful Islam, Farhad Islami, Troy Jacobs, Kathryn H. Jacobsen, Nader Jahanmehr, Rajesh Jain, Sudhir Kumar Jain, Mihajlo Jakovljevic, Amr Jamal, Mehdi Javanbakht, Achala Jayatilleke, Panniyammakal Jeemon, Vivekanand Jha, Oommen John, Jost B. Jonas, Jacek Jóźwiak, Mikk Jürisson, Alemneh Kabeta, Zubair Kabir, Rajendra Kadel, Amaha Kahsay, Molla Kahssay, Rizwan Kalani, Marina Karanikolos, André Karch, Seyed M Karimi, Amir Kasaeian, Dessalegn Haile Kassa, Getachew Mullu Kassa, Tesfaye Kassa, Nicholas J Kassebaum, S Vittal Katikireddi, Anil Kaul, Norito Kawakami, Konstantin Kazanjan, Seifu Kebede, Peter Keiyoro, Andre P Kengne, Maia Kereselidze, Yousef Khader, Ibrahim Khalil, Ejaz Khan, Gulfaraz Khan, Md Nuruzzaman Khan, Muhammad Ali Khan, Young-Ho Khang, Mona Khater, Abdullah Khoja, Ardeshir Khosravi, Jagdish Khubchandani, Getiye Kibret, Daniel Kiirithio, Daniel Kim, Yun Jin Kim, Ruth Kimokoti, Yohannes Kinfu, Sanjay Kinra, Adnan Kisa, Sonali Kochhar, Yoshihiro Kokubo, Jacek Kopec, Parvaiz Koul, Ai Koyanagi, Kewal Krishan, Kristopher J Krohn, Barthelemy Kuate Defo, G Anil Kumar, Pushpendra Kumar, Deepesh Lad, Alessandra Lafranconi, Dharmesh Lal, Ratilal Lalloo, Hilton Lam, Faris Lami, Qing Lan, Justin J Lang, Van Charles Lansingh, Sonia Lansky, Anders Larsson, Arman Latifi, Jeffrey Lazarus, Janet Leasher, Paul Lee, Yirga Legesse, Misgan Legesse Liben, James Leigh, Cheru T Leshargie, Samson Leta, Janni Leung, Ricky Leung, Miriam Levi, Yongmei Li, Lee-Ling Lim, Stephen S Lim, Shai Linn, Stefan Listl, Shiwei Liu, Rakesh Lodha, Alan Lopez, Scott Lorch, Stefan Lorkowski, Paulo Lotufo, Rafael Lozano, Raimundas Lunevicius, Grégoire Lurton, Ronan Lyons, Fadi Maalouf, Erlyn Macarayan, Erlyn Rachelle Macarayan, Mark Mackay, Fabiana Madotto, Hassan Magdy Abd El Razek, Mohammed Magdy Abd El Razek, Marek Majdan, Reza Majdzadeh, Azeem Majeed, Reza Malekzadeh, Abdullah Mamun, Mohammad Ali Mansournia, Lorenzo Mantovani, Tsegahun Manyazewal, Chabila Mapoma, Jose Martinez-Raga, Sheila Martins, Francisco RogerlndioMartins-Melo, Winfried März, Benjamin Massenburg, Manu Mathur, Pallab K Maulik, Mohsen Mazidi, Colm Mcalinden, John Mcgrath, Martin Mckee, Suresh Mehata, Ravi Mehrotra, Kala Mehta, Varshil Mehta, Toni Meier, Fabiola Mejia-Rodriguez, Kidanu Meles, Mulugeta Melku, Peter Memiah, Ziad Memish, Walter Mendoza, Desalegn Tadese Mengistu, Zerihun Menlkalew, Atte Meretoja, Tuomo Meretoja, Haftay Berhane Mezgebe, Tomasz Miazgowski, Eirini Micha, Ted Miller, Vuong Minh Nong, Gk Mini, Shawn Minnig, Andreea Mirica, Erkin Mirrakhimov, Awoke Misganaw, Mengistu Mitiku, Babak Moazen, Karzan Mohammad, Roghayeh Mohammadibakhsh, Mohammed Mohammed, Shafiu Mohammed, Ali H Mokdad, Mariam Molokhia, Fatemeh Momeniha, Lorenzo Monasta, Julio Montañez, Mahmood Moosazadeh, Maziar Moradi-Lakeh, Paula Moraga, Lidia Morawska, Rintaro Mori, Shane Morrison, Seyyed Meysam Mousavi, Ulrich Mueller, Srinivas Murthy, Jonah Musa, Kamarul Imran Musa, Ghulam Mustafa, Chie Nagata, Seyed Sina Naghibi Irvani, Aliya Naheed, Gurudatta Naik, Nitish Naik, Farid Najafi, Vinay Nangia, Jobert Richie Nansseu, K.M. Venkat Narayan, Bruno Nascimento, Ionut Negoi, Ruxandra Irina Negoi, Charles Newton, Josephine Ngunjiri, Dina Nur Anggraini Ningrum, Ellen Nolte, Ole F Norheim, Bo Norrving, Jean Jacques Noubiap, Alypio Nyandwi, Carla Obermeyer, Richard Ofori-Asenso, Felix Ogbo, In-Hwan Oh, Olanrewaju Oladimeji, Andrew T Olagunju, Tinuke O Olagunju, Pedro Olivares, Helen E Olsen, Bolajoko Olusanya, Jacob Olusanya, Kanyin Ong, John Nelson Opio, Eyal Oren, Doris Ortega-Altamirano, Alberto Ortiz, Raziye Özdemir, Mahesh P A, Adrian Pana, Songhomitra Panda-

Jonas, Eun-Kee Park, Hadi Parsian, Tejas Patel, Sanghamitra Pati, Snehal Patil, Ajay Patle, Deepak Paudel, Marcel Pedroso, Alexandre Pereira, David Pereira, Norberto Perico, Max Petzold, Michael Phillips, Michael R. Phillips, Frédéric Piel, David M Pigott, Julian Pillay, Suzanne Polinder, Constance Dimity Pond, Maarten Postma, Farshad Pourmalek, Swayam Prakash, V Prakash, Ravi Prakash Jha, Narayan Prasad, Noela Prasad, Mostafa Qorbani, Amir Radfar, Anwar Rafay, Alireza Rafiei, Kazem Rahimi, Afarin Rahimi-Movaghar, Vafa Rahimi-Movaghar, Mahfuzar Rahman, Muhammad Aziz Rahman, Rajesh Kumar Rai, Usha Ram, Saleem Rana, David Rawaf, Salman Rawaf, Sarah E Ray, Christian Aspacia Razo Garcia, Sasiragha Priscilla Reddy, Cesar Reis, Cesar Reis, Marissa Reitsma, Giuseppe Remuzzi, Andre Renzaho, Serge Resnikoff, Satar Rezaei, Antonio Luiz Ribeiro, Maria Jesus Rios-Blancas, Juan A Rivera, Leonardo Roever, Luca Ronfani, Gholamreza Roshandel, Ali Rostami, Gregory Roth, Dietrich Rothenbacher, Nobhojit Roy, George Ruhago, Yogesh Sabde, Perminder Sachdev, Mahdi Safdarian, Saeid Safiri, Rajesh Sagar, Amirhossein Sahebkar, Mohammad Ali Sahraian, Haniye Sadat Sajadi, Raphael Saldanha, Hamideh Salimzadeh, Joshua A Salomon, Abdallah M. Samy, Juan Sanabria, Parag Sancheti, Maria D Sanchez-Niño, Damian Santomauro, Itamar Santos, Milena Santric Milicevic, Muthupandian Saravanan, Abdur Sarker, Benn Sartorius, Maheswar Satpathy, Miloje Savic, Monika Sawhney, Sonia Saxena, Mete Saylan, Elke Schaeffner, Josef Schmidhuber, Maria Ins Schmidt, Ione Schneider, David C Schwebel, Falk Schwendicke, Sadaf Sepanlou, Edson Serván-Mori, Azadeh Shafieesabet, Masood Ali Shaikh, Mehran Shams-Beyranvand, Heidar Sharafi, Mahdi Sharif-Alhoseini, Rajesh Sharma, Aziz Sheikh, Chloe Shields, Girma Temam Shifa, Mika Shigematsu, Yukito Shinohara, Rahman Shiri, Reza Shirkoohi, Ivy Shiue, Mark Shrime, Sharvari Shukla, Soraya Siabani, Inga Sigfusdottir, Donald Silberberg, Diego Augusto Santos Silva, Joo Pedro Silva, Dayane Gabriele Alves Silveira, Jasvinder Singh, Narinder Pal Singh, Dhirendra Narain Sinha, Mekonnen Sisay, Adauto Martins Soares Filho, Melek Somai, Moslem Soofi, Reed Sorensen, Joan B Soriano, Luisa Sorio Flor, Ireneous Soyiri, Luciano A Sposato, Chandrashekhar T Sreeramareddy, Nicholas Steel, Dan Stein, Mark Stokes, Lela Sturua, Mu'awiyyah Babale Sufiyan, Bruno Sunguya, Bryan L Sykes, Pn Sylaja, Rafael Tabarés-Seisdedos, Santosh Tadakamadla, Getachew Tafrere, Nikhil Tandon, Amare Tariku, Nuno Taveira, Arash Tehrani-Banihashemi, Azeb Tesema, Dawit Tesfaye, Belay Tessema, Mebrahtu Teweldemedhin, Nihal Thomas, Matthew Thompson, Taavi Tillmann, Quyen G. To, Ruoyan Tobe-Gai, Moti Tolera, Marcello Tonelli, Roman Topor-Madry, Fotis Topouzis, Miguel Tortajada-Girbés, Marcos Roberto Tovani-Palone, Bach Tran, Khanh Bao Tran, Avnish Tripathi, Thomas Truelsen, Lorainne Tudor Car, Stefanos Tyrovolas, Uchenna Uchendu, Kingsley Nnanna Ukwaja, Irfan Ullah, Olalekan Uthman, Pascual Valdez, Job Fm Van Boven, Santosh Varughese, Tommi Vasankari, Patrcia Pereira Vasconcelos De Oliveira, Francesco S Violante, Vasiliy Vlassov, Stein Emil Vollset, Theo Vos, Fasil Wagnew, Yasir Waheed, Mitchell Wallin, Judd Walson, Yafeng Wang, Yuan-Pang Wang, Molla Wassie, Elisabete Weiderpass, Robert Weintraub, Jordan Weiss, Fitsum Weldegebreal, Andrea Werdecker, T Eoin West, Richard White, Harvey Whiteford, Justyna Widecka, Charles Shey Wiysonge, Abdulhalik Workicho, Grant Wyper, Denis Xavier, Gelin Xu, Lijing Yan, Yuichiro Yano, Mehdi Yaseri, Jamal Yearwood, Ebrahim M. Yimer, Paul Siu Fai Yip, Biruck Desalegn Yirsaw, Naohiro Yonemoto, Gerald Yonga, Seok-Jun Yoon, Marcel Yotebieng, Mustafa Younis, Chuanhua Yu, Zoubida Zaidi, Sojib Bin Zaman, and Liesl Zuhlke.

Drafting the work or revising is critically for important intellectual content

Olatunji Adetokunboh, Mohsen Afarideh, Sutapa Agrawal, Tomi Akinyemiju, Khurshid Alam, Mahmoud Alomari, Nelson Alvis-Guzman, Nahla Anber, Ashish Awasthi, Peter Azzopardi, Amrit Banstola, Yihalem Abebe Belay, Isabela Bensenor, Hailemichael Bizuneh, Devasahayam Christopher, Cyrus Cooper, Christopher Crowe, Berihun Dachew, Ahmad Daryani, Maryam Daryani, Nicole Davis Weaver, Jan-Walter De Neve, Samath Dharmaratne, Kate Dolan, Ray Dorsey, Kerrie Doyle, Manisha Dubey, Maysaa El Sayed

Zaki, Alireza Esteghamati, Andre Faro, Eduarda Fernandes, Joao Fernandes, Nataliya Foigt, Nancy Fullman, Morsaleh Ganji, Maryam Ghasemi-Kasman, Philimon Gona, Amador Goodridge, Rajeev Gupta, Nima Hafezi-Nejad, Hamid Y. Hassen, Stephen Hearps, Behnam Heidari, Enayatollah Homaie Rad, Nobuyuki Horita, Jacek Jóźwiak, Mikk Jürisson, Getachew Mullu Kassa, Nicholas J Kassebaum, Grant Kemp, Yousef Khader, Alireza Khajavi, Md Nuruzzaman Khan, Mona Khater, Jagdish Khubchandani, Yun Jin Kim, Adnan Kisa, Niranjan Kissoon, Parvaiz Koul, Ai Koyanagi, Kristopher Krohn, Ratilal Lalloo, Alan Lopez, Rafael Lozano, Hassan Magdy Abd El Razek, Mohammed Magdy Abd El Razek, Azeem Majeed, Jose Martinez-Raga, Francisco RogerIndio Martins-Melo, Benjamin Massenburg, Varshil Mehta, Kidanu Meles, George Mensah, Tuomo Meretoja, Haftay Berhane Mezgebe, Roghayeh Mohammadibakhsh, Ali H Mokdad, Shane Morrison, Srinivas Murthy, Kamarul Imran Musa, Ghulam Mustafa, Bruno Nascimento, Grant Nguyen, Andrew T Olagunju, Tinuke O Olagunju, Eyal Oren, Doris Ortega-Altamirano, Hadi Parsian, Michael Phillips, Michael R Phillips, David M Pigott, V Prakash, Ravi Prakash Jha, Hedley Quintana, Anwar Rafay, Alireza Rafiei, Vafa Rahimi-Movaghar, David Rawaf, Salman Rawaf, Perminder Sachdev, Juan Sanabria, Parag Sancheti, Milena Santric Milicevic, Abdur Sarker, Maheswar Satpathy, Monika Sawhney, Falk Schwendicke, Diego Augusto Santos Silva, Dhirendra Narain Sinha, Karen Sliwa, Nuno Taveira, Nihal Thomas, Marcos Roberto Tovani-Palone, Srikanth Tripathy, Olalekan Uthman, Benjamin Uzochukwu, Job Fm Van Boven, Yuan-Pang Wang, Elisabete Weiderpass, Robert Weintraub, T Eoin West, Richard White, Charles Wolfe, Ebrahim M Yimer, Seok-Jun Yoon, and Liesl Zuhlke.

Extracting, cleaning, or cataloging data; designing or coding figures and tables

Haftom Abraha, Olatunji Adetokunboh, Christine Allen, Nahla Anber, Solomon Weldegebreal Asgedom, Ian Bolliger, Julian Chalek, Cyrus Cooper, Leslie Cornaby, Ahmad Daryani, Gebre Demoz, Erika Eldrenkamp, Alireza Esteghamati, Nancy Fullman, Tsegaye Tewelde Gebrehiwot, Maryam Ghasemi-Kasman, Jiawei He, Behzad Heibati, Enayatollah Homaie Rad, Chad Ikeda, Caleb Irvine, Molla Kahssay, Tesfaye Kassa, Nicholas J Kassebaum, Yousef Khader, Young-Ho Khang, Adnan Kisa, Michael Kutz, Patrick Y Liu, Emilie Maddison, Helena Manguerra, Christopher Margono, Mohsen Mazidi, Robert Milam, Roghayeh Mohammadibakhsh, Maziar Moradi-Lakeh, Mohsen Naghavi, Emma Nichols, Bolajoko Olusanya, Kanyin Ong, Mahesh P A, Caroline Purcell, Anwar Rafay, Muhammad Aziz Rahman, Maheswar Satpathy, Jun She, Peilin Shi, Lavanya Singh, Alison Smith, Patrick Sur, Arash Tehrani-Banihashemi, Moti Tolera, Anna Torre, Avnish Tripathi, Rachel L Updike, Patrícia Pereira Vasconcelos De Oliveira, Yasir Waheed, Gelin Xu, Jamal Yearwood, Ebrahim M Yimer, Stephanie R M Zimsen, and Ben Zipkin.

Managing the overall research enterprise

Mohsen Afarideh, Ashkan Afshin, Tahiya Alam, Peter Allebeck, Nahla Anber, Till Bärnighausen, Deborah Carvalho Malta, Cyrus Cooper, Michael Criqui, Elizabeth Cromwell, Mahdi Fakhar, Mir Sohail Fazeli, Netsanet Fentahun, Daniel O Fijabi, Abraham Flaxman, Kyle J Foreman, Nancy Fullman, Thomas Fürst, Emmanuela Gakidou, Morsaleh Ganji, Vipin Gupta, Caitlin Hawley, Simon I Hay, Behnam Heidari, Amr Jamal, Pushpendra Kumar, Stephen S Lim, Rafael Lozano, Varshil Mehta, George Mensah, Awoke Misganaw, Ali Mokdad, Mohsen Naghavi, Bruno Nascimento, Carla Obermeyer, Helen E Olsen, Amanda Pain, Joseph Salama, Joshua A Salomon, Benn Sartorius, Chloe Shields, Stein Emil Vollset, Theo Vos, and Harvey Whiteford.

Did not provide contribution information

Netsanet Abera Asseffa, Shahrzad Bazargan-Hejazi, Kaylin Bolt, Adesegun Fatusi, Seyed Reza Hosseini, Mukti Khanal, Philip Mitchell, Glen Mola, Gabriele Nagel, Davide Rasella, Aletta Schutte, Peilin Shi, Abiy Sinke, JS Thakur, and Maigeng Zhou.

Preamble

This appendix provides methodological detail for estimating the Healthcare Access and Quality Index, as well as supplementary results. The appendix is organised into broad sections following the structure of the main paper. This study complies with the Guidelines for Accurate and Transparent Health Estimates Reporting (GATHER) recommendations. It includes detailed indicator modeling write-ups and flowcharts, and information on data sourcing in an effort to maximise transparency in our estimation processes and provide a comprehensive account of analytical steps. We intend this to be a living document, to be updated with each annual iteration of the Global Burden of Diseases, Injuries, and Risk Factors Study (GBD).

List of supplementary figures and tables

Supplementary figure 1. Comparing unscaled MIRs to age-standardised risk-standardised death rates across cancers. MIRs = mortality-to-incidence ratios. SDI = Socio-demographic index.

Supplementary figure 2. Comparing scaled MIRs to age-standardised risk-standardised death rates across cancers. MIRs = mortality-to-incidence ratios. SDI = Socio-demographic index.

Supplementary figure 3. Comparing PCA-derived cause weights for the HAQ Index in GBD 2016 and GBD 2016. PCA = principal components analysis. HAQ Index = Healthcare Access and Quality Index. GBD = Global Burden of Disease.

Supplementary figure 4. Map of HAQ Index values, by decile, in 1990 (A) and 2000 (B). Deciles were based on the distribution of HAQ Index values in 2016, as found in figure 1 of the main text, and then were applied for 1990 and 2000. HAQ Index = Healthcare Access and Quality Index. ATG=Antigua and Barbuda. VCT=Saint Vincent and the Grenadines. LCA=Saint Lucia. TTO=Trinidad and Tobago. TLS=Timor-Leste. FSM=Federated States of Micronesia.

Supplementary figure 5. Map of subnational HAQ Index values, by decile, in 1990, 2000, and 2016 for Japan (A), England (B), the USA (C), China (D), Mexico (E), Brazil (F), and India (G). Deciles were based on the distribution of HAQ Index values for countries and territories in 2016, as found in figure 1 of the main text, and then were applied for subnational locations over time. HAQ Index = Healthcare Access and Quality Index.

Supplementary figure 6. Map of subnational HAQ Index values, in 1990, 2000, and 2016, for Japan (A), England (B), the USA (C), China (D), Mexico (E), Brazil (F), and India (G). Scales were based on the range of HAQ Index values observed across subnational locations from 1990–2016. Due to similarities in their HAQ Index range, the same scales were used for Japan, England, and the USA, and then for Mexico and Brazil. HAQ Index = Healthcare Access and Quality Index.

Supplementary figure 7. Absolute change on the HAQ Index, by SDI quintile, from 1990 to 2016. Countries and territories are colour-coded by their SDI quintile, and are abbreviated according to the ISO3 code. HAQ Index = Healthcare Access and Quality Index. SDI = Socio-demographic Index.

Supplementary figure 8. Comparing the HAQ Index, by SDI quintile, from 1990–2016 (A), 1990–2000 (B), and 2000–2016 (C). Countries and territories are colour-coded by their SDI quintile, and are abbreviated according to the ISO3 code. HAQ Index = Healthcare Access and Quality Index. SDI = Socio-demographic Index.

Supplementary figure 9. Annualised rate of change on the HAQ Index, by SDI quintile, from 1990–2016 (A), 1990–2000 (B), and 2000–2016 (C). Countries and territories are colour-coded by their SDI quintile, and are abbreviated according to the ISO3 code. HAQ Index = Healthcare Access and Quality Index. SDI = Socio-demographic Index.

Supplementary figure 10. Absolute change on the HAQ Index and 32 individual causes, by country or territory, from 2000–2016. Countries are ranked by the difference in their HAQ Index in 2016 from their HAQ Index in 2000. Positive values represent improvements for overall the overall index and individual causes from 2000–2016, while negative values represent worsening performance during that time. HAQ Index = Healthcare Access and Quality Index.

Supplementary figure 11. Comparing the HAQ Index in 2016 to the log of government health spending per capita (A), the log of out-of-pocket health spending per capita (B), the log of DAH per capita (C), government health spending as a percent of total health spending (D), out-of-pocket health spending as a percent of total health spending (E), DAH as a percent of total health spending (F), and SDI (G). Total health spending, government health spending, out-of-pocket spending, and DAH per capita are based on the cumulative per capita spending from 2010 to 2015 (or 2016 in the case of DAH) in 2017 PPP. Percentages are based on the fraction of government health spending, out-of-pocket health spending, and DAH per capita from total health spending per capita for that period of time. Countries and territories are colour-coded by their SDI quintile, and are abbreviated according to the ISO3 code. HAQ Index = Healthcare Access and Quality Index. DAH = development assistance for health. SDI = Socio-demographic Index. PPP = purchasing power parity.

Supplementary figure 12. Comparing the HAQ Index in 2016 to physicians, nurses, and midwives per 1,000 (A), hospital beds per 1,000 (B), outpatient utilisation rates (C), and inpatient utilisation rates (D). Physicians, nurses, and midwives per 1,000 is based on the most recent location-year of data between 2010 and 2015 from the WHO Global Health Observatory database. Hospital beds per 1,000 were estimated for 2016. Outpatient utilisation rates are the annual number of outpatient visits per capita in 2016. Inpatient utilisation rates are the annual number of admissions for one night or more to a health facility per capita in 2016. Countries and territories are colour-coded by their SDI quintile, and are abbreviated according to the ISO3 code. HAQ Index = Healthcare Access and Quality Index. SDI = Socio-demographic Index.

Supplementary figure 13. **Comparing the PCA-derived HAQ Index with the arithmetic mean of the HAQ Index component parts, 2016.** The correlation between these two index construction methods is 0.994. PCA = principal components analysis. HAQ Index = Healthcare Access and Quality Index.

Supplementary figure 14. Comparing the GBD 2015 HAQ Index in 2015 to the GBD 2016 HAQ Index in 2016. GBD = Global Burden of Disease. HAQ Index = Healthcare Access and Quality Index.

Supplementary figure 15. Comparing rankings for the GBD 2015 HAQ Index in 2015 to the GBD 2016 HAQ Index in 2016. GBD = Global Burden of Disease. HAQ Index = Healthcare Access and Quality Index.

Supplementary table 1. List of ICD codes mapped to GBD causes for which mortality is amenable to healthcare. ICD=International Classification of Diseases. GBD = Global Burden of Disease.

Supplementary table 2. Factor loadings and weights from principal components analysis.

Supplementary table 3. Global, regional, national or territory, and select subnational estimates on the HAQ Index for 1990–2016, and absolute change and annualised rates of change for 1990–2016, 1990–2000, and 2000–2016. Starred estimates of change represent statistically significant changes during a given time period. HAQ Index=Healthcare Access and Quality Index.

Supplementary table 4. Associations between potential correlates of HAQ Index performance. All Pearson correlations with HAQ Index performance are for year 2016. Unless otherwise specified, we used the same year for indicators compared to the HAQ Index. For physicians, nurses, and midwives per 1,000, which is sourced by the WHO, we used the latest estimates reported by location from 2010-2015. HAQ Index=Healthcare Access and Quality Index. GBD=Global Burden of Disease. WHO=World Health Organization. DAH=development assistance for health.

GATHER statement

This study complies with the Guidelines for Accurate and Transparent Health Estimates Reporting (GATHER) recommendations. We have documented the steps involved in our analytical procedures and detailed the data sources used in compliance with the GATHER. For additional GATHER reporting, please refer to GATHER table on pages 6-7.

GATHER checklist of information that should be included in reports of global health estimates, with description of compliance and location of information

#	GATHER checklist item	Description of compliance	Reference
**Objectives and funding**			
1	Define the indicators, populations, and time periods for which estimates were made.	Narrative provided in paper and appendix describing indicators, definitions, and populations.	Summary; Main text; Appendix Part 1. Section 1-2
2	List the funding sources for the work.	Funding sources listed in paper.	Main text
**Data Inputs**			
For all data inputs from multiple sources that are synthesized as part of the study:			
3	Describe how the data were identified and how the data were accessed.	Narrative description of data seeking methodology provided.	Main text Methods; Appendix Parts 1-2
4	Specify the inclusion and exclusion criteria. Identify all ad-hoc exclusions.	Narrative about inclusion and exclusion criteria by data type provided; ad-hoc exclusions in cause-specific write-ups.	Main text Methods; Appendix Parts 1-2
5	Provide information on all included data sources and their main characteristics. For each data source used, report reference information or contact name/institution, population represented, data collection method, year(s) of data collection, sex and age range, diagnostic criteria or measurement method, and sample size, as relevant.	An interactive, online data source tool that provides metadata for data sources by component, geography, cause, risk, or impairment.	Appendix Part 3. Section 1.<br><br>Online data tools: http://ghdx.healthdata.org/gbd-2016
6	Identify and describe any categories of input data that have potentially important biases (e.g., based on characteristics listed in item 5).	Summary of known biases by cause included in cause-specific write-ups.	Appendix Part 2
For data inputs that contribute to the analysis but were not synthesized as part of the study:			
7	Describe and give sources for any other data inputs.	An interactive, online data source tool that provides metadata for data sources by component, geography, cause, risk, or impairment.	Appendix Part 3. Section 1.<br><br>Online data tools: http://ghdx.healthdata.org/gbd-2016
For all data inputs:			
8	Provide all data inputs in a file format from which data can be efficiently extracted (e.g., a spreadsheet as opposed to a PDF), including all relevant meta-data listed in item 5. For any data inputs that cannot be shared due to ethical or legal reasons, such as third-party ownership, provide a contact name or the name of the institution that retains the right to the data.	Downloads of input data are available through online tools, including data visualization tools and data query tools. Input data is not available in tools but can be made available upon request.	Appendix Part 3. Section 1.<br><br>Online data tools: http://ghdx.healthdata.org/gbd-2016

**Data analysis**			
9	Provide a conceptual overview of the data analysis method. A diagram may be helpful.	Flow diagrams of the overall methodological processes, as well as cause-specific modelling processes have been provided.	Main text; Appendix Parts 1-2
10	Provide a detailed description of all steps of the analysis, including mathematical formulae. This description should cover, as relevant, data cleaning, data pre-processing, data adjustments and weighting of data sources, and mathematical or statistical model(s).	Flow diagrams and corresponding methodological write-ups for each cause and modelling processes have been provided.	Appendix Parts 1-2
11	Describe how candidate models were evaluated and how the final model(s) were selected.	Provided in the methodological write-ups.	Main text Methods; Appendix Part 1. Section 2
12	Provide the results of an evaluation of model performance, if done, as well as the results of any relevant sensitivity analysis.	Provided in the methodological write-ups.	Appendix Part 1. Section 2
13	Describe methods for calculating uncertainty of the estimates. State which sources of uncertainty were, and were not, accounted for in the uncertainty analysis.	Provided in the methodological write-ups.	Main text Methods; Appendix Part 1. Section 2
14	State how analytic or statistical source code used to generate estimates can be accessed.	Access statement provided.	Links to code can be found here: http://ghdx.healthdata.org/gbd-2016
**Results and Discussion**			
15	Provide published estimates in a file format from which data can be efficiently extracted.	GBD 2016 results are available through online data visualization tools, the Global Health Data Exchange, and the online data query tool.	Main text Table 2; Appendix Part 3. Section 1.<br><br>Online data record: http://ghdx.healthdata.org/record/global-burden-disease-study-2016-gbd-2016-healthcare-access-and-quality-index-based-amenable
16	Report a quantitative measure of the uncertainty of the estimates (e.g. uncertainty intervals).	Uncertainty intervals are provided with all results.	Main text Table 2
17	Interpret results in light of existing evidence. If updating a previous set of estimates, describe the reasons for changes in estimates.	Discussion of methodological changes between HAQ 2015 provided in the narrative of the paper and appendix.	Research in Context; Main text; Appendix Part 4
18	Discuss limitations of the estimates. Include a discussion of any modelling assumptions or data limitations that affect interpretation of the estimates.	Discussion of limitations provided in the narrative of the main paper as well as in the methodological write-ups in the appendix.	Main text; Appendix Part 1. Section 2

Part 1. Estimating Healthcare Access and Quality Index

Section 1. Healthcare Access and Quality Index overview

Measuring death rates due to causes which are considered amenable to healthcare is a way to approximate average levels of personal healthcare access and quality. The Nolte and McKee list[1–5] of amenable causes is the most widely used framework by previous analyses of healthcare access and quality in higher-income countries. As part of the Global Burden of Diseases, Injuries, and Risk Factors Study 2015 (GBD 2015), we expanded upon past work and constructed the Healthcare Access and Quality (HAQ) Index to quantify personal healthcare access and quality for 195 countries and territories from 1990 to 2015.[6]

The GBD 2016 update to the HAQ Index uses the same overarching methodology developed in GBD 2015 (described in more detail below); the two main differences are (1) using mortality-to-incidence ratios (MIRs) for cancers instead of risk-standardised death rates; and (2) scaling by the 1st and 99th percentiles instead of the absolute minimum and maximum levels observed.

Section 2. Overall modelling strategy for the Healthcare Access and Quality Index

The methodology used in estimating the HAQ Index is visualised in the flowchart below.

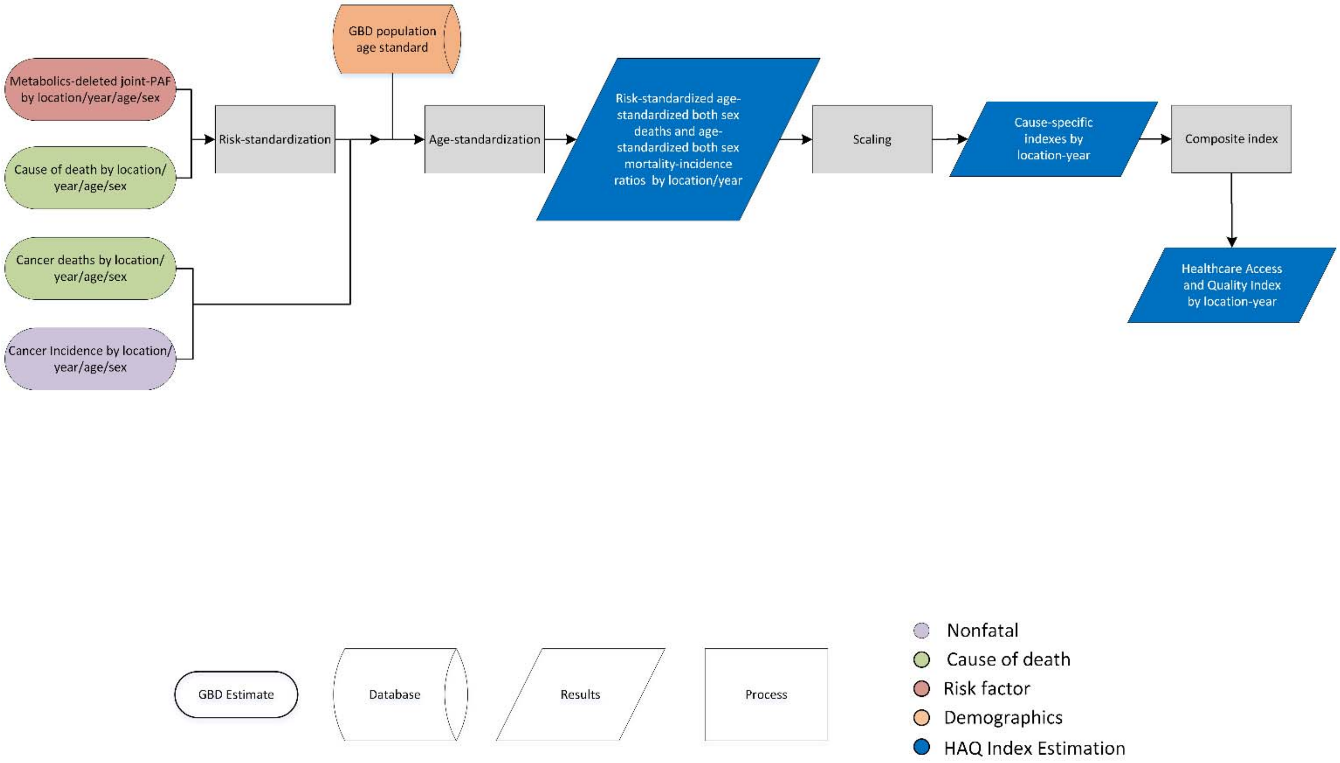

Input data and modelling strategies

The inputs to the HAQ Index were results from the GBD 2016 cause-specific mortality and comparative risk assessment analyses,[7,8] representing 693 most-detailed locations, 23 age groups, two sexes, and six years spanning a 26-year range (1990, 1995, 2000, 2005, 2010, and 2016). For each input, 1,000 draws were used in order to estimate uncertainty.

Input data and modelling strategies for estimating cause-specific mortality and population attributable fractions (PAFs) are detailed in GBD 2016 appendices,[7,8] and both input data and estimates can be downloaded through the Global Health Data Exchange (GHDx): http://ghdx.healthdata.org/gbd-2016

For non-cancers, location-year-age-sex-specific cause of death estimates from GBD 2016 are used directly. We estimated a joint-risk (PAF) for each cause, using all risks except three metabolic risks (high blood pressure, high total cholesterol, and high fasting plasma glucose), which are inextricably linked to personal healthcare, and thus would not be appropriate for risk-standardisation steps explained later. Assumptions about how one risk factor is mediated through other risk factors are needed in order to estimate the joint-risk factor burden for combinations of behavioural or environmental risks. To accomplish this, for every two risk factors for an outcome, we used published studies to estimate the fraction of risk that was mediated through the other risk. This resulted in a matrix of parameters containing each possible pairing of risk factors included in the GBD 2016.[8] Using this matrix, we computed the aggregated burden of disease at each level of the GBD 2016 hierarchy and for all risk factors using the following formula:

$$PAF_{J,o,a,s,l,t} = 1 - \prod_{j=1}^{J}\left(1 - PAF_{j,o,a,s,l,t}\prod_{i=1}^{J}(1 - MF_{j,i,o})\right)$$

where J is a set of risk factors for the aggregation; $PAF_{j,o,a,s,l,t}$ is the PAF for risk j for cause o, age group a, sex s, location l, and year t; and $MF_{j,i,o}$ is the mediation factor for risk j mediated through i for cause o.

Risk-standardisation of non-cancer deaths

The risk-standardisation process is a key innovation of this work on amenable mortality. The aim of this process is to eliminate the residual effects of localised risk exposure that would otherwise act as a confounding element in our ability to draw inferences about healthcare from mortality due to amenable causes. By imposing a global level of exposure on all locations, we de-contextualise them and create a measure of mortality that is a more appropriate proxy for healthcare access and quality. For non-cancer causes, deaths are risk-standardised using the formula:

$$RSD_{l,y,a,s} = D_{l,y,a,s} \times \left(1 - PAF_{l,y,a,s}\right) \times \frac{1}{1 - GPAF_{a,s}}$$

where $RSD_{l,y,a,s}$ is the risk-standardised deaths in location l, year y, age group a, and sex s; $D_{l,y,a,s}$ is the deaths for those specifications; $PAF_{l,y,a,s}$ is the PAF for those specifications; and $GPAF_{a,s}$ is the global PAF over all six estimation years for age group a, and sex s. If for a given cause no risk attribution

is present or all deaths are attributed to risks (ie, PAF of 0 or 1), the observed deaths are used. If any cause has a maximum observed mean joint-risk PAF greater than 0.9 but less than 1 for a given age and sex, PAFs across all location years are scaled such that the maximum is scaled down to 0.9.

Mortality-to-incidence ratios (MIRs) for cancers

In GBD, cancer mortality estimates are informed by MIRs that are derived from incidence and linked mortality data recorded in cancer registries. More detail on these data and derivation of MIRs can be found under the cancer cause-of-death write-up in this supplementary appendix (pp 41-49).

Due to the expansion of cancer registry data quantity and quality in GBD 2016, we tested the use of MIRs instead of risk-standardised death rates for the HAQ Index.[7] MIRs were not produced for non-melanoma skin cancer (squamous-cell carcinoma) for GBD 2016 due under-ascertainment of this cancer in registry data, and since MIRs calculated within the overall cancer cause-of-death estimation process are recalibrated to final mortality outputs, we constructed MIRs for each cancer based on post-CoDCorrect deaths (ie, a process that ensures internal consistently between all-cause mortality and cause-specific deaths) by age, sex, and location-year and final incidence estimates for the same dimensions.[9] We used the GBD 2016 final incidence and mortality estimates by age, sex, cancer, and location-year to generate the MIRs and limited the age groups to the bounds defined by the Nolte and McKee cause list. We age-standardised mortality and incidence rates per the age standardisation process described below, and then took the ratio of mortality to incidence to calculate MIRs.

To evaluate the potential effects of using MIRs instead of risk-standardised death rates for cancer, we ran a series of correlations with the Socio-demographic Index (SDI),[7] a summary measure of overall development based on average income per capita, educational attainment, and total fertility rates produced for every location-year in GBD on a scale of 0-1 (Table 1, below). We found substantially stronger associations with age-standardised MIRs and SDI across cancers, ranging from r=-0.96 for testicular and breast cancer to r=-0.63 (non-melanoma skin cancer [squamous-cell carcinoma]), than the correlations between age-standardised risk-standardised death rates and SDI. For the latter, r values ranged from r=-0.75 for cervical cancer to r=0.38 for colon and rectum cancer. The positive, albeit moderate, correlation with SDI and age-standardised risk-standardised death rates due to colon and rectum cancer reflects the challenge in using death rates for causes that are more common in high SDI countries and therefore have higher death rates and lower HAQ performance. In light of these results, we view using age-standardised MIRs for cancers a substantial improvement for HAQ Index estimation.

Table 1. Correlations (r) with SDI and age-standardised MIRs versus risk-standardised death rates by cancer site in 2016.

Cancer	Age-standardised MIR	Age-standardised risk-standardised death rate
Testicular cancer	-0.96	-0.11
Breast cancer	-0.96	-0.32
Uterine cancer	-0.94	-0.54
Colon and rectum cancer	-0.94	0.38
Hodgkin lymphoma	-0.93	-0.47

Cervical cancer	-0.88	-0.75
Leukaemia	-0.82	-0.06
Non-melanoma skin cancer (squamous-cell carcinoma)	-0.63	-0.12

Age-standardisation

Deaths outside of the ages defined in Table 1 in the main text were eliminated, as only deaths in those ages were deemed highly amenable to healthcare. We then aggregated cause-specific deaths by sex to both sexes. Using the GBD population age standard, we compiled risk-standardised deaths for non-cancers and the initial components of cancer MIRs (ie, mortality and incidence) for both sexes by location, year, and amenable cause:

$$RSASD_{l,y} = \sum_{a=1}^{n} RSD_{l,y,a} \times PAS_a$$

here $RSASD_{l,y,d}$ is the age-standardised risk-standardised deaths or component parts for MIRs for location l, and year y; $RSD_{l,y,a}$ is the risk-standardised deaths or component parts for MIRs in location l, year y, and age group a; and $PAS_a$is the population age standard for age group a.

Scaling

In order to have a standardised score of amenable mortality by cause across location-years, we then take the age-standardised MIRs and risk-standardised death rates and convert them to a 0 to 100 index value:

$$I_{l,y} = \frac{\log(RSASD_{l,y}) - 1\text{st}(\log(RSASD'_{CY}))}{99\text{th}(\log(RSASD'_{CY})) - 1\text{st}(\log(RSASD'_{CY}))}$$

where $I_{l,y}$ and $RSASD_{l,y}$ are the cause-specific index values and age-standardised MIRs and risk-standardised death rate for location l and year y, respectively. $RSASD'_{CY}$ is the matrix of MIRs and age-standardised risk-standardised death rates for the 195 countries and territories from which we identify the 1st and 99th percentile of draw-level values. The decision to use a percentile-based approach rather than the minimum and maximum values observed across location-years (ie, the method used for GBD 2015) was made to reduce sensitivity to outliers or fluctuations in estimates across GBD iterations. We applied the thresholds for 0 and 100 established at the country and territory level to subnational estimates by cause, and then scaled values between 0 and 100 accordingly. In order to eliminate zeroes while maintaining the observed range, we added one death per 1 million population to all values before log transformation.

Creating composite indicator

Using the above methodology, we have created 1,000 draws of location-year index values for 32 causes of death amenable to healthcare. We then constructed a composite measure using the 32 components, the overall HAQ Index, using principal components analysis (PCA).

To implement PCA, we first took the mean value of the 1,000 draws. We then produced a zero-centered matrix in which rows represented national level estimates from GBD and all six estimation years, and columns the 32 causes of death included in analysis. We aimed to use the factor loadings of these causes as weights in creating our summary indicator. The number of factors for which we retained loadings was determined on the basis that their cumulative variance explained exceeded 80%. Using those n factors, we created one composite factor by taking the average loading across factors, weighted by their explained variance. We last rescaled the composite loading to sum to 1 – these values would be used as weights in generating the summary indicator:

$$SI_{l,y} = \sum_{c=1}^{n} I_{l,y,c} \times weight_c$$

where $SI_{l,y}$, the summary indicator for location l and year y, is equal to the sum of the product of $I_{l,y,c}$ (index value for location l, year y, and cause c) and the weight for cause c across causes. Supplementary table 2 (appendix p 157) provides factor loadings, composite factors, and cause weights for each amenable cause.

The PCA weights derived at the national level were applied to subnational locations in constructing their overall HAQ Index scores. This approach differs from that of GBD 2015, where the most detailed GBD locations were used to establish PCA weights. Additions of GBD subnational locations are not at random and given the continuous expansion of subnational burden of disease assessments in the GBD annual cycle, restricting the derivation of PCA weights to national-level locations provides greater stability in the overall HAQ Index for future analyses.

References

1 Nolte E, McKee M. Measuring the health of nations: analysis of mortality amenable to health care. *BMJ* 2003; **327**: 1129.

2 Nolte E, McKee M. Does healthcare save lives? Avoidable mortality revisited. London, UK: Nuffield Trust, 2004.

3 Nolte E, McKee CM. Measuring The Health Of Nations: Updating An Earlier Analysis. *Health Aff (Millwood)* 2008; **27**: 58–71.

4 Nolte E, McKee M. Variations in amenable mortality--trends in 16 high-income nations. *Health Policy Amst Neth* 2011; **103**: 47–52.

5 Nolte E, McKee CM. In Amenable Mortality—Deaths Avoidable Through Health Care—Progress In The US Lags That Of Three European Countries. *Health Aff (Millwood)* 2012; **31**: 2114–22.

6 Barber RM, Fullman N, Sorensen RJD, *et al.* Healthcare Access and Quality Index based on mortality from causes amenable to personal health care in 195 countries and territories, 1990–2015: a novel analysis from the Global Burden of Disease Study 2015. *The Lancet* 2017; **390**: 231–66.

7 Naghavi M, Abajobir AA, Abbafati C, *et al.* Global, regional, and national age-sex specific mortality for 264 causes of death, 1980–2016: a systematic analysis for the Global Burden of Disease Study 2016. *The Lancet* 2017; **390**: 1151–210.

8 Gakidou E, Afshin A, Abajobir AA, *et al.* Global, regional, and national comparative risk assessment of 84 behavioural, environmental and occupational, and metabolic risks or clusters of risks, 1990–2016: a systematic analysis for the Global Burden of Disease Study 2016. *The Lancet* 2017; **390**: 1345–422.

9 Vos T, Abajobir AA, Abate KH, *et al.* Global, regional, and national incidence, prevalence, and years lived with disability for 328 diseases and injuries for 195 countries, 1990–2016: a systematic analysis for the Global Burden of Disease Study 2016. *The Lancet* 2017; **390**: 1211–59.

Part 2 to supplementary appendix: Tuberculosis

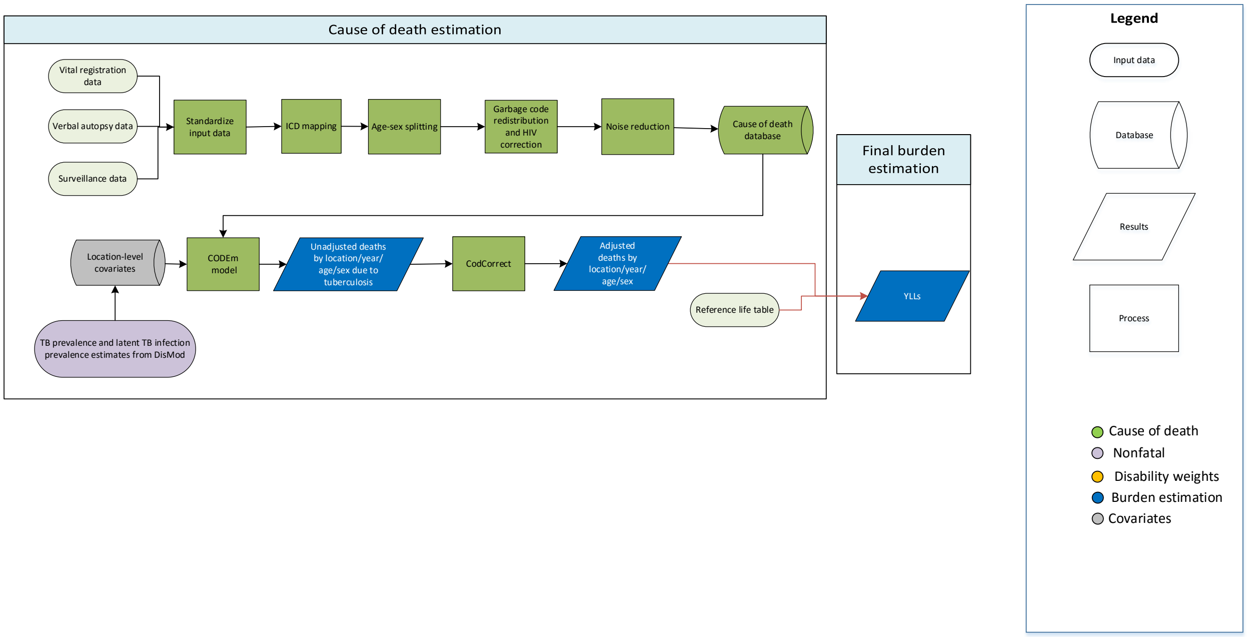

Input data

Input data for modeling tuberculosis mortality among HIV-negative individuals include vital registration, verbal autopsy, and surveillance data. Vital registration data were adjusted for garbage coding (including ill-defined codes, and the use of intermediate causes) following GBD algorithms and misclassified HIV deaths (i.e., HIV deaths being assigned to other underlying causes of death such as tuberculosis or diarrhea because of stigma or misdiagnosis). This correction was done based on examining changes in the age pattern of diseases over time.

Verbal autopsy data in countries with age-standardized HIV prevalence greater than 5% were removed because of a high probability of misclassification, as verbal autopsy studies have poor validity in distinguishing HIV deaths from HIV-TB deaths.

Modeling strategy

We changed the modeling strategy of tuberculosis in GBD2016 by first modeling prevalence of disease and prevalence of latent infection which were then used as covariates in the CODEm model. We dropped the health system access covariate and replaced it by the newly developed Healthcare Access and Quality Index covariate. We also added the adult underweight proportion covariate. Other location-level covariates included in the CODEm model were the same as in GBD 2015: alcohol (liters per capita), diabetes (fasting plasma glucose mmol/L), education (years per capita), lag-distributed income, indoor air pollution, outdoor air pollution, population density, smoking prevalence, sociodemographic status, and a summary exposure variable reflecting the average exposure to all of the risk factors.

Part 2 to supplementary appendix: Diarrheal diseases

Diarrheal diseases

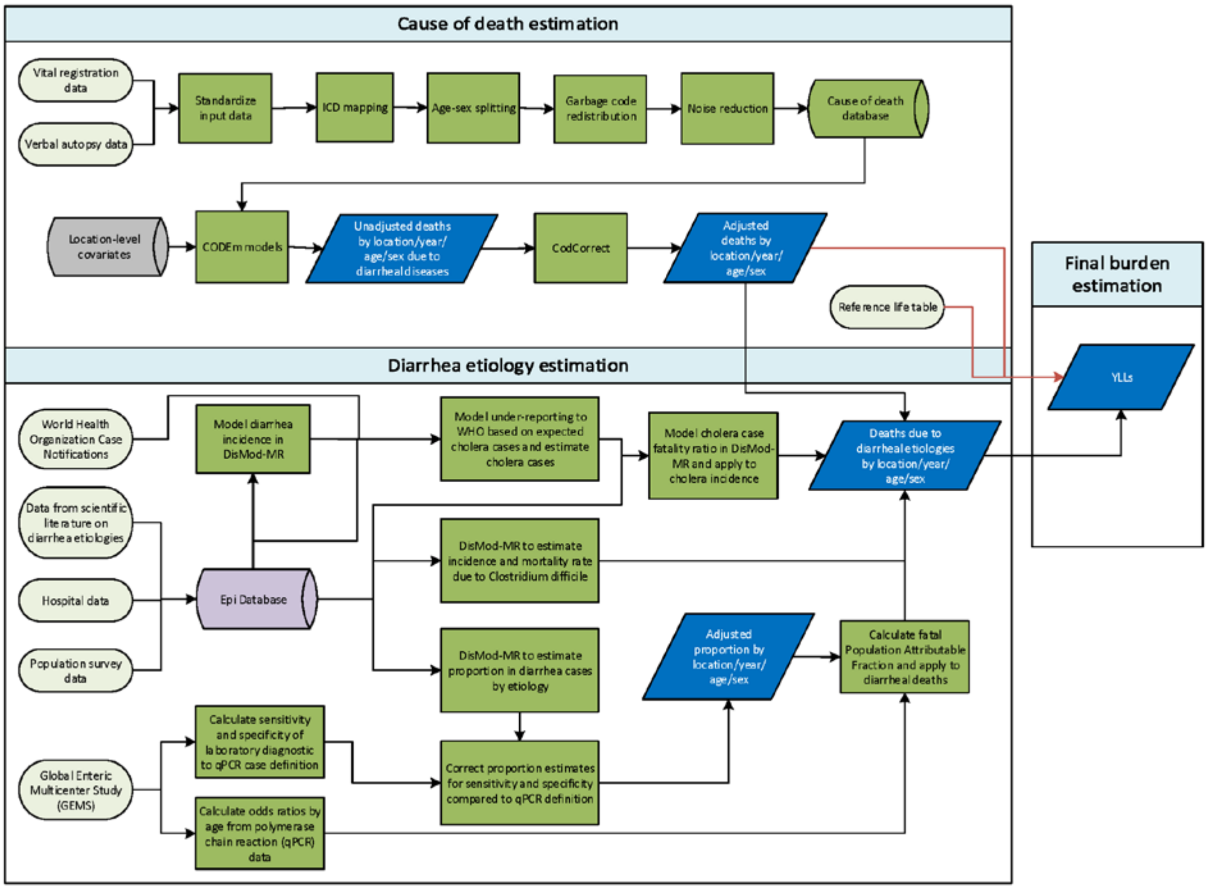

Input data

Cause of death. Diarrheal disease mortality was estimated in CODEm. We estimated diarrhea mortality separately for males and females and for children under 5 years and older than 5 years. We used all available data from vital registration systems, surveillance systems and verbal autopsy (**Table 1**). We checked for and excluded outliers from our data by country or region. We also excluded early neonatal mortality data in the Philippines (1994–1998) and India Civil Registration System data in all states (1986–1995).

Etiologies. We conducted a systematic literature review for the proportion of diarrhea cases that tested positive for each etiology. We updated our review of literature to include studies published between May 2015 and May 2016. Inclusion criteria included diarrhea as the case definition, studies with a sample size of at least 100, and studies with at least one year of follow-up. We excluded studies that reported on diarrheal outbreaks exclusively and those that used acute gastroenteritis with or without diarrhea. We identified 442 studies, of which 36 met our criteria of inclusion and were included. We extracted data points for location, sex, year, and age. We assigned an age range based on the prevalence-weighted mean age of diarrhea in the appropriate year/sex/location if the age of the study participants was not reported.

We used the Global Enteric Multicenter Study (GEMS), a seven-site, case-control study of moderate-to-severe diarrhea in children under 5 years,[1] to calculate odds ratios for the diarrheal pathogens. We analyzed raw data for a systematic reanalysis, representative of the distribution of cases and controls by age and site, of roughly half of the 22,000 original GEMS samples that were tested for the presence of pathogen using quantitative polymerase chain reaction (qPCR).[2]

Modeling strategy

Cause of death. We used country-level covariates to inform our CODEm models. We included covariates for years of education per capita, income per capita, prevalence of undernutrition (weight-for-age, weight-for-height, and height-for-age), population density above 1,000 or below 150 people per square kilometer, sanitation access, safe water access, Socio-Demographic Index, and rotavirus vaccine coverage. We evaluated our diarrheal disease cause of death models using in and out of sample predictive performance.

Etiologies. We estimated diarrheal disease etiologies separately from overall diarrhea mortality using a counterfactual strategy for enteric adenovirus, *Aeromonas*, *Entamoeba histolytica* (amoebiasis), *Campylobacter enteritis*, *Cryptosporidium*, typical enteropathogenic *Escherichia coli* (t-EPEC), enterotoxigenic *Escherichia coli* (ETEC), norovirus, non-typhoidal salmonella infections, rotavirus, and *Shigella*. *Vibrio cholerae* and *Clostridium difficile* were modeled separately.

Diarrheal etiologies are attributed to diarrheal deaths using a counter-factual approach. We calculated a population attributable fraction (PAF) from the proportion of severe diarrhea cases that are positive for each etiology. The PAF represents the relative reduction in diarrhea mortality if there was no exposure to a given etiology. As diarrhea can be caused by multiple pathogens and the pathogens may co-infect, PAFs can overlap and add up to more than 100%. We calculated the PAF from the proportion of severe diarrhea cases that are positive for each etiology. We assumed that hospitalized diarrhea cases are a proxy of severe and fatal cases. We used the following formula to estimate PAF:[4]

$$PAF = Proportion * (1 - \frac{1}{OR})$$

Where *Proportion* is the proportion of diarrhea cases positive for an etiology and *OR* is the odds ratio of diarrhea given the presence of the pathogen.

We dichotomized the continuous qPCR test result using the value of the cycle threshold (Ct) that most accurately discriminated between cases and controls. The Ct values range from 0 to 35 cycles representing the relative concentration of the target gene in the stool sample. A low value indicates a higher concentration of the pathogen while a value of 35 indicates the absence of the target in the sample. We used the lower Ct value when we had multiple Ct values for the cutpoint. The case definition for each pathogen is a Ct value that is below the established cutoff point.

We used a mixed effects conditional logistic regression model to calculate the odds ratio for under 1 year and 1-4 years old for each of our pathogens. The odds ratio for 1-4 years was applied to all GBD age groups over 5 years. There were three pathogen-age odds ratios that were not statistically significant: Aeromonas and Amoebiasis in under 1 year and Campylobacter in 1-4 years. The mean value of the odds ratio was above 1 in all three cases so we transformed the odds ratios for these three exceptions only in log-space such that exponentiated values could not be below 1. The transformation was:

$$Odds\ ratio = exp(log(or) - 1)) + 1$$

We modeled the proportion data using the meta-regression tool DisMod-MR to estimate the proportion of positive diarrhea cases for each separate etiology by location/year/age/sex and to adjust for the covariates.

We used the estimated sensitivity and specificity of the laboratory diagnostic technique used in the GEMS study compared to the qPCR case definition to adjust our proportion before we computed the PAF:[5]

$$Proportion_{True} = \frac{(Proportion_{Observed} + Specificity - 1)}{(Sensitivity + Specificity - 1)}$$

We used this correction to account for the fact that the proportions we used are based on a new test that is not consistent with the laboratory-based case definition (qPCR versus GEMS conventional laboratory testing for pathogens).[15]

Our literature review extracted the proportion of any enteropathogenic *Escherichia coli* (EPEC) without differentiating between typical (tEPEC) and atypical (aEPEC). In order to be consistent with the odds ratios that we obtained, we adjusted our proportion estimates of any EPEC to typical EPEC only. This adjustment was informed by a subset of our literature review that reported both atypical and typical EPEC. We estimated a ratio by super-region of tEPEC to any EPEC and adjusted our proportion estimates accordingly. We found that the majority of EPEC diarrhea cases were positive for atypical EPEC, consistent with other published work.[3]

For *Vibrio cholerae* (cholera), we used the literature review to estimate expected number of cholera cases for each country-year using the incidence of diarrhoea, estimated using DisMod-MR, and the proportion of diarrhoea cases that are positive for cholera. We assigned cholera PAF using odds ratios from the qPCR results to estimate a number of cholera-attributable cases. We compared this expected number of cholera cases to the number reported to the World Health Organization at the country-year level.[6] We modeled the underreporting fraction to correct the cholera case notification data for all countries using health system access and the diarrhoea SEV scalar to predict total cholera cases. We used the age-specific proportion of positive cholera samples in DisMod and our incidence estimates to predict the number of cholera cases for each age/sex/year/location. Finally, we modeled the case fatality ratio of cholera using DisMod-MR and to estimate the number of cholera deaths.

For *C. difficile*, we modeled incidence and mortality in DisMod-MR for each age, sex, year, location. DisMod-MR is a Bayesian meta-regression tool that uses spatio-temporal information as priors to estimate prevalence, incidence, remission, and mortality for *C. difficile* infection. DisMod-MR uses a compartmental model to relate prevalence, incidence, remission, and mortality. We set remission in our model to 1 month.

Table 1. Cause-specific mortality input data.

**Type of data**	**Input data**
**Total data sources**	**16,980 site-years**
Vital registration data	15,087 site-years
Surveillance data	877 site-years
Verbal autopsy data	1,016 site-years

References

1 Kotloff KL, Nataro JP, Blackwelder WC, *et al.* Burden and aetiology of diarrhoeal disease in infants and young children in developing countries (the Global Enteric Multicenter Study, GEMS): a prospective, case-control study. *Lancet Lond Engl* 2013; **382**: 209–22.

2 Liu J, Gratz J, Amour C, *et al.* A laboratory-developed TaqMan Array Card for simultaneous detection of 19 enteropathogens. *J Clin Microbiol* 2013; **51**: 472–80.

3 Ochoa TJ, Barletta F, Contreras C, Mercado E. New insights into the epidemiology of enteropathogenic Escherichia coli infection. *Trans R Soc Trop Med Hyg* 2008; **102**: 852–6.

4 Miettinen OS. Proportion of disease caused or prevented by a given exposure, trait or intervention. *Am J Epidemiol* 1974; **99**: 325–32.

5 Reiczigel J, Földi J, Ozsvári L. Exact confidence limits for prevalence of a disease with an imperfect diagnostic test. *Epidemiol Infect* 2010; **138**: 1674–8.

6 World Health Organization. Global Health Observatory data repository: Cholera. 2016. http://apps.who.int/gho/data/node.main.174?lang=en (accessed Aug 25, 2016).

Part 2 to supplementary appendix: Lower respiratory infections

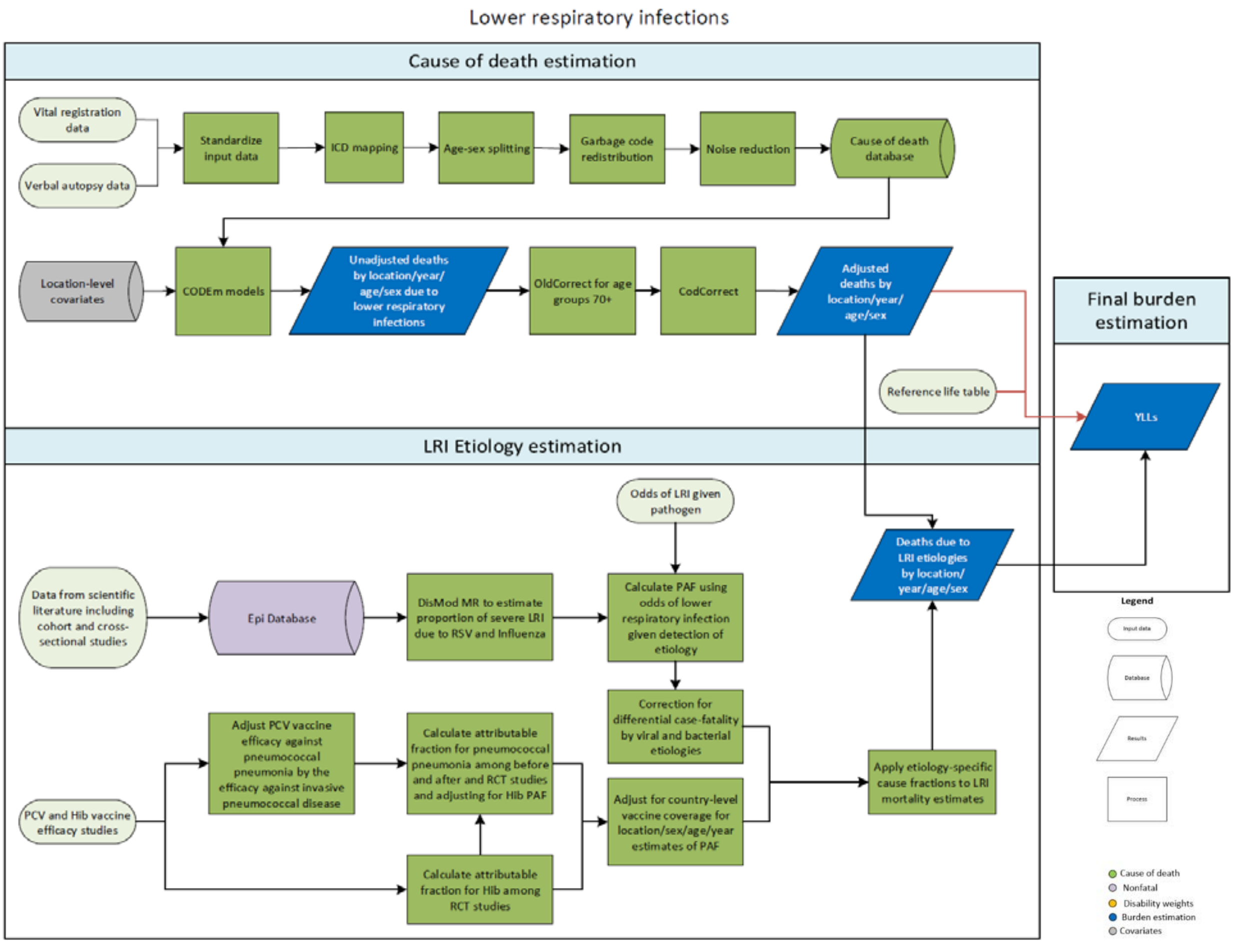

Input data

Cause of deaths. Lower respiratory infection (LRI) mortality was estimated in CODEm. We estimated LRI mortality separately for males and females and for children under 5 years and older than 5 years. We used all available data from vital registration systems, surveillance systems, and verbal autopsy (**Table 1**). We checked for and excluded outliers from our data by country or region. We also excluded ICD9-coded mortality data in Sri Lanka (1982, 1987–1992), ICD9-coded neonatal mortality data in Guatemala (1980, 1981, 1984, 2000–2004), and Civil Registration System data in many Indian states (1986–1995).

Etiologies. We updated our systematic review of scientific literature for the proportion of LRI that tested positive for influenza and respiratory syncytial virus (RSV) to include all data from GBD 2015 and from studies published between May 2015 and May 2016. Inclusion criteria were studies that had a sample size of at least 100, studies that were at least one year in duration, and studies describing lower respiratory infections, pneumonia, or bronchiolitis as the case definition. During our literature review we identified 209 studies, of which 7 met our inclusion criteria and were extracted. We excluded studies that described pandemic H1N1 influenza solely and studies that used influenza-like illness as the case definition. We assigned an age range based on the prevalence-weighted mean age of LRI in the appropriate year/sex/location if the ages of the study participants were not reported.

We also conducted a systematic literature review of studies on the Hib vaccine and PCV effectiveness studies against X-ray-confirmed pneumonia and against pneumococcal and Hib disease until May 2016. For PCV studies, we extracted, if available, the distribution of pneumococcal pneumonia serotypes and the serotypes included in the PCV used in the study. No new studies were identified for GBD 2016. We excluded observational and case-control studies due to implausibly high vaccine efficacy estimates. Hib trial data were exclusively from children <5 years so we did not include the effect of Hib on ages over 5 years of age. PCV trial data are also frequently limited to younger age populations. To understand the contribution of pneumococcal pneumonia in older populations, we also included PCV efficacy studies that used before-after approaches.

Modeling strategy

Cause of death. We used country-level covariates to inform our CODEm models. We included the following covariates in our LRI models: diphtheria-tetanus-pertussis vaccine coverage, years of education per capita, health system access, income per capita, prevalence of children malnutrition (<2 standard deviations below global mean of weight for age), prevalence of exposure to indoor air pollution (solid fuel use), outdoor air pollution level of $PM_{2.5}$, smoking prevalence, pneumococcal conjugate vaccine (PCV) coverage, *Haemophilus influenzae* type B (Hib) vaccine coverage, access to improved water, access to improved sanitation, and Socio-Demographic Index. We evaluated our LRI cause of death models using in and out of sample predictive performance.

Like all models of mortality in GBD, LRI mortality models are single-cause, requiring in effect that the sum of all mortality models must be equal to the all-cause mortality envelope. We correct LRI mortality estimates, and other causes of mortality, by re-scaling them according to the uncertainty around the cause-specific mortality rate. This process is called CoDCorrect and is essential to ensure internal consistency among causes of death. Before CoDCorrect, we also adjust LRI mortality for unreliable estimates due to improper death certification and ICD coding among elderly adults where the underlying cause of death should be Alzheimer's or Parkinson's diseases. This process scales LRI mortality among adult age groups 70+ years into a new envelope without Alzheimer's and Parkinson's. Further details can be found in section 4 of the appendix.

Etiologies. We estimated LRI etiologies separately from overall LRI mortality using two distinct counterfactual modeling strategies to estimate population attributable fractions (PAFs), described in detail below. The PAF represents the relative reduction in LRI mortality if there was no exposure to a given etiology. As LRIs can be caused by multiple pathogens and the pathogens may co-infect, PAFs can overlap and add up to more than 100%. Separate strategies were used for viral- influenza and respiratory syncytial virus (RSV)- and bacterial- *Streptococcus pneumoniae* and *Haemophilus influenzae* type B- etiologies. We did not attribute etiologies to neonatal pneumonia deaths due to a dearth of reliable data in this age group. We calculated uncertainty of our PAF estimates from 1,000 draws of each parameter using normal distributions in log space.

Influenza and RSV. We calculated the population attributable fraction (PAF) from the proportion of severe LRI cases positive for influenza and RSV. We assumed that hospitalized LRI cases are a proxy of severe cases. We used the following formula to estimate PAF:[1]

$$PAF = Proportion * (1-1/OR)$$

Where *Proportion* is the proportion of LRI cases that test positive for influenza or RSV and *OR* is the odds ratio of LRI given the presence of the pathogen. We used an odds ratio of 5.1 (3.19 – 8.14) for influenza and 9.79 (4.98 – 19.27) for RSV from a recently published meta-analysis.[2] These odds ratios are marginally different from those used in GBD 2013.

We modeled the proportion data using the meta-regression tool DisMod-MR to estimate the proportion of LRI cases that are positive for influenza and RSV, separately, by location/year/age/sex. We accounted for study-level covariates in our models such as PCR as the diagnostic technique, studies that investigated RSV or influenza exclusively, and studies from inpatient populations.

As the case-fatality of viral causes of pneumonia is lower than for bacterial causes, we adjusted for differential case-fatality by determining the etiological fractions for mortality attributable to RSV and influenza (**Table 2**). We measured the etiologic fractions by applying a relative case-fatality adjustment based on in-hospital case-fatality, which we coded to specific pneumonia etiologies. Hospital admissions data of this type were limited to data from the USA, Austria, Brazil, and Mexico. We generated the pooled estimate of the case-fatality differential between bacterial (pneumococcus, Hib) and viral etiologies (RSV, influenza) using DisMod-MR.

Pneumococcal pneumonia and Hib. For *Streptococcus pneumoniae* (pneumococcal pneumonia) and *Haemophilus influenzae* type B (Hib), we calculated the population attributable fraction using a vaccine probe design.[3,4] The ratio of vaccine effectiveness against nonspecific pneumonia to pathogen-specific disease represents the fraction of pneumonia cases attributable to each pathogen.

To estimate the PAF for Hib and pneumococcal pneumonia, we calculated the ratio of vaccine effectiveness against nonspecific pneumonia to pathogen-specific pneumonia (Equations 1 and 3). We estimated a study-level estimate of PAF from a meta-analysis of these ratios. To estimate the PAF for Hib, we only used randomized controlled trials because of implausibly high values of vaccine efficacy in case-control studies. To estimate the PAF for pneumococcal pneumonia, we included RCTs and before and after vaccine introduction longitudinal studies.

We adjusted the study-level PAF estimate by vaccine coverage and expected vaccine performance to estimate country- and year-specific PAF values. For pneumococcal pneumonia, we adjusted the PAF by the final Hib PAF estimate and by vaccine serotype coverage. Finally, we used an age distribution of PAF modeled in DisMod to determine the PAF by age. Because of an absence of data describing vaccine efficacy against Hib in children older than two years, we did not attribute Hib to episodes of LRI in ages five years and older.

We used a vaccine probe design to estimate the PAF for pneumococcal pneumonia and (Hib) by first calculating the ratio of vaccine effectiveness against nonspecific pneumonia to pathogen-specific pneumonia at the study level (Equations 1 and 2).[3–5] We then adjusted this estimate by vaccine coverage and expected vaccine performance to estimate country- and year-specific PAF values (Equations 3 and 4).

$$1)\quad HibPAF_{Base} = 1 - \frac{VE_{Pneumonia}}{VE_{Hib}}$$

$$2)\quad PneumoPAF_{Base} = 1 - \frac{VE_{Pneumonia} * (1 - PAF_{Hib} * VE_{Hib\ Optimal})}{VE_{Streptococcus} * Cov_{Serotype}}$$

3) $PAF_{Hib} = PAF_{Base} * \frac{(1-Cov_{Hib}*VE_{Hib\ Optimal})}{(1-PAF_{Base}*Cov_{Hib}*VE_{Hib\ Optimal})}$

4) $PAF_{Pneumo} = \frac{PAF_{Base}*(1-Cov_{PCV}*VE_{PCV\ Optimal})}{(1-PAF_{Hib}*Cov_{Hib}*VE_{Hib\ Optimal})*\left(1-\frac{PAF_{Base}Cov_{PCV}*VE_{PCV\ Optimal}}{(1-PAF_{Hib}*Cov_{Hib}*VE_{Hib\ Optimal})}\right)}$

Where $VE_{Pneumonia}$ is the vaccine efficacy against nonspecific pneumonia, VE_{Hib} is the vaccine efficacy against invasive Hib disease, $VE_{Streptococcus}$ is the vaccine efficacy against serotype-specific pneumococcal pneumonia, $Cov_{serotype}$ is the serotype-specific vaccine coverage for PCV,[6] $VE_{Hib\ Optimal}$ is the Hib effectiveness in the community (0.8)[7], PAF_{Hib} is the final PAF for Hib, Cov_{PCV} is the PCV coverage, Cov_{Hib} is the Hib coverage by country, and $VE_{PCV\ Optimal}$ is the vaccine effectiveness in the community (0.8).[8]

For Hib, we assumed that the vaccine efficacy against invasive Hib disease is the same against Hib pneumonia. For pneumococcal pneumonia, a recent study in adults [9] found that the vaccine efficacy against invasive pneumococcal disease may be significantly higher than against pneumococcal pneumonia. We used this ratio to adjust estimates of vaccine efficacy against invasive pneumococcal disease from other studies. However, recognizing that the study is unique in that it uses a urine antigen test among adults, we added uncertainty around our adjustment using a wide uniform distribution (median 0.65, 0.3-1.0). This has increased the estimates of pneumococcal pneumonia mortality in a meaningful way.

There are no major changes to the cause of death estimation strategy for LRI or its etiologies from GBD 2015 to GBD 2016.

Table 1. Summary of cause-specific mortality modeling input data.

Type of data	Input data
**Total data sources**	**12,155 site-years**
Vital registration data	10,312 site-years
Surveillance data	928 site-years
Verbal autopsy data	915 site-years

Table 2: **The median values for the ratio of viral to bacterial pneumonia case fatality ratio by age is shown.** These estimates are modeled using hospital-based, ICD-coded admissions and mortality for etiology-specified pneumonia. Values in parentheses represent 95% Uncertainty Interval.

**Age Group**	**Ratio**
Early Neonatal	0.34 (0.19-0.58)
Late Neonatal	0.34 (0.19-0.58)
Post Neonatal	0.34 (0.19-0.58)
1 to 4	0.28 (0.16-0.44)
5 to 9	0.31 (0.15-0.56)
10 to 14	0.33 (0.19-0.53)
15 to 19	0.37 (0.2-0.64)
20 to 24	0.46 (0.12-1.16)
25 to 29	0.44 (0.17-0.93)
30 to 34	0.46 (0.22-0.83)
35 to 39	0.5 (0.22-1)
40 to 44	0.61 (0.13-1.75)
45 to 49	0.5 (0.21-0.99)
50 to 54	0.44 (0.23-0.74)
55 to 59	0.42 (0.21-0.75)
60 to 64	0.42 (0.15-0.95)
65 to 69	0.39 (0.19-0.7)
70 to 74	0.38 (0.21-0.61)
75 to 79	0.37 (0.2-0.62)
80 to 84	0.37 (0.17-0.71)
85 to 89	0.34 (0.19-0.59)
90 to 94	0.33 (0.16-0.61)
95 to 99	0.34 (0.13-0.8)

References

1 Miettinen OS. Proportion of disease caused or prevented by a given exposure, trait or intervention. *Am J Epidemiol* 1974; **99**: 325–32.

2 Shi T, McLean K, Campbell H, Nair H. Aetiological role of common respiratory viruses in acute lower respiratory infections in children under five years: A systematic review and meta-analysis. *J Glob Health* 2015; **5**: 10408.

3 Feikin DR, Scott JAG, Gessner BD. Use of vaccines as probes to define disease burden. *Lancet Lond Engl* 2014; **383**: 1762–70.

4 O'Brien KL, Wolfson LJ, Watt JP, *et al.* Burden of disease caused by Streptococcus pneumoniae in children younger than 5 years: global estimates. *Lancet Lond Engl* 2009; **374**: 893–902.

5 Watt JP, Wolfson LJ, O'Brien KL, *et al.* Burden of disease caused by Haemophilus influenzae type b in children younger than 5 years: global estimates. *Lancet Lond Engl* 2009; **374**: 903–11.

6 Johnson HL, Deloria-Knoll M, Levine OS, *et al.* Systematic evaluation of serotypes causing invasive pneumococcal disease among children under five: the pneumococcal global serotype project. *PLoS Med* 2010; **7**. DOI:10.1371/journal.pmed.1000348.

7 Swingler G, Fransman D, Hussey G. Conjugate vaccines for preventing Haemophilus influenzae type B infections. *Cochrane Database Syst Rev* 2007; : CD001729.

8 Lucero MG, Dulalia VE, Nillos LT, *et al.* Pneumococcal conjugate vaccines for preventing vaccine-type invasive pneumococcal disease and X-ray defined pneumonia in children less than two years of age. *Cochrane Database Syst Rev* 2009; : CD004977.

9 Bonten MJM, Huijts SM, Bolkenbaas M, *et al.* Polysaccharide conjugate vaccine against pneumococcal pneumonia in adults. *N Engl J Med* 2015; **372**: 1114–25.

Part 2 to supplementary appendix: Upper respiratory infections

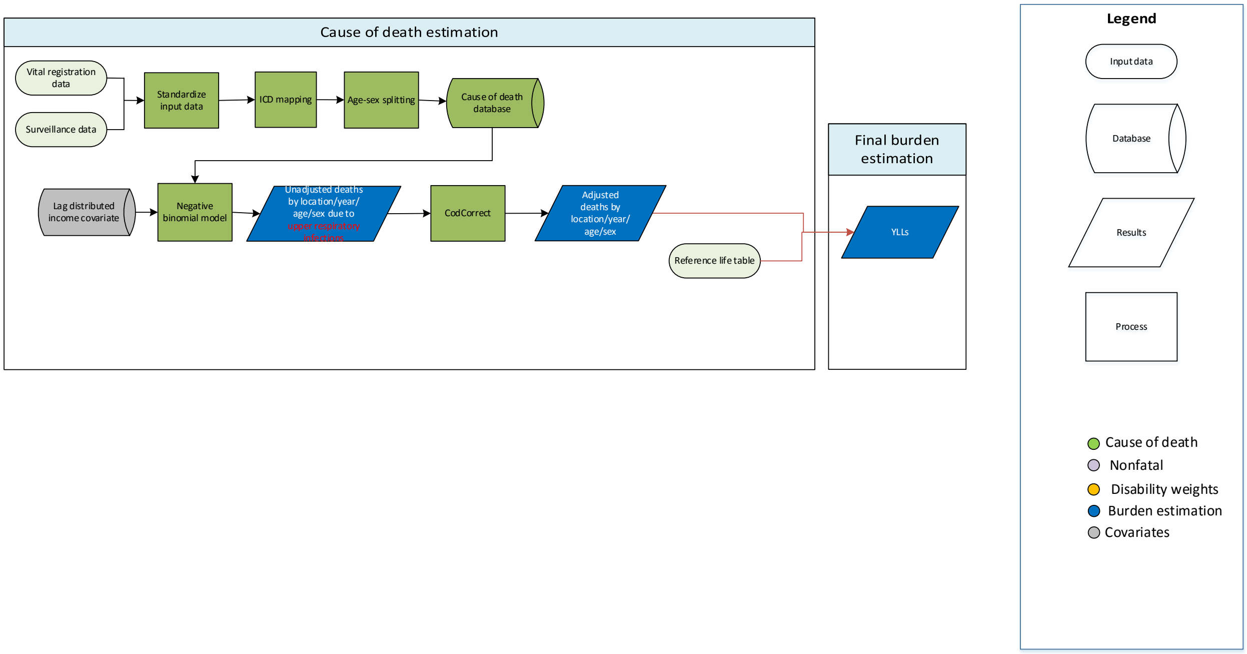

Input data

Vital registration and surveillance data from the cause of death database were used. Data with very high cause fractions (those greater than the 99th percentile values) were excluded in the regression.

Modeling strategy

Due to a small number of deaths, mortality from upper respiratory infections was modeled using a negative binomial regression, which is more appropriate than a Poisson count model as it accounts for greater variance (over-dispersion) in the data. By utilizing the exposure option in Stata, we model cause fractions with a negative binomial model. We tested both rate- and cause fraction-based models but selected a cause fraction model due to better model performance. Using the input data mentioned above, we modeled mortality from upper respiratory infections using the lag distributed income covariate and age dummy variables and the exposure set to the total number of deaths in the study. Uncertainty was estimated by taking 1,000 iterations of the predictions based on the variance covariance matrix and a random sample from a gamma distribution. The fit of the model was evaluated using diagnostic plots of predicted versus observed values.

Part 2 to supplementary appendix: Diphtheria

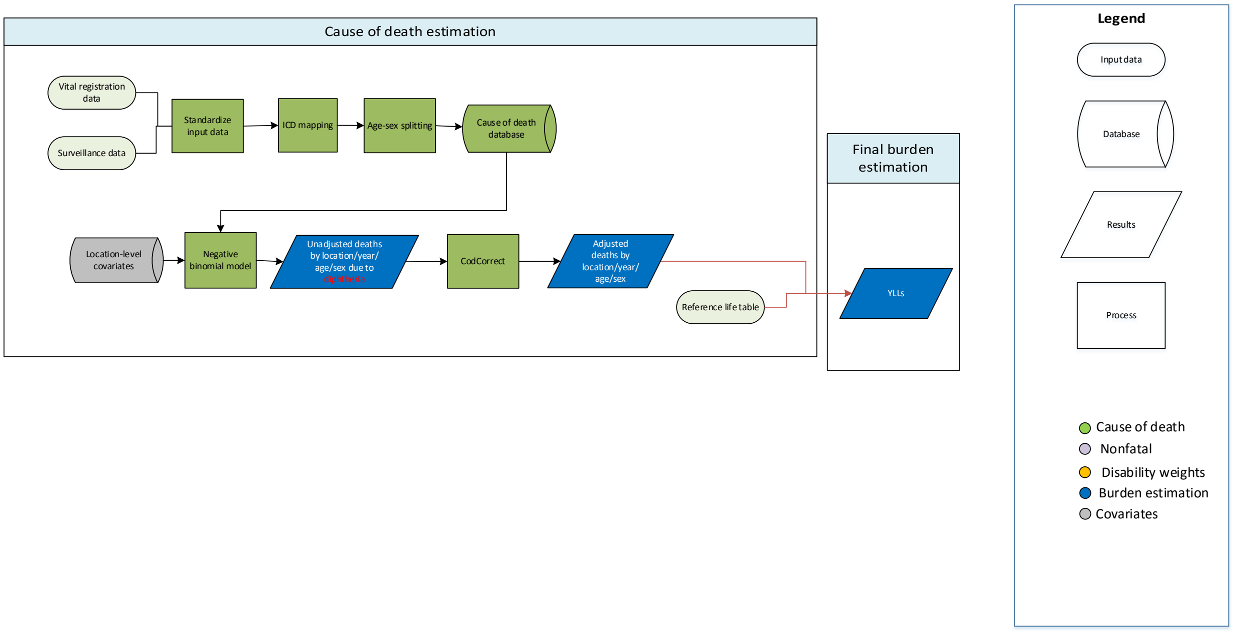

Input data

Vital registration and surveillance data from the cause of death database were used. Data with very high cause fractions (those greater than the 99th percentile values) were excluded in the regression.

Modeling strategy

Due to the small number of deaths, diphtheria mortality was modeled using a negative binomial regression, which is more appropriate than a Poisson count model as it accounts for greater variance (over-dispersion) in the data. Using the input data mentioned above, we modeled mortality due to diphtheria with the diphtheria-pertussis-tetanus third-dose (DPT3) vaccine coverage covariate and age dummy variables, with the offset as the total number of deaths in the study. Uncertainty was estimated by taking 1,000 iterations of the predictions based on the variance-covariance matrix and a random sample of the dispersion parameter from a gamma distribution.

Part 2 to supplementary appendix: Pertussis (whooping cough)

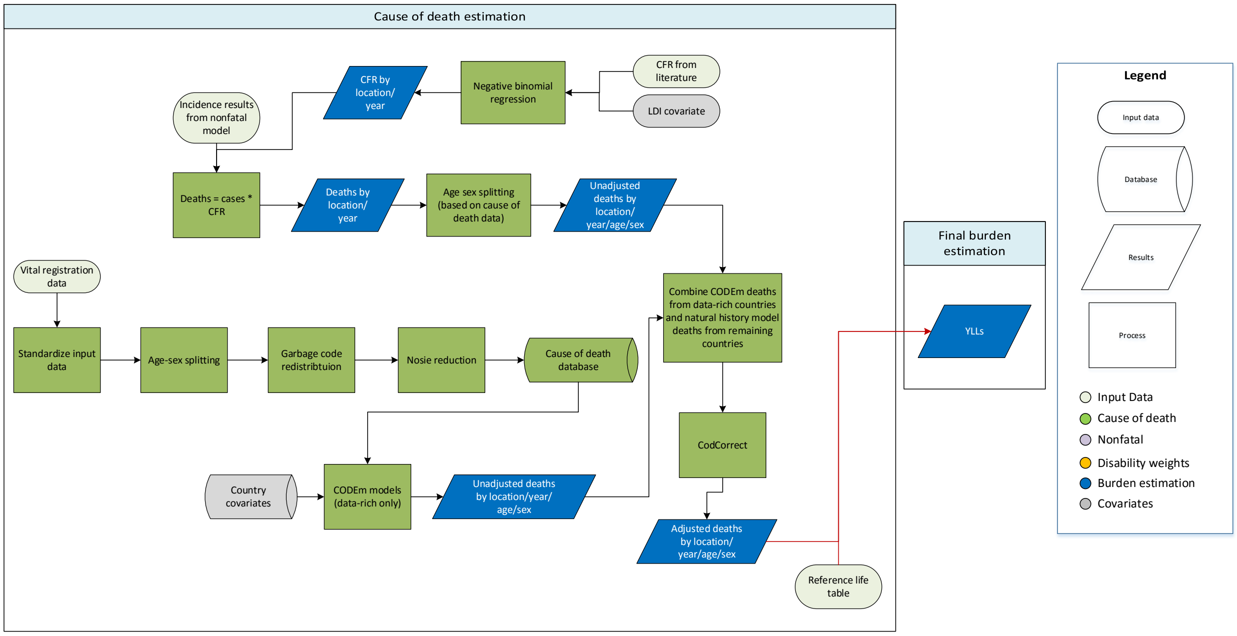

Input data

Vital registration data from the cause of death database were used for data-rich countries. To inform the natural history model, we used data from the following sources: World Health Organization (WHO) case notifications; historical case notifications for the United Kingdom back to 1940; case fatality data identified by collaborators; and case fatality data identified through systematic literature reviews for GBD 2010, GBD 2013, and GBD 2016. The PubMed search query for GBD 2016 was: (whooping cough [Title/Abstract]) OR (pertussis [Title/Abstract]) AND (case fatality [Title/Abstract]) AND ("2013"[Date - Publication]: "2016"[Date - Publication]). Studies were included if they reported case fatality rate, number of deaths, and number of cases. Studies were excluded if they included non-representative samples only.

Modeling strategy – data-rich countries

Mortality was modeled separately for data-rich and other countries. For data-rich countries (i.e., countries with vital registration more than 95% complete for more than 25 years), we used a general CODEm strategy with DPT3 vaccination coverage, lagged distributed income, and education as country-level covariates. We made estimations for the age range post-neonatal to 59 years.

Modeling strategy – other countries

For the remaining countries, we used a natural history-based model because CODEm does not predict well for those countries. First, we modeled log-transformed incidence with a mixed-effects linear regression of case notifications from the WHO (1985-2015) on diphtheria-tetanus-pertussis dose 3 (DTP3) vaccination coverage. Historical data of United Kingdom (UK) pertussis cases and UK DTP3 coverage rates (both back to 1940) were also used to inform the incidence model. The random effect by

country allowed for registration completeness to vary by country. The results of this model were then used to predict incidence as a function of vaccine coverage. To correct for underreporting in case notifications, we used a value of the random effect that matched the highest random effect in a high income region—Switzerland (which has a pertussis monitoring system which captures a high percentage of cases)—to get an implied attack rate assumed to be the same for all unvaccinated populations. Uncertainty was estimated by taking 1,000 iterations of the predictions based on the variance-covariance matrix.

Second, we modeled the pertussis case fatality rate using a negative binomial model with the health system access and lagged-distributed income covariates. Uncertainty was estimated by taking 1,000 iterations of the predictions based on the variance-covariance matrix and a random sample from a gamma distribution of the dispersion parameter. Finally, whooping cough deaths were calculated at the 1,000-draw level as

$$deaths = incidence * CFR \ .$$

We estimated overall number of deaths and then assigned an age-sex distribution based on the age- and sex-specific patterns found in the cause of death data. We made estimations for the age range post-neonatal to 59 years.

Part 2 to supplementary appendix: Tetanus

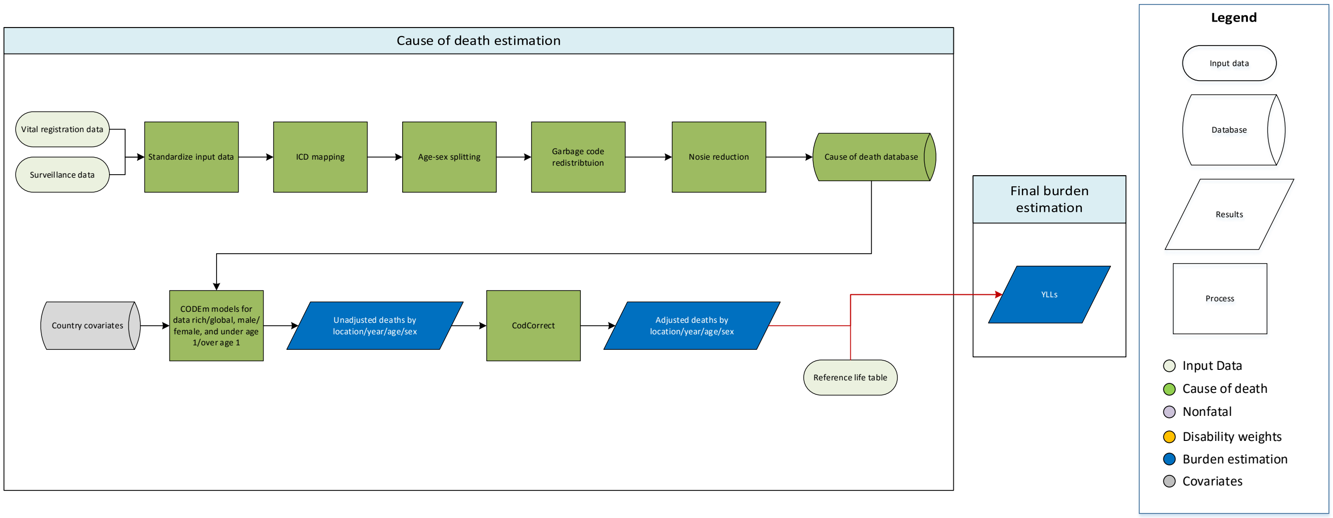

Input data

Mortality data from vital registration, verbal autopsy, and surveillance sources were used. Data were outliered if they largely conflicted with the majority of data from other studies conducted either in the same or different countries with similar sociodemographic characteristics in the same region.

Modeling strategy

A general CODEm modeling strategy was used. We ran separate models for under 1 year and 1 to 95+ years. There were no substantive changes in modeling strategy from GBD 2015.

Part 2 to supplementary appendix: Measles

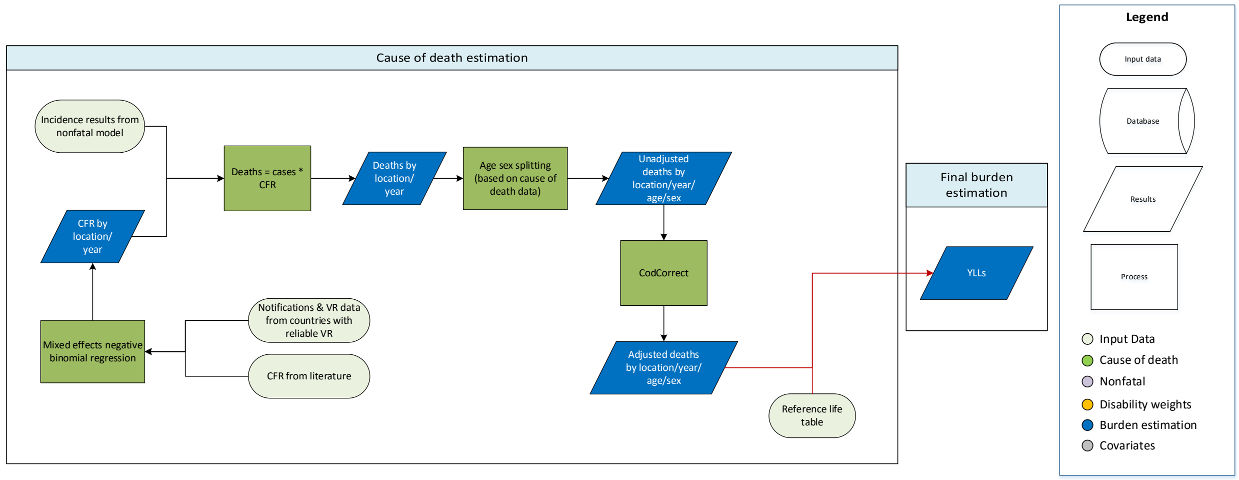

Input data

Vital registration data from the cause of death database were used for data-rich countries. To inform the natural history model, we used data from the following sources: World Health Organization (WHO) case notifications from 1995 to 2015; case notifications identified by collaborators; vital registration (VR) data in countries in the following three super-regions: high-income, Central Europe/Eastern Europe/Central Asia, and Latin America and Caribbean; and case fatality data identified through systematic literature reviews for GBD 2010, GBD 2013, and GBD 2016. The PubMed search query for GBD 2016 was: (measles [Title/Abstract]) AND (case fatality [Title/Abstract]) AND ("2013"[Date - Publication]: "2016"[Date - Publication]). Studies were included if they reported case fatality rate, number of deaths, and number of cases. Studies were excluded if they included non-representative samples only.

Modeling strategy – data-rich countries

Mortality was modeled separately for data-rich and other countries. For data-rich countries (i.e., countries with vital registration more than 95% complete for more than 25 years), we used a general CODEm strategy to model VR data with measles-containing vaccination dose one (MCV1) coverage, childhood malnutrition, lagged distributed income the healthcare access and quality index, and education as country-level covariates. We made estimations for the age range post-neonatal to 59 years.

Modeling strategy – other countries

Measles mortality in the remaining countries was modeled using a natural-history-based model. First, we modeled measles incidence with a mixed-effects linear regression of case notifications from the WHO (1995-2015) on routine measles vaccination rates and supplementary immunization activities (SIAs). More precisely, log-transformed incidence rates were regressed on the log of the proportion unvaccinated with first- and second-dose measles-containing vaccine, and additional SIA coverage lagged by one, two, three, four, and five years, with super-region, region, and country-level random effects. The results of this mixed effects regression model were then used to predict location-year-

specific incidence as a function of routine vaccine coverage and SIAs. To correct for underreporting in case notifications, we added the effect of a 95% attack rate, assumed to be the same across all unvaccinated populations. Uncertainty was estimated by taking 1,000 iterations of the predictions based on the variance-covariance matrix. For locations in three super-regions—high-income, Central Europe/Eastern Europe/Central Asia and Latin America and Caribbean—we used reported measles cases as incident cases.

Second, the case fatality rate was modeled using a mixed effects negative binomial regression with the child malnutrition covariate and study-level indicators (hospital-based or not; outbreak or not; and rural or urban/mixed), with country random effects. Uncertainty was estimated by taking 1,000 iterations of the predictions based on the variance-covariance matrix and uncertainty in country random effects. The fit of the model was evaluated using diagnostic plots of predicted versus observed values. Finally, estimated deaths were calculated at the 1,000-draw level as

$$deaths = incidence * CFR \ .$$

We estimated overall number of deaths and then assigned an age-sex distribution based on the age- and sex-specific patterns found in the cause of death data. We made estimations for the age range post-neonatal to 59 years.

Part 2 to supplementary appendix: Maternal disorders

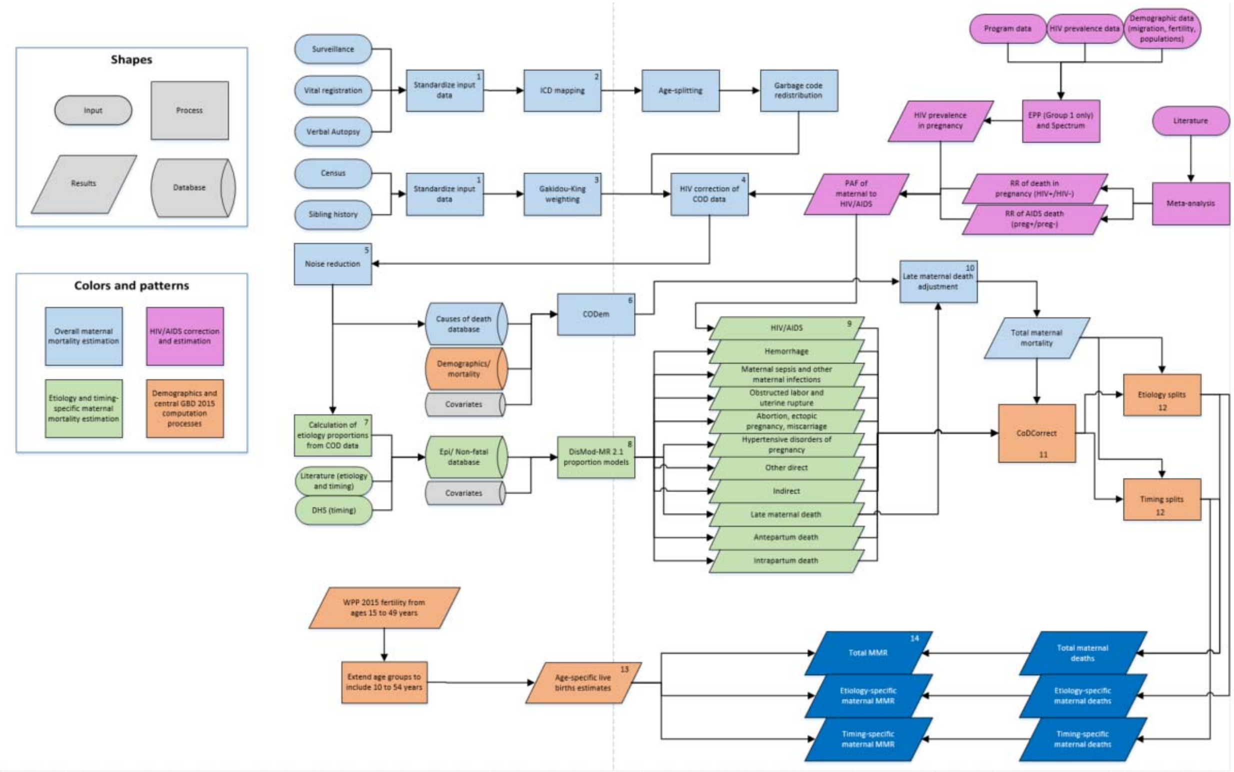

Input Data & Methodological Summary

Input data

CODEm models were informed by centrally prepped data stored in the cause of death (COD) database using standardized processes to adjust for bias due to incompleteness, misclassification, coding to non-specific causes (i.e. garbage codes), stochastic variability, and zero counts. Our GBD 2016 case definition for maternal mortality continues to be all pregnancy-related deaths excluding accidental or incidental causes up to 1 year after the end of the pregnancy.

An updated literature review to inform the relative risk of mortality in pregnancy in HIV-positive versus HIV-negative women produced 23 leads and one usable source. We completed this search on August 30, 2016, using the following search string:

(HIV[Title/Abstract] OR "Acquired Immunodeficiency Syndrome"[Title/Abstract] OR AIDS[Title/Abstract]) AND (“pregnant”[Title/Abstract] OR “pregnancy”[Title/Abstract] OR “postpartum”[Title/Abstract] OR "post partum"[Title/Abstract]) AND (“mortality”[Title/Abstract] OR “death”[Title/Abstract]) NOT "case report" AND "humans"[MeSH Terms] AND (2011/07/06[PDat] : 2016/12/31[PDat])

Correction for incidental HIV deaths was completed during the data preparation phase. Spectrum outputs of HIV prevalence in pregnancy were combined with relative risk of mortality during pregnancy (HIV+ versus HIV-negative) to calculate PAFs. A proportion of these deaths are incidental and a proportion are maternal as determined from two studies that looked at the relative risk of death in HIV positive women who are pregnant versus non-pregnant. All data were corrected using the PAFs. Incidental deaths were removed from sibling history and census data, while maternal HIV deaths were added to VR data. The maternal proportion of the PAF was retained to be combined with estimates of the aetiologic-proportion from other causes as described below.

DisMod-MR 2.1 aetiology proportion models were informed by two sources of data. First, we completed a systematic literature review on August 30, 2016, using the search string below:

(((("maternal mortality"[Title/Abstract] OR "maternal death"[Title/Abstract] OR "MM"[Title/Abstract] OR "confidential enquiry"[Title/Abstract] OR ((obstetric[Title/Abstract] OR pregnancy[Title/Abstract]) AND (etiology[Title/Abstract] OR cause[Title/Abstract] or pattern[Title/Abstract]) AND (death[Title/Abstract] OR mortality[Title/Abstract])))) AND "humans"[MeSH Terms] NOT (fetal[Title/Abstract] OR newborns[Title/Abstract] OR newborn[Title/Abstract] OR neonatal[Title/Abstract] OR “case report”[Title/Abstract] OR “case study”[Title/Abstract] OR pathogenesis[Title/Abstract] OR thromboprophylaxis[Title/Abstract])) OR ((“maternal mortality”[Title/Abstract] OR “maternal death*”[Title/Abstract] OR “MMR”[Title/Abstract]) AND (“Afghanistan”[Title/Abstract] OR “Albania”[Title/Abstract] OR “Algeria”[Title/Abstract] OR “Andorra”[Title/Abstract] OR “Angola”[Title/Abstract] OR “Antigua and Barbuda”[Title/Abstract] OR “Argentina”[Title/Abstract] OR “Armenia”[Title/Abstract] OR “Azerbaijan”[Title/Abstract] OR “Bahrain”[Title/Abstract] OR “Bangladesh”[Title/Abstract] OR “Barbados”[Title/Abstract] OR “Belarus”[Title/Abstract] OR “Belize”[Title/Abstract] OR “Benin”[Title/Abstract] OR “Bhutan”[Title/Abstract] OR “Bolivia”[Title/Abstract] OR “Bosnia and Herzegovina”[Title/Abstract] OR “Botswana”[Title/Abstract] OR “Brazil”[Title/Abstract] OR “Brunei”[Title/Abstract] OR “Bulgaria”[Title/Abstract] OR “Burkina Faso”[Title/Abstract] OR “Burundi”[Title/Abstract] OR “Cambodia”[Title/Abstract] OR “Cameroon”[Title/Abstract] OR “Cape Verde”[Title/Abstract] OR “Central African Republic”[Title/Abstract] OR “Chad”[Title/Abstract] OR “China”[Title/Abstract] OR “Colombia”[Title/Abstract] OR “Comoros”[Title/Abstract] OR “Congo”[Title/Abstract] OR “Costa Rica”[Title/Abstract] OR “Croatia”[Title/Abstract] OR “Cuba”[Title/Abstract] OR “Cyprus”[Title/Abstract] OR “Côte d’Ivoire”[Title/Abstract] OR “Democratic Republic of the Congo”[Title/Abstract] OR “Djibouti”[Title/Abstract] OR “Dominica”[Title/Abstract] OR “Dominican Republic”[Title/Abstract] OR “Ecuador”[Title/Abstract] OR “Egypt”[Title/Abstract] OR “El Salvador”[Title/Abstract] OR “Equatorial Guinea”[Title/Abstract] OR “Eritrea”[Title/Abstract] OR “Ethiopia”[Title/Abstract] OR “Federated States of Micronesia”[Title/Abstract] OR “Fiji”[Title/Abstract] OR “Gabon”[Title/Abstract] OR “Georgia”[Title/Abstract] OR “Ghana”[Title/Abstract] OR “Grenada”[Title/Abstract] OR “Guatemala”[Title/Abstract] OR “Guinea”[Title/Abstract] OR “Guinea-Bissau”[Title/Abstract] OR “Guyana”[Title/Abstract] OR “Haiti”[Title/Abstract] OR “Honduras”[Title/Abstract] OR “India”[Title/Abstract] OR “Indonesia”[Title/Abstract] OR “Iran”[Title/Abstract] OR “Iraq”[Title/Abstract] OR “Jamaica”[Title/Abstract] OR “Jordan”[Title/Abstract] OR “Kazakhstan”[Title/Abstract] OR “Kenya”[Title/Abstract] OR “Kiribati”[Title/Abstract] OR “Kuwait”[Title/Abstract] OR “Kyrgyzstan”[Title/Abstract] OR “Laos”[Title/Abstract] OR “Latvia”[Title/Abstract] OR “Lebanon”[Title/Abstract] OR “Lesotho”[Title/Abstract] OR “Liberia”[Title/Abstract] OR “Libya”[Title/Abstract] OR “Lithuania”[Title/Abstract] OR “Macedonia”[Title/Abstract] OR “Madagascar”[Title/Abstract] OR “Malawi”[Title/Abstract] OR “Malaysia”[Title/Abstract] OR “Maldives”[Title/Abstract] OR “Mali”[Title/Abstract] OR “Malta”[Title/Abstract] OR “Marshall Islands”[Title/Abstract] OR “Mauritania”[Title/Abstract] OR “Mauritius”[Title/Abstract] OR “Moldova”[Title/Abstract] OR “Mongolia”[Title/Abstract] OR “Montenegro”[Title/Abstract] OR “Morocco”[Title/Abstract] OR “Mozambique”[Title/Abstract] OR “Myanmar”[Title/Abstract] OR “Namibia”[Title/Abstract] OR “Nepal”[Title/Abstract] OR “Nicaragua”[Title/Abstract] OR “Niger”[Title/Abstract] OR “Nigeria”[Title/Abstract] OR “North Korea”[Title/Abstract] OR “Oman”[Title/Abstract] OR “Pakistan”[Title/Abstract] OR “Palestine”[Title/Abstract] OR “Panama”[Title/Abstract] OR “Papua New Guinea”[Title/Abstract] OR “Paraguay”[Title/Abstract] OR “Peru”[Title/Abstract] OR “Philippines”[Title/Abstract] OR “Qatar”[Title/Abstract] OR “Romania”[Title/Abstract] OR “Russia”[Title/Abstract] OR “Rwanda”[Title/Abstract] OR “Saint Lucia”[Title/Abstract] OR “Saint Vincent and the Grenadines”[Title/Abstract] OR “Samoa”[Title/Abstract] OR “Saudi

Arabia”[Title/Abstract] OR “Senegal”[Title/Abstract] OR “Serbia”[Title/Abstract] OR “Seychelles”[Title/Abstract] OR “Sierra Leone”[Title/Abstract] OR “Singapore”[Title/Abstract] OR “Solomon Islands”[Title/Abstract] OR “Somalia”[Title/Abstract] OR “South Africa”[Title/Abstract] OR "South Sudan"[Title/Abstract] OR “Sri Lanka”[Title/Abstract] OR “Sudan”[Title/Abstract] OR “Suriname”[Title/Abstract] OR “Swaziland”[Title/Abstract] OR “Syria”[Title/Abstract] OR “São Tomé and Príncipe”[Title/Abstract] OR “Taiwan”[Title/Abstract] OR “Tajikistan”[Title/Abstract] OR “Tanzania”[Title/Abstract] OR “Thailand”[Title/Abstract] OR “The Bahamas”[Title/Abstract] OR “The Gambia”[Title/Abstract] OR “Timor-Leste”[Title/Abstract] OR “Togo”[Title/Abstract] OR “Tonga”[Title/Abstract] OR “Trinidad and Tobago”[Title/Abstract] OR “Tunisia”[Title/Abstract] OR “Turkmenistan”[Title/Abstract] OR “Uganda”[Title/Abstract] OR “Ukraine”[Title/Abstract] OR “United Arab Emirates”[Title/Abstract] OR “Uruguay”[Title/Abstract] OR “Uzbekistan”[Title/Abstract] OR “Vanuatu”[Title/Abstract] OR “Venezuela”[Title/Abstract] OR “Vietnam”[Title/Abstract] OR “Yemen”[Title/Abstract] OR “Zambia”[Title/Abstract] OR “Zimbabwe”[Title/Abstract]) AND "humans"[MeSH] NOT (“demographic and health survey*”[Title/Abstract] OR DHS[Title/Abstract] OR “reproductive health survey*”[Title/Abstract] OR RHS[Title/Abstract]))) AND (2015/04/30[PDat] : 2016/12/31[PDat])) OR ((HIV[Title/Abstract] OR "Acquired Immunodeficiency Syndrome"[Title/Abstract] OR AIDS[Title/Abstract]) AND (“pregnant”[Title/Abstract] OR “pregnancy”[Title/Abstract] OR “postpartum”[Title/Abstract] OR "post partum"[Title/Abstract]) AND (“mortality”[Title/Abstract] OR “death”[Title/Abstract]) NOT "case report" AND "humans"[MeSH Terms] AND (2011/07/06[PDat] : 2016/12/31[PDat]))

A total of 698 sources were reviewed for their title and abstract. Of those selected for full text review, 17 had usable data for aetiology-specific maternal mortality models. All data were prepped as “proportion” of total maternal deaths due to that cause. The second source of data was from the COD database. All aetiology-specific COD data were processed to be “proportion” data by calculating the cause-specific deaths divided by the total maternal deaths for the matching data source, year, age, and location. Owing to the large volume of total COD data and small sample sizes in many locations, COD data were collapsed around each of the five-year periods for which DisMod-MR 2.1 makes distinct estimates (1990, 1995, 2000, 2005, 2010, and 2016). Late maternal death data were only included for the subset of locations where they were reliably coded in raw VR. All data were uploaded to the nonfatal database.

Modelling strategy

Overall maternal mortality was estimated with CODEm. All data from all geographies were reviewed. Outliers were identified as those data where age patterns or temporal patterns were inconsistent with neighbouring age groups or locations or where sparse data were predicting implausible overall temporal or age patterns for a given location.

DisMod-MR 2.1 proportion models for each sub-cause of maternal mortality were all single-parameter meta-regression models. Because many sources do not include the entire cause list, a series of study covariates were used to facilitate crosswalking back to the reference definition. The reference definition ***includes*** “other” direct obstetric complications, indirect maternal deaths, and late maternal death. Country covariates were specific for each model and included abortion legality (for abortion, ectopic pregnancy, and miscarriage), log-transformed lag-distributed income (for sepsis and late maternal death), and logit-transformed in-facility delivery proportion (for haemorrhage, hypertensive disorders of pregnancy, and obstructed labour). The time window was set at +/- 2 years for all models except late maternal death, which was +/- 5 years. The narrower window ensured that any given year of VR data only informed a single estimate.

We corrected the time trend in the CODEm model by identifying the year in which each location began consistently using O95 and O96 codes for late maternal death. These were identified as the earliest year in which the threshold proportion of total maternal deaths coded to late exceeded the lowest reported in the literature (0.5%). After a location was identified as having started using late maternal death codes, we

assumed that practice continued. We adjusted upward results for all years prior to the advent of late maternal death coding using the outputs of the late maternal death proportion DisMod model.

Etiology-specific estimates were derived by multiplying the proportion outputs from DisMod-MR 2.1 by the total maternal deaths for that age-group, location, and year. HIV-related maternal deaths were estimated for all locations using the PAF approach described above for mortality data processing.

ICD10 and ICD9 codes used for maternal disorders

Model	ICD10 code	ICD9 code
Abortion, ectopic pregnancy, miscarriage	O00-O08, O36.4	631, 633-639
Maternal hemorrhage	O20, O43.2, O44-O46, O62.2, O67, O72	640-641, 661.0, 666
Hypertensive disorders of pregnancy	O11-O16	642.3, 642.4, 642.5, 642.6, 642.7, 642.9
Obstructed labor and uterine rupture	O64-O66, O71, O83	659-660, 662, 665, 669.5, 669.6
Maternal sepsis and other infections	O23, O41, O75.2-3, O85, O86, O91	646.5, 646.6, 659.2, 659.3, 670, 672.0, 674.1, 674.2, 674.3, 675
Other maternal disorders	O09-O09.93, O21-O22-O22.93, O26-26.93, O28-O28.9, O29-O29.93, O30-O35.9, O40-O43.93, O47-48.1, O60-O61.9, O63-O63.9, O68-O70.9, O73-O77.9, O80-O84, O87-O90.9, O92-O92.79	646-646.44, 646.7-646.93, 648.1-649.9
Indirect maternal disorders	O24-O25.3, O98-O99.91	647-649.64

DisMod Proportion Models Covariates and Coefficients

Abortion, ectopic pregnancy and miscarriage

Study-level covariate	Parameter	Geography level	beta	Exponentiated beta
Only Maternal Direct Causes	Proportion	Global	0.20 (0.19 — 0.20	1.22 (1.21 — 1.22)

Hospital Inpatient	Proportion	Global	0.30 (0.30 — 0.30)	1.35 (1.35 — 1.35)
Late maternal deaths not included	Proportion	Global	- 0.20 (0.20 — 0.20)	1.22 (1.22 — 1.22)
Country-Level Covariate				
Legality of Abortion	Proportion	Global	0.054 (0.054 — 0.055)	1.06 (1.06 — 1.06)

Maternal hemorrhage

Study-level covariate	Parameter	Geography level	beta	Exponentiated beta
Only Maternal Direct Causes	Proportion	Global	0.20 (0.18 — 0.20)	1.22 (1.20 — 1.22)
Hospital Inpatient	Proportion	Global	0.30 (0.29 — 0.30)	1.35 (1.34 — 1.35)
Late maternal deaths not included	Proportion	Global	0.20 (0.20 — 0.20)	1.22 (1.22 — 1.22)
Country-level covariate				
In-Facility Delivery (proportion)	Proportion	Global	0.100 (0.100 — 0.100)	1.11 (1.10 — 1.11)

Hypertensive disorders of pregnancy

Study-level covariate	Parameter	Geography level	beta	Exponentiated beta
Only Maternal Direct Causes	Proportion	Global	0.20 (0.19 — 0.20)	1.22 (1.21 — 1.22)
Hospital Inpatient	Proportion	Global	0.30 (0.30 — 0.30)	1.35 (1.34 — 1.35)
Late maternal deaths not included	Proportion	Global	0.20 (0.20 — 0.20)	1.22 (1.22 — 1.22)
Country-level covariate				
In-Facility Delivery (proportion)	Proportion	Global	0.100 (0.100 — 0.100)	1.11 (1.11 — 1.11)

Obstructed labor and uterine rupture

Study-level covariate	Parameter	Geography level	beta	Exponentiated beta
Only Maternal Direct Causes	Proportion	Global	0.20 (0.20 — 0.20)	1.22 (1.22 — 1.22)
Hospital Inpatient	Proportion	Global	0.30 (0.30 — 0.30)	1.35 (1.35 — 1.35)

Late maternal deaths not included	Proportion	Global	0.20 (0.20 — 0.20)	1.22 (1.22 — 1.22)
Country-level covariate				
In-Facility Delivery (proportion)	Proportion	Global	0.100 (0.100 — 0.100)	1.11 (1.11 — 1.11)

Maternal sepsis and other infections

Study-level covariate	Parameter	Geography level	beta	Exponentiated beta
Only Maternal Direct Causes	Proportion	Global	0.20 (0.20 — 0.20)	1.22 (1.22 — 1.22)
Hospital Inpatient	Proportion	Global	0.30 (0.30 — 0.30)	1.35 (1.35 — 1.35)
Late maternal deaths not included	Proportion	Global	0.20 (0.20 — 0.20)	1.22 (1.22 — 1.22)
Country-level covariates				
LDI ($ per capita)	Proportion	Global	0.100 (0.100 — 0.100)	1.11 (1.10 — 1.11)

Other Maternal Disorders

Study-level covariate	Parameter	Geography level	beta	Exponentiated beta
Only Maternal Direct Causes	Proportion	Global	0.20 (0.19 — 0.20)	1.22 (1.21 — 1.22)
Hospital Inpatient	Proportion	Global	0.20 (0.19 — 0.20)	1.22 (1.21 — 1.22)
Late maternal deaths not included	Proportion	Global	0.20 (0.20 — 0.20)	1.22 (1.22 — 1.22)
Country-level covariate				
LDI ($ per capita)	Proportion	Global	0.100 (0.100 — 0.100)	1.11 (1.10 — 1.11)

Indirect Maternal Disorders

Study-level covariate	Parameter	Geography level	beta	Exponentiated beta
Hospital Inpatient	Proportion	Global	0.20 (0.20 — 0.30)	1.22 (1.22 — 1.22)
Late maternal deaths not included	Proportion	Global	0.20 (0.20 — 0.20)	1.22 (1.22 — 1.22)
Country-level covariate				

LDI ($ per capita)	Proportion	Global	0.100 (0.100 — 0.100)	1.11 (1.11 — 1.11)

Part 2 to supplementary appendix: Neonatal disorders

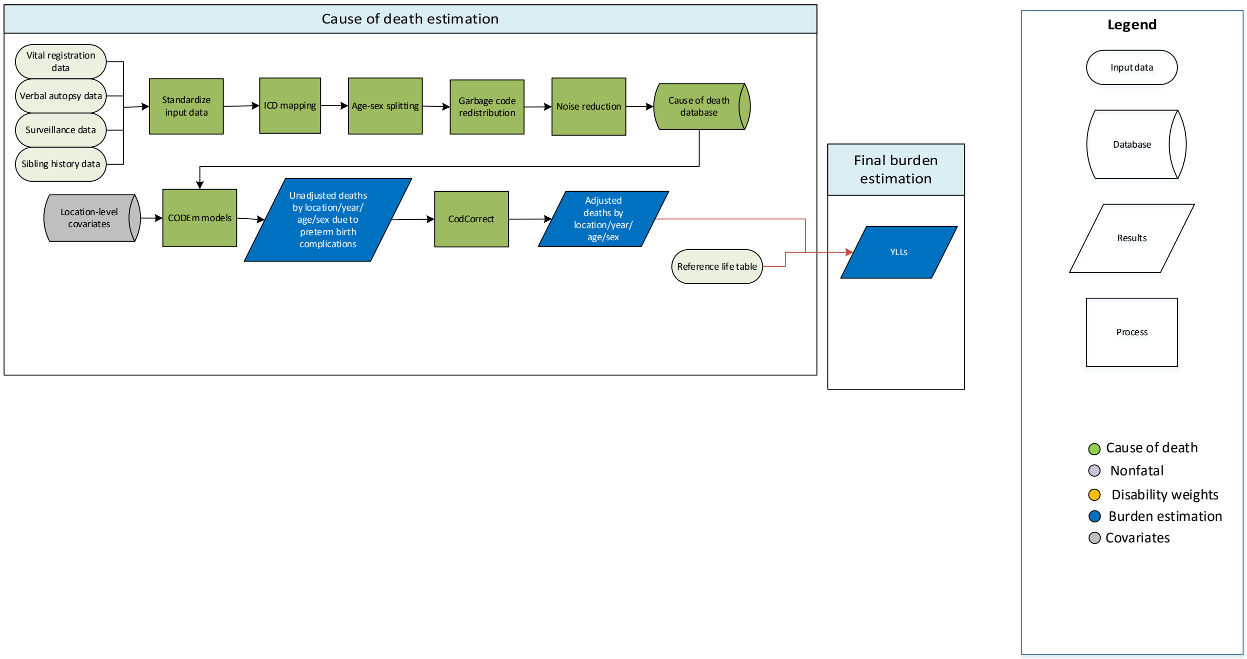

Input data

For the neonatal disorders envelope, preterm birth complications, and neonatal encephalopathy, vital registration, verbal autopsy, surveillance, and sibling history data were used for GBD 2016 to estimate number of deaths from each condition. For sepsis and other neonatal infections, vital registration, surveillance, and sibling history data were used. And for neonatal hemolytic disease and other neonatal conditions, vital registration and surveillance data were used. For all neonatal causes of death, vital registration was by far the most common data type. We only modelled deaths among males and females under age 5. Data points were selected as outliers if they were implausibly high, low, or significantly conflicted with established age or temporal patterns. Addition of significant new data from the Sample Registration System (SRS) in India had a significant effect on the estimates of mortality due to neonatal conditions at the global level.

Modelling strategy

For GBD 2016, an ensemble modelling approach was used via CODEm to model each of the different neonatal conditions. The same was done for GBD 2013 and 2015.

Varying levels of data quality and coding issues may still have affected our results. Validation studies suggest that verbal autopsy methods tend to be less accurate for cause of death ascertainment in the neonatal age groups.[1–4] This implies that in regions such as sub-Saharan Africa or South Asia, where the data primarily come from verbal autopsy studies, the distribution of sub-causes within all neonatal conditions may be less accurate. Furthermore, validation studies suggest that verbal autopsy methods tend to be particularly poor at ascertaining deaths from neonatal sepsis. Thus, for GBD 2016, all verbal autopsy data were excluded for neonatal sepsis and neonatal hemolytic disease.

Selected Covariates

Covariate	Transformation	Level	Direction
Education (years per capita)	None	3	-1
Health System Access	None	2	-1
In-Facility Delivery	None	2	-1
LDI (I$ per capita)	Log	3	-1
Underweight (proportion <2SD weight for age, <5 years)	None	2	1
Live Births 35+	None	2	1
Indoor Air Pollution (All cooking fuels)	None	1	1
Smoking prevalence (Reproductive Age-Standardized)	None	1	1
Total Fertility Rate	Log	3	1
SDI	None	3	-1
HAQI	None	2	-1
Skilled Birth Attendance	None	2	-1
Antenatal Care (4 visit)	None	2	-1

References

1 Anker M, Black RE, Coldham C, *et al.* A Standard Verbal Autopsy Method for Investigating Causes of Death in Infants and Children. Geneva, Switzerland: World Health Organization Department of Communicable Disease Surveillance and Response; The Johns Hopkins School of Hygiene and Public Health; The London School of Hygiene and Tropical Medicine, 1999.

2 Kalter HD, Gray RH, Black RE, Gultiano SA. Validation of postmortem interviews to ascertain selected causes of death in children. *Int J Epidemiol* 1990; **19**: 380–6.

3 Quigley MA, Armstrong Schellenberg JR, Snow RW. Algorithms for verbal autopsies: a validation study in Kenyan children. *Bull World Health Organ* 1996; **74**: 147–54.

4 Snow RW, Armstrong JR, Forster D, *et al.* Childhood deaths in Africa: uses and limitations of verbal autopsies. *The Lancet* 1992; **340**: 351–5.

Part 2 to supplementary appendix: Cancers

Input data and methodological summary for all cancers except for non-melanoma skin cancer

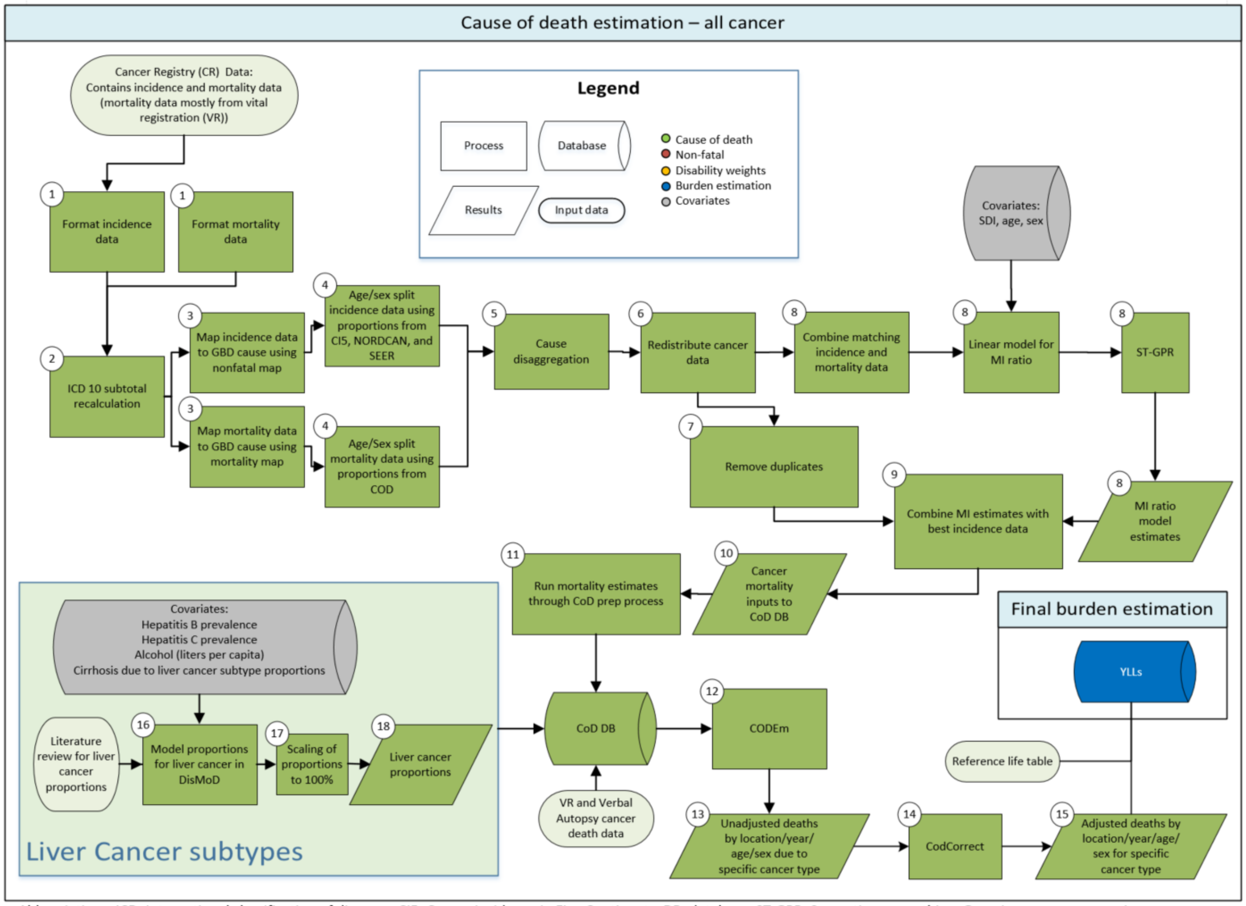

Abbreviations: ICD: International classification of diseases; CI5: Cancer Incidence in Five Continents, DB: database, ST-GPR: Space-time smoothing, Gaussian process regression

Data

The cause of death (COD) database contains multiple sources of cancer mortality data. These sources include vital registration, verbal autopsy, and cancer registry data. The cancer registry mortality estimates that are uploaded into the COD database stem from cancer registry incidence data that have been transformed to mortality estimates through the use of mortality-to-incidence ratios (MIR).

Data seeking processes

Cancer mortality data in the cause of death database other than cancer registry data

Sources for cancer mortality data other than cancer registry data are described in the COD database description (Section 2).

Cancer registry data

Cancer registry data were used from publicly available sources or provided by collaborators. We attempted to collect data from all registries that are members of the International Association of Cancer Registries (IACR) by either downloading publicly available data or contacting the registries. We also used cancer registry databases like Cancer Incidence in Five Continents (CI5), EUREG, and NORDCAN.[1–9]

Most cancer registries only report cancer incidence. However, if a cancer registry also reported cancer mortality, mortality data were also extracted from the source to be used in the MIR estimation.

Inclusion and exclusion criteria

Only population-based cancer registries were included, and only those that included all cancers (no specialty registries), data for all age groups, and data for both sexes. Pathology-based cancer registries were included if they had a defined population. Hospital-based cancer registries were excluded. Cancer registry data were excluded from either the final incidence data input or the MI model input if a more detailed source (e.g., providing more detailed age or diagnostic groups) was available for the same population. Preference was given to registries with national coverage over those with only local coverage, except those from countries where the GBD study provides subnational estimates. Data were excluded if the coverage population was unknown.

Bias of categories of input data

Cancer registry data can be biased in multiple ways. A high proportion of ill-defined cancer cases in the registry data requires redistribution of these cases to other cancers, which introduces a potential for bias. Changes between coding systems can lead to artificial differences in disease estimates; however, we adjust for this bias by mapping the different coding systems to the GBD causes. Underreporting of cancers that require advanced diagnostic techniques (e.g., leukemia and brain, pancreatic, and liver cancer) can be an issue in cancer registries from low-income countries. On the other hand, misclassification of metastatic sites as primary cancer can lead to overestimation of cancer sites that are common sites for metastases, like brain or liver. Since many cancer registries are located in urban areas, the representativeness of the registry for the general population can also be problematic. The accuracy of mortality data reported in cancer registries usually depends on the quality of the vital registration system. If the vital registration system is incomplete or of poor quality, the mortality-to-incidence ratio can be biased to lower ratios.

Data for liver cancer etiology splits

To find the proportion of liver cancer cases due to the four etiology groups included in GBD (1. Liver cancer due to hepatitis B, 2. Liver cancer due to hepatitis C, 3. Liver cancer due to alcohol, 4. Liver cancer due to other causes), a systematic literature search was performed in PubMed. Studies were included if the study population was representative of liver cancer population for the respective location. For each study the proportions of liver cancer due to the three specific risk factors were calculated. Remaining risk factors were included under a combined “other” group. Cryptogenic cases were only included if other etiologies like viral hepatitis or alcoholic cirrhosis had been excluded. If multiple risk factors were reported for an individual patient these were apportioned proportionally to the individual risk factors.

Methods

Steps of analysis and data transformation processes

Cancer registry data went through multiple processing steps before integration with the COD database. First, the original data were transformed into standardized files, which included standardization of format, categorization, and registry names (#1 in flowchart).

Second, some cancer registries report individual codes as well as aggregated totals (e.g., C18, C19, and C20 are reported individually but the aggregated group of C18-C20 [colorectal cancer] is also reported in

the registry data). The data processing step “subtotal recalculation” (#2 in flowchart) verifies these totals and subtracts the values of any individual codes from the aggregates.

In the third step (#3 in the flowchart), cancer registry incidence data and cancer registry mortality data are mapped to GBD causes. A different map is used for incidence and for mortality data because of the assumption that there are no deaths for certain cancers. One example is basal cell carcinoma of the skin. In the cancer registry incidence data, basal cell carcinoma is mapped to non-melanoma skin cancer (basal cell carcinoma). However, if basal cell skin cancer is recorded in the cancer registry mortality data, the deaths are instead mapped to non-melanoma skin cancer (squamous cell carcinoma) under the assumption that they were indeed misclassified squamous cell skin cancers. Other examples are benign or in situ neoplasms. Benign or in situ neoplasms found in the cancer registry incidence dataset were simply dropped from that dataset. The same neoplasms reported in a cancer registry mortality dataset were mapped to the respective invasive cancer (e.g., melanoma in situ in the cancer registry incidence dataset was dropped from the dataset; melanoma in situ in the cancer registry mortality dataset was mapped to melanoma).

In the fourth data processing step (#4 in the flowchart) cancer registry data were standardized to the GBD age groups. Age-specific incidence rates were generated using CI5, SEER, and NORDCAN data, while age-specific mortality rates were generated from the CoD data through a method described in Part 2. Age-specific proportions were then generated by applying the age-specific rates to a given registry population that required age-splitting to produce the expected number of cases/deaths for that registry by age. The expected number of cases/deaths for each sex, age, and cancer were then normalized to 1, creating final, age-specific proportions. These proportions were then applied to the total number of cases/deaths by sex and cancer to get the age-specific number of cases/deaths.

In the rare case that the cancer registry only contained data for both sexes combined, the now-age-specific cases/deaths were split and re-assigned to separate sexes using the same weights that are used for the age-splitting process. Starting from the expected number of deaths, proportions were generated by sex for each age (e.g., if for ages 15 to 19 years old there are six expected deaths for males and four expected deaths for females, then 60% of the combined-sex deaths for ages 15-19 years would be assigned to males and the remaining 40% would be assigned to females).

In the fifth step (#5 in the flowchart) data for cause entries that are aggregates of GBD causes were redistributed. Examples of these aggregated causes include some registries reporting ICD10 codes C00-C14 together as, “lip, oral cavity, and pharyngeal cancer.” These groups were broken down into sub-causes that could be mapped to single GBD causes. In this example, those include lip and oral cavity cancer (C00-C08), nasopharyngeal cancer (C11), cancer of other parts of the pharynx (C09-C10, C12-C13), and “Malignant neoplasm of other and ill-defined sites in the lip, oral cavity, and pharynx” (C14). To redistribute the data, weights were created using the same “rate-applied-to-population” method employed in age-sex splitting (see step four above). For the undefined code (C14 in the example) an “average all cancer” weight was used, which was generated by adding all cases from SEER/NORDCAN/CI5 and dividing the total by the combined population. Then, proportions were generated by sub-cause for each aggregate cause as in the sex-splitting example above (see step four). The total number of cases from the aggregated group (C00-C14) was then recalculated for each

subgroup and the undefined code (C14). C14 was then redistributed as a "garbage code" in step six. Distinct proportions were used for C44 (non-melanoma skin cancer) and C46 (Kaposi's sarcoma). Non-melanoma skin cancer processing is described under section "Input data and methodological summary for non-melanoma skin cancer (squamous-cell carcinoma)." C46 entries were redistributed as "other cancer," HIV, and C80 (other and unknown cancers) using proportions described in Part 2.
In the sixth step (#6 in the flowchart) unspecified codes ("garbage codes") were redistributed. Redistribution of cancer registry incidence and mortality data mirrored the process of the redistribution used in the cause of death database (Part 2).

In the seventh step (#7 in the flowchart) duplicate or redundant sources were removed from the processed cancer registry dataset. Duplicate sources were present if, for example, the cancer registry was part of the CI5 database but we also had data from the registry directly. Redundancies occurred and were removed as described in "Inclusion and Exclusion Criteria," where more detailed data were available, or when national registry data could replace regionally representative data. From here, two parallel selection processes were run to generate input data for the MI models and to generate incidence for final mortality estimation. Higher priority was given to registry data from the most standardized source when creating the final incidence input, whereas for the MI model input only sources that reported incidence and mortality were used. This is different to GBD 2015 where mortality and incidence could come from different sources as long as they covered the same population.
In the eighth step (#8 in the flowchart) the processed incidence and mortality data from cancer registries were matched by cancer, age, sex, year, and location to generate MI ratios. These MI ratios were used as input for a three-step modelling approach using the general GBD ST-GPR approach with SDI as a covariate in the linear step mixed effects model using a logit link function. Predictions were made without the random effects. The ST-GPR model has three main hyper-parameters that control for smoothing across time, age, and geography. The time adjustment parameter (λ) was set to 2, which aims to borrow strength from neighboring time points (i.e. the exposure in this year is highly correlated with exposure in the previous year but less so further back in time). The age adjustment parameter ω was set to 0.5, which borrows strength from data in neighboring age groups. The space adjustment parameter ξ was set to 0.95 in locations with data and to 0.5 in locations without data (the higher ξ was applied when at least one age-sex group in the country of estimation had at least five unique data points. The lower ξ was applied when estimating data-scarce countries). Zeta aims to borrow strength across the hierarchy of geographical locations.[10] For the amplitude parameter in the Gaussian process regression we used 2 and for the scale we used a value of 15.

As in GBD 2015 we have modified the approach to estimate MI ratios. Since for GBD 2015 MI ratio predictions for some cancers yielded similar predictions for low-SDI countries without data as for high-SDI countries we refined the estimation process. Inclusion criteria for the MI ratio input data were changed to only include mortality and incidence data if they were reported by the same source. We excluded MI ratios reported in the CI5[1,1–7] since mortality data used for the calculation of these MI ratios by definition has to be independent from the cancer registry. We also revised the outliering process and excluded data based on the SDI quintile categorization rather than on development status. For each cancer, MI ratios from locations in SDI quintiles 1-4 (low to high-middle SDI) were dropped if they were below the median of MI ratios from locations in SDI quintile 5 (high SDI). We also dropped MI ratios from locations in SDI quintiles 1-4 if the MI ratios were above the third quartile + 1.5 * IQR (inter-quartile range). We dropped all MIR that were based on less than 25 cases to avoid noise due to small

numbers except for mesothelioma and acute lymphoid leukemia where we dropped MIR that were based on less than 10 cases because of lower data availability for these two cancers. We also aggregated incidence and mortality to the youngest 5-year age bin where we had at least 50 data points to avoid MIR predictions in young age groups that were based on few data points. The MIR in the age-bin that was used to aggregate MIR to, was used to backfill the MIR for younger age groups.

Since MI ratios can be above 1, especially in older age groups and cancers with low cure rates, we used the 95th percentile of the cleaned dataset that only included MIR that were based on 50 or more cases, to cap the MIR input data. This "upper cap" was used to allow MIR over 1 but to constrain the MIR to a maximum level. To run the logit model, the input data was divided by the upper caps and model predictions after ST-GPR was rescaled by multiplying them by the upper caps.
Upper caps used for GBD 2016 were the following:

Age group	Maximum MIR
0-4	0.57
5-9	0.69
10-14	0.81
15-19	0.84
20-24	0.72
25-29	0.62
30-34	0.69
35-39	0.78
40-44	0.86
45-49	0.89
50-54	0.92
55-59	0.95
60-64	0.99
65-69	1.04
70-74	1.10
75-79	1.17
80+	1.32

To constrain the model at the lower end, we used the 5th percentile of the cancer specific cleaned MIR input data to replace all model predictions with this lower cap.

Final MI ratios were matched with the cancer registry incidence dataset in the ninth step (#9 in the flowchart) to generate mortality estimates (Incidence * Mortality/Incidence = Mortality) (#10 in the flowchart). The final mortality estimates were then uploaded into the COD database (#11 in the flowchart). Cancer-specific mortality modelling then followed the general CODEm process.

Liver cancer etiology split models

The proportion data found through the systematic literature review were used as input for four separate DisMod-MR 2.1 models to determine the proportion of liver cancers due to the four subgroups for all locations, both sexes, and all age groups (step #16 in the flowchart). A study covariate was used for

publications that only assessed liver cancer in a cirrhotic population. The reference or "gold standard" that was used for crosswalking was the compilation of all studies that assessed the etiology of liver cancer in a general population. For liver cancer due to hepatitis C and hepatitis B, a prior value of 0 was set between age 0 and 0.01. For liver cancer due to alcohol a prior value of 0 was set for ages 0 to 5 years. For liver cancer due to hepatitis C, hepatitis C (IgG) seroprevalence was used as a covariate as well as a covariate for alcohol (liters per capita) and hepatitis B prevalence (HBsAg seroprevalence), forcing a negative relationship between the alcohol and hepatitis B covariate and the outcome of liver cancer due to hepatitis C proportion. For liver cancer due to hepatitis B, seroprevalence of HBsAg was used as a covariate as well as a covariate for alcohol and hepatitis C IgG seroprevalence, forcing a negative relationship between the alcohol and hepatitis C covariate and the outcome of liver cancer due to hepatitis B proportion. For liver cancer due to alcohol, alcohol (liters per capita) was used as a covariate as well as a covariate for proportion of alcohol abstainers, hepatitis B and hepatitis C seroprevalence, forcing a negative relationship between the proportion of alcohol abstainers, hepatitis B and hepatitis C covariates and the outcome of liver cancer due to alcohol proportion. All covariates used were modelled independently. To ensure consistency between cirrhosis and liver cancer estimates and to take advantage of the data for the respective other related cause (e.g. liver cancer due to hepatitis C and the related cause cirrhosis due to hepatitis C), we generated covariates from the liver cancer proportion models that we used in the cirrhosis etiology proportion models. We then created covariates from the cirrhosis etiology proportion models and used those in the liver cancer etiology models.

Since the proportion models are run independently of each other, the final proportion models were scaled to sum to 100% within each age, sex, year, and location, by dividing each proportion by the sum of the four (step # 17). For the liver cancer subtype mortality estimates, we multiplied the parent cause "liver cancer" by the corresponding scaled proportions (step # 18). Single cause estimates were adjusted to fit into the separately modelled all-cause mortality in the process CoDCorrect.

Results

Interpretation of results

Cancer mortality estimates for GBD 2016 can differ from the GBD 2015 results for multiple reasons. Updated cancer mortality data were added from vital registration system data, verbal autopsy studies, as well as cancer registry incidence data. Mapping of cancer ICD codes to the GBD cancer causes was updated slightly based on collaborator comments. Mapping for the ICD10 code D46 (myelodysplastic syndrome) was changed back to "other cancer" as it had been in GBD 2013 based on collaborator comments and the consideration of adding myelodysplastic syndrome as a separate cause for future GBD iterations. To improve estimation of the leukemia sub-causes, a new cause, "leukemia other" was added since not all leukemia subtypes can be mapped the four most common types (acute and chronic lymphoid and myeloid leukemia). The mortality-to-incidence ratio estimation has changed compared to GBD 2015. Covariate inputs for the CODEm models were changed based on recommendations from collaborators. Covariates used in CODEm models were updated for GBD 2016.

The other group producing country-level cancer mortality estimates is the International Agency for Research on Cancer (IARC) with their GLOBOCAN database. Significantly different methods between the GBD study and GLOBOCAN can lead to differences in results. Whereas estimates in GLOBOCAN are based on the assumption that there are "In theory, […] as many methods as countries,"[11] the cancer estimation process for the GBD study follows a coherent, well-documented method for all cancers,

which allows cross-validation of models as well as determination of uncertainty. Another major difference is the ability in the GBD study to adjust single cause estimates to the all-cause mortality, which is being determined independently. This also allows us to adjust individual causes of death to the all-cause mortality envelope which permits us to correct for the underdiagnosis of cancer in countries with inadequate diagnostic resources. Redistribution of a fraction of undefined causes of death to certain cancers is another methodical advantage the GBD study has over GLOBOCAN, and estimates for cancer mortality can therefore differ substantially in countries with a large proportion of undefined causes of deaths in their vital registration data or a large proportion of undefined cancer cases in their cancer registry data.

Limitations

There are certain limitations to consider when interpreting the GBD mortality cancer estimates. First, even though every effort is made to include the most recently available data for each country, data-seeking resources are not limitless and new data cannot always be accessed as soon as they are made available. It is therefore possible that the GBD study does not include all available data sources for cancer incidence or cancer mortality. Second, different redistribution methods can potentially change the cancer estimates substantially if the data sources used for the estimated location contain a large number of undefined causes; however, neglecting to account for these undefined deaths would likely introduce an even greater bias in the disease estimates. Third, using mortality-to-incidence ratios to transform cancer registry incidence data to mortality estimates requires accurate MIR. For GBD 2016 we have made further changes to the MIR estimation, but the method remains sensitive to underdiagnosis of cancer cases or underascertainment of cancer deaths. However, given that the majority of data used for the cancer mortality estimation come from vital registration data and not cancer registry data this is not a major limitation.

Non-melanoma skin cancer (squamous-cell carcinoma)

Data

Data seeking processes

The input data were identified and processed using the same methods as all other cancers described above.

Inclusion and exclusion criteria

Inclusion and exclusion criteria followed the same methods as described for other cancers (see above).

Bias of categories of input data

The potential biases of the input data are the same as for other cancers (see above).

Methods

Overall methodological process

The GBD produces estimates for non-melanoma skin cancer via two subgroups: non-melanoma skin cancer (basal cell carcinoma) and non-melanoma skin cancer (squamous cell carcinoma). While some cancer registries report non-melanoma skin cancer at the four- or five- digit level required to distinguish between the subtypes (eg, “C44.01” versus “C44.02”, “173.01” versus “173.02), most registries report these cancers at the three-digit level as “C44” or “173” (“Other and unspecified malignant neoplasm of skin”). Because of this, those incident cases that were reported at this three-digit level were split to

“basal cell carcinoma” and “squamous cell carcinoma” based on proportions reported by Karagas et al during the cause disaggregation step (step #5 in the flowchart).[12] Since mortality estimates are produced for squamous cell carcinoma under the assumption that basal cell carcinoma causes almost no deaths, all mortalities reported as “C44” or “173” were mapped to the “squamous cell carcinoma” GBD cause. Apart from this additional step for some incident cases, the remainder of the cancer registry processing was the same as for other cancers as described above.

Steps of analysis and data transformation processes

Non-melanoma skin cancer (squamous cell carcinoma) mortality estimation followed the same steps as the other cancers (see flowchart and description above) except for step #5 in the flowchart as described above.

Model selection

The modelling strategy for non-melanoma skin cancer (squamous cell carcinoma) followed the general CODEm process.

Model performance and sensitivity

The modelling performance and sensitivity for non-melanoma skin cancer (squamous cell carcinoma) mirrored that of the general CODEm process.

Uncertainty intervals

Uncertainty was determined using standard CODEm methodology.

Results

Interpretation of results

Non-melanoma skin cancer mortality estimates are not available from other sources. GLOBOCAN, for example, does not report deaths due to non-melanoma skin cancer. Even though the data availability for non-melanoma skin cancer is poor, the fact that it is the most common incident cancer with rates expected to rise makes it a necessity to include the disease in the GBD framework.

Limitations

Cancer registry data for non-melanoma skin cancer incidence have to be interpreted with caution due to a substantial amount of underreporting or rules that only the first non-melanoma skin cancer has to be registered. Many cancer registries therefore do not include non-melanoma skin cancers at all. For vital registration data we make the assumption that there are no deaths due to non-melanoma skin cancer (basal cell carcinoma), therefore all deaths attributed to basal cell carcinoma were included instead as squamous cell carcinoma.

References

1 Waterhouse J, Muir C, Shanmugaratnam K, Powell J. Cancer Incidence in Five Continents IV. Lyon: IARC, 1982.

2 Curado M, Edwards B, Shin H, *et al.* Cancer Incidence in Five Continents IX. Lyon: IARC, 2007 http://www.iarc.fr/en/publications/pdfs-online/epi/sp160/CI5vol9-A.pdf.

3 Muir C, Mack T, Powell J, Whelan S. Cancer Incidence in Five Continents V. Lyon: IARC, 1987.

4 Parkin D, Muir C, Whelan S, Gao Y, Ferlay J, Powell J. Cancer Incidence in Five Continents VI. Lyon: IARC, 1992.

5 Parkin D, Whelan S, Ferlay J, Raymond L, Young J. Cancer Incidence in Five Continents VII. Lyon: IARC, 1997.

6 Parkin D, Whelan S, Ferlay J, Teppo L, Thomas D. Cancer Incidence in Five Continents VIII. Lyon: IARC, 2002.

7 Forman D, Bray F, Brewster D, *et al.* Cancer Incidence in Five Continents X. 2013. http://ci5.iarc.fr.

8 Engholm G, Ferlay J, Christensen N, *et al.* NORDCAN: Cancer Incidence, Mortality, Prevalence and Survival in the Nordic Countries, Version 7.3 Association of the Nordic Cancer Registries. Danish Cancer Society. 2016; published online Aug 7. http://www.ancr.nu.

9 Steliarova-Foucher E, O'Callaghan M, Ferlay J, Masuyer E, Forman D, Comber H, Bray F. European Cancer Observatory: Cancer Incidence, Mortality, Prevalence and Survival in Europe. Version 1.0 European Network of Cancer Registries, International Agency for Research on Cancer. 2012; published online Sept. http://eco.iarc.fr.

10 GBD 2015 Risk Factors Collaborators. Global, regional, and national comparative risk assessment of 79 behavioural, environmental and occupational, and metabolic risks or clusters of risks, 1990–2015: a systematic analysis for the Global Burden of Disease Study 2015. *The Lancet* 2016; **388**: 1659–724.

11 International Agency for Research on Cancer, World Health Organization. GLOBOCAN estimated cancer incidence, mortality, and prevalence worldwide in 2012. Lyon, France: IARC, 2014 http://globocan.iarc.fr/Default.aspx (accessed April 19, 2016).

12 Karagas MR, Greenberg ER, Spencer SK, Stukel TA, Mott LA. Increase in incidence rates of basal cell and squamous cell skin cancer in New Hampshire, USA. New Hampshire Skin Cancer Study Group. *Int J Cancer* 1999; **81**: 555–9.

Part 2 to supplementary appendix: Rheumatic heart disease

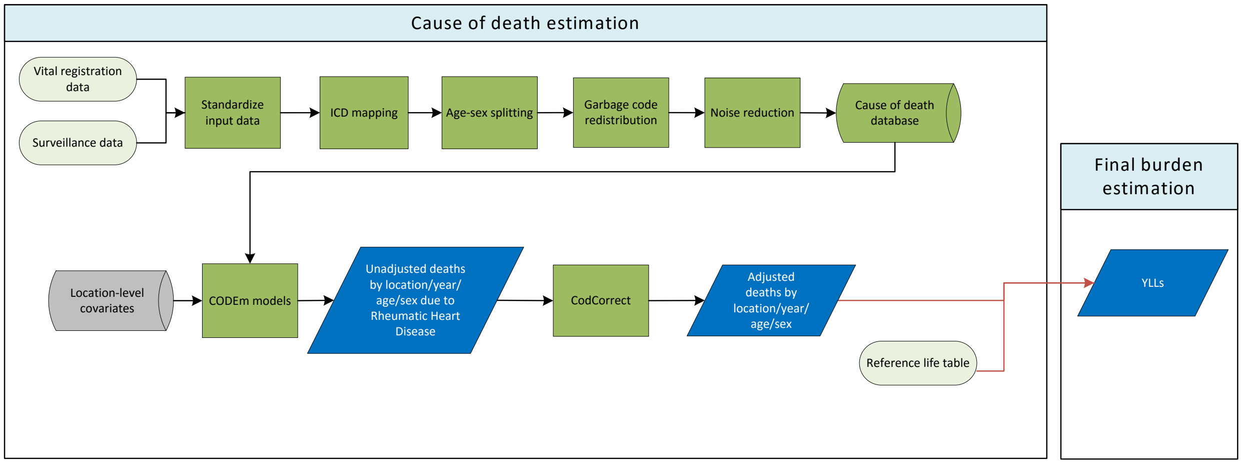

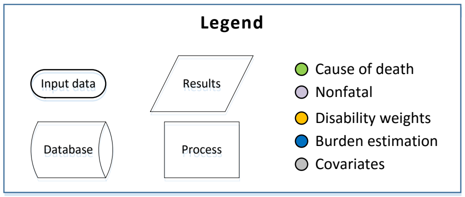

Input data

Vital registration and surveillance data were used to model rheumatic heart disease. We outliered ICD8 and ICD9 BTL data points which were inconsistent with the rest of the data and created implausible time trends. We also outliered data points which were too high after the redistribution process in a number of age groups.

Modelling strategy

We used a standard CODEm approach to model deaths from rheumatic heart disease. We have updated the covariates included in the ensemble modelling process (see Table). Otherwise, there have been no substantive changes from the approach used in GBD 2015.

Table: Selected covariates for CODEm models, rheumatic heart disease

Covariate	Transformation	Level	Direction
SEV	None	1	1
Improved water (proportion)	None	1	-1
Malnutrition	None	1	1
Sanitation (proportion with access)	None	1	-1
Healthcare access and quality index	None	2	-1
LDI	Log	3	-1
SDI	None	3	-1
Education (years per capita)	None	3	-1

IPart 2 to supplementary appendix: Ischemic heart disease

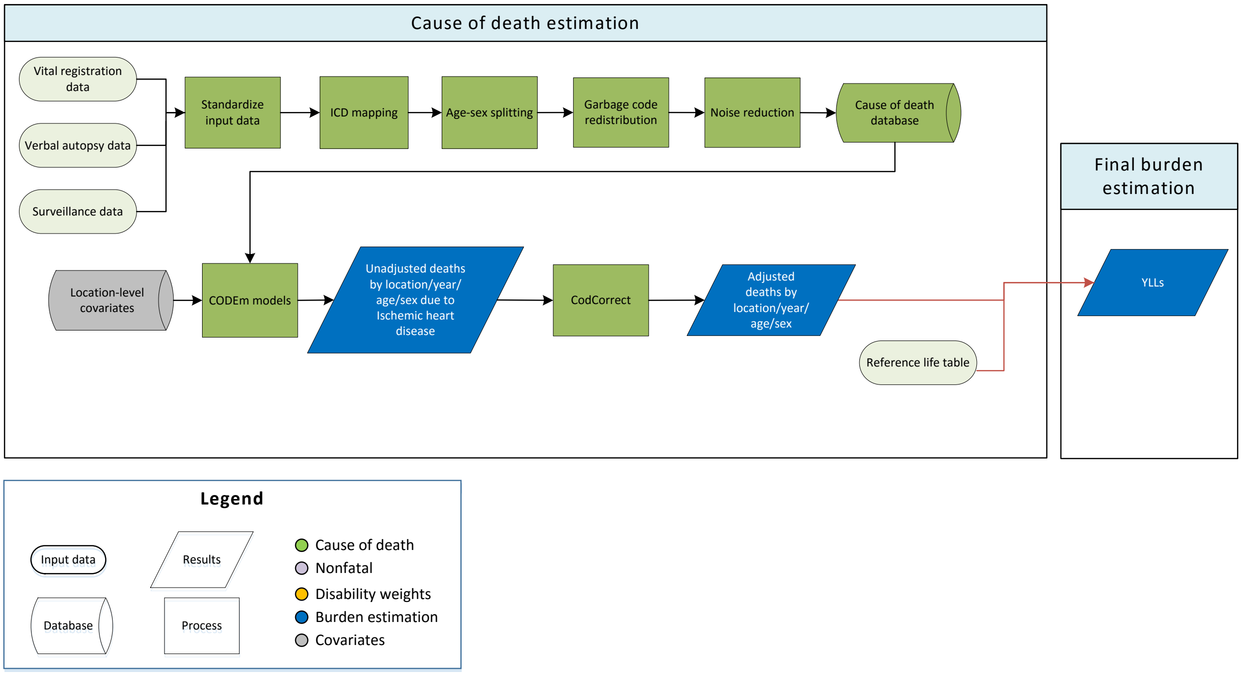

Input data

Vital registration, verbal autopsy, and surveillance data were used to model ischemic heart disease. We outliered verbal autopsy data in countries and subnational locations where high-quality vital registration data were also available. We also outliered non-representative subnational verbal autopsy data points, ICD8 and ICD9 BTL data points which were inconsistent with the rest of the data and created implausible time trends, and data in a number of Indian states identified by experts as poor-quality.

Modelling strategy

We used a standard CODEm approach to model deaths from ischemic heart disease. We have updated the covariates included in the ensemble modelling process (see Table). Otherwise, there have been no substantive changes from the approach used in GBD 2015.

Table: Selected covariates for CODEm models, ischemic heart disease

Covariate	Transformation	Level	Direction
Summary exposure variable	None	1	1
Cholesterol (total, mean per capita)	None	1	1
Smoking prevalence	None	1	1
Systolic blood pressure (mmHg)	None	1	1
Trans fatty acid	None	1	1
Mean BMI	None	2	1
Elevation over 1500m (proportion)	None	2	-1
Fasting plasma glucose	None	2	1
Outdoor pollution ($PM_{2.5}$)	None	2	1
Indoor air pollution	None	2	1
Healthcare access and quality index	None	2	-1
Lag distributed income per capita (I$)	Log	3	-1
Socio-demographic Index	None	3	0
Omega-3 (kcal/capita, adjusted)	Log	3	-1
Fruits (kcal/capita, adjusted)	None	3	-1
Vegetables (kcal/capita, adjusted)	None	3	-1
Nuts and seeds (kcal/capita, adjusted)	None	3	-1
Whole grains (kcal/capita, adjusted)	None	3	-1
Pulses/legumes (kcal/capita, adjusted)	None	3	-1
PUFA adjusted (percent)	None	3	-1
Alcohol (litres per capita)	None	3	0

Part 2 to supplementary appendix: Cerebrovascular disease

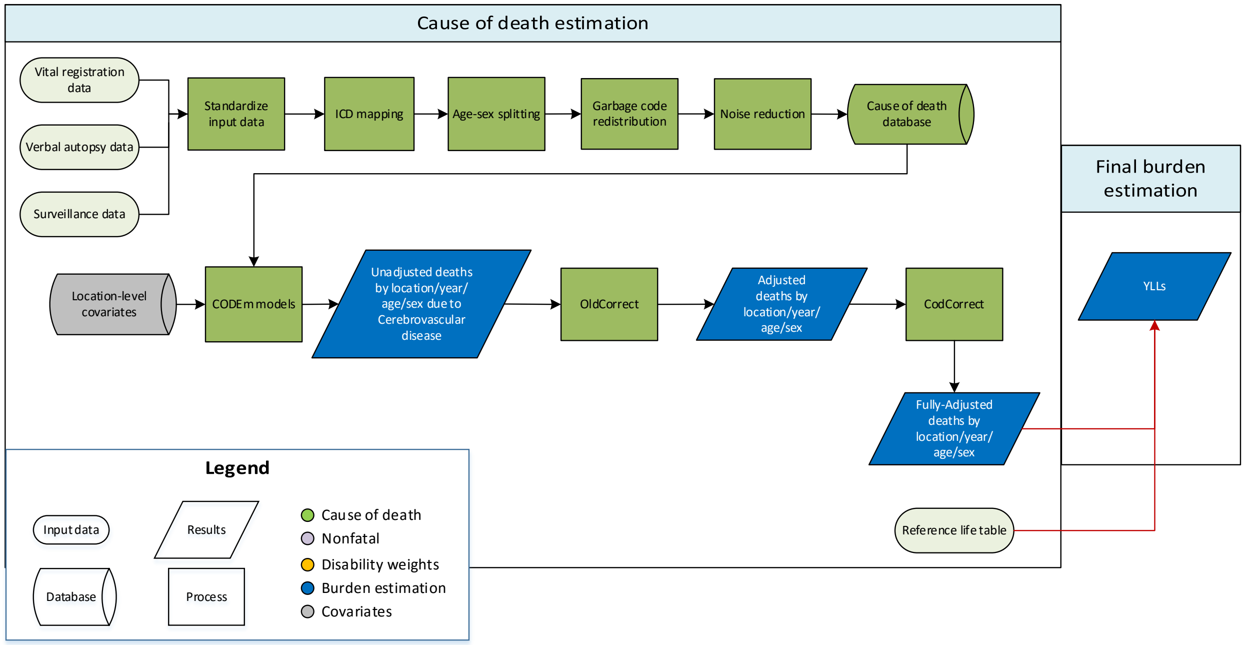

Input data

Verbal autopsy and vital registration data were used to model cerebrovascular disease. We outliered non-representative subnational verbal autopsy data points. We reassigned deaths from verbal autopsy reports for cerebrovascular disease to the parent cardiovascular disease for both sexes for those under 20 years of age. We also outliered ICD8, ICD9 BTL, and ICD10 Tabulated data points which were inconsistent with the rest of the data and created implausible time trends. Data points from sources which were implausibly low in all age groups and data points that were causing the regional estimates to be improbably high were outliered.

Modelling strategy

We used a standard CODEm approach to model deaths from cerebrovascular disease. The most significant update to the cerebrovascular method was the addition of a correction for miscoding of Alzheimer and other dementias and Parkinson disease to the post-CODEm adjustments to generate corrected cause-specific death estimates for final burden estimation. We have also updated the covariates included in the ensemble modelling process (see Table). Otherwise, there have been no substantive changes from the approach used in GBD 2015.

Table: Selected covariates for CODEm models, cerebrovascular disease

Covariate	Transformation	Level	Direction
Summary exposure variable	None	1	1
Cholesterol (total, mean per capita)	None	1	1
Smoking prevalence	None	1	1
Systolic blood pressure (mmHg)	None	1	1
Trans fatty acid	None	1	1
Mean BMI	None	2	1
Elevation over 1500m (proportion)	None	2	-1
Fasting plasma glucose	None	2	1
Outdoor pollution ($PM_{2.5}$)	None	2	1
Indoor air pollution	None	2	1
Healthcare access and quality index	None	2	-1
Lag distributed income per capita (I$)	Log	3	-1
Socio-demographic Index	None	3	0
Omega-3 (kcal/capita, adjusted)	Log	3	-1
Fruits (kcal/capita, adjusted)	None	3	-1
Vegetables (kcal/capita, adjusted)	None	3	-1
Nuts and seeds (kcal/capita, adjusted)	None	3	-1
Whole grains (kcal/capita, adjusted)	None	3	-1
Pulses/legumes (kcal/capita, adjusted)	None	3	-1
PUFA adjusted (percent)	None	3	-1
Alcohol (litres per capita)	None	3	0

Part 2 to supplementary appendix: Hypertensive heart disease

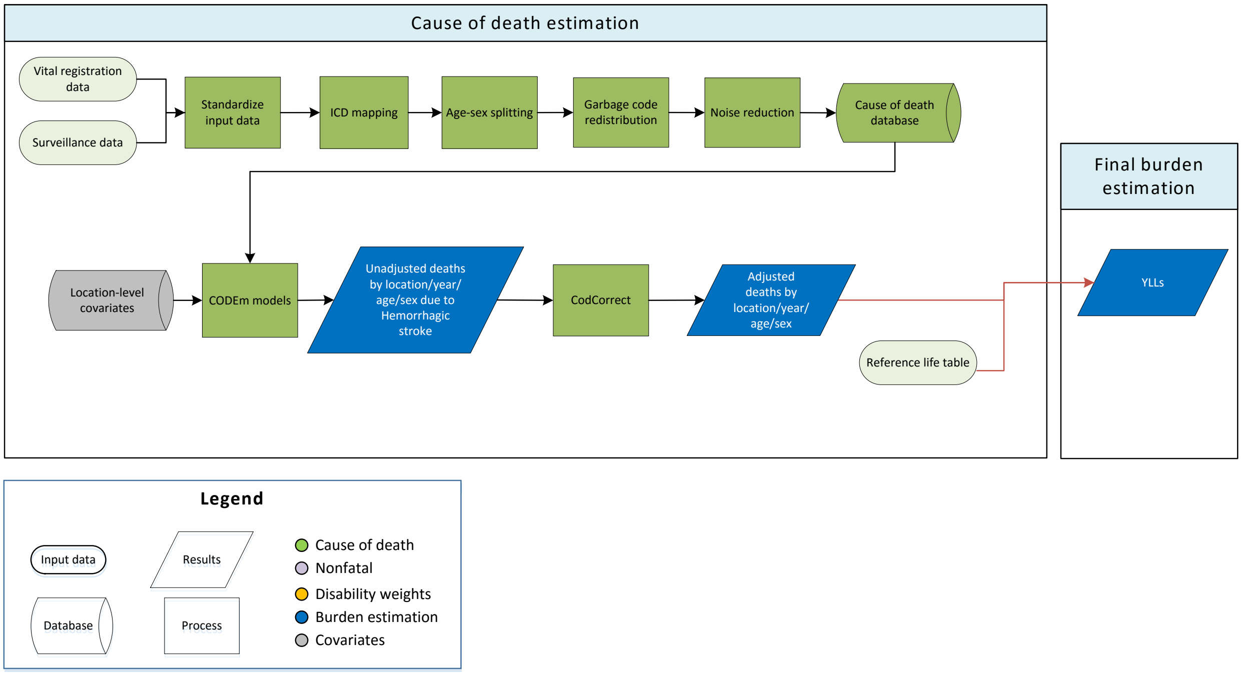

Input data

Vital registration and surveillance data were used to model hypertensive heart disease. We outliered ICD9 BTL data points, which were inconsistent with the rest of the data and created implausible time trends.

Modelling strategy

We used a standard CODEm approach to model deaths from cardiovascular diseases. We have updated the covariates included in the ensemble modelling process (see Table). Otherwise, there have been no substantive changes from the approach used in GBD 2015.

Table: Selected covariates for CODEm models, hypertensive heart disease

Covariate	Transformation	Level	Direction
Cholesterol (total, mean per capita)	None	1	1
Smoking prevalence	None	1	1
Systolic blood pressure (mmHg)	None	1	1
Mean BMI	None	2	1
Healthcare access and quality index	None	2	-1
Lag distributed income per capita (I$)	Log	3	-1
Socio-demographic index	None	3	0

Part 2 to supplementary appendix: Chronic respiratory diseases

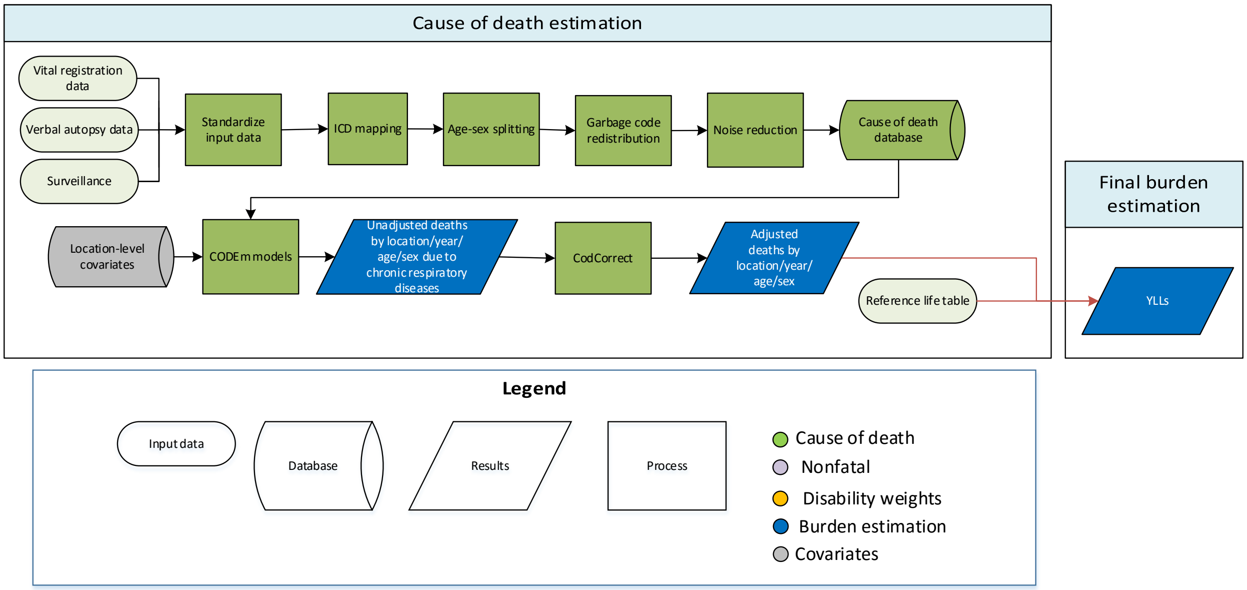

Input data

Sources used to estimate chronic respiratory disease mortality included vital registration, verbal autopsy, and surveillance data from China. Our outlier criteria excluded data points that (1) were implausibly high or low, (2) substantially conflicted with established age or temporal patterns, or (3) significantly conflicted with other data sources conducted from the same locations or locations with similar characteristics (ie, Socio-demographic Index).

Modelling strategy

The standard CODEm modelling approach was applied to estimate deaths due to chronic respiratory diseases. Chronic respiratory diseases served as the parent cause to chronic obstructive pulmonary disease, pneumoconiosis (including silicosis, asbestosis, coal worker's pneumoconiosis, other pneumoconiosis), asthma, interstitial lung disease and pulmonary sarcoidosis, and other chronic respiratory diseases. Functionally, this means the death estimates for Chronic Respiratory Diseases serve as a "parent" envelope into which the "child" causes are squeezed by the CodCorrect algorithm. This approach allows us to use a broader range of data – specifically verbal autopsy data – which cannot be accurately mapped to specific respiratory diseases.

Separate models were conducted for male and female mortality, and the age range for both models was 0 to 95+ years. The same covariates from GBD 2015 were used, with the exception of indoor air pollution, which was changed from cooking-fuel-specific covariates to a generic all cooking fuel covariate.

Level	Covariate	Direction
1	log-transformed SEV scalar: chronic respiratory diseases	+

	cumulative cigarettes (10 years)	+
	cumulative cigarettes (5 years)	+
	health care quality and access index	-
2	smoking prevalence	+
	indoor air pollution (all cooking fuels)	+
	outdoor air pollution ($PM_{2.5}$)	+
	population above 1500m elevation (proportion)	+
3	log LDI (I$ per capita)	-
	education (years per capita)	-
	Socio-demographic Index	-
	population between 500 and 1,500m elevation (proportion)	+
	population density over 1,000 people/square meter (proportion)	+

Beyond changes in the underlying covariates, there were no substantial deviations from the GBD 2015 approach.

Chronic Obstructive Pulmonary Disease

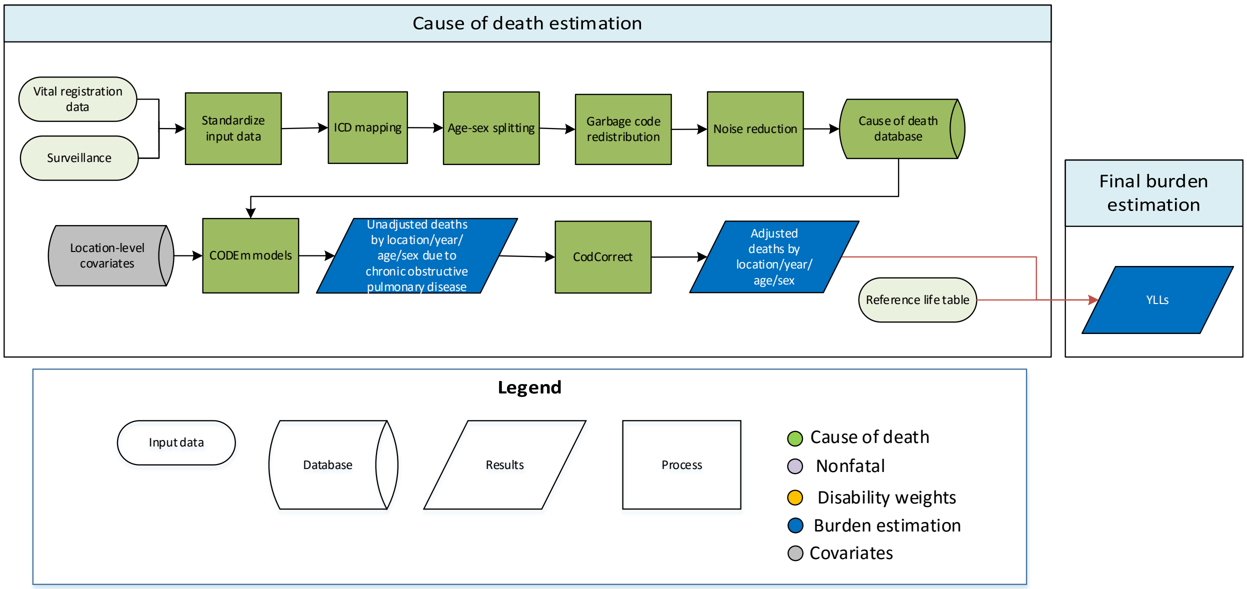

Input data

Data used to estimate chronic obstructive pulmonary disease (COPD) mortality included vital registration and surveillance data from the cause of death (COD) database. Our outlier criteria excluded data points that (1) were implausibly high or low, (2) substantially conflicted with established age or temporal patterns, or (3) significantly conflicted with other data sources conducted from the same locations or locations with similar characteristics (ie, Socio-demographic Index).

Modelling strategy

The standard CODEm modelling approach was applied to estimate deaths due to COPD. Separate models were conducted for male and female mortality, and the age range for both models was 1-95+ years. The mortality estimates from the COPD models were ultimately fit into the chronic respiratory diseases envelope.

The same covariates from GBD 2015 were used, with the exception of indoor air pollution, which was changed from cooking-fuel-specific covariates to a generic all cooking fuel covariate, and the health care access and quality index covariate, which was used in place of health systems access.

**Level**	**Covariate**	**Direction**
1	log-transformed SEV scalar: COPD	+
	cumulative cigarettes (10 years)	+
	cumulative cigarettes (5 years)	+
	elevation over 1,500m (proportion)	+

2	smoking prevalence	+
	indoor air pollution (all cooking fuels)	+
	outdoor air pollution ($PM_{2.5}$)	+
	health care access and quality index	-
3	Socio-demographic Index	-
	log LDI (I$ per capita)	-
	education (years per capita)	-

Pneumoconiosis diseases: Silicosis, asbestosis, coal worker's pneumoconiosis, and other pneumoconiosis

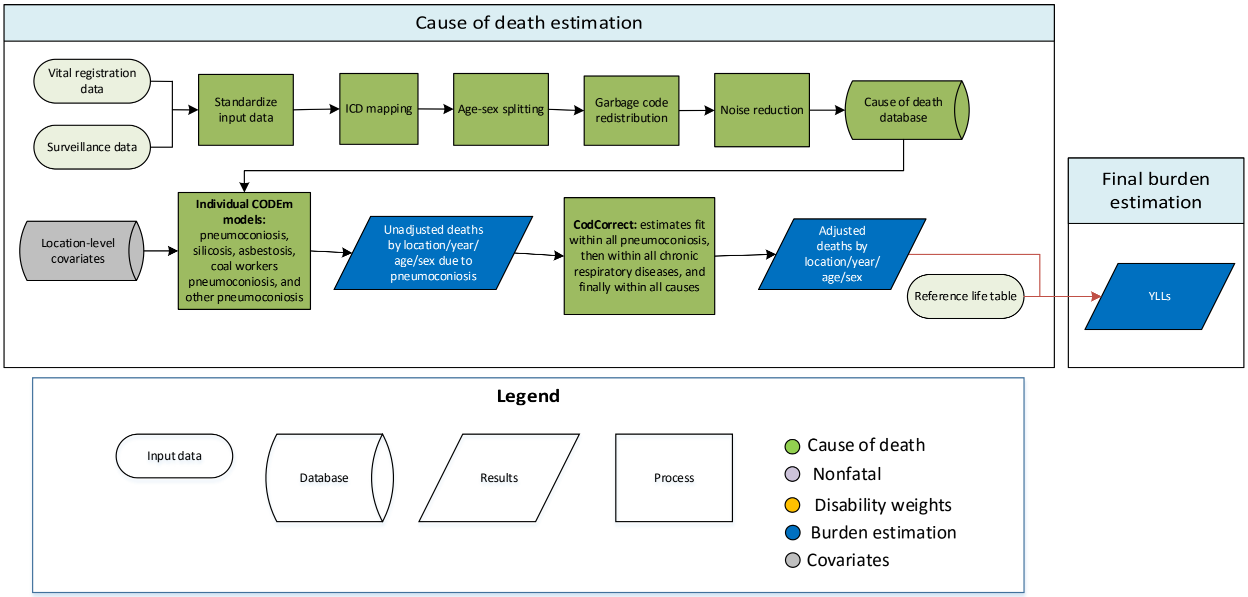

Input data

Data used to estimate pneumoconiosis diseases mortality included vital registration and China mortality surveillance data from the cause of death (COD) database. Our outlier criteria excluded data points that (1) were implausibly high or low, (2) substantially conflicted with established age or temporal patterns, or (3) significantly conflicted with other data sources conducted from the same locations or locations with similar characteristics (i.e., socio-demographic index).

Modelling strategy

The standard CODEm modelling approach was applied to estimate deaths due to pneumoconiosis diseases. Separate models were conducted for male and female mortality, and the age range for both models was 1–95+ years. The mortality estimates from pneumoconiosis disease models were ultimately fit into the chronic respiratory envelope, which is the parent cause for pneumoconiosis disease. The pneumoconiosis model serves as an envelope for silicosis, asbestosis, coal worker's pneumoconiosis, and other pneumoconiosis. In CoDCorrect, estimates are first fit within all pneumoconiosis, then within all chronic respiratory disease, before being fit to the all-cause mortality envelope.

For the most part, the same covariates from GBD 2015 were used. Indoor air pollution was changed from cooking-fuel specific covariates to a generic all cooking fuel covariate. Adjustments were also made to the coal and asbestos covariates.

The coal production covariate was improved to include subnational data for the United States and India. United States state-level data for 2001-2015 came from the U.S. Energy Information Administration. India state-level data for 2005-2014 came from the Ministry of Coal in India. We scaled these figures to the national estimates from the BP Statistical Review of World Energy 2016. For years with missing

state-level data we split the national-level data according to the proportions by state in the closest year for which we did have state-level data.

We also created a covariate for asbestos consumption per capita with a 30-year lag, and used that instead of the GBD 2015 asbestos production covariate. This change is based on the idea that asbestos production may be too limited in scope, given that asbestosis may occur in locations where asbestos is used and handled but not necessarily mined. To create the asbestos consumption covariate we used data from the United States Geological Survey to run a model in DisMod 2.1. A 30-year lag was placed on this model to account for the delay between asbestos consumption and occurrence of disease.

The following table indicates covariates used in the pneumoconiosis models, their level, and direction:

Level	Covariate	Direction
1	log-transformed SEV scalar: pneumoconiosis	+
	asbestos consumption per capita*	+
	coal production per capita*	+
	gold production per capita*	+
2	smoking prevalence	+
	indoor air pollution (all cooking fuels)	+
	cumulative cigarettes (5 years)	+
	elevation over 1,500m (proportion)	+
	elevation 500 to 1,500m (proportion)	+
	health care access and quality index	-
3	log LDI (I$ per capita)	-
	education (years per capita)	-
	Socio-demographic Index	-

* asbestos, coal, and gold covariates are each only used in a subset of the pneumoconiosis models, as follows: all three are included in the parent all pneumoconiosis model, asbestos consumption is included in the asbestosis model, coal production is included in the coal worker's pneumoconiosis model, and gold production is included in the silicosis model.

Asthma

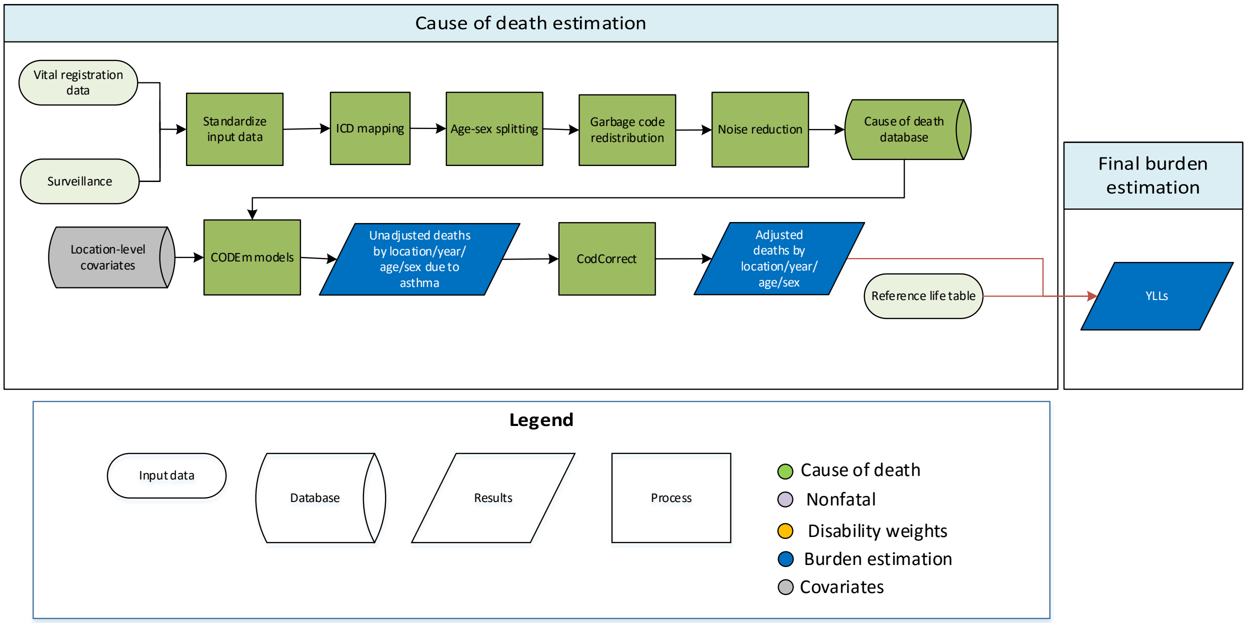

Input data

Data used to estimate asthma mortality included vital registration and surveillance data from the cause of death (COD) database. Verbal autopsy data were not included and were instead mapped to the parent model (Chronic Respiratory Diseases). Our outlier criteria excluded data points that (1) were implausibly high or low relative to global or regional patterns, (2) substantially conflicted with established age or temporal patterns, or (3) significantly conflicted with other data sources conducted from the same locations or locations with similar characteristics (ie, Socio-demographic Index).

Modelling strategy

The standard CODEm modelling approach was applied to estimate deaths due to asthma. Separate models were conducted for male and female mortality, and the age range for both models was 1–95+ years. The mortality estimates from the asthma models were ultimately fit into the chronic respiratory diseases envelope.

The same covariates from GBD 2015 were used, with the exception of indoor air pollution, which was changed from cooking-fuel-specific covariates to a generic all cooking fuel covariate.

Level	Covariate	Direction
1	log-transformed SEV scalar: asthma	+
	cumulative cigarettes (10 years)	+
	cumulative cigarettes (5 years)	+

	health care access and quality index	-
2	smoking prevalence	+
	indoor air pollution (all cooking fuels)	+
	outdoor air pollution ($PM_{2.5}$)	+
3	log LDI (I$ per capita)	-
	education (years per capita)	-
	Socio-demographic Index	-

Interstitial lung disease and pulmonary sarcoidosis

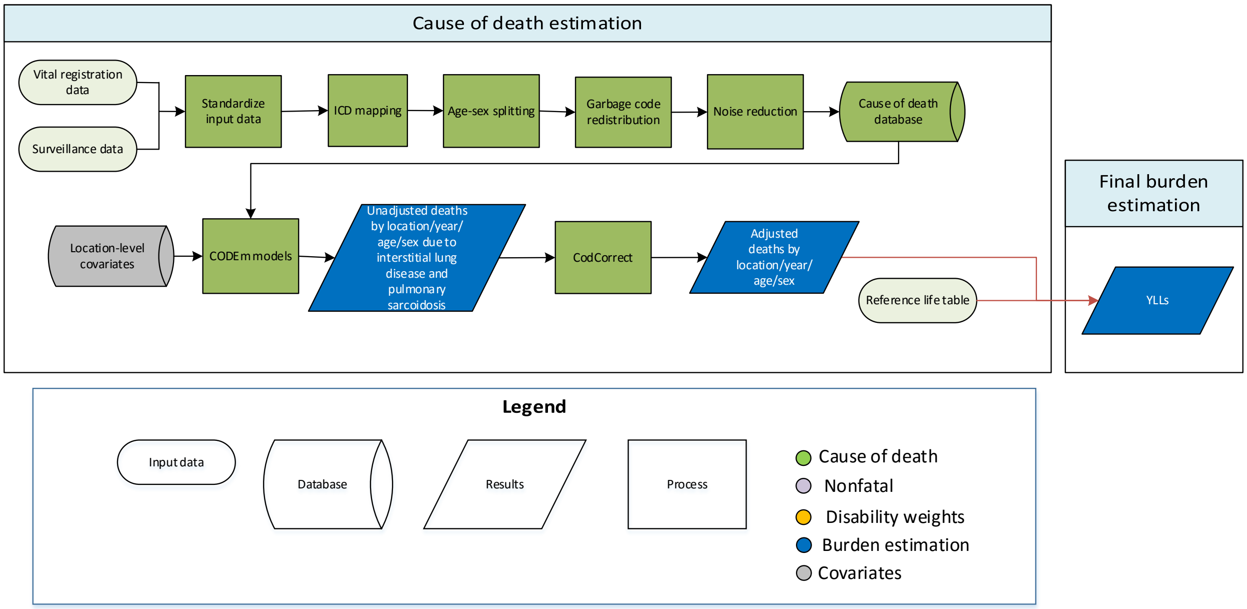

Input data

Data used to estimate interstitial lung disease and pulmonary sarcoidosis mortality included vital registration and surveillance data from the cause of death (COD) database. Our outlier criteria excluded data points that (1) were implausibly high or low, (2) substantially conflicted with established age or temporal patterns, or (3) significantly conflicted with other data sources conducted from the same locations or locations with similar characteristics (ie, Socio-demographic Index).

Modelling strategy

The standard CODEm modelling approach was applied to estimate deaths due to interstitial lung disease and pulmonary sarcoidosis. Separate models were conducted for male and female mortality, and the age range for both models was 1–95+ years. The mortality estimates from the interstitial lung disease and pulmonary sarcoidosis models were ultimately fit into the chronic respiratory envelope.

The same covariates from GBD 2015 were used, with the exception of indoor air pollution, which was changed from cooking-fuel-specific covariates to a generic all cooking fuel covariate.

Level	Covariate	Direction
1	log-transformed SEV scalar: interstitial lung disease	+
	smoking prevalence	+
	cumulative cigarettes (5 years)	+
2	elevation over 1,500m (proportion)	+

	elevation between 500 and 1,500m (proportion)	+
	population density over 1,000 ppl/sqkm (proportion)	+
	indoor air pollution (all cooking fuels)	+
	outdoor air pollution ($PM_{2.5}$)	+
	health care access and quality index	-
3	log LDI (I$ per capita)	-
	education (years per capita)	-
	Socio-demographic Index	-

Other chronic respiratory diseases

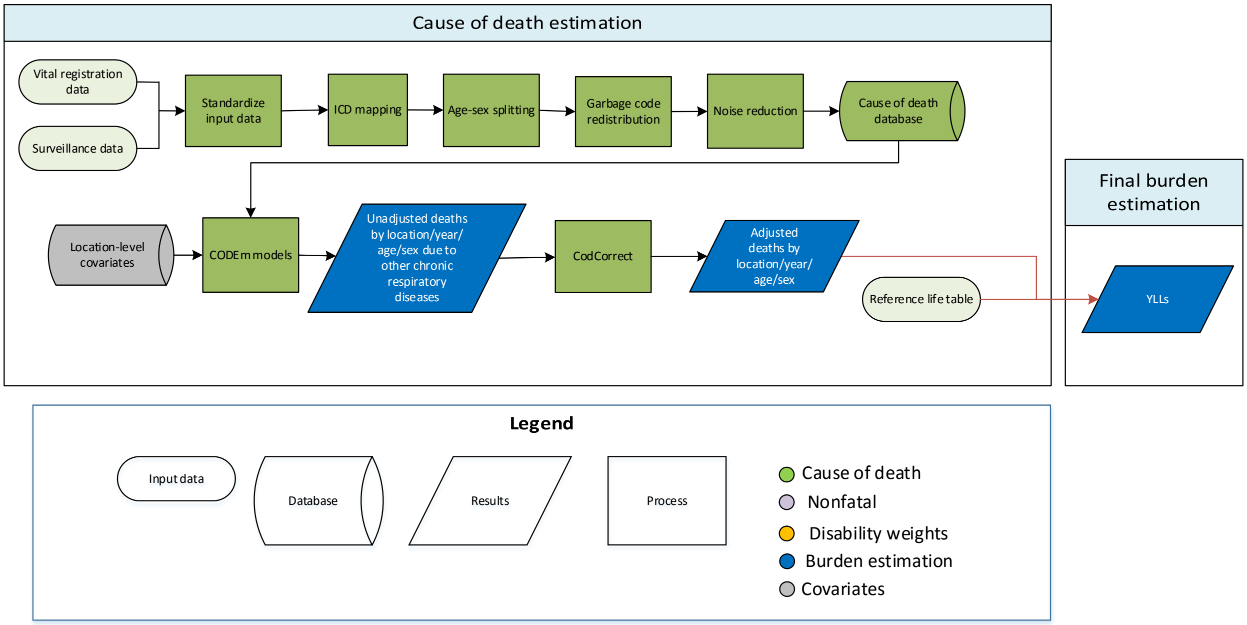

Input data

Data used to estimate other chronic respiratory diseases included vital registration and surveillance data from the cause of death (COD) database. Our outlier criteria excluded data points that (1) were implausibly high or low, (2) substantially conflicted with established age or temporal patterns, or (3) significantly conflicted with other data sources conducted from the same locations or locations with similar characteristics (ie, Socio-demographic Index).

Modelling strategy

The standard CODEm modelling approach was applied to estimate deaths due to other chronic respiratory diseases. Separate models were conducted for male and female mortality, and the age range for both models was 0 days to 95+ years. Like other respiratory causes, the mortality estimates from other chronic respiratory diseases were ultimately fit into the chronic respiratory envelope.

The same covariates from GBD 2015 were used, with the exception of indoor air pollution, which was changed from cooking-fuel-specific covariates to a generic all cooking fuel covariate.

Level	Covariate	Direction
1	log-transformed SEV scalar: other chronic respiratory diseases	+
	smoking prevalence	+
	cumulative cigarettes (5 years)	+
	indoor air pollution (all cooking fuels)	+

	outdoor air pollution ($PM_{2.5}$)	+
2	elevation over 1,500m (proportion)	+
	elevation between 500 and 1,500m (proportion)	+
	population density over 1,000 ppl/sqkm (proportion)	+
	health care access and quality index	-
3	log LDI (I$ per capita)	-
	education (years per capita)	-
	Socio-demographic Index	-

Part 2 to supplementary appendix: Peptic ulcer disease

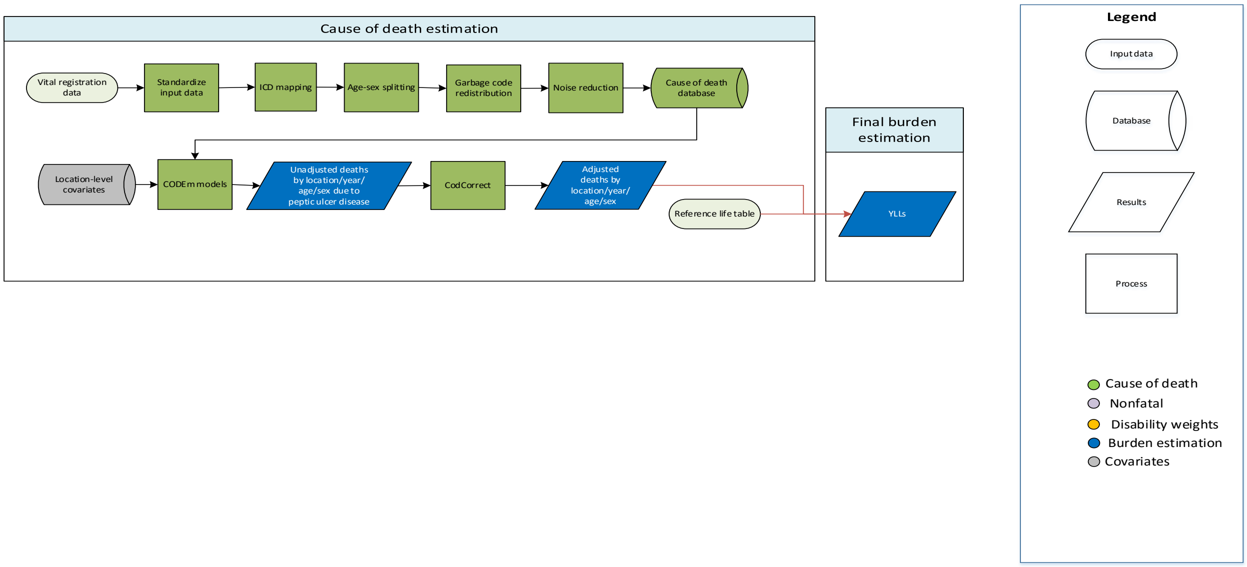

Input data

Data used to estimate mortality of peptic ulcer disease consisted of vital registration data from the cause of death (COD) database. We outliered data in instances where garbage code redistribution and noise reduction, in combination with small sample sizes, resulted in unreasonable cause fractions, and data that violated well-established time or age trends.

Modelling strategy

We modelled deaths due to peptic ulcer disease with a standard CODEm model using the cause of death database and location-level covariates as inputs. The model followed standard parameters, with the exception that the start age of the model was 1 year old instead of 0. We hybridized separate global and data-rich models to acquire unadjusted results, which we finalized and adjusted using CodCorrect to reach final years of life lost (YLLs) due to peptic ulcer disease. The covariate changes from GBD 2015 to GBD 2016 include changing the directionality of vegetables adjusted (grams per person availability) from -1 to 0, the addition of the summary exposure variable unsafe water, and the addition of the healthcare access and quality index (HAQI) covariate.

Covariate	Level	Direction
Alcohol (liters per capita)	1	1
Cumulative cigarettes (10 years)	1	1
Cumulative cigarettes (5 years)	1	1
Lag distributed income (per capita)	3	-1
Sanitation (proportion with access)	2	-1
Smoking (prevalence)	1	1
Maternal education (years per capita)	3	-1
Improved water source (proportion with access)	2	1
Sociodemographic index	3	-1
Vegetables (grams adjusted)	2	0
Health access and quality index	2	-1

Part 2 to supplementary appendix: Appendicitis

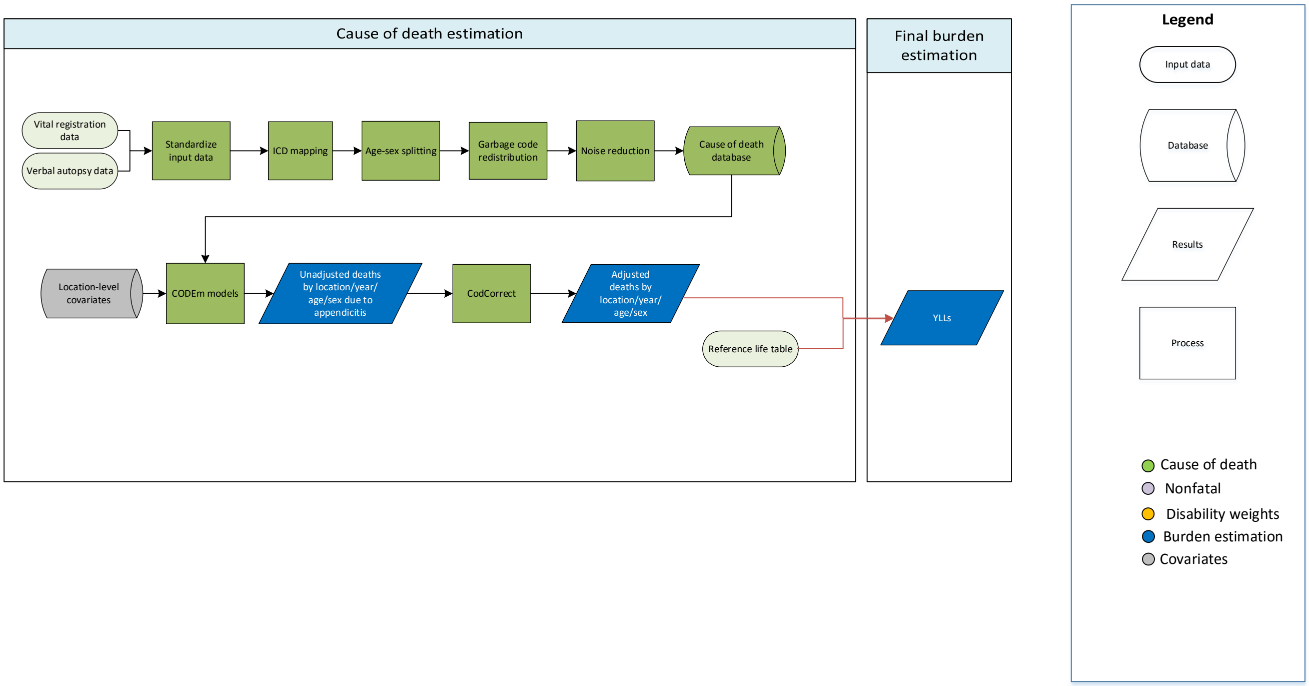

Input data

Data used to estimate appendicitis mortality consisted of vital registration and verbal autopsy data from the cause of death (COD) database. We outliered data in instances where garbage code redistribution and noise reduction, in combination with small sample sizes, resulted in unreasonable cause fractions; and data that violated well-established time or age trends.

Modelling strategy

We modelled deaths due to appendicitis with a standard CODEm model using the cause of death database and location-level covariates as inputs. The model followed standard parameters, with the exception that the start age of the model was 1 year old instead of 0 and the linear floor rate was lowered to 0.0001 in order to better capture low data. We hybridized separate global and data-rich models to acquire unadjusted results, which we finalized and adjusted using CodCorrect to reach final YLLs due to appendicitis.

There were no significant changes in the modelling process between GBD 2015 and GBD 2016.

Covariate	Level	Direction
Education (years per capita)	3	-1
Log LDI (I$ per capita)	3	-1
Health system access (capped)	3	-1
Socio-demographic Index	3	-1
Fruits adjusted (g)	2	-1
Vegetables adjusted (g)	2	-1
Healthcare access and quality index	2	-1

Part 2 to supplementary appendix: Inguinal, femoral, and abdominal hernias

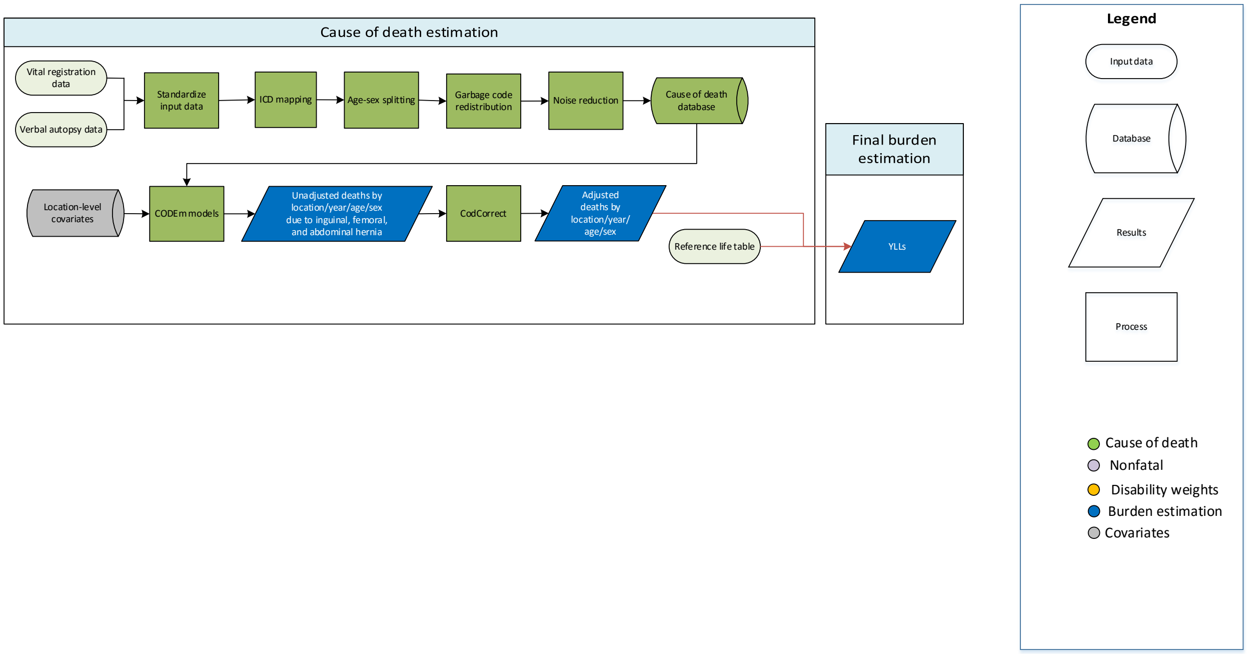

Input data

Vital registration and verbal autopsy data were used to model this cause. We outliered data in instances where garbage code redistribution and noise reduction, in combination with small sample sizes, resulted in unreasonable cause fractions; and data that violated well-established time or age trends. Outliering methods were consistent across both vital registration and verbal autopsy data.

Modelling strategy

We modelled deaths due to inguinal, femoral, and abdominal hernias with a standard CODEm model using the cause of death database and location-level covariates as inputs. The model followed standard parameters, with the exception that the start age of the model was 1 year old instead of 0 and the linear floor rate was lowered to 0.0001 in order to better capture low data. We hybridized separate global and data-rich models to acquire unadjusted results, which we finalized and adjusted using CodCorrect to reach final years of life lost (YLLs) due to inguinal, femoral, and abdominal hernias. In GBD 2016 we added the healthcare access and quality index (HAQI) covariate to the model.

Covariate	Level	Direction
Education (years per capita)	3	-1
Lag distributed income (per capita)	3	-1
Sociodemographic index	3	0
Health access and quality index	2	-1

Part 2 to supplementary appendix: Gallbladder and biliary diseases

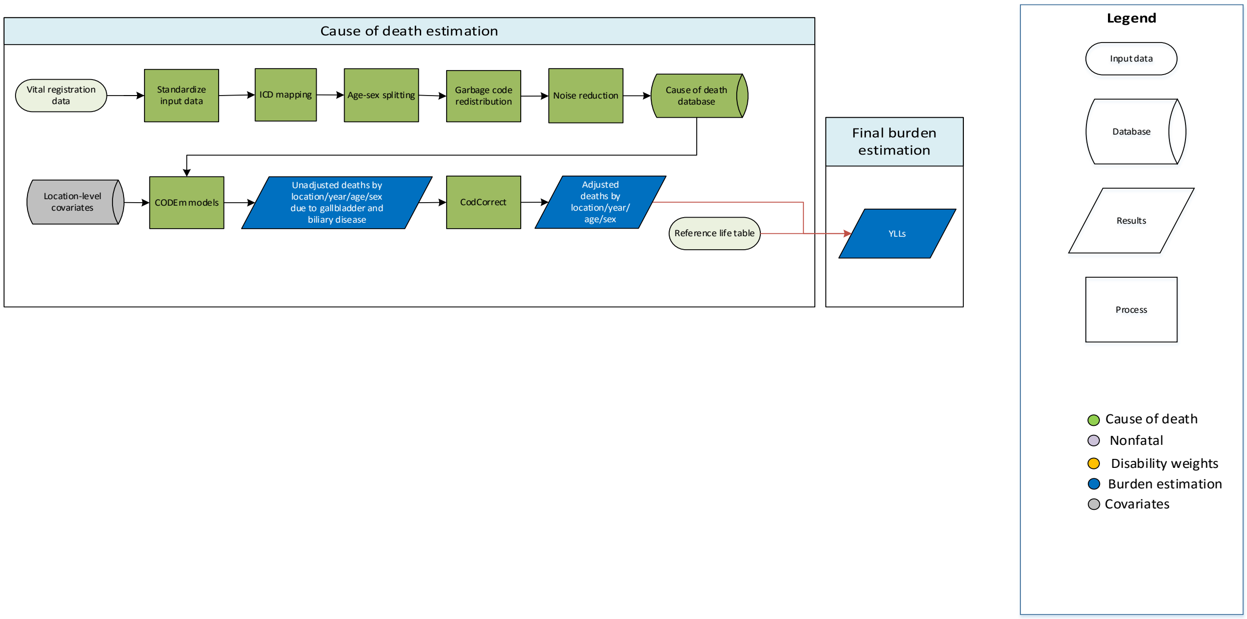

Input data

Data used to estimate mortality of gallbladder and biliary diseases consisted of vital registration data from the cause of death (COD) database. We outliered data in instances where garbage code redistribution and noise reduction, in combination with small sample sizes, resulted in unreasonable cause fractions; and data that violated well-established time or age trends.

Modelling strategy

We modelled deaths due to gallbladder and biliary diseases with a standard CODEm model using the cause of death database and location-level covariates as inputs. The model followed standard parameters, with the exception that the start age of the model was 1 year old instead of 0 and the linear floor rate was lowered to 0.0001 in order to better capture low data. We hybridized separate global and data-rich models to acquire unadjusted results, which we finalized and adjusted using CodCorrect to reach final years of life lost (YLLs) due to gallbladder and biliary diseases. In GBD 2016 we added the healthcare access and quality index (HAQI) covariate and replaced the animal fats (kcal per capita) covariate with an updated saturated fats (adjusted percent).

Covariate	Level	Direction
Alcohol (liters per capita)	2	1
Education (years per capita)	3	0
Lag distributed income (per capita)	3	0
Body mass index (mean)	1	1
Population over 65 (proportion)	2	1
Sociodemographic index	3	0
Red meats (grams adjusted)	2	1
Saturated fats (adjusted percent)	1	1
Health access and quality index	2	-1

Part 2 to supplementary appendix: Epilepsy

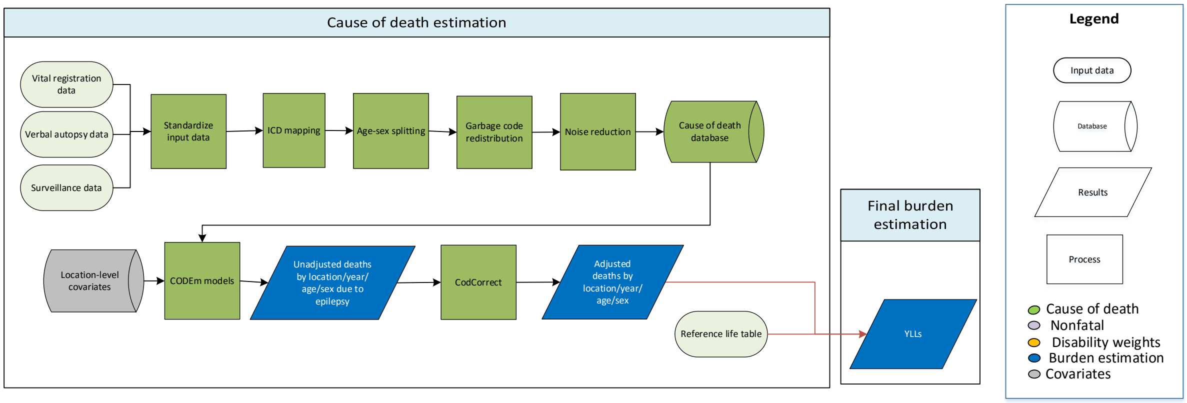

Input data

Data used to estimate epilepsy mortality included vital registration (VR), verbal autopsy, and China mortality surveillance data from the cause of death (COD) database. Our outlier criteria were to exclude data points that were (1) implausibly high or low relative to global or regional patterns, (2) substantially conflicted with established age or temporal patterns, or (3) significantly conflicted with other data sources based from the same locations or locations with similar characteristics (i.e., socio-demographic index).

Based on these criteria, we excluded ICD-9 BTL data for Sri Lanka, Fiji, and Kiribati as the estimates varied from year to year between zero and high values. We also excluded the Survey of Causes of Death Data and Medical Certification of Cause of Death Data for India, as these data types were not consistent with the Sample Registration System Data and would have led to discontinuities in our estimates over time.

Modelling strategy

The standard CODEm modelling approach was applied to estimate deaths due to epilepsy. Separate models were conducted for male and female mortality, and the age range for both models was 28 days–95+ years. For GBD 2016, the health systems access covariate was replaced with the health access and quality index covariate. There were no other substantial changes for GBD 2016. The covariates used are displayed below.

Level	Covariate	Direction
1	pig meat consumption (kcal per capita)	+
	pigs (per capita)	+
	SEV scalar: epilepsy	+
	mean systolic blood pressure (mmHg)	+
2	health access and quality index	-
	mean body mass index	+
	mean serum total cholesterol (mmol/L)	+

3	cumulative cigarettes (10 years)	+
	cumulative cigarettes (5 years)	+
	education (years per capita)	-
	log LDI (per capita)	-
	Socio-demographic Index	-

Part 2 to supplementary appendix: Diabetes mellitus

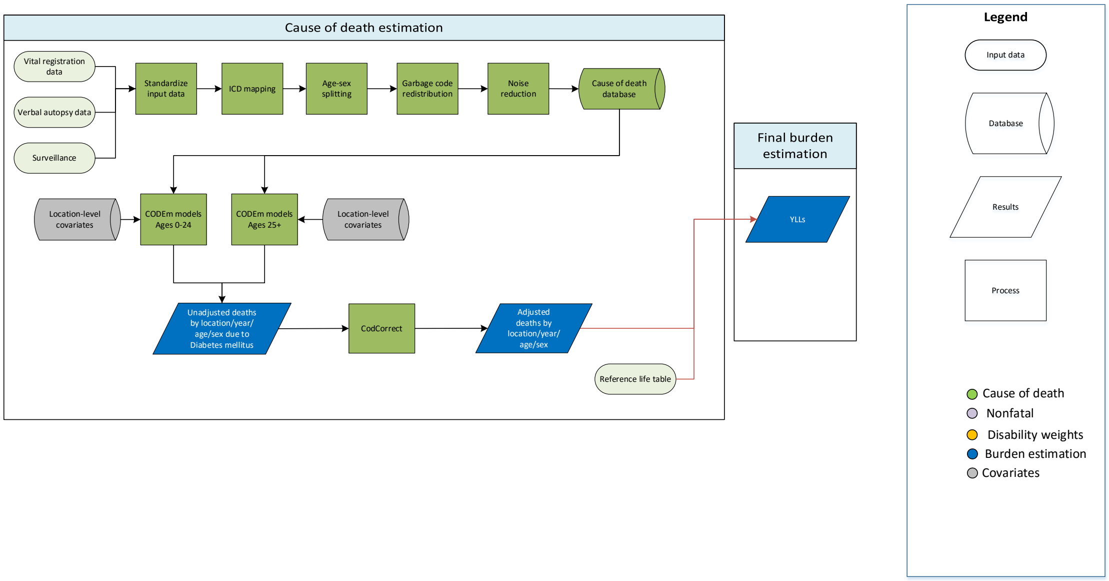

Input data

Verbal Autopsy Data: We outliered VA data points in urban Indian states where high-quality vital registration data were also available. We also outliered data points where the VA data were implausible in all age groups as we determined that these data sources were unreliable.

Vital Registration Data: We outliered all data in four urban Indian states where the source of the data was unreliable according to expert opinion. We also outliered ICD9BTL data points which were inconsistent with the rest of the data series and created unlikely time trends.

Modeling strategy

We used a slight variation on the standard CODEm approach to model deaths from diabetes mellitus. Since deaths in younger age groups are almost exclusively due to Type 1 diabetes while deaths in older ages are primarily due to Type 2, we used two models to estimate overall diabetes deaths. We reviewed the cause-fraction of deaths due to Type 1 and Type 2 diabetes at the global, super region, and regional level. We selected a conservative estimate of 25 years; one model is for deaths in 0-25 year olds and the second model is for deaths in 25+ year olds.

The following list are the covariates included in the model.

- Education years per-capita
- A composite score that approximates access to and quality of personal healthcare (Healthcare Access and Quality Index)
- Lag distributed GDP per capita in base 2010 international dollars
- Estimated national availability of animal fat expressed as kilocalories per capita
- Mean diabetes fasting plasma glucose (mmol/L) by age group
- Age-standardized prevalence of diabetes

- Age-standardized mean body mass index for adults ages 20+ (separate by sex)
- Mean serum total cholesterol (mmol/L) for individuals above age 25
- Mean systolic blood pressure (mmHg) for individuals above age 25
- Estimated energy adjusted national availability of fruits expressed in grams per person per day
- Estimated energy adjusted national availability of vegetables expressed in grams per person per day
- Estimated energy adjusted national availability of whole grains expressed in grams per person per day
- Estimated national availability of dietary energy expressed in kilocalories per person per day

Part 2 to supplementary appendix: Chronic kidney disease

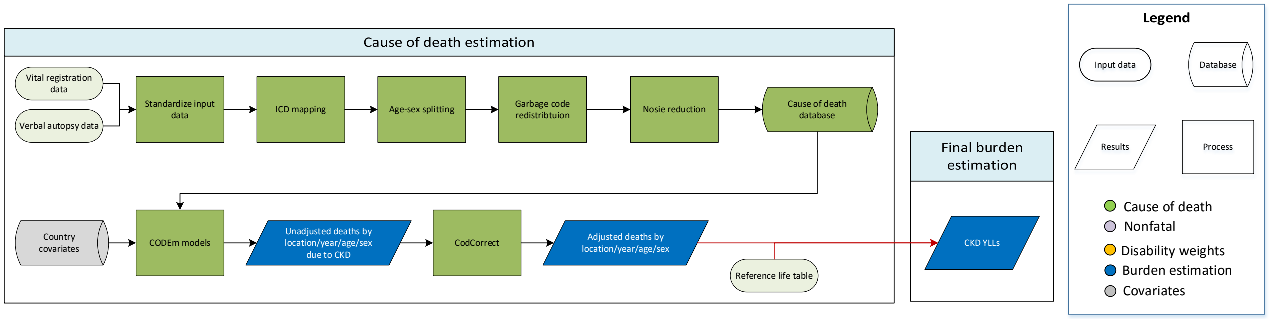

Input data

Vital registration and verbal autopsy data were used to model mortality due to urolithiasis. Outliers were identified by systematic examination of data points for all location-years. Data were standardised and mapped according to the GBD causes of death ICD mapping method. These data were then age-sex split, and appropriate redistribution of garbage code data was performed. Data points that violated well-established age or time trends or that resulted in extremely high or low cause fractions were determined to be outliers. For GBD 2016, deaths due to congenital kidney anomalies (cystic kidney disease and reflux hydronephrosis) were attributed to chronic kidney disease, marking a change from GBD 2015 when these deaths were assigned to congenital anomalies.

Modelling strategy

The estimation strategy used for fatal chronic kidney disease is largely similar to methods used in GBD 2015. A standard CODEm model with location-level was used to model deaths due to chronic kidney disease. Iterations of models were assessed at the location/year/age-group/sex level to determine whether data points merited exclusion via outliering. Unadjusted death estimates were adjusted using CoDCorrect to produce final estimates of YLLs. The covariates used are displayed below.

Level	Covariate	Direction
	Diabetes fasting plasma glucose (mmol/L)	+
	Diabetes age-standardized prevalence (proportion)	+
1	Mean systolic blood pressure (mmHg)	+
	Mean BMI	+
	Health care access and quality index	–
	Mean cholesterol	+
	Total calories (kcal per capita)	–
2	Red meat (kcal per capita)	0
	Whole grains (kcal per capita)	0
	Animal fat (kcal per capita)	0
	Socio-demographic Index	0
3	Education (years per capita)	–
	Log LDI ($I per capita)	–

Chronic kidney disease subtypes

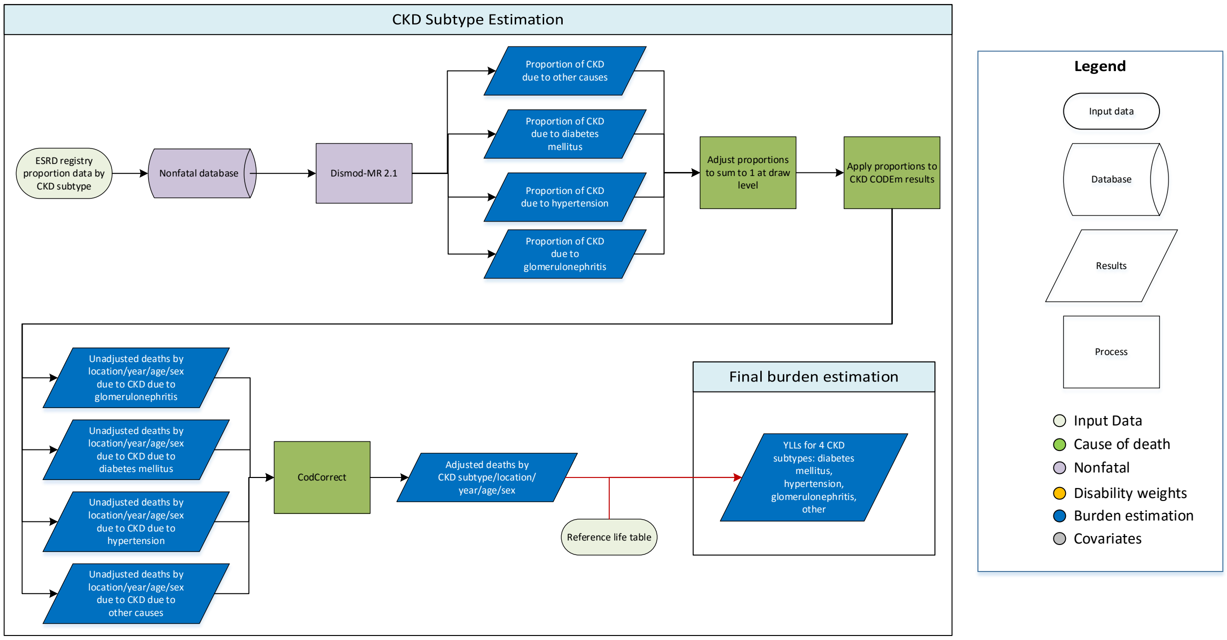

Input data

The estimation strategy for CKD subtypes of 1) diabetes mellitus, 2) hypertension, 3) glomerulonephritis, and 4) “other” has changed significantly from the GBD 2015 analysis to achieve consistency of method among the four subtypes. This improved method is detailed below.

Data from end-stage renal disease registries were used to inform estimates of proportion of CKD mortality attributable to each CKD subtype. These data were age-split using the age pattern obtained from the Australia & New Zealand Dialysis and Transplant Registry (ANZDATA) which provides age and subtype-specific data. The age-pattern was determined by calculating the number of cases of CKD by etiology over the total number of cases for all etiologies for each 5-year age group. Then, aggregate-age proportions were split using the age-specific prevalence proportions and rescaled to sum to 1 within each 5-year age group.

Vital registration (VR) data were excluded from estimates as etiology coding in VR sources was considered highly variable and inconsistent between countries.

Modelling strategy

We ran DisMod-MR 2.1 models including diabetes prevalence and mean systolic blood pressure as country-level covariates to obtain estimates of proportions for each subtype by location, year, age, and sex. The results from these models were adjusted so that estimates across the subtypes equaled 1 at each of 1,000 draws. These adjusted proportions were applied to the parent CKD CODEm model.

Model	Covariate	Value	Exponentiated
CKD proportion due to diabetes mellitus	Diabetes age-standardized prevalence	0.36<br>(0.29 – 0.42)	1.43<br>(1.34 – 1.53)
CKD proportion due to hypertension	Mean systolic blood pressure	0.013<br>(0.00036 – 0.043)	1.01<br>(1.00 – 1.04)

Part 2 to supplementary appendix: Congenital birth defects, including congenital heart anomalies

Neural tube defects, Congenital heart anomalies, Orofacial clefts, Down Syndrome, Turner syndrome, Klinefelter syndrome, Other chromosomal disorders, Congenital musculoskeletal anomalies, Urogenital congenital anomalies, Digestive congenital anomalies, and Other congenital birth defects.

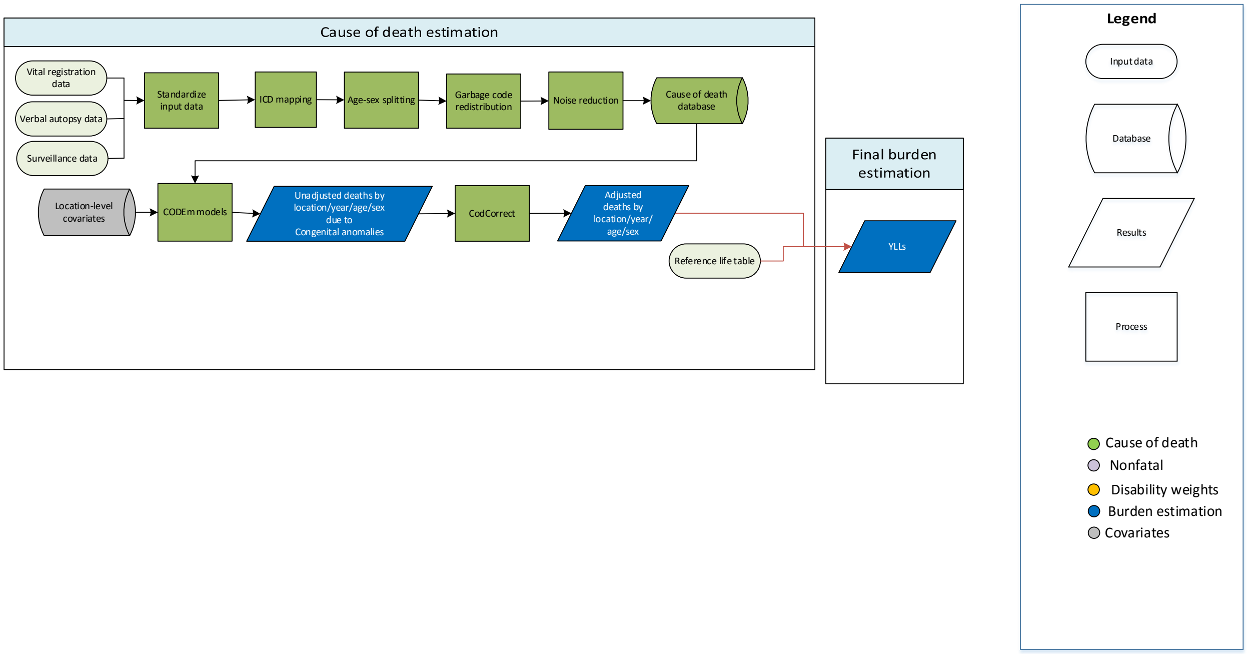

Input data

For GBD 2016, input data for estimating mortality due to congenital anomalies was centrally extracted, processed, and stored in causes of death (CoD) database. Vital registration (VR) was the dominant data type, followed by verbal autopsy (VA) and surveillance. Those CoD data sources that specified the sub-cause of birth defect were included in estimation of both the parent congenital anomalies model as well as in sub-type-specific models.

For GBD 2016, data exclusions were limited. We outliered all VA data in those over 5 years old as the age patterns were unreliable and led to poor model performance in the under-5 age groups. We also excluded some data sources from the parent model where only a subset of sub-causes were specified (eg, congenital heart disease, neural tube defects, and other congenital anomalies) and the sum of the sub-causes clearly represented systematic underreporting of one of the sub-causes. Systematic underreporting was suspected when sex- and age-specific rates were more than an order of magnitude lower than neighboring or comparable locations. Data sources for those locations were still included by default for sub-cause specific models because under-reporting of the total was not assumed to necessarily be associated with under-reporting of all of the component conditions.

Modeling strategy

All types of congenital anomalies were estimated using cause of death ensemble modeling (CODEm) for GBD 2016, as was done for previous iterations of the GBD study. Specific causes included neural tube defects, congenital heart anomalies, orofacial clefts, Down Syndrome, other chromosomal anomalies, congenital musculoskeletal anomalies, urogenital congenital anomalies, digestive congenital anomalies, and other congenital birth defects. We assumed no mortality from either Klinefelter syndrome or Turner syndrome, for which we model nonfatal outcomes only. For GBD 2016, we modeled congenital anomalies as a cause of death for ages 0-69 years only, assuming that all mortality from congenital conditions occurs before age 70 years of age.

For GBD 2016, we added three new causes to the congenital anomalies: congenital musculoskeletal and limb anomalies; urogenital congenital anomalies; and digestive congenital anomalies.

Covariates selected for CODEm model of overall congenital birth defects

Covariate	Transformation	Level	Direction
Maternal alcohol consumption during pregnancy (proportion)	None	1	Positive
In-Facility Delivery (proportion)	None	1	Negative
Live Births 35+ (proportion)	None	1	Positive
Folic acid unadjusted (ug)	None	1	Negative
Legality of Abortion	None	2	Negative
Antenatal Care (1 visit) Coverage (proportion)	None	2	Not specified
Smoking Prevalence (Reproductive Age Standardized)	None	2	Positive
Antenatal Care (4 visits) Coverage (proportion)	None	2	Negative
Healthcare access and quality index	None	2	Negative
Education (years per capita)	None	2	Negative
Alcohol (liters per capita)	None	3	Positive
fruits unadjusted(g)	None	3	Positive
Outdoor Air Pollution (PM2.5)	None	3	Positive
Indoor Air Pollution (All Cooking Fuels)	None	3	Positive
Socio-demographic Index	None	3	Negative
vegetables unadjusted(g)	None	3	Positive

Covariates selected for CODEm model of neural tube defects

Covariate	Transformation	Level	Direction
Health System Access (capped)	None	1	Negative
fruits adjusted(g)	None	2	Negative
vegetables adjusted(g)	None	2	Negative
Healthcare access and quality index	None	2	Negative
Education (years per capita)	None	3	Negative
LDI (I$ per capita)	Log	3	Negative

Socio-demographic Index	None	3	Negative

Covariates selected for CODEm model of congenital heart anomalies

Covariate	Transformation	Level	Direction
Maternal alcohol consumption during pregnancy (proportion)	None	1	Positive
Socio-demographic Index	Log	2	Negative
Smoking Prevalence (Reproductive Age Standardized)	None	2	Positive
Diabetes Age-Standardized Prevalence (proportion)	None	2	Positive
Healthcare access and quality index	None	2	Negative
Legality of Abortion	None	2	Negative
Antenatal Care (1 visit) Coverage (proportion)	None	2	Negative
In-Facility Delivery (proportion)	None	2	Negative
Education (years per capita)	None	2	Negative
Alcohol (liters per capita)	None	3	Positive
Antenatal Care (4 visits) Coverage (proportion)	None	3	Negative
Skilled Birth Attendance (proportion)	None	3	Negative
Live Births 35+ (proportion)	None	3	Positive

Covariates selected for CODEm model of cleft lip and cleft palate

Covariate	Transformation	Level	Direction
Indoor Air Pollution (All Cooking Fuels)	None	1	Positive
Diabetes Age-Standardized Prevalence (proportion)	None	2	Positive
Maternal alcohol consumption during pregnancy (proportion)	None	2	Positive
Healthcare access and quality index	None	2	Negative
Outdoor Air Pollution (PM2.5)	None	2	Positive
Legality of Abortion	None	2	Negative
Skilled Birth Attendance (proportion)	None	2	Negative
Smoking Prevalence (Reproductive Age Standardized)	None	2	Positive
vegetables unadjusted(g)	None	3	Not specified
Alcohol (liters per capita)	None	3	Positive
Antenatal Care (4 visits) Coverage (proportion)	None	3	Negative
Education (years per capita)	None	3	Negative
fruits unadjusted(g)	None	3	Not specified
Antenatal Care (1 visit) Coverage (proportion)	None	3	Negative

Covariates selected for CODEm model of Down Syndrome

Covariate	Transformation	Level	Direction
Live Births 35+ (proportion)	None	1	Positive
Legality of Abortion	None	1	Negative
Live Births 40+ (proportion)	None	1	Positive
Socio-demographic Index	None	2	Negative
LDI (I$ per capita)	Log	2	Negative
In-Facility Delivery (proportion)	None	2	Negative
Healthcare access and quality index	None	2	Negative
Maternal alcohol consumption during pregnancy (proportion)	None	3	Positive
Antenatal Care (1 visit) Coverage (proportion)	None	3	Negative
Education (years per capita)	None	3	Negative
Indoor Air Pollution (All Cooking Fuels)	None	3	Positive
Antenatal Care (4 visits) Coverage (proportion)	None	3	Negative
vegetables unadjusted(g)	None	3	Negative
Smoking Prevalence (Reproductive Age Standardized)	None	3	Positive

Covariates selected for CODEm model of other chromosomal abnormalities

Covariate	Transformation	Level	Direction
Live Births 35+ (proportion)	None	1	Positive
Live Births 40+ (proportion)	None	1	Positive
Legality of Abortion	None	1	Negative
LDI (I$ per capita)	Log	2	Negative
Healthcare access and quality index	None	2	Negative
Antenatal Care (4 visits) Coverage (proportion)	None	2	Negative
Antenatal Care (1 visit) Coverage (proportion)	None	2	Negative
In-Facility Delivery (proportion)	None	2	Negative
Maternal alcohol consumption during pregnancy (proportion)	None	2	Positive
Socio-demographic Index	None	3	Not specified
Alcohol (liters per capita)	None	3	Positive
Smoking Prevalence (Reproductive Age Standardized)	None	3	Positive
Education (years per capita)	None	3	Negative
Skilled Birth Attendance (proportion)	None	3	Negative

Covariates selected for CODEm model of congenital musculoskeletal and limb anomalies

Covariate	Transformation	Level	Direction
Maternal alcohol consumption during pregnancy (proportion)	None	1	Positive
Legality of Abortion	None	1	Negative
In-Facility Delivery (proportion)	None	2	Negative
Diabetes Age-Standardized Prevalence (proportion)	None	2	Positive
Socio-demographic Index	None	2	Negative
Healthcare access and quality index	None	2	Negative
Indoor Air Pollution (All Cooking Fuels)	None	2	Positive

Smoking Prevalence (Reproductive Age Standardized)	None	2	Positive
Antenatal Care (4 visits) Coverage (proportion)	None	3	Negative
Alcohol (liters per capita)	None	3	Positive
vegetables unadjusted(g)	None	3	Not specified
fruits unadjusted(g)	None	3	Not specified
Education (years per capita)	None	3	Negative
Antenatal Care (1 visit) Coverage (proportion)	None	3	Negative

Covariates selected for CODEm model of urogenital congenital anomalies

Covariate	Transformation	Level	Direction
Smoking Prevalence (Reproductive Age Standardized)	None	1	Positive
Maternal alcohol consumption during pregnancy (proportion)	None	1	Positive
Healthcare access and quality index	None	2	Negative
Diabetes Age-Standardized Prevalence (proportion)	None	2	Positive
Socio-demographic Index	None	2	Negative
Outdoor Air Pollution (PM2.5)	None	2	Positive
In-Facility Delivery (proportion)	None	2	Negative
Indoor Air Pollution (All Cooking Fuels)	None	2	Positive
Antenatal Care (1 visit) Coverage (proportion)	None	3	Negative
Alcohol (liters per capita)	None	3	Positive
Education (years per capita)	None	3	Negative
LDI (I$ per capita)	Log	3	Negative
Antenatal Care (4 visits) Coverage (proportion)	None	3	Negative

Covariates selected for CODEm model of digestive congenital anomalies

Covariate	Transformation	Level	Direction
Maternal alcohol consumption during pregnancy (proportion)	None	1	Positive
Smoking Prevalence (Reproductive Age Standardized)	None	1	Positive
Indoor Air Pollution (All Cooking Fuels)	None	2	Positive
Diabetes Age-Standardized Prevalence (proportion)	None	2	Positive
Socio-demographic Index	None	2	Negative
Prevalence of obesity (age-standardized)	None	2	Positive
In-Facility Delivery (proportion)	None	2	Negative
Healthcare access and quality index	None	2	Negative
Alcohol (liters per capita)	None	3	Positive
Health System Access (capped)	None	3	Negative
Education (years per capita)	None	3	Negative
vegetables unadjusted(g)	None	3	Not specified
Antenatal Care (1 visit) Coverage (proportion)	None	3	Negative
Antenatal Care (4 visits) Coverage (proportion)	None	3	Negative
fruits unadjusted(g)	None	3	Not specified
LDI (I$ per capita)	Log	3	Negative

Covariates selected for CODEm model of other congenital birth defects

**Covariate**	**Transformation**	**Level**	**Direction**
Maternal alcohol consumption during pregnancy (proportion)	None	1	Positive
Live Births 35+ (proportion)	None	1	Positive
Education (years per capita)	None	2	Negative
Smoking Prevalence (Reproductive Age Standardized)	None	2	Positive
Legality of Abortion	None	2	Negative
In-Facility Delivery (proportion)	None	2	Negative
Indoor Air Pollution (All Cooking Fuels)	None	2	Positive
Healthcare access and quality index	None	2	Negative
Antenatal Care (1 visit) Coverage (proportion)	None	3	Negative
Diabetes Age-Standardized Prevalence (proportion)	None	3	Positive
LDI (I$ per capita)	Log	3	Negative
Socio-demographic Index	None	3	Negative
Antenatal Care (4 visits) Coverage (proportion)	None	3	Negative
Alcohol (liters per capita)	None	3	Positive

Part 2 to supplementary appendix: Injuries, including adverse effects of medical treatment

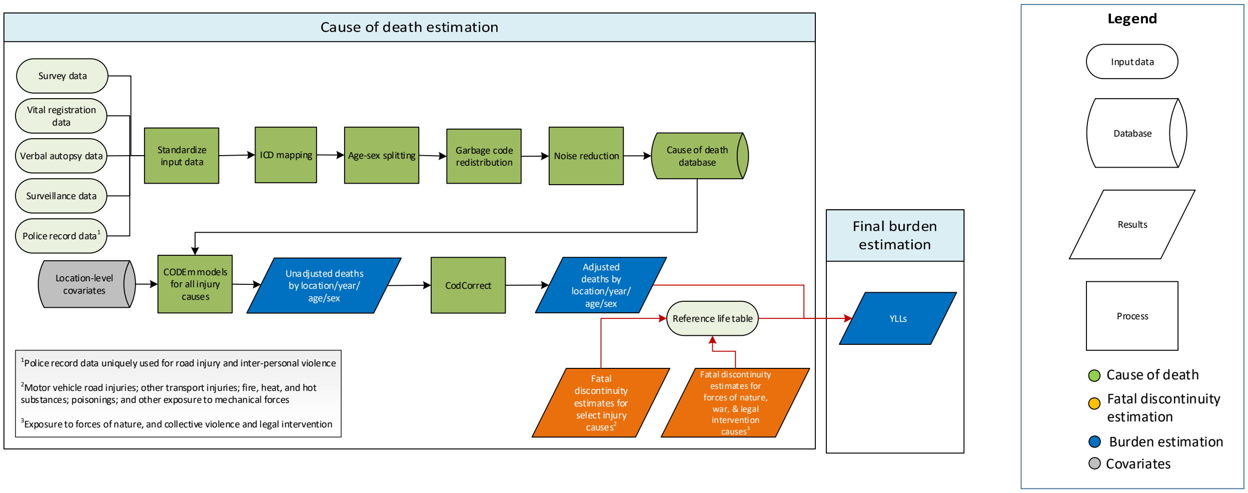

Input data

In GBD 2016, we estimated injury mortality from vital registration, verbal autopsy, mortality surveillance, censuses, surveys, and police record data. Police and crime reports were data sources uniquely used for the estimation of deaths from road traffic injury and interpersonal violence. The police data were collected from published studies, national agencies, and institutional surveys such as the United Nations Crime Trends Survey and the WHO Global Status Report on Road Safety Survey. For countries with vital registration data we did not use police records, except if the recorded number of road injury and interpersonal violence deaths from police records exceeded that in the vital registration.

Infrequently, data points were marked as outliers. Outlier criteria excluded data points that (1) were implausibly high or low relative to global or regional patterns, (2) substantially conflicted with established age or temporal patterns, or (3) significantly conflicted with other data sources conducted from the same locations or locations with similar characteristics (ie, Socio-demographic Index).

Modelling strategy

Overview

In GBD 2016, the standard CODEm modelling approach was applied to estimate deaths due to all causes of injury, excluding "Exposure to forces of nature," "Military operations and terrorism," and "State actor violence," which fall under the aggregate cause "Forces of nature, military operations and terrorism, and state actor violence." These causes were modelled solely outside of the CODEm process as fatal discontinuities estimation; this process is detailed further in the section on fatal discontinuities estimation in the appendix.

Fatal discontinuity was estimated for five injury causes also modeled in CODEm. These causes included "Motor vehicle road injuries," "Other transport injuries," "Fire, heat, and hot substances," "Poisonings," and "Other exposure to mechanical forces." Final fatal discontinuity estimations for these causes were merged with CODEm results post-CoDCorrect to produce final cause of death results.

Refer to the Table at the end of this section for a complete list of the cause-of-injury categories, modelling strategies, and covariate changes from GBD 2015.

GBD injury codes and categories

The International Classification of Diseases (ICD) was used to classify injuries. In GBD, injury incidence and death are defined as ICD-9 codes E000-E999 and ICD-10 chapters V to Y. There is one exception: deaths and cases of alcohol poisoning and drug overdoses are classified under drug and alcohol use disorders. In GBD 2016, injury causes were organized into 26 mutually exclusive and collectively exhaustive external cause-of-injury categories. For GBD 2016, "Self-harm" was distinguished into "Self-harm by firearm," and "Self-harm by other specified means."

Preparation of data

The preparation of cause of death data includes age splitting, age-sex splitting, smoothing, and outlier detection. These steps are described in detail by Naghavi et al and Lozano et al.[1,2,3] The concept of "garbage codes" and redistribution of these codes was proposed in GBD 1990.[4] Garbage codes are causes of death that should not be identified as specific underlying causes of death but have been entered as the underlying cause of death on death certificates. A classic example of these types of codes in injuries chapters are "Exposure to unspecified factor" (X59 in ICD-10 and E887 in ICD-9) and all undetermined

intent codes (Y10-Y34 in ICD-10 and E980-E988 in ICD-9). Other examples of garbage codes in injuries are the coding of an injury death to intermediate codes like septicemia or peritonitis or as an ill-defined and unknown cause of mortality (R99). Approximately 2% of total deaths in countries with vital registration data are assigned to these three injury garbage code categories.

Splitting into sublevel causes

In countries with non-detail ICD code data, cause-of-injury categories were proportionally split into sublevel cause-of-injury categories. The sublevel cause-of-injury causes were created in the CoDCorrect process. One of the countries with non-detail ICD code data is South Africa, and in GBD 2013 the proportions of sublevel cause-of-injury were based on vital registration data. For GBD iterations of 2015 and 2016 the proportions were based on post-mortem investigation of injury deaths as described in the paper by Matzopoulos et al. 2015.[5]

Limitations and model assumptions

We added police data for road injuries and interpersonal violence to help predict level and age patterns in countries with sparse or absent cause of death data even though we know from countries with near-complete vital registration data that police records tend to underestimate the true level of deaths. However, we applied police data estimates in instances where reported deaths were higher than vital registration numbers.

For the cause-of-injury category “Unintentional suffocation” we suspect that varying practices in coding deaths to sudden infant death syndrome (which end up in “Unintentional suffocation”) can explain some of the differences we see and we plan to explore that further in the next iteration of GBD.

Table – Injury Cause List			
ID	Cause	Modelling Strategy	Covariate changes from GBD 2015
1	Transport injuries	CODEm	
1.1	Road injuries	CODEm	
1.1.a	Pedestrian road injuries	CODEm	
1.1.b	Cyclist road injuries	CODEm	
1.1.c	Motorcyclist road injuries	CODEm	
1.1.d	Motor vehicle road injuries	CODEm and fatal discontinuity estimation	
1.1.e	Other road injuries	CODEm	
1.2	Other transport injuries	CODEm and fatal discontinuity estimation	
2	Unintentional injuries	CODEm	
2.1	Falls	CODEm	
2.2	Drowning	CODEm	
2.3	Fire, heat, and hot substances	CODEm and fatal discontinuity estimation	
2.4	Poisonings	CODEm and fatal discontinuity estimation	
2.5	Exposure to mechanical forces	CODEm	
2.5.a	Unintentional firearm injuries	CODEm	
2.5.b	Unintentional suffocation	CODEm	
2.5.c	Other exposure to mechanical forces	CODEm and fatal discontinuity estimation	
2.6	Adverse effects of medical treatment	CODEm	
2.7	Animal contact	CODEm	
2.7.a	Venomous animal contact	CODEm	
2.7.b	Non-venomous animal contact	CODEm	
2.8	Foreign body	CODEm	
2.8.a	Pulmonary aspiration and foreign body in airway	CODEm	
2.8.b	Foreign body in other body part	CODEm	
2.9	Environmental exposure to heat and cold	CODEm	
2.10	Other unintentional injuries	CODEm	
3	Self-harm and interpersonal violence	CODEm	
3.1	Self-harm	CODEm	
3.1.1	Self-harm by firearm	CODEm	Same covariates used as self-harm from GBD 2015
3.1.2	Self-harm by other specified means	CODEm	Same covariates used as self-harm from GBD 2015
3.2	Interpersonal violence	CODEm	
3.2.a	Assault by firearm	CODEm	
3.2.b	Assault by sharp object	CODEm	
3.2.c	Assault by other means	CODEm	
4	Forces of nature, military operations and terrorism, and state actor violence		

Table – Injury Cause List			
ID	Cause	Modelling Strategy	Covariate changes from GBD 2015
4.1	Exposure to forces of nature	Fatal discontinuity estimation for disaster (appended post-CoDCorrect)	N/A
4.2	State actor violence	Fatal discontinuity estimation for state actor violence (appended post-CoDCorrect)	N/A
4.3	Military operations and terrorism	Fatal discontinuity estimation for state actor violence (appended post-CoDCorrect)	N/A

References

1 Lozano R, Naghavi M, Foreman K, *et al.* Global and regional mortality from 235 causes of death for 20 age groups in 1990 and 2010: a systematic analysis for the Global Burden of Disease Study 2010. *The Lancet* 2012; **380**: 2095–128.

2 Global, regional, and national age–sex specific all-cause and cause-specific mortality for 240 causes of death, 1990–2013: a systematic analysis for the Global Burden of Disease Study 2013. *The Lancet* 2015; **385**: 117–71.

3 Global, regional, and national life expectancy, all-cause mortality, and cause-specific mortality for 249 causes of death, 1980-2015: a systematic analysis for the Global Burden of Disease Study 2015. *The Lancet* 2016; **388**: 1459-1544.

4 Murray CJL, Lopez AD, Harvard School of Public Health, World Health Organization, World Bank. The global burden of disease: a comprehensive assessment of mortality and disability from diseases, injuries, and risk factors in 1990 and projected to 2020. Cambridge, MA: Published by the Harvard School of Public Health on behalf of the World Health Organization and the World Bank : Distributed by Harvard University Press, 1996.

5 Matzopoulos R, Prinsloo M, Wyk VP, Gwebushe N, Mathews S, et al. Injury-related mortality in South Africa: a retrospective descriptive study of postmortem investigations. *Bull World Health Organ* 2015; **93**: 303–13.

Part 3. Online tools and glossary of terms

Section 1. Online tools

GBD 2016 data sources and additional results are presented in a series of tools and dynamic visualizations at http://ghdx.healthdata.org/gbd-2016

All results from the GBD 2016 HAQ Index analysis can be downloaded from the GHDx: http://ghdx.healthdata.org/record/global-burden-disease-study-2016-gbd-2016-healthcare-access-and-quality-index-based-amenable

Section 2. List of abbreviations

DAH	Development assistance for health
GATHER	Guidelines for Accurate and Transparent Health Estimates Reporting
GBD	Global Burden of Disease
GHDx	Global Health Data Exchange
HAQ Index	Healthcare Access and Quality Index
MDGs	Millennium Development Goals
MIRs	Mortality-to-incidence ratios
NCDs	Non-communicable diseases
OECD	Organisation for Economic Co-operation and Development
PAF	Population attributable fraction
PCA	Principal components analysis
SDGs	Sustainable Development Goals
SDI	Socio-demographic Index
UHC	Universal health coverage
UI	Uncertainty interval
VR	Vital registration

Section 3. List of ISO3 codes and location names

AFG	Afghanistan
AGO	Angola
ALB	Albania
AND	Andorra
ARE	United Arab Emirates
ARG	Argentina
ARM	Armenia
ASM	American Samoa
ATG	Antigua and Barbuda
AUS	Australia
AUT	Austria
AZE	Azerbaijan

BDI	Burundi
BEL	Belgium
BEN	Benin
BFA	Burkina Faso
BGD	Bangladesh
BGR	Bulgaria
BHR	Bahrain
BHS	The Bahamas
BIH	Bosnia and Herzegovina
BLR	Belarus
BLZ	Belize
BMU	Bermuda
BOL	Bolivia
BRA	Brazil
BRB	Barbados
BRN	Brunei
BTN	Bhutan
BWA	Botswana
CAF	Central African Republic
CAN	Canada
CHE	Switzerland
CHL	Chile
CHN	China
CIV	Cote d'Ivoire
CMR	Cameroon
COD	Democratic Republic of the Congo
COG	Congo (Brazzaville)
COL	Colombia
COM	Comoros
CPV	Cape Verde
CRI	Costa Rica
CUB	Cuba
CYP	Cyprus
CZE	Czech Republic
DEU	Germany
DJI	Djibouti
DMA	Dominica
DNK	Denmark
DOM	Dominican Republic
DZA	Algeria
ECU	Ecuador
EGY	Egypt
ERI	Eritrea
ESP	Spain

EST	Estonia
ETH	Ethiopia
FIN	Finland
FJI	Fiji
FRA	France
FSM	Federated States of Micronesia
GAB	Gabon
GBR	United Kingdom
GEO	Georgia
GHA	Ghana
GIN	Guinea
GMB	The Gambia
GNB	Guinea-Bissau
GNQ	Equatorial Guinea
GRC	Greece
GRD	Grenada
GRL	Greenland
GTM	Guatemala
GUM	Guam
GUY	Guyana
HND	Honduras
HRV	Croatia
HTI	Haiti
HUN	Hungary
IDN	Indonesia
IND	India
IRL	Ireland
IRN	Iran
IRQ	Iraq
ISL	Iceland
ISR	Israel
ITA	Italy
JAM	Jamaica
JOR	Jordan
JPN	Japan
KAZ	Kazakhstan
KEN	Kenya
KGZ	Kyrgyzstan
KHM	Cambodia
KIR	Kiribati
KOR	South Korea
KWT	Kuwait
LAO	Laos
LBN	Lebanon

LBR	Liberia
LBY	Libya
LCA	Saint Lucia
LKA	Sri Lanka
LSO	Lesotho
LTU	Lithuania
LUX	Luxembourg
LVA	Latvia
MAR	Morocco
MDA	Moldova
MDG	Madagascar
MDV	Maldives
MEX	Mexico
MHL	Marshall Islands
MKD	Macedonia
MLI	Mali
MLT	Malta
MMR	Myanmar
MNE	Montenegro
MNG	Mongolia
MNP	Northern Mariana Islands
MOZ	Mozambique
MRT	Mauritania
MUS	Mauritius
MWI	Malawi
MYS	Malaysia
NAM	Namibia
NER	Niger
NGA	Nigeria
NIC	Nicaragua
NLD	Netherlands
NOR	Norway
NPL	Nepal
NZL	New Zealand
OMN	Oman
PAK	Pakistan
PAN	Panama
PER	Peru
PHL	Philippines
PNG	Papua New Guinea
POL	Poland
PRI	Puerto Rico
PRK	North Korea
PRT	Portugal

PRY	Paraguay
PSE	Palestine
QAT	Qatar
ROU	Romania
RUS	Russia
RWA	Rwanda
SAU	Saudi Arabia
SDN	Sudan
SEN	Senegal
SGP	Singapore
SLB	Solomon Islands
SLE	Sierra Leone
SLV	El Salvador
SOM	Somalia
SRB	Serbia
SSD	South Sudan
STP	Sao Tome and Principe
SUR	Suriname
SVK	Slovakia
SVN	Slovenia
SWE	Sweden
SWZ	Swaziland
SYC	Seychelles
SYR	Syria
TCD	Chad
TGO	Togo
THA	Thailand
TJK	Tajikistan
TKM	Turkmenistan
TLS	Timor-Leste
TON	Tonga
TTO	Trinidad and Tobago
TUN	Tunisia
TUR	Turkey
TWN	Taiwan (Province of China)
TZA	Tanzania
UGA	Uganda
UKR	Ukraine
URY	Uruguay
USA	United States
UZB	Uzbekistan
VCT	Saint Vincent and the Grenadines
VEN	Venezuela
VIR	Virgin Islands

VNM	Vietnam
VUT	Vanuatu
WSM	Samoa
YEM	Yemen
ZAF	South Africa
ZMB	Zambia
ZWE	Zimbabwe

Supplementary figure 1. Comparing unscaled MIRs to age−standardised risk−standardised death rates for breast cancer, 2016. MIRs = mortality-to-incidence ratios. SDI = Socio-demographic index.

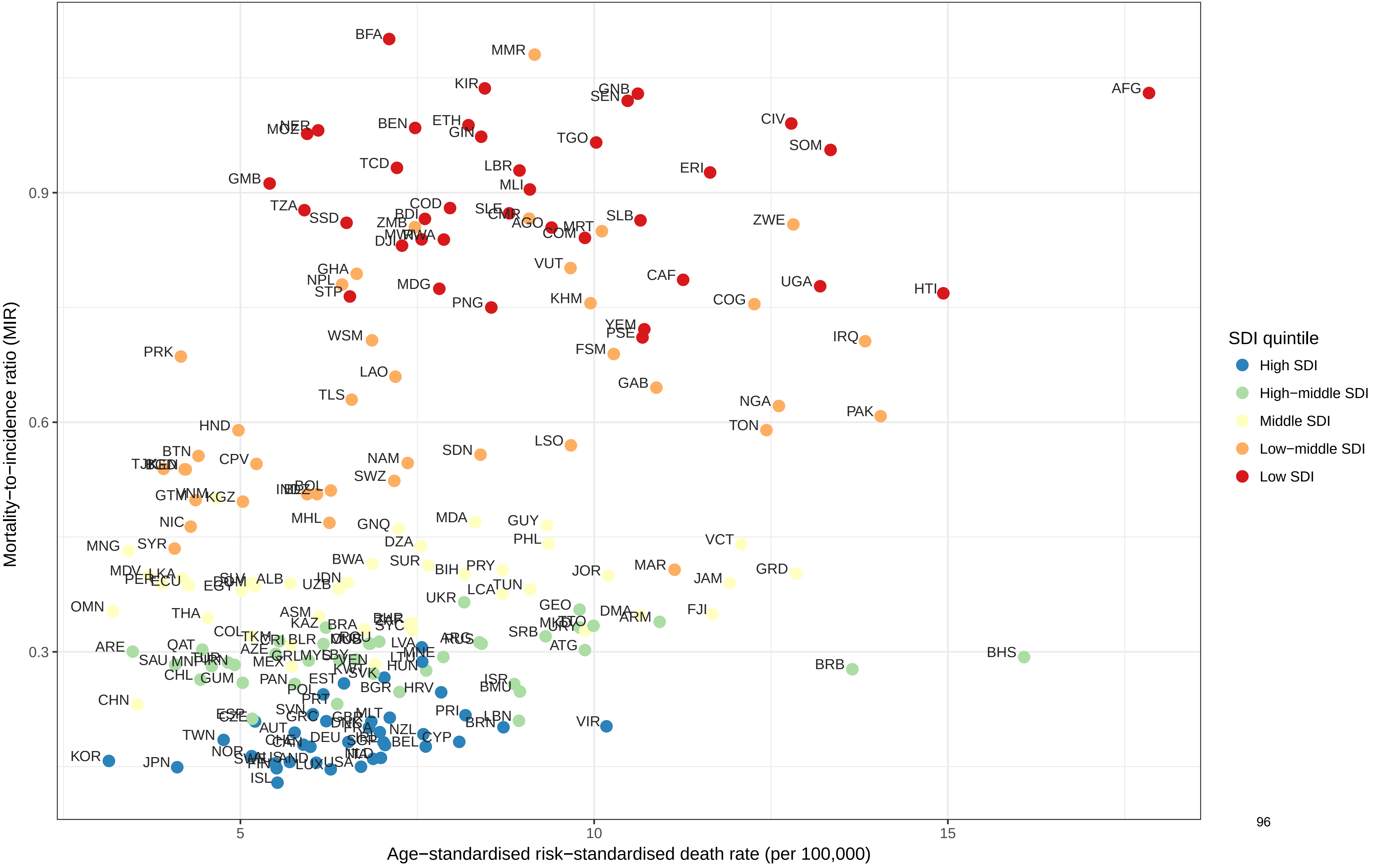

Supplementary figure 1. Comparing unscaled MIRs to age−standardised risk−standardised death rates for cervical cancer, 2016. MIRs = mortality-to-incidence ratios. SDI = Socio-demographic index.

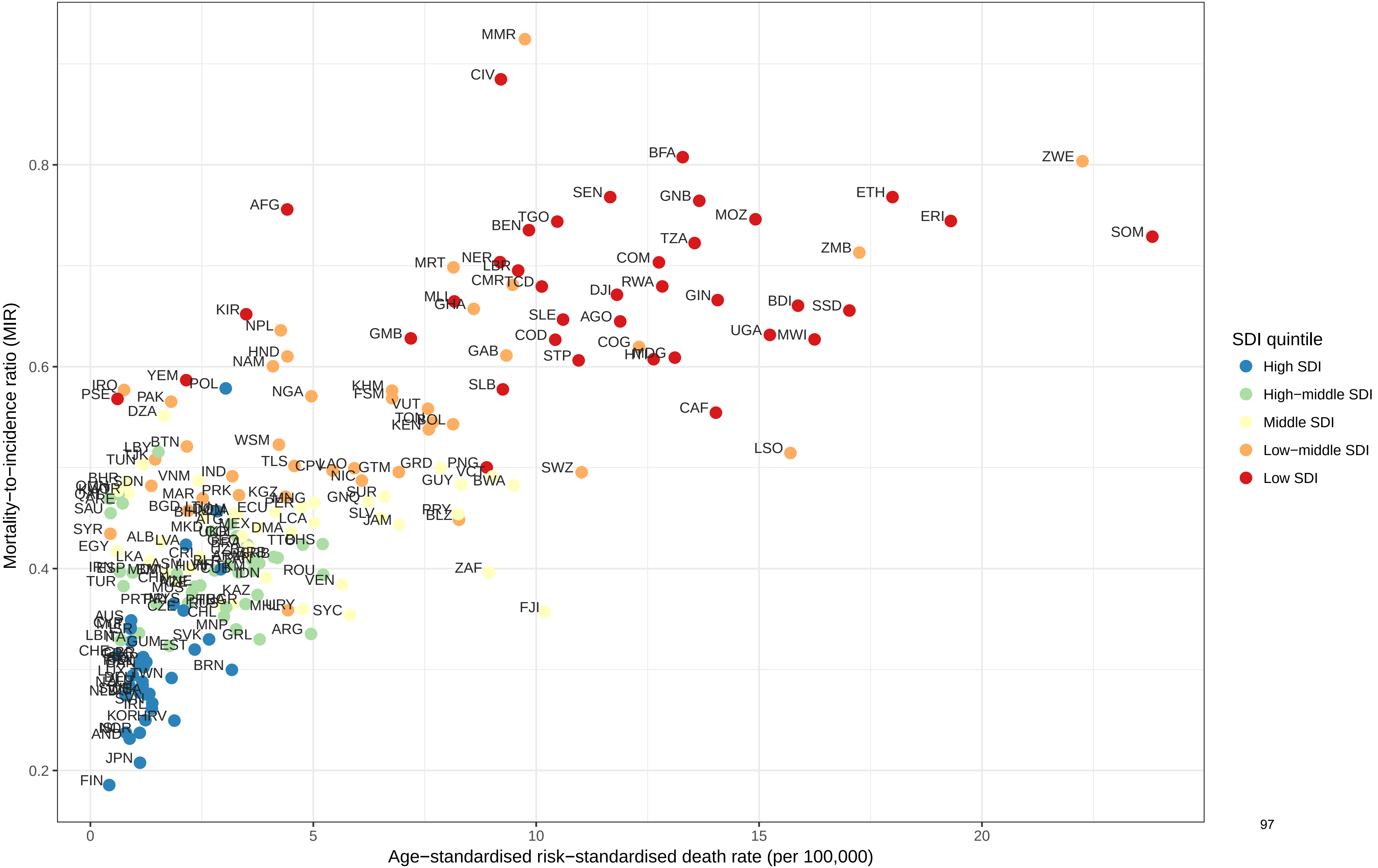

Supplementary figure 1. Comparing unscaled MIRs to age−standardised risk−standardised death rates for uterine cancer, 2016. MIRs = mortality-to-incidence ratios. SDI = Socio-demographic index.

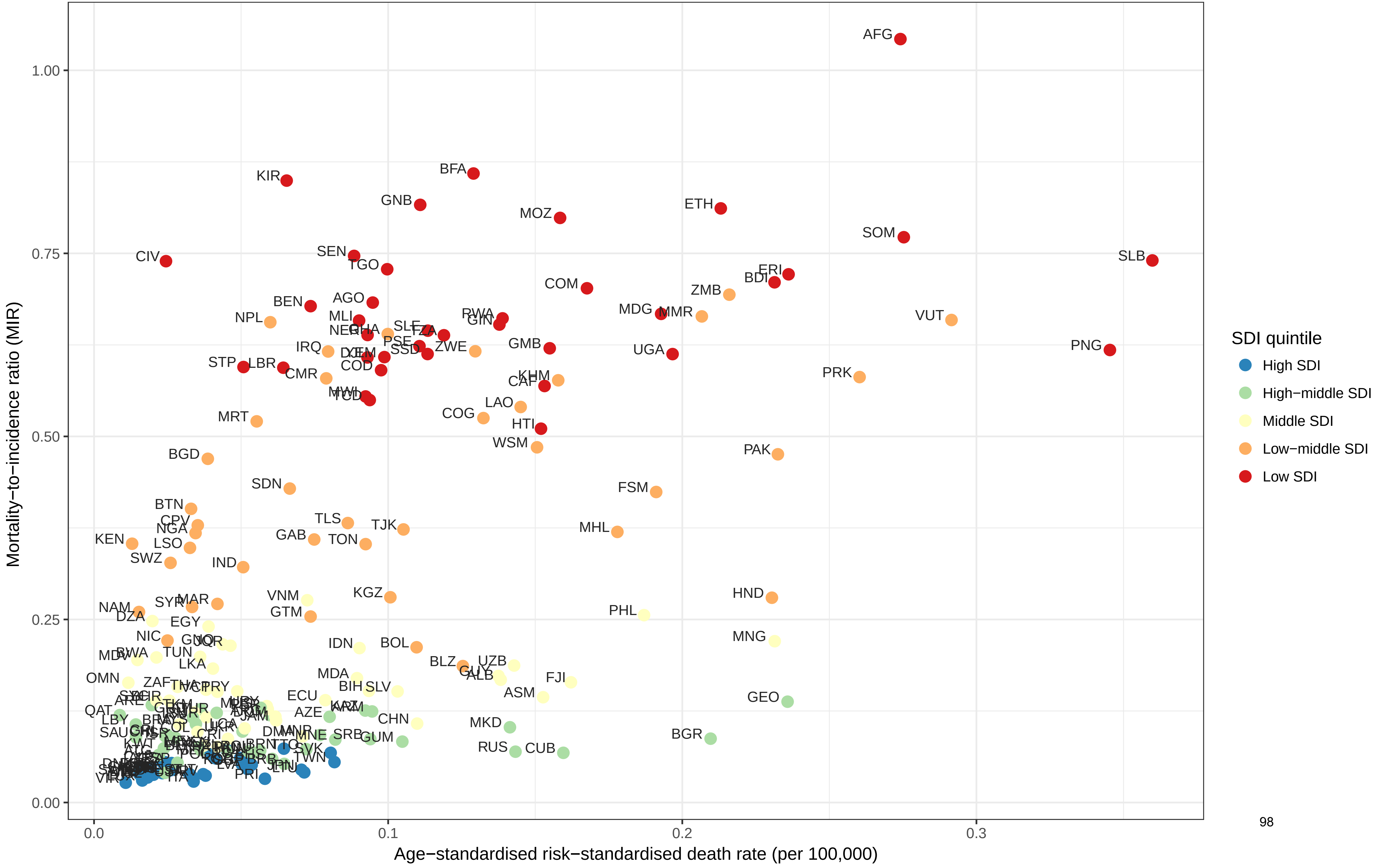

Supplementary figure 1. Comparing unscaled MIRs to age−standardised risk−standardised death rates for colon and rectum cancer, 2016. MIRs = mortality-to-incidence ratios. SDI = Socio-demographic index.

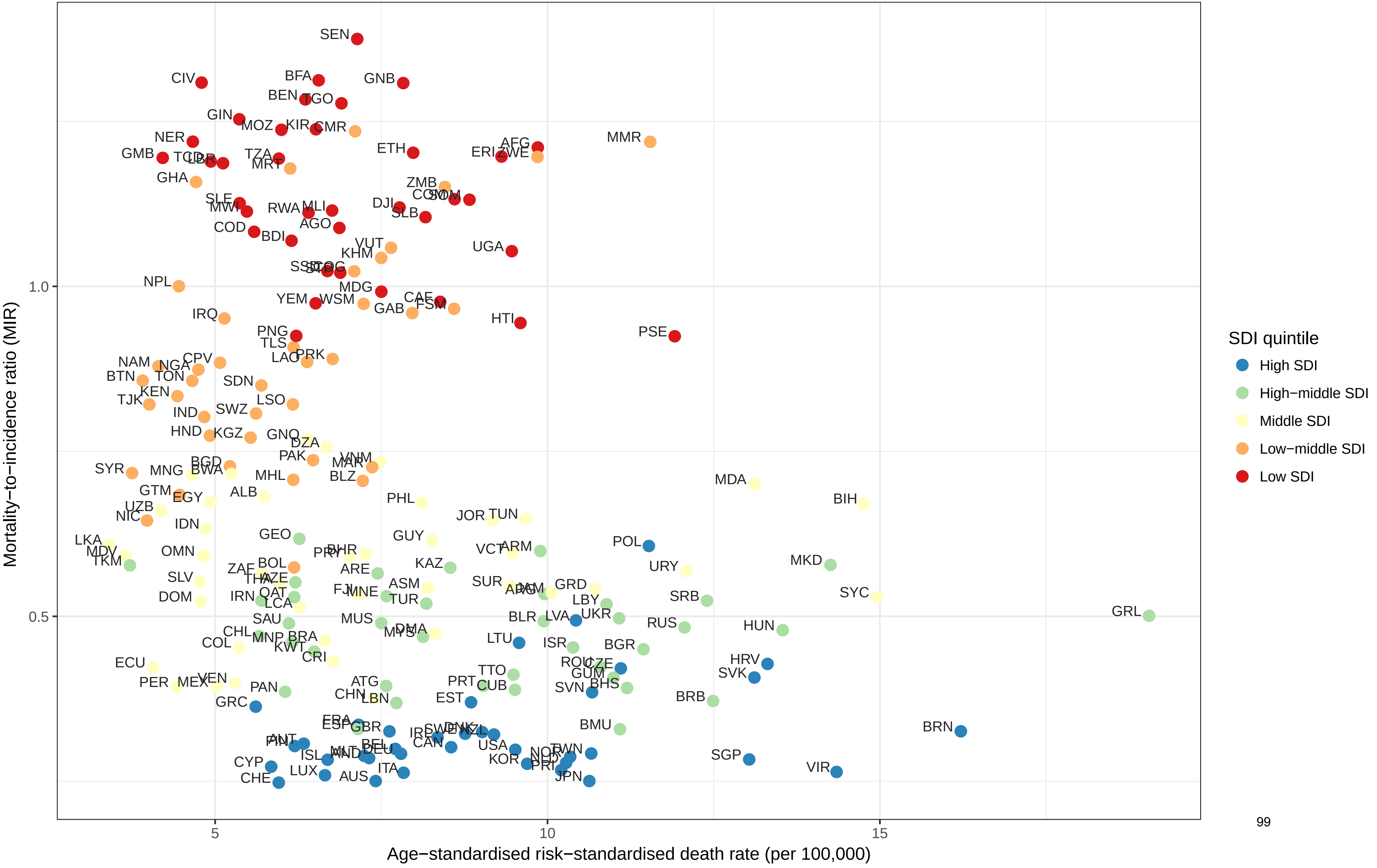

Supplementary figure 1. Comparing unscaled MIRs to age−standardized risk−standardised death rates for testicular cancer, 2016. MIRs = mortality-to-incidence ratios. SDI = Socio-demographic index.

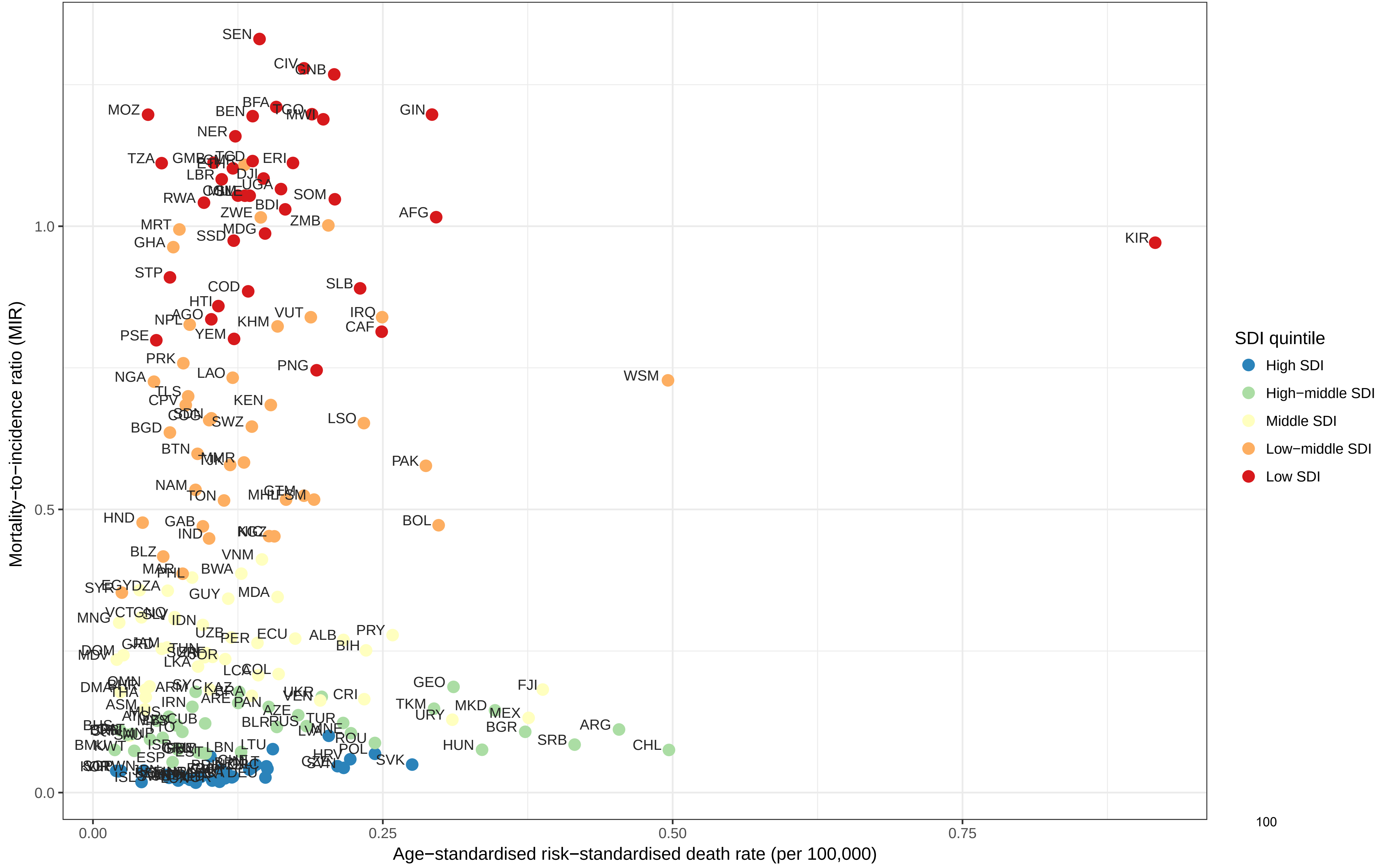

Supplementary figure 1. Comparing unscaled MIRs to age−standardised risk−standardised death rates for Hodgkin lymphoma, 2016. MIRs = mortality-to-incidence ratios. SDI = Socio-demographic index.

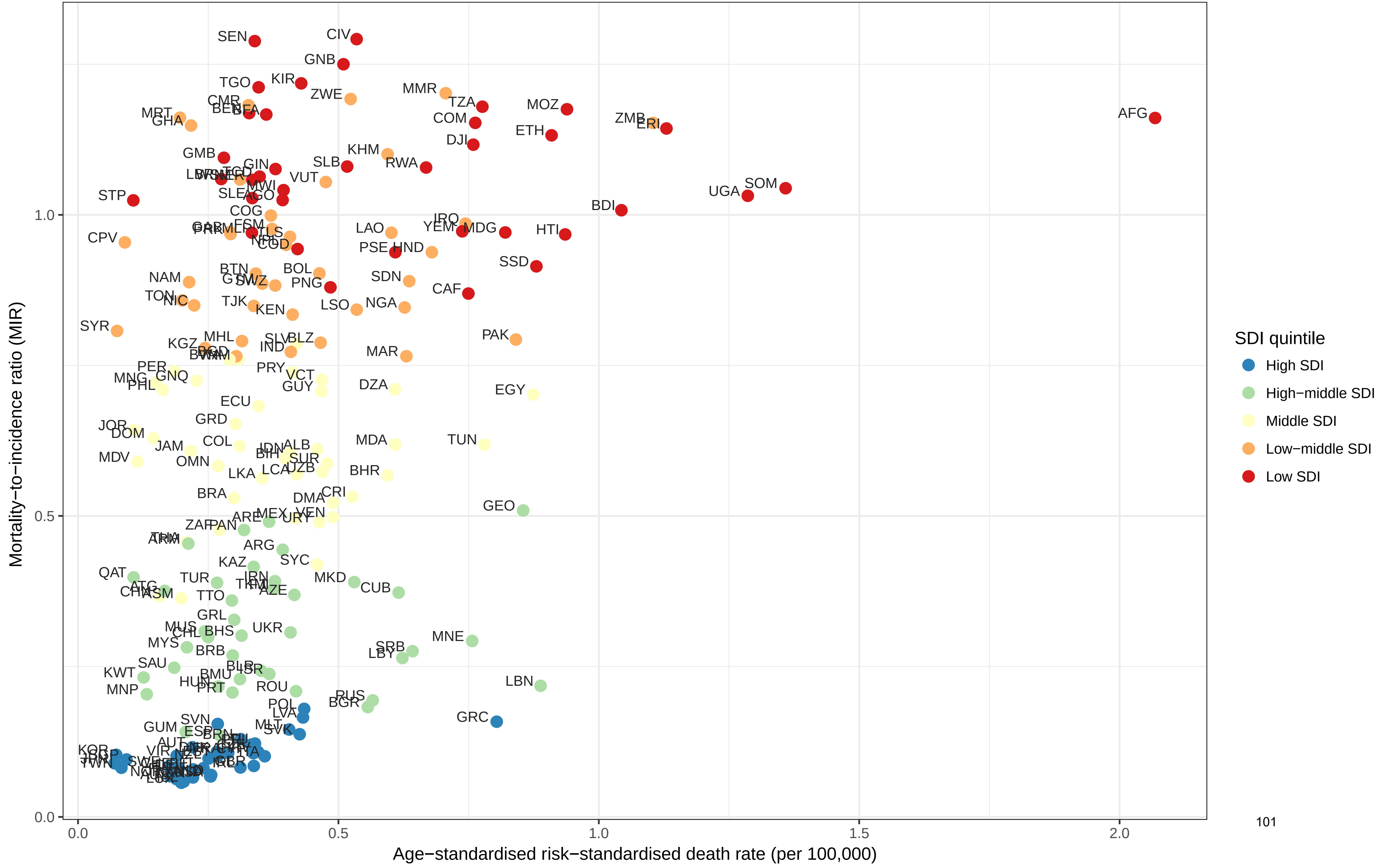

Supplementary figure 1. Comparing unscaled MIRs to age−standardised risk−standardised death rates for leukaemia, 2016. MIRs = mortality-to-incidence ratios. SDI = Socio-demographic index.

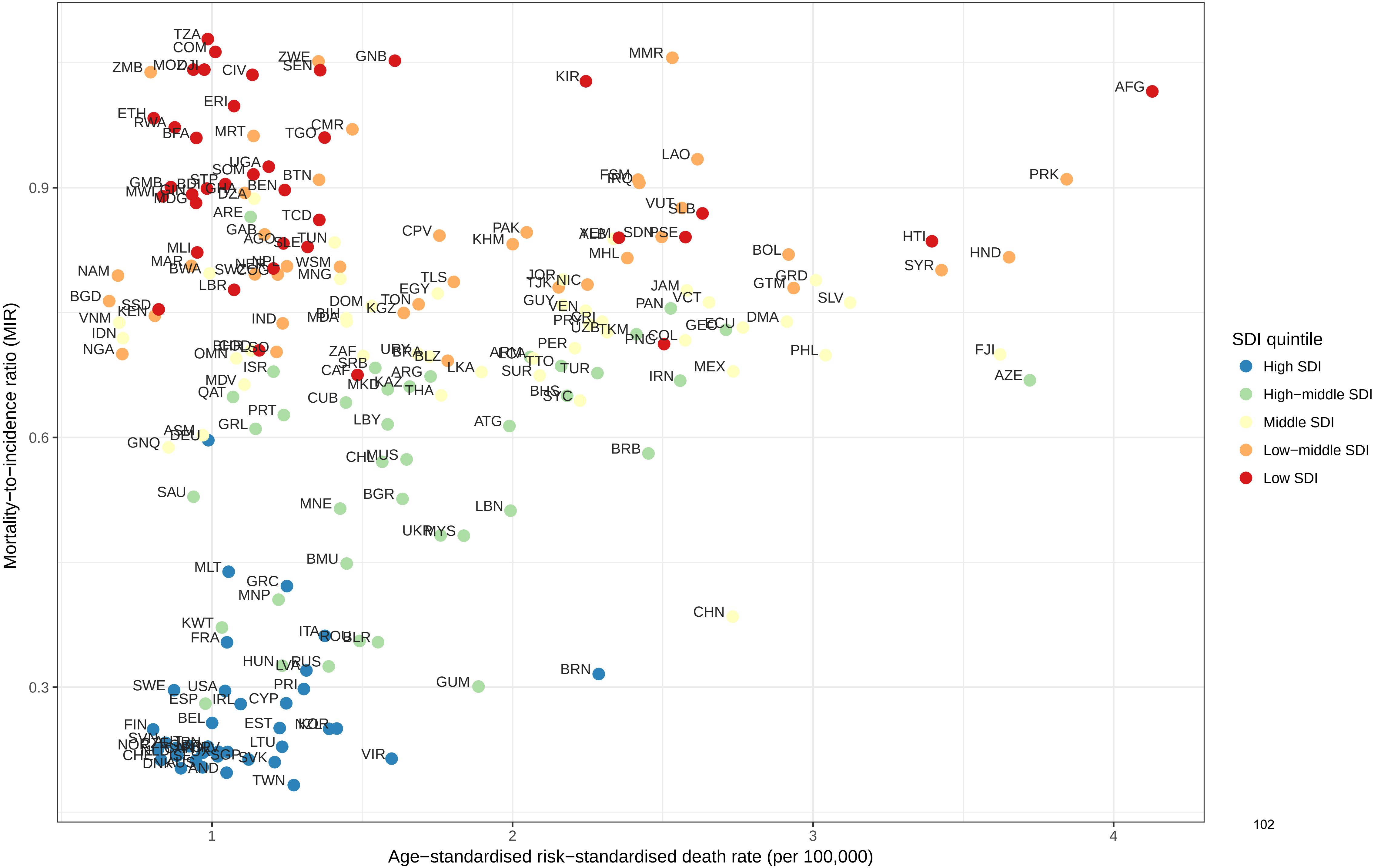

Supplementary figure 1. Comparing unscaled MIRs to age−standardised risk−standardised death rates for non−melanoma skin cancer (squamous−cell carcinoma), 2016. MIRs = mortality-to-incidence ratios. SDI = Socio-demographic index.

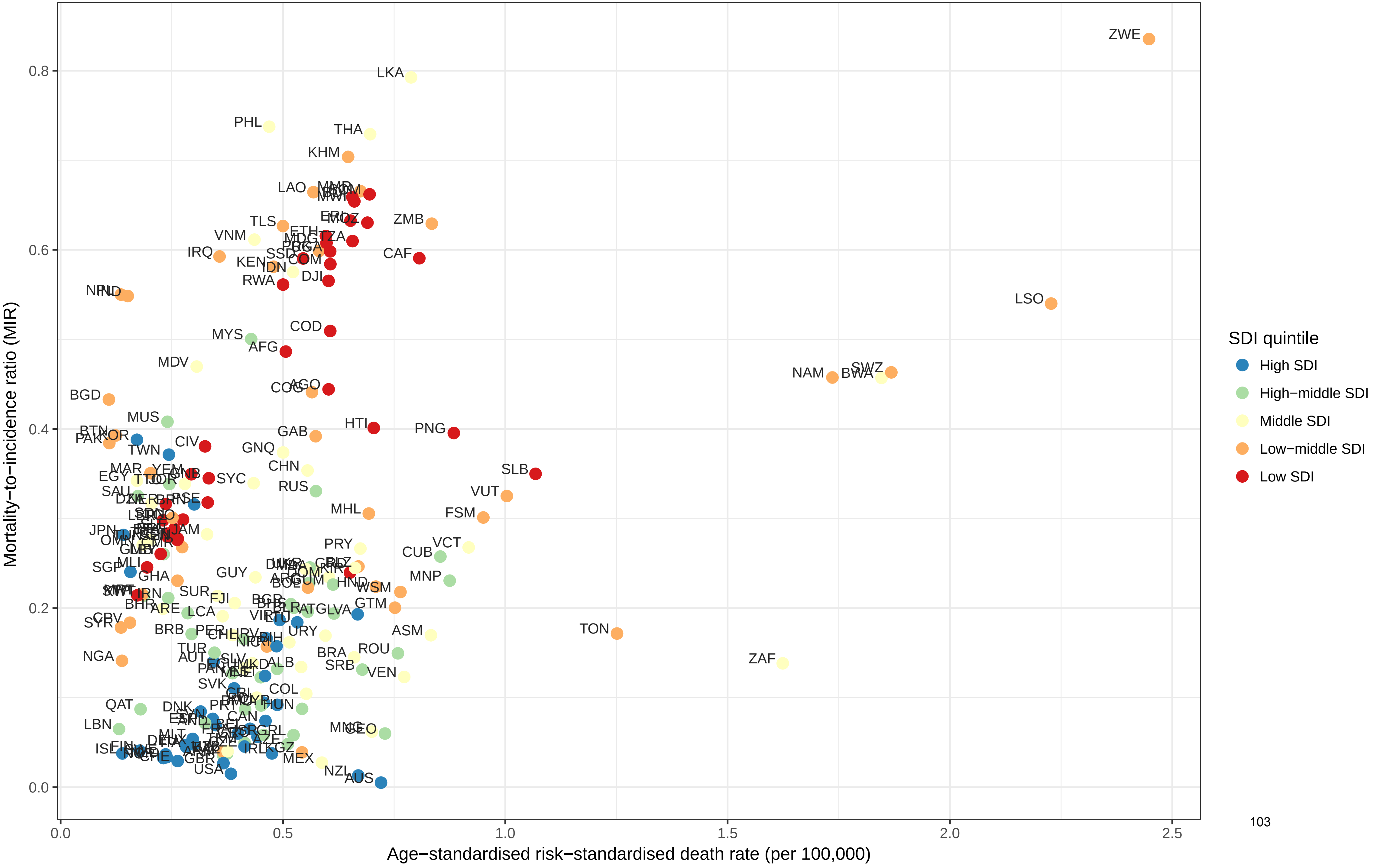

Supplementary figure 2. Comparing scaled MIRs to age−standised risk−standardised death rates for breast cancer, 2016. MIRs = mortality-to-incidence ratios. SDI = Socio-demographic index.

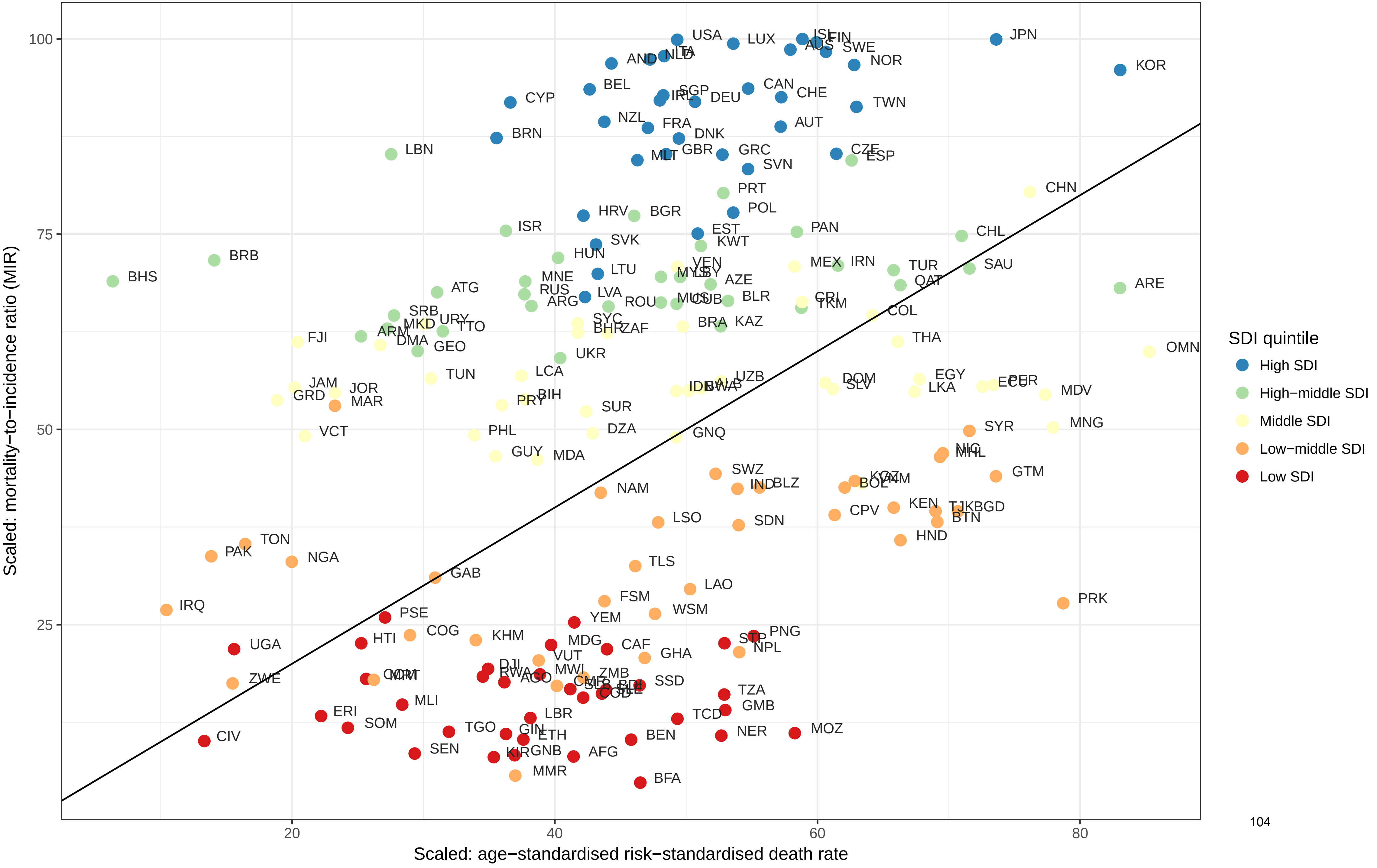

Supplementary figure 2. Comparing scaled MIRs to age−standised risk−standardised death rates for cervical cancer, 2016. MIRs = mortality-to-incidence ratios. SDI = Socio-demographic index.

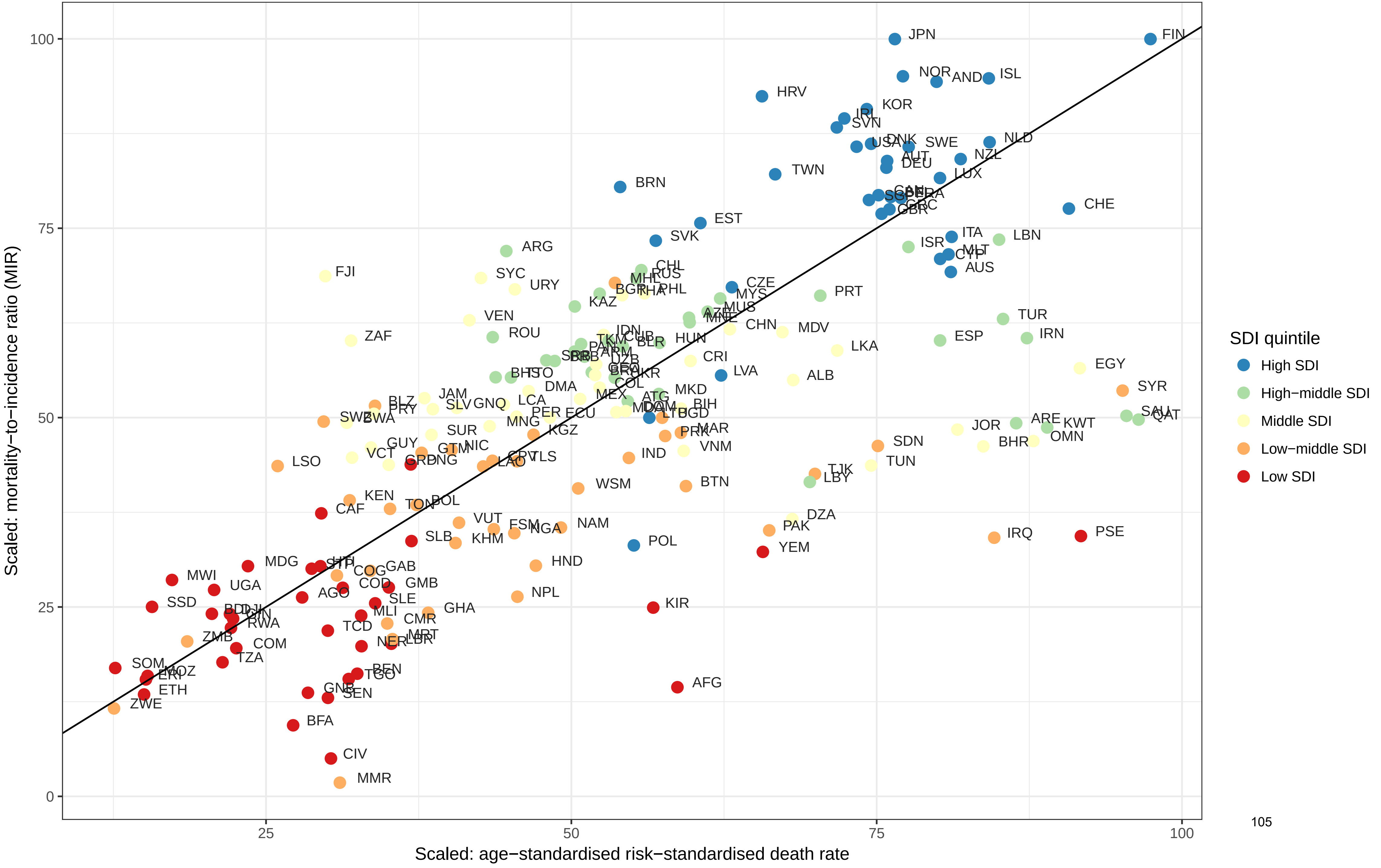

Supplementary figure 2. Comparing scaled MIRs to age−standised risk−standardised death rates for uterine cancer, 2016. MIRs = mortality-to-incidence ratios. SDI = Socio-demographic index.

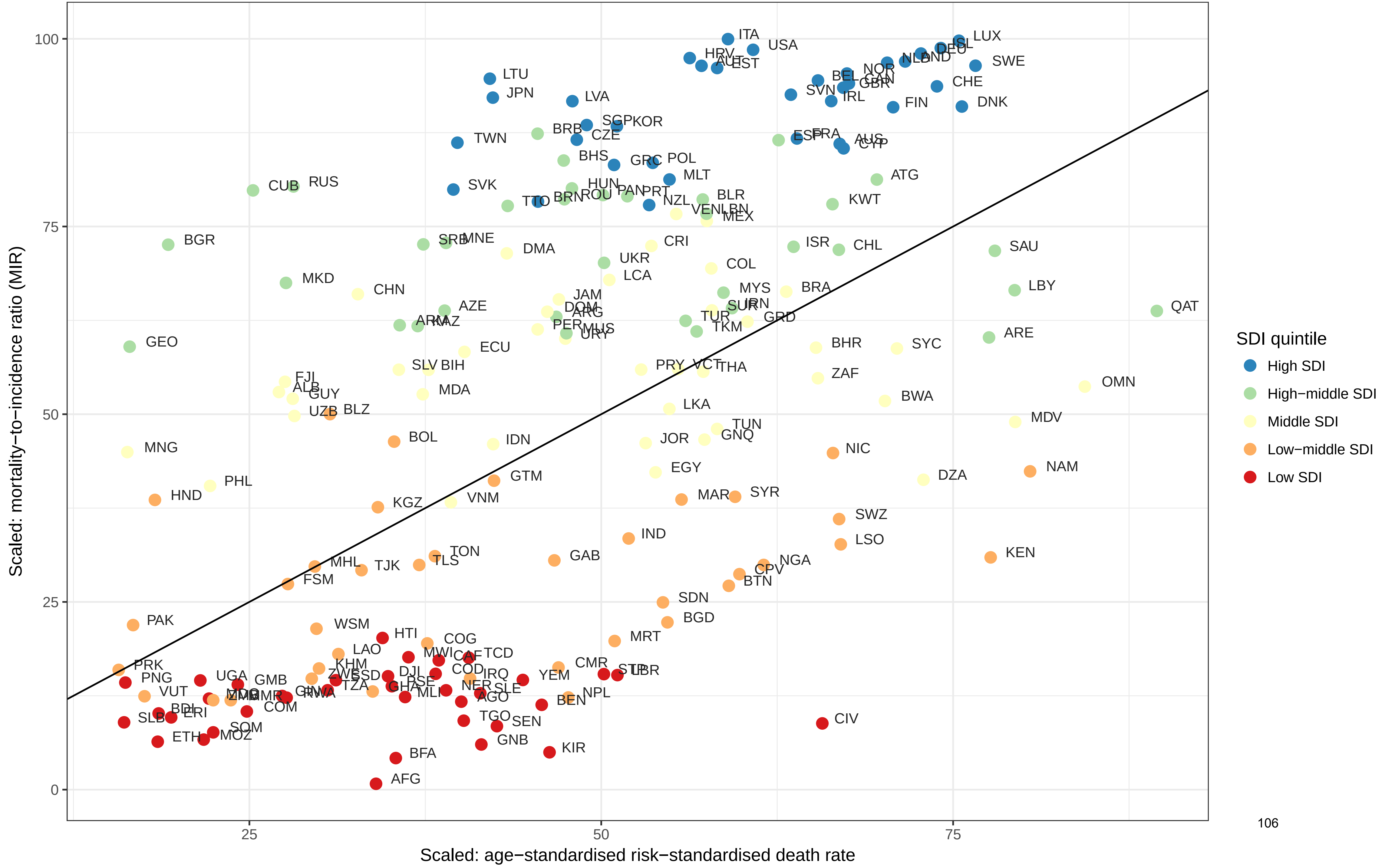

Supplementary figure 2. Comparing scaled MIRs to age−standised risk−standardised death rates for colon and rectum cancer, 2016. MIRs = mortality-to-incidence ratios. SDI = Socio-demographic index.

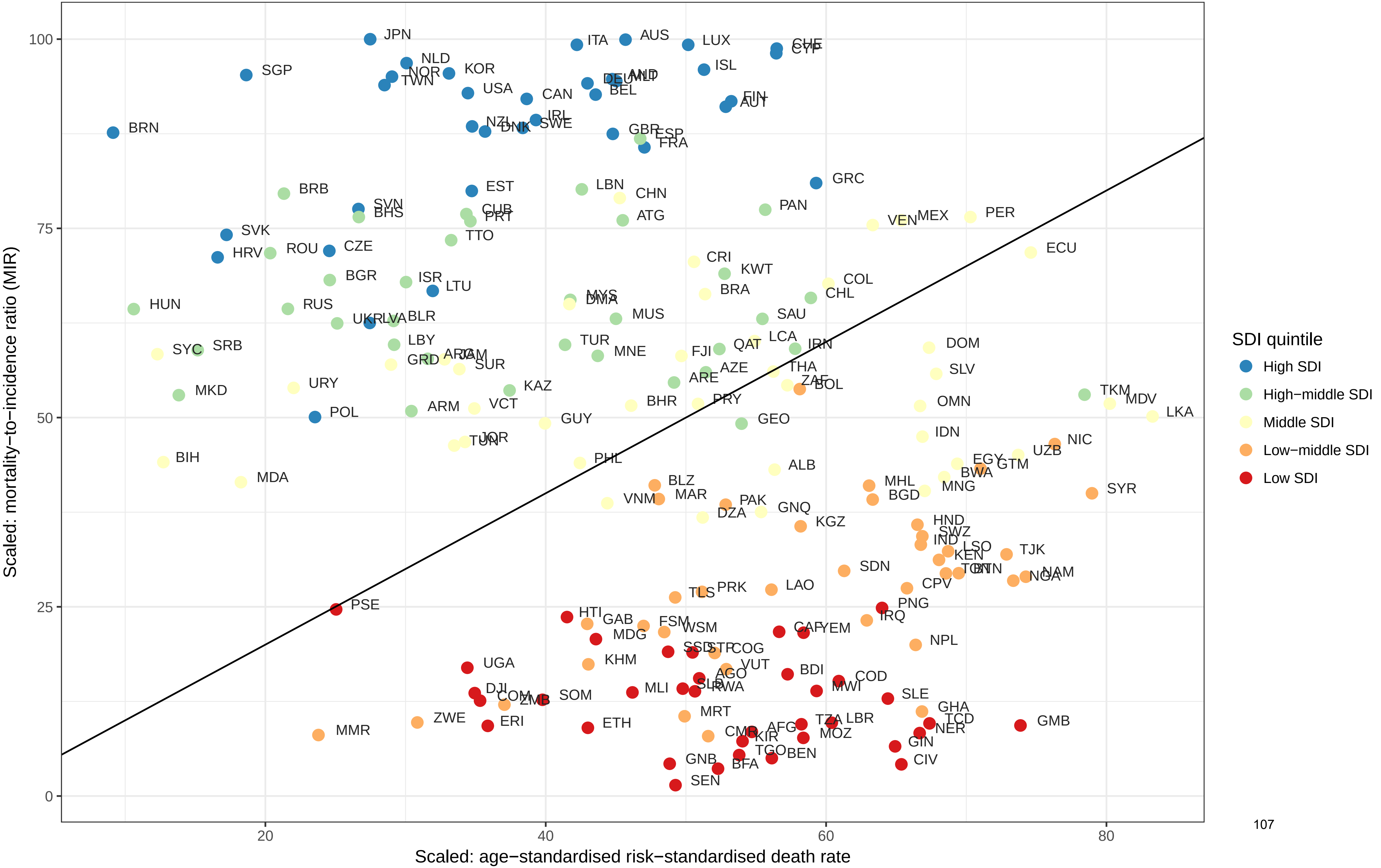

Supplementary figure 2. Comparing scaled MIRs to age−standised risk−standardised death rates for testicular cancer, 2016. MIRs = mortality-to-incidence ratios. SDI = Socio-demographic index.

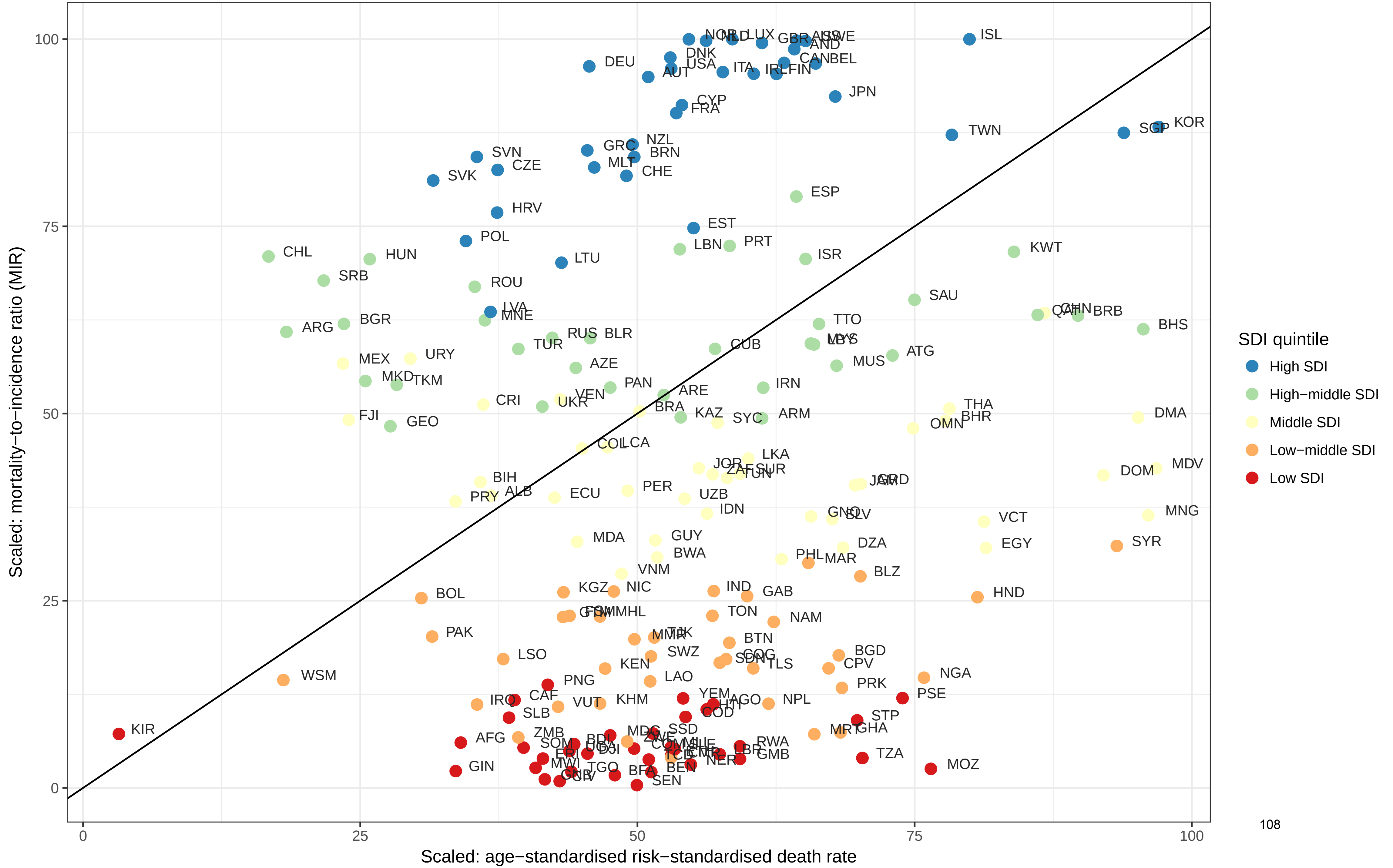

Supplementary figure 2. Comparing scaled MIRs to age−standised risk−standardised death rates for Hodgkin lymphoma, 2016. MIRs = mortality-to-incidence ratios. SDI = Socio-demographic index.

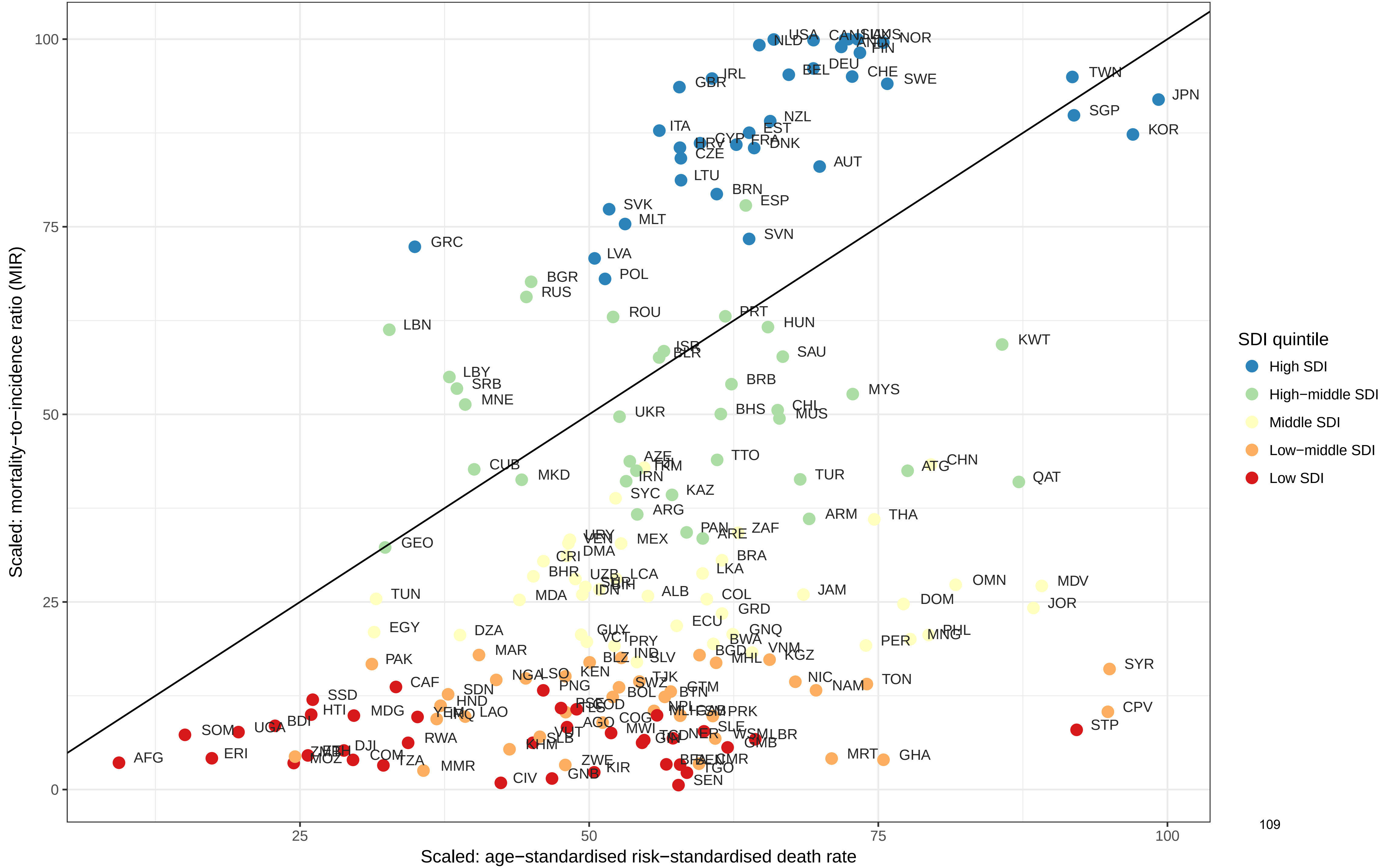

Supplementary figure 2. Comparing scaled MIRs to age−standised risk−standardised death rates for leukaemia, 2016.

MIRs = mortality-to-incidence ratios. SDI = Socio-demographic index.

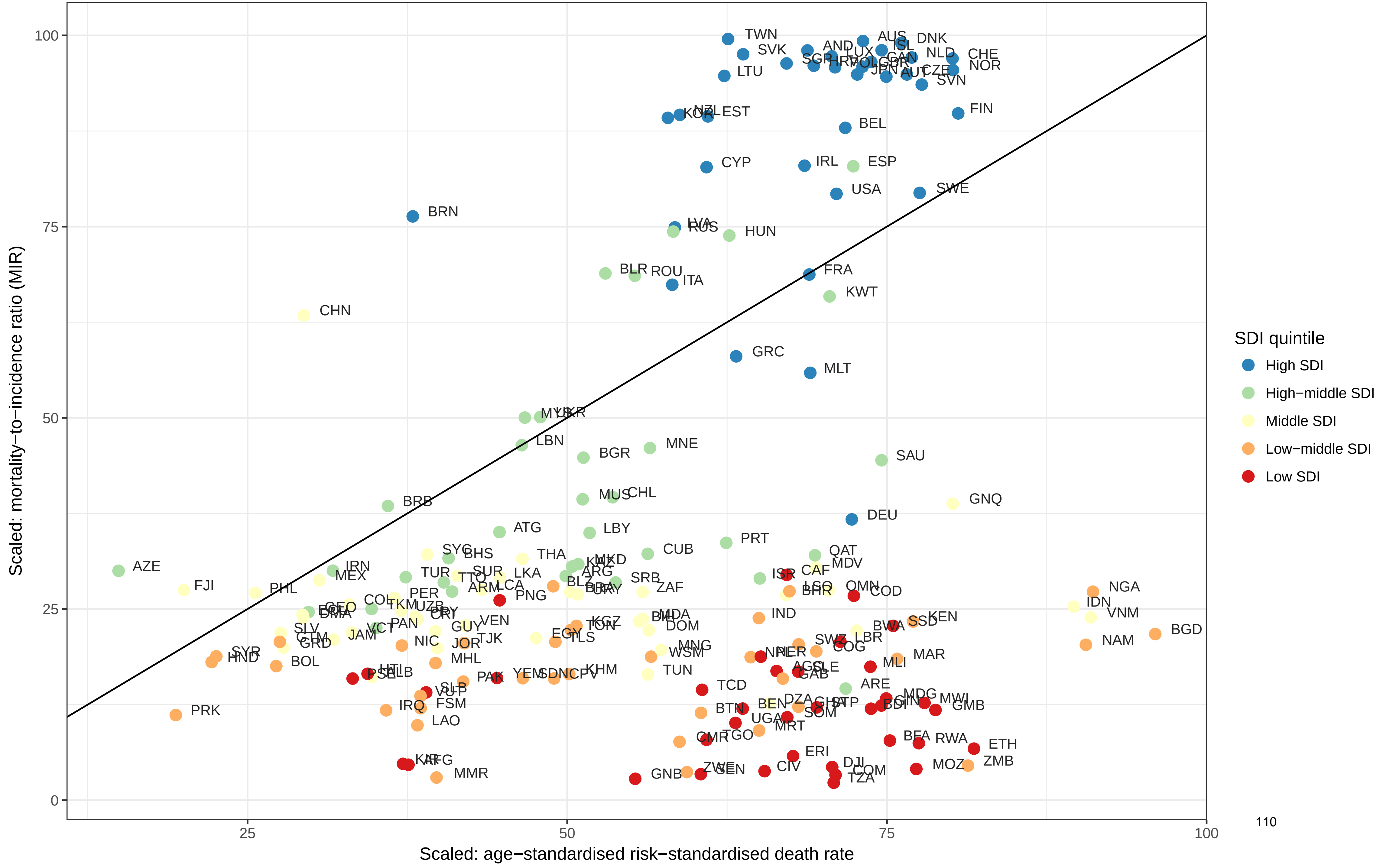

Supplementary figure 2. Comparing scaled MIRs to age−standised risk−standardised death rates for non−melanoma skin cancer (squamous−cell carcinoma), 2016. MIRs = mortality-to-incidence ratios. SDI = Socio-demographic index.

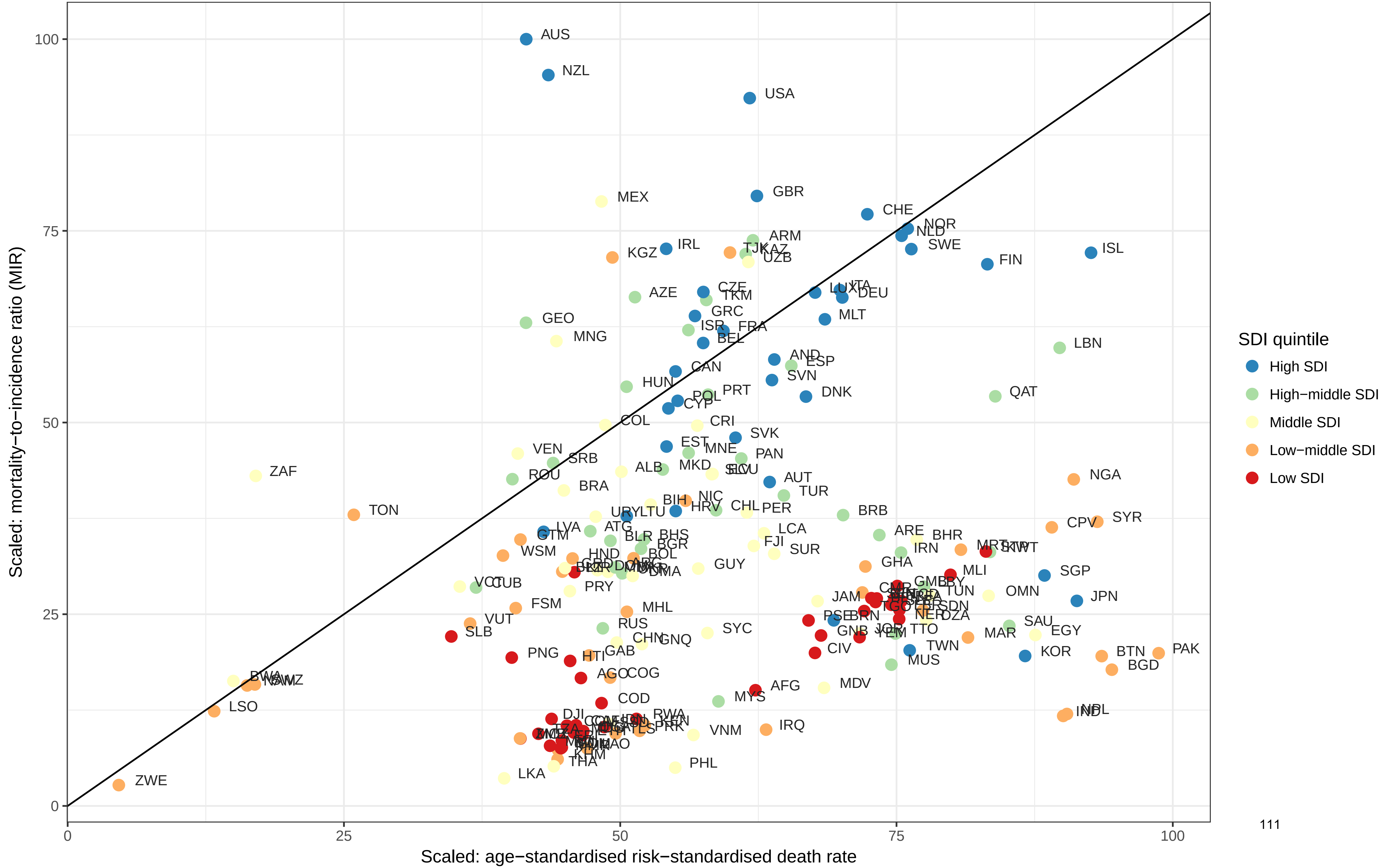

Supplementary figure 3. Comparing PCA-derived cause weights for the HAQ Index in GBD 2016 and GBD 2016. PCA = principal components analysis. HAQ Index = Healthcare Access and Quality Index. GBD = Global Burden of Disease.

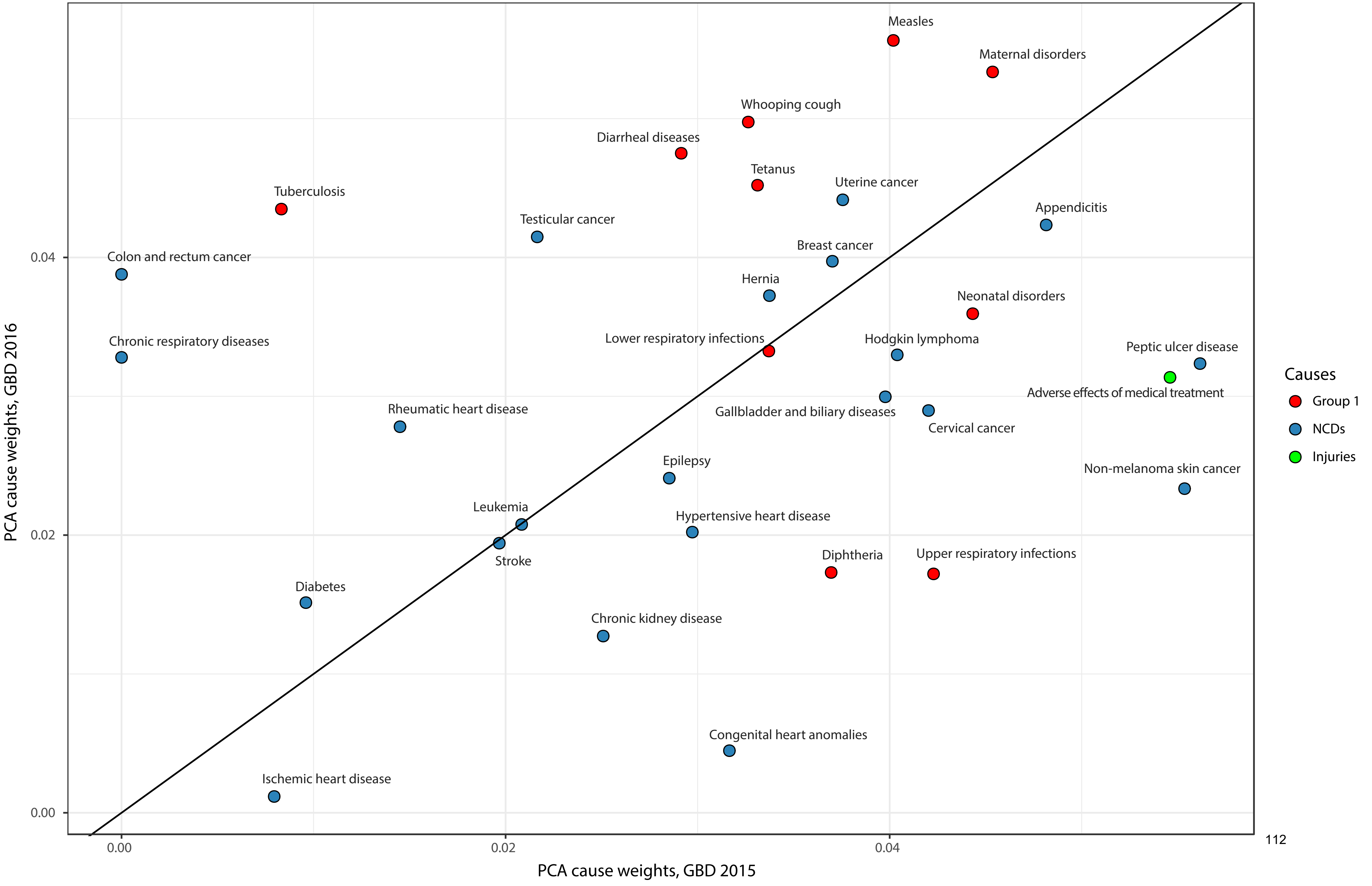

Supplementary figure 4. Map of HAQ Index values, by decile, in 1990 (A). Deciles were based on the distribution of HAQ Index values in 2016, as found in figure 1 of the main text, and then were applied for 1990 and 2000. HAQ Index = Healthcare Access and Quality Index. ATG=Antigua and Barbuda. VCT=Saint Vincent and the Grenadines. LCA=Saint Lucia. TTO=Trinidad and Tobago. TLS=Timor-Leste. FSM=Federated States of Micronesia.

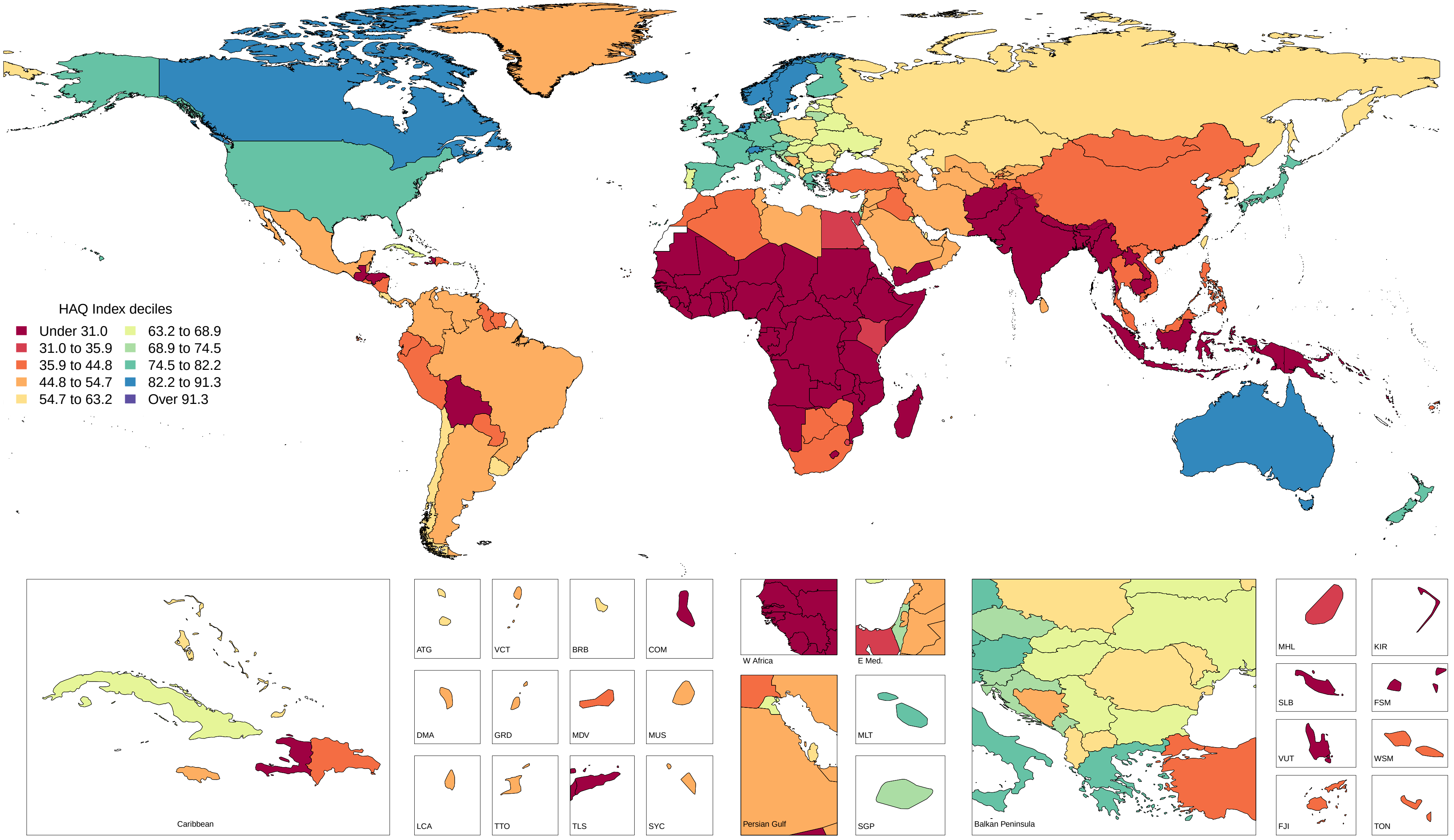

Supplementary figure 4. Map of HAQ Index values, by decile, in 2000 (B). Deciles were based on the distribution of HAQ Index values in 2016, as found in figure 1 of the main text, and then were applied for 1990 and 2000. HAQ Index = Healthcare Access and Quality Index. ATG=Antigua and Barbuda. VCT=Saint Vincent and the Grenadines. LCA=Saint Lucia. TTO=Trinidad and Tobago. TLS=Timor-Leste. FSM=Federated States of Micronesia.

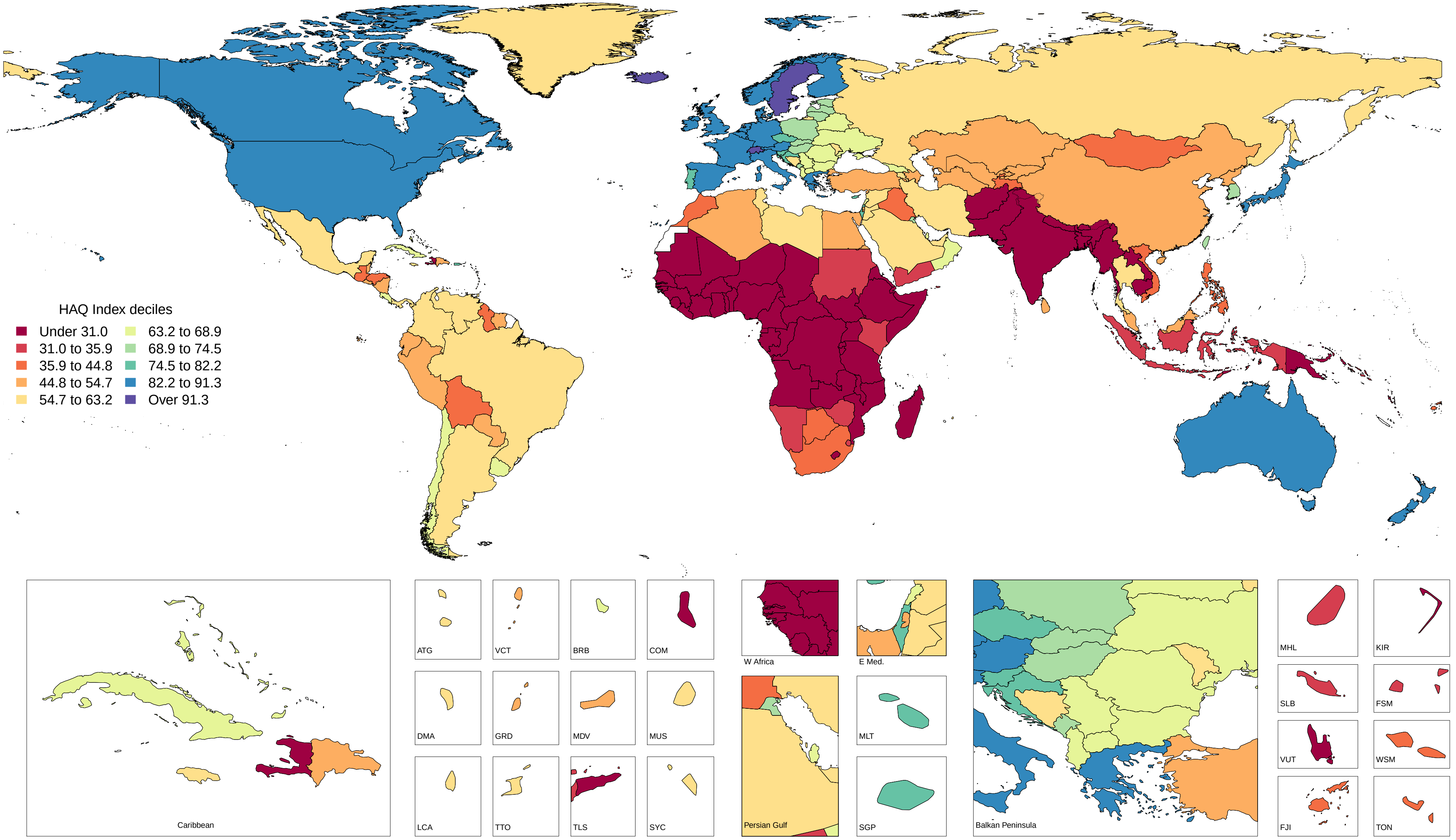

Supplementary figure 5. Map of subnational HAQ Index values, by decile, in 1990, 2000, and 2016 for Japan (A). Deciles were based on the distribution of HAQ Index values for countries and territories in 2016, as found in figure 1 of the main text, and then were applied for subnational locations over time. HAQ Index = Healthcare Access and Quality Index.

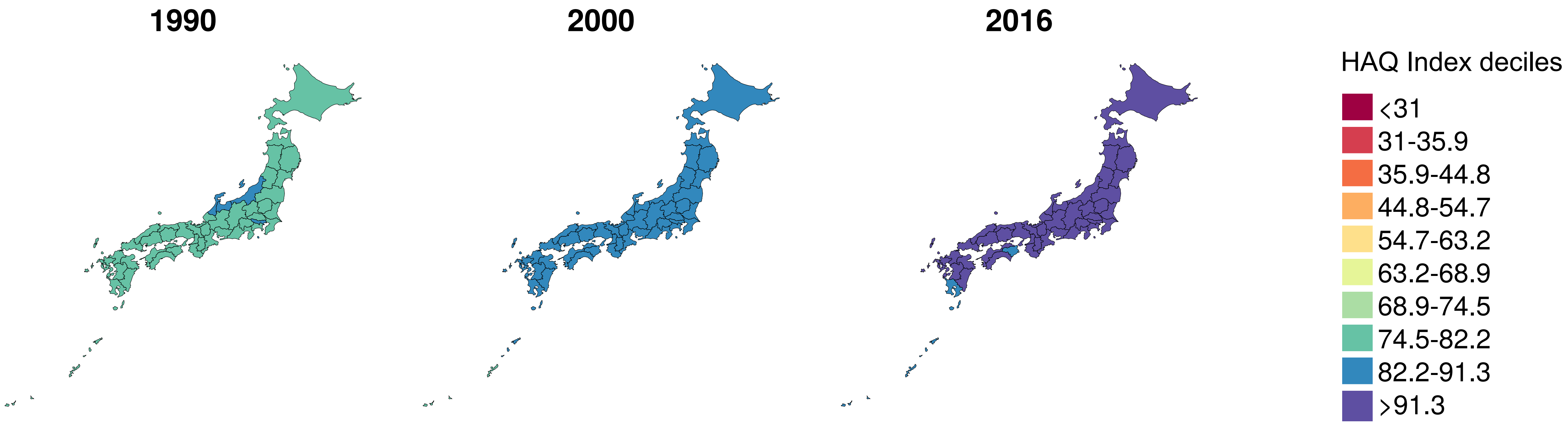

Supplementary figure 5. Map of subnational HAQ Index values, by decile, in 1990, 2000, and 2016 for England (B). Deciles were based on the distribution of HAQ Index values for countries and territories in 2016, as found in figure 1 of the main text, and then were applied for subnational locations over time. HAQ Index = Healthcare Access and Quality Index.

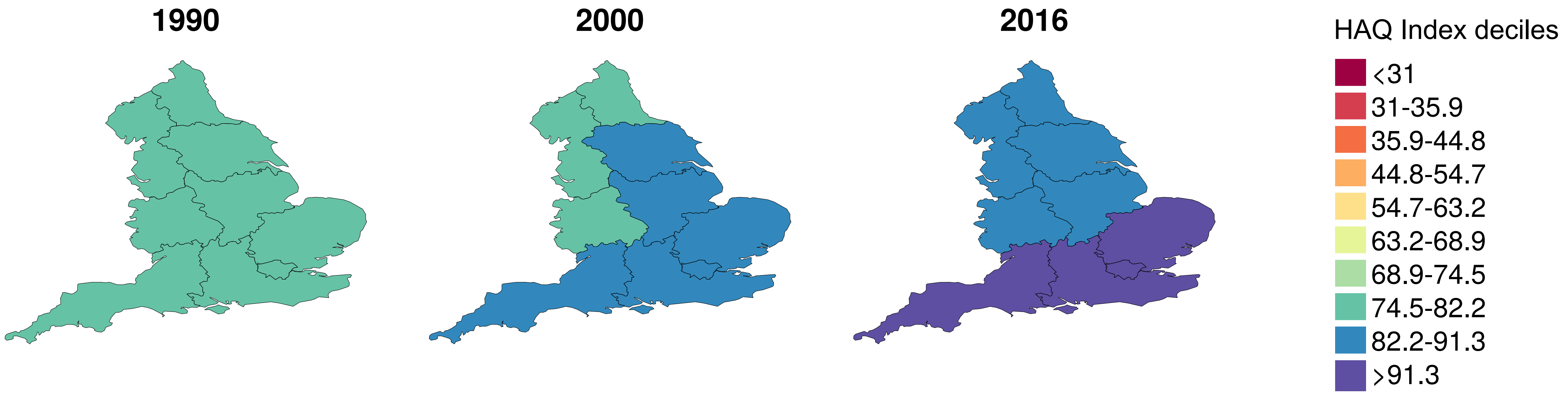

Supplementary figure 5. Map of subnational HAQ Index values, by decile, in 1990, 2000, and 2016 for the USA (C). Deciles were based on the distribution of HAQ Index values for countries and territories in 2016, as found in figure 1 of the main text, and then were applied for subnational locations over time. HAQ Index = Healthcare Access and Quality Index.

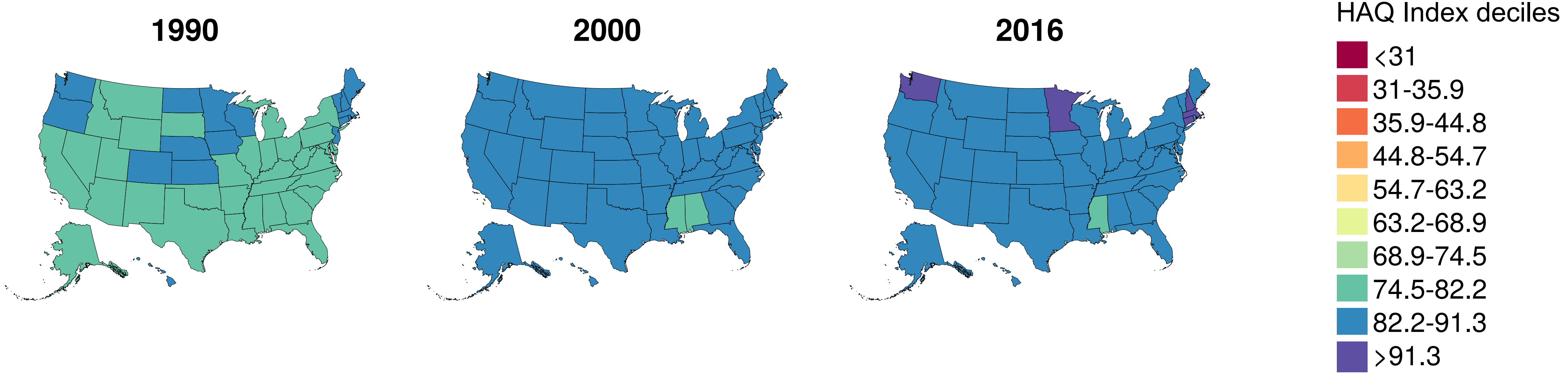

Supplementary figure 5. Map of subnational HAQ Index values, by decile, in 1990, 2000, and 2016 for China (D). Deciles were based on the distribution of HAQ Index values for countries and territories in 2016, as found in figure 1 of the main text, and then were applied for subnational locations over time. HAQ Index = Healthcare Access and Quality Index.

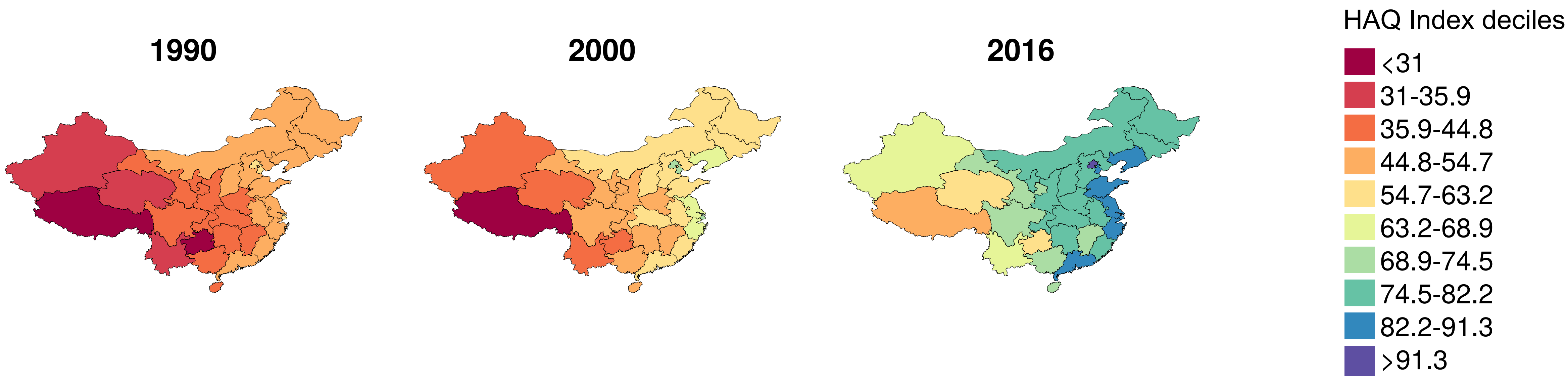

Supplementary figure 5. Map of subnational HAQ Index values, by decile, in 1990, 2000, and 2016 for Mexico (E). Deciles were based on the distribution of HAQ Index values for countries and territories in 2016, as found in figure 1 of the main text, and then were applied for subnational locations over time. HAQ Index = Healthcare Access and Quality Index.

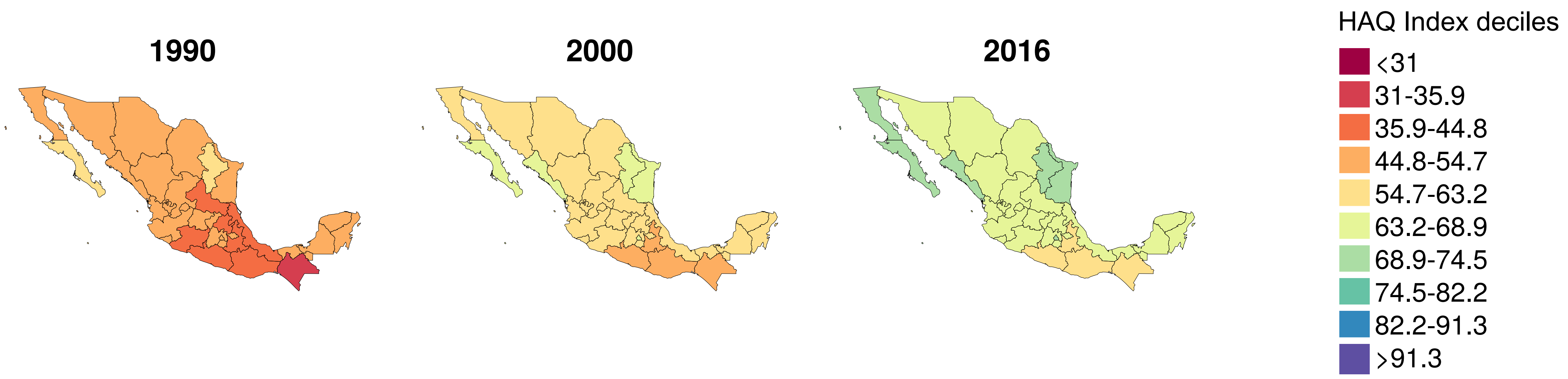

Supplementary figure 5. Map of subnational HAQ Index values, by decile, in 1990, 2000, and 2016 for Brazil (F). Deciles were based on the distribution of HAQ Index values for countries and territories in 2016, as found in figure 1 of the main text, and then were applied for subnational locations over time. HAQ Index = Healthcare Access and Quality Index.

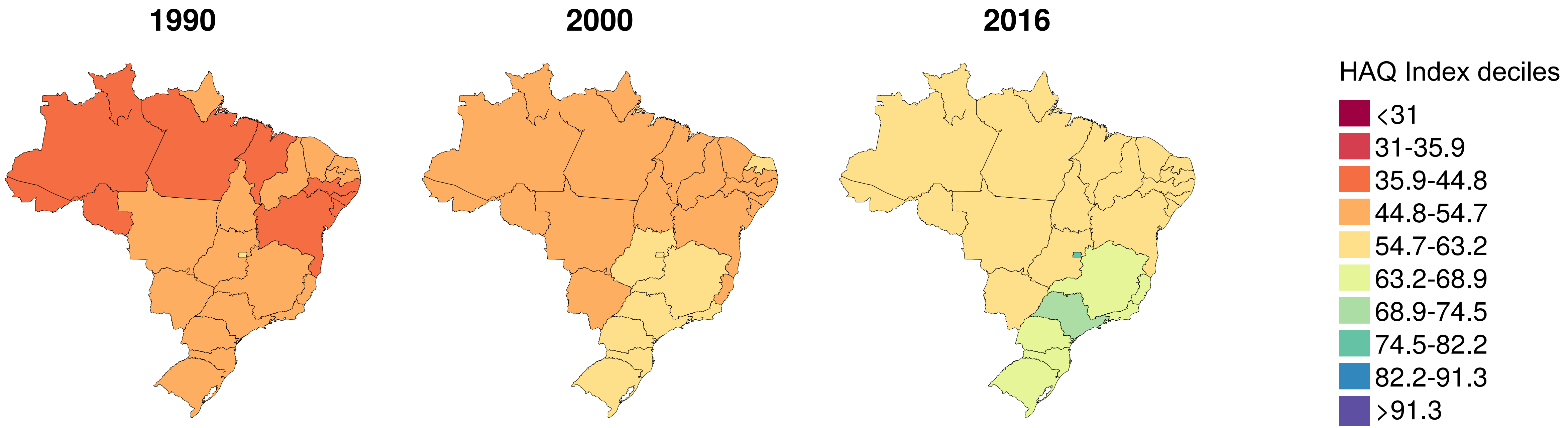

Supplementary figure 5. Map of subnational HAQ Index values, by decile, in 1990, 2000, and 2016 for India (G). Deciles were based on the distribution of HAQ Index values for countries and territories in 2016, as found in figure 1 of the main text, and then were applied for subnational locations over time. HAQ Index = Healthcare Access and Quality Index.

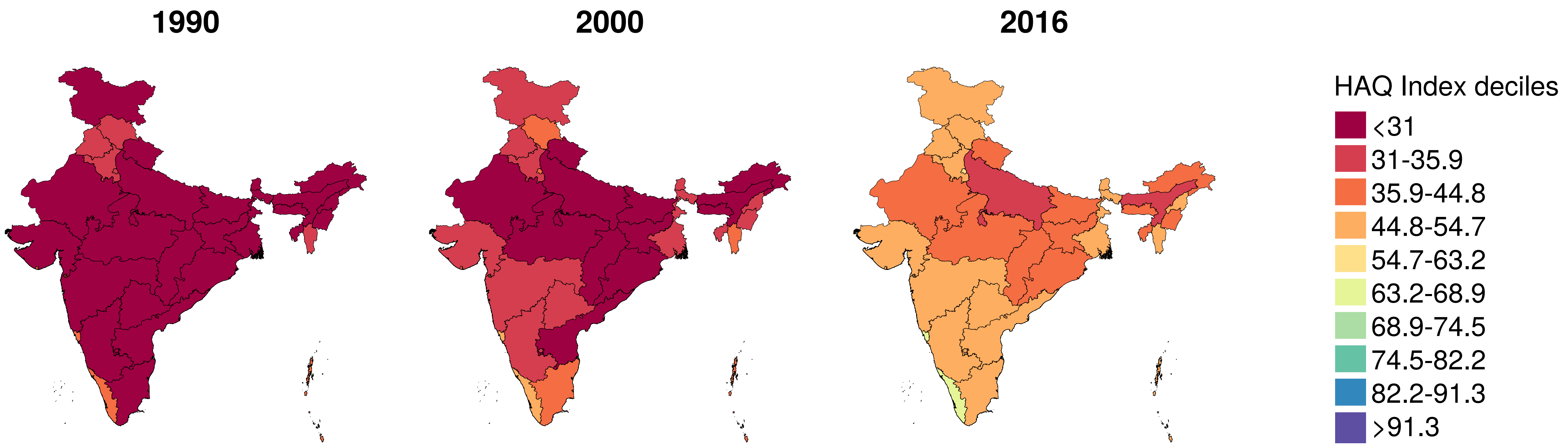

Supplementary figure 6. Map of subnational HAQ Index values, in 1990, 2000, and 2016, for Japan (A). Scales were based on the range of HAQ Index values observed across subnational locations from 1990–2016. Due to similarities in their HAQ Index range, the same scales were used for Japan, England, and the USA, and then for Mexico and Brazil. HAQ Index = Healthcare Access and Quality Index.

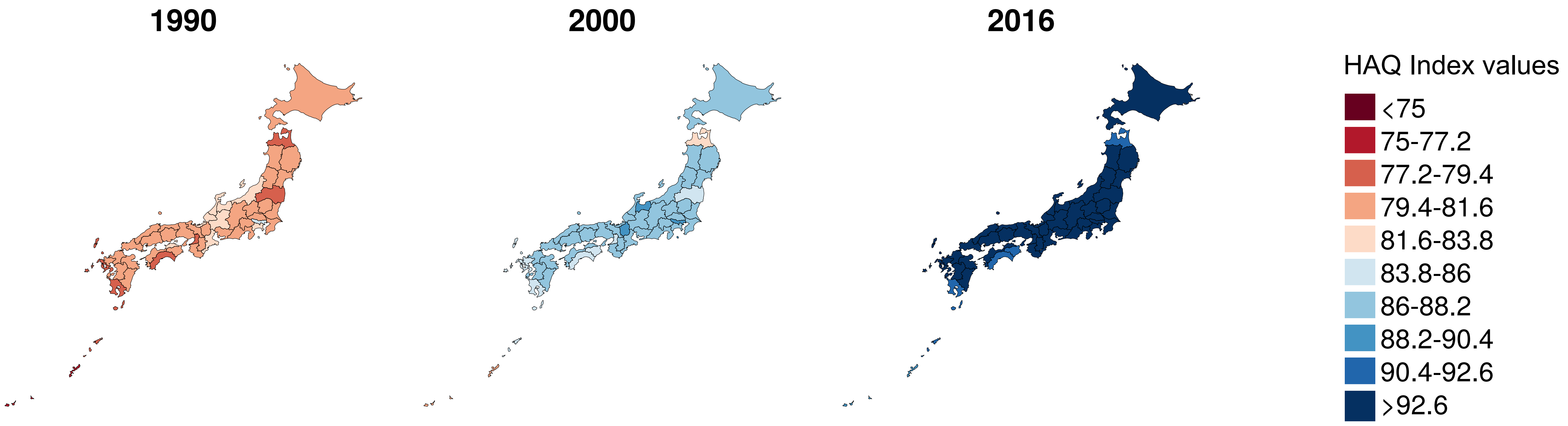

Supplementary figure 6. Map of subnational HAQ Index values, in 1990, 2000, and 2016, for England (B). Scales were based on the range of HAQ Index values observed across subnational locations from 1990–2016. Due to similarities in their HAQ Index range, the same scales were used for Japan, England, and the USA, and then for Mexico and Brazil. HAQ Index = Healthcare Access and Quality Index.

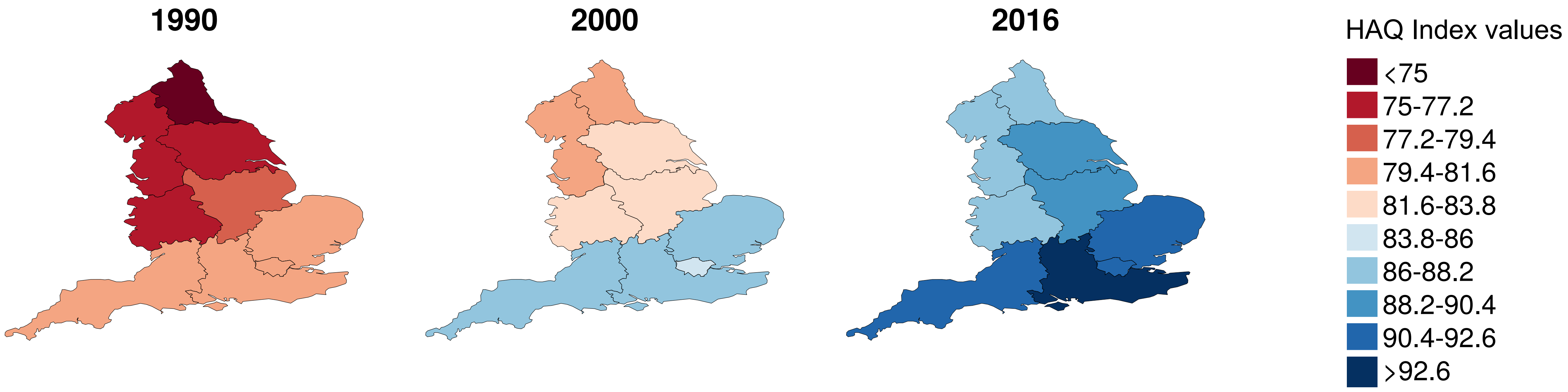

Supplementary figure 6. Map of subnational HAQ Index values, in 1990, 2000, and 2016, for the USA (C). Scales were based on the range of HAQ Index values observed across subnational locations from 1990–2016. Due to similarities in their HAQ Index range, the same scales were used for Japan, England, and the USA, and then for Mexico and Brazil. HAQ Index = Healthcare Access and Quality Index.

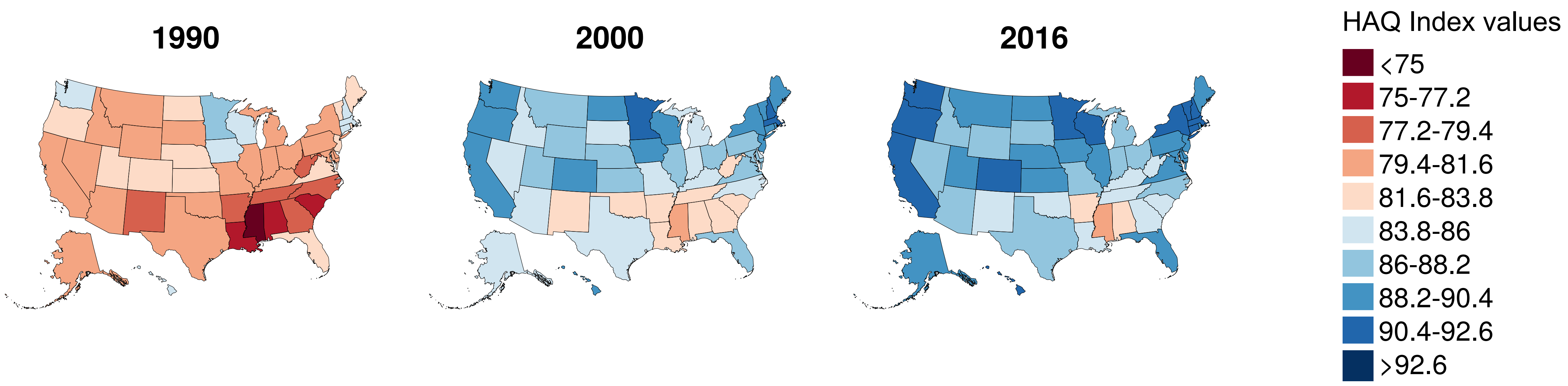

Supplementary figure 6. Map of subnational HAQ Index values, in 1990, 2000, and 2016, for China (D). Scales were based on the range of HAQ Index values observed across subnational locations from 1990–2016. Due to similarities in their HAQ Index range, the same scales were used for Japan, England, and the USA, and then for Mexico and Brazil. HAQ Index = Healthcare Access and Quality Index.

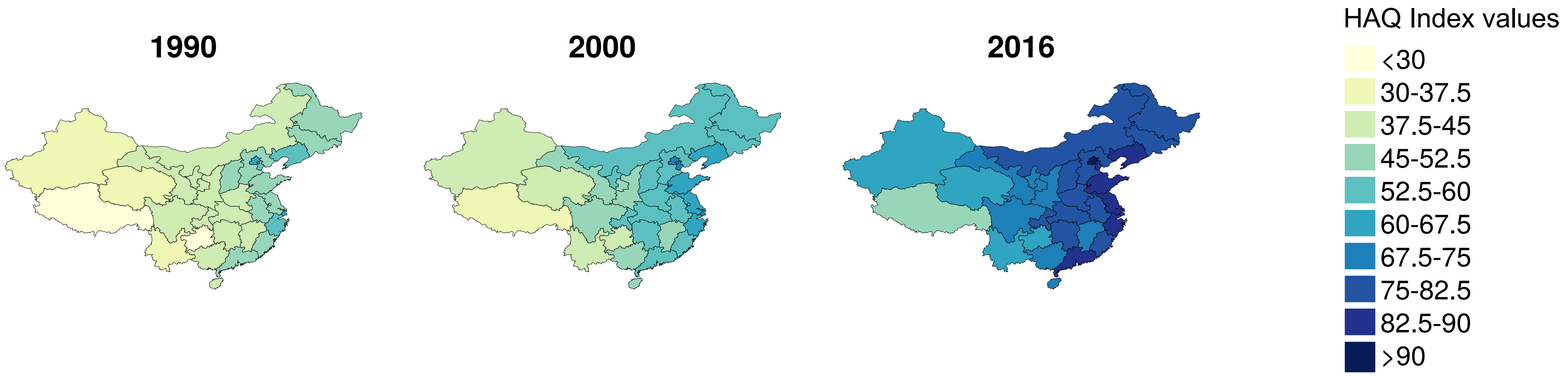

Supplementary figure 6. Map of subnational HAQ Index values, in 1990, 2000, and 2016, for Mexico (E). Scales were based on the range of HAQ Index values observed across subnational locations from 1990–2016. Due to similarities in their HAQ Index range, the same scales were used for Japan, England, and the USA, and then for Mexico and Brazil. HAQ Index = Healthcare Access and Quality Index.

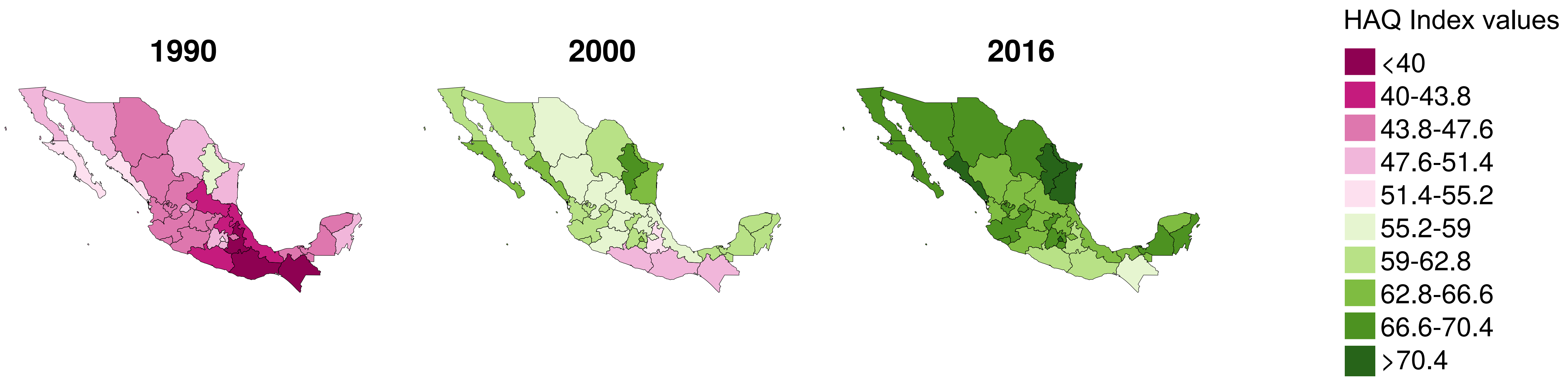

Supplementary figure 6. Map of subnational HAQ Index values, in 1990, 2000, and 2016, for Brazil (F). Scales were based on the range of HAQ Index values observed across subnational locations from 1990–2016. Due to similarities in their HAQ Index range, the same scales were used for Japan, England, and the USA, and then for Mexico and Brazil. HAQ Index = Healthcare Access and Quality Index.

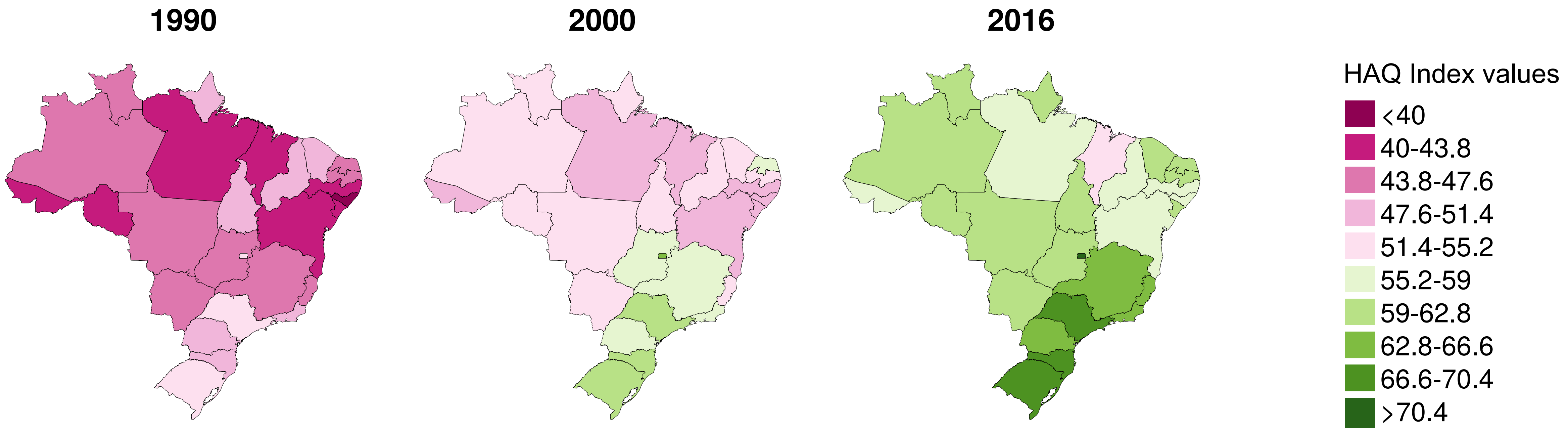

Supplementary figure 6. Map of subnational HAQ Index values, in 1990, 2000, and 2016, for India (G). Scales were based on the range of HAQ Index values observed across subnational locations from 1990–2016. Due to similarities in their HAQ Index range, the same scales were used for Japan, England, and the USA, and then for Mexico and Brazil. HAQ Index = Healthcare Access and Quality Index.

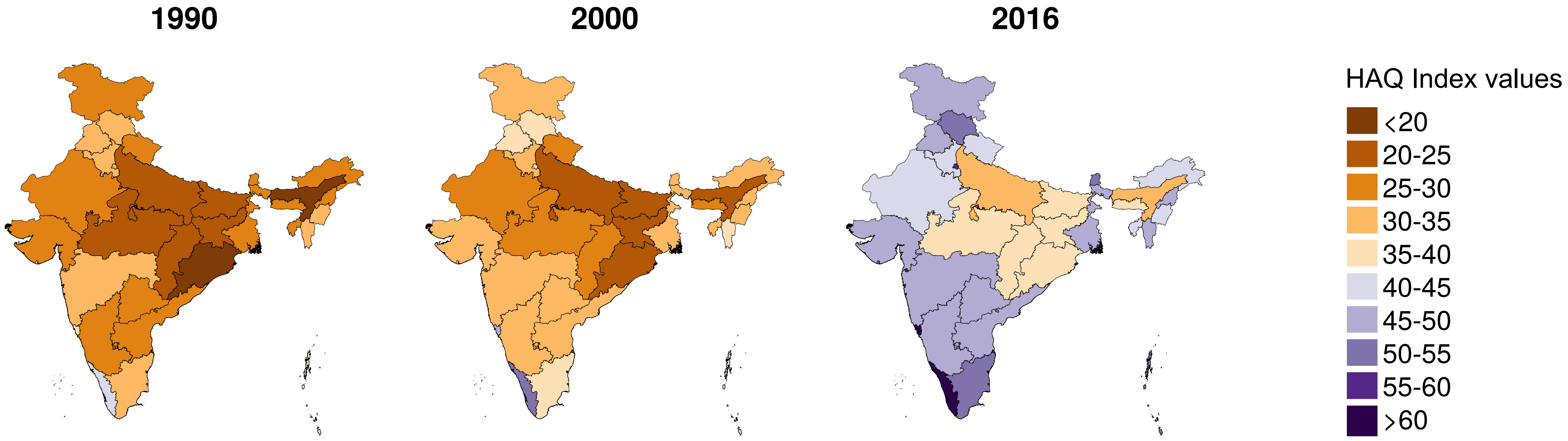

Supplementary figure 7. Absolute change on the HAQ Index, by SDI quintile, from 1990 to 2016. Countries and territories are colour-coded by their SDI quintile, and are abbreviated according to the ISO3 code. HAQ Index = Healthcare Access and Quality Index. SDI = Socio-demographic Index.

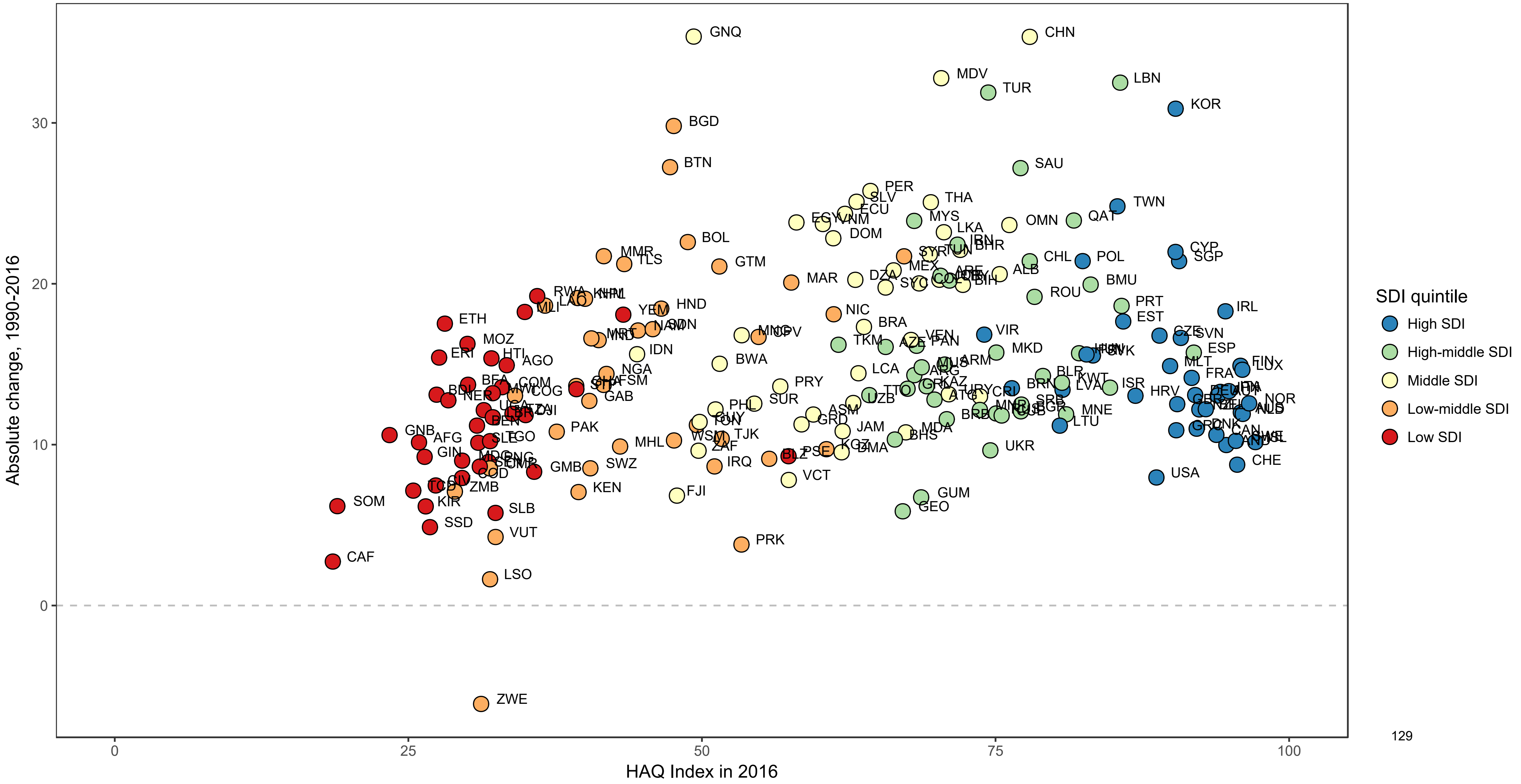

Supplementary figure 8. Comparing the HAQ Index, by SDI quintile, from 1990–2016 (A). Countries and territories are colour-coded by their SDI quintile, and are abbreviated according to the ISO3 code. HAQ Index = Healthcare Access and Quality Index. SDI = Socio-demographic Index.

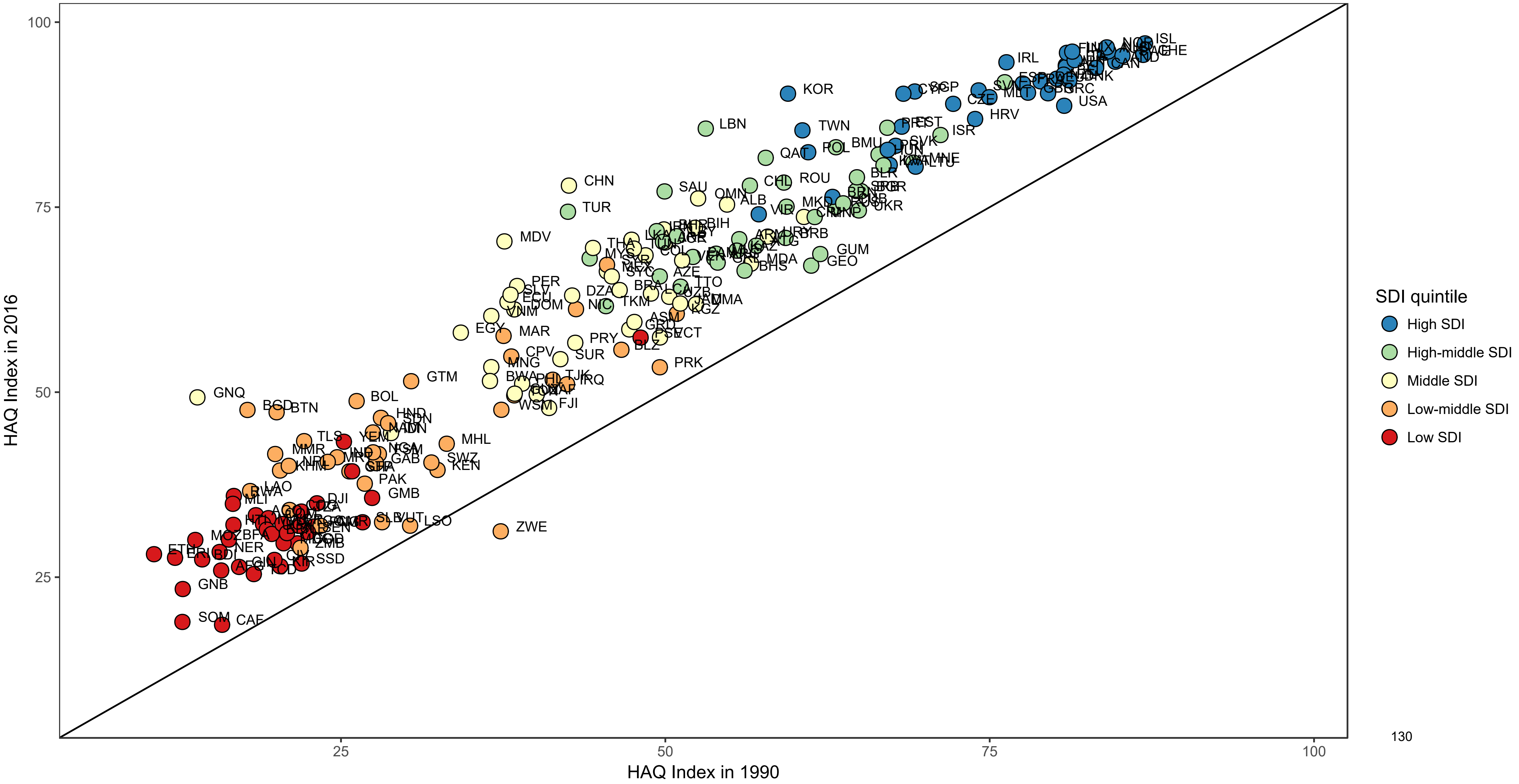

Supplementary figure 8. Comparing the HAQ Index, by SDI quintile, from 1990–2000 (B). Countries and territories are colour-coded by their SDI quintile, and are abbreviated according to the ISO3 code. HAQ Index = Healthcare Access and Quality Index. SDI = Socio-demographic Index.

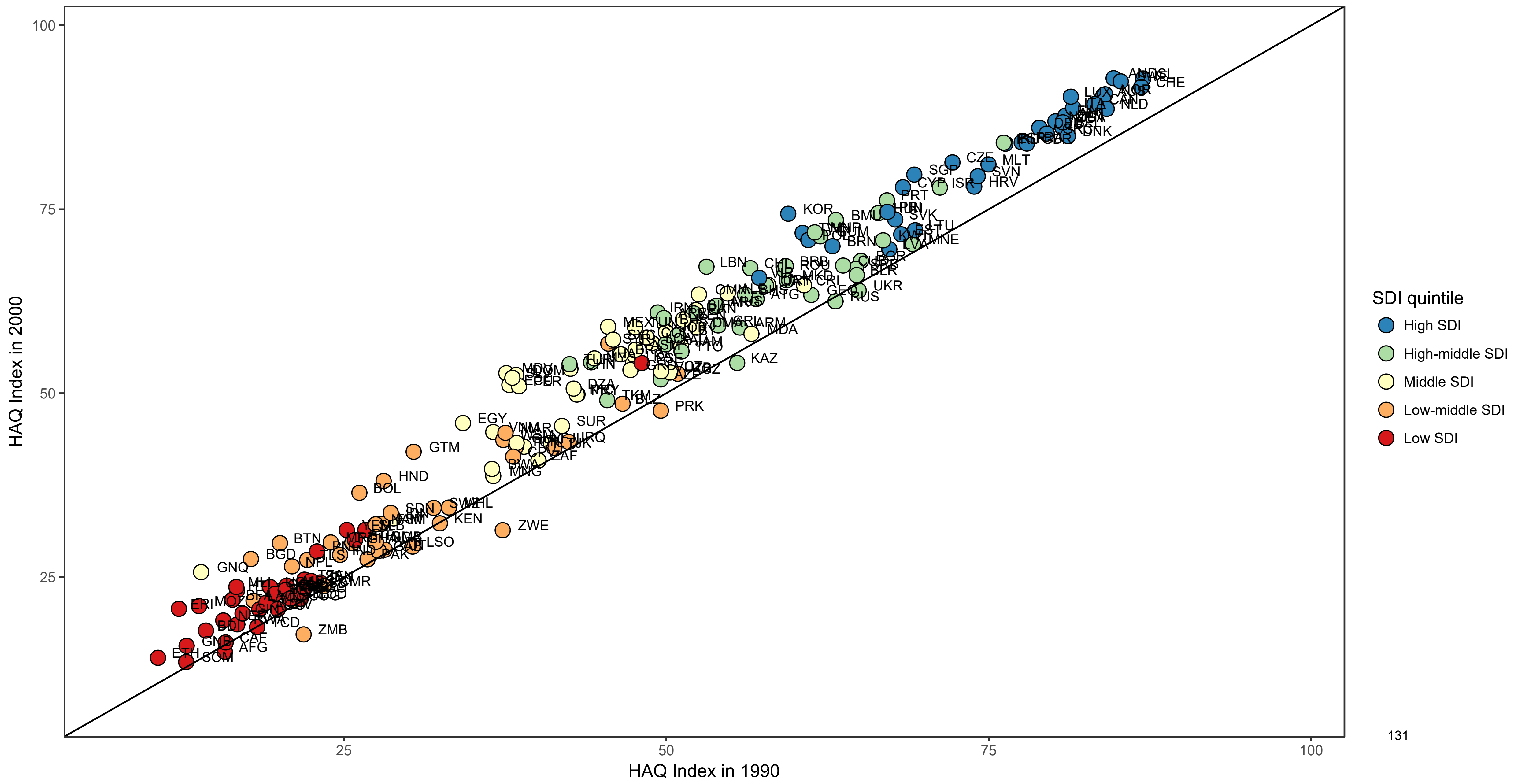

Supplementary figure 8. Comparing the HAQ Index, by SDI quintile, from 2000–2016 (C). Countries and territories are colour-coded by their SDI quintile, and are abbreviated according to the ISO3 code. HAQ Index = Healthcare Access and Quality Index. SDI = Socio-demographic Index.

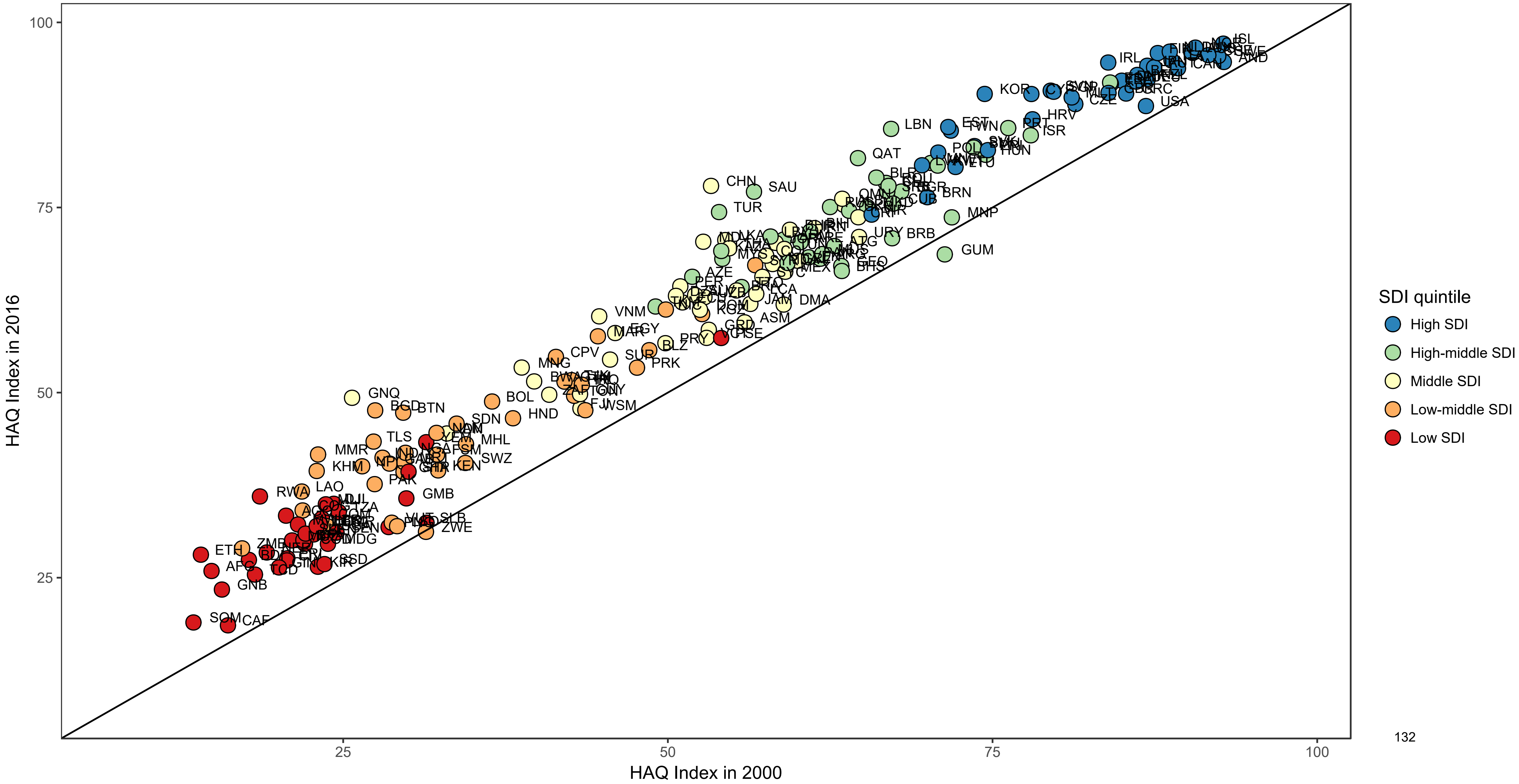

Supplementary figure 9. Annualised rate of change on the HAQ Index, by SDI quintile, from 1990–2016 (A). Countries and territories are colour-coded by their SDI quintile, and are abbreviated according to the ISO3 code. HAQ Index = Healthcare Access and Quality Index. SDI = Socio-demographic Index.

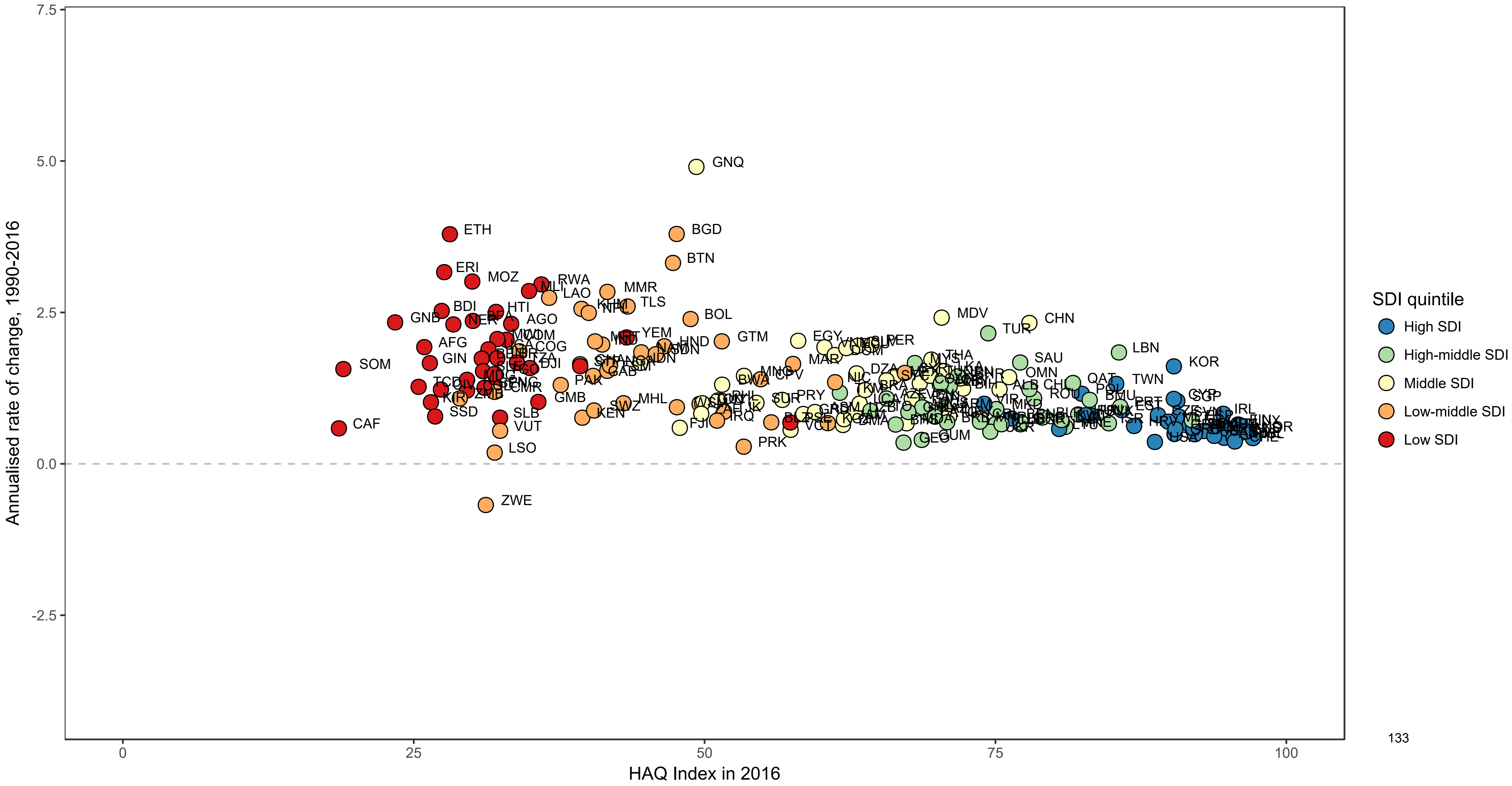

Supplementary figure 9. Annualised rate of change on the HAQ Index, by SDI quintile, from 1990–2000 (B). Countries and territories are colour-coded by their SDI quintile, and are abbreviated according to the ISO3 code. HAQ Index = Healthcare Access and Quality Index. SDI = Socio-demographic Index.

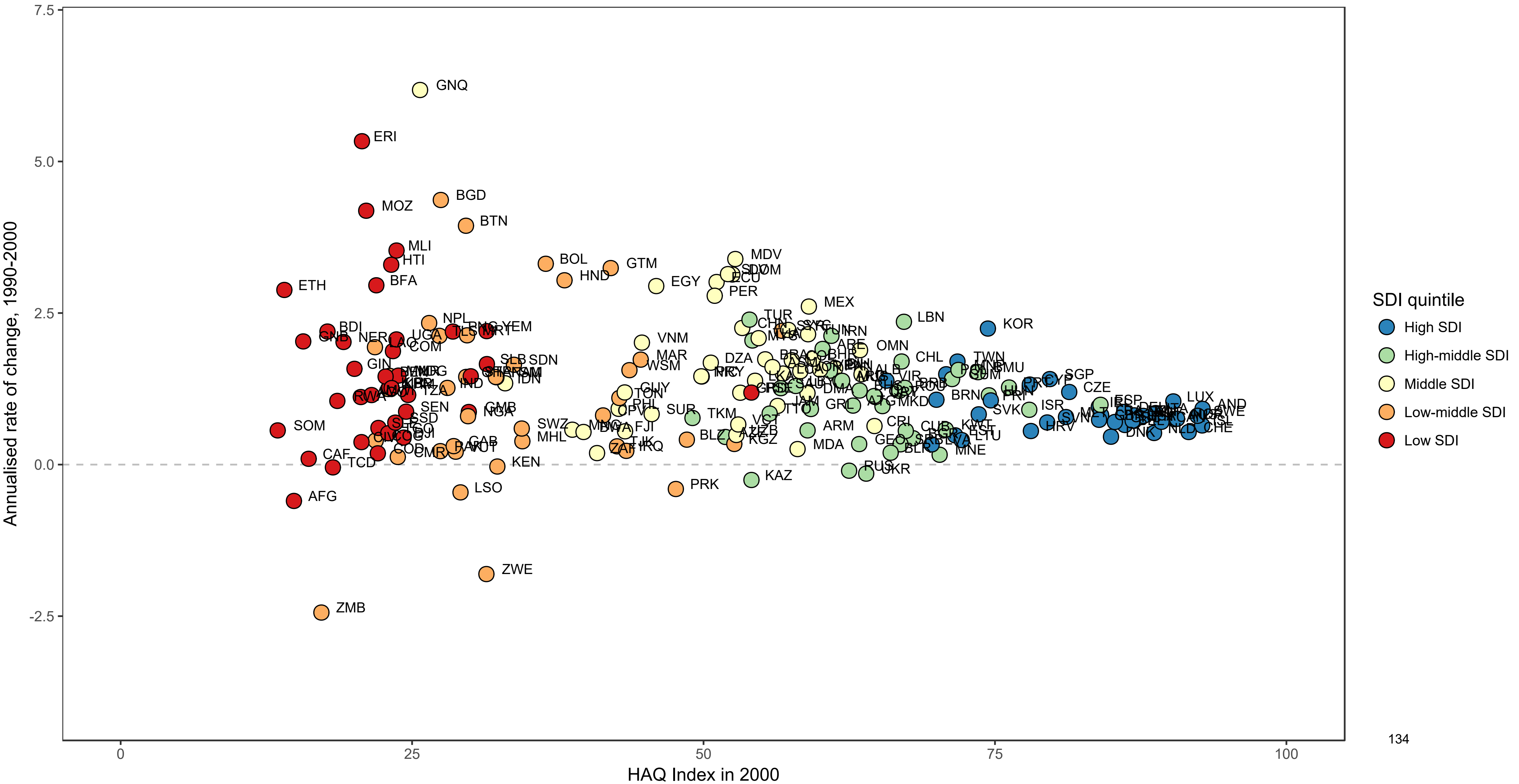

Supplementary figure 9. Annualised rate of change on the HAQ Index, by SDI quintile, from 2000–2016 (C). Countries and territories are colour-coded by their SDI quintile, and are abbreviated according to the ISO3 code. HAQ Index = Healthcare Access and Quality Index. SDI = Socio-demographic Index.

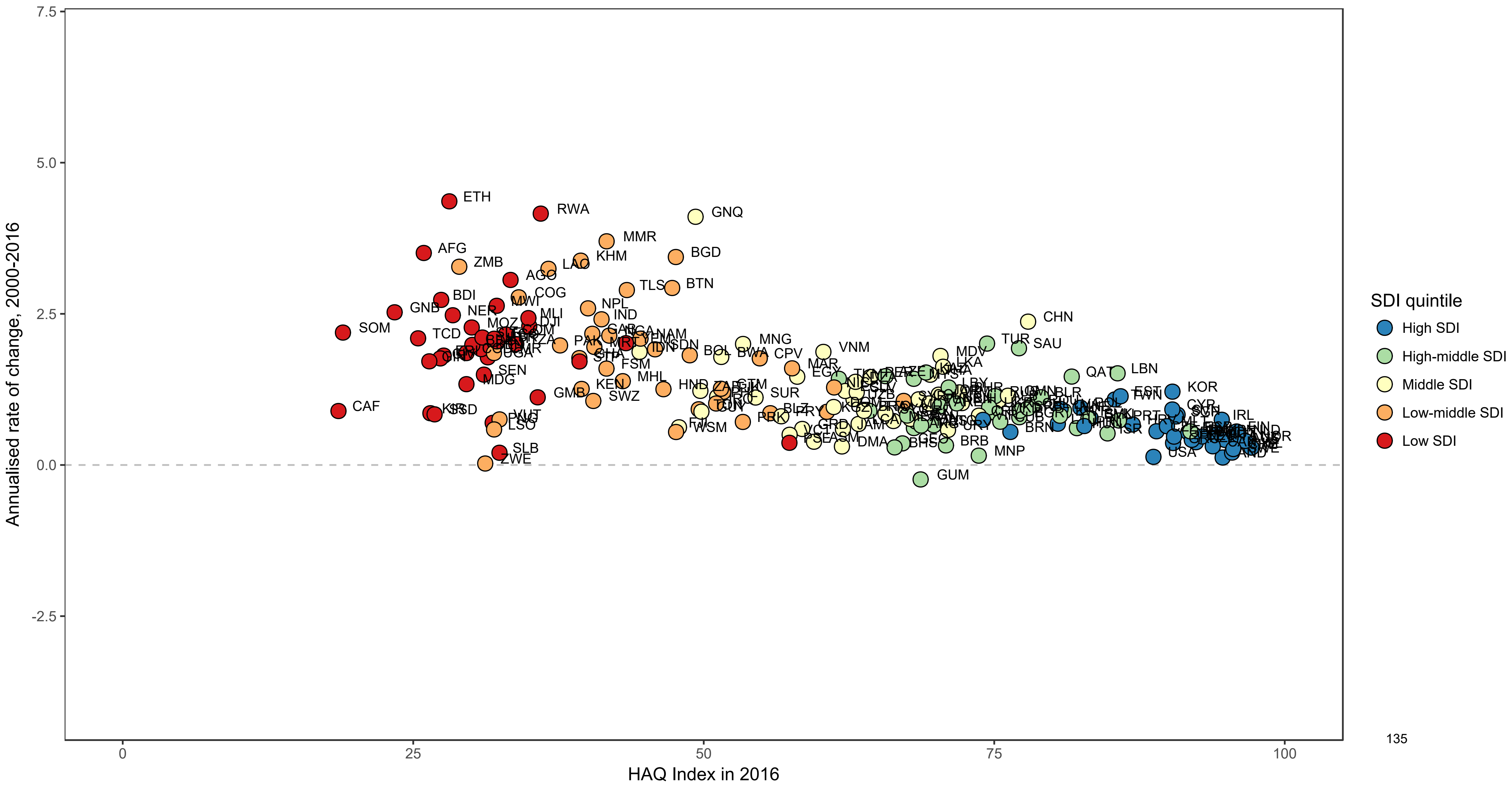

Supplementary figure 10. Absolute change on the HAQ Index and 32 individual causes, by country or territory, from 2000–2016. Countries are ranked by the difference in their HAQ Index in 2016 from their HAQ Index in 2000. Positive values represent improvements for overall the overall index and individual causes from 2000–2016, while negative values represent worsening performance during that time. HAQ Index = Healthcare Access and Quality Index.

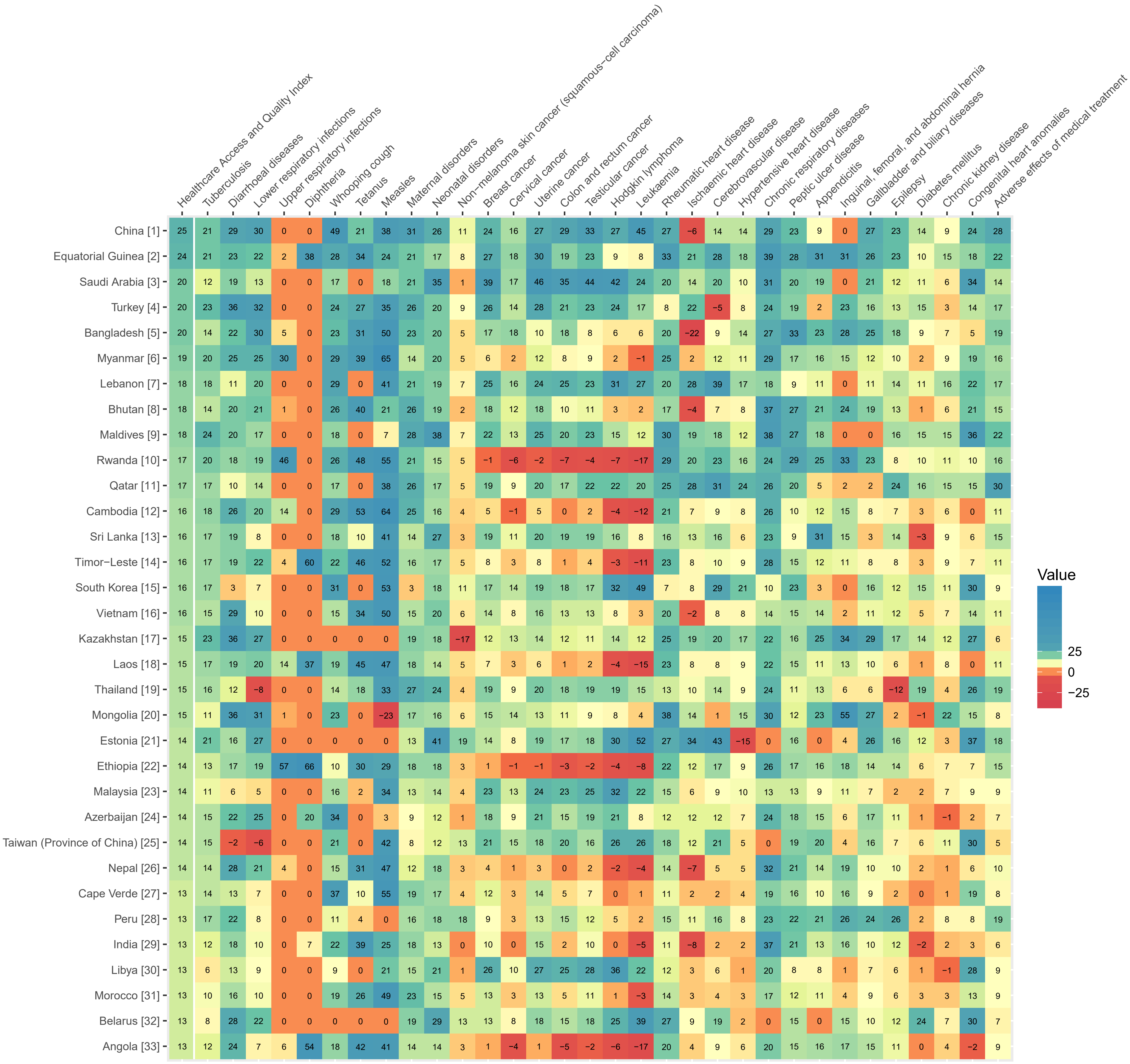

Supplementary figure 10. Absolute change on the HAQ Index and 32 individual causes, by country or territory, from 2000–2016. Countries are ranked by the difference in their HAQ Index in 2016 from their HAQ Index in 2000. Positive values represent improvements for overall the overall index and individual causes from 2000–2016, while negative values represent worsening performance during that time. HAQ Index = Healthcare Access and Quality Index.

	Healthcare Access and Quality Index	Tuberculosis	Diarrhoeal diseases	Lower respiratory infections	Upper respiratory infections	Diphtheria	Whooping cough	Tetanus	Measles	Maternal disorders	Neonatal disorders	Non−melanoma skin cancer (squamous−cell carcinoma)	Breast cancer	Cervical cancer	Uterine cancer	Colon and rectum cancer	Testicular cancer	Hodgkin lymphoma	Leukaemia	Rheumatic heart disease	Ischaemic heart disease	Cerebrovascular disease	Hypertensive heart disease	Chronic respiratory diseases	Peptic ulcer disease	Appendicitis	Inguinal, femoral, and abdominal hernia	Gallbladder and biliary diseases	Epilepsy	Diabetes mellitus	Chronic kidney disease	Congenital heart anomalies	Adverse effects of medical treatment
Oman [34]	13	8	8	6	0	0	1	14	1	27	19	2	36	19	40	28	33	15	3	5	10	8	10	15	10	5	0	0	10	0	4	13	8
Bahrain [35]	13	15	11	8	0	0	10	0	0	15	16	4	16	9	16	13	14	12	4	19	31	23	11	14	24	20	26	28	11	8	13	20	9
Turkmenistan [36]	13	13	31	27	0	0	−11	0	0	13	12	0	20	9	24	16	23	22	5	21	4	2	5	30	12	20	24	15	9	0	−3	−8	5
Russia [37]	13	11	22	11	0	0	0	0	0	12	24	12	10	11	13	12	13	20	38	28	19	25	7	12	9	13	21	16	20	16	10	32	7
Algeria [38]	12	13	14	9	0	0	27	0	20	17	15	3	20	7	22	13	18	10	2	16	12	8	0	17	13	13	4	12	7	3	2	18	12
Namibia [39]	12	14	4	11	0	0	14	19	4	20	7	7	13	4	17	7	10	2	−2	23	25	26	13	9	23	18	23	18	13	12	13	5	14
Cyprus [40]	12	11	3	17	0	0	1	0	0	12	27	20	18	15	15	16	14	24	41	18	25	29	13	18	17	1	9	13	10	11	9	28	14
Bolivia [41]	12	14	21	11	0	0	17	30	0	11	14	1	10	3	15	14	10	1	−2	13	7	13	5	25	18	17	20	13	14	4	0	9	14
Congo (Brazzaville) [42]	12	14	3	10	4	33	17	41	36	9	10	4	5	−1	6	1	4	−2	−9	16	16	15	9	14	16	14	21	12	10	8	8	5	12
Egypt [43]	12	8	9	19	0	0	20	18	35	14	23	0	16	7	17	10	13	7	5	9	−7	4	3	18	9	7	8	5	6	−2	0	12	10
Sudan [44]	12	13	17	7	2	1	12	27	59	13	7	4	10	4	8	2	5	−2	−10	16	9	10	5	11	15	11	11	12	6	2	7	0	9
Nigeria [45]	12	16	7	11	14	64	12	29	29	10	8	5	8	3	9	−1	−1	−4	−8	18	10	12	12	12	24	15	19	12	3	8	7	0	9
Jordan [46]	12	15	13	12	0	0	21	0	4	15	12	4	17	10	20	14	16	10	5	12	16	21	3	15	23	20	0	9	8	12	4	15	15
Yemen [47]	12	11	17	12	1	0	3	29	61	15	15	0	7	3	5	1	2	−2	−5	14	7	6	1	21	13	10	11	9	7	1	5	10	11
Gabon [48]	12	12	1	6	0	21	11	38	35	16	8	3	8	0	9	5	8	1	−10	18	19	17	10	6	17	15	19	13	7	7	6	5	10
Botswana [49]	12	8	6	8	0	0	19	5	9	26	26	5	14	1	16	9	14	5	−3	17	15	13	8	23	17	15	16	13	3	4	4	16	11
Albania [50]	12	15	25	26	0	0	25	0	10	12	14	−3	14	4	18	10	13	10	1	17	0	6	6	18	15	0	14	1	16	11	5	3	14
Armenia [51]	12	11	32	19	0	0	0	0	0	16	29	−8	15	4	22	12	19	18	5	18	12	22	5	12	3	8	9	−3	42	7	−5	16	17
Zambia [52]	12	4	17	15	33	0	14	27	32	20	9	3	9	7	7	1	−1	−2	−5	16	12	16	10	17	15	15	6	13	3	3	7	7	10
Poland [53]	12	13	8	3	0	0	0	0	0	0	24	42	18	4	22	13	18	28	55	23	23	22	9	0	6	5	21	19	0	3	16	22	6
Romania [54]	12	12	20	15	0	0	0	0	0	12	22	−14	12	−2	19	16	18	27	41	27	16	15	2	13	15	8	18	13	8	10	4	21	4
Indonesia [55]	11	11	17	17	0	0	8	25	22	13	13	2	14	5	13	9	11	8	1	19	5	−5	10	22	12	10	13	11	3	−6	0	14	8
Nicaragua [56]	11	14	23	8	0	0	35	1	0	16	19	−8	12	5	16	11	9	2	0	19	1	8	4	21	15	13	14	12	14	0	−7	16	12
Slovenia [57]	11	16	3	23	0	0	0	0	0	0	22	10	13	6	13	15	14	20	44	19	27	29	15	0	32	0	23	12	14	14	11	37	5
Mali [58]	11	12	13	14	32	35	12	31	39	8	8	6	4	4	2	−1	−1	−1	−1	16	12	10	7	10	17	12	13	14	−6	3	1	−4	10
Latvia [59]	11	17	19	12	0	0	0	0	0	9	29	14	12	4	16	13	15	24	44	24	16	22	−26	0	11	12	16	18	13	3	−12	37	5
El Salvador [60]	11	14	23	8	0	0	13	5	0	13	24	−1	16	8	21	17	15	5	−1	14	4	13	4	18	22	11	11	3	9	−7	−7	14	15
Ecuador [61]	11	18	23	7	0	0	15	12	0	12	18	3	12	7	15	16	13	6	2	17	17	11	−3	15	16	12	11	1	18	5	−2	6	14
Afghanistan [62]	11	10	14	11	35	87	11	38	52	8	10	3	1	1	0	−2	−1	−3	−13	15	2	5	3	6	7	5	5	7	4	−4	1	−5	6
Colombia [63]	11	13	23	11	0	0	4	6	0	14	12	−19	14	7	19	17	19	10	4	21	16	23	16	11	20	7	14	4	7	15	9	7	13
Singapore [64]	11	16	4	7	0	0	0	0	0	2	3	8	16	9	19	21	19	35	57	20	25	27	5	24	26	0	0	17	3	18	−5	23	0
Chile [65]	11	11	13	21	0	0	−1	0	0	6	5	8	18	10	18	17	19	24	17	21	13	13	6	10	17	19	8	16	7	2	4	8	12
Bosnia and Herzegovina [66]	11	10	6	7	0	0	22	0	−8	6	23	−6	11	8	17	9	14	11	5	8	18	10	9	10	25	24	30	15	12	−8	7	17	27

Value: 25, 0, −25

Supplementary figure 10. Absolute change on the HAQ Index and 32 individual causes, by country or territory, from 2000–2016. Countries are ranked by the difference in their HAQ Index in 2016 from their HAQ Index in 2000. Positive values represent improvements for overall the overall index and individual causes from 2000–2016, while negative values represent worsening performance during that time. HAQ Index = Healthcare Access and Quality Index.

	Healthcare Access and Quality Index	Tuberculosis	Diarrhoeal diseases	Lower respiratory infections	Upper respiratory infections	Diphtheria	Whooping cough	Tetanus	Measles	Maternal disorders	Neonatal disorders	Non−melanoma skin cancer (squamous−cell carcinoma)	Breast cancer	Cervical cancer	Uterine cancer	Colon and rectum cancer	Testicular cancer	Hodgkin lymphoma	Leukaemia	Rheumatic heart disease	Ischaemic heart disease	Cerebrovascular disease	Hypertensive heart disease	Chronic respiratory diseases	Peptic ulcer disease	Appendicitis	Inguinal, femoral, and abdominal hernia	Gallbladder and biliary diseases	Epilepsy	Diabetes mellitus	Chronic kidney disease	Congenital heart anomalies	Adverse effects of medical treatment
Mauritania [67]	11	12	20	8	2	15	6	37	23	11	9	2	8	4	5	3	2	−1	−4	15	10	8	7	12	21	15	19	17	−4	3	4	−11	9
Iran [68]	11	8	20	11	0	0	6	0	11	18	20	−3	18	5	19	15	17	20	10	4	12	15	3	23	15	13	8	4	9	3	7	15	−11
Montenegro [69]	11	13	15	23	0	0	20	0	0	6	34	−1	14	6	17	12	16	20	29	14	10	15	3	0	16	0	0	11	9	5	6	31	9
Djibouti [70]	11	2	13	12	10	14	7	27	49	10	11	3	6	4	4	1	1	−1	−3	11	1	10	4	22	11	11	11	12	4	−3	−2	20	10
Ireland [71]	11	14	−3	21	0	0	0	0	0	0	16	10	17	11	15	17	16	28	41	17	32	21	6	15	26	0	14	9	8	7	12	20	7
Malawi [72]	11	7	18	15	37	0	13	30	−1	16	10	2	5	4	5	0	−1	−2	−2	17	13	14	8	21	16	17	15	14	7	5	6	17	14
Ukraine [73]	11	9	25	17	0	0	0	0	0	12	22	−9	9	6	15	11	13	17	21	26	11	18	2	7	12	1	20	15	14	15	7	24	11
Syria [74]	10	13	15	3	0	0	−6	0	2	27	16	1	19	9	20	11	16	4	0	38	12	18	10	4	11	21	0	3	9	11	13	16	15
Tunisia [75]	10	12	11	9	0	0	19	0	5	14	19	0	17	5	19	12	17	11	5	14	7	8	2	21	12	12	1	8	9	4	4	24	11
Serbia [76]	10	15	−2	6	0	0	16	0	0	1	17	26	10	4	13	11	13	20	9	23	21	24	9	14	12	15	16	10	6	4	5	23	14
Pakistan [77]	10	10	3	13	0	0	4	25	60	12	9	−5	8	3	7	3	5	−1	−5	9	3	7	4	20	15	4	11	8	6	−2	2	−2	7
United Arab Emirates [78]	10	8	8	6	0	0	8	19	21	8	17	−1	18	12	18	13	18	17	8	5	7	6	4	17	9	5	0	3	5	2	2	17	3
Uzbekistan [79]	10	12	26	12	0	0	0	0	0	14	10	2	15	7	17	10	16	10	−1	17	4	17	0	11	17	24	16	12	−4	−1	−3	9	10
Kuwait [80]	10	17	4	−4	0	0	0	0	0	5	9	8	19	1	28	21	28	35	43	11	18	3	12	10	15	−5	0	1	15	26	18	12	0
Macedonia [81]	10	18	26	7	0	0	9	0	0	6	9	−6	12	3	16	11	14	17	13	21	12	9	4	11	18	0	20	7	5	1	7	13	11
Slovakia [82]	10	15	4	9	0	0	8	0	0	0	15	6	14	6	16	14	13	22	53	16	21	15	1	9	12	3	16	9	7	9	12	15	1
Burundi [83]	10	7	6	8	21	0	13	34	40	14	8	1	−1	−3	−3	−5	−4	−5	−16	19	11	16	12	10	17	13	16	11	5	6	9	2	9
Ghana [84]	10	11	21	9	3	0	19	27	43	11	10	−2	3	−3	2	−3	0	−3	−8	20	8	7	2	11	7	15	18	10	−11	−5	−4	4	0
Comoros [85]	10	5	21	4	6	0	−1	28	21	19	8	2	7	4	5	2	1	−1	−3	17	4	10	9	13	14	13	15	10	6	3	4	10	10
Bermuda [86]	10	1	5	12	0	0	0	0	0	0	3	2	13	12	14	12	12	23	23	24	34	22	14	6	21	10	18	22	13	12	16	3	8
Portugal [87]	10	15	2	10	0	0	0	0	4	0	22	28	11	4	12	11	12	18	11	19	26	29	10	19	22	0	11	−1	−5	14	9	30	15
Guatemala [88]	9	15	18	9	0	0	11	12	12	13	13	−9	13	5	17	12	9	2	0	−1	6	10	2	30	14	11	11	−1	2	−7	−15	−9	14
Federated States of Micronesia [89]	9	9	46	1	0	0	6	0	49	9	12	−7	11	6	12	8	8	4	−2	5	−8	0	3	8	6	3	9	2	−2	−6	−2	1	4
Moldova [90]	9	11	24	7	0	0	0	0	0	9	26	−11	9	4	15	8	9	8	5	27	4	9	−8	7	10	13	18	13	20	19	−6	23	13
Niger [91]	9	6	13	16	30	72	17	28	46	2	11	−1	1	2	0	−3	−2	−4	−3	6	1	−2	−1	18	8	5	3	4	−9	−4	−4	−9	8
Sao Tome and Principe [92]	9	10	23	4	4	0	17	31	15	14	12	1	4	0	1	0	0	−2	−3	10	6	3	3	20	20	11	14	13	−8	−2	3	14	7
Tanzania [93]	9	3	15	13	24	0	6	20	38	7	11	2	6	3	6	0	−1	−3	−8	15	1	8	6	11	13	13	11	11	3	1	3	11	8
Bulgaria [94]	9	11	10	17	0	0	0	0	0	13	18	1	13	6	14	16	17	28	23	19	12	13	−1	9	10	−13	31	−1	2	8	0	16	0
Tajikistan [95]	9	12	20	13	4	0	13	0	2	12	10	1	7	4	8	4	4	1	1	16	7	8	6	21	17	14	19	13	4	4	8	3	9
Costa Rica [96]	9	15	14	8	0	0	0	0	0	13	10	9	14	10	19	17	18	13	3	13	10	12	9	14	12	1	3	7	1	10	2	10	11
Togo [97]	9	7	14	10	15	19	18	35	41	13	8	−2	2	−2	0	−1	0	−2	−4	7	3	0	−1	16	11	4	9	6	−9	−2	−4	2	6
Mozambique [98]	9	4	18	11	49	1	9	23	16	5	12	2	2	1	2	−1	−1	−2	−4	13	6	9	4	19	13	12	13	11	7	0	4	10	10
Liberia [99]	9	10	15	14	44	47	14	34	20	9	16	−1	1	−3	0	−4	−2	−5	−7	8	1	−1	−1	22	12	5	7	6	−9	0	−3	12	8

Value: 25, 0, −25

Supplementary figure 10. Absolute change on the HAQ Index and 32 individual causes, by country or territory, from 2000–2016. Countries are ranked by the difference in their HAQ Index in 2016 from their HAQ Index in 2000. Positive values represent improvements for overall the overall index and individual causes from 2000–2016, while negative values represent worsening performance during that time. HAQ Index = Healthcare Access and Quality Index.

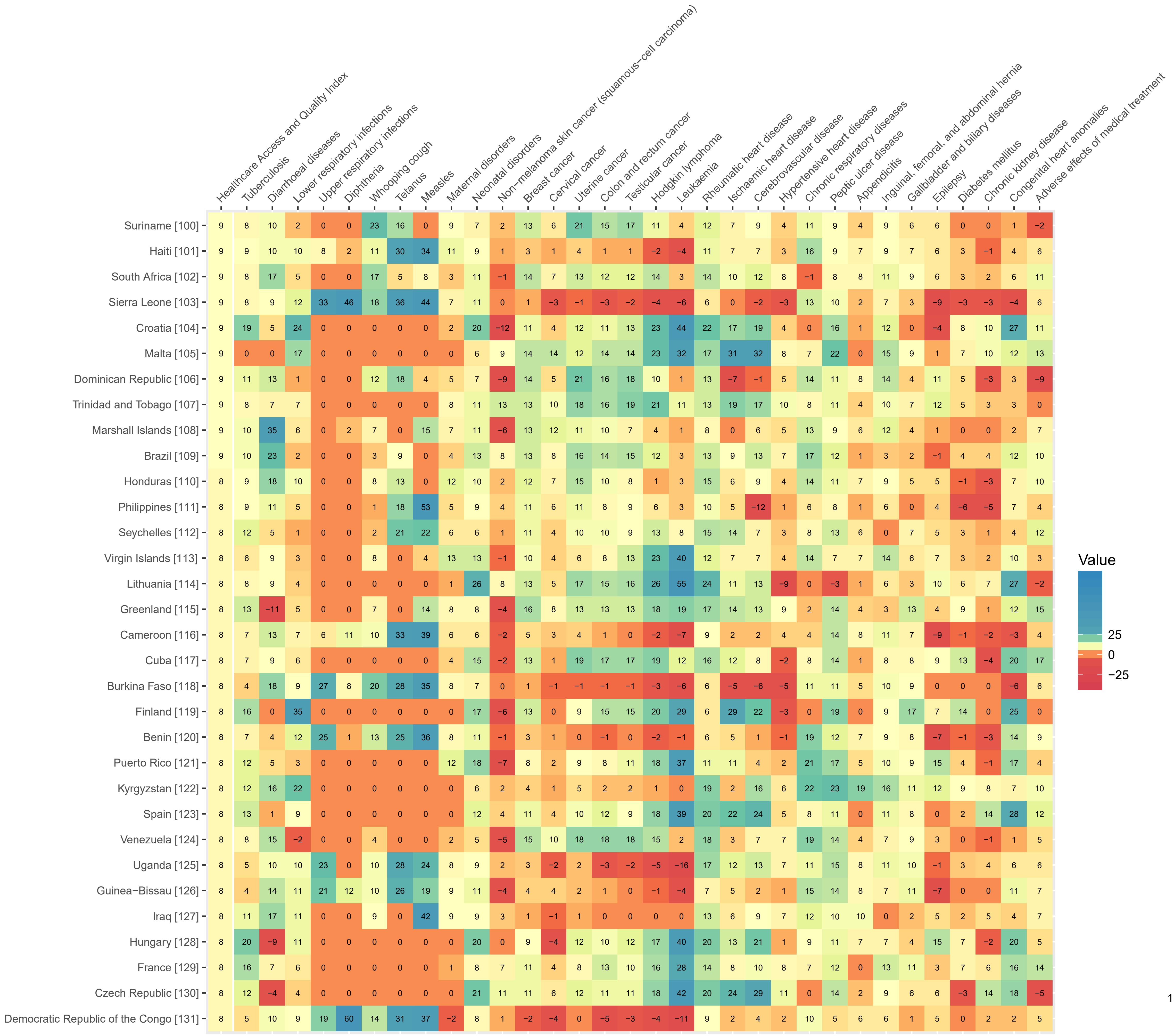

Supplementary figure 10. Absolute change on the HAQ Index and 32 individual causes, by country or territory, from 2000–2016. Countries are ranked by the difference in their HAQ Index in 2016 from their HAQ Index in 2000. Positive values represent improvements for overall the overall index and individual causes from 2000–2016, while negative values represent worsening performance during that time. HAQ Index = Healthcare Access and Quality Index.

	Healthcare Access and Quality Index	Tuberculosis	Diarrhoeal diseases	Lower respiratory infections	Upper respiratory infections	Diphtheria	Whooping cough	Tetanus	Measles	Maternal disorders	Neonatal disorders	Non−melanoma skin cancer (squamous−cell carcinoma)	Breast cancer	Cervical cancer	Uterine cancer	Colon and rectum cancer	Testicular cancer	Hodgkin lymphoma	Leukaemia	Rheumatic heart disease	Ischaemic heart disease	Cerebrovascular disease	Hypertensive heart disease	Chronic respiratory diseases	Peptic ulcer disease	Appendicitis	Inguinal, femoral, and abdominal hernia	Gallbladder and biliary diseases	Epilepsy	Diabetes mellitus	Chronic kidney disease	Congenital heart anomalies	Adverse effects of medical treatment
Panama [132]	7	7	3	−8	0	0	−1	0	0	2	9	9	16	8	21	20	20	17	2	18	1	10	3	5	11	−2	15	15	4	−5	−1	8	12
Netherlands [133]	7	5	−3	10	0	0	0	0	0	4	12	16	14	11	10	14	9	14	25	3	36	23	2	11	18	0	4	10	3	16	8	22	8
Mexico [134]	7	12	18	6	0	0	1	0	0	10	13	−11	13	7	18	16	17	14	6	19	−7	5	3	16	10	−1	0	−3	5	1	−8	6	2
Japan [135]	7	16	2	11	0	0	0	0	9	0	4	−7	12	6	14	7	8	17	33	15	20	21	8	17	14	0	0	9	0	1	11	22	−1
Chad [136]	7	9	7	10	28	53	6	26	25	4	5	2	2	1	1	0	−1	−2	−7	8	8	5	2	6	12	6	8	5	−7	0	−2	−8	6
Kenya [137]	7	0	12	8	0	0	13	14	14	13	8	3	9	1	8	1	2	−1	−8	12	−1	8	4	12	9	9	10	8	4	1	3	1	6
Denmark [138]	7	7	−7	−2	0	0	0	0	0	0	6	5	17	14	10	20	11	15	10	6	32	18	11	12	18	12	12	15	−1	10	8	20	0
Belize [139]	7	9	16	6	0	0	1	10	−7	25	16	2	7	5	13	5	7	2	1	13	18	9	3	16	8	2	8	0	9	4	0	9	0
Antigua and Barbuda [140]	7	9	8	4	0	0	0	3	0	2	16	−2	11	9	13	12	14	16	12	12	14	11	5	8	11	4	10	6	7	2	5	4	0
Eritrea [141]	7	−4	8	3	8	0	24	14	29	7	8	0	6	4	1	2	1	−1	−3	10	−3	3	4	16	5	6	3	5	2	−2	2	9	6
Paraguay [142]	7	4	20	4	0	0	2	22	0	10	12	5	16	8	21	15	17	6	1	0	−6	2	−3	11	7	−5	1	−4	3	−7	−13	0	2
Mauritius [143]	7	6	3	9	0	0	0	0	0	1	10	−1	16	9	16	16	15	23	17	14	33	30	3	10	17	7	1	−4	2	−16	−8	9	−4
Israel [144]	7	13	3	1	0	0	0	0	0	3	24	−7	10	6	8	9	8	12	8	11	33	23	6	17	10	0	7	7	1	9	8	22	14
Tonga [145]	7	7	5	−1	0	0	24	0	18	8	7	−8	12	8	9	9	7	4	−1	8	3	5	4	5	8	6	9	4	3	−2	1	4	5
Belgium [146]	7	11	−2	7	0	0	0	0	0	0	12	18	11	5	8	12	8	13	21	1	26	19	6	12	15	0	9	10	6	13	6	18	6
Cote d'Ivoire [147]	7	8	3	9	6	0	8	27	43	6	6	1	2	−1	1	−1	0	−1	−7	6	8	3	1	4	13	4	10	4	−10	1	−2	1	5
Senegal [148]	7	8	14	8	11	0	13	29	29	7	13	−1	−2	−6	−1	−4	−2	−4	−10	7	2	0	0	13	12	5	9	5	−10	−3	−2	3	4
Guyana [149]	7	8	19	3	0	0	0	8	0	3	10	−3	10	4	16	12	12	8	5	11	2	7	2	17	4	1	5	2	8	8	−3	6	1
United Kingdom [150]	7	9	3	12	0	0	0	0	0	0	11	−2	11	9	9	13	9	17	22	21	31	19	3	7	16	0	7	−4	5	7	4	13	3
Saint Lucia [151]	7	9	10	1	0	0	0	0	0	15	0	−2	10	4	15	11	15	10	3	12	7	7	3	13	11	6	10	4	5	7	0	−1	−1
Austria [152]	6	9	−1	2	0	0	0	0	0	0	15	0	8	2	9	11	11	15	31	22	28	20	4	10	20	0	2	3	12	5	−7	20	3
Brunei [153]	6	7	−4	−3	0	0	8	2	0	9	−3	−4	14	3	16	16	16	29	48	9	5	10	−1	6	9	0	0	−1	1	9	0	−1	−2
Argentina [154]	6	9	12	−6	0	0	3	0	0	4	16	1	13	6	14	12	15	14	6	10	10	13	4	13	4	−2	3	5	3	5	4	11	3
Guinea [155]	6	4	13	10	18	33	4	19	30	7	9	0	2	0	0	−1	−2	−3	−6	4	−1	−1	−1	13	10	4	6	5	−10	−2	−3	4	6
Uruguay [156]	6	7	14	5	0	0	−1	0	0	6	17	7	13	8	14	11	15	14	6	8	14	14	3	12	9	1	2	−4	−2	0	−1	12	−4
Swaziland [157]	6	3	6	1	0	0	15	11	−1	12	7	4	12	7	11	7	5	1	−5	10	−2	2	5	12	9	6	5	7	0	−5	−1	5	5
Italy [158]	6	7	−1	1	0	0	0	0	0	0	16	9	7	6	3	8	9	16	28	14	20	17	7	10	15	0	4	4	0	9	9	21	11
Norway [159]	6	8	−1	8	0	0	0	0	0	0	16	−3	16	8	11	16	5	12	15	11	31	20	0	4	14	0	0	10	5	10	2	24	0
Germany [160]	6	7	−4	4	0	0	0	0	0	0	10	23	14	10	8	14	8	12	7	16	27	19	7	10	14	0	2	3	0	8	4	18	−1
The Gambia [161]	6	7	8	8	4	0	6	21	21	3	9	8	3	1	3	−1	−1	−3	−6	8	5	1	2	7	13	7	10	7	−9	−1	−1	3	6
Madagascar [162]	6	7	9	7	6	0	9	16	23	7	4	2	3	2	2	0	0	−2	−5	8	7	5	3	9	5	4	6	3	2	0	1	7	5
North Korea [163]	6	5	17	5	0	0	27	18	35	3	4	1	−1	−2	−1	−3	−1	−2	−3	6	−5	−4	1	8	2	1	0	1	−1	−2	−2	0	3

Value: 25, 0, −25

Supplementary figure 10. Absolute change on the HAQ Index and 32 individual causes, by country or territory, from 2000–2016. Countries are ranked by the difference in their HAQ Index in 2016 from their HAQ Index in 2000. Positive values represent improvements for overall the overall index and individual causes from 2000–2016, while negative values represent worsening performance during that time. HAQ Index = Healthcare Access and Quality Index.

	Healthcare Access and Quality Index	Tuberculosis	Diarrhoeal diseases	Lower respiratory infections	Upper respiratory infections	Diphtheria	Whooping cough	Tetanus	Measles	Maternal disorders	Neonatal disorders	Non−melanoma skin cancer (squamous−cell carcinoma)	Breast cancer	Cervical cancer	Uterine cancer	Colon and rectum cancer	Testicular cancer	Hodgkin lymphoma	Leukaemia	Rheumatic heart disease	Ischaemic heart disease	Cerebrovascular disease	Hypertensive heart disease	Chronic respiratory diseases	Peptic ulcer disease	Appendicitis	Inguinal, femoral, and abdominal hernia	Gallbladder and biliary diseases	Epilepsy	Diabetes mellitus	Chronic kidney disease	Congenital heart anomalies	Adverse effects of medical treatment
Luxembourg [164]	6	3	−1	14	0	0	0	0	0	0	20	−3	9	7	6	8	1	2	5	14	29	28	6	8	17	0	10	7	7	7	4	9	10
Jamaica [165]	6	8	6	3	0	0	22	0	0	9	7	1	9	4	13	9	10	8	1	11	3	5	−11	4	9	8	−2	2	4	3	3	1	−5
Australia [166]	5	0	−1	7	0	0	0	0	0	0	13	0	10	2	10	10	9	12	31	10	29	14	2	11	12	0	1	6	8	4	1	17	8
Somalia [167]	5	−1	4	6	15	38	0	12	31	5	6	1	1	0	−1	−1	−1	−2	−7	9	2	6	5	12	5	5	4	4	2	−2	1	5	6
New Zealand [168]	5	5	−9	5	0	0	0	0	0	−3	4	−5	9	4	9	10	11	20	33	12	29	21	9	12	14	0	1	6	9	7	6	15	0
Grenada [169]	5	4	5	−3	0	0	0	21	0	6	0	−3	15	7	23	16	18	10	1	9	3	3	1	5	4	−3	−5	8	−4	0	−4	−2	−4
Greece [170]	5	3	0	8	0	0	0	0	0	0	16	−8	13	5	10	12	11	19	21	12	14	21	−7	0	2	0	0	0	0	0	12	19	10
Fiji [171]	5	5	−2	1	0	0	12	0	−8	9	0	−4	15	8	14	14	13	19	9	12	6	8	5	1	7	5	−7	−13	3	0	1	−21	4
Canada [172]	5	0	−5	5	0	0	0	0	0	0	1	22	9	4	7	9	8	13	21	10	17	13	0	4	8	0	2	4	5	6	4	12	7
Saint Vincent and the Grenadines [173]	4	6	9	−2	0	0	0	0	0	2	10	−2	10	5	16	11	12	6	3	8	0	−2	0	13	4	−3	0	11	2	2	0	3	−5
Iceland [174]	4	9	−2	8	0	0	0	0	0	0	11	−5	1	6	10	5	7	9	16	7	25	17	2	9	12	0	0	4	−3	0	0	20	7
Samoa [175]	4	9	6	2	0	1	7	1	−13	7	13	−4	6	4	5	4	3	1	1	7	0	5	5	13	7	5	8	1	2	0	−2	6	5
Switzerland [176]	4	2	−4	11	0	0	0	0	0	0	8	6	10	3	7	−1	9	9	16	7	23	8	6	2	11	0	0	1	9	7	2	21	3
Georgia [177]	4	6	26	40	0	0	3	0	0	−13	20	−22	12	2	16	9	15	13	11	−3	20	15	−16	14	−3	−10	−15	−3	5	−6	−26	0	−16
Vanuatu [178]	4	8	9	−1	1	0	1	3	9	5	5	−3	7	5	5	3	3	0	−2	4	−1	2	2	6	7	4	8	2	−3	−5	−3	−3	4
Barbados [179]	4	5	9	1	0	0	0	4	0	−3	7	−5	5	3	9	5	7	12	11	9	14	4	1	10	5	3	2	−3	6	1	1	2	−4
American Samoa [180]	4	5	1	4	0	0	7	0	−36	4	18	−13	16	8	14	16	15	20	10	5	4	9	4	13	8	−4	12	−5	−16	−5	−4	0	−1
Kiribati [181]	3	6	13	5	7	0	9	0	−5	7	7	−4	4	2	3	1	1	−1	−2	4	4	6	1	9	7	2	8	2	1	0	1	3	0
Papua New Guinea [182]	3	8	7	0	5	0	−3	13	12	6	4	−4	5	4	2	1	0	−2	−11	5	−1	4	4	1	8	3	7	2	−3	−5	0	−5	3
South Sudan [183]	3	−4	13	6	4	47	1	14	7	4	1	1	0	−1	−2	−2	−2	−3	−9	7	−1	5	3	11	1	1	−1	2	0	−3	−1	2	4
Palestine [184]	3	6	18	7	0	0	13	1	19	−11	4	−2	−4	−3	−5	−1	0	0	4	26	2	3	−36	12	1	3	0	−2	1	−6	−9	18	19
Sweden [185]	3	3	−1	3	0	0	0	0	0	0	10	−15	7	8	2	8	3	9	14	4	21	16	−4	3	9	0	1	3	8	7	0	17	1
The Bahamas [186]	3	6	8	0	0	0	0	0	0	1	8	−9	5	1	7	5	5	11	4	8	7	1	2	5	6	2	7	0	1	−1	2	1	−4
Dominica [187]	3	5	5	−4	0	0	−9	6	0	7	−8	−14	12	6	16	13	14	13	4	7	6	−4	0	3	4	0	3	0	0	−2	−1	−13	−5
Lesotho [188]	3	−4	5	0	5	0	9	10	−6	7	6	3	12	7	11	8	7	2	−5	3	−5	−1	−2	2	3	−2	−1	0	−2	−14	−5	1	−2
Central African Republic [189]	2	0	−2	6	1	24	−3	12	12	3	4	2	1	0	−1	0	−1	−1	−7	5	3	3	2	3	3	2	3	0	0	−1	0	−2	4
United States [190]	2	2	−1	−2	0	0	0	0	0	−10	4	−8	4	−2	5	7	6	9	22	7	14	7	−3	1	8	0	1	5	0	1	−7	9	4
Andorra [191]	2	1	−2	0	0	0	11	0	0	0	−1	−1	6	2	4	7	5	6	2	4	3	1	0	−2	8	0	1	−1	−5	0	0	2	0
Northern Mariana Islands [192]	2	1	0	−1	0	0	0	0	7	5	9	−17	9	3	5	8	3	12	11	−1	−4	4	0	12	−1	−8	0	−7	−4	−8	−5	2	−2
Solomon Islands [193]	1	7	6	2	1	0	0	3	−15	5	2	−4	3	3	1	1	1	−1	−9	4	−2	3	2	1	7	3	8	1	−1	−8	−2	−12	2
Zimbabwe [194]	0	1	−5	1	−7	0	7	8	20	2	−1	−1	−8	−14	−4	−9	−3	−5	−10	8	13	1	4	−5	15	−9	11	−15	−5	−7	−6	−4	2
Guam [195]	−3	−1	−3	−3	0	0	−2	0	−39	−9	−3	−8	8	3	5	8	4	12	20	−2	−8	1	2	−2	−2	−3	3	−5	−11	−11	−9	−10	−10

Value: 25, 0, −25

Supplementary figure 11. Comparing the HAQ Index in 2016 to the log of government health spending per capita (A). Government health spending per capita is based on the cumulative per capita spending from 2010 to 2015 in 2017 PPP. Countries and territories are colour-coded by their SDI quintile, and are abbreviated according to the ISO3 code. HAQ Index = Healthcare Access and Quality Index. PPP = purchasing power parity. SDI = Socio-demographic Index.

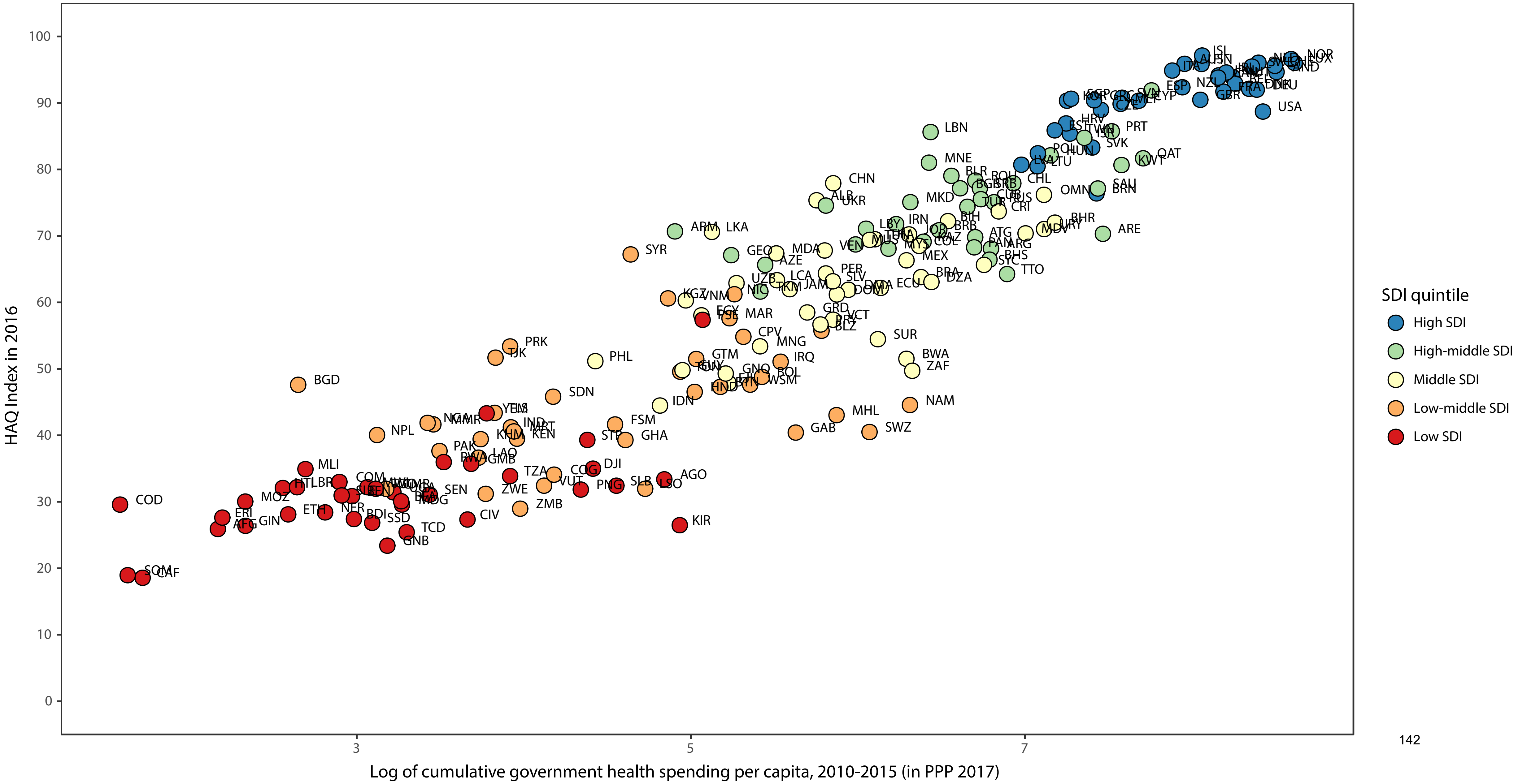

Supplementary figure 11. Comparing the HAQ Index in 2016 to the log of out-of-pocket health spending per capita (B). Out-of-pocket spending per capita is based on the cumulative per capita spending from 2010 to 2015 in 2017 PPP. Countries and territories are colour-coded by their SDI quintile, and are abbreviated according to the ISO3 code. HAQ Index = Healthcare Access and Quality Index. PPP = purchasing power parity. SDI = Socio-demographic Index.

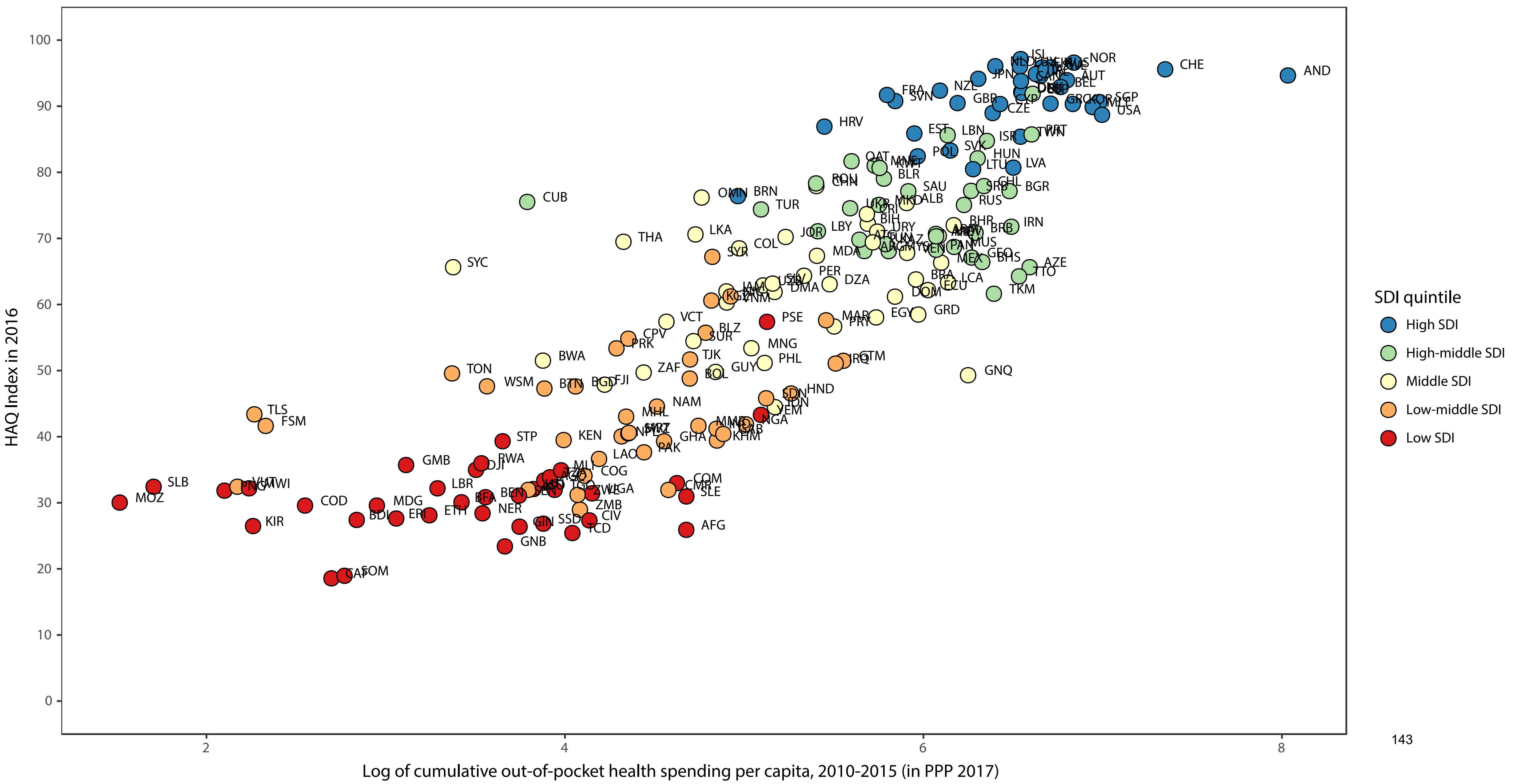

Supplementary figure 11. Comparing the HAQ Index in 2016 to the log of DAH per capita (C). DAH per capita is based on the cumulative per capita spending from 2010 to 2016 in 2017 PPP. Countries and territories are colour-coded by their SDI quintile, and are abbreviated according to the ISO3 code. HAQ Index = Healthcare Access and Quality Index. DAH = development assistance for health. PPP = purchasing power parity. SDI = Socio-demographic Index.

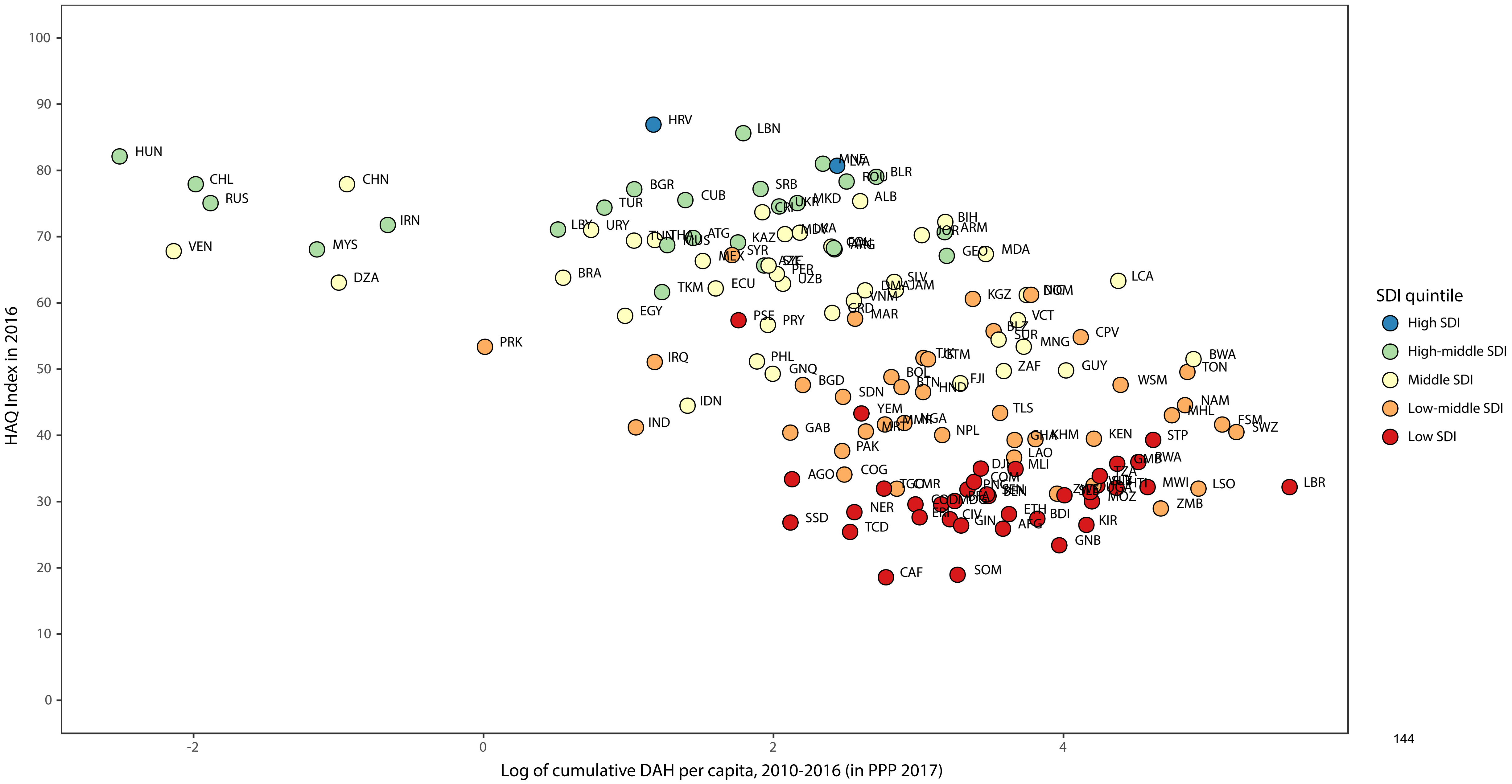

Supplementary figure 11. Comparing the HAQ Index in 2016 to government health spending as a percent of total health spending (D). Government health spending per capita is based on the cumulative per capita spending from 2010 to 2015 in 2017 PPP. Percentages are based on the fraction of government health spending from total health spending per capita for that period of time. Countries and territories are colour-coded by their SDI quintile, and are abbreviated according to the ISO3 code. HAQ Index = Healthcare Access and Quality Index. PPP = purchasing power parity. SDI = Socio-demographic Index.

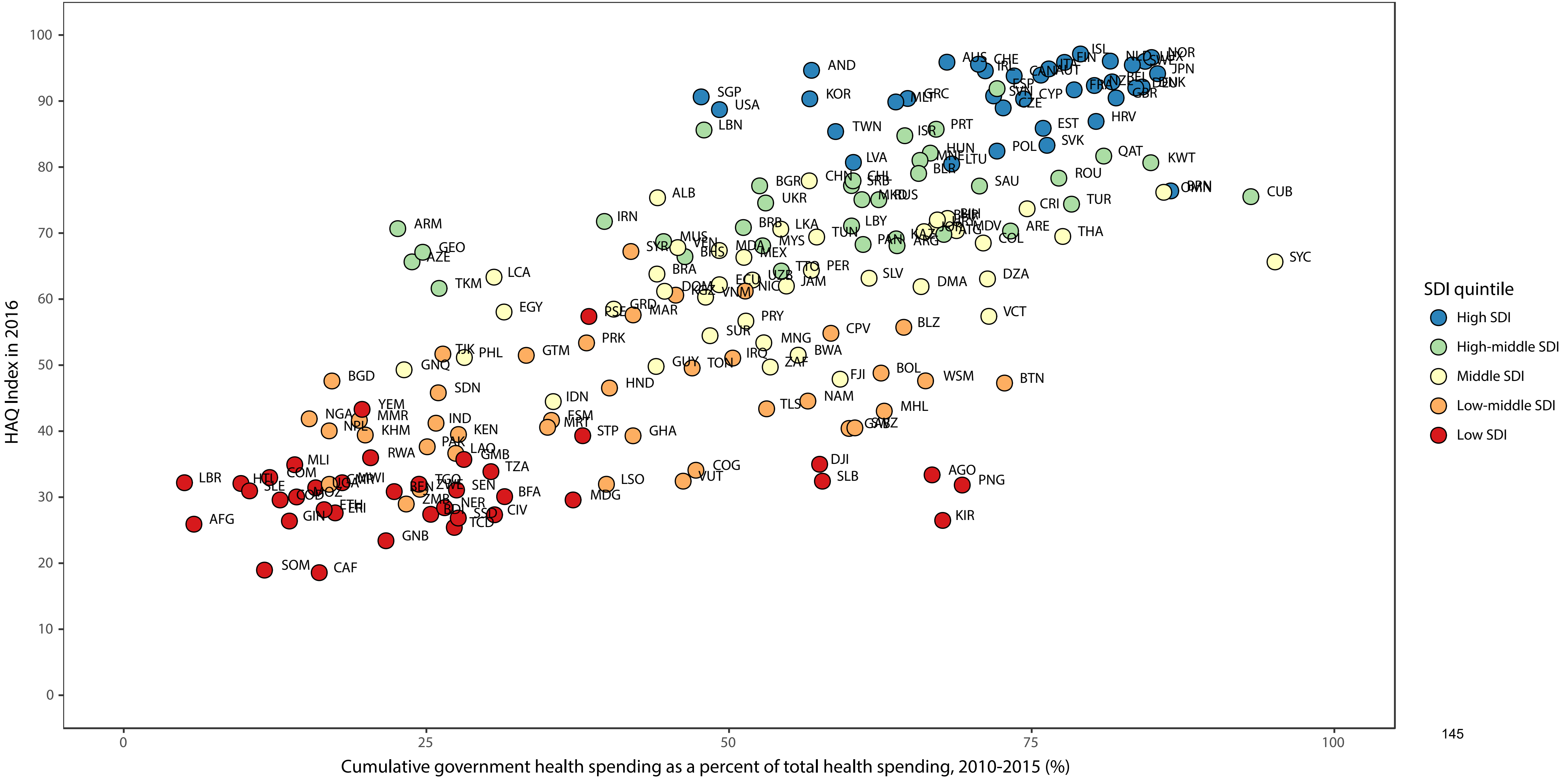

Supplementary figure 11. Comparing the HAQ Index in 2016 to out-of-pocket health spending as a percent of total health spending (E). Out-of-pocket spending per capita is based on the cumulative per capita spending from 2010 to 2015 in 2017 PPP. Percentages are based on the fraction of out-of-pocket health spending from total health spending per capita for that period of time. Countries and territories are colour-coded by their SDI quintile, and are abbreviated according to the ISO3 code. HAQ Index = Healthcare Access and Quality Index. PPP = purchasing power parity. SDI = Socio-demographic Index.

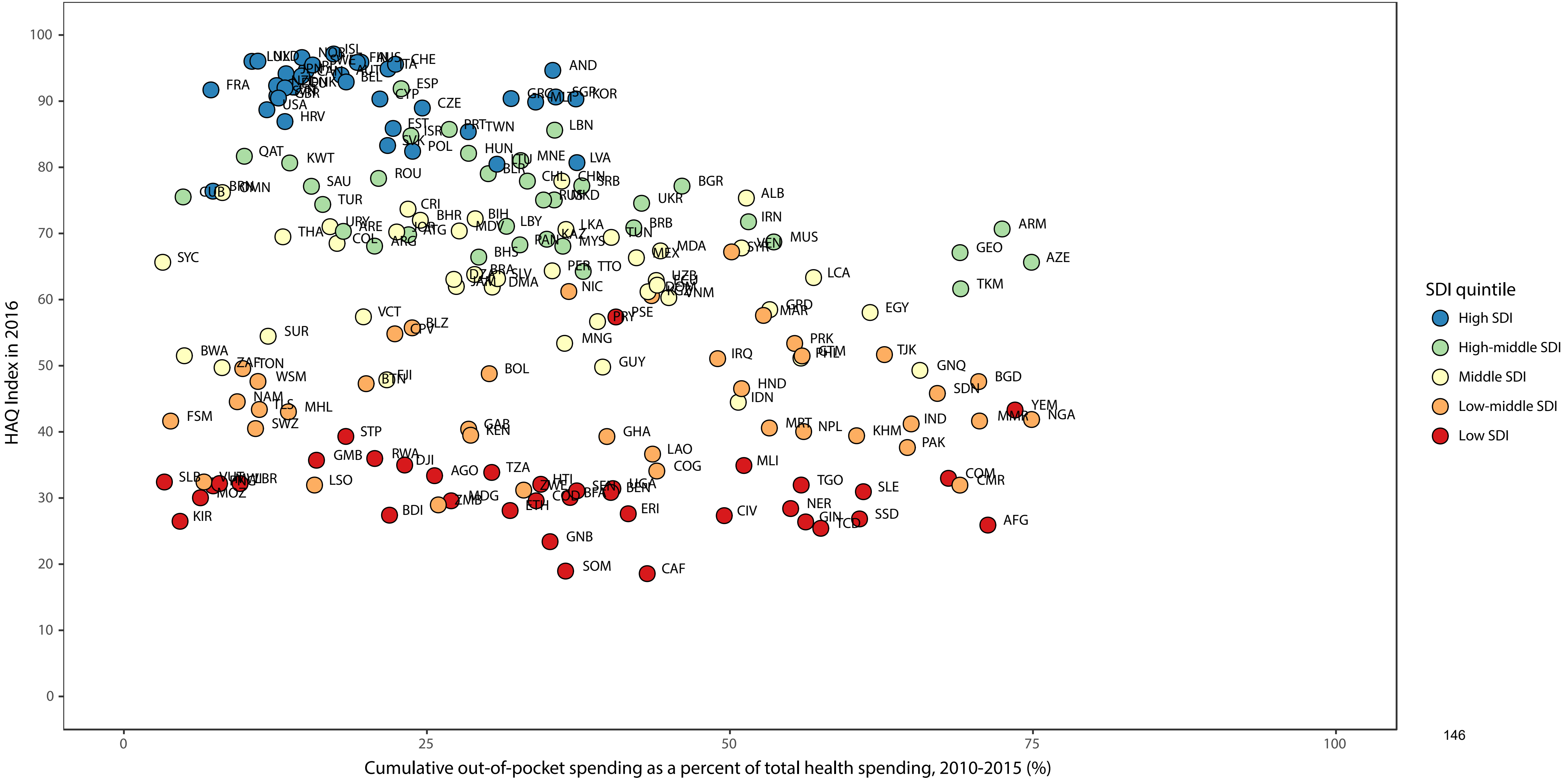

Supplementary figure 11. Comparing the HAQ Index in 2016 to DAH as a percent of total health spending (F). DAH per capita is based on the cumulative per capita expenditure from 2010 to 2016 in 2017 PPP. Percentages were based on the fraction of DAH per capita from total health spending per capita for that period of time. Countries and territories are colour-coded by their SDI quintile, and are abbreviated according to the ISO3 code. HAQ Index = Healthcare Access and Quality Index. DAH = development assistance for health.PPP = purchasing power parity. SDI = Socio-demographic Index.

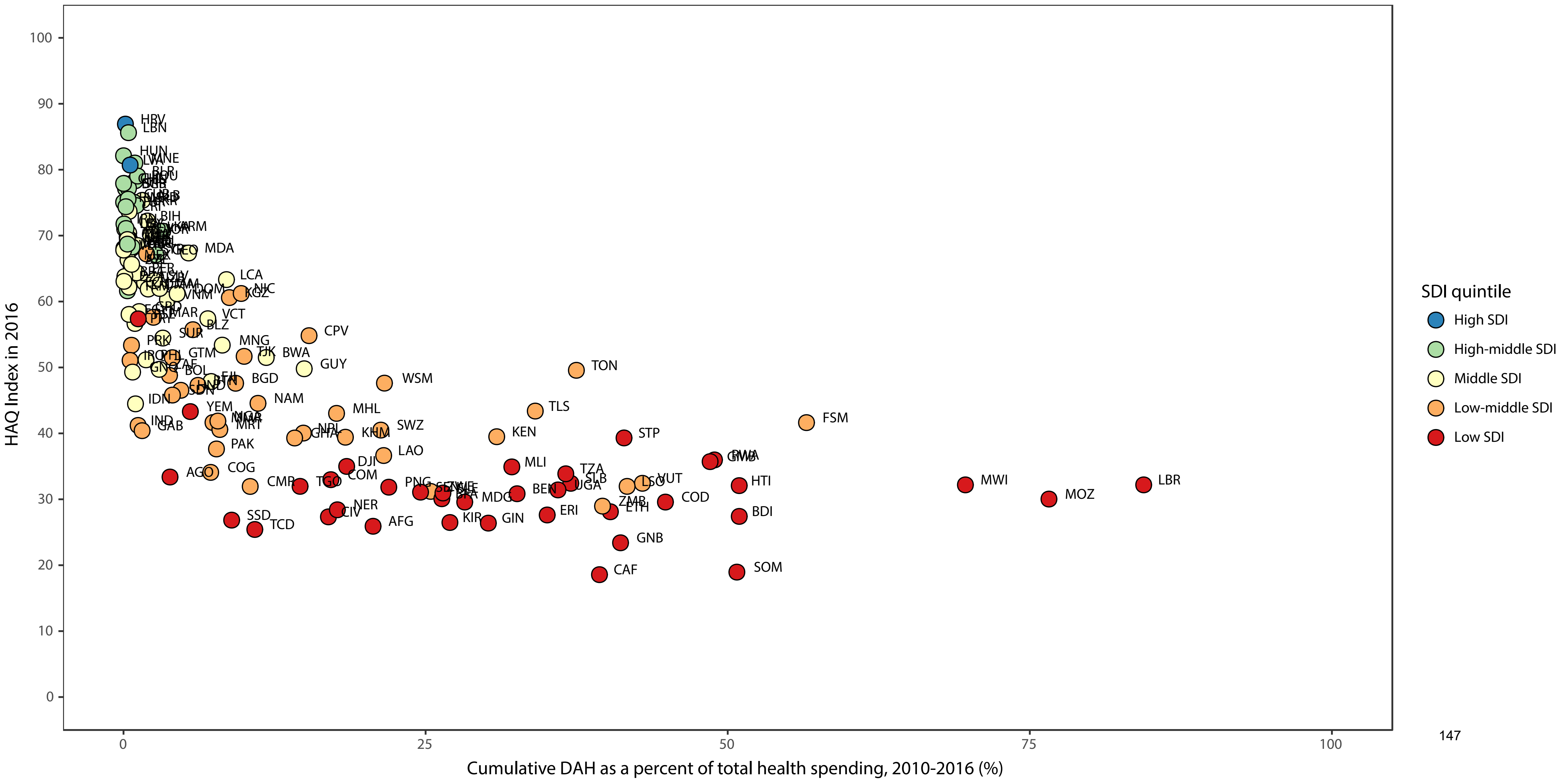

Supplementary figure 11. Comparing the HAQ Index in 2016 to and SDI (G). Countries and territories are colour-coded by their SDI quintile, and are abbreviated according to the ISO3 code. HAQ Index = Healthcare Access and Quality Index. SDI = Socio-demographic Index.

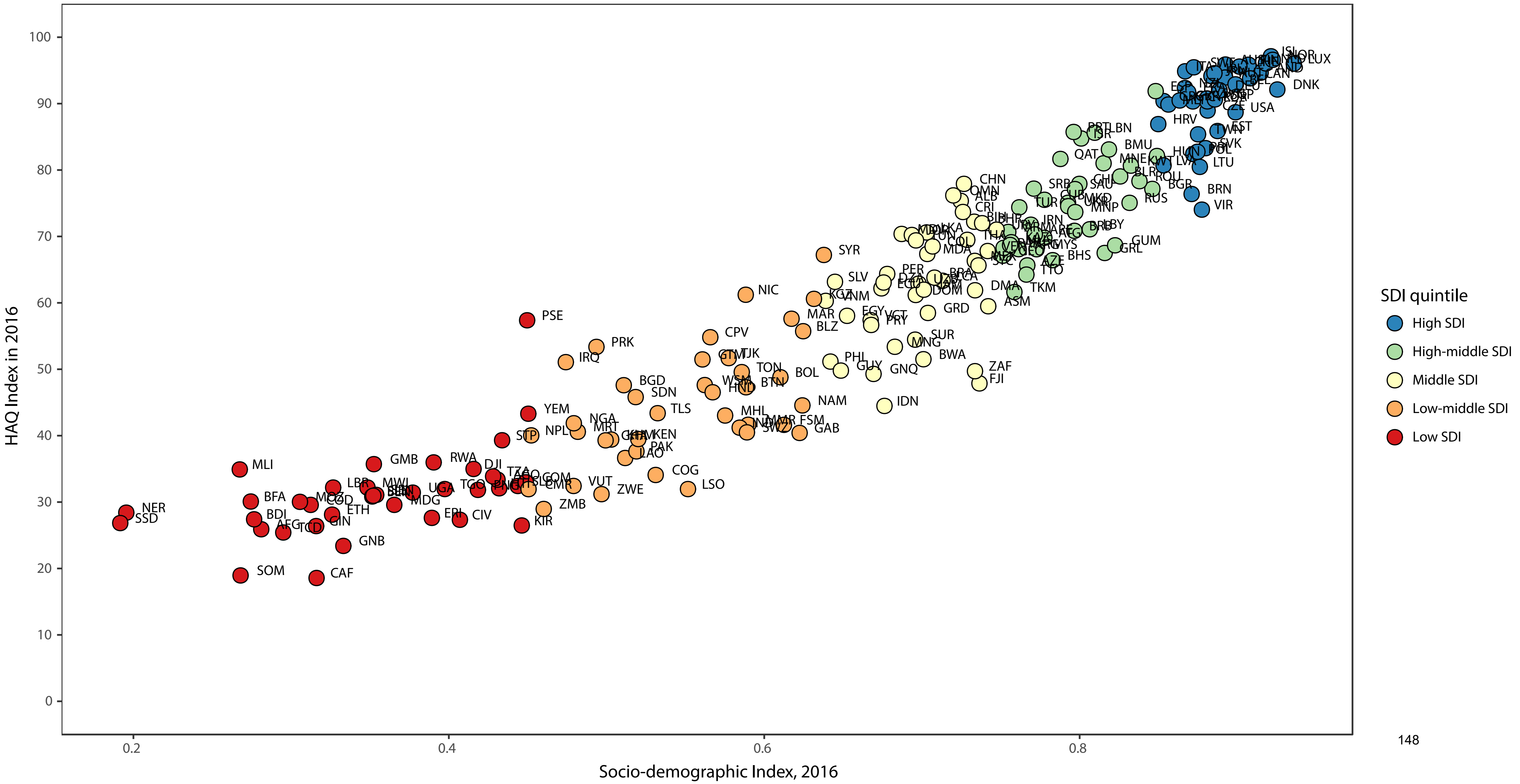

Supplementary figure 12. Comparing the HAQ Index in 2016 to physicians, nurses, and midwives per 1,000 (A). Physicians, nurses, and midwives per 1,000 is based on the most recent location-year of data between 2010 and 2015 from the WHO Global Health Observatory database. Countries and territories are colour-coded by their SDI quintile, and are abbreviated according to the ISO3 code. HAQ Index = Healthcare Access and Quality Index. SDI = Socio-demographic Index.

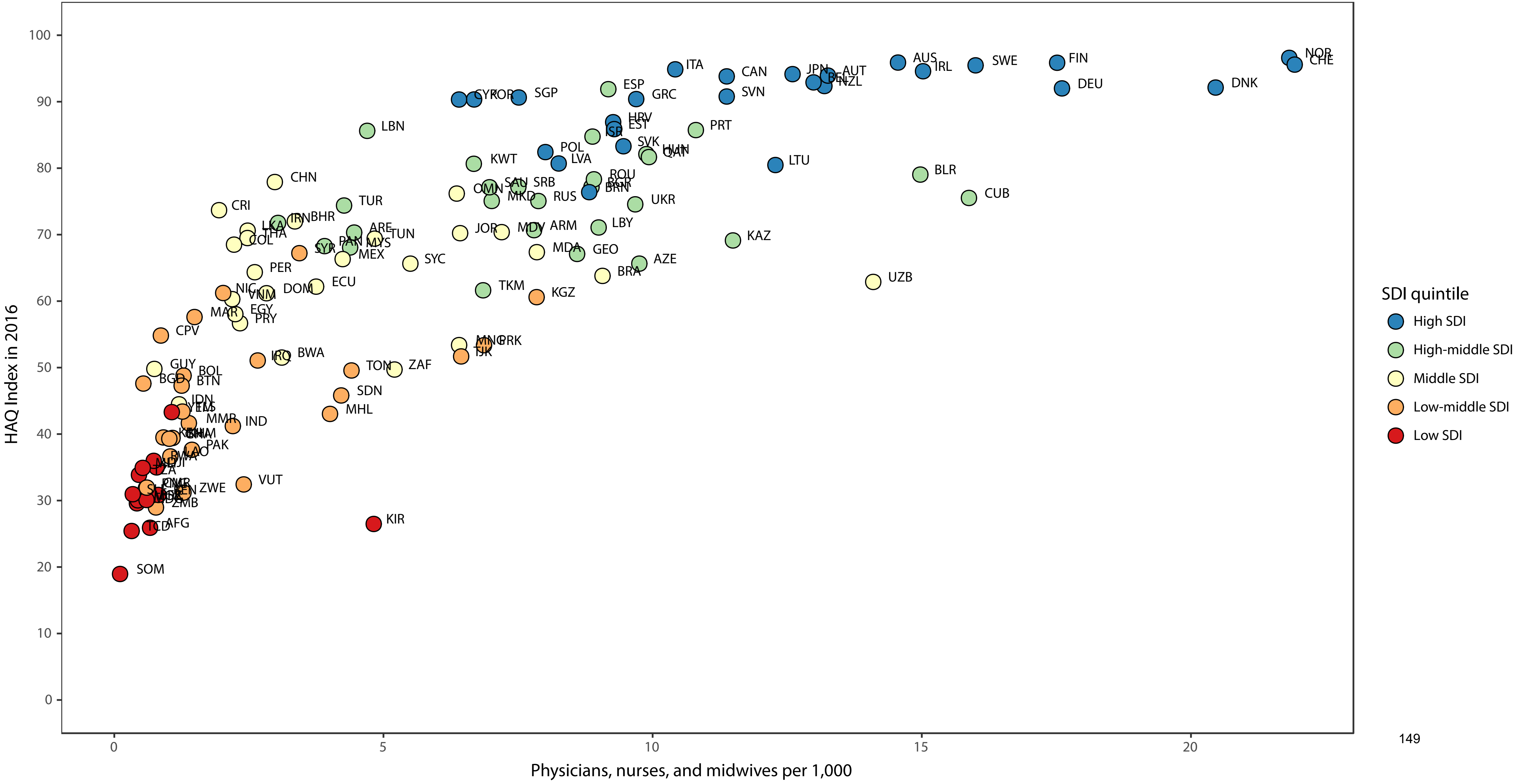

Supplementary figure 12. Comparing the HAQ Index in 2016 to hospital beds per 1,000 (B). Hospital beds per 1,000 were estimated for 2016. Countries and territories are colour-coded by their SDI quintile, and are abbreviated according to the ISO3 code. HAQ Index = Healthcare Access and Quality Index. SDI = Socio-demographic Index.

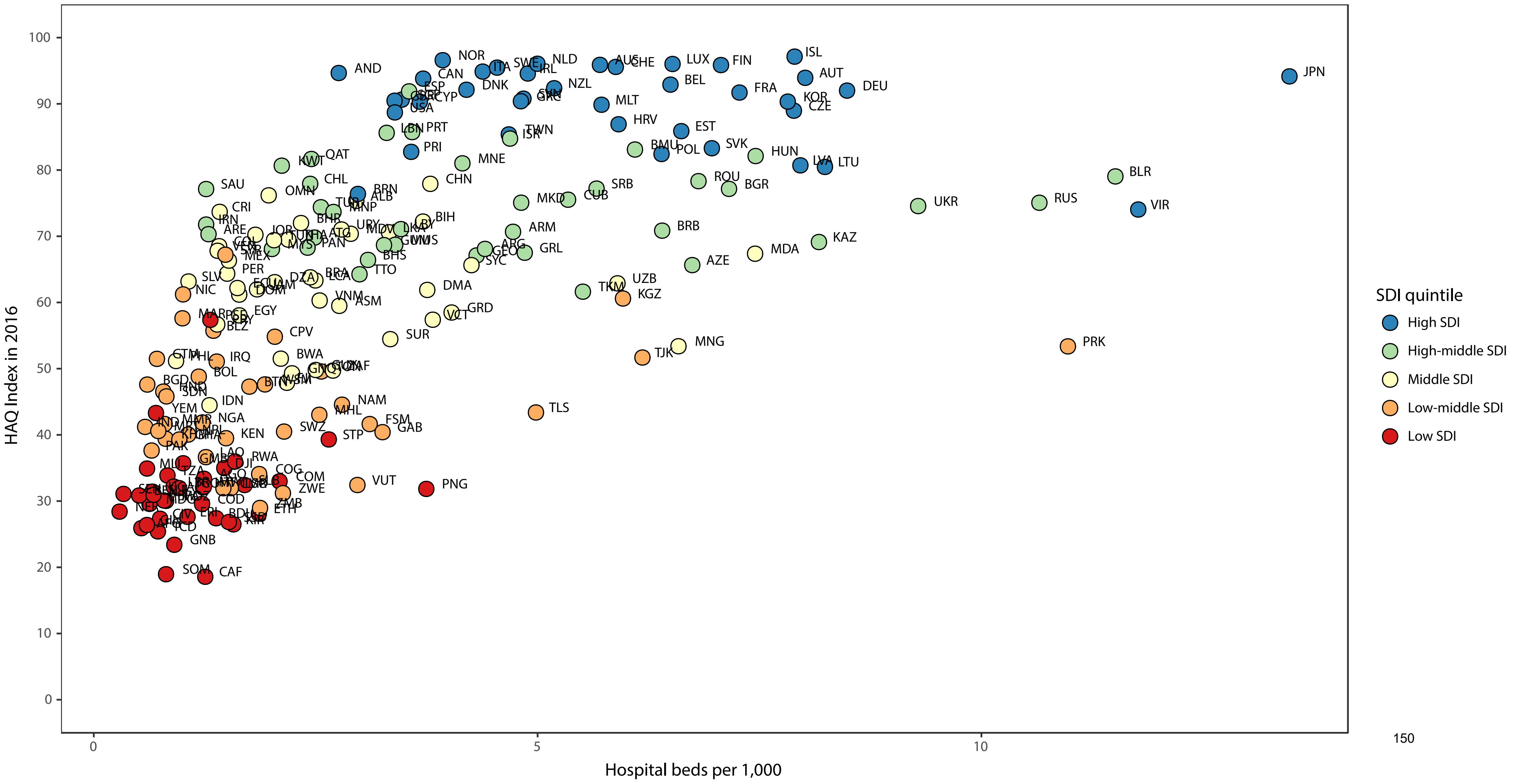

Supplementary figure 12. Comparing the HAQ Index in 2016 to outpatient utilisation rates (C). Outpatient utilisation rates are the annual number of outpatient visits per capita in 2016. Countries and territories are colour-coded by their SDI quintile, and are abbreviated according to the ISO3 code. HAQ Index = Healthcare Access and Quality Index. SDI = Socio-demographic Index.

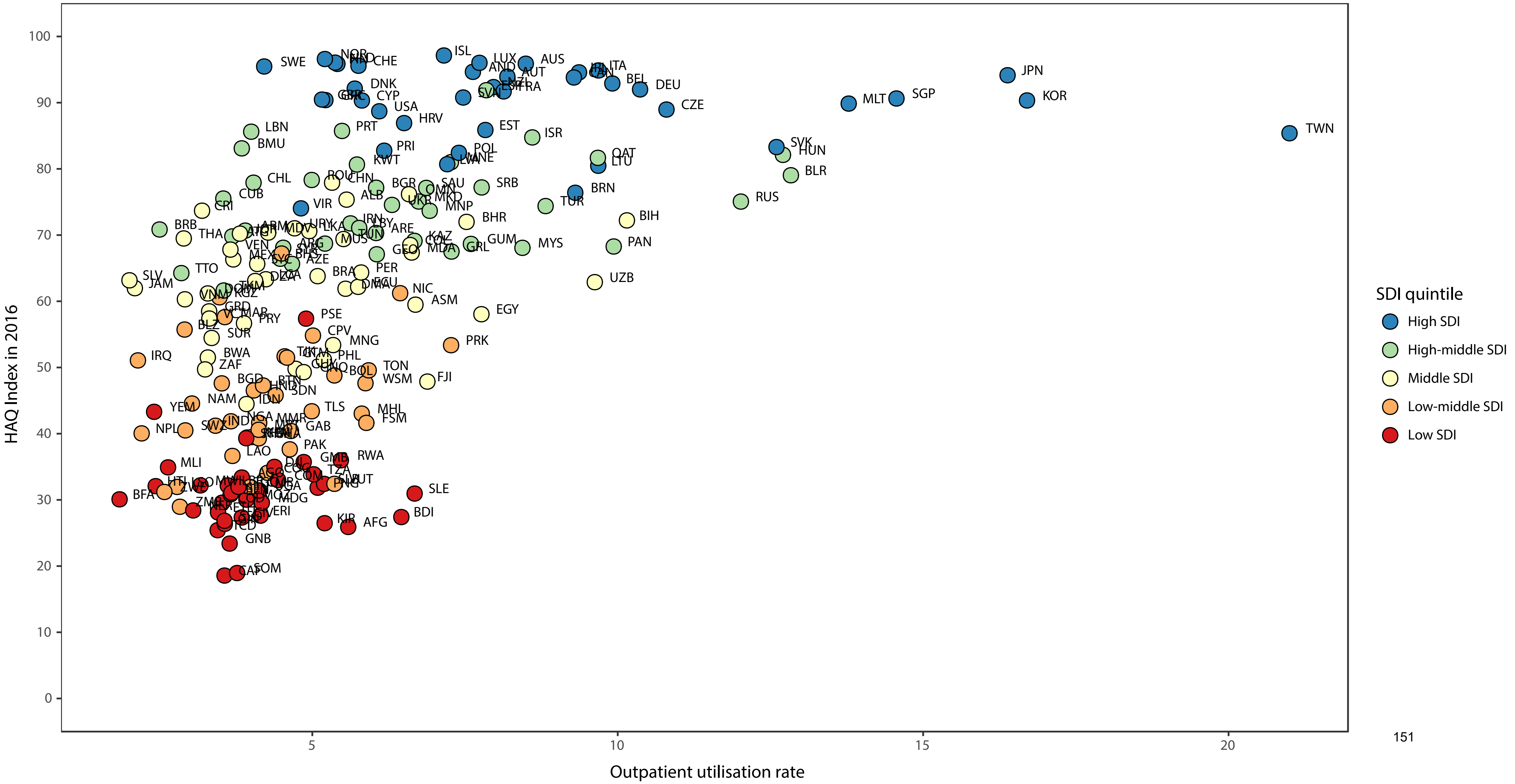

Supplementary figure 12. Comparing the HAQ Index in 2016 to inpatient utilisation rates (D). Inpatient utilisation rates are the annual number of admissions for one night or more to a health facility per capita in 2016. Countries and territories are colour-coded by their SDI quintile, and are abbreviated according to the ISO3 code. HAQ Index = Healthcare Access and Quality Index. SDI = Socio-demographic Index.

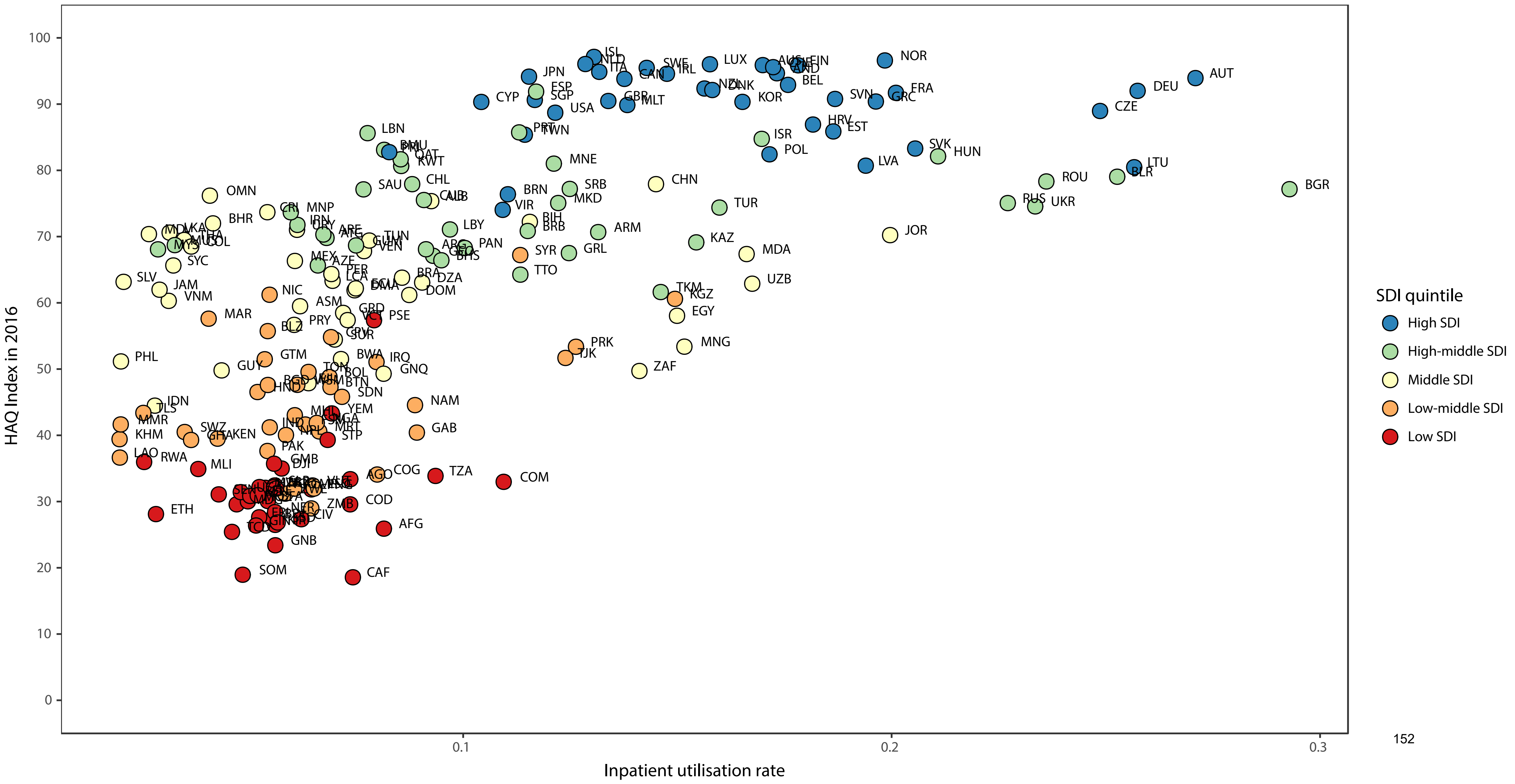

Supplementary figure 13. Comparing the PCA-derived HAQ Index with the arithmetic mean of the HAQ Index component parts, 2016. The correlation between these two index construction methods is 0.994. PCA = principal components analysis. HAQ Index = Healthcare Access and Quality Index.

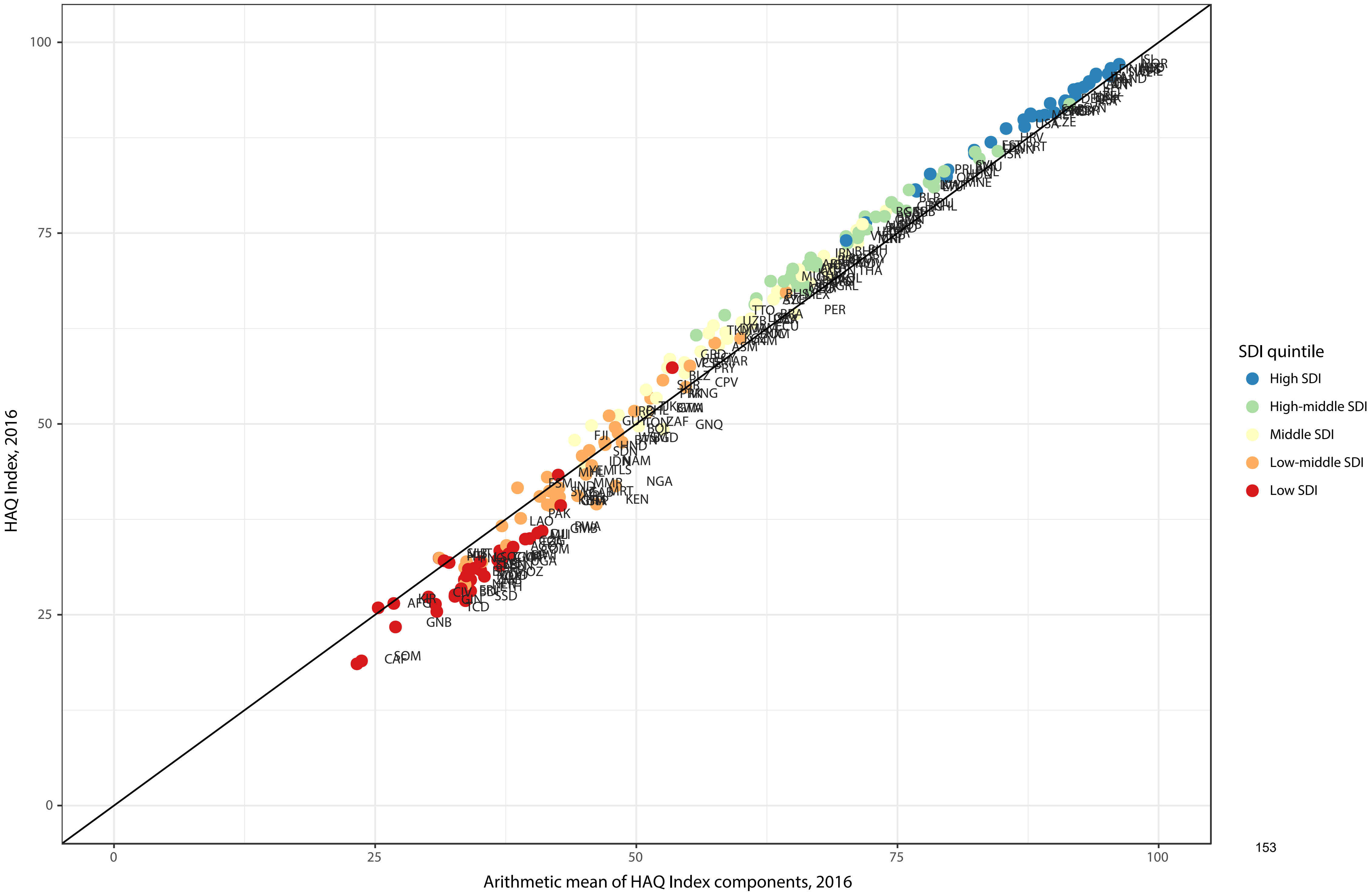

Supplementary figure 14. Comparing the GBD 2015 HAQ Index in 2015 to the GBD 2016 HAQ Index in 2016. GBD = Global Burden of Disease. HAQ Index = Healthcare Access and Quality Index.

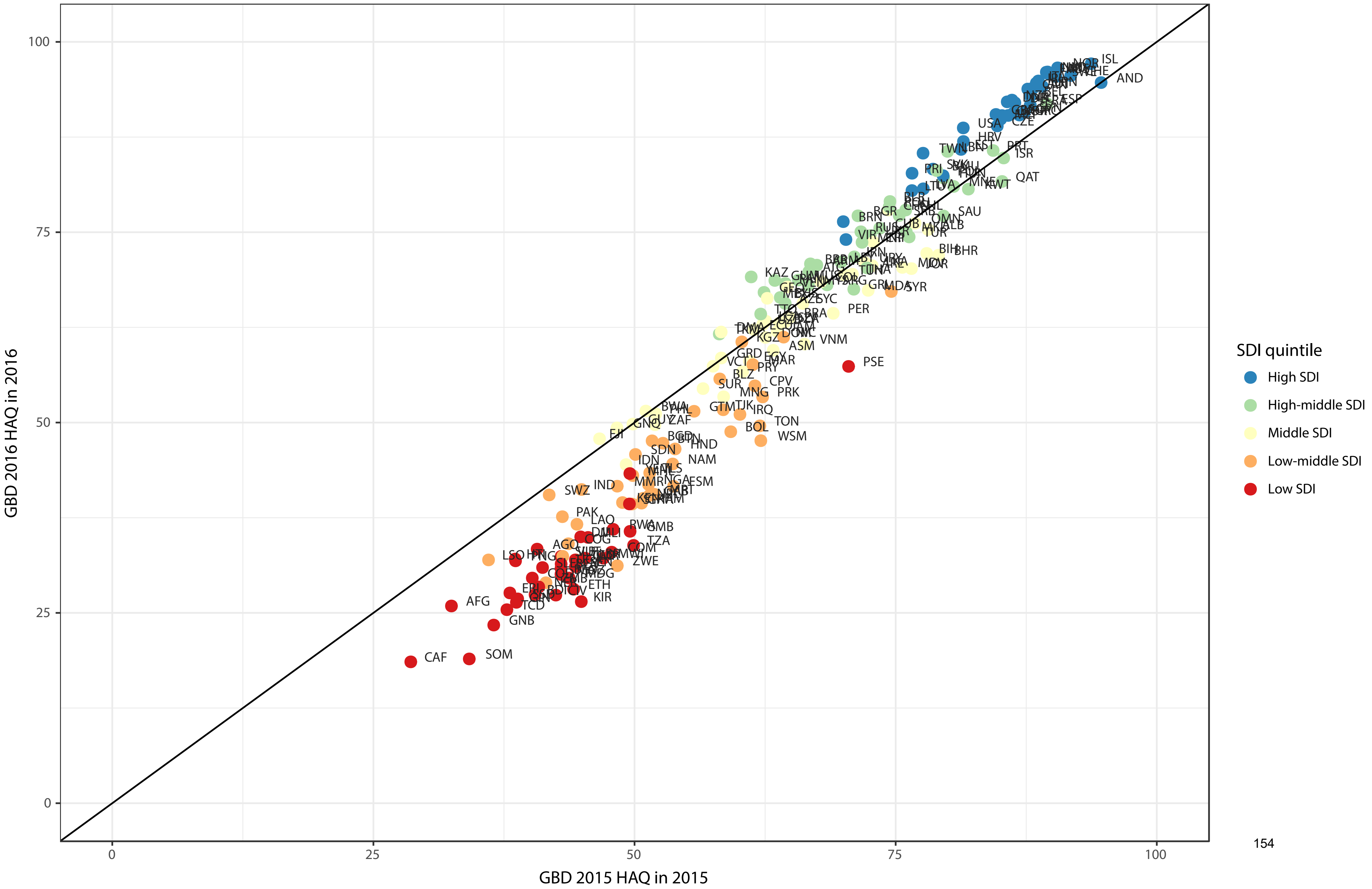

Supplementary figure 15. Comparing rankings for the GBD 2015 HAQ Index in 2015 to the GBD 2016 HAQ Index in 2016. GBD = Global Burden of Disease. HAQ Index = Healthcare Access and Quality Index.

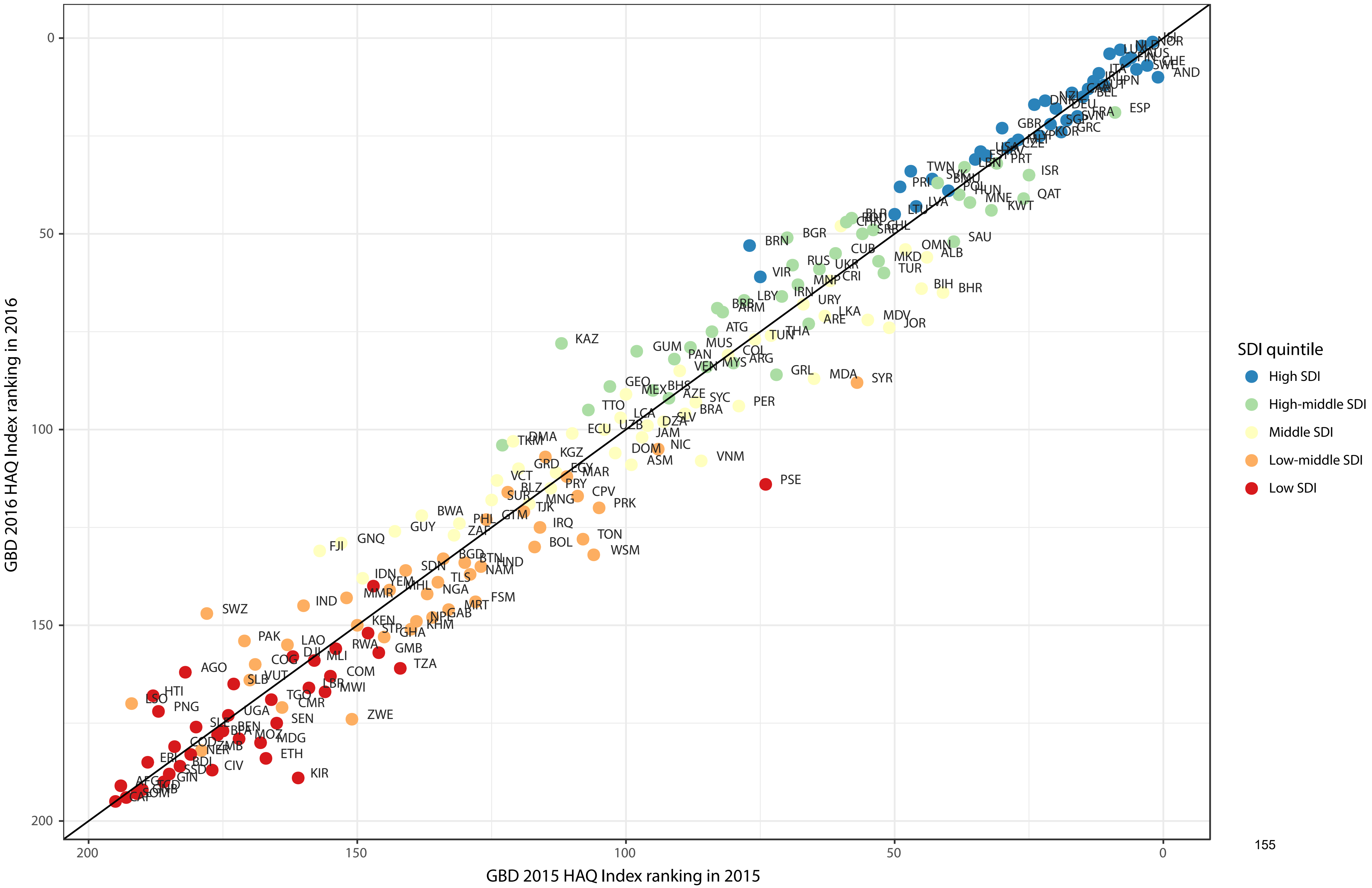

***Supplementary table 1.* List of ICD codes mapped to GBD causes for which mortality is amenable to healthcare.** ICD=International Classification of Diseases. GBD=Global Burden of Disease.

Cause	ICD-10	ICD-9
**Communicable, maternal, neonatal, and nutritional diseases**		
Tuberculosis	A10-A14, A15-A19.9, B90-B90.9, K67.3, K93.0, M49.0, P37.0	A10-A14, A15-A19.9, B90-B90.9, K67.3, K93.0, M49.0, P37.0
Diarrhoea, lower respiratory, and other common infectious diseases		
Diarrhoeal diseases	A00-A00.9, A02-A04.1, A04.3, A04.5-A07, A07.2-A07.4, A08-A09.9, R19.7	001-001.9, 003-006.9, 007.4-007.8, 008.01-008.02, 008.04, 008.2-009.9, 787.91
Lower respiratory infections	A48.1, A70, J09-J15.8, J16-J16.9, J20-J21.9, P23.0-P23.4, U04-U04.9	073.0-073.6, 466-469, 470.0, 480-482.89, 483.0-483.9, 484.1-484.2, 484.6-484.7, 487-489
Upper respiratory infections	J01-J01.91, J04.0, J05-J05.0, J05.11, J36-J36.0	461-461.9, 464.0, 464.01, 464.11-464.2, 464.21, 464.31-464.4, 464.8-464.9, 475-475.9, 476.9
Diphtheria	A36-A36.9	032-032.9
Whooping cough	A37-A37.91	033-033.9, 484.3-484.4
Tetanus	A33-A35.0	037-037.9, 771.3
Measles	B05-B05.9	055-055.9, 323.1, 484.0
Maternal disorders	N96, N98-N98.9, O00-O07.9, O09-O16.9, O20-O26.93, O28-O36.93, O40-O48.1, O60-O77.9, O80-O92.79, O96-O99.91	630-636.92, 638-638.92, 640-679.14
Neonatal disorders	P00-P04.2, P04.5-P05.9, P07-P15.9, P19-P22.9, P24-P29.9, P36-P36.9, P38-P39.9, P50-P61.9, P70, P70.3-P72.9, P74-P78.9, P80-P81.9, P83-P84, P90-P94.9, P96, P96.3-P96.4, P96.8-P96.89	760-760.64, 760.8-768, 768.2-770, 770.1-771, 771.4-775, 775.4-779.34, 779.6-779.89
**Non-communicable diseases**		
Neoplasms		
Colon and rectum cancer	C18-C21.9, D01.0-D01.3, D12-D12.9, D37.3-D37.5	153-154.9, 209.1-209.17, 209.5-209.57, 211.3-211.4, 230.3-230.6
Non-melanoma skin cancer (squamous-cell carcinoma)	C44-C44.99, D04-D04.9, D49.2	173-173.99, 222.4, 232-232.9, 238.2
Breast cancer	C50-C50.929, D05-D05.92, D24-D24.9, D48.6-D48.62, D49.3, N60-N60.99	174-175.9, 217-217.8, 233.0, 238.3, 239.3, 610-610.9
Cervical cancer	C53-C53.9, D06-D06.9, D26.0	180-180.9, 219.0, 233.1
Uterine cancer	C54-C54.9, D07.0-D07.2, N87-N87.9	182-182.8, 233.2
Testicular cancer	C62-C62.92, D29.2-D29.8, D40.1-D40.8	186-186.9, 222.0, 222.3, 236.4
Hodgkin lymphoma	C81-C81.99	201-201.98
Leukaemia	C91-C95.92	204-208.92
Cardiovascular diseases		
Rheumatic heart disease	I01-I01.9, I02.0, I05-I09.9	391-391.9, 392.0, 393-398.99
Ischaemic heart disease	I20-I25.9	410-414.9
Cerebrovascular disease	G45-G46.8, I60-I61.9, I62.0-I62.03, I63-I63.9, I65-I66.9, I67.0-I67.3, I67.5-I67.6, I68.1-I68.2, I69.0-I69.398	430-435.9, 437.0-437.2, 437.5-437.8
Hypertensive heart disease	I11-I11.9	402-402.91
Chronic respiratory diseases	D86-D86.2, D86.89-D86.9, G47.3-G47.39, J30-J35.9, J37-J47.9, J60-J63.8, J65-J68.9, J70-J70.1, J70.8-J70.9, J82, J84-J84.9, J91-J92.9	135-135.9, 136.6, 327.2-327.8, 470, 470.9-474.9, 476-476.1, 477-479, 490-504.9, 506-506.9, 508-509, 515, 516-517.8, 518.6, 518.9, 519.1-519.4, 780.57, 786.03
Digestive diseases		
Peptic ulcer disease	K25-K28.9, K31, K31.1-K31.6, K31.8, K31.82-K31.89	531-534.91
Appendicitis	K35-K37.9, K38.3-K38.9	540-542.9
Inguinal, femoral, and abdominal hernia	K40-K42.9, K44-K46.9	550-551.1, 551.3-552.1, 552.3-553.03, 553.6
Gallbladder and biliary diseases	K80-K83.9	574-576.9
Neurological disorders		
Epilepsy	G40-G41.9	345-345.91
Diabetes, urogenital, blood, and endocrine diseases		
Diabetes mellitus	E10-E10.11, E10.3-E11.1, E11.3-E12.1, E12.3-E13.11, E13.3-E14.1, E14.3-E14.9, P70.0-P70.2, R73-R73.9	250-250.39, 250.5-250.99, 357.2, 775.0-775.1, 790.2-790.22
Chronic kidney disease	D63.1, E10.2-E10.29, E11.2-E11.29, E12.2, E13.2-E13.29, E14.2, I12-I13.9, N02-N08.8, N15.0, N18-N18.9	250.4-250.49, 403-404.93, 581-583.9, 585-585.9, 589-589.9
Other non-communicable diseases		
Congenital heart anomalies	Q20-Q28.9	745-747.9
**Injuries**		
Unintentional injuries		
Adverse effects of medical treatment	Y38.9-Y84.9, Y88-Y88.3	E870-E876.9, E878-E879.9, E930-E949.9

Supplementary table 2 . **Factor loadings and weights from principal components analysis.**						
	**Iteration # 1**					**Cause weight**
	**Factor 1**	**Factor 2**	**Factor 3**	**Factor 4**	**Composite factor**	
**Cause**						
Tuberculosis	-0.2169	-0.0716	0.0388	-0.1215	-0.2080	0.0435
Diarrhoeal diseases	-0.2414	-0.0788	-0.0667	0.1275	-0.2301	0.0475
Lower respiratory infections	-0.1883	0.0261	0.1500	0.0465	-0.1419	0.0333
Upper respiratory infections	-0.0692	-0.1524	0.0241	-0.1062	-0.0357	0.0172
Diphtheria	-0.0603	-0.1762	-0.0005	-0.2314	-0.0390	0.0173
Whooping cough	-0.2236	-0.2036	-0.1934	-0.0496	-0.1855	0.0498
Tetanus	-0.1847	-0.3586	-0.0506	-0.1671	-0.1717	0.0452
Measles	-0.2353	-0.2912	-0.1199	-0.3638	-0.2149	0.0556
Maternal disorders	-0.2664	-0.1428	-0.0106	0.0940	-0.2336	0.0534
Neonatal disorders	-0.1916	0.0642	-0.0840	0.0038	-0.1614	0.0359
Non-melanoma skin cancer (squamous-cell carcinoma)	-0.1153	-0.0083	-0.0795	-0.0388	-0.1555	0.0233
Breast cancer	-0.2018	0.0323	-0.1710	-0.0155	-0.1313	0.0397
Cervical cancer	-0.1480	0.0202	-0.1499	0.0538	-0.1550	0.0290
Uterine cancer	-0.2216	0.0463	-0.2561	-0.0182	-0.1060	0.0442
Colon and rectum cancer	-0.1955	0.0579	-0.2137	-0.0607	-0.1797	0.0388
Testicular cancer	-0.2215	0.1123	-0.2165	0.0720	-0.1916	0.0415
Hodgkin lymphoma	-0.1900	0.2058	-0.2294	0.1390	-0.1646	0.0330
Leukaemia	-0.1276	0.2296	-0.2270	0.1077	-0.0958	0.0208
Rheumatic heart disease	-0.1557	0.1184	0.1956	-0.3784	-0.1305	0.0278
Ischaemic heart disease	-0.0392	0.3713	0.1119	-0.4268	-0.0232	0.0012
Cerebrovascular disease	-0.1304	0.2593	0.1598	-0.2810	-0.1068	0.0194
Hypertensive heart disease	-0.1425	0.1871	0.2964	-0.0751	-0.1437	0.0202
Chronic respiratory diseases	-0.1857	0.0291	0.0260	0.2063	-0.1315	0.0328
Peptic ulcer disease	-0.1816	-0.0078	0.2928	-0.1048	-0.1531	0.0324
Appendicitis	-0.2387	-0.0552	0.2252	0.2381	-0.2158	0.0423
Inguinal, femoral, and abdominal hernia	-0.2016	-0.1615	0.2855	0.2091	-0.1782	0.0372
Gallbladder and biliary diseases	-0.1740	-0.0679	0.3300	0.1362	-0.1649	0.0300
Epilepsy	-0.1377	0.0577	0.0775	-0.0053	-0.1139	0.0241
Diabetes mellitus	-0.1196	0.1824	0.2239	0.1311	-0.1188	0.0151
Chronic kidney disease	-0.1151	0.3150	0.0069	0.2493	-0.0991	0.0127
Congenital heart anomalies	-0.0459	0.3323	-0.1973	-0.1359	-0.0035	0.0045
Adverse effects of medical treatment	-0.1786	0.0683	0.0481	0.0727	-0.1597	0.0314
Variance explained	*0.6730*	*0.0801*	*0.0391*	*0.0279*	--	

Supplementary table 3. **Global, regional, national or territory, and select subnational estimates on the HAQ Index for 1990–2016, and absolute change and annualised rates of change for 1990–2016, 1990–2000, and 2000–2016.** Starred estimates of change represent statistically significant changes during a given time period. HAQ Index=Healthcare Access and Quality Index.

	HAQ Index						Absolute change			Annualised rate of change		
	1990	1995	2000	2005	2010	2016	1990-2016	1990-2000	2000-2016	1990-2016	1990-2000	2000-2016
**Global**	37·6 (36·8 to 38·8)	40·0 (39·2 to 40·9)	42·4 (41·6 to 43·2)	46·1 (45·3 to 46·9)	50·1 (49·3 to 51·0)	54·4 (53·5 to 55·4)	16·8 (15·2 to 18·0)*	4·7 (4·0 to 5·4)*	12·0 (10·9 to 13·1)*	1·42 (1·28 to 1·53)*	1·18 (0·99 to 1·36)*	1·56 (1·42 to 1·70)*
Southeast Asia, East Asia, and Oceania	37·1 (35·9 to 38·6)	41·0 (39·9 to 42·4)	44·9 (43·9 to 46·2)	50·7 (49·6 to 51·9)	57·0 (55·9 to 58·1)	62·9 (61·8 to 64·2)	25·9 (24·1 to 27·3)*	7·8 (6·9 to 8·8)*	18·0 (16·6 to 19·4)*	2·04 (1·88 to 2·16)*	1·92 (1·67 to 2·17)*	2·11 (1·93 to 2·27)*
East Asia	42·8 (41·4 to 44·6)	47·8 (46·5 to 49·5)	53·3 (52·1 to 54·9)	61·6 (60·2 to 63·2)	70·7 (69·3 to 71·9)	77·0 (75·5 to 78·1)	34·2 (31·7 to 35·9)*	10·5 (8·8 to 12·2)*	23·7 (21·7 to 25·3)*	2·26 (2·08 to 2·39)*	2·20 (1·80 to 2·56)*	2·30 (2·11 to 2·46)*
China	42·6 (41·2 to 44·5)	47·6 (46·2 to 49·4)	53·3 (52·0 to 55·1)	62·0 (60·5 to 63·9)	71·3 (69·8 to 72·4)	77·9 (76·5 to 78·9)	35·3 (32·8 to 37·0)*	10·8 (8·8 to 12·6)*	24·6 (22·4 to 26·2)*	2·33 (2·13 to 2·46)*	2·25 (1·83 to 2·63)*	2·37 (2·15 to 2·54)*
Anhui	45·0 (41·7 to 48·6)	50·1 (46·5 to 53·6)	55·4 (51·9 to 58·7)	63·1 (59·7 to 65·8)	71·4 (68·1 to 73·7)	77·0 (73·3 to 79·6)	32·0 (27·3 to 36·0)*	10·4 (6·6 to 14·6)*	21·5 (17·5 to 25·2)*	2·07 (1·74 to 2·37)*	2·09 (1·31 to 2·95)*	2·05 (1·65 to 2·42)*
Beijing	60·5 (57·4 to 63·9)	64·7 (61·5 to 68·0)	70·9 (68·1 to 73·6)	78·7 (75·9 to 81·2)	86·0 (83·7 to 88·0)	91·5 (89·1 to 93·6)	31·0 (26·8 to 34·6)*	10·4 (6·9 to 13·9)*	20·5 (17·0 to 24·1)*	1·59 (1·36 to 1·81)*	1·59 (1·05 to 2·16)*	1·59 (1·30 to 1·88)*
Chongqing	42·6 (38·5 to 46·8)	48·4 (44·6 to 52·1)	54·2 (50·7 to 57·6)	63·0 (59·1 to 66·4)	71·1 (67·4 to 74·0)	76·7 (72·9 to 80·0)	34·0 (29·1 to 38·7)*	11·6 (7·1 to 15·9)*	22·4 (18·1 to 27·1)*	2·26 (1·88 to 2·66)*	2·41 (1·43 to 3·35)*	2·17 (1·73 to 2·62)*
Fujian	45·8 (42·4 to 49·3)	52·0 (48·9 to 55·0)	57·3 (54·4 to 60·2)	64·5 (62·0 to 67·0)	73·4 (71·2 to 75·3)	81·2 (78·5 to 83·8)	35·4 (30·9 to 39·4)*	11·4 (7·6 to 15·3)*	24·0 (19·9 to 27·6)*	2·20 (1·89 to 2·50)*	2·23 (1·45 to 3·04)*	2·19 (1·81 to 2·54)*
Gansu	38·6 (35·4 to 42·1)	42·4 (39·5 to 45·5)	47·6 (44·9 to 50·5)	55·7 (53·1 to 58·7)	64·5 (62·0 to 66·8)	70·3 (67·6 to 72·8)	31·7 (27·2 to 35·8)*	9·0 (5·1 to 12·8)*	22·7 (18·9 to 26·5)*	2·31 (1·94 to 2·66)*	2·09 (1·15 to 3·04)*	2·44 (2·00 to 2·86)*
Guangdong	47·6 (44·4 to 51·2)	52·6 (49·4 to 56·0)	58·5 (55·5 to 61·7)	67·4 (64·7 to 69·7)	76·8 (74·6 to 78·9)	83·9 (81·2 to 86·2)	36·3 (32·3 to 40·5)*	11·0 (6·9 to 14·8)*	25·3 (21·3 to 29·5)*	2·18 (1·89 to 2·47)*	2·08 (1·29 to 2·85)*	2·25 (1·88 to 2·63)*
Guangxi	38·8 (35·6 to 42·4)	44·0 (41·0 to 47·4)	49·9 (47·1 to 53·4)	57·5 (54·6 to 60·7)	66·2 (63·7 to 68·8)	73·1 (69·8 to 76·1)	34·3 (29·6 to 38·6)*	11·0 (7·4 to 14·8)*	23·2 (19·0 to 27·3)*	2·44 (2·08 to 2·80)*	2·51 (1·66 to 3·47)*	2·39 (1·94 to 2·83)*
Guizhou	29·8 (26·2 to 33·9)	33·3 (29·7 to 37·0)	37·6 (34·4 to 40·8)	45·7 (42·7 to 48·6)	55·5 (52·4 to 58·6)	61·5 (58·0 to 65·0)	31·7 (26·9 to 36·5)*	7·7 (3·7 to 11·5)*	24·0 (19·3 to 28·3)*	2·79 (2·26 to 3·31)*	2·32 (1·06 to 3·56)*	3·09 (2·46 to 3·68)*
Hainan	41·6 (38·1 to 46·1)	45·9 (42·4 to 49·9)	50·9 (47·7 to 54·5)	58·3 (55·0 to 61·4)	67·5 (64·8 to 70·1)	73·4 (70·2 to 76·6)	31·8 (26·7 to 36·5)*	9·3 (5·5 to 13·1)*	22·5 (17·9 to 26·9)*	2·19 (1·79 to 2·56)*	2·02 (1·16 to 2·90)*	2·29 (1·80 to 2·76)*
Hebei	49·3 (46·4 to 52·2)	53·1 (50·2 to 56·0)	58·4 (55·6 to 61·5)	67·0 (64·0 to 70·0)	74·7 (72·1 to 77·1)	79·9 (77·2 to 82·5)	30·7 (26·6 to 34·3)*	9·2 (5·7 to 12·7)*	21·5 (17·5 to 25·1)*	1·86 (1·60 to 2·10)*	1·71 (1·07 to 2·41)*	1·96 (1·58 to 2·30)*
Heilongjiang	48·9 (45·5 to 52·5)	53·8 (50·2 to 57·4)	59·3 (55·1 to 62·9)	67·3 (63·3 to 70·6)	74·2 (70·5 to 77·1)	78·5 (74·7 to 81·7)	29·6 (24·5 to 33·9)*	10·4 (6·2 to 14·3)*	19·2 (14·7 to 23·6)*	1·82 (1·50 to 2·12)*	1·93 (1·16 to 2·66)*	1·76 (1·34 to 2·17)*
Henan	44·6 (41·7 to 47·6)	48·8 (45·8 to 52·0)	54·7 (52·0 to 57·7)	63·4 (60·6 to 65·9)	72·1 (69·2 to 74·2)	78·0 (74·9 to 80·3)	33·4 (29·5 to 37·2)*	10·1 (6·7 to 13·6)*	23·3 (19·4 to 26·9)*	2·15 (1·87 to 2·43)*	2·04 (1·35 to 2·74)*	2·22 (1·84 to 2·59)*
Hong Kong Special Administrative Region of China	71·8 (68·9 to 75·8)	76·8 (74·3 to 80·1)	80·8 (78·7 to 83·2)	83·7 (82·0 to 85·9)	86·8 (85·0 to 88·9)	89·5 (87·4 to 91·8)	17·8 (14·3 to 21·1)*	9·0 (6·1 to 11·8)*	8·8 (6·2 to 11·2)*	0·85 (0·67 to 1·02)*	1·19 (0·78 to 1·58)*	0·64 (0·45 to 0·83)*
Hubei	44·2 (41·1 to 47·8)	51·5 (48·7 to 54·4)	58·0 (55·4 to 60·8)	65·8 (63·1 to 68·3)	73·1 (70·7 to 75·1)	79·1 (76·1 to 81·5)	34·9 (30·2 to 39·1)*	13·8 (9·8 to 17·4)*	21·1 (16·9 to 24·7)*	2·24 (1·91 to 2·55)*	2·72 (1·90 to 3·50)*	1·94 (1·55 to 2·28)*
Hunan	41·7 (38·7 to 45·1)	47·1 (44·0 to 50·1)	52·9 (50·2 to 55·8)	61·5 (59·1 to 63·9)	70·1 (67·9 to 71·9)	75·8 (73·3 to 78·0)	34·1 (29·9 to 37·8)*	11·2 (7·5 to 14·9)*	22·9 (19·1 to 26·4)*	2·30 (1·98 to 2·61)*	2·38 (1·56 to 3·21)*	2·25 (1·86 to 2·62)*
Inner Mongolia	44·8 (41·7 to 48·3)	50·6 (47·4 to 53·7)	57·6 (54·7 to 60·7)	66·2 (63·2 to 69·2)	74·1 (71·5 to 76·5)	79·9 (76·8 to 82·8)	35·0 (30·5 to 39·1)*	12·8 (9·0 to 16·6)*	22·2 (18·1 to 26·0)*	2·22 (1·92 to 2·53)*	2·52 (1·73 to 3·27)*	2·04 (1·65 to 2·41)*
Jiangsu	51·7 (48·9 to 54·9)	57·2 (54·5 to 59·9)	63·8 (61·1 to 66·3)	71·9 (69·2 to 74·4)	79·7 (77·2 to 81·7)	85·9 (83·2 to 88·0)	34·1 (30·3 to 37·8)*	12·0 (8·4 to 15·9)*	22·1 (18·7 to 25·3)*	1·95 (1·70 to 2·20)*	2·09 (1·44 to 2·78)*	1·86 (1·55 to 2·15)*
Jiangxi	38·8 (35·6 to 42·1)	44·1 (41·2 to 47·4)	49·9 (47·2 to 52·8)	59·4 (56·7 to 62·0)	68·1 (65·9 to 70·1)	73·9 (71·1 to 76·6)	35·1 (30·9 to 39·2)*	11·1 (7·3 to 15·0)*	24·0 (20·2 to 27·6)*	2·49 (2·16 to 2·84)*	2·52 (1·64 to 3·47)*	2·46 (2·04 to 2·85)*
Jilin	46·9 (43·7 to 50·1)	52·8 (49·7 to 56·0)	59·2 (55·8 to 62·6)	67·6 (64·3 to 70·7)	74·4 (71·6 to 77·1)	79·3 (76·2 to 82·1)	32·4 (28·1 to 36·7)*	12·3 (8·4 to 16·1)*	20·1 (15·7 to 24·3)*	2·02 (1·72 to 2·32)*	2·33 (1·58 to 3·07)*	1·83 (1·42 to 2·25)*
Liaoning	53·7 (50·8 to 56·9)	57·9 (54·8 to 60·9)	63·6 (60·6 to 66·5)	71·7 (68·7 to 74·2)	78·5 (75·8 to 80·6)	83·1 (80·1 to 85·6)	29·4 (24·7 to 33·4)*	9·8 (6·0 to 13·5)*	19·5 (15·8 to 23·5)*	1·68 (1·40 to 1·93)*	1·68 (1·02 to 2·33)*	1·68 (1·34 to 2·03)*
Macao Special Administrative Region of China	68·0 (65·2 to 70·9)	74·0 (71·6 to 76·4)	77·4 (75·1 to 79·7)	81·7 (79·5 to 83·9)	85·5 (83·3 to 88·0)	89·3 (86·8 to 92·2)	21·2 (17·4 to 25·3)*	9·4 (6·4 to 12·7)*	11·8 (8·6 to 15·1)*	1·05 (0·85 to 1·25)*	1·29 (0·88 to 1·76)*	0·89 (0·65 to 1·14)*
Ningxia	42·8 (39·6 to 45·9)	47·4 (44·1 to 50·7)	53·4 (50·1 to 56·5)	61·0 (57·9 to 63·9)	68·9 (66·1 to 71·4)	74·7 (71·4 to 77·6)	31·9 (27·3 to 36·1)*	10·6 (7·0 to 14·3)*	21·3 (17·2 to 25·3)*	2·15 (1·81 to 2·46)*	2·22 (1·45 to 2·99)*	2·10 (1·69 to 2·52)*
Qinghai	31·7 (27·9 to 36·5)	35·5 (32·1 to 39·8)	39·9 (36·4 to 43·8)	47·4 (43·8 to 51·1)	56·4 (53·1 to 59·5)	62·1 (58·2 to 65·6)	30·4 (25·1 to 35·6)*	8·2 (4·0 to 12·5)*	22·2 (17·5 to 27·2)*	2·60 (2·06 to 3·12)*	2·32 (1·08 to 3·62)*	2·77 (2·13 to 3·45)*
Shaanxi	39·9 (36·7 to 43·2)	45·1 (41·8 to 48·3)	51·0 (48·0 to 54·3)	59·5 (56·3 to 62·8)	68·6 (65·9 to 71·3)	74·8 (71·7 to 77·7)	35·0 (30·3 to 39·4)*	11·1 (7·1 to 15·3)*	23·8 (19·4 to 28·2)*	2·42 (2·07 to 2·77)*	2·47 (1·57 to 3·42)*	2·40 (1·94 to 2·85)*
Shandong	51·4 (48·3 to 54·5)	56·8 (54·2 to 59·8)	62·5 (59·9 to 65·0)	69·9 (67·0 to 72·3)	76·8 (74·4 to 78·9)	83·2 (80·3 to 85·6)	31·8 (27·7 to 35·5)*	11·1 (7·3 to 14·8)*	20·7 (16·9 to 24·0)*	1·86 (1·60 to 2·11)*	1·96 (1·26 to 2·65)*	1·79 (1·47 to 2·08)*
Shanghai	63·1 (59·7 to 66·5)	66·7 (63·3 to 69·9)	72·4 (69·7 to 75·0)	79·3 (76·6 to 81·6)	84·8 (82·4 to 87·0)	89·6 (87·2 to 91·8)	26·5 (22·2 to 30·7)*	9·3 (5·4 to 13·0)*	17·2 (13·9 to 20·6)*	1·35 (1·11 to 1·58)*	1·37 (0·79 to 1·95)*	1·33 (1·07 to 1·61)*
Shanxi	47·4 (44·4 to 50·6)	52·4 (49·4 to 55·6)	58·1 (55·2 to 61·0)	66·4 (63·3 to 69·2)	73·6 (70·8 to 76·1)	78·8 (75·6 to 81·3)	31·3 (27·2 to 35·4)*	10·6 (7·1 to 14·3)*	20·7 (16·3 to 24·3)*	1·95 (1·68 to 2·25)*	2·02 (1·33 to 2·74)*	1·91 (1·51 to 2·25)*
Sichuan	38·2 (34·8 to 41·7)	43·4 (40·3 to 46·7)	49·0 (46·1 to 52·3)	57·3 (54·2 to 60·6)	67·1 (64·1 to 69·9)	73·7 (70·4 to 76·9)	35·5 (31·0 to 39·8)*	10·8 (6·8 to 14·6)*	24·7 (20·6 to 28·6)*	2·53 (2·16 to 2·89)*	2·50 (1·55 to 3·41)*	2·55 (2·12 to 2·98)*
Tianjin	58·9 (55·4 to 61·9)	63·5 (60·3 to 66·5)	69·6 (66·7 to 72·4)	76·7 (73·9 to 79·4)	83·7 (81·1 to 85·9)	88·6 (85·9 to 90·8)	29·6 (25·7 to 33·6)*	10·6 (6·8 to 14·5)*	19·0 (15·4 to 22·3)*	1·57 (1·34 to 1·80)*	1·66 (1·05 to 2·28)*	1·51 (1·21 to 1·79)*
Tibet	26·1 (22·4 to 31·2)	28·2 (24·5 to 33·3)	30·6 (27·2 to 35·0)	35·7 (32·6 to 40·7)	43·3 (39·8 to 48·5)	48·0 (43·5 to 53·2)	22·0 (17·0 to 27·5)*	4·5 (1·2 to 7·9)*	17·4 (12·5 to 22·3)*	2·36 (1·77 to 3·03)*	1·61 (0·43 to 2·84)*	2·82 (2·04 to 3·62)*
Xinjiang	32·6 (28·8 to 37·0)	37·0 (33·8 to 40·8)	43·0 (39·9 to 46·2)	51·0 (48·0 to 54·4)	60·3 (57·5 to 63·4)	67·0 (63·1 to 70·4)	34·5 (29·0 to 39·6)*	10·4 (6·2 to 14·7)*	24·0 (19·0 to 28·6)*	2·78 (2·26 to 3·29)*	2·79 (1·61 to 4·01)*	2·78 (2·21 to 3·32)*
Yunnan	32·7 (29·6 to 37·2)	37·1 (34·0 to 41·4)	41·6 (38·5 to 45·8)	48·9 (46·1 to 52·5)	59·2 (56·4 to 62·3)	66·4 (63·2 to 69·6)	33·8 (29·0 to 37·9)*	8·9 (5·1 to 12·4)*	24·8 (20·4 to 28·9)*	2·74 (2·25 to 3·15)*	2·42 (1·34 to 3·40)*	2·93 (2·33 to 3·46)*
Zhejiang	52·8 (49·7 to 56·0)	58·5 (55·5 to 61·5)	64·7 (61·8 to 67·4)	72·6 (70·1 to 75·0)	79·7 (77·1 to 82·0)	86·3 (83·4 to 88·8)	33·4 (29·4 to 37·7)*	11·8 (8·2 to 15·6)*	21·6 (17·7 to 25·5)*	1·89 (1·64 to 2·16)*	2·02 (1·39 to 2·69)*	1·80 (1·46 to 2·14)*
North Korea	49·6 (46·2 to 52·9)	50·7 (47·2 to 54·4)	47·6 (44·1 to 51·2)	47·8 (44·1 to 51·3)	51·4 (47·8 to 54·7)	53·4 (49·6 to 56·9)	3·8 (-1·3 to 8·2)	-1·9 (-6·2 to 2·0)	5·7 (1·2 to 10·2)*	0·28 (-0·10 to 0·62)	-0·40 (-1·26 to 0·41)	0·71 (0·15 to 1·26)*
Taiwan (Province of China)	60·6 (58·6 to 62·7)	65·5 (63·5 to 67·6)	71·8 (69·9 to 73·7)	78·0 (76·3 to 79·8)	82·9 (81·2 to 84·5)	85·4 (82·5 to 88·2)	24·8 (21·4 to 28·1)*	11·2 (8·6 to 13·6)*	13·6 (10·2 to 16·7)*	1·32 (1·14 to 1·49)*	1·70 (1·30 to 2·07)*	1·08 (0·82 to 1·32)*
Oceania	27·2 (22·9 to 31·0)	29·0 (24·9 to 32·8)	32·4 (28·4 to 36·3)	32·7 (28·6 to 36·8)	33·1 (28·9 to 37·1)	36·0 (31·8 to 40·4)	8·8 (4·0 to 13·5)*	5·2 (1·9 to 8·5)*	3·6 (-0·5 to 7·8)	1·08 (0·49 to 1·66)*	1·76 (0·62 to 2·97)*	0·66 (-0·10 to 1·44)
American Samoa	47·6 (44·6 to 50·6)	49·7 (46·3 to 53·2)	55·9 (52·9 to 59·1)	57·3 (54·0 to 60·1)	59·4 (56·3 to 62·5)	59·5 (55·0 to 64·1)	11·9 (6·5 to 17·4)*	8·3 (4·1 to 12·5)*	3·6 (-1·8 to 8·9)	0·86 (0·46 to 1·23)*	1·61 (0·79 to 2·42)*	0·38 (-0·20 to 0·96)
Federated States of Micronesia	27·9 (23·4 to 32·5)	30·7 (25·8 to 35·5)	32·2 (27·2 to 37·1)	37·5 (31·8 to 42·7)	37·9 (31·4 to 44·3)	41·6 (34·8 to 49·1)	13·7 (5·8 to 21·4)*	4·3 (0·0 to 8·0)	9·4 (2·3 to 17·2)*	1·54 (0·68 to 2·40)*	1·44 (0·02 to 2·72)*	1·59 (0·44 to 2·77)*
Fiji	41·0 (34·8 to 47·2)	42·0 (37·0 to 46·6)	43·3 (39·7 to 47·0)	43·8 (40·7 to 47·1)	45·4 (41·8 to 49·3)	47·9 (41·9 to 54·3)	6·8 (-1·9 to 15·4)	2·2 (-4·0 to 8·6)	4·6 (-2·4 to 11·8)	0·59 (-0·17 to 1·35)	0·55 (-0·92 to 2·16)	0·62 (-0·34 to 1·59)
Guam	61·9 (59·0 to 64·9)	67·1 (64·0 to 70·0)	71·3 (68·7 to 74·0)	71·4 (68·7 to 74·0)	69·6 (66·9 to 72·6)	68·7 (64·8 to 72·9)	6·7 (2·0 to 11·6)*	9·4 (5·6 to 13·4)*	-2·7 (-7·5 to 2·5)	0·40 (0·12 to 0·67)*	1·41 (0·83 to 2·03)*	-0·24 (-0·67 to 0·21)
Kiribati	20·3 (17·0 to 23·8)	21·0 (18·1 to 24·1)	23·0 (19·9 to 26·3)	23·7 (20·4 to 27·5)	25·6 (21·5 to 30·0)	26·5 (21·4 to 31·1)	6·2 (1·0 to 11·1)*	2·7 (-1·0 to 6·0)	3·4 (-1·1 to 7·9)	1·02 (0·19 to 1·81)*	1·27 (-0·49 to 2·79)	0·86 (-0·27 to 1·95)
Marshall Islands	33·1 (30·4 to 36·1)	34·5 (31·7 to 37·6)	34·5 (31·1 to 38·0)	37·3 (34·0 to 40·8)	39·4 (34·9 to 43·8)	43·0 (38·0 to 48·2)	9·9 (4·3 to 15·1)*	1·3 (-2·5 to 5·3)	8·6 (3·5 to 13·7)*	1·00 (0·46 to 1·50)*	0·38 (-0·76 to 1·54)	1·39 (0·56 to 2·17)*
Northern Mariana Islands	61·5 (56·0 to 67·0)	68·7 (64·2 to 73·4)	71·9 (67·7 to 75·9)	75·1 (71·3 to 79·1)	74·8 (70·7 to 78·6)	73·7 (69·2 to 78·3)	12·2 (5·4 to 19·4)*	10·4 (5·6 to 15·1)*	1·8 (-3·8 to 7·4)	0·70 (0·30 to 1·12)*	1·56 (0·83 to 2·37)*	0·15 (-0·33 to 0·64)
Papua New Guinea	22·9 (17·8 to 27·7)	24·7 (19·8 to 29·7)	28·5 (23·2 to 33·6)	28·6 (23·4 to 34·0)	28·8 (23·4 to 34·3)	31·8 (26·2 to 37·4)	8·9 (2·7 to 15·1)*	5·6 (1·4 to 9·8)*	3·3 (-2·1 to 8·6)	1·27 (0·37 to 2·15)*	2·19 (0·52 to 3·95)*	0·70 (-0·43 to 1·85)
Samoa	37·4 (32·8 to 41·7)	39·9 (35·4 to 44·5)	43·6 (38·8 to 48·2)	45·5 (40·7 to 50·4)	46·7 (41·9 to 51·9)	47·6 (42·8 to 52·6)	10·3 (4·5 to 16·1)*	6·3 (2·6 to 9·7)*	4·0 (-1·2 to 9·0)	0·93 (0·41 to 1·48)*	1·56 (0·67 to 2·43)*	0·55 (-0·16 to 1·21)
Solomon Islands	26·7 (21·2 to 32·3)	28·5 (23·1 to 33·9)	31·4 (25·9 to 36·8)	29·1 (23·7 to 34·4)	28·8 (23·9 to 33·9)	32·4 (27·1 to 37·7)	5·8 (-0·9 to 12·3)	4·8 (0·4 to 8·8)*	1·0 (-4·6 to 6·2)	0·76 (-0·11 to 1·65)	1·66 (0·14 to 3·15)*	0·20 (-0·87 to 1·24)
Tonga	38·4 (33·7 to 42·9)	39·3 (34·7 to 44·1)	42·8 (38·4 to 47·2)	44·8 (40·3 to 49·1)	46·7 (42·0 to 51·3)	49·6 (44·4 to 54·4)	11·2 (5·0 to 17·4)*	4·4 (0·4 to 8·3)*	6·8 (1·7 to 11·8)*	0·99 (0·44 to 1·55)*	1·10 (0·08 to 2·11)*	0·92 (0·22 to 1·60)*
Vanuatu	28·2 (23·2 to 33·1)	27·9 (23·3 to 32·6)	28·7 (24·0 to 33·2)	28·4 (23·7 to 32·9)	30·4 (25·1 to 35·3)	32·4 (26·9 to 37·5)	4·3 (-2·0 to 10·3)	0·6 (-3·4 to 4·8)	3·7 (-1·9 to 8·9)	0·55 (-0·26 to 1·36)	0·21 (-1·17 to 1·73)	0·75 (-0·39 to 1·82)
Southeast Asia	29·3 (27·8 to 30·8)	32·0 (30·5 to 33·7)	34·5 (33·0 to 36·0)	38·1 (36·6 to 39·6)	42·4 (41·0 to 44·0)	47·5 (45·9 to 49·2)	18·1 (16·4 to 20·0)*	5·1 (4·0 to 6·2)*	13·0 (11·4 to 14·6)*	1·85 (1·67 to 2·05)*	1·61 (1·25 to 1·97)*	2·00 (1·76 to 2·27)*
Cambodia	20·3 (17·7 to 23·6)	21·2 (18·9 to 23·7)	23·0 (20·9 to 25·3)	29·3 (27·0 to 32·1)	32·5 (29·9 to 35·4)	39·4 (36·4 to 42·5)	19·1 (14·8 to 23·0)*	2·7 (-0·7 to 5·7)	16·5 (13·0 to 19·9)*	2·56 (1·90 to 3·12)*	1·25 (-0·33 to 2·76)	3·38 (2·65 to 4·10)*
Indonesia	28·9 (26·4 to 31·7)	31·4 (29·3 to 34·1)	33·0 (31·1 to 35·3)	35·9 (34·1 to 38·1)	39·6 (37·9 to 41·8)	44·5 (42·6 to 46·8)	15·6 (12·8 to 18·4)*	4·1 (1·8 to 6·0)*	11·5 (9·2 to 13·8)*	1·67 (1·33 to 2·01)*	1·34 (0·57 to 2·00)*	1·87 (1·49 to 2·27)*
Laos	18·0 (15·4 to 21·4)	19·6 (16·9 to 22·4)	21·8 (18·8 to 24·7)	24·9 (22·1 to 28·1)	30·3 (26·8 to 34·1)	36·6 (32·6 to 41·1)	18·6 (13·6 to 24·0)*	3·8 (0·4 to 7·1)*	14·8 (10·2 to 19·6)*	2·74 (1·97 to 3·51)*	1·94 (0·21 to 3·58)*	3·24 (2·23 to 4·25)*
Malaysia	44·2 (42·5 to 46·1)	49·3 (47·6 to 51·2)	54·2 (52·6 to 55·9)	59·5 (57·6 to 61·3)	61·7 (59·9 to 63·5)	68·1 (65·9 to 70·2)	23·9 (21·3 to 26·6)*	10·0 (7·8 to 12·3)*	13·9 (11·5 to 16·2)*	1·66 (1·47 to 1·85)*	2·05 (1·59 to 2·51)*	1·43 (1·19 to 1·66)*
Maldives	37·6 (33·6 to 41·0)	44·0 (40·2 to 47·2)	52·7 (49·9 to 55·4)	58·2 (55·8 to 60·6)	65·1 (62·8 to 67·3)	70·4 (65·7 to 74·8)	32·8 (26·9 to 39·1)*	15·1 (11·9 to 18·6)*	17·6 (11·9 to 22·8)*	2·41 (1·98 to 2·92)*	3·39 (2·62 to 4·32)*	1·80 (1·24 to 2·29)*
Mauritius	53·9 (52·6 to 55·3)	59·0 (57·2 to 60·6)	61·9 (60·4 to 63·2)	63·5 (62·0 to 64·9)	65·3 (64·0 to 66·6)	68·7 (65·5 to 71·9)	14·8 (11·6 to 18·0)*	8·0 (6·4 to 9·4)*	6·8 (3·6 to 10·0)*	0·93 (0·75 to 1·12)*	1·38 (1·10 to 1·64)*	0·65 (0·35 to 0·94)*
Myanmar	19·9 (17·2 to 22·6)	21·1 (18·3 to 24·1)	23·1 (20·2 to 26·0)	27·4 (24·3 to 30·6)	33·0 (30·0 to 36·2)	41·6 (38·0 to 45·5)	21·7 (17·4 to 26·4)*	3·1 (-0·2 to 6·2)	18·6 (14·5 to 22·5)*	2·84 (2·29 to 3·44)*	1·46 (-0·08 to 2·86)	3·70 (2·82 to 4·54)*
Philippines	39·0 (37·3 to 40·6)	40·9 (39·3 to 42·7)	42·7 (40·7 to 44·5)	44·7 (42·7 to 46·3)	47·8 (45·9 to 49·4)	51·2 (47·9 to 54·4)	12·2 (8·7 to 15·8)*	3·8 (1·9 to 5·7)*	8·4 (4·8 to 11·9)*	1·05 (0·76 to 1·33)*	0·92 (0·46 to 1·41)*	1·12 (0·65 to 1·56)*
Seychelles	45·9 (43·8 to 48·1)	52·4 (50·0 to 54·9)	57·3 (55·2 to 59·4)	59·7 (57·7 to 61·8)	62·6 (60·5 to 64·6)	65·6 (62·2 to 68·9)	19·8 (16·1 to 23·5)*	11·4 (8·6 to 14·0)*	8·4 (4·6 to 12·0)*	1·38 (1·12 to 1·63)*	2·22 (1·67 to 2·75)*	0·85 (0·48 to 1·22)*
Sri Lanka	47·4 (45·1 to 49·8)	52·1 (48·8 to 54·8)	54·4 (52·1 to 56·9)	59·5 (57·2 to 61·6)	64·7 (62·4 to 67·0)	70·6 (66·3 to 75·3)	23·2 (18·5 to 28·1)*	7·0 (3·8 to 10·2)*	16·2 (11·5 to 21·0)*	1·53 (1·24 to 1·84)*	1·38 (0·75 to 2·02)*	1·62 (1·16 to 2·08)*
Thailand	44·4 (42·4 to 46·6)	49·2 (46·8 to 51·5)	54·7 (52·2 to 57·4)	60·6 (58·2 to 63·1)	64·9 (62·5 to 67·4)	69·5 (66·5 to 72·6)	25·1 (21·4 to 28·7)*	10·3 (7·2 to 13·3)*	14·8 (10·9 to 18·6)*	1·72 (1·46 to 1·95)*	2·09 (1·49 to 2·66)*	1·49 (1·09 to 1·88)*
Timor-Leste	22·2 (17·2 to 27·8)	25·0 (20·8 to 31·1)	27·3 (23·0 to 34·5)	32·4 (28·0 to 40·0)	39·0 (33·7 to 47·9)	43·4 (37·2 to 51·9)	21·2 (12·9 to 29·8)*	5·2 (-0·9 to 12·3)	16·0 (9·3 to 22·8)*	2·60 (1·51 to 3·68)*	2·12 (-0·33 to 4·94)	2·89 (1·61 to 4·06)*
Vietnam	36·6 (33·1 to 40·4)	40·5 (37·2 to 44·5)	44·7 (41·6 to 48·2)	50·1 (46·5 to 53·7)	54·7 (50·8 to 58·7)	60·3 (56·3 to 64·1)	23·7 (18·1 to 29·0)*	8·1 (4·2 to 12·1)*	15·6 (10·8 to 20·3)*	1·92 (1·46 to 2·40)*	2·01 (1·01 to 3·05)*	1·87 (1·28 to 2·47)*
Central Europe, Eastern Europe, and Central Asia	57·1 (55·8 to 58·6)	57·3 (56·1 to 58·7)	59·5 (58·1 to 60·8)	62·7 (61·1 to 64·1)	67·9 (66·3 to 69·2)	71·4 (68·1 to 74·3)	14·3 (10·9 to 17·4)*	2·5 (0·6 to 4·2)*	11·8 (8·4 to 14·9)*	0·86 (0·66 to 1·03)*	0·43 (0·10 to 0·73)*	1·13 (0·82 to 1·41)*
Central Asia	48·4 (47·0 to 49·9)	48·5 (47·1 to 49·9)	49·6 (48·2 to 51·0)	52·0 (50·6 to 53·5)	56·3 (54·8 to 57·7)	60·2 (58·2 to 62·4)	11·8 (9·5 to 14·1)*	1·2 (-0·5 to 2·8)	10·6 (8·3 to 12·9)*	0·84 (0·68 to 1·00)*	0·25 (-0·10 to 0·58)	1·21 (0·96 to 1·47)*
Armenia	55·7 (53·6 to 58·0)	55·4 (53·5 to 57·6)	58·9 (57·2 to 61·0)	62·0 (60·1 to 64·1)	65·8 (63·9 to 67·9)	70·7 (67·8 to 73·5)	15·0 (11·9 to 18·0)*	3·2 (1·1 to 5·3)*	11·7 (8·9 to 14·8)*	0·92 (0·74 to 1·10)*	0·56 (0·18 to 0·94)*	1·14 (0·87 to 1·42)*
Azerbaijan	49·6 (47·0 to 52·1)	50·0 (47·6 to 52·4)	51·9 (49·4 to 54·4)	52·6 (49·7 to 55·4)	59·4 (56·6 to 62·2)	65·6 (61·2 to 69·6)	16·1 (11·1 to 20·6)*	2·3 (-1·1 to 5·6)	13·8 (9·2 to 18·4)*	1·08 (0·76 to 1·37)*	0·46 (-0·21 to 1·10)	1·47 (1·00 to 1·93)*
Georgia	61·2 (59·0 to 63·5)	62·8 (60·8 to 64·6)	63·4 (60·8 to 65·4)	63·5 (61·6 to 65·4)	64·4 (62·3 to 66·5)	67·1 (62·7 to 71·0)	5·9 (1·1 to 10·7)*	2·1 (-0·7 to 4·7)	3·7 (-0·8 to 7·9)	0·35 (0·07 to 0·63)*	0·34 (-0·11 to 0·76)	0·36 (-0·08 to 0·76)
Kazakhstan	55·5 (53·1 to 57·6)	52·8 (50·1 to 55·2)	54·1 (51·4 to 56·5)	56·4 (54·0 to 59·1)	62·2 (59·6 to 64·6)	69·1 (64·7 to 73·2)	13·6 (9·3 to 18·0)*	-1·4 (-4·3 to 1·5)	15·0 (10·2 to 19·6)*	0·84 (0·58 to 1·10)*	-0·25 (-0·80 to 0·27)	1·53 (1·05 to 1·97)*
Kyrgyzstan	50·9 (49·5 to 53·1)	49·9 (48·5 to 51·8)	52·6 (51·3 to 54·2)	54·3 (52·9 to 55·7)	57·0 (55·7 to 58·4)	60·6 (58·3 to 62·8)	9·7 (6·7 to 12·4)*	1·8 (0·1 to 3·3)*	8·0 (5·2 to 10·3)*	0·67 (0·46 to 0·85)*	0·34 (0·02 to 0·63)*	0·88 (0·57 to 1·13)*
Mongolia	36·6 (34·0 to 39·3)	36·4 (34·0 to 39·3)	38·7 (36·1 to 41·5)	47·2 (44·7 to 49·9)	52·3 (49·0 to 55·4)	53·4 (49·1 to 57·6)	16·8 (11·3 to 21·9)*	2·2 (-1·3 to 5·6)	14·6 (9·5 to 19·7)*	1·45 (0·98 to 1·86)*	0·58 (-0·35 to 1·46)	2·00 (1·32 to 2·65)*

Supplementary table 3. **Global, regional, national or territory, and select subnational estimates on the HAQ Index for 1990–2016, and absolute change and annualised rates of change for 1990–2016, 1990–2000, and 2000–2016.** Starred estimates of change represent statistically significant changes during a given time period. HAQ Index=Healthcare Access and Quality Index.

	HAQ Index						Absolute change			Annualised rate of change		
	1990	1995	2000	2005	2010	2016	1990-2016	1990-2000	2000-2016	1990-2016	1990-2000	2000-2016
Tajikistan	41·3 (38·7 to 44·2)	42·5 (39·7 to 45·4)	42·6 (39·9 to 45·5)	45·5 (42·6 to 48·3)	48·5 (45·3 to 51·8)	51·7 (47·7 to 55·5)	10·4 (5·7 to 15·3)*	1·3 (-2·7 to 5·1)	9·1 (4·4 to 13·8)*	0·86 (0·48 to 1·25)*	0·30 (-0·64 to 1·24)	1·21 (0·60 to 1·82)*
Turkmenistan	45·4 (43·8 to 46·9)	46·8 (45·4 to 48·3)	49·1 (47·1 to 51·0)	51·2 (48·8 to 53·8)	56·0 (53·3 to 58·9)	61·6 (58·7 to 64·8)	16·2 (13·0 to 20·2)*	3·6 (1·3 to 6·1)*	12·6 (9·8 to 15·3)*	1·17 (0·96 to 1·44)*	0·77 (0·27 to 1·29)*	1·43 (1·12 to 1·71)*
Uzbekistan	50·3 (48·4 to 52·2)	50·8 (49·1 to 52·5)	52·8 (51·0 to 54·6)	55·5 (53·2 to 57·6)	59·5 (57·0 to 61·6)	62·9 (59·3 to 66·0)	12·6 (8·6 to 16·1)*	2·5 (0·2 to 4·8)*	10·1 (6·2 to 13·2)*	0·86 (0·60 to 1·09)*	0·49 (0·04 to 0·92)*	1·09 (0·69 to 1·42)*
Central Europe	58·8 (57·7 to 60·2)	63·7 (62·7 to 64·9)	68·9 (67·6 to 69·9)	73·1 (71·8 to 74·1)	77·3 (76·0 to 78·2)	80·6 (79·2 to 81·7)	21·8 (19·6 to 23·2)*	10·1 (8·3 to 11·3)*	11·7 (10·5 to 12·9)*	1·21 (1·09 to 1·30)*	1·58 (1·30 to 1·79)*	0·98 (0·88 to 1·09)*
Albania	54·8 (52·7 to 56·9)	60·5 (58·6 to 62·4)	63·6 (61·5 to 65·7)	67·8 (65·8 to 69·6)	72·3 (70·1 to 74·4)	75·4 (72·5 to 78·2)	20·6 (17·2 to 24·0)*	8·8 (6·1 to 11·7)*	11·8 (8·4 to 15·0)*	1·23 (1·03 to 1·42)*	1·49 (1·03 to 1·96)*	1·06 (0·77 to 1·35)*
Bosnia and Herzegovina	52·3 (49·4 to 55·2)	53·0 (50·0 to 56·0)	61·3 (58·1 to 64·4)	65·8 (62·2 to 69·0)	69·6 (65·3 to 72·9)	72·2 (67·2 to 76·4)	19·9 (14·8 to 24·6)*	9·0 (5·8 to 12·3)*	10·9 (5·9 to 16·1)*	1·24 (0·94 to 1·52)*	1·59 (1·01 to 2·18)*	1·02 (0·56 to 1·51)*
Bulgaria	65·1 (64·0 to 66·4)	66·0 (64·9 to 67·0)	68·0 (66·5 to 69·0)	70·6 (69·1 to 71·7)	73·5 (72·1 to 74·6)	77·2 (73·3 to 80·7)	12·1 (8·4 to 15·8)*	2·9 (1·2 to 4·2)*	9·2 (5·4 to 12·8)*	0·65 (0·46 to 0·84)*	0·43 (0·18 to 0·63)*	0·79 (0·48 to 1·08)*
Croatia	73·9 (71·9 to 76·2)	74·6 (72·8 to 76·5)	78·1 (76·5 to 79·7)	81·8 (80·1 to 83·4)	84·6 (83·0 to 86·1)	86·9 (84·5 to 89·4)	13·0 (9·7 to 16·4)*	4·2 (1·6 to 6·7)*	8·8 (5·8 to 11·8)*	0·63 (0·46 to 0·79)*	0·55 (0·21 to 0·90)*	0·67 (0·45 to 0·89)*
Czech Republic	72·2 (70·9 to 73·4)	77·5 (76·3 to 78·5)	81·4 (79·8 to 82·4)	83·9 (82·7 to 84·8)	86·4 (85·3 to 87·3)	89·0 (87·5 to 90·4)	16·8 (14·9 to 18·7)*	9·2 (7·4 to 10·4)*	7·6 (6·0 to 9·5)*	0·80 (0·72 to 0·89)*	1·20 (0·96 to 1·35)*	0·56 (0·44 to 0·70)*
Hungary	66·4 (64·8 to 68·6)	70·3 (68·7 to 72·1)	74·5 (73·0 to 76·0)	77·9 (76·5 to 79·5)	80·3 (78·7 to 82·3)	82·1 (79·5 to 84·9)	15·7 (12·6 to 18·7)*	8·0 (6·0 to 9·9)*	7·6 (4·7 to 10·7)*	0·81 (0·66 to 0·96)*	1·14 (0·83 to 1·41)*	0·61 (0·38 to 0·84)*
Macedonia	59·3 (57·2 to 61·6)	61·5 (59·7 to 63·6)	65·3 (63·6 to 67·4)	68·6 (66·9 to 70·6)	73·1 (71·2 to 75·1)	75·1 (72·6 to 77·5)	15·7 (12·3 to 18·9)*	6·0 (3·4 to 8·4)*	9·7 (6·7 to 12·6)*	0·90 (0·71 to 1·09)*	0·96 (0·54 to 1·36)*	0·87 (0·61 to 1·11)*
Montenegro	69·1 (66·5 to 71·7)	69·8 (67·7 to 71·8)	70·3 (68·4 to 72·4)	74·3 (72·4 to 76·5)	78·1 (76·1 to 80·2)	81·0 (78·6 to 83·5)	11·9 (8·3 to 15·5)*	1·1 (-1·8 to 3·9)	10·8 (7·8 to 13·9)*	0·61 (0·42 to 0·80)*	0·16 (-0·26 to 0·57)	0·89 (0·64 to 1·14)*
Poland	61·0 (59·8 to 62·4)	64·7 (63·5 to 65·9)	70·8 (69·1 to 72·0)	74·5 (72·5 to 75·7)	78·7 (76·8 to 80·0)	82·4 (79·7 to 84·6)	21·4 (18·2 to 23·8)*	9·8 (7·6 to 11·4)*	11·6 (9·3 to 14·0)*	1·16 (0·99 to 1·28)*	1·49 (1·16 to 1·73)*	0·95 (0·77 to 1·13)*
Romania	59·1 (57·6 to 61·0)	62·1 (60·6 to 63·7)	66·8 (65·2 to 68·4)	70·6 (68·9 to 72·1)	74·3 (72·6 to 75·8)	78·3 (75·9 to 80·7)	19·2 (16·3 to 21·9)*	7·7 (5·3 to 9·5)*	11·5 (8·9 to 14·2)*	1·08 (0·91 to 1·22)*	1·22 (0·83 to 1·51)*	0·99 (0·78 to 1·21)*
Serbia	64·7 (61·9 to 67·5)	64·3 (62·2 to 66·5)	66·9 (64·9 to 69·2)	70·2 (68·1 to 72·2)	74·1 (72·2 to 76·0)	77·2 (74·9 to 79·3)	12·5 (9·3 to 15·6)*	2·2 (-0·7 to 5·2)	10·3 (7·4 to 13·0)*	0·68 (0·51 to 0·86)*	0·33 (-0·11 to 0·80)	0·90 (0·64 to 1·13)*
Slovakia	67·8 (65·8 to 69·4)	71·5 (69·4 to 73·3)	73·6 (71·6 to 75·4)	75·8 (74·3 to 77·4)	79·7 (78·1 to 81·2)	83·3 (80·4 to 86·3)	15·5 (12·3 to 18·9)*	5·9 (3·6 to 8·1)*	9·7 (6·6 to 12·8)*	0·79 (0·64 to 0·95)*	0·83 (0·51 to 1·15)*	0·77 (0·53 to 1·02)*
Slovenia	74·1 (72·2 to 76·1)	76·3 (74·3 to 78·2)	79·5 (77·8 to 81·3)	83·5 (81·6 to 85·4)	87·6 (85·9 to 89·4)	90·8 (88·2 to 93·4)	16·6 (13·5 to 19·8)*	5·3 (3·0 to 7·9)*	11·3 (8·0 to 14·6)*	0·78 (0·63 to 0·92)*	0·70 (0·39 to 1·03)*	0·83 (0·59 to 1·06)*
Eastern Europe	63·5 (61·7 to 65·3)	61·2 (59·5 to 62·8)	63·1 (61·1 to 64·8)	66·3 (64·3 to 68·0)	71·9 (70·1 to 73·6)	75·0 (69·6 to 80·2)	11·5 (5·7 to 16·5)*	-0·4 (-3·0 to 1·9)	11·9 (6·4 to 17·1)*	0·64 (0·33 to 0·90)*	-0·07 (-0·48 to 0·29)	1·08 (0·60 to 1·51)*
Belarus	64·8 (63·4 to 66·3)	64·5 (63·0 to 66·0)	66·1 (63·7 to 67·6)	68·8 (66·8 to 70·2)	73·2 (71·7 to 74·6)	79·0 (75·3 to 82·8)	14·3 (10·5 to 18·1)*	1·3 (-1·6 to 3·1)	13·0 (9·1 to 16·9)*	0·76 (0·58 to 0·96)*	0·20 (-0·25 to 0·48)	1·12 (0·79 to 1·46)*
Estonia	68·2 (66·8 to 69·8)	66·7 (65·3 to 68·1)	71·6 (70·2 to 72·8)	77·0 (75·8 to 78·2)	82·3 (81·0 to 83·6)	85·9 (83·6 to 88·3)	17·7 (15·1 to 20·6)*	3·4 (1·7 to 5·0)*	14·3 (11·8 to 17·0)*	0·89 (0·76 to 1·03)*	0·48 (0·25 to 0·72)*	1·14 (0·94 to 1·35)*
Latvia	67·3 (65·9 to 68·8)	64·3 (62·8 to 66·0)	69·6 (68·1 to 71·0)	73·1 (71·8 to 74·3)	77·2 (75·9 to 78·5)	80·7 (78·0 to 83·3)	13·4 (10·5 to 16·4)*	2·3 (0·4 to 4·1)*	11·1 (8·3 to 14·2)*	0·70 (0·55 to 0·84)*	0·33 (0·06 to 0·61)*	0·93 (0·70 to 1·17)*
Lithuania	69·3 (68·0 to 70·6)	67·5 (66·2 to 68·8)	72·1 (70·6 to 73·4)	73·6 (72·3 to 74·7)	77·0 (75·8 to 78·3)	80·5 (78·7 to 82·3)	11·2 (9·2 to 13·2)*	2·9 (1·2 to 4·4)*	8·3 (6·0 to 10·7)*	0·58 (0·47 to 0·68)*	0·40 (0·17 to 0·62)*	0·68 (0·49 to 0·87)*
Moldova	56·6 (54·4 to 59·0)	54·6 (52·4 to 56·8)	58·1 (56·0 to 60·2)	60·7 (58·4 to 62·9)	62·8 (60·6 to 64·9)	67·4 (64·5 to 70·4)	10·8 (7·3 to 14·0)*	1·5 (-1·5 to 4·3)	9·3 (6·2 to 12·6)*	0·67 (0·46 to 0·86)*	0·26 (-0·25 to 0·76)	0·93 (0·62 to 1·24)*
Russia	63·1 (60·6 to 65·4)	60·5 (58·4 to 62·7)	62·5 (60·1 to 64·7)	66·2 (63·6 to 68·6)	72·1 (69·7 to 74·3)	75·1 (67·7 to 81·7)	11·9 (4·5 to 19·0)*	-0·6 (-3·8 to 2·5)	12·6 (5·0 to 19·4)*	0·66 (0·26 to 1·01)*	-0·10 (-0·63 to 0·40)	1·14 (0·48 to 1·73)*
Ukraine	64·9 (63·3 to 66·5)	62·6 (60·8 to 64·3)	64·0 (61·8 to 65·8)	66·1 (64·1 to 67·8)	71·7 (69·8 to 73·4)	74·6 (68·3 to 79·8)	9·6 (3·3 to 15·2)*	-1·0 (-3·6 to 1·2)	10·6 (4·2 to 16·5)*	0·53 (0·19 to 0·81)*	-0·15 (-0·56 to 0·18)	0·95 (0·39 to 1·45)*
High-income	75·5 (74·4 to 76·6)	80·9 (80·0 to 81·6)	83·2 (82·3 to 83·8)	86·2 (85·6 to 86·7)	88·3 (87·8 to 88·8)	89·8 (89·2 to 90·4)	14·4 (13·3 to 15·5)*	7·7 (6·7 to 8·8)*	6·6 (6·0 to 7·4)*	0·67 (0·62 to 0·73)*	0·98 (0·84 to 1·11)*	0·48 (0·43 to 0·54)*
Australasia	83·2 (82·4 to 84·0)	86·6 (85·8 to 87·3)	89·7 (89·0 to 90·5)	92·0 (91·2 to 92·7)	93·6 (92·8 to 94·2)	95·5 (94·5 to 96·4)	12·3 (11·2 to 13·3)*	6·5 (5·8 to 7·3)*	5·8 (4·8 to 6·8)*	0·53 (0·48 to 0·57)*	0·76 (0·67 to 0·85)*	0·39 (0·32 to 0·46)*
Australia	83·9 (83·0 to 84·7)	87·2 (86·3 to 88·0)	90·4 (89·6 to 91·2)	92·7 (91·8 to 93·5)	94·3 (93·5 to 95·0)	95·9 (94·8 to 96·8)	12·0 (10·9 to 13·1)*	6·5 (5·6 to 7·5)*	5·5 (4·4 to 6·6)*	0·51 (0·47 to 0·56)*	0·75 (0·65 to 0·86)*	0·37 (0·30 to 0·44)*
New Zealand	80·2 (79·2 to 81·4)	84·2 (83·4 to 85·0)	87·0 (86·0 to 87·8)	89·2 (88·3 to 90·1)	89·9 (89·0 to 90·7)	92·4 (90·3 to 94·3)	12·2 (9·8 to 14·3)*	6·8 (5·4 to 7·9)*	5·4 (3·1 to 7·4)*	0·54 (0·44 to 0·64)*	0·81 (0·64 to 0·95)*	0·38 (0·22 to 0·51)*
High-income Asia Pacific	73·7 (72·1 to 75·6)	80·1 (78·7 to 81·6)	81·8 (80·6 to 83·1)	87·9 (86·8 to 88·9)	91·0 (90·2 to 91·8)	93·2 (91·8 to 94·2)	19·5 (16·9 to 21·5)*	8·1 (5·9 to 10·0)*	11·4 (9·7 to 13·0)*	0·90 (0·78 to 1·00)*	1·04 (0·75 to 1·30)*	0·81 (0·69 to 0·93)*
Brunei	62·9 (60·0 to 65·6)	66·1 (63·1 to 68·8)	70·0 (67·5 to 72·7)	73·3 (70·9 to 76·0)	75·4 (73·0 to 77·7)	76·4 (71·9 to 81·0)	13·5 (8·4 to 18·7)*	7·1 (3·9 to 10·6)*	6·4 (1·4 to 11·3)*	0·75 (0·48 to 1·02)*	1·07 (0·60 to 1·60)*	0·55 (0·12 to 0·94)*
Japan	80·9 (80·3 to 81·7)	83·9 (83·4 to 84·5)	86·9 (86·3 to 87·5)	90·2 (89·7 to 90·8)	92·3 (91·8 to 92·8)	94·1 (93·5 to 94·6)	13·3 (12·2 to 13·9)*	6·1 (5·4 to 6·4)*	7·2 (6·6 to 7·8)*	0·58 (0·54 to 0·62)*	0·72 (0·65 to 0·77)*	0·50 (0·45 to 0·54)*
Aichi	80·9 (79·9 to 82·0)	83·6 (82·6 to 84·6)	86·4 (85·4 to 87·3)	89·5 (88·6 to 90·4)	91·6 (90·7 to 92·5)	93·5 (92·1 to 94·8)	12·6 (10·9 to 14·2)*	5·5 (4·3 to 6·5)*	7·2 (5·5 to 8·7)*	0·56 (0·48 to 0·63)*	0·65 (0·52 to 0·78)*	0·50 (0·38 to 0·60)*
Akita	80·1 (78·9 to 81·3)	83·4 (82·3 to 84·4)	86·4 (85·2 to 87·6)	88·7 (87·4 to 89·9)	90·8 (89·6 to 91·9)	92·8 (91·2 to 94·2)	12·7 (10·7 to 14·6)*	6·3 (4·9 to 7·6)*	6·4 (4·7 to 8·0)*	0·57 (0·48 to 0·65)*	0·76 (0·59 to 0·92)*	0·45 (0·33 to 0·56)*
Aomori	77·6 (76·4 to 78·9)	80·4 (79·2 to 81·6)	83·0 (81·6 to 84·2)	86·7 (85·5 to 87·9)	89·4 (88·1 to 90·4)	91·7 (89·9 to 93·3)	14·1 (12·0 to 16·0)*	5·4 (4·1 to 6·8)*	8·7 (6·7 to 10·8)*	0·64 (0·55 to 0·73)*	0·67 (0·50 to 0·84)*	0·62 (0·48 to 0·77)*
Chiba	81·2 (80·1 to 82·4)	83·8 (82·7 to 85·0)	86·6 (85·5 to 87·7)	89·9 (88·9 to 90·9)	92·2 (91·3 to 93·2)	93·9 (92·6 to 95·0)	12·7 (10·9 to 14·3)*	5·4 (4·3 to 6·4)*	7·3 (5·8 to 8·7)*	0·56 (0·48 to 0·63)*	0·64 (0·51 to 0·77)*	0·51 (0·40 to 0·61)*
Ehime	80·8 (79·5 to 82·0)	83·3 (82·1 to 84·5)	86·6 (85·3 to 87·9)	89·1 (87·9 to 90·2)	91·4 (90·2 to 92·4)	92·7 (89·9 to 95·1)	11·9 (8·7 to 14·4)*	5·8 (4·4 to 7·2)*	6·2 (3·1 to 8·7)*	0·53 (0·39 to 0·64)*	0·69 (0·52 to 0·86)*	0·43 (0·22 to 0·60)*
Fukui	82·0 (80·9 to 83·0)	84·2 (83·2 to 85·3)	87·6 (86·5 to 88·7)	91·0 (89·9 to 92·0)	93·1 (92·1 to 93·9)	94·5 (93·1 to 95·6)	12·5 (10·8 to 14·0)*	5·5 (4·2 to 6·9)*	6·9 (5·3 to 8·4)*	0·54 (0·47 to 0·61)*	0·65 (0·49 to 0·81)*	0·47 (0·37 to 0·58)*
Fukuoka	80·0 (79·0 to 81·2)	83·0 (82·0 to 84·1)	86·0 (85·0 to 87·1)	89·9 (88·9 to 90·9)	92·1 (91·1 to 93·0)	94·2 (92·9 to 95·4)	14·2 (12·5 to 15·7)*	6·0 (4·9 to 7·1)*	8·2 (6·6 to 9·7)*	0·63 (0·55 to 0·69)*	0·72 (0·59 to 0·86)*	0·57 (0·46 to 0·67)*
Fukushima	79·1 (77·9 to 80·2)	82·4 (81·3 to 83·6)	85·4 (84·1 to 86·6)	89·2 (87·9 to 90·2)	91·2 (90·1 to 92·1)	93·2 (91·1 to 95·1)	14·1 (11·6 to 16·3)*	6·3 (5·0 to 7·6)*	7·8 (5·4 to 10·2)*	0·63 (0·52 to 0·73)*	0·76 (0·61 to 0·93)*	0·55 (0·38 to 0·71)*
Gifu	81·9 (80·8 to 83·0)	84·5 (83·5 to 85·7)	87·0 (85·9 to 88·1)	89·8 (88·6 to 90·9)	92·2 (91·1 to 93·3)	93·4 (90·8 to 95·4)	11·5 (8·9 to 14·0)*	5·1 (3·8 to 6·4)*	6·4 (3·7 to 8·7)*	0·51 (0·39 to 0·61)*	0·61 (0·46 to 0·76)*	0·44 (0·26 to 0·60)*
Gunma	80·5 (79·4 to 81·7)	83·6 (82·5 to 84·6)	86·6 (85·4 to 87·7)	90·0 (89·0 to 91·1)	92·1 (91·1 to 93·0)	93·8 (92·5 to 95·0)	13·3 (11·7 to 14·9)*	6·1 (4·8 to 7·5)*	7·2 (5·5 to 8·8)*	0·59 (0·51 to 0·66)*	0·73 (0·58 to 0·89)*	0·50 (0·38 to 0·61)*
Hiroshima	81·5 (80·5 to 82·5)	84·6 (83·6 to 85·5)	87·7 (86·8 to 88·6)	90·6 (89·6 to 91·5)	92·6 (91·7 to 93·4)	94·4 (93·2 to 95·4)	12·9 (11·3 to 14·3)*	6·2 (5·1 to 7·2)*	6·7 (5·3 to 8·0)*	0·56 (0·49 to 0·63)*	0·73 (0·60 to 0·85)*	0·46 (0·37 to 0·55)*
Hokkaido	79·9 (78·9 to 81·0)	83·0 (81·9 to 84·1)	86·1 (85·1 to 87·1)	89·1 (88·0 to 90·1)	91·3 (90·4 to 92·2)	93·1 (90·3 to 95·2)	13·2 (10·3 to 15·5)*	6·2 (5·0 to 7·2)*	7·0 (4·0 to 9·2)*	0·59 (0·46 to 0·68)*	0·74 (0·60 to 0·87)*	0·49 (0·28 to 0·64)*
Hyogo	79·9 (79·0 to 81·0)	83·0 (81·9 to 84·1)	86·1 (85·1 to 87·1)	89·5 (88·5 to 90·4)	92·0 (91·1 to 92·9)	93·8 (92·3 to 95·1)	13·9 (12·1 to 15·6)*	6·2 (5·1 to 7·4)*	7·7 (6·2 to 9·1)*	0·62 (0·54 to 0·69)*	0·75 (0·61 to 0·88)*	0·53 (0·43 to 0·64)*
Ibaraki	80·1 (79·1 to 81·3)	83·4 (82·3 to 84·6)	86·4 (85·3 to 87·6)	90·0 (89·0 to 91·0)	92·0 (91·0 to 92·7)	93·7 (92·5 to 94·8)	13·6 (11·9 to 15·2)*	6·3 (5·1 to 7·5)*	7·3 (5·8 to 8·9)*	0·60 (0·53 to 0·67)*	0·76 (0·61 to 0·90)*	0·51 (0·40 to 0·61)*
Ishikawa	82·3 (81·4 to 83·3)	84·9 (83·9 to 85·9)	88·0 (86·9 to 89·1)	90·7 (89·6 to 91·8)	92·9 (92·0 to 93·8)	94·7 (93·3 to 95·8)	12·3 (10·7 to 13·8)*	5·7 (4·4 to 7·0)*	6·6 (5·1 to 8·1)*	0·54 (0·46 to 0·60)*	0·67 (0·52 to 0·82)*	0·45 (0·35 to 0·55)*
Iwate	80·5 (79·5 to 81·5)	83·2 (82·2 to 84·2)	86·4 (85·2 to 87·4)	88·7 (87·5 to 89·7)	90·4 (89·3 to 91·5)	92·9 (91·3 to 94·3)	12·4 (10·6 to 14·1)*	5·9 (4·7 to 7·1)*	6·5 (4·8 to 8·0)*	0·55 (0·47 to 0·62)*	0·71 (0·56 to 0·85)*	0·45 (0·34 to 0·56)*
Kagawa	81·9 (80·9 to 82·9)	84·8 (83·7 to 85·8)	87·4 (86·3 to 88·5)	90·0 (89·0 to 91·0)	91·4 (90·4 to 92·4)	93·2 (91·6 to 94·5)	11·3 (9·5 to 12·9)*	5·5 (4·3 to 6·7)*	5·8 (4·2 to 7·4)*	0·50 (0·42 to 0·57)*	0·65 (0·51 to 0·79)*	0·40 (0·29 to 0·51)*
Kagoshima	78·6 (77·4 to 79·8)	81·5 (80·2 to 82·7)	84·5 (83·2 to 85·8)	87·4 (86·1 to 88·6)	89·3 (88·1 to 90·4)	91·1 (87·3 to 93·7)	12·4 (8·8 to 15·1)*	5·9 (4·5 to 7·4)*	6·6 (2·7 to 9·4)*	0·56 (0·41 to 0·68)*	0·72 (0·54 to 0·90)*	0·47 (0·20 to 0·66)*
Kanagawa	81·8 (80·8 to 82·8)	84·3 (83·4 to 85·3)	87·4 (86·4 to 88·4)	90·8 (89·9 to 91·9)	93·0 (92·0 to 93·8)	94·6 (93·4 to 95·7)	12·9 (11·2 to 14·2)*	5·6 (4·6 to 6·7)*	7·3 (5·9 to 8·6)*	0·56 (0·49 to 0·62)*	0·66 (0·54 to 0·78)*	0·50 (0·40 to 0·59)*
Kochi	79·3 (78·2 to 80·4)	82·1 (81·0 to 83·2)	84·7 (83·6 to 85·9)	87·3 (86·1 to 88·6)	89·4 (88·3 to 90·6)	91·8 (89·8 to 93·4)	12·4 (10·3 to 14·2)*	5·4 (4·2 to 6·6)*	7·0 (5·1 to 8·8)*	0·56 (0·47 to 0·64)*	0·66 (0·52 to 0·80)*	0·50 (0·36 to 0·62)*
Kumamoto	80·4 (79·3 to 81·5)	83·6 (82·6 to 84·7)	86·7 (85·7 to 87·7)	89·4 (88·4 to 90·5)	91·3 (90·3 to 92·4)	93·6 (91·8 to 95·1)	13·2 (11·2 to 15·0)*	6·3 (5·0 to 7·5)*	7·0 (5·0 to 8·6)*	0·59 (0·50 to 0·66)*	0·75 (0·60 to 0·90)*	0·48 (0·35 to 0·60)*
Kyoto	80·5 (79·5 to 81·7)	83·7 (82·7 to 84·7)	86·9 (85·7 to 87·9)	90·7 (89·7 to 91·8)	93·0 (92·0 to 93·9)	94·5 (93·1 to 95·7)	13·9 (12·1 to 15·4)*	6·3 (5·2 to 7·5)*	7·6 (6·0 to 9·1)*	0·61 (0·54 to 0·68)*	0·76 (0·62 to 0·90)*	0·52 (0·42 to 0·63)*
Mie	81·7 (80·6 to 82·8)	84·6 (83·7 to 85·7)	87·4 (86·4 to 88·4)	90·4 (89·4 to 91·5)	92·4 (91·5 to 93·3)	93·9 (92·6 to 95·0)	12·2 (10·6 to 13·6)*	5·7 (4·5 to 6·8)*	6·5 (5·1 to 7·9)*	0·53 (0·47 to 0·60)*	0·67 (0·53 to 0·80)*	0·45 (0·35 to 0·55)*
Miyagi	81·5 (80·5 to 82·6)	84·6 (83·7 to 85·6)	87·4 (86·5 to 88·4)	90·0 (89·0 to 90·9)	92·5 (91·5 to 93·3)	94·6 (93·2 to 95·7)	13·0 (11·1 to 14·5)*	5·9 (4·7 to 7·1)*	7·1 (5·6 to 8·5)*	0·57 (0·49 to 0·64)*	0·70 (0·55 to 0·84)*	0·49 (0·38 to 0·59)*
Miyazaki	79·5 (78·4 to 80·7)	82·8 (81·8 to 83·8)	86·0 (84·9 to 87·1)	89·0 (87·8 to 90·1)	90·5 (89·4 to 91·6)	93·0 (91·2 to 94·4)	13·4 (11·3 to 15·3)*	6·5 (5·2 to 7·7)*	7·0 (5·1 to 8·6)*	0·60 (0·51 to 0·68)*	0·78 (0·63 to 0·94)*	0·49 (0·36 to 0·60)*
Nagano	81·6 (80·4 to 82·7)	85·1 (84·0 to 86·2)	88·1 (86·9 to 89·2)	91·4 (90·3 to 92·5)	93·6 (92·5 to 94·5)	95·1 (92·6 to 96·8)	13·5 (10·6 to 15·6)*	6·5 (5·1 to 7·8)*	7·0 (4·5 to 9·1)*	0·59 (0·47 to 0·68)*	0·77 (0·60 to 0·93)*	0·48 (0·31 to 0·62)*
Nagasaki	78·9 (77·8 to 80·1)	81·9 (80·9 to 82·9)	85·3 (84·3 to 86·3)	88·0 (87·0 to 89·1)	90·1 (89·1 to 91·2)	92·8 (91·0 to 94·3)	13·8 (11·8 to 15·8)*	6·4 (5·2 to 7·6)*	7·4 (5·6 to 9·2)*	0·62 (0·53 to 0·71)*	0·78 (0·63 to 0·93)*	0·52 (0·39 to 0·65)*
Nara	81·0 (80·0 to 82·1)	83·2 (82·1 to 84·2)	86·7 (85·6 to 87·8)	89·2 (88·2 to 90·3)	91·3 (90·2 to 92·2)	93·0 (91·3 to 94·4)	12·0 (10·1 to 13·6)*	5·7 (4·5 to 6·9)*	6·3 (4·5 to 7·8)*	0·53 (0·45 to 0·60)*	0·68 (0·53 to 0·82)*	0·43 (0·31 to 0·54)*
Niigata	82·5 (81·4 to 83·7)	85·0 (83·9 to 86·2)	87·9 (86·6 to 89·1)	91·0 (89·7 to 92·0)	93·1 (92·0 to 94·0)	94·4 (92·0 to 96·3)	11·9 (9·3 to 14·2)*	5·3 (3·8 to 6·8)*	6·6 (4·2 to 8·7)*	0·52 (0·41 to 0·61)*	0·62 (0·45 to 0·79)*	0·45 (0·29 to 0·60)*
Oita	80·9 (79·5 to 82·1)	84·0 (82·9 to 85·3)	87·3 (86·0 to 88·4)	89·8 (88·6 to 91·0)	91·9 (90·7 to 93·0)	93·5 (90·8 to 95·5)	12·6 (9·7 to 15·1)*	6·4 (4·9 to 7·9)*	6·2 (3·3 to 8·5)*	0·56 (0·43 to 0·66)*	0·76 (0·58 to 0·94)*	0·43 (0·23 to 0·59)*
Okayama	81·2 (80·1 to 82·3)	84·6 (83·6 to 85·6)	87·4 (86·4 to 88·5)	90·3 (89·4 to 91·4)	92·3 (91·4 to 93·2)	93·6 (91·9 to 94·8)	12·4 (10·5 to 13·9)*	6·2 (4·9 to 7·4)*	6·1 (4·4 to 7·7)*	0·55 (0·47 to 0·61)*	0·74 (0·58 to 0·88)*	0·42 (0·30 to 0·53)*
Okinawa	76·0 (74·8 to 77·3)	79·0 (77·8 to 80·2)	81·6 (80·2 to 82·9)	85·7 (84·5 to 87·0)	87·1 (86·0 to 88·3)	90·3 (88·5 to 91·9)	14·3 (12·1 to 16·3)*	5·6 (4·2 to 6·9)*	8·7 (6·8 to 10·8)*	0·66 (0·56 to 0·76)*	0·71 (0·53 to 0·87)*	0·64 (0·50 to 0·78)*
Osaka	78·9 (77·9 to 80·1)	82·3 (81·3 to 83·4)	85·2 (84·1 to 86·3)	89·0 (87·9 to 90·0)	91·4 (90·5 to 92·2)	93·4 (91·8 to 94·7)	14·4 (12·6 to 16·1)*	6·3 (5·3 to 7·4)*	8·1 (6·3 to 9·7)*	0·65 (0·56 to 0·72)*	0·77 (0·64 to 0·90)*	0·57 (0·44 to 0·68)*
Saga	80·2 (79·0 to 81·4)	82·7 (81·4 to 83·9)	85·6 (84·3 to 86·9)	89·0 (87·8 to 90·2)	91·1 (89·8 to 92·3)	92·8 (90·1 to 94·9)	12·6 (9·4 to 15·2)*	5·4 (4·0 to 7·0)*	7·2 (4·5 to 9·5)*	0·56 (0·42 to 0·67)*	0·65 (0·48 to 0·84)*	0·50 (0·32 to 0·67)*
Saitama	80·0 (79·0 to 81·1)	83·1 (82·1 to 84·1)	86·2 (85·2 to 87·3)	89·8 (88·8 to 90·8)	91·8 (90·9 to 92·7)	93·5 (92·1 to 94·9)	13·6 (11·8 to 15·0)*	6·2 (5·0 to 7·3)*	7·4 (5·9 to 8·9)*	0·60 (0·53 to 0·67)*	0·75 (0·60 to 0·89)*	0·51 (0·41 to 0·62)*
Shiga	81·4 (80·2 to 82·7)	84·9 (83·6 to 86·2)	88·2 (87·1 to 89·4)	91·4 (90·2 to 92·4)	93·7 (92·7 to 94·6)	94·7 (92·3 to 96·4)	13·3 (10·6 to 15·3)*	6·8 (5·3 to 8·3)*	6·5 (3·6 to 8·6)*	0·58 (0·47 to 0·67)*	0·80 (0·63 to 0·98)*	0·44 (0·25 to 0·58)*
Shimane	81·2 (79·9 to 82·4)	83·7 (82·4 to 85·0)	86·8 (85·4 to 88·1)	89·6 (88·1 to 91·0)	91·6 (90·2 to 92·9)	92·9 (90·0 to 95·3)	11·7 (8·6 to 14·3)*	5·7 (4·2 to 7·2)*	6·1 (3·0 to 8·6)*	0·52 (0·39 to 0·63)*	0·67 (0·49 to 0·86)*	0·42 (0·21 to 0·60)*
Shizuoka	82·5 (81·4 to 83·7)	85·5 (84·5 to 86·6)	88·7 (87·7 to 89·6)	91·2 (90·2 to 92·2)	93·2 (92·3 to 94·0)	94·7 (93·6 to 95·7)	12·2 (10·6 to 13·6)*	6·2 (5·0 to 7·2)*	6·0 (4·7 to 7·2)*	0·53 (0·46 to 0·59)*	0·72 (0·58 to 0·84)*	0·41 (0·33 to 0·49)*
Tochigi	79·7 (78·5 to 80·9)	83·2 (82·1 to 84·4)	86·1 (84·9 to 87·3)	89·6 (88·6 to 90·8)	91·9 (91·0 to 92·8)	93·8 (92·4 to 94·9)	14·1 (12·4 to 15·6)*	6·4 (5·1 to 7·6)*	7·7 (6·0 to 9·1)*	0·63 (0·55 to 0·69)*	0·77 (0·61 to 0·93)*	0·54 (0·42 to 0·64)*
Tokushima	80·3 (79·1 to 81·5)	82·9 (81·6 to 84·1)	85·6 (84·3 to 86·9)	88·1 (86·8 to 89·4)	90·3 (88·9 to 91·6)	91·2 (87·9 to 93·5)	11·0 (7·6 to 13·5)*	5·4 (3·8 to 6·9)*	5·6 (2·2 to 8·2)*	0·49 (0·34 to 0·60)*	0·65 (0·46 to 0·83)*	0·40 (0·15 to 0·57)*
Tokyo	84·1 (83·0 to 85·3)	86·9 (85·8 to 87·9)	89·7 (88·8 to 90·7)	92·7 (91·9 to 93·4)	94·0 (93·4 to 94·6)	95·1 (94·2 to 96·0)	11·1 (9·6 to 12·3)*	5·6 (4·6 to 6·7)*	5·4 (4·2 to 6·6)*	0·48 (0·41 to 0·53)*	0·65 (0·52 to 0·78)*	0·37 (0·28 to 0·45)*
Tottori	80·6 (79·3 to 81·9)	83·0 (81·7 to 84·3)	86·3 (84·9 to 87·6)	89·1 (87·9 to 90·5)	91·0 (89·6 to 92·1)	92·7 (89·6 to 95·1)	12·0 (8·6 to 14·7)*	5·6 (4·0 to 7·2)*	6·4 (3·0 to 9·0)*	0·53 (0·39 to 0·65)*	0·68 (0·48 to 0·87)*	0·45 (0·22 to 0·62)*

Supplementary table 3. **Global, regional, national or territory, and select subnational estimates on the HAQ Index for 1990–2016, and absolute change and annualised rates of change for 1990–2016, 1990–2000, and 2000–2016.** Starred estimates of change represent statistically significant changes during a given time period. HAQ Index=Healthcare Access and Quality Index.

	HAQ Index						Absolute change			Annualised rate of change		
	1990	1995	2000	2005	2010	2016	1990-2016	1990-2000	2000-2016	1990-2016	1990-2000	2000-2016
Toyama	82·5 (81·5 to 83·5)	85·5 (84·4 to 86·4)	88·5 (87·3 to 89·5)	91·3 (90·1 to 92·3)	93·1 (92·1 to 94·0)	94·7 (93·3 to 95·7)	12·2 (10·6 to 13·6)*	6·0 (4·6 to 7·2)*	6·2 (4·7 to 7·5)*	0·53 (0·46 to 0·59)*	0·70 (0·54 to 0·84)*	0·42 (0·32 to 0·51)*
Wakayama	80·8 (79·8 to 81·9)	83·6 (82·5 to 84·6)	86·3 (85·1 to 87·4)	89·0 (87·9 to 90·1)	91·2 (90·1 to 92·2)	92·9 (90·4 to 95·0)	12·1 (9·4 to 14·3)*	5·5 (4·2 to 6·8)*	6·6 (4·1 to 9·0)*	0·54 (0·42 to 0·63)*	0·66 (0·50 to 0·81)*	0·46 (0·29 to 0·62)*
Yamagata	81·0 (80·0 to 82·1)	83·2 (82·2 to 84·2)	86·1 (85·2 to 87·2)	89·5 (88·5 to 90·5)	91·6 (90·6 to 92·6)	93·3 (91·6 to 94·7)	12·2 (10·4 to 13·9)*	5·1 (3·9 to 6·3)*	7·1 (5·4 to 8·7)*	0·54 (0·46 to 0·61)*	0·61 (0·47 to 0·76)*	0·50 (0·38 to 0·61)*
Yamaguchi	81·3 (80·3 to 82·4)	83·9 (82·8 to 84·9)	87·0 (85·9 to 88·1)	89·8 (88·8 to 90·8)	91·9 (90·9 to 92·8)	93·8 (92·5 to 95·0)	12·6 (11·0 to 14·1)*	5·7 (4·5 to 6·9)*	6·9 (5·2 to 8·4)*	0·55 (0·48 to 0·62)*	0·67 (0·54 to 0·82)*	0·48 (0·36 to 0·58)*
Yamanashi	81·5 (80·4 to 82·7)	84·0 (82·8 to 85·2)	87·5 (86·1 to 88·7)	90·1 (88·9 to 91·3)	92·3 (91·0 to 93·3)	93·6 (90·7 to 95·9)	12·1 (8·8 to 14·6)*	6·0 (4·4 to 7·5)*	6·1 (2·9 to 8·7)*	0·53 (0·39 to 0·64)*	0·71 (0·53 to 0·89)*	0·42 (0·21 to 0·59)*
Singapore	69·2 (66·5 to 72·0)	74·5 (72·1 to 76·8)	79·7 (77·2 to 82·0)	84·4 (82·2 to 86·5)	88·3 (86·0 to 90·5)	90·6 (87·2 to 93·3)	21·4 (17·5 to 25·0)*	10·5 (7·1 to 13·9)*	10·9 (7·1 to 14·8)*	1·04 (0·85 to 1·21)*	1·41 (0·95 to 1·88)*	0·80 (0·53 to 1·08)*
South Korea	59·5 (56·2 to 62·9)	71·2 (68·3 to 74·3)	74·4 (71·4 to 77·0)	83·0 (80·3 to 85·5)	87·7 (85·4 to 89·8)	90·3 (85·6 to 93·9)	30·9 (24·6 to 35·7)*	14·9 (10·0 to 18·9)*	15·9 (10·9 to 20·4)*	1·61 (1·28 to 1·87)*	2·24 (1·47 to 2·86)*	1·21 (0·84 to 1·54)*
High-income North America	81·0 (80·1 to 81·7)	85·2 (84·5 to 85·8)	87·1 (86·5 to 87·7)	87·7 (87·0 to 88·3)	89·1 (88·5 to 89·6)	89·1 (88·4 to 89·8)	8·1 (7·4 to 9·0)*	6·1 (5·5 to 6·8)*	2·0 (1·5 to 2·6)*	0·37 (0·34 to 0·41)*	0·73 (0·66 to 0·81)*	0·14 (0·11 to 0·18)*
Canada	83·2 (82·2 to 84·1)	85·7 (84·6 to 86·7)	89·3 (88·4 to 90·2)	91·2 (90·3 to 92·0)	92·8 (91·9 to 93·6)	93·8 (92·8 to 94·8)	10·6 (9·3 to 11·9)*	6·1 (5·1 to 6·9)*	4·5 (3·4 to 5·7)*	0·46 (0·40 to 0·52)*	0·71 (0·59 to 0·80)*	0·31 (0·24 to 0·39)*
Greenland	54·0 (50·6 to 57·5)	57·9 (55·1 to 61·3)	59·2 (56·4 to 62·8)	59·9 (56·9 to 63·8)	63·0 (59·8 to 67·0)	67·5 (62·7 to 72·7)	13·5 (8·0 to 19·0)*	5·2 (1·6 to 8·9)*	8·3 (3·3 to 13·5)*	0·86 (0·52 to 1·19)*	0·92 (0·29 to 1·55)*	0·82 (0·33 to 1·31)*
United States	80·7 (79·8 to 81·5)	85·1 (84·4 to 85·7)	86·8 (86·1 to 87·4)	87·3 (86·7 to 87·9)	88·8 (88·2 to 89·3)	88·7 (88·0 to 89·4)	8·0 (7·2 to 8·8)*	6·1 (5·5 to 6·7)*	1·9 (1·4 to 2·5)*	0·36 (0·33 to 0·40)*	0·72 (0·65 to 0·81)*	0·13 (0·10 to 0·18)*
Alabama	76·6 (75·1 to 78·1)	80·8 (79·2 to 82·2)	81·9 (80·4 to 83·3)	82·3 (80·6 to 83·7)	83·7 (82·0 to 85·0)	83·4 (80·6 to 86·2)	6·8 (3·8 to 9·7)*	5·3 (3·6 to 6·9)*	1·5 (-1·4 to 4·6)	0·33 (0·18 to 0·46)*	0·67 (0·46 to 0·88)*	0·11 (-0·11 to 0·34)
Alaska	79·8 (78·2 to 81·4)	84·5 (83·0 to 86·0)	85·9 (84·2 to 87·4)	86·9 (85·3 to 88·3)	88·0 (86·6 to 89·4)	88·4 (86·0 to 90·8)	8·6 (5·8 to 11·4)*	6·1 (4·2 to 7·9)*	2·5 (-0·1 to 5·2)	0·40 (0·27 to 0·51)*	0·74 (0·51 to 0·95)*	0·18 (-0·01 to 0·37)
Arizona	80·7 (79·1 to 82·0)	84·4 (83·0 to 85·6)	85·5 (84·2 to 86·7)	86·0 (84·7 to 87·1)	88·7 (87·5 to 89·8)	88·0 (85·8 to 89·8)	7·3 (5·0 to 9·7)*	4·8 (3·4 to 6·3)*	2·5 (0·5 to 4·7)*	0·33 (0·23 to 0·44)*	0·58 (0·40 to 0·76)*	0·18 (0·03 to 0·33)*
Arkansas	77·3 (75·5 to 78·7)	81·3 (79·7 to 82·6)	82·4 (80·9 to 83·5)	82·6 (81·1 to 83·9)	83·9 (82·4 to 85·4)	83·8 (81·6 to 85·8)	6·5 (4·1 to 8·9)*	5·1 (3·6 to 6·7)*	1·4 (-0·8 to 3·7)	0·31 (0·20 to 0·42)*	0·64 (0·46 to 0·84)*	0·11 (-0·06 to 0·27)
California	80·5 (79·1 to 81·8)	85·3 (84·0 to 86·5)	88·2 (87·1 to 89·2)	88·4 (87·3 to 89·3)	90·7 (89·7 to 91·7)	91·0 (89·0 to 92·8)	10·5 (8·2 to 12·7)*	7·7 (6·4 to 9·2)*	2·8 (0·7 to 4·8)*	0·47 (0·37 to 0·57)*	0·92 (0·75 to 1·09)*	0·20 (0·05 to 0·33)*
Colorado	83·8 (82·4 to 85·0)	87·7 (86·6 to 88·8)	88·8 (87·6 to 89·9)	89·6 (88·5 to 90·7)	90·9 (89·9 to 91·7)	90·8 (89·3 to 92·1)	7·1 (5·3 to 8·8)*	5·0 (3·6 to 6·5)*	2·0 (0·4 to 3·6)*	0·31 (0·23 to 0·39)*	0·58 (0·42 to 0·75)*	0·14 (0·03 to 0·25)*
Connecticut	84·1 (82·8 to 85·4)	87·2 (85·9 to 88·5)	89·2 (88·0 to 90·4)	90·1 (89·0 to 91·2)	91·4 (90·6 to 92·3)	91·5 (89·8 to 93·0)	7·4 (5·3 to 9·3)*	5·0 (3·5 to 6·5)*	2·3 (0·4 to 4·0)*	0·32 (0·23 to 0·40)*	0·58 (0·41 to 0·76)*	0·16 (0·03 to 0·28)*
Delaware	80·2 (78·7 to 81·7)	85·4 (84·1 to 86·8)	85·9 (84·6 to 87·3)	87·1 (85·9 to 88·3)	88·2 (87·0 to 89·3)	88·6 (87·0 to 90·0)	8·4 (6·4 to 10·1)*	5·7 (4·1 to 7·3)*	2·6 (0·8 to 4·4)*	0·38 (0·29 to 0·46)*	0·69 (0·49 to 0·87)*	0·19 (0·05 to 0·31)*
District of Columbia	69·8 (67·1 to 72·2)	76·9 (74·0 to 79·1)	81·1 (79·2 to 83·2)	81·8 (80·0 to 84·0)	84·9 (83·6 to 86·6)	86·1 (84·0 to 88·3)	16·2 (13·6 to 19·0)*	11·3 (9·4 to 13·2)*	5·0 (2·7 to 7·0)*	0·80 (0·68 to 0·96)*	1·50 (1·24 to 1·79)*	0·37 (0·20 to 0·53)*
Florida	82·1 (80·6 to 83·4)	86·5 (85·1 to 87·7)	88·0 (86·8 to 89·1)	88·2 (87·0 to 89·4)	89·8 (88·8 to 90·7)	89·6 (87·8 to 91·1)	7·5 (5·4 to 9·4)*	5·9 (4·5 to 7·4)*	1·6 (-0·3 to 3·5)	0·34 (0·24 to 0·42)*	0·70 (0·53 to 0·88)*	0·11 (-0·02 to 0·25)
Georgia	77·2 (75·6 to 78·9)	81·9 (80·5 to 83·4)	82·9 (81·5 to 84·5)	83·9 (82·6 to 85·3)	86·1 (84·9 to 87·3)	85·5 (83·1 to 87·8)	8·3 (5·7 to 10·9)*	5·7 (4·1 to 7·4)*	2·6 (-0·0 to 5·0)	0·39 (0·27 to 0·51)*	0·71 (0·51 to 0·92)*	0·19 (-0·00 to 0·37)
Hawaii	84·2 (83·1 to 85·5)	87·8 (86·8 to 88·8)	89·2 (88·2 to 90·0)	89·3 (88·2 to 90·3)	91·0 (90·0 to 91·9)	91·1 (89·6 to 92·3)	6·8 (5·1 to 8·5)*	4·9 (3·6 to 6·1)*	1·9 (0·4 to 3·3)*	0·30 (0·22 to 0·37)*	0·57 (0·42 to 0·71)*	0·13 (0·03 to 0·23)*
Idaho	81·1 (79·5 to 82·6)	84·8 (83·2 to 86·2)	85·9 (84·4 to 87·2)	86·3 (85·0 to 87·6)	87·8 (86·3 to 89·0)	87·3 (84·6 to 89·7)	6·2 (3·3 to 8·8)*	4·8 (3·0 to 6·4)*	1·4 (-1·2 to 4·0)	0·28 (0·15 to 0·40)*	0·57 (0·36 to 0·77)*	0·10 (-0·09 to 0·28)
Illinois	79·8 (78·5 to 81·0)	84·0 (82·8 to 85·2)	86·3 (85·2 to 87·4)	87·8 (86·8 to 88·8)	89·1 (88·2 to 89·9)	89·3 (88·0 to 90·7)	9·6 (7·9 to 11·2)*	6·5 (5·1 to 7·9)*	3·0 (1·6 to 4·4)*	0·44 (0·36 to 0·51)*	0·79 (0·61 to 0·95)*	0·22 (0·11 to 0·32)*
Indiana	80·8 (79·4 to 82·2)	84·5 (83·2 to 85·8)	85·6 (84·2 to 86·8)	86·0 (84·7 to 87·3)	86·8 (85·5 to 87·9)	86·4 (83·8 to 88·7)	5·6 (2·7 to 8·3)*	4·7 (3·2 to 6·4)*	0·8 (-2·2 to 3·4)	0·26 (0·13 to 0·38)*	0·57 (0·38 to 0·78)*	0·06 (-0·16 to 0·25)
Iowa	84·6 (83·2 to 86·0)	87·7 (86·5 to 88·8)	89·1 (88·0 to 90·2)	89·5 (88·4 to 90·5)	90·0 (88·8 to 91·0)	89·9 (88·0 to 91·6)	5·3 (3·1 to 7·3)*	4·6 (3·2 to 6·1)*	0·8 (-1·2 to 2·7)	0·23 (0·14 to 0·32)*	0·52 (0·36 to 0·70)*	0·05 (-0·08 to 0·19)
Kansas	83·2 (81·7 to 84·6)	86·2 (84·8 to 87·5)	87·1 (85·6 to 88·3)	87·2 (85·8 to 88·4)	88·2 (86·7 to 89·4)	88·3 (85·7 to 90·4)	5·2 (2·5 to 7·6)*	3·9 (2·2 to 5·6)*	1·3 (-1·5 to 3·8)	0·23 (0·11 to 0·34)*	0·46 (0·26 to 0·66)*	0·09 (-0·11 to 0·27)
Kentucky	80·6 (79·2 to 81·9)	84·8 (83·5 to 85·9)	85·5 (84·3 to 86·6)	85·8 (84·6 to 86·8)	86·1 (84·8 to 87·2)	85·6 (83·6 to 87·4)	5·0 (3·0 to 7·0)*	5·0 (3·5 to 6·5)*	0·0 (-1·9 to 2·1)	0·23 (0·14 to 0·32)*	0·60 (0·43 to 0·78)*	0·00 (-0·14 to 0·15)
Louisiana	77·1 (75·7 to 78·5)	81·3 (80·0 to 82·5)	82·3 (81·0 to 83·5)	82·5 (81·2 to 83·7)	84·4 (83·0 to 85·7)	84·3 (82·4 to 86·1)	7·1 (5·1 to 9·0)*	5·2 (3·7 to 6·5)*	2·0 (-0·1 to 3·9)	0·34 (0·24 to 0·43)*	0·65 (0·47 to 0·81)*	0·15 (-0·01 to 0·29)
Maine	83·6 (82·4 to 84·8)	87·3 (86·2 to 88·4)	88·7 (87·6 to 89·9)	89·5 (88·4 to 90·6)	90·5 (89·5 to 91·4)	90·4 (88·9 to 91·7)	6·8 (4·9 to 8·5)*	5·1 (3·7 to 6·5)*	1·7 (-0·0 to 3·2)	0·30 (0·22 to 0·37)*	0·59 (0·43 to 0·75)*	0·12 (-0·00 to 0·22)
Maryland	80·9 (79·5 to 82·2)	85·0 (83·8 to 86·2)	86·7 (85·6 to 87·8)	87·7 (86·7 to 88·7)	88·9 (88·1 to 89·7)	88·9 (87·7 to 90·1)	8·1 (6·4 to 9·7)*	5·8 (4·4 to 7·3)*	2·2 (0·9 to 3·6)*	0·37 (0·29 to 0·44)*	0·70 (0·52 to 0·88)*	0·16 (0·06 to 0·26)*
Massachusetts	85·6 (84·4 to 86·9)	89·6 (88·6 to 90·7)	91·0 (90·1 to 91·9)	91·9 (91·1 to 92·7)	92·9 (92·2 to 93·6)	92·5 (91·4 to 93·6)	6·9 (5·4 to 8·5)*	5·4 (4·2 to 6·7)*	1·5 (0·2 to 2·6)*	0·30 (0·23 to 0·37)*	0·62 (0·47 to 0·76)*	0·10 (0·01 to 0·18)*
Michigan	79·5 (78·2 to 80·7)	83·9 (82·7 to 85·0)	85·4 (84·2 to 86·4)	86·1 (85·0 to 87·1)	86·9 (85·8 to 88·0)	87·1 (85·3 to 88·8)	7·6 (5·6 to 9·4)*	5·8 (4·6 to 7·2)*	1·7 (-0·1 to 3·5)	0·35 (0·26 to 0·43)*	0·71 (0·55 to 0·88)*	0·12 (-0·01 to 0·25)
Minnesota	86·5 (85·3 to 87·7)	89·4 (88·3 to 90·4)	90·9 (89·8 to 91·9)	91·7 (90·7 to 92·7)	92·4 (91·5 to 93·2)	92·3 (90·6 to 93·6)	5·8 (4·0 to 7·4)*	4·4 (3·0 to 5·8)*	1·4 (-0·2 to 2·9)	0·25 (0·17 to 0·32)*	0·49 (0·34 to 0·65)*	0·10 (-0·02 to 0·20)
Mississippi	74·5 (73·0 to 76·1)	78·5 (76·9 to 80·0)	79·5 (77·9 to 81·1)	79·8 (78·1 to 81·2)	81·5 (79·9 to 82·8)	81·5 (78·6 to 84·2)	7·0 (4·0 to 10·1)*	5·0 (3·3 to 6·8)*	2·0 (-1·1 to 4·7)	0·35 (0·20 to 0·49)*	0·65 (0·42 to 0·88)*	0·15 (-0·09 to 0·36)
Missouri	80·7 (79·2 to 81·9)	84·4 (83·1 to 85·6)	85·6 (84·4 to 86·8)	86·5 (85·3 to 87·7)	87·7 (86·5 to 88·8)	87·6 (86·0 to 89·1)	7·0 (5·0 to 8·8)*	5·0 (3·4 to 6·3)*	2·0 (0·2 to 3·6)*	0·32 (0·23 to 0·40)*	0·60 (0·41 to 0·76)*	0·14 (0·01 to 0·26)*
Montana	80·8 (79·1 to 82·4)	85·0 (83·4 to 86·6)	86·5 (84·8 to 88·0)	87·1 (85·4 to 88·4)	88·0 (86·4 to 89·1)	88·7 (86·2 to 90·6)	7·8 (5·3 to 10·2)*	5·7 (3·8 to 7·6)*	2·1 (-0·4 to 4·3)	0·36 (0·24 to 0·46)*	0·68 (0·46 to 0·91)*	0·15 (-0·03 to 0·31)
Nebraska	83·1 (81·8 to 84·3)	86·4 (85·3 to 87·5)	87·2 (86·1 to 88·3)	87·8 (86·6 to 88·8)	89·1 (87·9 to 90·1)	88·9 (87·1 to 90·5)	5·8 (3·8 to 7·6)*	4·2 (2·8 to 5·6)*	1·7 (-0·1 to 3·5)	0·26 (0·17 to 0·34)*	0·49 (0·33 to 0·65)*	0·12 (-0·00 to 0·25)
Nevada	79·5 (78·1 to 80·9)	83·5 (82·2 to 84·8)	84·9 (83·7 to 86·2)	84·9 (83·7 to 86·2)	87·6 (86·5 to 88·8)	87·1 (85·2 to 89·0)	7·6 (5·4 to 9·7)*	5·4 (3·9 to 6·9)*	2·2 (0·1 to 4·4)*	0·35 (0·25 to 0·44)*	0·66 (0·48 to 0·84)*	0·16 (0·01 to 0·32)*
New Hampshire	84·8 (83·6 to 86·1)	88·8 (87·7 to 90·0)	90·8 (89·7 to 91·7)	91·8 (90·8 to 92·7)	92·1 (91·3 to 93·0)	91·7 (90·4 to 92·8)	6·9 (5·1 to 8·3)*	6·0 (4·7 to 7·3)*	0·9 (-0·6 to 2·2)	0·30 (0·22 to 0·36)*	0·68 (0·54 to 0·83)*	0·06 (-0·04 to 0·15)
New Jersey	82·6 (81·2 to 84·0)	86·7 (85·5 to 87·9)	88·4 (87·5 to 89·5)	89·2 (88·3 to 90·3)	90·3 (89·5 to 91·3)	90·4 (88·8 to 91·7)	7·8 (6·0 to 9·6)*	5·9 (4·3 to 7·3)*	1·9 (0·2 to 3·4)*	0·35 (0·27 to 0·43)*	0·69 (0·51 to 0·86)*	0·13 (0·02 to 0·24)*
New Mexico	77·6 (76·2 to 79·3)	81·9 (80·5 to 83·5)	83·8 (82·4 to 85·3)	84·2 (82·7 to 85·7)	86·0 (84·7 to 87·5)	85·4 (82·8 to 88·0)	7·8 (5·0 to 10·4)*	6·1 (4·4 to 8·0)*	1·6 (-1·1 to 4·3)	0·37 (0·24 to 0·49)*	0·76 (0·55 to 0·99)*	0·12 (-0·08 to 0·31)
New York	80·1 (78·7 to 81·6)	85·6 (84·2 to 86·9)	88·5 (87·3 to 89·7)	89·4 (88·4 to 90·6)	90·5 (89·6 to 91·5)	90·7 (88·8 to 92·4)	10·6 (8·3 to 12·6)*	8·4 (6·8 to 9·9)*	2·2 (0·2 to 3·9)*	0·48 (0·37 to 0·57)*	0·99 (0·79 to 1·18)*	0·15 (0·01 to 0·28)*
North Carolina	78·7 (77·3 to 79·9)	83·5 (82·3 to 84·6)	84·6 (83·3 to 85·7)	85·4 (84·2 to 86·4)	87·2 (86·2 to 88·2)	86·8 (85·2 to 88·4)	8·1 (6·3 to 10·1)*	5·9 (4·5 to 7·3)*	2·3 (0·5 to 4·1)*	0·38 (0·30 to 0·47)*	0·72 (0·56 to 0·90)*	0·16 (0·03 to 0·30)*
North Dakota	82·8 (81·3 to 84·2)	86·6 (85·2 to 87·8)	88·5 (87·1 to 89·8)	88·8 (87·4 to 90·1)	89·1 (87·7 to 90·4)	89·9 (87·7 to 91·8)	7·1 (4·6 to 9·6)*	5·8 (4·1 to 7·5)*	1·4 (-1·0 to 3·7)	0·32 (0·21 to 0·43)*	0·67 (0·47 to 0·87)*	0·10 (-0·07 to 0·26)
Ohio	81·1 (79·9 to 82·3)	85·0 (83·9 to 86·2)	86·0 (84·9 to 87·1)	86·6 (85·5 to 87·6)	87·1 (86·1 to 88·2)	87·1 (85·5 to 88·5)	6·0 (4·1 to 7·7)*	4·9 (3·6 to 6·3)*	1·1 (-0·7 to 2·7)	0·27 (0·19 to 0·36)*	0·59 (0·43 to 0·75)*	0·08 (-0·05 to 0·19)
Oklahoma	80·5 (78·9 to 81·7)	83·3 (81·8 to 84·6)	83·7 (82·2 to 85·0)	83·7 (82·1 to 85·0)	84·4 (82·9 to 85·6)	84·5 (82·7 to 86·2)	4·1 (2·2 to 6·1)*	3·3 (1·9 to 4·6)*	0·8 (-1·2 to 2·6)	0·19 (0·10 to 0·29)*	0·40 (0·23 to 0·56)*	0·06 (-0·09 to 0·20)
Oregon	83·6 (82·2 to 84·9)	87·5 (86·2 to 88·6)	89·1 (87·9 to 90·2)	89·6 (88·4 to 90·7)	91·0 (89·8 to 92·0)	90·9 (89·5 to 92·3)	7·3 (5·5 to 8·9)*	5·5 (4·2 to 6·9)*	1·8 (0·2 to 3·3)*	0·32 (0·25 to 0·39)*	0·64 (0·48 to 0·80)*	0·12 (0·01 to 0·23)*
Pennsylvania	81·5 (80·4 to 82·7)	85·9 (84·7 to 87·0)	87·6 (86·6 to 88·6)	87·9 (86·9 to 88·9)	88·8 (87·9 to 89·8)	89·2 (87·9 to 90·4)	7·7 (6·1 to 9·3)*	6·1 (4·9 to 7·4)*	1·6 (0·1 to 3·0)*	0·35 (0·28 to 0·42)*	0·72 (0·58 to 0·87)*	0·12 (0·01 to 0·21)*
Rhode Island	83·0 (81·6 to 84·6)	87·3 (86·0 to 88·5)	88·6 (87·3 to 90·0)	89·5 (88·3 to 90·7)	90·6 (89·6 to 91·7)	90·4 (88·3 to 92·1)	7·3 (5·2 to 9·4)*	5·6 (3·9 to 7·2)*	1·8 (-0·2 to 3·8)	0·33 (0·23 to 0·41)*	0·65 (0·46 to 0·84)*	0·12 (-0·02 to 0·26)
South Carolina	75·9 (74·3 to 77·5)	81·3 (79·7 to 82·7)	82·4 (80·8 to 83·9)	83·2 (81·6 to 84·6)	85·5 (84·0 to 86·9)	85·2 (82·6 to 87·5)	9·3 (6·3 to 12·0)*	6·4 (4·6 to 8·2)*	2·9 (-0·0 to 5·5)	0·44 (0·31 to 0·57)*	0·81 (0·58 to 1·04)*	0·21 (-0·00 to 0·41)
South Dakota	80·5 (78·9 to 82·0)	84·6 (83·0 to 86·0)	85·9 (84·4 to 87·3)	86·4 (84·8 to 87·7)	87·2 (85·6 to 88·6)	86·8 (84·5 to 89·0)	6·3 (3·8 to 8·9)*	5·5 (3·7 to 7·3)*	0·9 (-1·6 to 3·2)	0·29 (0·17 to 0·41)*	0·66 (0·44 to 0·87)*	0·06 (-0·12 to 0·23)
Tennessee	78·7 (77·3 to 79·9)	82·5 (81·2 to 83·7)	83·7 (82·5 to 85·0)	84·5 (83·3 to 85·6)	85·5 (84·3 to 86·7)	85·1 (83·4 to 86·9)	6·4 (4·5 to 8·4)*	5·0 (3·7 to 6·4)*	1·4 (-0·3 to 3·4)	0·30 (0·21 to 0·39)*	0·62 (0·45 to 0·79)*	0·11 (-0·02 to 0·25)
Texas	79·7 (78·2 to 80·9)	83·5 (82·2 to 84·6)	85·0 (83·9 to 86·0)	85·2 (84·2 to 86·2)	86·9 (85·7 to 88·0)	86·8 (85·0 to 88·5)	7·1 (5·0 to 9·3)*	5·3 (3·9 to 6·7)*	1·8 (0·0 to 3·8)	0·33 (0·23 to 0·43)*	0·64 (0·48 to 0·82)*	0·13 (0·00 to 0·28)
Utah	82·0 (80·7 to 83·3)	85·5 (84·3 to 86·7)	86·5 (85·1 to 87·6)	87·2 (85·9 to 88·2)	88·6 (87·3 to 89·6)	88·6 (86·9 to 90·0)	6·6 (4·7 to 8·4)*	4·5 (3·1 to 6·0)*	2·1 (0·5 to 3·7)*	0·30 (0·21 to 0·38)*	0·54 (0·36 to 0·71)*	0·15 (0·03 to 0·27)*
Vermont	83·2 (81·7 to 84·8)	87·3 (85·9 to 88·7)	89·8 (88·6 to 91·0)	90·4 (89·2 to 91·6)	91·8 (90·7 to 92·8)	91·2 (89·7 to 92·6)	8·0 (6·0 to 9·8)*	6·6 (5·0 to 8·2)*	1·5 (-0·2 to 3·1)	0·35 (0·27 to 0·43)*	0·76 (0·58 to 0·95)*	0·10 (-0·01 to 0·21)
Virginia	81·8 (80·4 to 83·1)	86·2 (85·1 to 87·3)	87·7 (86·6 to 88·7)	88·4 (87·4 to 89·4)	90·0 (89·0 to 90·8)	90·0 (88·6 to 91·1)	8·2 (6·4 to 9·8)*	6·0 (4·5 to 7·3)*	2·2 (0·8 to 3·7)*	0·37 (0·29 to 0·44)*	0·70 (0·54 to 0·86)*	0·16 (0·05 to 0·26)*
Washington	84·5 (83·2 to 85·6)	88·5 (87·4 to 89·5)	90·3 (89·2 to 91·3)	90·9 (89·9 to 91·9)	91·9 (90·9 to 92·8)	92·0 (90·6 to 93·3)	7·5 (5·8 to 9·3)*	5·8 (4·5 to 7·2)*	1·7 (0·3 to 3·2)*	0·33 (0·25 to 0·40)*	0·66 (0·52 to 0·82)*	0·12 (0·02 to 0·22)*
West Virginia	78·9 (77·5 to 80·1)	82·6 (81·3 to 83·8)	83·6 (82·3 to 84·7)	84·0 (82·8 to 85·2)	84·3 (83·0 to 85·6)	84·1 (82·1 to 85·8)	5·2 (3·0 to 7·2)*	4·8 (3·3 to 6·2)*	0·5 (-1·5 to 2·5)	0·25 (0·14 to 0·34)*	0·59 (0·41 to 0·76)*	0·04 (-0·12 to 0·18)
Wisconsin	84·5 (83·3 to 85·6)	88·0 (86·9 to 89·0)	88·9 (87·8 to 90·0)	89·6 (88·5 to 90·5)	90·6 (89·5 to 91·5)	90·6 (89·2 to 91·8)	6·1 (4·4 to 7·6)*	4·4 (3·0 to 5·7)*	1·7 (0·0 to 3·2)	0·27 (0·19 to 0·34)*	0·51 (0·34 to 0·66)*	0·12 (0·00 to 0·23)
Wyoming	81·6 (79·9 to 83·0)	85·3 (83·7 to 86·6)	86·1 (84·6 to 87·5)	86·4 (84·9 to 87·7)	87·6 (86·3 to 88·7)	87·7 (85·7 to 89·5)	6·1 (3·6 to 8·5)*	4·5 (2·7 to 6·5)*	1·6 (-0·7 to 3·8)	0·28 (0·16 to 0·39)*	0·54 (0·32 to 0·78)*	0·11 (-0·05 to 0·27)
Southern Latin America	54·2 (52·9 to 55·5)	58·9 (57·5 to 60·0)	62·6 (61·0 to 63·8)	64·6 (62·9 to 65·7)	66·7 (65·3 to 67·8)	70·0 (67·9 to 72·0)	15·8 (13·7 to 17·8)*	8·4 (6·8 to 9·7)*	7·4 (5·2 to 9·5)*	0·99 (0·86 to 1·10)*	1·45 (1·17 to 1·66)*	0·70 (0·50 to 0·89)*
Argentina	53·8 (52·3 to 55·2)	58·3 (56·8 to 59·6)	61·7 (59·8 to 63·1)	63·2 (61·4 to 64·5)	65·0 (63·5 to 66·2)	68·1 (65·8 to 70·1)	14·3 (12·0 to 16·5)*	8·0 (6·1 to 9·5)*	6·3 (4·2 to 8·5)*	0·91 (0·77 to 1·04)*	1·38 (1·06 to 1·64)*	0·61 (0·41 to 0·82)*
Chile	56·5 (54·9 to 58·4)	61·9 (60·3 to 63·5)	67·0 (65·4 to 68·5)	70·9 (69·3 to 72·4)	74·0 (72·3 to 75·6)	77·9 (72·3 to 83·7)	21·4 (15·5 to 27·5)*	10·5 (8·4 to 12·5)*	10·9 (5·3 to 16·4)*	1·23 (0·93 to 1·53)*	1·70 (1·35 to 2·03)*	0·94 (0·47 to 1·39)*
Uruguay	57·9 (56·7 to 59·1)	60·7 (59·5 to 61·8)	64·7 (63·2 to 65·8)	66·3 (64·8 to 67·4)	68·3 (67·1 to 69·6)	71·0 (68·9 to 73·0)	13·1 (10·9 to 15·2)*	6·8 (5·1 to 8·2)*	6·3 (4·1 to 8·5)*	0·79 (0·66 to 0·90)*	1·12 (0·84 to 1·34)*	0·58 (0·39 to 0·78)*
Western Europe	78·6 (77·9 to 79·6)	82·4 (81·7 to 83·1)	85·3 (84·6 to 86·0)	88·1 (87·3 to 88·7)	90·4 (89·7 to 91·0)	92·6 (91·7 to 93·3)	13·9 (12·8 to 14·8)*	6·7 (6·0 to 7·3)*	7·2 (6·6 to 7·9)*	0·63 (0·58 to 0·67)*	0·82 (0·73 to 0·90)*	0·51 (0·46 to 0·56)*
Andorra	84·7 (79·5 to 89·3)	88·5 (83·9 to 93·0)	92·8 (88·9 to 96·0)	94·3 (91·0 to 96·7)	94·8 (91·8 to 97·0)	94·7 (91·2 to 97·0)	10·0 (4·4 to 15·4)*	8·1 (3·8 to 12·6)*	1·8 (-2·5 to 5·8)	0·43 (0·19 to 0·67)*	0·92 (0·43 to 1·45)*	0·12 (-0·17 to 0·39)
Austria	80·9 (79·9 to 82·2)	84·4 (83·5 to 85·5)	87·4 (86·5 to 88·5)	89·9 (89·0 to 90·9)	91·6 (90·7 to 92·7)	93·9 (92·6 to 95·3)	13·1 (11·3 to 14·7)*	6·6 (5·6 to 7·6)*	6·5 (5·1 to 8·0)*	0·58 (0·50 to 0·65)*	0·78 (0·66 to 0·91)*	0·45 (0·36 to 0·55)*
Belgium	80·7 (79·4 to 82·2)	83·9 (82·7 to 85·1)	86·1 (84·8 to 87·3)	88·7 (87·4 to 89·9)	90·2 (88·8 to 91·4)	92·9 (90·7 to 95·0)	12·2 (9·6 to 14·7)*	5·4 (3·7 to 7·1)*	6·8 (4·6 to 9·1)*	0·54 (0·43 to 0·65)*	0·65 (0·44 to 0·85)*	0·47 (0·32 to 0·63)*
Cyprus	68·3 (66·3 to 70·5)	73·0 (71·1 to 74·8)	78·0 (76·6 to 79·7)	83·1 (81·8 to 84·6)	88·4 (87·1 to 89·6)	90·3 (88·8 to 91·8)	22·0 (19·6 to 24·3)*	9·6 (7·7 to 11·5)*	12·3 (10·5 to 14·3)*	1·07 (0·95 to 1·20)*	1·32 (1·04 to 1·59)*	0·92 (0·78 to 1·06)*
Denmark	81·1 (79·3 to 82·7)	82·5 (81·0 to 84·1)	85·0 (83·5 to 86·8)	87·4 (85·6 to 89·0)	90·0 (88·6 to 91·6)	92·1 (89·8 to 94·3)	11·0 (8·2 to 13·7)*	3·8 (1·7 to 6·5)*	7·2 (4·5 to 10·0)*	0·49 (0·36 to 0·61)*	0·46 (0·20 to 0·78)*	0·51 (0·32 to 0·70)*
Finland	81·0 (79·8 to 82·3)	84·4 (83·3 to 85·5)	87·7 (86·7 to 88·7)	90·4 (89·4 to 91·4)	93·0 (92·0 to 94·0)	95·9 (94·6 to 96·9)	14·9 (13·0 to 16·5)*	6·8 (5·3 to 8·0)*	8·1 (6·7 to 9·5)*	0·65 (0·56 to 0·72)*	0·80 (0·62 to 0·95)*	0·55 (0·46 to 0·65)*
France	77·6 (76·4 to 79·1)	81·3 (80·1 to 82·9)	84·1 (83·0 to 85·3)	87·6 (86·5 to 88·6)	89·9 (88·8 to 91·0)	91·7 (90·3 to 93·1)	14·1 (12·1 to 16·0)*	6·6 (5·4 to 7·7)*	7·6 (6·0 to 9·1)*	0·64 (0·55 to 0·73)*	0·81 (0·66 to 0·95)*	0·54 (0·42 to 0·65)*

Supplementary table 3. **Global, regional, national or territory, and select subnational estimates on the HAQ Index for 1990–2016, and absolute change and annualised rates of change for 1990–2016, 1990–2000, and 2000–2016.** Starred estimates of change represent statistically significant changes during a given time period. HAQ Index=Healthcare Access and Quality Index.

	HAQ Index						Absolute change			Annualised rate of change		
	1990	1995	2000	2005	2010	2016	1990-2016	1990-2000	2000-2016	1990-2016	1990-2000	2000-2016
Germany	78·9 (77·5 to 80·6)	83·4 (82·1 to 84·7)	86·1 (84·9 to 87·3)	88·1 (87·0 to 89·3)	89·6 (88·5 to 90·8)	92·0 (90·4 to 93·6)	13·1 (10·8 to 15·1)*	7·2 (5·4 to 8·9)*	5·9 (4·1 to 8·0)*	0·59 (0·49 to 0·68)*	0·87 (0·65 to 1·09)*	0·42 (0·29 to 0·56)*
Greece	79·5 (78·4 to 80·5)	82·4 (81·6 to 83·4)	85·3 (84·4 to 86·3)	88·2 (87·3 to 89·1)	90·0 (89·2 to 91·0)	90·4 (88·8 to 91·9)	10·9 (9·1 to 12·6)*	5·8 (4·8 to 6·8)*	5·1 (3·5 to 6·7)*	0·49 (0·42 to 0·57)*	0·70 (0·58 to 0·84)*	0·36 (0·25 to 0·47)*
Iceland	87·0 (85·6 to 88·5)	89·7 (88·4 to 91·1)	92·8 (91·5 to 93·9)	95·4 (94·3 to 96·5)	96·7 (95·8 to 97·7)	97·1 (95·8 to 98·1)	10·2 (8·6 to 11·7)*	5·8 (4·1 to 7·3)*	4·4 (2·8 to 6·0)*	0·42 (0·36 to 0·49)*	0·65 (0·46 to 0·81)*	0·29 (0·18 to 0·39)*
Ireland	76·3 (74·9 to 77·5)	80·5 (79·1 to 81·8)	83·9 (82·4 to 85·4)	89·3 (87·8 to 90·6)	91·8 (90·3 to 93·2)	94·6 (91·8 to 96·8)	18·3 (15·3 to 20·9)*	7·6 (6·0 to 9·3)*	10·7 (7·8 to 13·4)*	0·83 (0·70 to 0·94)*	0·95 (0·75 to 1·17)*	0·75 (0·55 to 0·93)*
Israel	71·2 (68·9 to 73·7)	75·2 (73·0 to 77·6)	77·9 (75·5 to 80·5)	80·9 (78·3 to 83·0)	83·0 (80·8 to 85·0)	84·8 (80·7 to 88·4)	13·5 (8·6 to 18·0)*	6·7 (3·4 to 10·0)*	6·8 (2·3 to 10·8)*	0·67 (0·43 to 0·88)*	0·90 (0·46 to 1·34)*	0·52 (0·18 to 0·83)*
Italy	81·5 (80·6 to 82·4)	85·1 (84·1 to 85·9)	88·8 (87·8 to 89·7)	91·8 (91·0 to 92·6)	93·5 (92·6 to 94·3)	94·9 (93·4 to 96·0)	13·3 (11·8 to 14·7)*	7·2 (6·3 to 8·1)*	6·1 (4·7 to 7·4)*	0·58 (0·52 to 0·64)*	0·85 (0·74 to 0·96)*	0·41 (0·32 to 0·51)*
Luxembourg	81·4 (79·7 to 83·0)	86·4 (85·0 to 87·9)	90·3 (88·8 to 91·6)	93·1 (91·7 to 94·4)	94·9 (93·6 to 96·0)	96·0 (94·4 to 97·3)	14·7 (12·4 to 16·7)*	8·9 (7·2 to 10·6)*	5·7 (3·9 to 7·4)*	0·64 (0·53 to 0·73)*	1·04 (0·83 to 1·24)*	0·38 (0·26 to 0·49)*
Malta	75·0 (73·0 to 77·0)	77·9 (75·9 to 80·0)	81·1 (79·0 to 83·0)	84·2 (82·2 to 86·4)	87·2 (85·3 to 89·1)	89·9 (86·3 to 93·0)	14·9 (10·8 to 18·8)*	6·1 (3·5 to 8·7)*	8·8 (4·9 to 12·6)*	0·70 (0·52 to 0·87)*	0·78 (0·45 to 1·11)*	0·64 (0·36 to 0·91)*
Netherlands	84·1 (82·8 to 85·4)	86·7 (85·4 to 87·9)	88·6 (87·1 to 89·8)	91·9 (90·6 to 93·0)	94·6 (93·4 to 95·6)	96·1 (94·5 to 97·3)	11·9 (10·0 to 13·6)*	4·5 (3·1 to 6·0)*	7·4 (5·6 to 9·1)*	0·51 (0·43 to 0·58)*	0·52 (0·36 to 0·69)*	0·50 (0·38 to 0·62)*
Norway	84·0 (82·9 to 85·1)	87·5 (86·5 to 88·6)	90·6 (89·5 to 91·7)	93·4 (92·3 to 94·3)	94·9 (94·0 to 95·8)	96·6 (94·9 to 97·9)	12·6 (10·6 to 14·3)*	6·6 (5·4 to 7·9)*	6·0 (4·1 to 7·6)*	0·54 (0·46 to 0·61)*	0·76 (0·62 to 0·91)*	0·40 (0·27 to 0·51)*
Portugal	67·1 (65·9 to 68·3)	72·2 (71·1 to 73·3)	76·2 (75·1 to 77·3)	80·5 (79·2 to 81·4)	82·7 (81·5 to 83·7)	85·7 (84·1 to 87·3)	18·6 (16·9 to 20·4)*	9·1 (8·0 to 10·4)*	9·5 (7·8 to 11·3)*	0·94 (0·86 to 1·03)*	1·27 (1·11 to 1·46)*	0·74 (0·61 to 0·87)*
Spain	76·2 (75·2 to 77·2)	80·5 (79·6 to 81·4)	84·1 (83·1 to 84·9)	87·2 (86·2 to 88·1)	90·1 (89·2 to 91·0)	91·9 (90·5 to 93·2)	15·7 (14·2 to 17·3)*	7·9 (6·9 to 8·8)*	7·8 (6·5 to 9·2)*	0·72 (0·65 to 0·79)*	0·99 (0·87 to 1·11)*	0·56 (0·46 to 0·65)*
Sweden	85·2 (84·2 to 86·2)	89·7 (88·7 to 90·7)	92·4 (91·5 to 93·2)	93·5 (92·6 to 94·4)	94·4 (93·5 to 95·3)	95·5 (93·4 to 97·2)	10·2 (7·9 to 12·1)*	7·1 (6·1 to 8·2)*	3·1 (1·0 to 5·0)*	0·44 (0·34 to 0·51)*	0·81 (0·69 to 0·93)*	0·21 (0·07 to 0·33)*
Switzerland	86·8 (85·2 to 88·2)	89·6 (88·1 to 90·9)	91·6 (90·2 to 93·0)	93·4 (92·3 to 94·5)	94·4 (93·3 to 95·5)	95·6 (92·4 to 97·8)	8·8 (5·3 to 11·4)*	4·8 (3·0 to 6·6)*	4·0 (0·5 to 6·7)*	0·37 (0·22 to 0·48)*	0·54 (0·33 to 0·74)*	0·26 (0·04 to 0·45)*
United Kingdom	78·0 (77·1 to 78·6)	81·1 (80·2 to 81·8)	83·9 (83·0 to 84·6)	86·2 (85·4 to 86·9)	88·5 (87·7 to 89·2)	90·5 (89·6 to 91·3)	12·5 (11·8 to 13·4)*	6·0 (5·5 to 6·5)*	6·5 (5·9 to 7·2)*	0·57 (0·54 to 0·61)*	0·74 (0·69 to 0·80)*	0·47 (0·43 to 0·51)*
England	78·3 (77·5 to 79·0)	81·5 (80·7 to 82·2)	84·3 (83·5 to 85·0)	86·5 (85·7 to 87·3)	88·8 (88·1 to 89·6)	90·7 (90·0 to 91·6)	12·3 (11·7 to 13·3)*	6·0 (5·6 to 6·6)*	6·4 (5·8 to 7·1)*	0·56 (0·53 to 0·61)*	0·73 (0·69 to 0·81)*	0·46 (0·42 to 0·51)*
East Midlands	77·6 (76·6 to 78·5)	80·8 (79·9 to 81·7)	83·7 (82·6 to 84·6)	86·0 (85·0 to 87·0)	88·1 (87·1 to 89·1)	89·7 (88·6 to 91·1)	12·2 (10·9 to 13·6)*	6·1 (5·2 to 7·1)*	6·0 (4·8 to 7·4)*	0·56 (0·50 to 0·62)*	0·76 (0·64 to 0·89)*	0·44 (0·35 to 0·53)*
East of England	80·8 (79·8 to 81·7)	83·8 (82·9 to 84·7)	86·7 (85·9 to 87·7)	88·9 (88·0 to 89·9)	90·7 (89·8 to 91·8)	92·2 (91·1 to 93·5)	11·5 (10·2 to 12·7)*	6·0 (5·1 to 6·9)*	5·5 (4·3 to 6·7)*	0·51 (0·46 to 0·56)*	0·71 (0·60 to 0·83)*	0·38 (0·30 to 0·46)*
Greater London	79·7 (78·8 to 80·5)	82·6 (81·7 to 83·4)	85·1 (84·3 to 85·9)	87·4 (86·6 to 88·2)	89·9 (89·1 to 90·9)	92·1 (91·3 to 93·2)	12·4 (11·5 to 13·6)*	5·4 (4·8 to 6·1)*	7·0 (6·2 to 7·8)*	0·56 (0·52 to 0·61)*	0·66 (0·59 to 0·74)*	0·49 (0·44 to 0·55)*
North East England	74·7 (73·8 to 75·7)	77·6 (76·7 to 78·6)	80·6 (79·6 to 81·6)	83·2 (82·2 to 84·2)	86·0 (85·0 to 87·1)	87·8 (86·6 to 89·1)	13·1 (11·8 to 14·5)*	5·9 (5·1 to 6·8)*	7·2 (5·8 to 8·4)*	0·62 (0·56 to 0·68)*	0·76 (0·66 to 0·88)*	0·53 (0·43 to 0·63)*
North West England	75·4 (74·6 to 76·3)	78·8 (77·8 to 79·6)	81·4 (80·4 to 82·3)	83·6 (82·7 to 84·6)	85·9 (85·0 to 86·9)	88·0 (87·1 to 89·1)	12·6 (11·5 to 13·8)*	6·0 (5·2 to 6·8)*	6·6 (5·7 to 7·6)*	0·59 (0·54 to 0·65)*	0·76 (0·67 to 0·86)*	0·49 (0·42 to 0·56)*
South East England	81·0 (80·1 to 81·9)	84·4 (83·4 to 85·2)	87·2 (86·3 to 88·1)	89·3 (88·4 to 90·3)	91·2 (90·4 to 92·2)	92·8 (91·8 to 93·8)	11·7 (10·7 to 13·0)*	6·2 (5·4 to 7·0)*	5·6 (4·6 to 6·6)*	0·52 (0·47 to 0·57)*	0·74 (0·65 to 0·84)*	0·39 (0·32 to 0·46)*
South West England	80·6 (79·5 to 81·4)	83·7 (82·6 to 84·6)	86·8 (85·8 to 87·7)	88·9 (88·0 to 89·8)	90·8 (89·9 to 91·7)	92·4 (91·4 to 93·4)	11·8 (10·7 to 13·0)*	6·3 (5·5 to 7·1)*	5·6 (4·5 to 6·7)*	0·53 (0·48 to 0·58)*	0·75 (0·65 to 0·84)*	0·39 (0·31 to 0·46)*
West Midlands	75·8 (74·9 to 76·8)	79·4 (78·4 to 80·3)	81·6 (80·6 to 82·7)	83·7 (82·8 to 84·8)	86·3 (85·4 to 87·5)	88·1 (86·9 to 89·6)	12·3 (11·1 to 13·7)*	5·8 (4·9 to 6·8)*	6·5 (5·4 to 7·7)*	0·58 (0·52 to 0·64)*	0·74 (0·62 to 0·86)*	0·48 (0·40 to 0·56)*
Yorkshire and the Humber	76·7 (75·8 to 77·5)	79·7 (78·7 to 80·5)	82·6 (81·7 to 83·4)	85·0 (84·0 to 85·9)	87·3 (86·4 to 88·2)	89·0 (88·0 to 90·0)	12·2 (11·1 to 13·4)*	5·9 (5·2 to 6·7)*	6·3 (5·3 to 7·4)*	0·57 (0·52 to 0·62)*	0·74 (0·65 to 0·84)*	0·46 (0·39 to 0·54)*
Northern Ireland	78·6 (76·1 to 80·7)	82·1 (79·4 to 84·3)	85·4 (83·0 to 87·4)	87·5 (84·9 to 89·7)	89·4 (86·8 to 91·2)	91·6 (88·4 to 94·1)	13·0 (9·9 to 15·8)*	6·8 (4·5 to 9·2)*	6·2 (3·0 to 9·0)*	0·59 (0·45 to 0·71)*	0·83 (0·55 to 1·13)*	0·43 (0·22 to 0·65)*
Scotland	74·2 (72·4 to 76·1)	77·9 (75·8 to 79·6)	80·7 (78·7 to 82·6)	83·4 (81·3 to 85·3)	86·3 (84·3 to 88·1)	88·5 (85·6 to 91·2)	14·3 (11·2 to 17·4)*	6·5 (4·3 to 8·6)*	7·8 (4·8 to 10·5)*	0·68 (0·53 to 0·82)*	0·84 (0·56 to 1·11)*	0·58 (0·35 to 0·77)*
Wales	76·1 (74·4 to 77·7)	79·0 (77·3 to 80·8)	82·1 (80·2 to 84·1)	85·2 (83·4 to 87·1)	87·3 (85·3 to 89·0)	89·3 (86·8 to 91·8)	13·2 (10·5 to 16·0)*	6·1 (3·9 to 8·3)*	7·2 (4·2 to 10·0)*	0·62 (0·49 to 0·74)*	0·77 (0·50 to 1·04)*	0·52 (0·31 to 0·72)*
Latin America and Caribbean	41·3 (40·3 to 42·5)	48·0 (47·0 to 49·0)	52·6 (51·3 to 53·7)	55·8 (54·3 to 56·9)	58·2 (56·7 to 59·3)	61·8 (60·4 to 63·0)	20·5 (19·0 to 21·8)*	11·3 (9·8 to 12·3)*	9·2 (8·1 to 10·2)*	1·55 (1·43 to 1·65)*	2·42 (2·09 to 2·66)*	1·01 (0·89 to 1·12)*
Andean Latin America	34·1 (32·4 to 36·0)	39·6 (38·0 to 41·3)	46·9 (45·3 to 48·6)	51·8 (50·0 to 53·8)	54·9 (52·9 to 56·7)	59·3 (56·3 to 62·4)	25·2 (21·4 to 28·8)*	12·8 (10·0 to 15·0)*	12·4 (9·5 to 15·3)*	2·13 (1·82 to 2·42)*	3·19 (2·47 to 3·76)*	1·47 (1·14 to 1·77)*
Bolivia	26·2 (23·6 to 29·0)	31·4 (28·9 to 34·2)	36·5 (34·2 to 38·9)	41·0 (38·2 to 43·9)	44·5 (40·5 to 48·5)	48·8 (43·5 to 54·0)	22·6 (16·6 to 28·1)*	10·3 (7·1 to 13·2)*	12·3 (6·8 to 17·6)*	2·39 (1·82 to 2·93)*	3·31 (2·27 to 4·38)*	1·81 (1·08 to 2·51)*
Ecuador	37·8 (36·1 to 39·9)	44·5 (42·9 to 46·1)	51·1 (48·9 to 52·8)	55·4 (53·0 to 57·2)	58·4 (55·7 to 60·4)	62·2 (59·5 to 64·6)	24·3 (20·8 to 27·4)*	13·3 (10·4 to 15·6)*	11·1 (8·8 to 13·4)*	1·91 (1·63 to 2·15)*	3·01 (2·35 to 3·55)*	1·22 (0·98 to 1·47)*
Peru	38·6 (36·3 to 41·3)	42·8 (40·4 to 45·6)	51·0 (48·8 to 53·4)	56·5 (53·7 to 59·5)	59·2 (56·2 to 61·8)	64·3 (59·2 to 69·4)	25·8 (19·8 to 31·4)*	12·4 (8·7 to 15·7)*	13·4 (8·0 to 18·5)*	1·97 (1·52 to 2·34)*	2·79 (1·92 to 3·53)*	1·45 (0·90 to 1·97)*
Caribbean	37·9 (36·1 to 40·0)	43·3 (41·5 to 45·1)	45·6 (43·6 to 47·7)	49·0 (46·8 to 51·1)	50·5 (47·7 to 52·9)	54·2 (51·1 to 57·3)	16·3 (12·7 to 19·7)*	7·7 (5·0 to 10·2)*	8·7 (5·3 to 12·1)*	1·38 (1·09 to 1·64)*	1·85 (1·20 to 2·43)*	1·09 (0·67 to 1·50)*
Antigua and Barbuda	57·0 (54·5 to 59·5)	60·0 (57·4 to 62·7)	62·8 (60·2 to 65·4)	65·2 (62·7 to 67·6)	68·0 (65·3 to 70·6)	69·8 (66·5 to 73·3)	12·8 (8·7 to 16·7)*	5·8 (2·7 to 9·0)*	7·0 (3·2 to 11·2)*	0·78 (0·53 to 1·01)*	0·97 (0·46 to 1·51)*	0·66 (0·31 to 1·04)*
Barbados	59·3 (57·1 to 61·6)	64·3 (61·7 to 66·6)	67·3 (64·3 to 69·7)	68·4 (65·9 to 70·6)	70·7 (68·4 to 72·8)	70·8 (67·3 to 73·8)	11·6 (7·5 to 15·4)*	8·0 (4·8 to 11·0)*	3·6 (-0·2 to 7·5)	0·69 (0·45 to 0·90)*	1·27 (0·76 to 1·73)*	0·32 (-0·02 to 0·67)
Belize	46·6 (44·3 to 48·8)	48·5 (46·2 to 50·7)	48·6 (46·1 to 50·8)	51·3 (48·8 to 53·8)	53·7 (50·9 to 56·4)	55·7 (50·8 to 59·9)	9·1 (4·0 to 13·6)*	2·0 (-1·0 to 4·8)	7·2 (2·5 to 11·4)*	0·69 (0·31 to 1·01)*	0·41 (-0·21 to 1·02)	0·86 (0·31 to 1·35)*
Bermuda	63·1 (60·8 to 65·8)	66·9 (64·6 to 69·2)	73·5 (71·0 to 76·0)	76·4 (74·0 to 78·7)	79·7 (77·2 to 82·1)	83·1 (79·7 to 86·3)	20·0 (15·7 to 24·0)*	10·4 (6·8 to 13·7)*	9·6 (5·6 to 13·5)*	1·06 (0·84 to 1·26)*	1·52 (0·98 to 2·01)*	0·76 (0·45 to 1·08)*
Cuba	63·7 (62·4 to 65·5)	65·1 (63·7 to 66·5)	67·3 (66·2 to 68·6)	70·6 (69·2 to 71·9)	72·4 (71·1 to 73·7)	75·5 (73·5 to 77·7)	11·8 (9·5 to 14·2)*	3·6 (2·1 to 5·2)*	8·2 (6·0 to 10·4)*	0·65 (0·53 to 0·78)*	0·56 (0·32 to 0·79)*	0·72 (0·53 to 0·91)*
Dominica	52·4 (50·1 to 54·8)	54·1 (51·8 to 56·3)	58·9 (56·3 to 61·2)	61·2 (59·0 to 63·5)	60·7 (58·1 to 62·9)	61·9 (58·2 to 65·3)	9·5 (5·3 to 13·2)*	6·5 (3·8 to 9·3)*	3·0 (-1·3 to 6·9)	0·64 (0·37 to 0·88)*	1·18 (0·69 to 1·67)*	0·31 (-0·14 to 0·71)
Dominican Republic	38·4 (35·8 to 41·5)	46·7 (44·3 to 49·3)	52·5 (49·5 to 55·5)	54·9 (51·4 to 58·3)	57·9 (54·0 to 61·5)	61·2 (57·3 to 65·6)	22·8 (17·8 to 27·5)*	14·1 (9·6 to 18·1)*	8·7 (4·2 to 13·4)*	1·80 (1·40 to 2·14)*	3·14 (2·07 to 3·95)*	0·96 (0·46 to 1·44)*
Grenada	47·2 (44·1 to 50·4)	50·7 (47·9 to 53·4)	53·2 (50·4 to 55·8)	55·5 (52·7 to 58·3)	57·5 (54·7 to 60·4)	58·5 (54·7 to 62·2)	11·3 (6·7 to 16·2)*	5·9 (2·0 to 9·6)*	5·3 (1·2 to 9·7)*	0·82 (0·49 to 1·19)*	1·19 (0·40 to 1·95)*	0·60 (0·13 to 1·07)*
Guyana	38·4 (36·3 to 40·5)	41·4 (39·5 to 43·1)	43·2 (41·0 to 45·1)	44·0 (41·6 to 46·2)	45·4 (43·5 to 47·6)	49·8 (46·8 to 53·0)	11·4 (8·0 to 15·3)*	4·8 (1·9 to 7·2)*	6·6 (3·4 to 9·9)*	1·00 (0·71 to 1·32)*	1·19 (0·48 to 1·77)*	0·88 (0·46 to 1·32)*
Haiti	16·7 (13·8 to 19·8)	21·6 (18·8 to 24·6)	23·2 (19·6 to 26·9)	27·0 (22·8 to 31·3)	28·2 (23·1 to 33·2)	32·1 (26·6 to 37·8)	15·4 (9·5 to 21·4)*	6·5 (2·0 to 10·9)*	8·9 (2·7 to 15·1)*	2·51 (1·59 to 3·48)*	3·30 (1·02 to 5·61)*	2·02 (0·65 to 3·35)*
Jamaica	51·1 (48·2 to 54·2)	55·2 (51·8 to 58·5)	56·4 (52·4 to 59·8)	58·7 (54·9 to 62·0)	60·4 (55·9 to 64·3)	62·0 (56·8 to 67·3)	10·8 (5·0 to 16·7)*	5·2 (0·7 to 9·2)*	5·6 (0·2 to 10·9)*	0·74 (0·35 to 1·12)*	0·97 (0·13 to 1·69)*	0·59 (0·03 to 1·12)*
Puerto Rico	67·1 (65·7 to 68·8)	70·0 (68·4 to 71·7)	74·6 (73·0 to 76·2)	78·6 (76·9 to 80·2)	80·4 (78·6 to 82·0)	82·7 (80·2 to 85·0)	15·6 (12·7 to 18·2)*	7·5 (5·7 to 9·4)*	8·1 (5·5 to 10·7)*	0·80 (0·66 to 0·93)*	1·06 (0·80 to 1·32)*	0·64 (0·45 to 0·84)*
Saint Lucia	48·9 (46·6 to 51·1)	53·1 (51·1 to 55·1)	56·8 (54·5 to 58·9)	59·1 (56·8 to 61·1)	61·5 (59·2 to 63·6)	63·3 (60·3 to 66·0)	14·4 (10·9 to 17·7)*	7·9 (5·0 to 10·8)*	6·5 (3·3 to 9·7)*	1·00 (0·76 to 1·21)*	1·50 (0·95 to 2·07)*	0·68 (0·35 to 0·98)*
Saint Vincent and the Grenadines	49·6 (47·2 to 51·7)	50·5 (48·1 to 52·6)	53·0 (50·7 to 55·1)	55·8 (53·8 to 57·8)	56·8 (54·7 to 58·8)	57·4 (54·8 to 59·9)	7·8 (4·6 to 11·1)*	3·4 (0·9 to 5·8)*	4·4 (1·5 to 7·6)*	0·56 (0·34 to 0·79)*	0·66 (0·18 to 1·14)*	0·50 (0·17 to 0·86)*
Suriname	41·9 (39·9 to 44·2)	44·1 (42·1 to 46·3)	45·6 (43·0 to 47·9)	47·4 (45·0 to 49·9)	50·7 (47·9 to 53·4)	54·5 (51·2 to 57·6)	12·5 (8·3 to 16·4)*	3·6 (0·3 to 6·4)*	8·9 (5·6 to 12·4)*	1·01 (0·66 to 1·29)*	0·83 (0·07 to 1·47)*	1·12 (0·71 to 1·55)*
The Bahamas	56·1 (54·0 to 58·3)	58·7 (56·7 to 60·7)	63·4 (61·3 to 65·4)	64·8 (62·7 to 66·9)	66·2 (63·9 to 68·2)	66·4 (62·9 to 69·7)	10·3 (6·3 to 14·0)*	7·3 (4·7 to 9·8)*	3·0 (-0·7 to 6·4)	0·65 (0·40 to 0·88)*	1·22 (0·79 to 1·67)*	0·29 (-0·06 to 0·61)
Trinidad and Tobago	51·2 (49·7 to 52·6)	53·2 (51·6 to 54·6)	55·7 (53·7 to 57·3)	59·2 (57·0 to 61·0)	62·2 (59·5 to 64·6)	64·3 (60·7 to 67·5)	13·1 (9·0 to 16·6)*	4·5 (2·3 to 6·4)*	8·6 (5·3 to 11·8)*	0·87 (0·62 to 1·10)*	0·84 (0·43 to 1·18)*	0·89 (0·57 to 1·20)*
Virgin Islands	57·2 (54·6 to 60·4)	64·0 (61·2 to 67·2)	65·7 (63·0 to 68·8)	72·6 (69·8 to 75·9)	75·2 (72·2 to 78·7)	74·0 (70·0 to 79·1)	16·8 (11·9 to 21·9)*	8·5 (4·9 to 12·1)*	8·3 (4·0 to 13·2)*	0·99 (0·72 to 1·28)*	1·38 (0·80 to 1·96)*	0·75 (0·36 to 1·18)*
Central Latin America	43·3 (42·3 to 44·5)	51·1 (49·9 to 52·1)	55·8 (54·2 to 56·8)	58·6 (57·1 to 59·7)	61·0 (59·3 to 62·1)	64·4 (62·6 to 65·6)	21·1 (19·3 to 22·6)*	12·5 (10·8 to 13·7)*	8·6 (7·6 to 9·7)*	1·53 (1·40 to 1·63)*	2·54 (2·20 to 2·78)*	0·90 (0·79 to 1·01)*
Colombia	48·5 (46·7 to 50·6)	54·0 (52·6 to 55·6)	57·6 (55·9 to 59·0)	60·8 (59·0 to 62·2)	63·9 (62·2 to 65·5)	68·5 (65·8 to 70·9)	20·0 (16·6 to 23·0)*	9·1 (6·9 to 11·0)*	10·9 (8·3 to 13·4)*	1·33 (1·11 to 1·53)*	1·72 (1·29 to 2·11)*	1·09 (0·84 to 1·31)*
Costa Rica	60·7 (59·2 to 61·9)	62·4 (61·1 to 63·8)	64·7 (63·2 to 65·9)	67·9 (66·2 to 69·4)	70·8 (69·0 to 72·2)	73·7 (71·2 to 76·0)	13·0 (10·4 to 15·5)*	4·0 (2·5 to 5·5)*	9·0 (6·5 to 11·6)*	0·75 (0·60 to 0·88)*	0·64 (0·40 to 0·88)*	0·82 (0·60 to 1·04)*
El Salvador	38·1 (35·9 to 41·8)	44·2 (42·0 to 46·6)	52·1 (49·5 to 54·5)	56·5 (52·9 to 59·2)	59·4 (55·2 to 62·3)	63·2 (58·9 to 67·2)	25·1 (17·9 to 29·7)*	14·0 (8·5 to 17·2)*	11·1 (7·6 to 15·0)*	1·95 (1·38 to 2·27)*	3·14 (1·86 to 3·86)*	1·20 (0·84 to 1·60)*
Guatemala	30·4 (27·4 to 33·4)	35·2 (32·0 to 38·4)	42·0 (38·3 to 45·7)	46·5 (42·3 to 50·4)	48·7 (44·3 to 52·6)	51·5 (45·3 to 57·7)	21·1 (14·5 to 27·5)*	11·6 (7·1 to 16·1)*	9·4 (2·8 to 16·2)*	2·02 (1·42 to 2·57)*	3·24 (1·97 to 4·51)*	1·26 (0·38 to 2·10)*
Honduras	28·1 (24·8 to 31·3)	35·3 (31·1 to 39·2)	38·1 (33·1 to 43·3)	40·8 (35·1 to 47·2)	43·6 (37·6 to 50·3)	46·5 (40·1 to 53·1)	18·5 (11·4 to 25·5)*	10·0 (5·5 to 15·2)*	8·5 (2·1 to 15·1)*	1·94 (1·26 to 2·65)*	3·04 (1·73 to 4·52)*	1·25 (0·32 to 2·21)*
Mexico	45·5 (44·5 to 46·9)	54·5 (53·2 to 55·5)	59·0 (57·6 to 59·9)	61·4 (60·1 to 62·3)	63·0 (61·6 to 64·0)	66·3 (64·9 to 67·4)	20·8 (19·5 to 22·0)*	13·5 (12·0 to 14·6)*	7·3 (6·4 to 8·2)*	1·45 (1·34 to 1·54)*	2·61 (2·29 to 2·82)*	0·73 (0·64 to 0·82)*
Aguascalientes	49·2 (47·2 to 51·1)	57·3 (55·4 to 59·0)	60·6 (58·7 to 62·5)	62·5 (60·5 to 64·5)	64·1 (62·0 to 66·2)	67·8 (64·9 to 70·6)	18·6 (15·5 to 21·7)*	11·5 (9·0 to 14·0)*	7·1 (4·2 to 10·1)*	1·23 (1·04 to 1·43)*	2·10 (1·64 to 2·55)*	0·70 (0·41 to 0·98)*
Baja California	50·1 (48·2 to 52·1)	59·4 (57·1 to 61·2)	62·6 (59·8 to 64·5)	64·8 (62·3 to 66·7)	66·8 (64·3 to 68·9)	70·4 (67·4 to 73·3)	20·3 (17·1 to 23·5)*	12·5 (9·8 to 15·0)*	7·8 (5·1 to 10·9)*	1·31 (1·11 to 1·50)*	2·23 (1·75 to 2·69)*	0·73 (0·48 to 1·03)*
Baja California Sur	55·0 (52·7 to 57·3)	62·5 (59·9 to 64·7)	65·1 (62·7 to 67·1)	66·0 (63·7 to 68·0)	67·2 (64·9 to 69·4)	69·6 (66·1 to 73·0)	14·6 (10·5 to 18·2)*	10·1 (7·2 to 12·9)*	4·5 (0·6 to 8·2)*	0·90 (0·66 to 1·12)*	1·69 (1·20 to 2·17)*	0·41 (0·06 to 0·75)*
Campeche	46·4 (44·1 to 48·9)	55·0 (52·8 to 57·1)	60·4 (58·4 to 62·5)	63·3 (61·4 to 65·4)	64·6 (62·7 to 66·8)	67·4 (64·4 to 70·4)	21·0 (17·6 to 24·7)*	14·0 (11·1 to 17·1)*	7·0 (3·7 to 10·3)*	1·44 (1·19 to 1·67)*	2·64 (2·06 to 3·20)*	0·68 (0·37 to 0·99)*
Chiapas	35·1 (32·7 to 37·9)	43·2 (41·1 to 45·5)	47·8 (45·7 to 50·1)	50·3 (48·2 to 52·5)	52·7 (50·3 to 55·1)	55·8 (52·6 to 59·5)	20·8 (17·3 to 24·3)*	12·7 (9·9 to 15·5)*	8·0 (4·4 to 11·7)*	1·79 (1·49 to 2·11)*	3·09 (2·38 to 3·87)*	0·97 (0·54 to 1·40)*
Chihuahua	46·8 (44·3 to 48·9)	54·7 (51·9 to 57·0)	58·3 (55·3 to 60·7)	61·1 (58·2 to 63·5)	61·2 (57·8 to 64·1)	67·6 (63·8 to 71·0)	20·9 (16·9 to 24·9)*	11·5 (8·4 to 14·6)*	9·4 (5·8 to 12·9)*	1·42 (1·17 to 1·67)*	2·21 (1·62 to 2·78)*	0·93 (0·59 to 1·28)*
Coahuila	48·3 (46·3 to 50·1)	57·8 (55·6 to 59·7)	61·6 (59·5 to 63·4)	63·6 (61·4 to 65·4)	65·2 (63·1 to 67·0)	68·1 (65·1 to 70·8)	19·8 (16·7 to 22·7)*	13·3 (10·9 to 15·6)*	6·5 (3·6 to 9·3)*	1·32 (1·12 to 1·51)*	2·44 (2·00 to 2·86)*	0·62 (0·35 to 0·88)*
Colima	45·9 (43·5 to 48·3)	56·1 (53·9 to 58·3)	61·6 (59·3 to 63·8)	64·6 (62·2 to 66·8)	65·4 (63·0 to 67·6)	67·2 (64·0 to 70·3)	21·3 (17·7 to 25·1)*	15·6 (12·6 to 18·5)*	5·7 (2·1 to 9·2)*	1·46 (1·22 to 1·72)*	2·93 (2·34 to 3·49)*	0·55 (0·21 to 0·90)*
Distrito Federal	53·3 (51·4 to 55·2)	61·7 (59·8 to 63·5)	65·5 (63·6 to 67·2)	67·5 (65·6 to 69·3)	68·7 (66·6 to 70·4)	72·4 (69·8 to 74·9)	19·1 (16·4 to 21·7)*	12·2 (10·1 to 14·3)*	6·9 (4·0 to 9·6)*	1·18 (1·02 to 1·34)*	2·06 (1·70 to 2·42)*	0·62 (0·37 to 0·85)*
Durango	45·5 (43·1 to 47·6)	53·8 (51·0 to 56·0)	58·3 (55·7 to 60·5)	60·8 (58·3 to 63·0)	61·1 (58·2 to 63·7)	65·2 (61·5 to 68·4)	19·7 (16·1 to 23·5)*	12·7 (10·2 to 15·3)*	7·0 (3·3 to 10·6)*	1·38 (1·14 to 1·64)*	2·47 (1·98 to 2·98)*	0·71 (0·34 to 1·07)*
Guanajuato	46·1 (44·2 to 47·9)	53·9 (52·0 to 55·7)	58·2 (56·3 to 60·0)	60·6 (58·7 to 62·3)	62·3 (60·3 to 64·1)	65·2 (62·5 to 67·8)	19·1 (16·3 to 22·0)*	12·1 (10·0 to 14·4)*	7·0 (4·0 to 9·7)*	1·34 (1·14 to 1·54)*	2·34 (1·93 to 2·79)*	0·71 (0·40 to 0·98)*
Guerrero	42·3 (40·1 to 44·6)	47·1 (44·8 to 49·1)	51·3 (49·0 to 53·5)	54·7 (52·3 to 57·2)	55·6 (52·7 to 58·2)	60·0 (56·0 to 63·8)	17·7 (13·5 to 21·7)*	9·0 (6·3 to 11·9)*	8·6 (4·7 to 12·7)*	1·34 (1·04 to 1·64)*	1·94 (1·34 to 2·56)*	0·97 (0·53 to 1·40)*
Hidalgo	43·2 (41·2 to 45·0)	52·6 (50·7 to 54·4)	57·1 (55·2 to 58·9)	59·3 (57·5 to 60·9)	61·1 (59·3 to 62·8)	64·3 (61·8 to 66·8)	21·1 (18·1 to 24·1)*	13·9 (11·5 to 16·2)*	7·2 (4·5 to 9·9)*	1·53 (1·31 to 1·75)*	2·80 (2·32 to 3·28)*	0·74 (0·47 to 1·02)*
Jalisco	47·6 (45·5 to 49·7)	56·8 (54·4 to 58·6)	60·7 (58·3 to 62·6)	63·3 (61·0 to 65·2)	64·9 (62·6 to 66·8)	67·1 (64·0 to 69·9)	19·5 (16·0 to 22·6)*	13·1 (10·5 to 15·5)*	6·4 (3·3 to 9·3)*	1·32 (1·09 to 1·53)*	2·44 (1·95 to 2·89)*	0·62 (0·33 to 0·91)*
Mexico	47·7 (45·6 to 49·3)	57·4 (54·9 to 59·3)	60·9 (58·6 to 62·7)	63·4 (61·4 to 65·2)	65·2 (63·1 to 67·0)	68·2 (65·2 to 70·7)	20·5 (17·7 to 23·5)*	13·2 (11·0 to 15·4)*	7·3 (4·5 to 10·0)*	1·38 (1·19 to 1·57)*	2·45 (2·05 to 2·84)*	0·71 (0·44 to 0·97)*

Supplementary table 3. **Global, regional, national or territory, and select subnational estimates on the HAQ Index for 1990–2016, and absolute change and annualised rates of change for 1990–2016, 1990–2000, and 2000–2016.** Starred estimates of change represent statistically significant changes during a given time period. HAQ Index=Healthcare Access and Quality Index.

	HAQ Index						Absolute change			Annualised rate of change		
	1990	1995	2000	2005	2010	2016	1990-2016	1990-2000	2000-2016	1990-2016	1990-2000	2000-2016
Michoacan de Ocampo	44·6 (42·1 to 47·0)	52·7 (49·8 to 55·3)	57·5 (54·4 to 59·7)	60·1 (57·2 to 62·3)	61·7 (59·0 to 64·0)	64·9 (61·5 to 68·3)	20·3 (16·9 to 23·9)*	12·9 (10·1 to 15·5)*	7·4 (3·9 to 11·0)*	1·44 (1·21 to 1·67)*	2·54 (2·01 to 3·06)*	0·76 (0·40 to 1·12)*
Morelos	47·6 (45·0 to 49·9)	56·8 (54·3 to 59·0)	61·7 (59·3 to 63·7)	63·6 (61·3 to 65·6)	64·7 (62·2 to 66·9)	67·0 (63·5 to 70·1)	19·4 (15·8 to 22·9)*	14·0 (11·5 to 16·8)*	5·4 (1·8 to 8·7)*	1·32 (1·07 to 1·55)*	2·58 (2·12 to 3·13)*	0·52 (0·18 to 0·84)*
Nayarit	46·5 (44·5 to 48·5)	55·2 (52·7 to 57·0)	60·0 (57·4 to 62·0)	62·0 (59·6 to 64·0)	62·9 (60·4 to 65·1)	66·2 (63·0 to 68·9)	19·7 (16·6 to 22·5)*	13·4 (11·1 to 15·6)*	6·2 (3·5 to 9·1)*	1·36 (1·16 to 1·55)*	2·54 (2·10 to 2·95)*	0·62 (0·34 to 0·89)*
Nuevo Leon	56·0 (53·9 to 58·1)	63·1 (61·0 to 65·1)	66·6 (64·5 to 68·7)	68·5 (66·1 to 70·5)	69·3 (66·8 to 71·3)	72·8 (69·7 to 75·6)	16·7 (13·5 to 20·1)*	10·6 (8·2 to 13·1)*	6·1 (2·8 to 9·4)*	1·00 (0·82 to 1·20)*	1·73 (1·34 to 2·16)*	0·55 (0·25 to 0·83)*
Oaxaca	37·1 (35·1 to 39·3)	46·3 (44·4 to 48·2)	51·0 (49·0 to 52·8)	54·0 (52·2 to 55·8)	56·6 (54·7 to 58·6)	59·5 (56·8 to 62·1)	22·4 (19·3 to 25·4)*	13·9 (11·4 to 16·3)*	8·5 (5·6 to 11·7)*	1·81 (1·56 to 2·08)*	3·18 (2·56 to 3·76)*	0·96 (0·65 to 1·30)*
Puebla	39·7 (37·7 to 42·2)	49·3 (47·5 to 51·1)	54·1 (52·3 to 55·7)	57·2 (55·4 to 58·9)	59·9 (58·0 to 61·8)	62·7 (60·1 to 65·5)	23·0 (19·7 to 26·1)*	14·3 (11·7 to 16·8)*	8·7 (5·7 to 11·6)*	1·76 (1·50 to 1·99)*	3·08 (2·49 to 3·66)*	0·93 (0·61 to 1·23)*
Queretaro	45·7 (44·0 to 47·5)	55·8 (53·9 to 57·4)	60·4 (58·4 to 62·2)	62·6 (60·8 to 64·4)	64·0 (62·2 to 65·8)	67·0 (64·5 to 69·4)	21·3 (18·3 to 24·3)*	14·7 (12·3 to 17·0)*	6·6 (3·7 to 9·5)*	1·47 (1·27 to 1·67)*	2·79 (2·33 to 3·23)*	0·65 (0·37 to 0·93)*
Quintana Roo	49·0 (46·6 to 51·3)	57·0 (54·8 to 59·2)	61·2 (58·6 to 63·3)	63·0 (60·6 to 65·2)	64·0 (61·7 to 66·4)	67·1 (63·6 to 70·4)	18·1 (14·1 to 21·6)*	12·2 (9·0 to 15·2)*	5·9 (2·1 to 9·8)*	1·21 (0·96 to 1·45)*	2·22 (1·65 to 2·78)*	0·58 (0·21 to 0·96)*
San Luis Potosi	43·8 (41·8 to 45·7)	52·3 (50·3 to 54·2)	57·0 (54·6 to 58·9)	59·9 (58·0 to 61·7)	62·0 (60·0 to 64·0)	66·0 (63·3 to 68·5)	22·2 (19·1 to 25·2)*	13·2 (10·5 to 15·9)*	9·0 (5·8 to 12·0)*	1·58 (1·37 to 1·78)*	2·63 (2·11 to 3·20)*	0·92 (0·59 to 1·22)*
Sinaloa	52·0 (49·6 to 54·2)	58·8 (56·0 to 61·2)	64·1 (60·8 to 66·4)	66·4 (63·3 to 68·7)	67·7 (64·4 to 70·4)	71·6 (67·4 to 75·2)	19·6 (15·7 to 23·4)*	12·0 (9·1 to 14·7)*	7·5 (3·7 to 11·0)*	1·23 (1·00 to 1·45)*	2·08 (1·57 to 2·56)*	0·69 (0·35 to 1·02)*
Sonora	50·4 (48·1 to 52·5)	58·0 (55·5 to 60·1)	62·3 (59·6 to 64·4)	64·1 (61·3 to 66·3)	65·7 (63·3 to 67·9)	68·8 (65·3 to 72·0)	18·5 (14·7 to 22·2)*	11·9 (9·2 to 14·4)*	6·6 (3·0 to 10·4)*	1·20 (0·96 to 1·44)*	2·12 (1·64 to 2·56)*	0·63 (0·29 to 0·98)*
Tabasco	46·0 (44·0 to 48·1)	54·0 (51·6 to 56·0)	59·2 (56·5 to 61·3)	62·0 (59·3 to 64·1)	63·4 (61·1 to 65·4)	66·1 (63·0 to 69·0)	20·1 (16·6 to 23·4)*	13·2 (10·7 to 15·7)*	6·9 (3·7 to 10·1)*	1·39 (1·16 to 1·62)*	2·52 (2·05 to 3·02)*	0·69 (0·38 to 1·01)*
Tamaulipas	51·2 (49·2 to 52·9)	60·5 (58·2 to 62·4)	65·2 (62·7 to 67·1)	67·3 (64·8 to 69·2)	69·0 (66·7 to 71·0)	72·3 (69·2 to 75·1)	21·1 (18·1 to 24·2)*	14·1 (11·5 to 16·5)*	7·1 (4·3 to 9·9)*	1·33 (1·14 to 1·52)*	2·43 (2·02 to 2·87)*	0·64 (0·39 to 0·89)*
Tlaxcala	45·7 (43·8 to 47·7)	54·7 (53·0 to 56·5)	58·8 (57·0 to 60·6)	61·2 (59·6 to 63·0)	63·1 (61·3 to 64·9)	66·3 (63·6 to 69·2)	20·5 (17·5 to 23·7)*	13·1 (10·5 to 15·4)*	7·5 (4·4 to 10·7)*	1·43 (1·22 to 1·64)*	2·51 (2·03 to 2·94)*	0·75 (0·45 to 1·06)*
Veracruz de Ignacio de la Llave	43·0 (41·1 to 45·0)	51·6 (49·6 to 53·3)	56·3 (54·1 to 58·3)	59·4 (57·2 to 61·2)	61·0 (58·9 to 62·8)	63·8 (60·7 to 66·6)	20·8 (17·5 to 24·0)*	13·3 (10·7 to 15·7)*	7·5 (4·5 to 10·4)*	1·52 (1·28 to 1·75)*	2·70 (2·14 to 3·20)*	0·78 (0·47 to 1·08)*
Yucatan	46·4 (44·3 to 48·8)	55·4 (53·6 to 57·3)	60·1 (58·3 to 62·0)	62·6 (60·7 to 64·4)	63·6 (61·6 to 65·4)	65·6 (62·6 to 68·4)	19·2 (15·9 to 22·2)*	13·7 (11·0 to 16·2)*	5·5 (2·3 to 8·4)*	1·33 (1·11 to 1·55)*	2·58 (2·06 to 3·07)*	0·55 (0·23 to 0·83)*
Zacatecas	46·8 (44·9 to 48·6)	54·3 (52·3 to 56·1)	57·9 (55·9 to 59·5)	60·1 (58·2 to 61·8)	60·9 (58·8 to 62·8)	64·2 (61·4 to 66·9)	17·5 (14·6 to 20·4)*	11·1 (8·8 to 13·3)*	6·4 (3·5 to 9·2)*	1·22 (1·02 to 1·43)*	2·13 (1·68 to 2·58)*	0·65 (0·37 to 0·93)*
Nicaragua	43·1 (41·0 to 46·2)	45·1 (43·0 to 47·3)	49·8 (47·9 to 52·0)	53·7 (51·6 to 55·9)	57·5 (55·0 to 59·7)	61·2 (57·0 to 65·4)	18·1 (11·9 to 22·9)*	6·7 (3·1 to 9·6)*	11·4 (7·2 to 15·7)*	1·35 (0·88 to 1·67)*	1·45 (0·65 to 2·09)*	1·28 (0·83 to 1·74)*
Panama	52·1 (49·3 to 55·5)	57·0 (54·9 to 59·3)	60·8 (58·6 to 62·9)	62·2 (60·2 to 64·3)	63·6 (61·2 to 65·6)	68·3 (64·6 to 71·9)	16·1 (10·8 to 21·2)*	8·7 (5·0 to 12·0)*	7·4 (3·3 to 11·6)*	1·04 (0·69 to 1·36)*	1·55 (0·86 to 2·15)*	0·72 (0·33 to 1·11)*
Venezuela	51·3 (49·0 to 53·9)	56·2 (54·1 to 58·5)	60·0 (58·0 to 61·8)	63·0 (60·6 to 65·0)	65·9 (63·3 to 68·0)	67·8 (63·6 to 71·8)	16·5 (11·1 to 21·5)*	8·7 (5·4 to 11·6)*	7·8 (3·5 to 11·9)*	1·07 (0·74 to 1·38)*	1·57 (0·97 to 2·10)*	0·76 (0·35 to 1·15)*
Tropical Latin America	46·1 (44·9 to 47·2)	51·0 (49·6 to 52·1)	54·9 (53·6 to 55·9)	57·9 (56·5 to 58·9)	60·3 (59·0 to 61·3)	63·4 (62·0 to 64·4)	17·3 (16·1 to 18·5)*	8·9 (7·9 to 9·7)*	8·4 (7·3 to 9·6)*	1·23 (1·14 to 1·31)*	1·76 (1·57 to 1·94)*	0·89 (0·77 to 1·02)*
Brazil	46·5 (45·2 to 47·7)	51·4 (49·9 to 52·5)	55·3 (53·9 to 56·4)	58·3 (56·9 to 59·3)	60·7 (59·4 to 61·8)	63·8 (62·3 to 64·9)	17·3 (16·1 to 18·5)*	8·8 (8·0 to 9·6)*	8·5 (7·4 to 9·6)*	1·22 (1·13 to 1·30)*	1·74 (1·57 to 1·90)*	0·89 (0·78 to 1·02)*
Acre	42·2 (39·5 to 44·6)	45·6 (43·0 to 47·9)	48·9 (46·7 to 51·1)	50·9 (48·6 to 53·4)	53·5 (51·2 to 55·9)	57·2 (54·3 to 60·2)	15·0 (11·5 to 18·4)*	6·6 (4·0 to 9·5)*	8·4 (5·2 to 11·8)*	1·17 (0·90 to 1·45)*	1·46 (0·87 to 2·13)*	0·99 (0·62 to 1·37)*
Alagoas	38·0 (35·7 to 40·3)	43·2 (40·8 to 45·3)	47·8 (45·8 to 50·0)	50·6 (48·4 to 52·7)	53·3 (50·3 to 55·8)	56·6 (53·2 to 59·8)	18·6 (14·7 to 22·3)*	9·8 (7·2 to 12·8)*	8·8 (5·0 to 12·1)*	1·53 (1·21 to 1·84)*	2·30 (1·69 to 3·00)*	1·05 (0·61 to 1·44)*
Amapa	48·5 (46·3 to 50·9)	49·1 (46·8 to 51·4)	53·2 (51·0 to 55·7)	57·0 (54·8 to 59·3)	58·8 (56·4 to 61·1)	60·3 (57·2 to 63·3)	11·8 (8·4 to 15·1)*	4·6 (2·0 to 7·6)*	7·1 (3·7 to 10·5)*	0·84 (0·60 to 1·07)*	0·91 (0·38 to 1·50)*	0·79 (0·41 to 1·16)*
Amazonas	44·1 (41·8 to 46·1)	49·5 (47·3 to 51·5)	52·8 (50·6 to 54·9)	55·5 (53·4 to 57·6)	57·7 (55·4 to 59·7)	60·4 (57·5 to 63·4)	16·3 (12·9 to 19·4)*	8·6 (6·1 to 11·4)*	7·7 (4·4 to 10·9)*	1·21 (0·97 to 1·44)*	1·79 (1·25 to 2·39)*	0·85 (0·50 to 1·20)*
Bahia	43·4 (41·2 to 45·4)	48·0 (45·9 to 50·0)	50·9 (48·9 to 53·1)	53·2 (51·2 to 55·1)	55·1 (52·8 to 57·3)	58·8 (55·4 to 61·7)	15·4 (12·0 to 18·6)*	7·6 (5·1 to 9·9)*	7·8 (4·0 to 11·3)*	1·17 (0·91 to 1·40)*	1·61 (1·09 to 2·13)*	0·89 (0·47 to 1·28)*
Ceara	47·7 (45·0 to 50·3)	50·5 (48·2 to 52·6)	51·9 (49·7 to 54·1)	53·9 (51·8 to 55·8)	56·7 (54·6 to 58·7)	59·7 (56·9 to 62·5)	12·0 (8·2 to 15·6)*	4·2 (1·7 to 6·7)*	7·7 (4·2 to 11·1)*	0·86 (0·59 to 1·14)*	0·85 (0·35 to 1·38)*	0·87 (0·48 to 1·24)*
Distrito Federal	55·2 (53·0 to 57·2)	58·9 (56·7 to 60·9)	62·9 (60·8 to 64·9)	66·9 (64·7 to 68·8)	70·9 (68·6 to 73·0)	75·4 (72·3 to 78·1)	20·2 (16·7 to 23·2)*	7·7 (5·2 to 10·2)*	12·5 (9·2 to 15·5)*	1·20 (1·00 to 1·38)*	1·31 (0·88 to 1·73)*	1·13 (0·84 to 1·40)*
Espirito Santo	47·4 (45·2 to 49·6)	51·4 (48·9 to 53·7)	54·5 (52·1 to 56·7)	57·6 (55·0 to 59·7)	60·2 (57·6 to 62·5)	64·1 (60·8 to 67·1)	16·7 (13·3 to 20·2)*	7·1 (4·5 to 9·9)*	9·6 (6·1 to 13·1)*	1·16 (0·93 to 1·39)*	1·40 (0·89 to 1·95)*	1·01 (0·64 to 1·38)*
Goias	46·0 (43·9 to 48·1)	52·3 (50·2 to 54·1)	56·0 (53·8 to 57·8)	58·4 (56·4 to 60·2)	60·5 (58·1 to 62·4)	62·7 (60·0 to 65·5)	16·7 (13·6 to 19·6)*	9·9 (7·6 to 12·3)*	6·7 (3·7 to 9·7)*	1·19 (0·98 to 1·39)*	1·96 (1·49 to 2·44)*	0·71 (0·39 to 1·01)*
Maranhao	40·9 (38·4 to 43·7)	45·5 (42·9 to 48·4)	48·5 (45·9 to 51·6)	49·3 (47·1 to 52·3)	52·0 (49·6 to 54·8)	55·0 (51·8 to 58·6)	14·1 (10·6 to 17·7)*	7·7 (4·8 to 10·5)*	6·4 (2·6 to 10·0)*	1·14 (0·85 to 1·42)*	1·72 (1·11 to 2·34)*	0·78 (0·31 to 1·19)*
Mato Grosso	46·2 (44·0 to 48·3)	50·9 (48·6 to 53·0)	53·7 (51·6 to 55·8)	56·6 (54·6 to 58·5)	59·5 (57·2 to 61·7)	62·5 (59·4 to 65·7)	16·2 (12·9 to 19·8)*	7·5 (4·8 to 10·3)*	8·8 (5·4 to 12·2)*	1·16 (0·93 to 1·40)*	1·50 (0·96 to 2·06)*	0·94 (0·59 to 1·29)*
Mato Grosso do Sul	46·9 (44·8 to 48·8)	50·1 (47·9 to 52·3)	53·5 (51·5 to 55·6)	56·0 (53·8 to 58·1)	58·8 (56·5 to 60·9)	62·4 (59·6 to 65·2)	15·5 (12·3 to 18·7)*	6·7 (4·1 to 9·2)*	8·9 (5·5 to 11·9)*	1·10 (0·89 to 1·32)*	1·33 (0·82 to 1·85)*	0·96 (0·59 to 1·28)*
Minas Gerais	46·4 (44·4 to 48·3)	51·7 (49·8 to 53·6)	56·4 (54·4 to 58·2)	59·0 (56·9 to 60·8)	61·6 (59·5 to 63·5)	64·5 (61·8 to 67·2)	18·1 (15·0 to 21·2)*	9·9 (7·8 to 12·2)*	8·2 (5·2 to 11·3)*	1·27 (1·06 to 1·47)*	1·94 (1·52 to 2·39)*	0·85 (0·54 to 1·16)*
Para	43·7 (41·5 to 45·8)	49·2 (47·1 to 51·2)	51·2 (49·2 to 53·4)	52·3 (50·2 to 54·4)	54·5 (52·2 to 56·9)	57·8 (54·6 to 60·8)	14·1 (10·8 to 17·6)*	7·5 (4·9 to 10·0)*	6·6 (3·2 to 9·8)*	1·07 (0·83 to 1·33)*	1·58 (1·02 to 2·13)*	0·76 (0·37 to 1·12)*
Paraiba	46·5 (44·2 to 48·6)	49·9 (47·5 to 52·2)	54·3 (52·1 to 56·4)	56·5 (54·2 to 58·4)	57·7 (55·5 to 59·8)	60·4 (57·4 to 63·3)	13·9 (10·4 to 17·2)*	7·8 (5·4 to 10·3)*	6·1 (2·8 to 9·5)*	1·00 (0·76 to 1·24)*	1·54 (1·07 to 2·05)*	0·67 (0·31 to 1·02)*
Parana	48·2 (46·3 to 50·0)	52·6 (50·6 to 54·4)	56·2 (54·3 to 58·1)	59·5 (57·5 to 61·4)	62·1 (59·9 to 64·1)	65·3 (62·5 to 68·1)	17·2 (14·1 to 20·6)*	8·0 (5·9 to 10·6)*	9·1 (6·2 to 12·4)*	1·17 (0·96 to 1·40)*	1·54 (1·13 to 2·03)*	0·94 (0·64 to 1·25)*
Pernambuco	41·9 (39·3 to 44·2)	46·9 (44·2 to 49·1)	49·8 (47·3 to 51·9)	52·8 (50·3 to 55·1)	55·2 (52·3 to 57·8)	58·2 (54·7 to 61·2)	16·3 (12·4 to 19·8)*	7·9 (5·3 to 10·5)*	8·4 (5·0 to 11·8)*	1·26 (0·98 to 1·54)*	1·72 (1·15 to 2·32)*	0·97 (0·58 to 1·36)*
Piaui	48·5 (46·2 to 50·7)	52·7 (50·4 to 54·8)	53·0 (50·8 to 55·4)	53·0 (50·9 to 55·0)	55·2 (52·9 to 57·2)	57·8 (54·9 to 60·7)	9·3 (6·1 to 12·7)*	4·5 (1·9 to 7·0)*	4·8 (1·3 to 8·1)*	0·68 (0·45 to 0·93)*	0·89 (0·39 to 1·39)*	0·54 (0·15 to 0·92)*
Rio Grande do Norte	47·4 (45·1 to 49·3)	51·1 (48·7 to 53·3)	55·3 (53·1 to 57·1)	58·4 (56·5 to 60·4)	60·4 (57·9 to 62·9)	62·7 (60·0 to 65·8)	15·4 (12·0 to 18·9)*	7·9 (5·2 to 10·6)*	7·5 (4·1 to 11·0)*	1·08 (0·84 to 1·32)*	1·55 (1·01 to 2·07)*	0·79 (0·44 to 1·16)*
Rio Grande do Sul	52·7 (50·6 to 54·6)	56·7 (54·5 to 58·8)	60·6 (58·3 to 62·6)	63·6 (61·2 to 65·8)	66·0 (63·4 to 68·3)	68·1 (65·1 to 71·0)	15·4 (12·2 to 18·4)*	7·9 (5·5 to 10·3)*	7·5 (4·3 to 10·5)*	0·98 (0·79 to 1·16)*	1·39 (0·97 to 1·82)*	0·73 (0·43 to 1·02)*
Rio de Janeiro	49·2 (46·7 to 51·3)	53·7 (51·0 to 56·0)	58·1 (55·6 to 60·2)	61·0 (58·6 to 63·1)	63·0 (60·8 to 65·2)	65·1 (62·4 to 67·7)	15·8 (13·1 to 19·0)*	8·9 (6·5 to 11·3)*	6·9 (4·0 to 9·8)*	1·07 (0·88 to 1·28)*	1·66 (1·21 to 2·12)*	0·70 (0·41 to 1·00)*
Rondonia	42·5 (39·6 to 44·9)	48·8 (46·3 to 51·2)	52·5 (50·3 to 54·7)	55·9 (53·7 to 58·1)	58·7 (56·3 to 60·9)	62·2 (59·3 to 65·2)	19·7 (16·3 to 23·1)*	10·0 (7·1 to 12·9)*	9·7 (6·3 to 13·1)*	1·47 (1·21 to 1·73)*	2·11 (1·48 to 2·77)*	1·06 (0·70 to 1·40)*
Roraima	44·5 (42·2 to 46·6)	49·5 (47·4 to 51·4)	52·7 (50·8 to 54·7)	55·7 (53·7 to 57·4)	58·8 (56·7 to 60·8)	60·6 (58·2 to 63·1)	16·1 (13·1 to 19·2)*	8·2 (5·9 to 10·7)*	7·9 (5·3 to 10·6)*	1·19 (0·97 to 1·42)*	1·69 (1·19 to 2·25)*	0·87 (0·59 to 1·17)*
Santa Catarina	51·2 (49·1 to 53·4)	55·6 (53·4 to 57·9)	59·4 (57·3 to 61·4)	62·8 (60·7 to 64·9)	65·5 (63·5 to 67·6)	68·6 (65·5 to 71·6)	17·5 (14·0 to 20·8)*	8·2 (5·6 to 10·9)*	9·2 (5·7 to 12·5)*	1·13 (0·91 to 1·35)*	1·49 (1·00 to 1·97)*	0·90 (0·57 to 1·20)*
Sao Paulo	52·0 (49·8 to 53·9)	55·6 (53·4 to 57·3)	60·2 (58·0 to 62·0)	64·2 (62·2 to 65·9)	66·4 (64·5 to 68·4)	69·1 (66·6 to 71·5)	17·1 (14·5 to 19·9)*	8·2 (6·2 to 10·3)*	8·9 (6·3 to 11·8)*	1·09 (0·93 to 1·28)*	1·46 (1·10 to 1·85)*	0·86 (0·61 to 1·14)*
Sergipe	43·7 (41·4 to 46·0)	47·0 (44·8 to 49·0)	51·3 (49·1 to 53·5)	55·1 (52·9 to 57·2)	57·2 (55·0 to 59·3)	59·5 (56·6 to 62·4)	15·7 (12·2 to 19·0)*	7·5 (4·9 to 10·4)*	8·2 (4·9 to 11·5)*	1·18 (0·92 to 1·43)*	1·59 (1·03 to 2·19)*	0·93 (0·56 to 1·29)*
Tocantins	48·6 (45·5 to 51·4)	51·9 (49·7 to 54·2)	54·0 (51·8 to 56·4)	56·2 (53·8 to 58·7)	57·7 (55·3 to 60·0)	61·2 (58·2 to 64·1)	12·6 (9·3 to 16·4)*	5·4 (2·1 to 8·9)*	7·2 (3·4 to 10·6)*	0·89 (0·65 to 1·17)*	1·06 (0·42 to 1·77)*	0·78 (0·37 to 1·15)*
Paraguay	43·1 (41·1 to 45·1)	47·7 (45·3 to 50·1)	49·8 (46·8 to 52·3)	51·7 (48·5 to 54·2)	53·6 (50·3 to 56·2)	56·7 (53·1 to 60·2)	13·6 (9·9 to 17·4)*	6·8 (3·7 to 9·5)*	6·9 (3·4 to 10·3)*	1·06 (0·78 to 1·32)*	1·46 (0·82 to 2·07)*	0·81 (0·41 to 1·20)*
North Africa and Middle East	35·9 (33·7 to 37·9)	38·6 (36·6 to 40·4)	42·3 (40·5 to 44·0)	46·5 (44·9 to 48·2)	49·6 (48·1 to 51·2)	55·8 (54·0 to 57·8)	19·9 (17·6 to 22·2)*	6·4 (5·1 to 7·6)*	13·5 (11·6 to 15·5)*	1·70 (1·49 to 1·93)*	1·63 (1·29 to 2·00)*	1·73 (1·50 to 2·00)*
North Africa and Middle East	35·9 (33·7 to 38·0)	38·6 (36·6 to 40·5)	42·3 (40·5 to 44·0)	46·5 (45·0 to 48·2)	49·6 (48·1 to 51·2)	55·8 (54·0 to 57·8)	19·9 (17·6 to 22·2)*	6·4 (5·1 to 7·6)*	13·5 (11·6 to 15·5)*	1·70 (1·49 to 1·93)*	1·63 (1·29 to 2·00)*	1·73 (1·50 to 2·00)*
Afghanistan	15·8 (12·2 to 19·4)	14·6 (11·3 to 18·3)	14·9 (11·5 to 19·1)	16·9 (13·6 to 20·6)	21·3 (17·3 to 25·5)	25·9 (22·0 to 29·5)	10·1 (5·2 to 14·5)*	-0·9 (-4·1 to 2·7)	11·0 (6·4 to 15·4)*	1·93 (0·96 to 2·83)*	-0·60 (-2·75 to 1·68)	3·51 (1·88 to 5·10)*
Algeria	42·8 (37·6 to 46·7)	46·9 (42·0 to 50·5)	50·6 (46·1 to 54·2)	55·3 (51·2 to 58·5)	58·2 (54·5 to 61·1)	63·1 (59·4 to 66·4)	20·2 (16·0 to 24·6)*	7·8 (4·2 to 11·6)*	12·4 (8·7 to 16·7)*	1·49 (1·16 to 1·90)*	1·68 (0·89 to 2·56)*	1·38 (0·95 to 1·88)*
Bahrain	49·9 (46·7 to 53·1)	53·8 (50·3 to 57·0)	59·4 (56·3 to 62·2)	62·8 (59·7 to 65·9)	69·1 (66·0 to 72·2)	72·0 (67·3 to 76·5)	22·1 (16·5 to 27·2)*	9·5 (5·4 to 13·6)*	12·6 (7·3 to 17·9)*	1·41 (1·07 to 1·73)*	1·75 (0·98 to 2·51)*	1·20 (0·71 to 1·68)*
Egypt	34·2 (31·9 to 37·7)	39·5 (37·1 to 42·8)	45·9 (43·4 to 49·2)	49·9 (47·2 to 53·3)	53·4 (50·8 to 56·5)	58·0 (53·9 to 62·5)	23·8 (19·1 to 28·4)*	11·7 (8·8 to 14·5)*	12·1 (8·1 to 16·5)*	2·03 (1·64 to 2·39)*	2·94 (2·21 to 3·66)*	1·46 (1·00 to 1·95)*
Iran	49·3 (45·0 to 53·5)	55·6 (51·5 to 59·6)	61·0 (57·2 to 64·7)	65·4 (60·6 to 69·4)	68·3 (63·5 to 72·9)	71·8 (67·3 to 76·3)	22·4 (16·3 to 28·6)*	11·6 (6·4 to 16·7)*	10·8 (5·0 to 16·3)*	1·44 (1·04 to 1·87)*	2·12 (1·16 to 3·11)*	1·02 (0·47 to 1·54)*
Iraq	42·4 (38·5 to 47·1)	42·2 (38·4 to 46·6)	43·4 (40·0 to 46·8)	46·2 (42·6 to 50·2)	48·0 (43·5 to 52·8)	51·1 (45·9 to 56·6)	8·6 (1·2 to 15·8)*	0·9 (-3·8 to 5·6)	7·7 (1·6 to 13·7)*	0·71 (0·11 to 1·29)*	0·23 (-0·87 to 1·34)	1·02 (0·21 to 1·75)*
Jordan	50·0 (46·5 to 53·4)	53·6 (49·7 to 57·5)	58·3 (53·8 to 62·7)	60·1 (55·5 to 64·5)	66·2 (61·7 to 70·5)	70·2 (64·8 to 75·3)	20·2 (13·5 to 26·3)*	8·3 (4·0 to 13·0)*	11·9 (5·4 to 18·4)*	1·31 (0·88 to 1·70)*	1·54 (0·76 to 2·41)*	1·16 (0·54 to 1·82)*
Kuwait	66·8 (63·3 to 70·3)	67·4 (64·8 to 70·2)	70·8 (68·3 to 73·5)	71·8 (69·1 to 74·6)	75·7 (73·0 to 78·3)	80·7 (75·5 to 86·1)	13·8 (7·8 to 19·7)*	4·0 (-0·4 to 8·4)	9·9 (4·4 to 15·4)*	0·72 (0·42 to 1·02)*	0·58 (-0·05 to 1·22)	0·81 (0·37 to 1·25)*
Lebanon	53·1 (48·5 to 57·1)	59·8 (56·0 to 63·4)	67·2 (63·6 to 70·6)	74·0 (70·9 to 77·0)	81·3 (78·3 to 84·1)	85·6 (82·8 to 88·2)	32·5 (27·5 to 38·0)*	14·1 (9·8 to 18·5)*	18·4 (14·2 to 23·0)*	1·84 (1·52 to 2·21)*	2·36 (1·60 to 3·19)*	1·52 (1·15 to 1·93)*
Libya	50·9 (46·8 to 54·5)	54·7 (51·2 to 57·9)	57·9 (54·5 to 61·0)	62·6 (59·2 to 65·9)	69·9 (66·6 to 72·9)	71·1 (67·4 to 74·6)	20·2 (15·7 to 24·7)*	7·0 (4·1 to 9·9)*	13·2 (9·5 to 16·8)*	1·29 (1·00 to 1·60)*	1·30 (0·74 to 1·87)*	1·28 (0·93 to 1·65)*
Morocco	37·5 (34·7 to 40·7)	40·6 (37·6 to 43·6)	44·6 (41·5 to 47·5)	47·6 (44·5 to 50·7)	52·0 (48·7 to 55·3)	57·6 (54·5 to 60·8)	20·1 (16·2 to 23·6)*	7·1 (4·1 to 10·0)*	13·0 (9·9 to 16·1)*	1·65 (1·33 to 1·95)*	1·73 (0·99 to 2·45)*	1·60 (1·23 to 2·00)*
Oman	52·5 (49·6 to 55·5)	59·0 (56·3 to 61·8)	63·4 (61·1 to 65·9)	68·4 (66·4 to 70·5)	70·6 (68·4 to 72·9)	76·2 (74·0 to 78·6)	23·7 (20·4 to 27·1)*	10·9 (8·4 to 13·6)*	12·8 (10·0 to 15·4)*	1·43 (1·21 to 1·67)*	1·89 (1·44 to 2·38)*	1·15 (0·89 to 1·38)*
Palestine	48·1 (43·1 to 53·5)	51·9 (48·4 to 56·1)	54·1 (51·2 to 57·6)	56·0 (53·3 to 58·9)	56·1 (53·6 to 58·9)	57·4 (54·1 to 60·6)	9·3 (2·7 to 15·3)*	6·0 (0·4 to 11·7)*	3·3 (-1·0 to 7·2)	0·68 (0·20 to 1·15)*	1·19 (0·07 to 2·37)*	0·37 (-0·11 to 0·80)
Qatar	57·7 (53·3 to 62·2)	59·6 (55·4 to 63·4)	64·6 (60·3 to 69·1)	72·2 (68·4 to 76·2)	78·0 (74·6 to 81·3)	81·7 (75·9 to 86·6)	23·9 (16·8 to 30·8)*	6·9 (1·0 to 12·9)*	17·0 (10·4 to 24·0)*	1·33 (0·94 to 1·74)*	1·13 (0·16 to 2·11)*	1·46 (0·89 to 2·03)*
Saudi Arabia	49·9 (47·0 to 53·0)	53·3 (50·7 to 56·0)	56·6 (54·8 to 58·7)	61·2 (59·2 to 63·1)	68·1 (66·0 to 70·0)	77·1 (74·9 to 79·3)	27·2 (23·4 to 31·2)*	6·7 (3·9 to 9·7)*	20·5 (17·8 to 23·2)*	1·67 (1·42 to 1·95)*	1·26 (0·72 to 1·85)*	1·93 (1·69 to 2·18)*
Sudan	28·6 (24·3 to 31·8)	31·1 (27·0 to 34·3)	33·7 (29·8 to 36·7)	37·3 (33·5 to 40·5)	40·5 (36·2 to 44·0)	45·8 (41·0 to 50·0)	17·2 (13·1 to 21·3)*	5·1 (2·5 to 8·0)*	12·1 (8·0 to 16·0)*	1·81 (1·37 to 2·28)*	1·65 (0·76 to 2·66)*	1·91 (1·31 to 2·51)*
Syria	45·5 (42·6 to 48·3)	50·2 (47·8 to 53·0)	56·7 (54·6 to 58·8)	59·6 (57·6 to 61·5)	64·1 (62·0 to 66·4)	67·2 (64·4 to 70·2)	21·7 (17·9 to 25·7)*	11·2 (8·1 to 14·5)*	10·5 (7·1 to 14·0)*	1·50 (1·23 to 1·79)*	2·21 (1·58 to 2·94)*	1·06 (0·74 to 1·41)*
Tunisia	47·6 (43·2 to 50·9)	52·6 (48·3 to 55·7)	59·0 (55·3 to 62·3)	61·4 (57·6 to 65·1)	64·7 (60·7 to 68·7)	69·4 (65·4 to 73·7)	21·8 (17·1 to 26·8)*	11·4 (8·1 to 14·7)*	10·4 (6·6 to 14·3)*	1·45 (1·14 to 1·83)*	2·15 (1·50 to 2·85)*	1·02 (0·64 to 1·40)*
Turkey	42·5 (38·8 to 46·3)	46·0 (42·5 to 49·7)	53·9 (50·8 to 56·8)	63·4 (60·8 to 66·0)	69·8 (66·9 to 72·8)	74·4 (70·0 to 78·4)	31·9 (26·2 to 37·3)*	11·4 (7·9 to 15·2)*	20·4 (15·5 to 25·2)*	2·16 (1·76 to 2·53)*	2·39 (1·61 to 3·22)*	2·01 (1·53 to 2·50)*
United Arab Emirates	49·8 (43·7 to 55·4)	54·4 (49·5 to 59·4)	60·2 (56·0 to 64·4)	66·2 (63·2 to 68·9)	66·9 (63·0 to 71·2)	70·3 (65·5 to 75·4)	20·5 (12·8 to 28·6)*	10·4 (5·2 to 15·8)*	10·1 (4·1 to 16·6)*	1·33 (0·81 to 1·90)*	1·91 (0·93 to 3·00)*	0·97 (0·39 to 1·59)*
Yemen	25·2 (20·8 to 29·1)	28·3 (23·9 to 32·4)	31·4 (26·9 to 35·6)	35·0 (30·6 to 39·1)	39·7 (35·3 to 43·8)	43·3 (38·3 to 47·9)	18·1 (12·9 to 22·7)*	6·2 (2·6 to 9·9)*	11·9 (7·5 to 16·2)*	2·09 (1·45 to 2·72)*	2·20 (0·92 to 3·57)*	2·01 (1·22 to 2·75)*
South Asia	23·8 (22·3 to 25·6)	25·7 (24·2 to 27·5)	27·6 (26·1 to 29·3)	30·7 (29·2 to 32·4)	34·8 (33·2 to 36·3)	40·4 (38·7 to 42·2)	16·6 (14·0 to 18·9)*	3·8 (2·1 to 5·2)*	12·9 (10·9 to 14·8)*	2·04 (1·70 to 2·32)*	1·47 (0·84 to 2·09)*	2·39 (2·01 to 2·77)*

Supplementary table 3. **Global, regional, national or territory, and select subnational estimates on the HAQ Index for 1990–2016, and absolute change and annualised rates of change for 1990–2016, 1990–2000, and 2000–2016.** Starred estimates of change represent statistically significant changes during a given time period. HAQ Index=Healthcare Access and Quality Index.

	HAQ Index						Absolute change			Annualised rate of change		
	1990	1995	2000	2005	2010	2016	1990-2016	1990-2000	2000-2016	1990-2016	1990-2000	2000-2016
South Asia	23·8 (22·3 to 25·6)	25·7 (24·2 to 27·5)	27·6 (26·1 to 29·3)	30·7 (29·2 to 32·4)	34·8 (33·2 to 36·3)	40·4 (38·7 to 42·2)	16·6 (14·0 to 18·9)*	3·8 (2·1 to 5·2)*	12·9 (10·9 to 14·8)*	2·04 (1·70 to 2·32)*	1·47 (0·84 to 2·09)*	2·39 (2·01 to 2·77)*
Bangladesh	17·8 (15·0 to 20·7)	22·1 (19·5 to 24·7)	27·5 (25·2 to 30·0)	34·1 (31·7 to 36·4)	41·0 (38·5 to 43·5)	47·6 (44·3 to 50·9)	29·8 (25·7 to 34·2)*	9·7 (6·5 to 12·8)*	20·1 (16·3 to 23·8)*	3·80 (3·18 to 4·50)*	4·36 (2·84 to 6·05)*	3·44 (2·78 to 4·09)*
Bhutan	20·0 (16·2 to 23·9)	23·8 (20·4 to 27·3)	29·6 (26·1 to 33·1)	34·5 (31·2 to 38·4)	41·6 (37·7 to 45·9)	47·3 (42·6 to 52·0)	27·2 (22·1 to 32·6)*	9·6 (5·7 to 13·5)*	17·7 (13·1 to 22·3)*	3·32 (2·58 to 4·11)*	3·94 (2·29 to 5·80)*	2·93 (2·18 to 3·70)*
India	24·7 (22·9 to 27·2)	26·6 (24·9 to 28·7)	28·0 (26·3 to 30·3)	31·1 (29·3 to 33·1)	35·2 (33·4 to 37·2)	41·2 (39·1 to 43·4)	16·5 (13·4 to 19·4)*	3·3 (1·3 to 5·5)*	13·2 (10·7 to 15·6)*	1·97 (1·56 to 2·31)*	1·27 (0·46 to 2·03)*	2·41 (1·93 to 2·85)*
Andhra Pradesh	27·0 (22·6 to 31·0)	29·0 (24·9 to 33·2)	30·9 (26·8 to 35·3)	34·9 (30·4 to 39·4)	40·0 (35·6 to 44·3)	46·5 (41·7 to 51·0)	19·5 (13·8 to 25·1)*	3·9 (-0·0 to 7·8)	15·6 (10·7 to 20·9)*	2·10 (1·50 to 2·77)*	1·36 (-0·02 to 2·80)	2·56 (1·72 to 3·45)*
Arunachal Pradesh	27·7 (23·1 to 32·8)	29·1 (24·7 to 34·0)	30·9 (26·5 to 35·9)	34·2 (29·7 to 38·7)	38·1 (33·8 to 42·5)	44·3 (39·7 to 48·6)	16·6 (9·8 to 23·0)*	3·1 (-1·5 to 7·7)	13·4 (7·0 to 18·8)*	1·81 (1·05 to 2·63)*	1·08 (-0·52 to 2·72)	2·27 (1·15 to 3·24)*
Assam	19·9 (16·5 to 23·7)	21·5 (18·0 to 25·3)	21·9 (18·8 to 25·4)	24·7 (21·6 to 28·2)	28·0 (24·8 to 31·4)	34·0 (30·3 to 38·1)	14·1 (9·2 to 19·3)*	2·0 (-1·4 to 5·7)	12·1 (7·5 to 17·1)*	2·07 (1·29 to 2·85)*	0·97 (-0·67 to 2·77)	2·75 (1·68 to 3·87)*
Bihar	23·3 (19·7 to 27·2)	23·7 (20·2 to 27·2)	24·0 (20·7 to 27·7)	26·4 (23·0 to 30·3)	31·2 (27·5 to 35·1)	37·0 (32·7 to 41·3)	13·7 (8·0 to 19·2)*	0·7 (-3·0 to 4·6)	13·0 (8·0 to 17·7)*	1·79 (1·02 to 2·53)*	0·31 (-1·29 to 1·93)	2·71 (1·66 to 3·68)*
Chhattisgarh	23·6 (19·5 to 27·9)	25·1 (21·3 to 28·9)	26·5 (23·1 to 30·8)	28·4 (25·1 to 32·1)	31·5 (28·2 to 35·1)	37·4 (33·7 to 41·3)	13·8 (8·4 to 19·0)*	2·9 (-0·9 to 6·8)	10·9 (6·0 to 15·3)*	1·79 (1·04 to 2·55)*	1·18 (-0·33 to 2·80)	2·16 (1·18 to 3·08)*
Delhi	32·5 (29·0 to 36·0)	34·8 (31·3 to 38·4)	38·2 (34·6 to 42·1)	41·6 (37·9 to 45·5)	47·2 (43·4 to 51·1)	56·2 (52·0 to 60·5)	23·8 (18·6 to 29·1)*	5·8 (1·7 to 9·6)*	18·0 (12·9 to 23·0)*	2·12 (1·64 to 2·61)*	1·64 (0·46 to 2·68)*	2·42 (1·70 to 3·11)*
Goa	38·0 (33·6 to 42·6)	40·8 (36·5 to 45·4)	45·4 (41·1 to 49·6)	51·3 (46·5 to 55·9)	57·3 (51·8 to 61·5)	64·8 (59·6 to 68·8)	26·8 (20·5 to 32·5)*	7·3 (2·7 to 11·8)*	19·4 (13·7 to 24·7)*	2·06 (1·54 to 2·56)*	1·77 (0·65 to 2·87)*	2·23 (1·59 to 2·90)*
Gujarat	28·6 (25·6 to 32·1)	30·9 (27·8 to 34·0)	33·1 (29·9 to 36·2)	35·7 (32·4 to 39·2)	39·7 (36·2 to 42·9)	45·0 (41·0 to 48·6)	16·4 (11·8 to 20·9)*	4·4 (1·1 to 8·0)*	12·0 (7·9 to 15·9)*	1·74 (1·25 to 2·24)*	1·44 (0·34 to 2·60)*	1·93 (1·28 to 2·55)*
Haryana	31·3 (28·0 to 35·3)	33·3 (29·9 to 36·8)	33·4 (30·3 to 37·0)	35·8 (32·9 to 39·1)	38·9 (35·8 to 42·0)	45·0 (41·5 to 48·5)	13·6 (8·6 to 18·6)*	2·1 (-1·5 to 5·8)	11·5 (7·1 to 15·7)*	1·39 (0·86 to 1·90)*	0·66 (-0·47 to 1·85)	1·85 (1·14 to 2·49)*
Himachal Pradesh	32·2 (28·1 to 36·7)	34·9 (30·9 to 38·9)	38·0 (34·4 to 41·9)	41·2 (37·9 to 44·7)	45·2 (42·0 to 48·5)	51·7 (48·2 to 55·5)	19·5 (14·3 to 24·7)*	5·8 (1·9 to 10·0)*	13·7 (9·2 to 18·1)*	1·83 (1·30 to 2·37)*	1·68 (0·52 to 2·91)*	1·92 (1·26 to 2·57)*
Jammu and Kashmir	28·2 (24·8 to 32·3)	29·8 (26·4 to 33·5)	32·5 (29·2 to 36·2)	36·3 (32·8 to 39·9)	40·2 (36·6 to 43·9)	46·7 (42·9 to 50·8)	18·5 (13·6 to 23·4)*	4·2 (0·6 to 7·7)*	14·3 (9·8 to 18·9)*	1·94 (1·37 to 2·49)*	1·41 (0·18 to 2·62)*	2·28 (1·56 to 3·03)*
Jharkhand	21·3 (17·3 to 25·7)	21·8 (18·0 to 25·8)	23·3 (19·4 to 27·7)	27·4 (23·8 to 31·2)	31·5 (27·9 to 35·5)	37·4 (33·4 to 41·6)	16·2 (10·9 to 21·6)*	2·0 (-2·2 to 6·2)	14·2 (9·1 to 18·9)*	2·19 (1·42 to 3·01)*	0·92 (-0·94 to 2·76)	2·98 (1·89 to 4·08)*
Karnataka	29·9 (25·9 to 34·3)	32·3 (28·1 to 36·5)	33·9 (29·6 to 38·0)	37·4 (33·1 to 41·6)	41·1 (36·9 to 44·8)	46·6 (42·3 to 50·2)	16·7 (11·9 to 21·6)*	3·9 (0·3 to 7·3)*	12·8 (8·3 to 17·4)*	1·71 (1·18 to 2·26)*	1·24 (0·10 to 2·28)*	2·01 (1·28 to 2·75)*
Kerala	43·1 (38·8 to 47·1)	47·3 (43·0 to 51·4)	51·0 (46·8 to 55·0)	55·3 (50·3 to 58·8)	58·6 (53·8 to 61·6)	63·9 (58·6 to 67·0)	20·8 (16·5 to 25·0)*	7·9 (4·5 to 11·4)*	12·9 (9·2 to 16·4)*	1·51 (1·19 to 1·85)*	1·68 (0·94 to 2·44)*	1·41 (0·98 to 1·81)*
Madhya Pradesh	23·3 (20·1 to 26·9)	25·2 (22·0 to 28·8)	26·5 (23·4 to 30·2)	29·0 (25·7 to 32·9)	33·0 (29·8 to 36·6)	39·5 (35·8 to 43·1)	16·2 (11·5 to 20·7)*	3·2 (-0·3 to 6·8)	13·0 (8·6 to 17·1)*	2·04 (1·41 to 2·67)*	1·31 (-0·12 to 2·87)	2·49 (1·65 to 3·30)*
Maharashtra	30·5 (27·4 to 33·9)	33·0 (29·8 to 36·3)	34·9 (31·8 to 38·3)	38·6 (35·2 to 42·1)	43·6 (40·4 to 46·8)	49·8 (46·2 to 53·3)	19·3 (15·3 to 24·0)*	4·4 (1·0 to 8·0)*	14·9 (10·7 to 19·0)*	1·89 (1·46 to 2·34)*	1·36 (0·30 to 2·46)*	2·22 (1·56 to 2·88)*
Manipur	30·1 (25·2 to 35·1)	31·8 (27·1 to 36·9)	33·3 (28·7 to 37·9)	36·3 (31·8 to 41·2)	39·2 (34·5 to 43·8)	44·2 (39·5 to 48·7)	14·1 (8·2 to 20·0)*	3·3 (-0·8 to 7·8)	10·9 (5·7 to 15·9)*	1·49 (0·84 to 2·19)*	1·04 (-0·26 to 2·50)	1·77 (0·90 to 2·68)*
Meghalaya	25·9 (21·5 to 31·1)	27·1 (22·8 to 32·0)	28·1 (23·7 to 32·6)	31·2 (26·8 to 35·8)	35·0 (30·5 to 39·7)	39·6 (35·0 to 44·4)	13·7 (7·0 to 20·0)*	2·2 (-2·2 to 6·5)	11·5 (6·1 to 16·8)*	1·64 (0·84 to 2·41)*	0·83 (-0·78 to 2·42)	2·15 (1·14 to 3·28)*
Mizoram	33·4 (28·5 to 38·6)	34·9 (30·3 to 39·8)	36·7 (32·1 to 41·9)	39·9 (35·2 to 44·4)	43·1 (38·5 to 47·7)	48·9 (44·0 to 53·6)	15·5 (9·5 to 21·6)*	3·3 (-0·8 to 7·2)	12·2 (7·0 to 17·4)*	1·47 (0·89 to 2·08)*	0·96 (-0·24 to 2·09)	1·79 (0·98 to 2·55)*
Nagaland	29·4 (25·1 to 34·2)	30·4 (25·9 to 34·8)	31·4 (26·9 to 36·0)	35·9 (31·4 to 40·9)	40·3 (35·6 to 45·1)	46·1 (41·3 to 50·9)	16·7 (10·2 to 22·9)*	2·0 (-2·4 to 6·5)	14·7 (9·4 to 20·2)*	1·74 (1·06 to 2·43)*	0·66 (-0·77 to 2·19)	2·41 (1·49 to 3·40)*
Odisha	19·7 (16·3 to 23·6)	21·4 (17·9 to 25·3)	21·9 (18·7 to 25·5)	24·6 (21·1 to 28·6)	29·2 (25·9 to 33·0)	36·3 (32·4 to 40·0)	16·6 (11·5 to 21·3)*	2·2 (-1·5 to 5·8)	14·4 (9·6 to 18·9)*	2·36 (1·58 to 3·15)*	1·07 (-0·68 to 2·89)	3·16 (2·09 to 4·21)*
Punjab	32·6 (29·4 to 36·1)	34·7 (31·6 to 38·0)	35·8 (32·7 to 39·0)	39·0 (35·7 to 42·2)	43·2 (39·8 to 46·5)	49·5 (45·4 to 53·4)	16·9 (11·9 to 21·8)*	3·2 (-0·3 to 6·8)	13·7 (9·3 to 17·8)*	1·61 (1·13 to 2·09)*	0·95 (-0·09 to 2·00)	2·02 (1·40 to 2·65)*
Rajasthan	25·9 (22·6 to 29·7)	27·9 (24·4 to 31·5)	29·7 (26·1 to 33·6)	33·0 (29·0 to 36·7)	36·3 (32·2 to 40·3)	40·7 (36·2 to 44·8)	14·8 (9·9 to 19·2)*	3·7 (0·3 to 7·3)*	11·1 (6·5 to 15·5)*	1·74 (1·16 to 2·29)*	1·35 (0·12 to 2·59)*	1·98 (1·18 to 2·77)*
Sikkim	28·5 (24·3 to 33·1)	30·3 (25·9 to 34·9)	32·3 (28·1 to 36·6)	36·4 (32·5 to 40·6)	41·9 (37·7 to 45·9)	50·5 (45·8 to 54·8)	22·0 (16·0 to 27·4)*	3·8 (-0·4 to 7·9)	18·2 (13·1 to 23·0)*	2·21 (1·60 to 2·82)*	1·26 (-0·12 to 2·65)	2·80 (1·99 to 3·58)*
Tamil Nadu	30·2 (26·4 to 34·2)	33·5 (29·8 to 37·1)	36·0 (32·4 to 39·7)	39·5 (36·1 to 43·0)	44·4 (40·9 to 47·7)	51·2 (47·4 to 54·8)	21·0 (16·3 to 25·6)*	5·9 (2·1 to 9·5)*	15·1 (10·9 to 19·5)*	2·04 (1·54 to 2·51)*	1·79 (0·62 to 2·91)*	2·19 (1·55 to 2·85)*
Telangana	28·0 (23·7 to 32·5)	30·2 (25·8 to 34·6)	32·0 (27·5 to 36·8)	35·5 (30·6 to 40·3)	41·0 (35·9 to 45·5)	48·5 (43·6 to 53·2)	20·6 (14·7 to 26·3)*	4·1 (0·2 to 8·3)*	16·5 (11·6 to 21·7)*	2·13 (1·49 to 2·77)*	1·36 (0·08 to 2·82)*	2·61 (1·78 to 3·53)*
Tripura	28·1 (23·3 to 32·9)	29·7 (25·4 to 34·3)	32·0 (27·7 to 36·5)	35·3 (31·0 to 39·8)	37·8 (33·2 to 42·3)	42·3 (37·6 to 46·8)	14·2 (8·3 to 20·2)*	4·0 (-0·0 to 8·1)	10·2 (5·0 to 15·3)*	1·58 (0·91 to 2·34)*	1·33 (-0·01 to 2·78)	1·74 (0·83 to 2·58)*
Union Territories other than Delhi	36·7 (33·0 to 41·0)	38·2 (34·2 to 42·3)	40·5 (36·6 to 44·5)	44·4 (40·1 to 49·1)	48·2 (43·7 to 53·1)	54·3 (48·9 to 59·7)	17·6 (11·5 to 23·3)*	3·8 (-0·2 to 8·1)	13·8 (8·5 to 19·3)*	1·51 (0·98 to 1·98)*	0·98 (-0·05 to 2·14)	1·84 (1·14 to 2·54)*
Uttar Pradesh	21·1 (17·8 to 25·0)	22·7 (19·7 to 25·9)	24·1 (21·0 to 27·5)	27·1 (23·9 to 30·3)	30·1 (26·8 to 33·5)	34·9 (31·1 to 38·4)	13·7 (8·4 to 18·4)*	3·0 (-0·8 to 6·5)	10·7 (6·3 to 14·7)*	1·93 (1·16 to 2·65)*	1·33 (-0·31 to 2·89)	2·31 (1·37 to 3·20)*
Uttarakhand	25·3 (21·8 to 29·1)	26·5 (23·1 to 30·2)	27·4 (24·0 to 31·1)	30·0 (26·4 to 33·8)	35·2 (31·6 to 38·9)	43·2 (39·2 to 47·2)	17·9 (12·5 to 23·2)*	2·0 (-2·0 to 5·9)	15·9 (11·5 to 20·5)*	2·06 (1·40 to 2·72)*	0·78 (-0·71 to 2·27)	2·87 (2·00 to 3·73)*
West Bengal	26·6 (23·3 to 30·4)	28·4 (25·4 to 31·8)	31·1 (28·0 to 34·4)	35·6 (32·5 to 38·8)	40·6 (37·4 to 43·9)	47·1 (43·4 to 50·6)	20·5 (15·7 to 25·4)*	4·5 (0·9 to 7·8)*	16·0 (11·7 to 20·3)*	2·20 (1·65 to 2·79)*	1·57 (0·31 to 2·78)*	2·59 (1·91 to 3·30)*
Nepal	21·0 (18·1 to 24·1)	23·4 (20·6 to 26·4)	26·5 (23·7 to 29·4)	30·7 (27·5 to 34·0)	34·8 (31·3 to 38·8)	40·0 (36·5 to 44·4)	19·1 (14·6 to 23·9)*	5·5 (2·5 to 8·5)*	13·6 (10·0 to 17·6)*	2·49 (1·90 to 3·14)*	2·33 (1·05 to 3·69)*	2·59 (1·94 to 3·31)*
Pakistan	26·8 (24·0 to 30·0)	25·9 (23·0 to 29·1)	27·4 (24·9 to 30·5)	29·4 (26·6 to 32·5)	33·2 (29·8 to 36·8)	37·6 (33·7 to 41·9)	10·8 (6·1 to 15·5)*	0·6 (-2·4 to 3·5)	10·2 (5·7 to 14·6)*	1·30 (0·73 to 1·86)*	0·22 (-0·86 to 1·32)	1·98 (1·11 to 2·77)*
Sub-Saharan Africa	19·6 (18·2 to 21·1)	20·7 (19·4 to 22·2)	22·3 (20·9 to 23·8)	25·7 (24·3 to 27·3)	28·9 (27·5 to 30·6)	31·9 (30·5 to 33·7)	12·3 (10·5 to 14·1)*	2·7 (1·4 to 4·1)*	9·6 (8·0 to 11·3)*	1·88 (1·58 to 2·17)*	1·30 (0·65 to 1·96)*	2·24 (1·85 to 2·65)*
Central Sub-Saharan Africa	19·6 (16·6 to 22·9)	19·9 (16·5 to 23·5)	20·6 (17·4 to 24·2)	23·7 (20·3 to 27·2)	25·7 (22·4 to 29·1)	29·2 (25·8 to 32·7)	9·7 (6·0 to 13·1)*	1·1 (-1·7 to 3·8)	8·6 (5·2 to 11·8)*	1·55 (0·96 to 2·19)*	0·54 (-0·86 to 1·87)	2·18 (1·26 to 3·11)*
Angola	18·4 (12·7 to 24·4)	19·1 (12·9 to 25·6)	20·6 (14·2 to 27·2)	23·4 (16·8 to 30·1)	26·9 (20·0 to 33·8)	33·4 (25·5 to 40·4)	14·9 (7·2 to 22·6)*	2·2 (-2·6 to 6·9)	12·8 (6·1 to 19·7)*	2·31 (1·09 to 3·64)*	1·11 (-1·26 to 3·57)	3·06 (1·34 to 4·95)*
Central African Republic	15·8 (12·7 to 19·6)	16·0 (11·8 to 20·6)	16·1 (11·2 to 22·1)	17·4 (12·1 to 23·5)	18·7 (13·5 to 24·7)	18·6 (13·1 to 24·4)	2·7 (-3·2 to 8·9)	0·3 (-4·7 to 5·4)	2·4 (-3·6 to 8·6)	0·59 (-0·80 to 1·85)	0·10 (-3·25 to 2·99)	0·89 (-1·33 to 3·19)
Congo (Brazzaville)	21·0 (17·0 to 25·1)	20·4 (16·6 to 24·2)	21·9 (18·0 to 25·9)	27·6 (23·4 to 31·8)	29·6 (25·0 to 34·5)	34·1 (28·4 to 40·4)	13·0 (6·7 to 20·0)*	0·8 (-3·3 to 5·2)	12·2 (6·4 to 18·6)*	1·86 (0·96 to 2·84)*	0·40 (-1·49 to 2·44)	2·77 (1·49 to 4·19)*
Democratic Republic of the Congo	21·7 (17·6 to 26·4)	21·7 (17·4 to 26·4)	22·1 (17·5 to 27·0)	24·9 (20·8 to 29·2)	26·2 (22·2 to 30·7)	29·6 (25·7 to 33·7)	7·9 (2·8 to 12·7)*	0·4 (-3·6 to 4·4)	7·5 (2·9 to 11·7)*	1·21 (0·44 to 2·00)*	0·19 (-1·59 to 1·98)	1·85 (0·69 to 2·94)*
Equatorial Guinea	13·9 (8·9 to 19·3)	15·7 (10·4 to 21·4)	25·7 (18·7 to 34·1)	37·0 (26·6 to 48·3)	41·7 (31·1 to 52·8)	49·3 (38·3 to 62·0)	35·4 (24·4 to 47·7)*	11·8 (6·1 to 18·5)*	23·6 (13·3 to 33·8)*	4·90 (3·41 to 6·58)*	6·18 (3·18 to 9·59)*	4·11 (2·43 to 5·84)*
Gabon	27·7 (24·2 to 31·4)	28·0 (24·4 to 31·7)	28·6 (24·5 to 32·9)	30·7 (26·4 to 35·2)	34·8 (30·1 to 39·6)	40·4 (35·0 to 46·1)	12·7 (6·6 to 18·9)*	0·9 (-3·5 to 5·4)	11·8 (5·4 to 17·9)*	1·45 (0·76 to 2·13)*	0·30 (-1·35 to 1·87)	2·17 (1·01 to 3·30)*
Eastern Sub-Saharan Africa	15·0 (13·3 to 16·8)	16·2 (14·5 to 17·9)	18·8 (17·0 to 20·6)	22·9 (21·0 to 24·8)	26·0 (24·0 to 27·9)	29·2 (27·3 to 31·3)	14·2 (11·9 to 16·4)*	3·7 (1·9 to 5·5)*	10·5 (8·4 to 12·5)*	2·56 (2·11 to 3·03)*	2·22 (1·12 to 3·31)*	2·77 (2·18 to 3·34)*
Burundi	14·3 (10·7 to 18·2)	15·1 (11·4 to 18·8)	17·7 (14·2 to 21·3)	24·2 (20·3 to 28·1)	26·8 (23·0 to 30·8)	27·4 (23·1 to 32·1)	13·1 (7·3 to 18·2)*	3·4 (-0·8 to 7·5)	9·7 (4·6 to 14·5)*	2·52 (1·40 to 3·70)*	2·19 (-0·51 to 4·93)	2·73 (1·29 to 4·14)*
Comoros	19·4 (16·1 to 23·1)	20·9 (17·9 to 24·3)	23·4 (20·3 to 26·4)	27·5 (24·4 to 30·8)	31·6 (28·3 to 35·6)	33·0 (29·5 to 36·7)	13·6 (8·5 to 18·2)*	3·9 (0·5 to 7·4)*	9·6 (5·6 to 13·7)*	2·05 (1·24 to 2·82)*	1·87 (0·20 to 3·57)*	2·16 (1·25 to 3·06)*
Djibouti	23·1 (20·2 to 26·6)	23·1 (19·8 to 26·8)	24·3 (19·8 to 30·0)	27·4 (22·2 to 33·6)	30·6 (25·6 to 36·7)	35·0 (29·7 to 42·0)	11·8 (5·8 to 19·1)*	1·1 (-3·5 to 6·3)	10·7 (5·6 to 15·9)*	1·58 (0·78 to 2·41)*	0·45 (-1·58 to 2·47)	2·29 (1·22 to 3·43)*
Eritrea	12·2 (9·2 to 15·6)	17·0 (14·1 to 20·7)	20·7 (17·3 to 24·3)	23·7 (20·1 to 27·3)	24·2 (20·7 to 27·9)	27·6 (23·4 to 31·3)	15·4 (10·7 to 19·9)*	8·5 (5·1 to 12·1)*	6·9 (2·8 to 10·8)*	3·16 (2·12 to 4·28)*	5·33 (3·13 to 7·94)*	1·81 (0·71 to 2·89)*
Ethiopia	10·6 (7·8 to 14·1)	11·7 (8·9 to 14·7)	14·0 (11·1 to 17·3)	18·5 (15·5 to 21·7)	22·9 (19·2 to 26·4)	28·1 (24·3 to 32·2)	17·5 (12·2 to 22·1)*	3·5 (-0·5 to 7·2)	14·1 (9·3 to 18·9)*	3·79 (2·53 to 5·04)*	2·88 (-0·38 to 6·12)	4·36 (2·85 to 6·01)*
Kenya	32·4 (27·6 to 37·4)	31·7 (27·4 to 36·5)	32·3 (28·0 to 36·8)	35·1 (30·6 to 39·5)	37·0 (32·4 to 41·3)	39·5 (35·0 to 43·9)	7·1 (3·3 to 11·0)*	-0·1 (-3·1 to 2·6)	7·2 (4·2 to 10·2)*	0·76 (0·33 to 1·20)*	-0·03 (-0·96 to 0·82)	1·26 (0·73 to 1·81)*
Madagascar	20·6 (18·0 to 23·2)	21·9 (19·3 to 24·6)	23·8 (21·0 to 26·9)	27·8 (24·1 to 31·2)	27·5 (23·5 to 32·2)	29·6 (24·3 to 35·1)	9·0 (3·5 to 15·0)*	3·3 (0·1 to 6·6)*	5·8 (-0·1 to 11·6)	1·39 (0·57 to 2·23)*	1·47 (0·05 to 2·94)*	1·34 (-0·02 to 2·61)
Malawi	19·0 (13·9 to 25·5)	19·3 (13·3 to 27·8)	21·5 (14·8 to 31·9)	26·1 (18·7 to 34·6)	28·7 (23·1 to 36·1)	32·2 (26·9 to 38·2)	13·2 (6·3 to 20·1)*	2·5 (-2·7 to 8·9)	10·7 (1·0 to 19·3)*	2·06 (0·96 to 3·30)*	1·15 (-1·45 to 3·95)	2·63 (0·19 to 5·04)*
Mozambique	13·8 (11·0 to 17·0)	16·4 (13·0 to 20·7)	21·1 (15·9 to 28·1)	25·7 (19·0 to 32·8)	28·0 (21·8 to 34·8)	30·0 (25·3 to 35·0)	16·3 (11·2 to 21·4)*	7·3 (2·2 to 13·6)*	9·0 (2·0 to 15·2)*	3·01 (2·10 to 3·93)*	4·19 (1·50 to 7·25)*	2·27 (0·45 to 4·02)*
Rwanda	16·7 (13·0 to 20·8)	16·0 (11·5 to 21·1)	18·6 (14·4 to 22·8)	26·1 (22·5 to 30·2)	31·6 (27·9 to 35·6)	36·0 (31·6 to 40·5)	19·2 (14·1 to 24·1)*	1·8 (-2·1 to 5·6)	17·4 (12·1 to 22·7)*	2·96 (2·06 to 3·90)*	1·05 (-1·22 to 3·27)	4·16 (2·77 to 5·75)*
Somalia	12·8 (8·2 to 18·3)	12·6 (8·5 to 18·4)	13·5 (9·1 to 19·1)	14·9 (10·7 to 20·2)	16·9 (12·1 to 22·1)	19·0 (14·3 to 23·7)	6·2 (0·6 to 11·1)*	0·7 (-2·8 to 3·7)	5·5 (0·5 to 9·8)*	1·56 (0·13 to 3·01)*	0·56 (-2·17 to 3·03)	2·19 (0·19 to 4·19)*
South Sudan	22·0 (16·8 to 28·9)	21·4 (15·7 to 28·9)	23·6 (17·4 to 30·7)	26·3 (20·1 to 32·8)	26·4 (20·5 to 32·5)	26·8 (21·0 to 33·1)	4·9 (-2·0 to 11·2)	1·6 (-3·4 to 6·6)	3·3 (-2·7 to 9·0)	0·78 (-0·31 to 1·81)	0·69 (-1·51 to 2·87)	0·84 (-0·66 to 2·31)
Tanzania	21·9 (18·7 to 25·5)	22·8 (19·1 to 27·7)	24·7 (20·5 to 30·1)	28·6 (24·2 to 34·1)	31·0 (27·0 to 36·3)	33·9 (30·0 to 38·4)	11·9 (7·3 to 16·6)*	2·7 (-1·5 to 7·4)	9·2 (4·1 to 14·3)*	1·67 (1·01 to 2·35)*	1·15 (-0·68 to 2·99)	2·00 (0·83 to 3·19)*
Uganda	19·3 (15·6 to 23·5)	20·7 (16·0 to 26·2)	23·7 (20·0 to 27·7)	28·3 (24·7 to 32·1)	29·3 (25·8 to 33·1)	31·4 (27·2 to 35·6)	12·1 (7·2 to 16·8)*	4·4 (0·5 to 8·4)*	7·8 (3·2 to 12·4)*	1·89 (1·11 to 2·70)*	2·06 (0·25 to 3·95)*	1·78 (0·73 to 2·88)*
Zambia	21·9 (17·6 to 27·2)	17·6 (13·5 to 22·3)	17·2 (13·0 to 22·7)	20·4 (15·7 to 26·7)	22·7 (17·4 to 28·0)	29·0 (23·0 to 35·4)	7·1 (0·4 to 14·8)*	-4·7 (-9·2 to 0·3)	11·7 (5·0 to 19·1)*	1·08 (0·07 to 2·22)*	-2·44 (-4·83 to 0·13)	3·28 (1·32 to 5·30)*
Southern Sub-Saharan Africa	38·2 (36·3 to 40·4)	41·1 (38·3 to 44·0)	37·8 (34·8 to 40·6)	37·1 (34·2 to 39·8)	39·5 (37·0 to 42·1)	44·7 (42·4 to 47·0)	6·5 (3·8 to 9·1)*	-0·4 (-3·0 to 2·2)	7·0 (3·8 to 10·1)*	0·61 (0·34 to 0·84)*	-0·11 (-0·81 to 0·55)	1·06 (0·58 to 1·56)*
Botswana	36·5 (30·6 to 43·0)	39·7 (23·9 to 60·7)	39·7 (22·3 to 55·7)	40·1 (24·9 to 53·1)	46·6 (33·5 to 59·3)	51·5 (40·8 to 69·2)	15·0 (3·5 to 32·8)*	3·2 (-11·5 to 17·2)	11·8 (-8·6 to 34·7)	1·31 (0·32 to 2·57)*	0·54 (-4·08 to 3·84)	1·79 (-1·07 to 5·36)
Lesotho	30·3 (25·9 to 35·5)	31·1 (26·1 to 38·3)	29·2 (23·0 to 38·0)	28·4 (22·4 to 35·7)	28·9 (23·4 to 35·5)	32·0 (24·6 to 40·3)	1·6 (-6·2 to 10·0)	-1·2 (-7·8 to 7·5)	2·8 (-6·1 to 12·2)	0·19 (-0·81 to 1·17)	-0·46 (-2·73 to 2·36)	0·59 (-1·18 to 2·52)
Namibia	27·5 (24·6 to 31·1)	29·4 (25·0 to 35·4)	32·2 (24·1 to 43·3)	34·6 (26·5 to 42·4)	39·5 (32·1 to 48·1)	44·6 (36·4 to 56·2)	17·1 (9·4 to 27·7)*	4·7 (-2·9 to 15·7)	12·4 (3·2 to 20·9)*	1·84 (1·14 to 2·71)*	1·45 (-1·08 to 4·60)	2·09 (0·49 to 3·62)*
South Africa	40·1 (38·0 to 42·3)	43·7 (41·5 to 46·1)	40·9 (38·2 to 43·8)	41·1 (37·9 to 44·2)	45·2 (42·5 to 47·8)	49·7 (47·2 to 52·4)	9·6 (6·6 to 12·7)*	0·8 (-2·3 to 3·9)	8·8 (5·4 to 12·1)*	0·83 (0·57 to 1·08)*	0·19 (-0·56 to 0·95)	1·23 (0·75 to 1·70)*
Swaziland	32·0 (27·3 to 37·0)	35·8 (27·8 to 44·7)	34·4 (22·6 to 43·6)	30·6 (20·0 to 36·9)	34·5 (25·2 to 41·9)	40·5 (30·4 to 52·2)	8·5 (-1·2 to 18·4)	2·4 (-11·1 to 13·5)	6·1 (-9·6 to 20·6)	0·88 (-0·14 to 1·78)	0·59 (-3·91 to 3·86)	1·06 (-1·64 to 3·73)
Zimbabwe	37·3 (31·2 to 48·0)	39·6 (28·9 to 51·0)	31·4 (22·6 to 39·7)	27·0 (20·3 to 33·7)	26·2 (20·0 to 33·8)	31·2 (25·8 to 37·0)	-6·1 (-17·7 to 1·0)	-5·9 (-12·0 to 0·0)	-0·2 (-9·5 to 9·2)	-0·68 (-1·81 to 0·11)	-1·81 (-3·80 to 0·01)	0·02 (-1·79 to 2·04)
Western Sub-Saharan Africa	22·4 (20·3 to 24·4)	23·2 (21·0 to 25·4)	24·8 (22·4 to 27·2)	28·3 (25·8 to 30·7)	31·7 (29·3 to 34·0)	34·3 (31·9 to 36·7)	11·9 (9·2 to 14·6)*	2·4 (0·1 to 4·9)*	9·5 (6·5 to 12·6)*	1·64 (1·26 to 2·04)*	1·03 (0·05 to 2·04)*	2·02 (1·35 to 2·74)*
Benin	19·7 (16·9 to 22·7)	21·3 (18·4 to 24·5)	22·7 (19·7 to 26·0)	27·4 (24·3 to 30·5)	28·4 (25·6 to 31·4)	30·8 (27·8 to 34·0)	11·2 (7·2 to 15·2)*	3·1 (-0·1 to 6·4)	8·1 (4·3 to 11·9)*	1·74 (1·09 to 2·38)*	1·45 (-0·06 to 3·08)	1·92 (0·98 to 2·82)*
Burkina Faso	16·4 (13·4 to 20·3)	18·4 (15·1 to 22·9)	21·9 (18·7 to 25·6)	27·2 (24·1 to 30·7)	29·0 (26·0 to 32·2)	30·1 (27·0 to 33·3)	13·7 (9·2 to 17·6)*	5·6 (2·1 to 9·0)*	8·2 (4·2 to 12·1)*	2·36 (1·46 to 3·10)*	2·96 (1·07 to 4·76)*	1·99 (0·99 to 3·02)*
Cameroon	23·4 (20·6 to 26·8)	23·0 (19·5 to 27·4)	23·8 (19·7 to 28·1)	29·2 (24·9 to 33·6)	28·5 (24·1 to 32·9)	31·9 (26·9 to 37·5)	8·5 (3·3 to 14·3)*	0·4 (-3·8 to 4·5)	8·2 (2·9 to 13·3)*	1·19 (0·49 to 1·92)*	0·13 (-1·73 to 1·88)	1·85 (0·69 to 3·00)*
Cape Verde	38·1 (35·4 to 41·2)	37·6 (33·9 to 41·6)	41·4 (37·0 to 46·1)	44·8 (40·6 to 49·4)	49·4 (46·1 to 53·0)	54·8 (51·2 to 58·9)	16·7 (12·5 to 21·2)*	3·3 (-1·3 to 7·8)	13·4 (7·6 to 19·5)*	1·40 (1·03 to 1·76)*	0·81 (-0·34 to 1·91)	1·76 (0·99 to 2·61)*
Chad	18·3 (15·6 to 21·4)	19·3 (16·4 to 22·4)	18·2 (15·3 to 21·5)	20·4 (17·0 to 24·3)	22·3 (19·2 to 25·7)	25·4 (21·9 to 29·0)	7·1 (2·8 to 11·6)*	-0·1 (-3·5 to 3·3)	7·2 (3·2 to 11·1)*	1·27 (0·49 to 2·05)*	-0·05 (-1·92 to 1·81)	2·09 (0·93 to 3·27)*

***Supplementary table 3.* Global, regional, national or territory, and select subnational estimates on the HAQ Index for 1990–2016, and absolute change and annualised rates of change for 1990–2016, 1990–2000, and 2000–2016.** Starred estimates of change represent statistically significant changes during a given time period. HAQ Index=Healthcare Access and Quality Index.

	HAQ Index						Absolute change			Annualised rate of change		
	1990	1995	2000	2005	2010	2016	1990-2016	1990-2000	2000-2016	1990-2016	1990-2000	2000-2016
Cote d'Ivoire	19·9 (17·3 to 22·6)	19·4 (16·3 to 22·9)	20·7 (17·1 to 24·3)	23·2 (19·7 to 26·9)	26·0 (22·6 to 29·8)	27·3 (24·2 to 31·1)	7·5 (3·3 to 11·1)*	0·8 (-2·5 to 4·3)	6·7 (2·5 to 10·8)*	1·23 (0·56 to 1·83)*	0·37 (-1·29 to 2·05)	1·76 (0·61 to 2·95)*
Ghana	25·6 (22·5 to 28·9)	27·8 (24·6 to 31·6)	29·6 (26·2 to 33·5)	33·0 (29·5 to 37·1)	34·6 (31·3 to 38·3)	39·3 (36·0 to 43·4)	13·6 (9·1 to 18·4)*	4·0 (0·1 to 8·0)*	9·7 (5·1 to 14·1)*	1·64 (1·08 to 2·25)*	1·45 (0·04 to 2·82)*	1·77 (0·93 to 2·62)*
Guinea	17·1 (14·3 to 20·3)	18·2 (15·6 to 21·1)	20·1 (17·2 to 23·0)	23·5 (20·2 to 26·7)	23·9 (20·6 to 27·7)	26·4 (22·6 to 30·2)	9·2 (4·5 to 14·2)*	2·9 (-0·6 to 6·3)	6·3 (2·2 to 10·8)*	1·66 (0·80 to 2·54)*	1·58 (-0·31 to 3·49)	1·71 (0·62 to 2·85)*
Guinea-Bissau	12·8 (10·0 to 16·0)	14·3 (11·5 to 17·2)	15·7 (12·7 to 19·0)	17·3 (14·2 to 21·4)	20·5 (17·2 to 24·3)	23·4 (20·2 to 26·8)	10·6 (5·9 to 14·9)*	2·9 (-0·6 to 6·7)	7·7 (3·6 to 11·9)*	2·34 (1·25 to 3·36)*	2·03 (-0·40 to 4·67)	2·53 (1·17 to 3·97)*
Liberia	20·5 (17·6 to 23·6)	21·0 (18·1 to 24·3)	23·2 (19·8 to 26·8)	29·3 (25·8 to 33·1)	30·7 (27·6 to 34·0)	32·2 (29·3 to 35·4)	11·7 (8·0 to 15·5)*	2·8 (-0·7 to 6·6)	8·9 (4·9 to 13·0)*	1·74 (1·15 to 2·36)*	1·26 (-0·31 to 2·98)	2·04 (1·09 to 3·07)*
Mali	16·7 (13·7 to 20·5)	19·4 (16·3 to 22·5)	23·7 (20·4 to 27·2)	29·9 (26·1 to 34·2)	30·7 (26·6 to 35·5)	34·9 (29·9 to 40·1)	18·2 (12·6 to 23·8)*	7·0 (2·9 to 10·6)*	11·2 (5·8 to 16·7)*	2·85 (1·97 to 3·68)*	3·53 (1·38 to 5·44)*	2·43 (1·30 to 3·59)*
Mauritania	24·0 (20·8 to 27·5)	26·1 (22·9 to 31·9)	29·7 (25·9 to 36·2)	32·9 (28·6 to 39·9)	35·7 (31·1 to 42·3)	40·6 (35·0 to 47·5)	16·6 (10·7 to 23·7)*	5·7 (1·8 to 11·5)*	10·9 (4·9 to 17·2)*	2·02 (1·35 to 2·70)*	2·13 (0·68 to 3·91)*	1·95 (0·86 to 2·98)*
Niger	15·6 (12·6 to 19·3)	17·0 (14·0 to 20·5)	19·1 (16·0 to 22·3)	23·4 (20·0 to 27·1)	26·4 (22·4 to 30·4)	28·4 (23·9 to 33·1)	12·8 (7·2 to 18·1)*	3·5 (-0·1 to 7·4)	9·3 (3·8 to 14·7)*	2·30 (1·27 to 3·24)*	2·02 (-0·06 to 4·21)	2·48 (1·06 to 3·87)*
Nigeria	27·5 (23·4 to 31·6)	27·9 (23·8 to 32·7)	29·8 (24·9 to 35·3)	33·7 (28·9 to 39·5)	38·9 (33·8 to 44·3)	41·9 (37·2 to 47·3)	14·4 (8·7 to 20·4)*	2·3 (-2·6 to 7·7)	12·1 (5·5 to 19·0)*	1·62 (0·97 to 2·35)*	0·80 (-0·92 to 2·66)	2·14 (0·93 to 3·38)*
Sao Tome and Principe	25·9 (22·4 to 29·7)	27·5 (23·2 to 37·5)	30·0 (25·7 to 40·5)	31·8 (27·6 to 41·9)	36·7 (32·3 to 45·6)	39·3 (34·9 to 44·4)	13·4 (8·2 to 19·2)*	4·2 (-0·1 to 13·3)	9·3 (-0·4 to 14·9)	1·61 (0·97 to 2·28)*	1·46 (-0·05 to 4·06)	1·71 (-0·06 to 2·74)
Senegal	22·4 (19·8 to 25·1)	23·6 (21·1 to 26·3)	24·5 (22·0 to 27·3)	26·6 (24·2 to 29·3)	28·1 (25·5 to 30·9)	31·1 (28·3 to 33·8)	8·6 (5·3 to 11·9)*	2·0 (-0·8 to 5·1)	6·6 (3·4 to 9·7)*	1·26 (0·75 to 1·77)*	0·87 (-0·33 to 2·21)	1·49 (0·73 to 2·21)*
Sierra Leone	20·8 (17·4 to 24·6)	21·8 (18·6 to 25·5)	22·1 (19·0 to 25·5)	26·3 (23·0 to 29·7)	27·5 (24·1 to 30·8)	31·0 (27·4 to 34·5)	10·1 (5·7 to 14·5)*	1·3 (-2·0 to 4·6)	8·8 (4·3 to 12·8)*	1·53 (0·84 to 2·22)*	0·60 (-0·94 to 2·25)	2·11 (1·05 to 3·10)*
The Gambia	27·4 (23·9 to 30·9)	28·5 (25·3 to 31·8)	29·9 (26·5 to 33·4)	32·5 (29·1 to 35·8)	33·5 (30·2 to 37·1)	35·7 (32·3 to 39·3)	8·3 (4·1 to 12·9)*	2·5 (-0·7 to 5·6)	5·8 (1·9 to 9·6)*	1·02 (0·50 to 1·58)*	0·87 (-0·24 to 2·02)	1·12 (0·38 to 1·82)*
Togo	21·7 (19·0 to 24·5)	21·7 (18·8 to 24·9)	23·0 (19·1 to 27·9)	27·6 (23·7 to 32·4)	28·2 (24·8 to 32·0)	32·0 (28·7 to 35·6)	10·2 (6·3 to 14·3)*	1·2 (-3·0 to 6·0)	9·0 (4·0 to 13·6)*	1·48 (0·89 to 2·09)*	0·52 (-1·46 to 2·53)	2·09 (0·85 to 3·24)*

Supplementary table 4. **Associations between potential correlates of HAQ Index performance.** All Pearson correlations with HAQ Index performance are for year 2016. Unless otherwise specified, we used the same year for indicators compared to the HAQ Index. For physicians, nurses, and midwives per 1,000, which is sourced by the WHO, we used the latest estimates reported by location from 2010-2015. HAQ Index=Healthcare Access and Quality Index. GBD=Global Burden of Disease. WHO=World Health Organization. DAH=Development assistance for health.

	Source and year	Locations represented	Correlation with the HAQ Index in 2016
**Health financing and development**			
Log of cumulative total health spending per capita, 2010-2015	GBD 2017	188	0·94
Log of cumulative out-of-pocket spending per capita, 2010-2015	GBD 2017	188	0·85
Log of cumulative government health spending per capita, 2010-2015	GBD 2017	188	0·93
Log of cumulative DAH per capita, 2010-2016	GBD 2017	142	-0·59
Cumulative out-of-pocket spending as a percent of total health spending, 2010-2015 (%)	GBD 2017	188	-0·31
Cumulative government health spending as a percent of total health spending, 2010-2015 (%)	GBD 2017	188	0·76
Cumulative DAH as a percent of total health spending, 2010-2016 (%)	GBD 2017	142	-0·71
Socio-demographic Index (SDI)	GBD 2016	195	0·94
**Health system inputs and outputs**			
Physicians, nurses, and midwives per 1,000	WHO 2017	120	0·79
Hospital beds per 1,000	GBD 2016	195	0·63
Outpatient utilisation	GBD 2016	195	0·56
Inpatient utilisation	GBD 2016	195	0·64
